# Desymmetrization of *N*-Cbz glutarimides through N-heterocyclic carbene organocatalysis

**DOI:** 10.1038/s41467-022-31760-z

**Published:** 2022-07-13

**Authors:** Zhouli Hu, Chenlong Wei, Qianqian Shi, Xianfang Hong, Jinhua Liu, Xiangui Zhou, Jinna Han, Wei Cao, Ashis Kumar Gupta, Xiaoxiang Zhang, Donghui Wei, Zhenqian Fu, Wei Huang

**Affiliations:** 1 Key Laboratory of Flexible Electronics & Institute of Advanced Materials, Nanjing Tech University, 30 South Puzhu Road, Nanjing, 211816 China; 2 College of Chemistry, Zhengzhou University, 100 Science Avenue, Zhengzhou, Henan Province 450001 China; 3 Jiangsu Co-Innovation Center of Efficient Processing and Utilization of Forest Resources, College of Chemical Engineering, Nanjing Forestry University, Nanjing, 210037 China; 4 Ningbo Institute, Chongqing Technology Innovation Center, Frontiers Science Center for Flexible Electronics (FSCFE), Northwestern Polytechnical University, 127 West Youyi Road, Xi’an, 710072 China

**Keywords:** Organocatalysis, Synthetic chemistry methodology

## Abstract

Over the past decade, the catalysis of *N*-heterocyclic carbenes has achieved significant advances. In this area, aldehydes, enals, and esters, are commonly employed as starting materials through various catalytic activation modes. However, NHC-activated strategy of amide and its derivatives remains elusive. Described herein is the realization of asymmetric desymmetrization of N-Cbz glutarimides with alcohols through an imide C-N bond cleavage under NHC organocatalysis. A structurally diverse set of enantioenriched 4-amido esters is generated with acceptable yields and high enantioselectivities. This method features mild reaction conditions, excellent substrate scope, and excellent atom economy. DFT calculations have been performed to explore the detailed reaction mechanism and the origin of the enantioselectivity, which indicate that the strength of the C-H···O hydrogen bond and C–H⋯π interactions should be responsible for the stereoselectivity. The current strategy could open a door for efficient construction of (*R*)-Rolipram with excellent stereoselectivity.

## Abstract (web summary)

Desymmetrization of achiral building blocks is one of the most efficient ways to access enantiopure compounds of synthetic relevance. Here, the authors desymmetrize glutarimides with alcohols via an imide C–N bond cleavage under NHC organocatalysis.

## Introduction

**Fig. 1:**
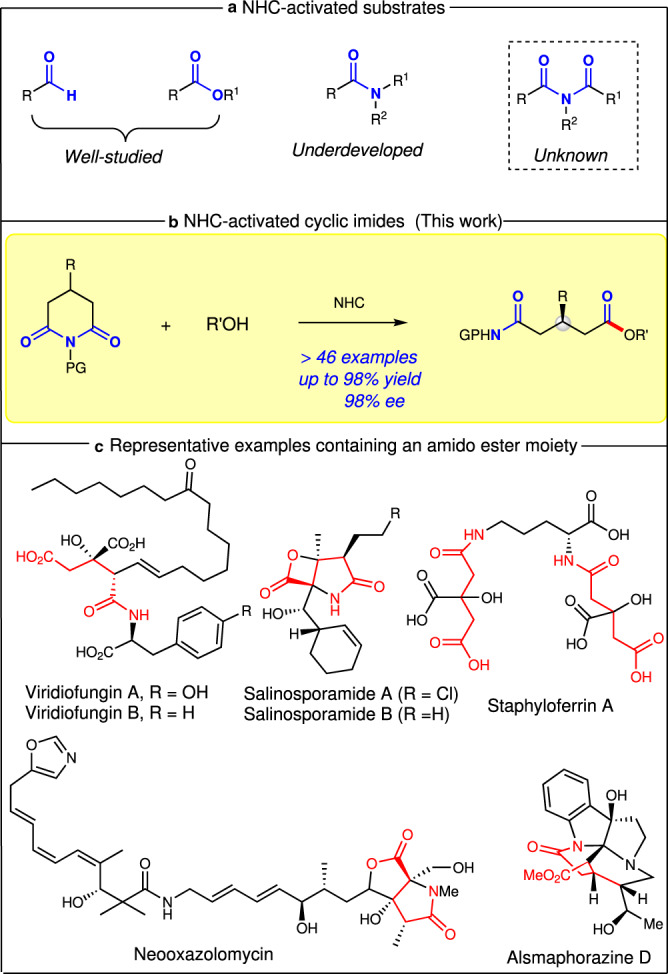
NHC-activated substrates and our strategy. **a** NHC-activated substrates. **b** NHC-activated cyclic imides. **c** Representative compounds containing an amido ester moiety.

Over the past decade, N-heterocyclic carbene (NHC) catalysis has proven to be one of the most privileged organocatalytic methods. It enables the construction of a structurally diverse set of synthetically valuable scaffolds from simple raw materials^1–5^. Since Breslow intermediate was revealed^6^, umpolung reactions of aldehydes under NHC catalysis have been frequently investigated, most notably on benzoin condensations^7–11^ and Stetter reactions^12–15^. The Glorius^16^ and Bode^17^ groups independently realized the seminal discovery that NHCs could further reverse the reactivity of β-carbon of enals to form homoenolate intermediates^18–20^, which greatly promoted the development of NHC chemistry. Subsequently, the Studer group^21^ reported in a pioneering work that NHCs could activate enals to generate α, β-unsaturated acyl azoliums^22–24^ under oxidative conditions. In addition to aldehydes (or enals), stable and relatively less reactive esters could also be activated creatively by NHCs, which was discovered by the groups of Chi and Lupton^25–28^, significantly expanding the scope of NHC chemistry. In addition, α-activation (enolate intermediates)^29–33^ and remote activation (such as γ^34–37^, δ^38,39^, and ε-activation^40,41^) of the aforementioned substrates, have also been achieved significantly under NHC catalysis. Beside commonly classical two-electron pathways, NHC-catalyzed radical reactions via a single electron transfer (SET) pathway of aldehydes or esters, and their surrogates have also received continuous attention^42–46^. Besides the aforementioned use of NHC as Lewis base catalyst, the asymmetric Bronsted base catalysis with NHC has also made impressive advances, in which NHC can activate 1,3-dicarbonyl compounds, amines, and thiols to realize asymmetric reactions via noncovalent interactions^47–51^. In sharp contrast to the aforementioned well-developed NHC-activation of aldehydes, enals, and esters, NHC-activation of valuable amide and its derivatives has been largely underdeveloped (Fig. 1a).

Amide bonds are not only essential functional groups in organic chemistry but also fundamental units of polypeptides and proteins. Direct activation of an amide bond is very challenging due to the high stability of the amide linkage. Notably, NHC-catalyzed amide C–N bond activation remains to be established. As a part of our ongoing interest in organocatalysis^52–56^, we envisioned the imides, containing a more reactive amide bond and unknown in the area of NHC, might be activated by NHC and further realize asymmetric reactions^57–59^. If successful, this methodology will expand the scope of carbene chemistry. Specifically, easily available cyclic imides were selected to investigate their asymmetric desymmetrization via amide bond activation by NHCs^36,60–63^. The success of the current strategy is determined by two key issues, namely: (i) increasing the reactivity of the imides to ensure C–N imide cleavage under NHC catalysis; (ii) both avoiding the background reaction and controlling the reaction conditions to ensure excellent enantioselectivities. Notably, the chiral center is far away from the reactive site, which makes enantiocontrol difficult.

Herein, we present the successful development of a strategy for NHC-catalyzed desymmetrization of cyclic imides with alcohols via an imide C–N bond cleavage under mild conditions (Fig. 1b). Notably, the resulting amido esters as key motifs are frequently found in natural products associated with a variety of promising biological activities (Fig. 1c). Importantly, the resulting product could be efficiently converted into (*R*)-Rolipram.

## Results

### Reaction optimization

Initial attempts were performed with prochiral cyclic imide **1a** or *N*-Bn cyclic imide **1b**, and ethanol **2a**, one of the simplest alcohols, in the presence of the achiral triazolium N-Mes pre-catalyst **A** with K_2_CO_3_ as the base in DCM at room temperature. Unfortunately, no reaction occurred, probably because of the insufficient reactivity of the imide C–N bond (entries 1–2). Next, prochiral *N*-Cbz **1c** (Cbz: an electron-withdrawing and good leaving group), was investigated under the same reaction conditions. To our delight, the desired ethyl 5-(((benzyloxy)carbonyl)amino)-5-oxo-3-phenylpentanoate **3a** was obtained in 75% yield.

Gratifyingly, the desired enantioenriched **3a**, with much structural difference of the remote third atom from the chiral center, was obtained in 78% yield with 84% ee when the aminoindanol-derived triazolium pre-catalyst **B** was used. This result confirmed the feasibility of NHC-activation of the imide C–N bond and catalytic asymmetric desymmetrization of prochiral cyclic imides. Next, several bases were investigated, with the inorganic base K_2_CO_3_ proving to be the best choice (entries 3–12). Subsequent solvent screening revealed that DCM was the most effective (entries 13–16). Lowering the temperature could improve the enantioselectivity considerably (entries 17–19). The product **3a** was obtained in 85% yield with 95% ee when the temperature was dropped to −30 °C. Reducing the catalyst loading led to a decrease in the yield and enantioselectivity considerably (entries 20 and 21). Low enantioselectivity (9% ee) was observed when the NHC **C** was employed (entry 22). In the absence of the catalyst, no reaction occurred (entry 23). The absolute configuration of products **3** was determined via X-ray of **3i**.

### Substrate scope

**Fig. 2:**
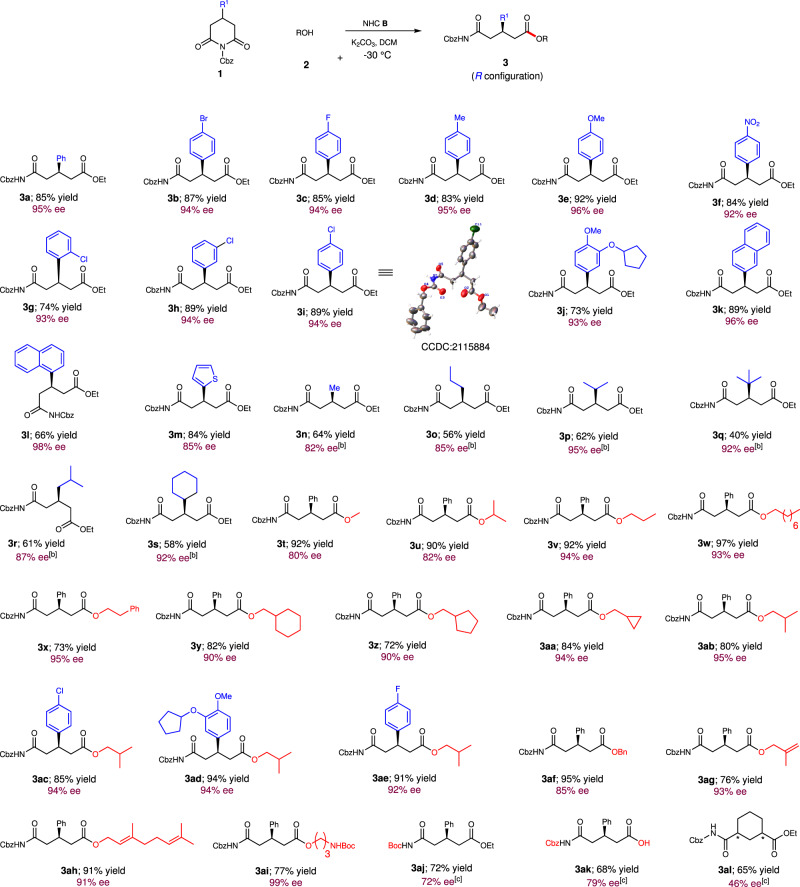
Substrate scope with respective to 4-substituted glutarimides. Reaction conditions as in Table 1, entry 19; yields (after SiO_2_ chromatography purification) were based on **1**. ^[b]^ Reaction was performed at −40 °C. ^[c]^ Reaction was performed at 30 °C.

With acceptable optimized conditions in hand (Table 1, entry 19), we then investigated the scope of the reaction for prochiral cyclic imide substrates by using ethanol **2a** as a model substrate (Fig. 2). R^1^ groups bearing substituents with a variety of electronic and steric properties on the aromatic ring were well tolerated, which resulted in the formation of the corresponding products **3b**-**3j** in good to excellent yields (73–92%) with excellent enantioselectivities (92–95% ees). Substrates with both 1-naphthyl and 2-naphthyl groups reacted smoothly to deliver the products **3k** and **3l**, respectively, with excellent enantioselectivities. The 2-thienyl group is well tolerated, yielding the product **3m** in 84% yield with 85% ee. Notably, alkyl groups, such as Me, *n*- Pr, *i*-Pr, *t*-Bu, *i*-Bu, and Cy, were efficient substrates for this reaction to provide the products **3n-3s** with good to excellent enantioselectivities (82–95% ee values). To demonstrate the generality of this catalytic asymmetric desymmetrization, the scope of the alcohols was further investigated (Fig. 2). Besides ethanol, other alkyl alcohols, such as MeOH, *i*-PrOH, *n*-PrOH, phenethyl alcohol, 1-octanol, i-BuOH, cyclohexylmethanol, cyclopentylmethanol, cyclopropylmethanol, and benzyl alcohol worked efficiently to access the desired products **3t-3af** with good to excellent enantioselectivities (80–97% ee). Pleasingly, several other functionalized groups, such as terminal alkenyl, double bond, and even NHBoc groups were well-tolerated, affording the products **3ag**-**3ai** in good to excellent yields (76–91%) with excellent enantioselectivities (91–98% ee). The protective group on the imide moiety was investigated; when Cbz was replaced with Boc, no reaction occurred under the optimized conditions, perhaps due to the greater steric hindrance of the Boc group. To our delight, raising the temperature to 30°C afforded the *N*-Boc product **3ad** in 72% yield with 71% ee. Notably, in addition to alcohols, H_2_O is also compatible with this transformation to form **3ak** in 68% yield with 79% ee. Unfortunately, other nucleophiles, such as benzyl mercaptan, and amine, did not give satisfying outcomes under the current conditions (see SI for details). Delightingly, cyclic imides with 3,5-substituents, such as benzyl 2,4-dioxo-3-azabicyclo[3.3.1]nonane-3-carboxylate, reacted smoothly to give the desired product **3al** in 65% yield with 46% ee. Mild reaction conditions, excellent functional group tolerance, and broad substrate scope significantly increased the utility of this method for further synthetic transformations.

**Table 1:** Condition optimization.

Entry^a^	NHC	Base	Solvent	Yield (%)^b^	ee (%)^c^
1^d^	**A**	K_2_CO_3_	DCM	nr	–
2^e^	**A**	K_2_CO_3_	DCM	nr	–
3	**A**	K_2_CO_3_	DCM	75	–
4	**B**	K_2_CO_3_	DCM	78	84
5	**B**	Cs_2_CO_3_	DCM	72	79
6	**B**	DBU	DCM	70	22
7	**B**	NaOAc	DCM	Trace	–
8	**B**	Et_3_N	DCM	Trace	–
9	**B**	DIPEA	DCM	Trace	–
10	**B**	DABCO	DCM	77	84
11	**B**	KHCO_3_	DCM	81	83
12	**B**	K_3_PO_4_	DCM	52	79
13	**B**	K_2_CO_3_	THF	Trace	–
14	**B**	K_2_CO_3_	DCE	63	87
15	**B**	K_2_CO_3_	CHCl_3_	75	69
16	**B**	K_2_CO_3_	1,4-Dioxane	Trace	–
17^f^	**B**	K_2_CO_3_	DCM	80	89
18^g^	**B**	K_2_CO_3_	DCM	89	93
19^h^	**B**	K_2_CO_3_	DCM	85	95
20^h,i^	**B**	K_2_CO_3_	DCM	82	93
21^h,j^	**B**	K_2_CO_3_	DCM	80	92
22^h^	**C**	K_2_CO_3_	DCM	84	9
23^h^	–	K_2_CO_3_	DCM	nr	–

*nr* no reaction.

^a^Standard condition: **1c** (0.1 mmol), **2a** (1.5 equiv.), NHC precursor (20 mol %), base (1.5 equiv.), solvent (0.1 M), 30 °C, N_2_, 12–72 h.

^b^Yield (after SiO_2_ chromatography purification) were based on **1a**.

^c^Determined via chiral phase HPLC analysis.

^d^**1a** was used.

^e^**1b** was used.

^f^0 °C.

^g^−20 °C.

^h^−30 °C.

^i^NHC precursor (15 mol %).

^j^NHC precursor (10 mol %).

**Fig. 3:**
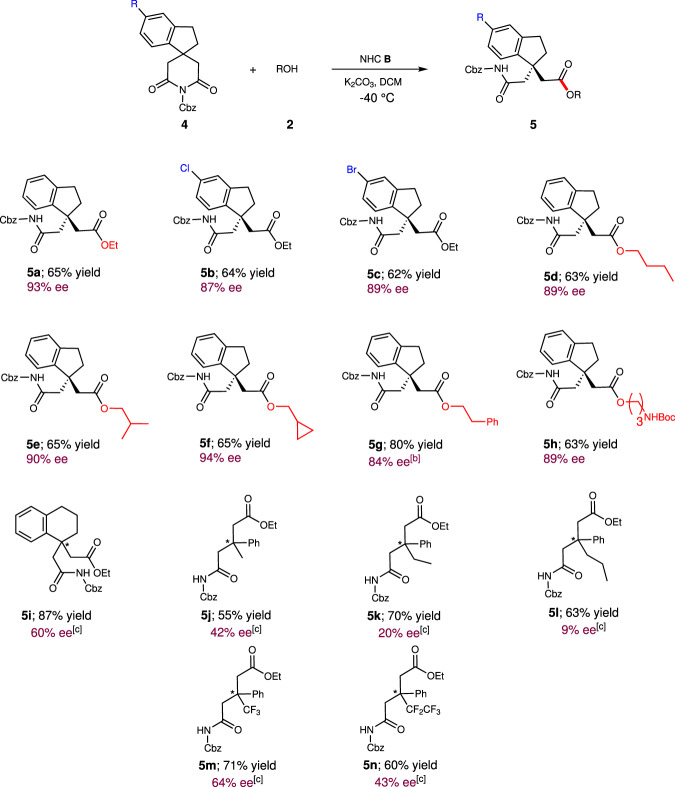
Substrate scope with respective to 4,4-disubstituted glutarimides. Reaction conditions as in Table 1, entry 19; yields (after SiO_2_ chromatography purification) were based on **4**. ^[b]^ Reaction was performed at −30 °C. ^[c]^ NHC precursor **C** was used. Reaction was performed at −30 °C.

After the establishment of this catalytic desymmetrization strategy, 4,4-disubstituted glutarimides **4** was further tested (Fig. 3). A variety of 4,4-disubstituted glutarimides were investigated, and 2,3-dihydrospiro[indene-1,4'-piperidine]-2',6'-diones were identified as the best skeleton to give the desired products **5a-5h** in 62–80% yields with 84–94% ee values. The absolute configruation of the product **5** was detemined via X-ray of **10**, which was derived from the product **6b**. However, other frameworks, such as tetrahydronaphthalenyl or acyclic structures could not give the desired products when NHC **B** was used. Instead, decarbobenzoxylation product was observed. Then, although several reaction conditions have been profoundly investigated (For details, see Supporting Information), products **5j-5o** were obtained in 55–87% yields with 9–66% ee. The obtained results indicated that NHC **B** has proven to be the more effective for 4-substituted glutarimides and indenyl glutarimides, and NHC **C** has proven to be the more effective for other 4,4-disubstituted glutarimides in most cases, probably due to more steric hindrance of 4,4-disubstituted glutarimides. Furthermore, prochiral center far away from the reaction site and similar two substituted groups on the prochiral center led to difficulty realize considerable enantiomeric control for 4,4-disubstituted glutarimides.

### Mechanistic studies

**Fig. 4:**
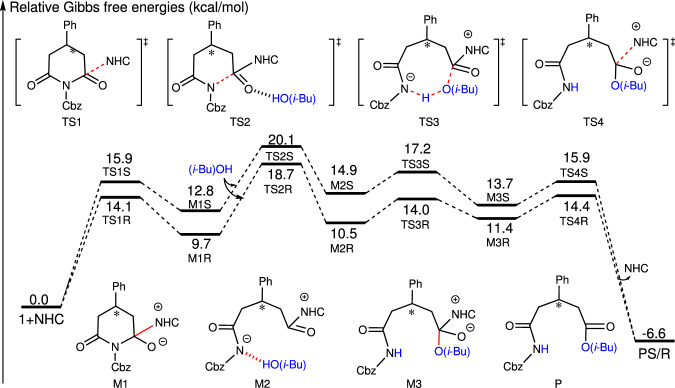
Energy profiles of the NHC B catalyzed stereoselective reaction pathways. All the energies were computed at the M06-2X/6-311 + + G(2d, 2p)/IEF-PCM_DCM/_/M06-2X/6-31 G(d, p)/IEF-PCM_DCM_ level of theory.

To shed light on the mechanism and origin of stereoselectivity for these efficient desymmetrizations of cyclimides in an excellent enantioselecitivity manner, we have DFT calculations by using Gaussian program^64^, and more computational details can be found in Supporting Information. As shown in Fig. 4, four steps including nucleophilic addition via transition states TS1R (14.1 kcal/mol) and TS1S (15.9 kcal/mol), which is followed by C–N bond cleavage via transition states TS2R (18.7 kcal/mol) and TS2S (20.1 kcal/mol). Subsequently, nucleophilic addition coupled with a proton transfer undergoes transition states TS3R (14.0 kcal/mol) and TS3S (17.2 kcal/mol). The final step is the dissociation of NHC with products via transition states

TS4R (14.4 kcal/mol) and TS4S (15.9 kcal/mol). The *R*-isomer pathway locates below the *S*-isomer pathway in the Gibbs free energy file. Particularly, the energy of TS3R is 2.8 kcal/mol lower than that of TS3S, indicating the pathway associated with *R*-isomer should be more energetically favorable in kinetics. This conclusion is in agreement with the experimental results.

**Fig. 5:**
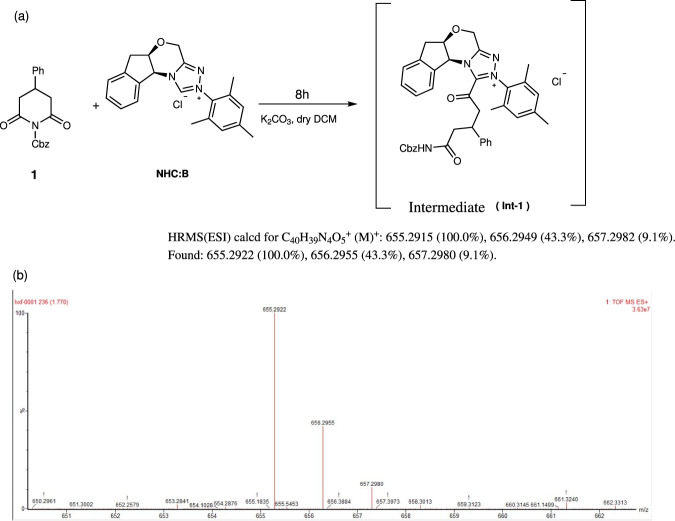
Mechanistic studies. **a** Control experiment and **b** HRMS for **Int-1**.

To probe the reaction pathway, control experiment was performed as shown in Fig. 5. Treatment of **1c** with 1.0 equiv. of NHC **B** was performed in the presence of K_2_CO_3_ in DCM for 8 h. Immediately, the high-resolution mass spectroscopy (HRMS-ESI) analysis of the reaction mixture was carried out. And one signal peak was detected at *m/z* 655.2922. M^+^ value combined with the isotope distribution pattern on HRMS analysis of the NHC-substrate adduct which probably corresponded to the intermediate **Int-1**.

**Fig. 6:**
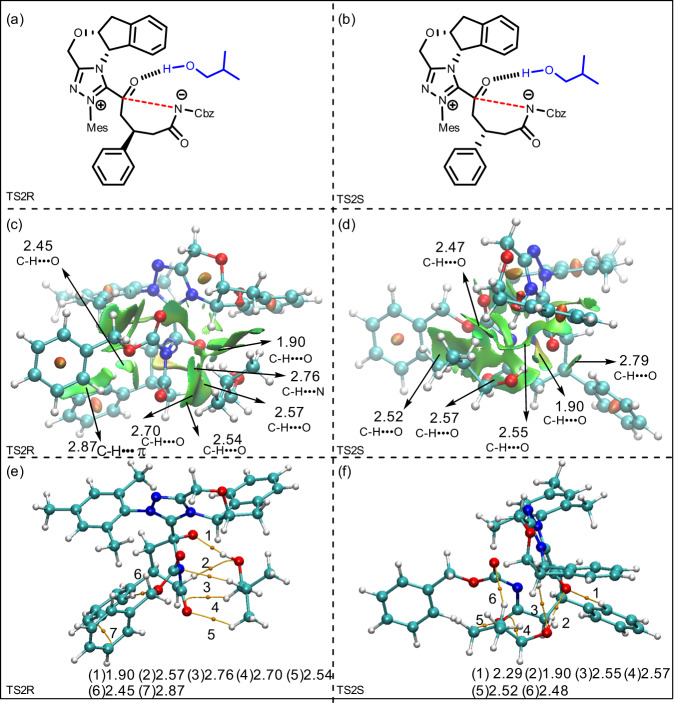
Theoretical analysis on origin of stereoselectivity. Optmized structures of transition states TS2R (**a**) and TS2S (**b**), NCI analyses for transition states TS2R (**c**) and TS2S (**d**), and AIM analyses for transition states TS2R (**e**) and TS2S (**f**) (unit: Å).

To reveal the origin and determining factor of enantioselectivity, the energy decomposition has been performed by using PSI4^65,66^. The energy decomposition analysis summarized in Table S2 of SI indicates that the interim dispersion should be responsible for the favorability of *R*-isomer pathway via TS2R. Non-covalent interaction (NCI) and atoms-in-molecules (AIM) analyses have been performed by using Multiwfn^67^, and the results have been shown in Fig. 6. As depicted in Fig. 6, there are five C–H⋯O (1.90, 2.57, 2.54 2.70, and 2.45 Å), one C–H⋯π (2.87 Å), and one C–H⋯N (2.76 Å) interactions in TS2R, while there are six C–H⋯O (2.79, 1.90, 2.55, 2.57, 2.52, and 2.48 Å) interactions in TS2S, indicating the strength of C–H⋯π and C–H⋯N hydrogen-bond interactions should be responsible for the favorability of *R*-isomer pathway. Inspired by the good examples^68–70^, we have also additionally performed the NCI analysis by employing NCIplot^71,72^, and similar weak interactions have been identified and depicted in the Supplementary Fig. 301 of SI. More computational details and results can be found in Supporting Information.

### Synthetic transformations

**Fig. 7:**
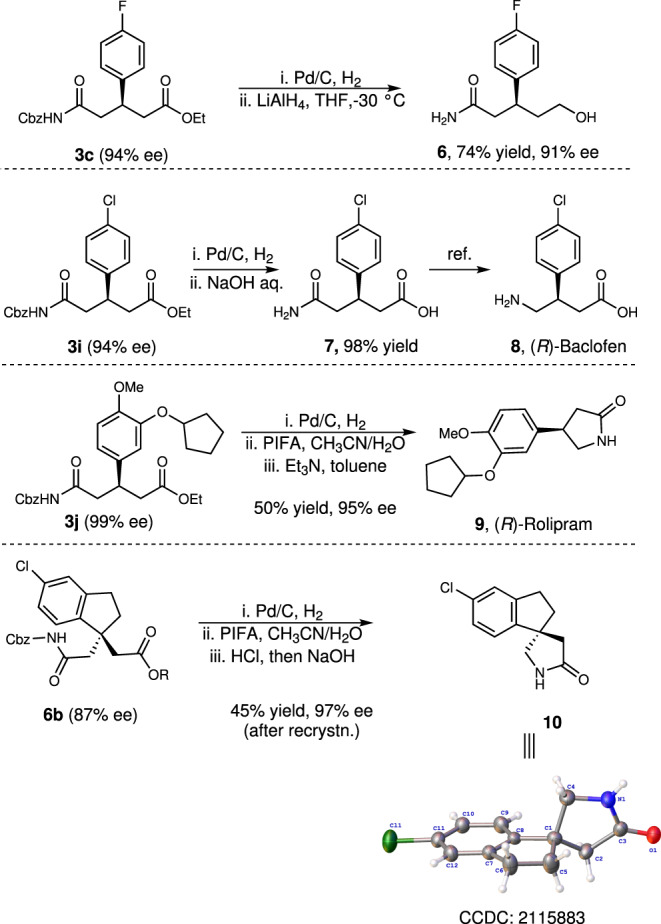
Synthetic transformations. **a** Conversion of **3c** to hydroxyl amide **6**. **b** The synthesis of (*R*)-Baclofen **8** from **3i. c** The synthesis of (*R*)-Rolipram **9** from **3j. d** Transformation of **6b** to lactam **10**.

Subsequently, further synthetic transformations of the resulting products were conducted as shown in Fig. 7. Treatment of **3c** with Pd/C under 1 atm H_2_, followed by the reduction with LiAlH_4_ furnished product **6** in 74% yield with 91% ee. Especially, **3i** underwent decarbobenzyloxylation followed by hydrolysis to result in the formation of **7** in excellent yield, which was then converted into the skeletal muscular relaxants (*R*)-Baclofen **8** via a known process^73^. More importantly, (*R*)-Rolipram **9**, which is a phosphodiesterase inhibitor with antidepressant properties^74^, was prepared in 50% overall yields with 95% ee from **3j** via a stepwise sequence of decarbobenzyloxylation, decarbonylation of the amide moiety and an intramolecular lactamization. The compound **6b** was smoothly converted into product **10** in 45% yield with 97% ee after recrystallization by using a similar strategy.

## Discussion

In summary, we have developed an NHC-enabled desymmetrization of N-Cbz glutarimides with alcohols under mild conditions via imide C–N bond activation. A diverse set of enantioenriched 4-amido esters was obtained in good yields with good to excellent enantioselectivities. This reaction features mild reaction conditions, wide substrate scope, and an excellent atom economy. DFT calculations and NCI analysis indicate that the C–H⋯O hydrogen bond and C–H⋯π interactions between the NHC catalyst and substrate should be the key for controlling the enantioselectivity. Importantly, the resulting product was converted into (*R*)-Rolipram in 50% overall yields with 95% ee. In summary, this case work combined with experimental and theoretical studies should be valuable for understanding the similar organocatalytic desymmetrization reactions of cyclic imides with imide C–N bond activation. Further investigations and exploration of this catalytic process are underway in our laboratory.

## Methods

### General procedure for the synthesis of 3

To a suspension of starting materials **1** (0.10 mmol), catalyst **B** (7.3 mg, 20 mol%) and K2CO3 (0.15 mmol, 20.7 mg) in DCM (1.0 mL, 0.10 M) was added the appropriate alcohols (0.15 mmol, 1.5 equiv.). The mixture was stirred for 72 h at −30 °C. Upon completion of the reaction (monitored by TLC), the reaction mixture was directly purified by column chromatography on silica gel to afford the desired product **3**.

## Supplementary information

Supplementary Information

Description of Additional Supplementary Files

Supplementary Data 1

## Data Availability

The X-ray crystallographic coordinates for structures of **3i** and **10** reported in this study have been deposited in the Cambridge Crystallographic Data Centre (CCDC) under deposition numbers CCDC 2115884(**3i**), and 2115883(**10**). These data can be obtained free of charge from http://www.ccdc.cam.ac.uk/data_request/cif. The experimental procedures, characterization of the new compounds, and  computational studies in this study are provided in Supplementary Information and Supplementary Data 1. All other data are available from the authors upon request.
