# Supplementary material for: Desymmetrization of N-Cbz glutarimides through N-heterocyclic carbene organocatalysis
Source: Nat Commun. 2022 Jul 13;13:4042. doi: 10.1038/s41467-022-31760-z (PMC9279320; doi:10.1038/s41467-022-31760-z)
Supplement: Supplementary file 1 — Supplementary Information [file 41467_2022_31760_MOESM1_ESM.pdf]

Supplementary Information

Asymmetric Desymmetrization of N-Cbz Glutarimides through N-Heterocyclic Carbene Organocatalysis

Zhouli Hu, Chenlong Wei, Qianqian Shi, Xianfang Hong, Jinhua Liu, Xiangui Zhou, Jinna Han, Wei Cao, Ashis Kumar Gupta, Xiaoxiang Zhang, Donghui Wei,* Zhenqian Fu* and Wei Huang

**I**	**Supplementary Methods**
1	General information
2	a) Optimal condition<br>b) Additional reactions<br>c) General procedure for the catalytic reactions of **1** and alcohols **2** to synthesize products **3**<br>d) Mechanistic studies<br>e) Synthetic transformations
3	a) Characterization data of substrates<br>b) Characterization data of products
4	^{1}H, ^{13}C NMR and HPLC data
5	Computational details
6	Conformational search
7	Comparisons of the relative Gibbs free energies for selected stationary points at different computational levels
8	Energy decomposition analyses for key transition states TS2R and TS2S
9	NCI anayses for key transition states TS2R and TS2S by using NCIplot
**II**	**Supplementary References**

I. Supplementary Methods

1. General Information

Commercially available materials purchased from Adamas-beta® and Energy Chemical were used as received. Toluene and DCM were dried over Pure Solv solvent purification system. THF was distilled over sodium. Other solvents were dried over 4Å molecular sieve prior use. Proton nuclear magnetic resonance (^{1}H NMR) spectra were recorded on a Bruker (400 MHz) spectrometer. Chemical shifts were recorded in parts per million (ppm, δ) relative to tetramethylsilane (δ 0.00) or chloroform (δ = 7.26, singlet). ^{1}H NMR splitting patterns are designated as singlet (s), doublet (d), triplet (t), quartet (q), dd (doublet of doublets); m (multiplets), and etc. All first-order splitting patterns were assigned on the basis of the appearance of the multiplet. Splitting patterns that could not be easily interpreted are designated as multiplet (m) or broad (br). Carbon nuclear magnetic resonance (^{13}C NMR) spectra were recorded on a Bruker (400 MHz) (100 MHz) spectrometer. Fourier transform infrared spectroscopy (FT-IR, a Bruker model VECTOR-22 Fourier transform spectrometer). High resolution mass spectral analysis (HRMS) was performed on a Waters Q–TOF Permier Spectrometer. X-ray crystallography analysis was performed on Bruker X8 APEX X-ray diffractionmeter. Optical rotations were measured using a 1 mL cell with a 1 dm path length on a Jasco P-1030 polarimeter and are reported as follows: [α]rtD (c is in g per 100 mL solvent).Analytical thin-layer chromatography (TLC) was carried out on Merck 60 F254 pre-coated silica gel plate (0.2 mm thickness).

2. General procedure
a) Optimal conditions

Supplementary Table 1. Optimized conditions for 4,4-disubstituted glutarimides [a]

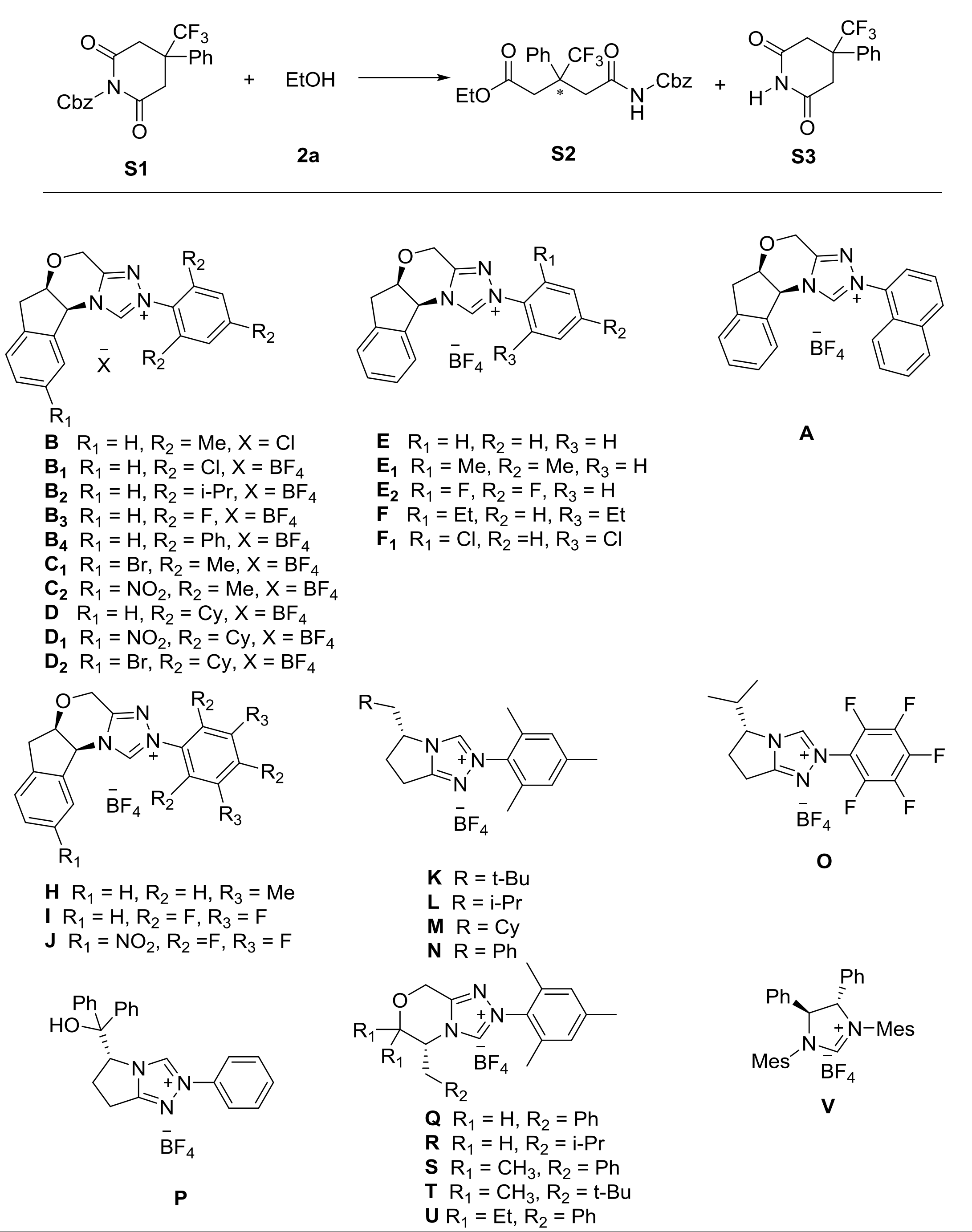

Entry[a]	NHC	Base	Solvent	Yield(%)[b] **S2**	Yield(%)[b] **S3**	ee(%)[c]
1	**B**	K_2CO_3	DCM	54	32	46
2	**B**	[b2]K_2CO_3	DCM	32	46	12
3	**B**	[b2]K_2CO_3	DCM	34	40	13
4	**B**	K_2CO_3	CCl_4	36	45	13
5	**B**	K_2CO_3	C_6H_5Cl	40	35	0
6	**B**	K_2CO_3	[d1]DCM	60	32	10

7	**B**	K_2CO_3	[d2]DCM	30	54	13
8[f1]	**B**	K_2CO_3	DCM	35	40	13
9[f2]	**B**	K_2CO_3	DCM	32	45	13
10	$\mathbf{B_1}$	K_2CO_3	DCM	nr	trace	-
11	$\mathbf{B_2}$	K_2CO_3	DCM	trace	trace	-
12	$\mathbf{B_3}$	K_2CO_3	DCM	trace	45	-
13	$\mathbf{B_4}$	K_2CO_3	DCM	trace	50	-
14	**C**	K_2CO_3	DCM	70	nr	62
15	**D**	K_2CO_3	DCM	nd	nd	-
16	**E**	K_2CO_3	DCM	23	50	3
17	**F**	K_2CO_3	DCM	49	42	46
18	**G**	K_2CO_3	DCM	35	42	10
19	$\mathbf{G_1}$	K_2CO_3	DCM	32	45	38
20	$\mathbf{G_2}$	K_2CO_3	DCM	28	43	26
21	**H**	K_2CO_3	DCM	trace	47	-
22	$\mathbf{H_1}$	K_2CO_3	DCM	trace	38	-
23	**I**	K_2CO_3	DCM	32	43	40
24	**J**	K_2CO_3	DCM	33	45	20
25	**K**	K_2CO_3	DCM	trace	42	-
26	**L**	K_2CO_3	DCM	nr	nr	-
27	$\mathbf{M_1}$	K_2CO_3	DCM	trace	70	-
28	$\mathbf{M_2}$	K_2CO_3	DCM	trace	72	-
29	**N**	K_2CO_3	DCM	trace	64	-
30	$\mathbf{N_1}$	K_2CO_3	DCM	nr	62	-
31	$\mathbf{N_2}$	K_2CO_3	DCM	22	56	10
32	**O**	K_2CO_3	DCM	trace	nr	-
33	**P**	K_2CO_3	DCM	nr	nr	-
34	**Q**	K_2CO_3	DCM	trace	80	-
35	**R**	K_2CO_3	DCM	32	62	19
36	**S**	K_2CO_3	DCM	trace	75	-
37	**T**	K_2CO_3	DCM	trace	nr	-
38	**U**	K_2CO_3	DCM	30	61	3
39	**V**	K_2CO_3	DCM	nr	nr	-
40	**F**	CH_3COONa	DCM	nr	nr	-
41	**F**	HCOONa	DCM	nr	nr	-
42	**F**	CH_3COOK	DCM	32	42	38
43	**F**	KOH	DCM	52	30	24
44	**F**	t-BuOK	DCM	64	trace	2
45	**F**	DABCO	DCM	41	55	51
46	**F**	NaH	DCM	trace	trace	-
47	**F**	DMAP	DCM	nr	72	-
48	**F**	DBN	DCM	nr	65	-
49	**F**	KH_2PO_4	DCM	nr	nr	-
50	**F**	DIPEA	DCM	nr	nr	-
51	**F**	C_2H_5ONa	DCM	nr	nr	-
52	**F**	K_2HPO_4	DCM	45	40	50
53	**F**	K_2HPO_4	1,2-Dimethoxyethane	nr	nr	-
54	**F**	K_2HPO_4	1,2-Dichloroethane	65	trace	50
55	**F**	K_2HPO_4	Acetonitrile	nr	nr	-
56	**F**	K_2HPO_4	Cyclopentyl Methyl Ether	nr	63	-
57	**F**	K_2HPO_4	1,4-Dioxane	nr	nr	-
58	**F**	K_2HPO_4	DME	nr	nr	-
59	**F**	K_2HPO_4	Acetone	nr	nr	-
60	**F**	K_2HPO_4	2-Methyltetrahydrofuran	nr	nr	-
61	**F**	K_2HPO_4	EA	nr	nr	-

62	**F**	K_2HPO_4	Benzotrifluoride	42	32	28
63	**F**	K_2HPO_4	dibutyl ether	trace	66	-
64	**F**	K_2HPO_4	DMSO	nr	nr	-
65	**F**	K_2HPO_4	1,4-dimethyl-benzene	trace	61	-
66	**F**	K_2HPO_4	Ethyl ether	trace	64	-
67	**F**	K_2HPO_4	m-Xylene	31	42	23
68	**F**	K_2HPO_4	Mesitylene	trace	55	-
69	**F**	K_2HPO_4	$CHCl_3$	42	50	44
70	**F**	K_2HPO_4	DCM/Hexane	39	52	40
71	**F**	K_2HPO_4	THF	nr	nr	-
72	**F**	DABCO	1,2-Dichloroethane	35	52	50
73	**F**	DABCO	$CHCl_3$	42	41	45
74	**C**	K_2HPO_4	1,2-Dichloroethane	36	41	57
75	**C**	DABCO	DCM	29	31	54
76	**C**	K_2HPO_4	DCM	71	nr	66
77	**C**	DABCO	$CHCl_3$	31	33	54
78[d]	**C**	K_2CO_3	DCE	55	-	54
79[d]	**C**	K_2CO_3	DCM	70	-	62
80[d]	**C**	K_2CO_3	1,1,2,2-tetrachloroethane	nr	-	nr
81[e]	**C**	K_2CO_3	DCM	70	-	64
82	**C**	Et_3N	DCM	nd	-	nr
83	**C**	DBU	DCM	53	-	3
84	**C**	K_3PO_4	DCM	57	-	52
85	**C**	Cs_2CO_3	DCM	nr	-	nr
86	**C**	Na_2CO_3	DCM	42	-	33
87	**C**	$NaHCO_3$	DCM	nr	-	nr
88	**C**	$(NH_4)_2CO_3$	DCM	nr	-	nr
89	**C**	$KHCO_3$	DCM	48	-	30

[a] Standard condition: **S1** (0.1 mmol), **2a** (1.5 equiv), NHC precursor (20 mol %), base (1.5 equiv), solvent (0.1 M), 30 ℃, N_2, 12-72h. [b] Yield (after SiO_2 chromatography purification) were based on **S1**. [c] Determined via chiral phase HPLC analysis. [d] -20 ℃. [e] -30 ℃. nr = no reaction. nd = not detected. [b1] K_2CO_3 (1.0 equiv), [b2] K_2CO_3 (2.0 equiv); [d1]DCM (0.2 M), [d2]DCM (0.05 M). [f1] 2a (1.2 equiv), [f2] 2a (2.0 equiv).

Supplementary Table 2. Optimized conditions for 4-methyl glutarimides [a]

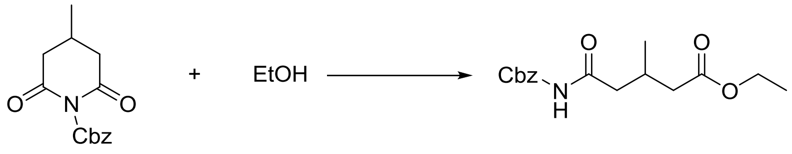

1n **2a** **T2**

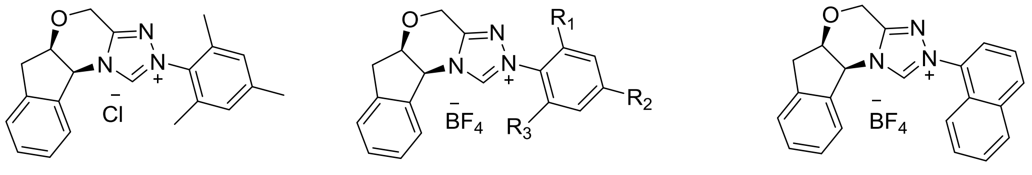

B

B2 R1 = i-Pr, R2= i-Pr, R3= i-Pr
B3 R1 = F, R2= F, R3= F
B4 R1 = Ph, R2= Ph, R3= Ph
G R1 = H, R2= H, R3= H
G1 R1 = Me, R2= Me, R3= H
G2 R1 = F, R2= F, R3= H
H R1 = Et, R2= H, R3=Et
H1 R1 = Cl, R2= H, R3=Cl

I

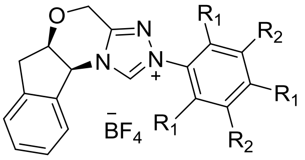

J R1 = H, R2 = Me
K R1 =F, R2 = F

Entry[a]	NHC	Base	Solvent	Yield(%)[b] **T2**	ee(%)[c]
1	**B**	K_2CO_3	DCM	85	71
2	**B**	[b1]K_2CO_3	DCM	79	70
3	**B**	[b2]K_2CO_3	DCM	80	70
4	**B**	K_2CO_3	CCl_4	80	43
5	**B**	K_2CO_3	C_6H_5F	82	72
6	**B**	K_2CO_3	C_6H_5Cl	84	72
7	**B**	K_2CO_3	C_6H_5Br	83	72
8	**B**	K_2CO_3	tert-Butanol	80	69
9	**B**	K_2CO_3	[d1]DCM	83	69
10	**B**	K_2CO_3	[d2]DCM	80	70
11[h1]	**B**	K_2CO_3	DCM	81	71
12[h2]	**B**	K_2CO_3	DCM	83	70
13	**B$_2$**	K_2CO_3	DCM	trace	-
14	**B$_3$**	K_2CO_3	DCM	trace	-
15	**B$_4$**	K_2CO_3	DCM	trace	-
16	**G**	K_2CO_3	DCM	80	10
17	**G$_1$**	K_2CO_3	DCM	84	73
18	**G$_2$**	K_2CO_3	DCM	75	60
19	**H**	K_2CO_3	DCM	trace	-
20	**H$_1$**	K_2CO_3	DCM	trace	-

21	**I**	K_2CO_3	DCM	78	71
22	**J**	K_2CO_3	DCM	84	52
23	**K**	K_2CO_3	DCM	trace	-
24	**G_1**	K_2CO_3	C_6H_5Cl	82	76
25[d]	**G_1**	K_2CO_3	C_6H_5Cl	84	78
26[e]	**G_1**	K_2CO_3	C_6H_5Cl	82	77
27[f]	**G_1**	K_2CO_3	C_6H_5Cl	82	60
28[f]	**G_1**	K_2CO_3	DCM	81	60
29[g]	**B**	K_2CO_3	DCM	84	82
30[d]	**B**	K_2CO_3	C_6H_5F	82	77
31[d]	**B**	K_2CO_3	C_6H_5Br	83	76

[a] Standard condition: 1**n** (0.1 mmol), **2a** (1.5 equiv), NHC precursor (20 mol %), base (1.5 equiv), solvent (0.1 M), 30 ℃, N_2, 12-72h. [b] Yield (after SiO_2 chromatography purification) were based on **S1**. [c] Determined via chiral phase HPLC analysis. [d] 0 ℃. [e] -10 ℃. [f] -20 ℃. [g] -40 ℃. [h1] **2a** (1.2 equiv), [h2] **2a** (2.0 equiv). [b1] K_2CO_3 (1.0 equiv), [b2] K_2CO_3 (2.0 equiv); [d1]DCM (0.2 M), [d2]DCM (0.05 M).

b) Additional reactions

Supplementary Table 3. Other 4,4-disubstituted glutarimides investigation [a]

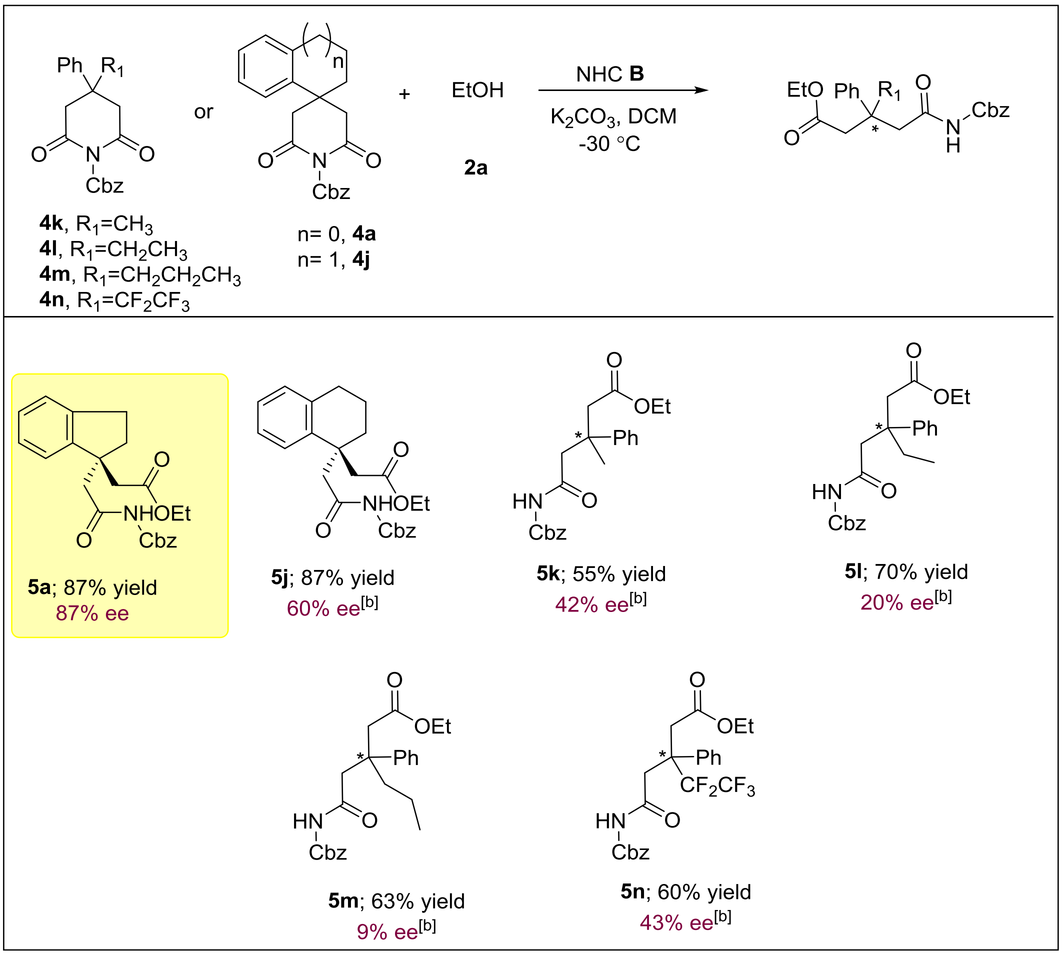

[a] Standard condition: **4** (0.1 mmol), **2a** (1.5 equiv), NHC precursor **B** (20 mol %), K_2CO_3 (1.5 equiv), DCM (0.1 M), -30 ℃, N_2, 72h. [b] NHC precursor K was used.

Supplementary Table 4. Other 4-methyl glutarimides investigation [a]

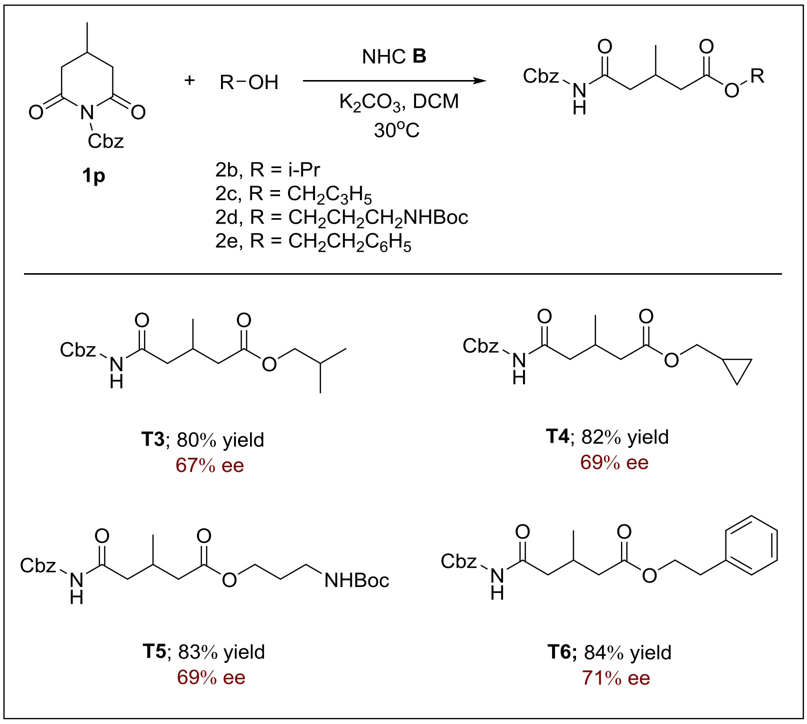

[a] Standard condition: **1p** (0.1 mmol), **2** (1.5 equiv), NHC precursor **B** (20 mol %), K_2CO_3 (1.5 equiv), DCM (0.1 M), 30 ℃, N_2, 72h.

Supplementary Table 5. Other nucleophiles investigation[a]

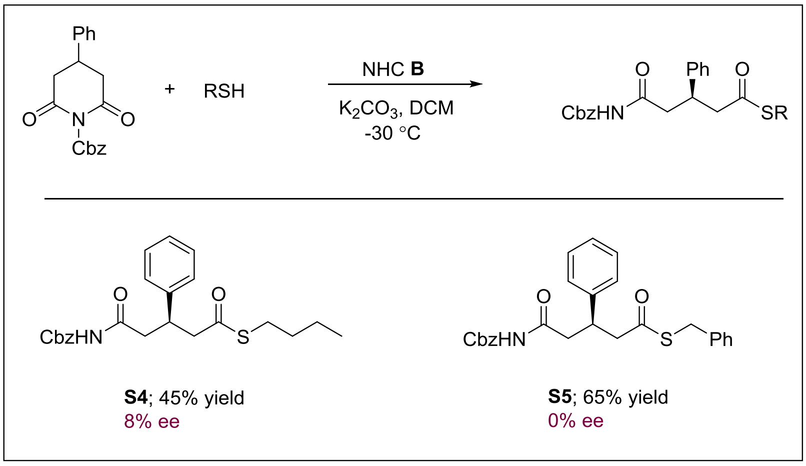

Supplementary Table 6. Scope of reaction by using ent-NHC **B** [a]

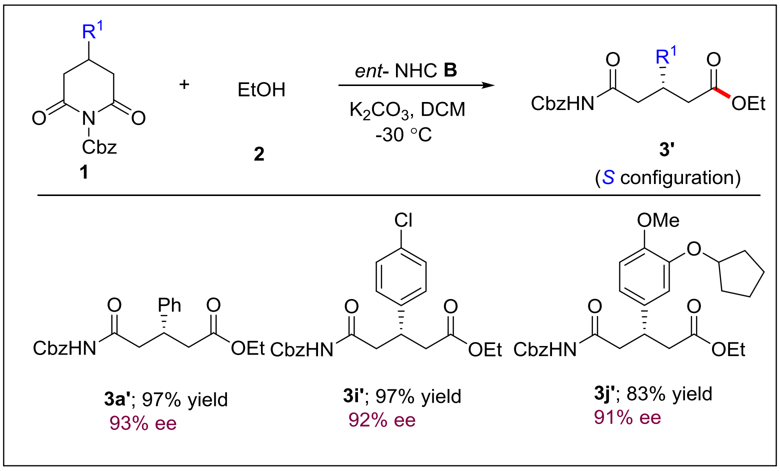

To our delight, the catalyst ent-NHC **B**, with the oppositive absolute configuration of NHC **B**, promoted this transformation smoothly to generate **3a'**, **3i'** and **3m'** with *S*-configuration in excellent yields and enantioselectivities. Importantly, the resulting products are available in the both *R* and *S*-configuration to further increase the utility of this reaction.

c) General procedure for the synthesis of compounds 1 (1c as an example) [1]

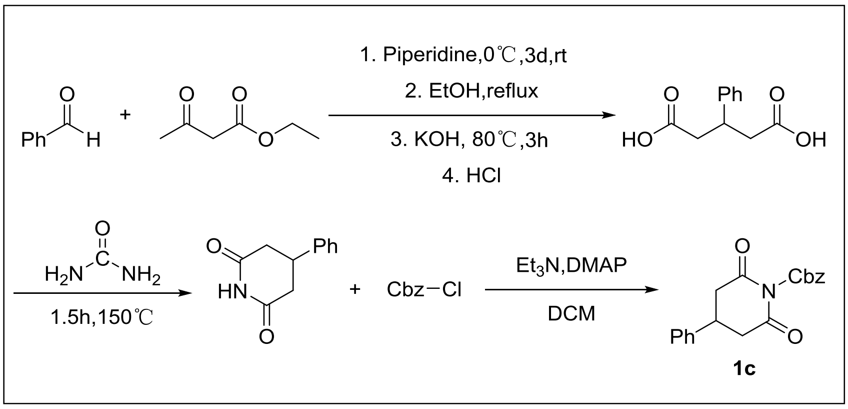

To a dry flask was added aromatic aldehydes (10 mmol) and ethyl acetoacetate (20 mmol), and piperidine (0.2 mL) was added dropwise at 0 ℃. The mixture was left at room temperature for 3 days. The resulting solid was dissolved in ethanol and refluxed. After cooling to room temperature, the crystalline product was filtered off and the precipitate was added to an aqueous solution of KOH (20 M), which was stirred at 80 ℃. After 2 h, ethyl acetate was added and the aqueous phase was separated. Then to aqueous phase was added concentrated aqueous HCl to adjust the pH to 1. The reaction mixture was extracted with ethyl acetate, and the combined organic layers were dried over anhydrous Na_2SO_4, filtered and concentrated under reduce pressure.3-Arylglutaric acid was given as a white solid without any purification.

3-Arylglutaric acid (7 mmol) and urea (17.5 mmol) were stirred 2 h at 150 ℃ without the addition of a solvent. Upon cooling, the crude product mixture was poured to the water and extracted with CH_2Cl_2, the combined organic layers were dried over anhydrous Na_2SO_4, filtered and concentrated under reduce pressure. The product 3-arylglutarimide was obtained as a white solid.

To a stirred mixture of product 3-arylglutarimide (5 mmol) and DMAP (0.5 mmol) in DCM (15 mL) was added Et_3N(10 mmol) followed by Cbz-Cl (15 mmol) and the mixture was stirred at rt overnight. The mixture was concentrated under reduced pressure, and crude residue was purified by flash chromatoghraphy (PE: EtOAc =3:1) to afford **1c** with 48% yield as a white solid.

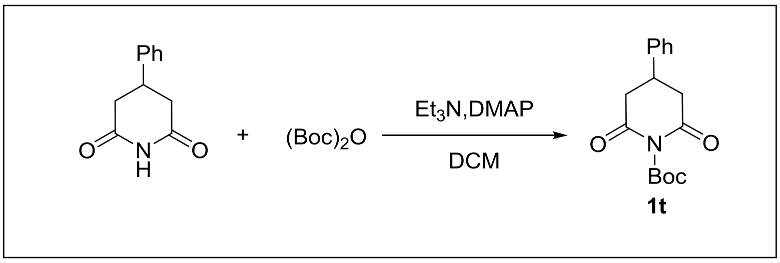

To a stirred mixture of product 3-arylglutarimide (5 mmol) and DMAP (0.5 mmol) in DCM (15 mL) was added $(Boc)_2O$ (15 mmol) and the mixture was stirred at rt 5h. The mixture was concentrated under reduced pressure, and crude residue was purified by flash chromatoghraphy (PE: EtOAc =3:1) to afford **1t** with 78% yield as a white solid.

d) General procedure for the catalytic reactions of glutarimides 1 and alcohols 2 to synthesize products 3 (3a as an example)

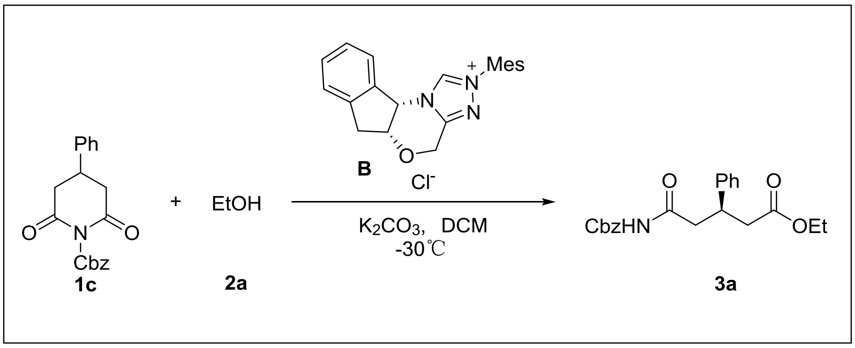

To a flame-dried screw-capped test tube equipped with a magnetic stir bar compound **1c** (0.1 mmol, 32.3 mg), alcohol **2a** (0.15 mmol, 9 μL), K_2CO_3 (0.15 mmol, 20.7 mg), NHC **B** (0.02 mmol, 7.3 mg) and dryed DCM (1 mL) was added. The reaction mixture was kept stirring at -30 ℃ for 3 d. When the reaction is complete, the crude residue was purified by flash column chromatography on silica gel using（PE：EtOAc =5:1) as eluent to afford the product **3a** as a colorless liquild in 85% yield and 95% *ee*.

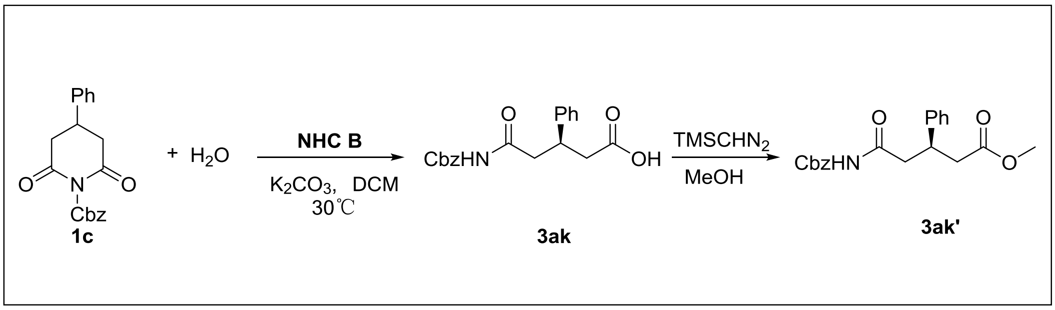

To a flame-dried screw-capped test tube equipped with a magnetic stir bar compound **1c** (0.1 mmol, 32.3 mg), H_2O (1.5 mmol, 15 equiv), K_2CO_3 (0.15 mmol, 20.7 mg), NHC **B** (0.02 mmol,7.3 mg) and dried DCM (1 mL) was added. The reaction mixture was kept stirring at 30 ℃ for 3 d. When the reaction is complete, the crude residue was purified by flash column chromatography on silica gel using （PE：EtOAc = 5:1) as eluent to afford the product **3ak** as a colorless liquild in 68% yield，$TMSCHN_2$(120 μL) and MeOH (10 μL) were added to the **3ak** in 1 mL DCM,which was stirred for 0.5h. Then, the reaction mixture was directly purified through preparative thin layer chromatography on silica gel to afford **3ak'**. 79% ee as determined by HPLC (Chiralcel ASH, 90:10 hexanes/i-PrOH, 1.0 mL/min), t_r (major) = 44.66 min, t_r (minor) = 69.63min. （Note: the ee value of **3ak** was indirectly determined from **3ak'** which was formed through acid methylation of **3ak'**.）

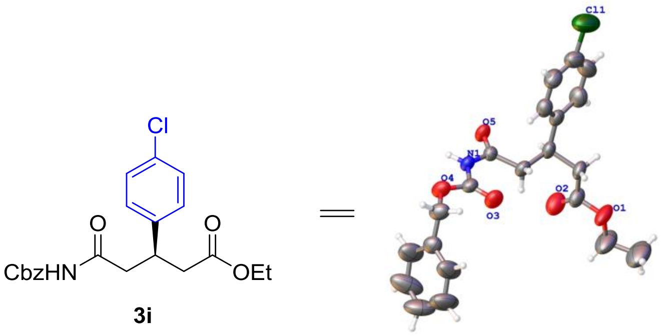

Supplementary Fig. 1. X-ray crystal structure of **3i.** Product **3i** was crystallized as a colorless crystal *via* vaporization of a hexane/ethyl acetate solution, and its absolute configuration was determined by X-ray structure analaysis. CCDC 2115884 contains the supplementary crystallographic data that can be obtained free of charge from The Cambridge Crystallographic Data Centrevia www.ccdc.cam.ac.uk/data request/cif.

Supplementary Table 7. Crystal data and structure refinement for **3i**

Identification code	ZLF-HXF
Empirical formula	$C_{21}H_{22}ClNO_5$
Formula weight	403.84
Temperature	293(2)
Crystal system	triclinic

Space group	P1
Unit cell dimensions	
a/Å	4.9416(5)
b/Å	12.9215(12)
c/Å	17.3385(16)
α/°	108.628(8)
β/°	91.469(8)
γ/°	93.732(8)
Volume	1045.63(18)
Z	2
Density(calculated)	1.283 g/cm^3
μ	0.213 mm^{-1}
F(000)	424.0
Crystal size	0.14 × 0.12 × 0.11 mm^3
Radiation	MoKα (λ = 0.71073)
2Θ range for data collection	4.756 to 60.764
Index ranges	-6 ≤ h ≤ 7, -18 ≤ k ≤ 18, -24 ≤ l ≤ 24
Reflections collected	14406
Independent reflections	9651 [R_{int} = 0.0510, R_{sigma} = 0.0854]
Data/restraints/parameters	9651/60/568
Goodness-of-fit on F^2	0.907
Final R indexes [I>=2σ (I)]	R_1 = 0.0657, wR_2 = 0.1459
Final R indexes [all data]	R_1 = 0.1436, wR_2 = 0.1803
Largest diff. peak/hole	0.19/-0.27 Å^{-3}
Flack parameter	0.07(6)/0.10(5)

e) Mechanistic studies

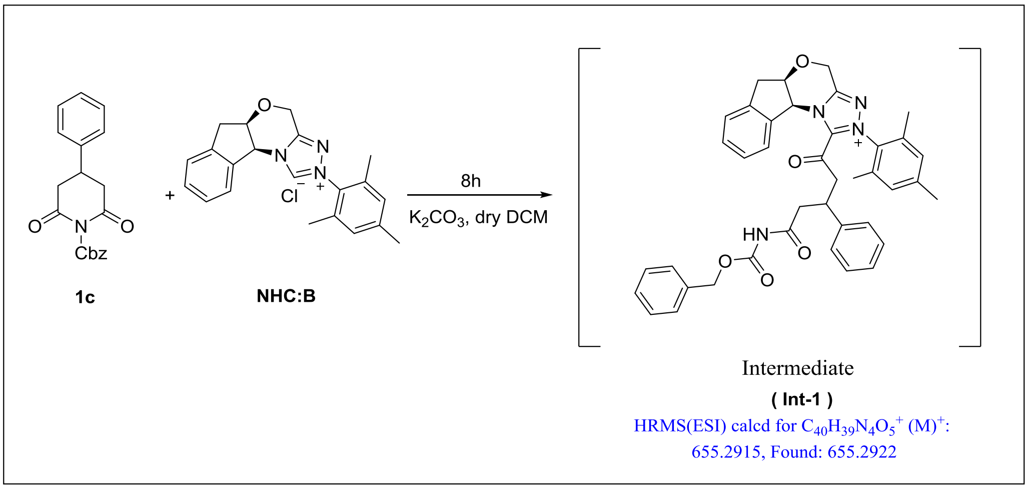

To a flame-dried screw-capped test tube equipped with a magnetic stir bar, compound **1c** (0.1 mmol, 32.3 mg), K_2CO_3 (0.15 mmol, 20.7 mg), NHC **B** (0.1 mmol, 36.5 mg) and dried DCM (1 mL) was added. The reaction mixture was kept stirring at 30 ℃ for 8 h. Immediately, the high resolution mass spectroscopy (HRMS-ESI) analysis of the reaction mixture was carried out. And one signal peak was detected at m/z 655.2922, which corresponded to the intermediate **Int-1**. **HRMS(ESI)** calcd for $C_{40}H_{39}N_4NaO_5^+$ $(M)^+$: 655.2915, Found: 655.2922;.

Monoisotopic Mass, Even Electron Ions
1 formula(e) evaluated with 1 results within limits (up to 50 closest results for each mass)
Elements Used:
C: 39-41 H: 38-40 N: 3-5 O: 3-6
hxf-0001 235 (1.763)
1: TOF MS ES+

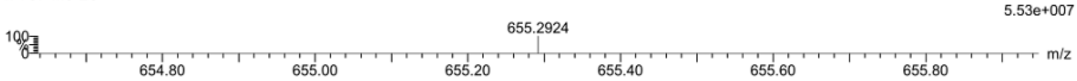

	Calc. Mass	mDa	PPM	DBE	i-FIT	Norm	Conf(%)	Formula
Minimum:				-1.5				
Maximum:		5.0	10.0	50.0				

Mass	Calc. Mass	mDa	PPM	DBE	i-FIT	Norm	Conf(%)	Formula
655.2924	655.2920	0.4	0.6	23.5	52.2	n/a	n/a	C40 H39 N4 O5

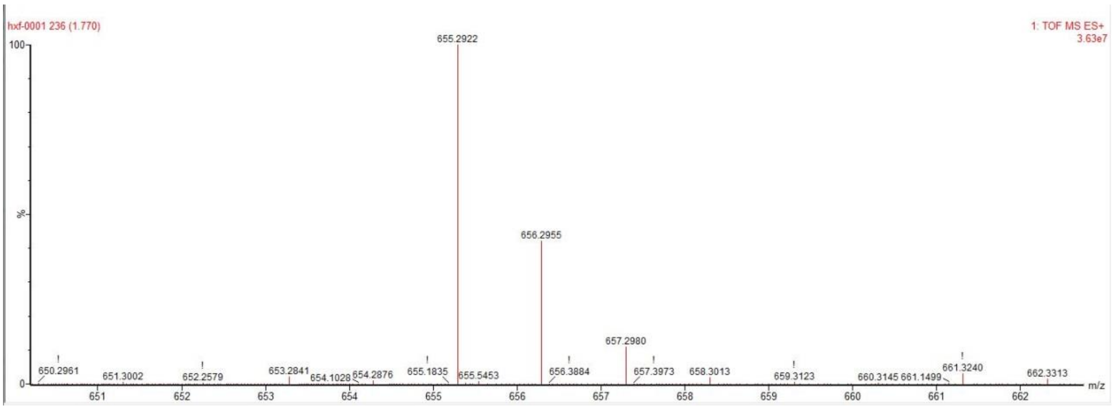

f) Synthetic transformations

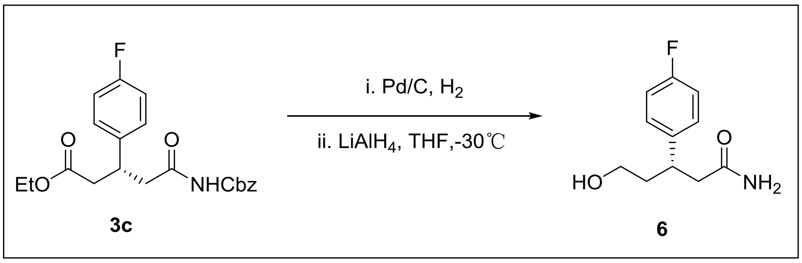

Product **3c** (0.2 mmol, 77.4 mg) and 5% Pd/C (0.02 mmol, 42.6 mg) were mixed in 2 mL of ethanol and stirred under an atmosphere of H_2 for 0.5 hour. The solids were filtered off, and the filtrate was concentrated under reduced pressure, which was used in the next step without purification. Then to the solid of THF (2 mL) was added $LiAlH_4$ (0.4 mmol, 15.2mg) at -30 ℃ and stirred for 2 days. After the completion of reaction, the reaction mixture was quenched with water, and extracted with EtOAc (3 × 20 mL), the combined organic layers were dried over anhydrous Na_2SO_4, filtered and concentrated under reduce pressure. And crude residue was purified by flash chromatoghraphy to afford **6** with 74% total yield and 91% *ee*.

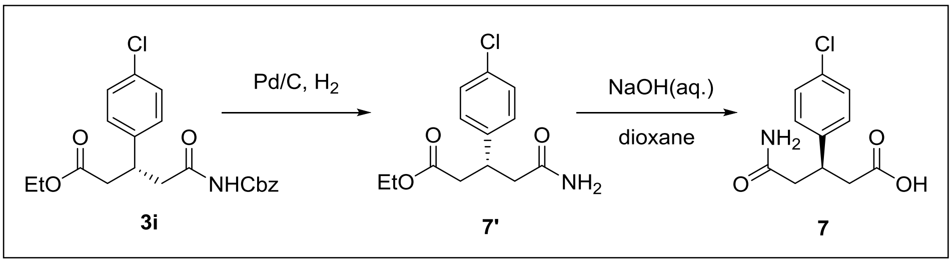

Product **3i** (0.2 mmol, 80.6 mg) and 5% Pd/C (0.02 mmol, 42.6 mg) were mixed in 2 mL of ethyl acetate and stirred at 0 ℃ under an atmosphere of H_2 for 0.5 hour. The solids were filtered off, and the filtrate was concentrated under reduced pressure and purified by flash column chromatography on silica gel using Petroleum ether/EtOAc (1:1) as eluent to afford the product **7'** as a white solid in 98% yield and 93% *ee.*

To the product **7'** (0.1 mmol, 26.9 mg) in dioxane (0.5 mL) at was added 6M NaOH(0.5 mL) and the reaction mixture stirred at 60 ℃ for 2 h. Then to the mixture was added concentrated aqueous HCl to adjust the pH to 1 and extracted with ethyl acetate, and the combined organic layers were dried over anhydrous Na_2SO_4, filtered and concentrated under reduce pressure and the crude residue was purified by flash chromatoghraphy to afford product **7** with 98% yield.

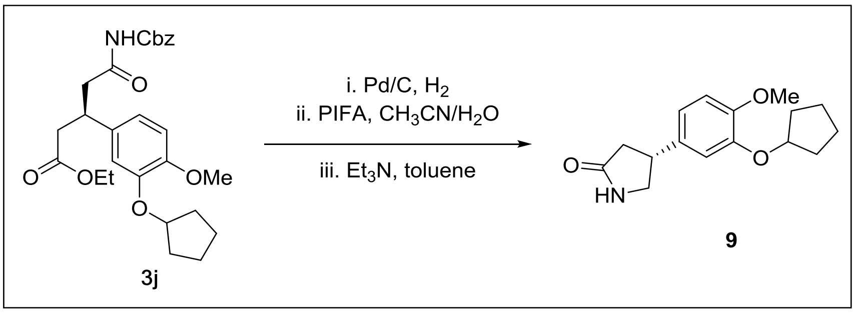

Product **3j** (0.2 mmol, 96.7 mg) and 5% Pd/C (0.02 mmol, 42.6 mg) were mixed in 2 mL of ethanol and stirred under an atmosphere of H_2 for 0.5 hour. The solids were filtered off, and the filtrate was concentrated under reduced pressure, which was used in the next step without purification. Then to the solid of CH_3CN/H_2O (1:1, 2 mL) was added PIFA (0.24 mmol, 103.2 mg) and stirred at room temperature for 24h. After the completion of reaction, the reaction mixture was quenched by 2M HCl and stirred another 1h, the reaction mixture extracted with EA (3 × 10 mL). The organic layer was dried over anhydrous sodium sulfate, and concentrated under reduced pressure. The last step is to the product in the toluene (1 mL) was added Et_3N (0.2 mmol, 27.8 μL) and the reaction mixture was stired at 110 ℃ for 24h. After the completion of reaction, the solvent was concentrated under reduced pressure, and the crude residue was purified by flash chromatoghraphy to afford product **9**[2] with 50% total yield and 95% *ee*.

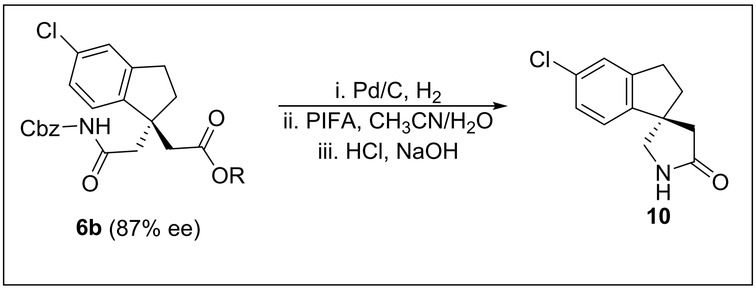

Product **6b** (0.2 mmol, 85.8 mg) and 5% Pd/C (0.02 mmol, 42.6 mg) were mixed in 2 mL of ethyl acetate and stirred under an atmosphere of H_2 at 0 ℃ for 4 hour. The solids were filtered off, and the filtrate was concentrated under reduced pressure, which was used in the next step without purification. Then to the solid of CH_3CN/H_2O (1:1, 2 mL) was added PIFA (0.24 mmol, 103.2 mg) and stirred at room temperature for 24h. After the completion of reaction, the reaction mixture was quenched by 2M HCl and stired another 1h, The mother liquor was spin-dried through a vacuum pump, and then recrystallized from ethyl acetate to obtain a pure hydrochloride compound. Adjust pH of the hydrochloride with excess sodium hydroxide aqueous solution, and then extract with ethyl acetate to obtain compound **10**.

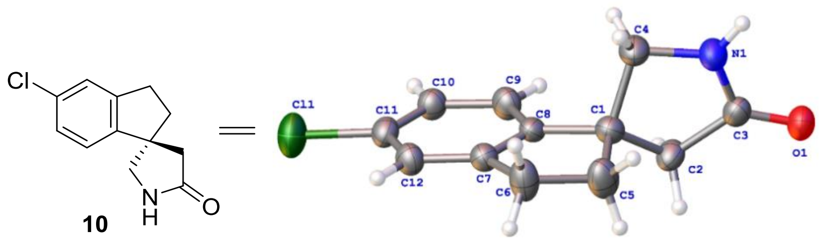

Supplementary Fig. 2. X-ray crystal structure of **10.** Product **10** was crystallized as a colorless crystal *via* vaporization of a hexane/ethyl acetate solution, and its absolute configuration was determined by X-ray structure analaysis. CCDC 2115883 contains the supplementary crystallographic data that can be obtained free of charge from The Cambridge Crystallographic Data Centrevia www.ccdc.cam.ac.uk/data request/cif.

Supplementary Table 8. Crystal data and structure refinement for **10**

Identification code	hxf
Empirical formula	$C_{12}H_{13}ClNO_{1.5}$
Formula weight	230.68
Temperature	293(2)
Crystal system	triclinic
Space group	P1
Unit cell dimensions	
a/Å	8.3572(8)
b/Å	9.7033(7)
c/Å	14.7449(11)
α/°	89.407(6)
β/°	77.647(8)
γ/°	87.371(7)
Volume	1166.79(17)
Z	4
Density(calculated)	1.313 g/cm^3
μ	0.306 mm^{-1}
F(000)	484.0
Crystal size	0.14 × 0.11 × 0.09 mm^3
Radiation	MoKα (λ = 0.71073)
2Θ range for data collection	4.202 to 60.49
Index ranges	$-11 \leq h \leq 11$, $-13 \leq k \leq 13$, $-20 \leq l \leq 20$
Reflections collected	16574
Independent reflections	10301 [R_{int} = 0.0576, R_{sigma} = 0.0808]
Data/restraints/parameters	10301/3/565

Goodness-of-fit on F^2	0.974
Final R indexes [I>=2σ (I)]	R_1 = 0.0612, wR_2 = 0.1519
Final R indexes [all data]	R_1 = 0.1275, wR_2 = 0.1870
Largest diff. peak/hole	0.23/-0.27 Å^{-3}
Flack parameter	-0.01(5)/0.01(4)

3. Characterizationof substrates and products, reference

a) Characterizations of substrates:

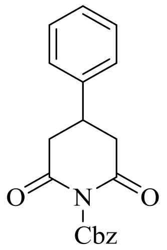

1c

Benzyl 2, 6-dioxo-4-phenylpiperidine-1-carboxylate (1c): white solid. **^{1}H NMR (400 MHz, $CDCl_3$)** *δ* 7.33-7.42 (m, 7H), 7.27-7.32 (m, 1H), 7.17-7.19 (m, 2H), 5.38 (s, 2H), 3.35-3.43 (m,1H), 2.92-2.98 (m, 2H), 2.73-2.81 (m, 2H). **^{13}C NMR (100 MHz, $CDCl_3$)** *δ* 169.4, 151.0, 140.0, 133.9, 129.4, 129.1, 128.8, 128.7, 128.0, 126.5, 71.3, 39.1, 35.0. **HRMS(ESI)** calcd for $C_{19}H_{17}NNaO_4^+$ $(M+Na)^+$: 346.1050, Found: 346.1058.

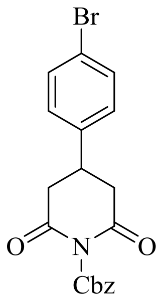

1d

Benzyl 4-(4-bromophenyl)-2,6-dioxopiperidine-1-carboxylate (1d): white solid. **^{1}H NMR (400 MHz, $CDCl_3$)** *δ* 7.48-7.52 (m, 2H), 7.34-7.43 (m, 5H), 7.06-7.09 (m, 2H), 5.40 (s, 2H), 3.36-3.45 (m, 1H), 2.94-2.99 (m, 2H), 2.73-2.80 (m, 2H). **^{13}C NMR (100 MHz, $CDCl_3$)** *δ* 168.9, 150.8, 138.9, 133.8, 132.5, 129.1, 128.8, 128.7, 128.2, 121.9, 71.4, 38.9, 34.5. **HRMS(ESI)** calcd for $C_{19}H_{16}BrNNaO_4^+$ $(M+Na)^+$: 424.0155, Found: 424.0150.

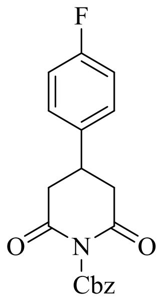

1e

Benzyl 4-(4-fluorophenyl)-2,6-dioxopiperidine-1-carboxylate (1e): white solid. **^{1}H NMR (400 MHz, $CDCl_3$)** *δ* 7.35-7.43 (m, 5H), 7.14-7.18 (m, 2H), 7.04-7.08 (m, 2H),

5.40 (s, 2H), 3.38-3.46 (m, 1H), 2.93-2.99 (m, 2H), 2.72-2.80 (m, 2H). **^{13}C NMR (100 MHz, $CDCl_3$)** *δ* 169.1, 163.5, 161.0, 150.9, 135.7, 133.8, 129.1, 128.8, 128.7, 128.1, 128.0, 116.4, 116.2, 71.3, 39.2, 34.3. **HRMS(ESI)** calcd for $C_{19}H_{16}FNNaO_4^+$ $(M+Na)^+$: 364.0956, Found: 364.0952.

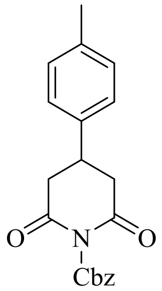

1f

Benzyl 2,6-dioxo-4-(p-tolyl)piperidine-1-carboxylate (1f): white solid. **^{1}H NMR (400 MHz, $CDCl_3$)** *δ* 7.34-7.43 (m, 5H), 7.17 (d, *J* = 8.0 Hz, 2H), 7.07 (d, *J* = 8.0 Hz, 2H), 5.39 (s, 2H), 3.34-3.42 (m, 1H), 2.92-2.98 (m, 2H), 2.72-2.80 (m, 2H), 2.34 (s, 3H). **^{13}C NMR (100 MHz, $CDCl_3$)** *δ* 169.5, 151.0, 137.8, 137.0, 133.9, 130.0, 129.0, 128.8, 128.7, 126.3, 71.3, 39.2, 34.6, 21.1. **HRMS(ESI)** calcd for $C_{20}H_{19}NNaO_4^+$ $(M+Na)^+$: 360.1206, Found: 360.1216.

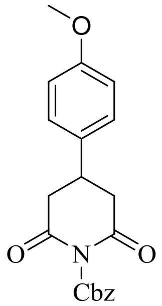

1g

Benzyl 4-(4-methoxyphenyl)-2,6-dioxopiperidine-1-carboxylate (1g): white solid. **^{1}H NMR (400 MHz, $CDCl_3$)** *δ* 7.34-7.43 (m, 5H), 7.10 (d, *J* = 8.8 Hz, 2H), 6.89 (d, *J* = 8.8 Hz, 2H), 5.39 (s, 2H), 3.79 (s, 3H), 3.33-3.41 (m, 1H), 2.92-2.97 (m, 2H), 2.75 (dd, *J* = 17.2, 12.0 Hz, 2H). **^{13}C NMR (100 MHz, $CDCl_3$)** *δ* 169.5, 159.2, 151.0, 133.9, 132.0, 129.0, 128.8, 128.7, 127.5, 114.7, 71.3, 55.4, 39.4, 34.2. **HRMS(ESI)** calcd for $C_{20}H_{19}NNaO_5^+$ $(M+Na)^+$: 376.1155, Found: 376.1153.

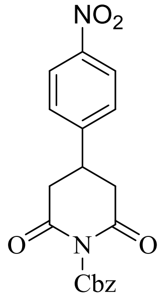

1h

Benzyl 4-(4-nitrophenyl)-2,6-dioxopiperidine-1-carboxylate (1h): yellow solid. **^{1}H NMR (400 MHz, CDCl$_3$)** δ 8.24-8.26 (m, 2H), 7.37-7.43 (m, 7H), 5.41 (s, 2H), 3.54-3.62 (m, 1H), 3.00-3.06 (m, 2H), 2.80-2.88 (m, 2H). **^{13}C NMR (100 MHz, CDCl$_3$)** δ 168.3, 150.5, 147.7, 146.9, 137.6, 133.7, 129.2, 128.8, 128.7, 127.6, 124.7, 71.5, 38.6, 34.9. **HRMS(ESI)** calcd for $C_{19}H_{16}N_2NaO_6^+$ (M+Na)$^+$: 391.0901, Found: 391.0911.

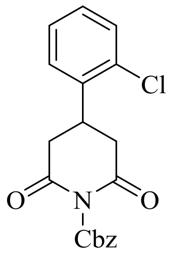

1i

Benzyl 4-(2-chlorophenyl)-2,6-dioxopiperidine-1-carboxylate(1i): yellow liquid. **^{1}H NMR (400 MHz, CDCl$_3$)** δ 7.27-7.45 (m, 8H), 7.18 (dd, *J* = 7.6, 2.0 Hz, 1H), 5.41 (s, 2H), 3.87-3.94 (m, 1H), 2.99-3.04 (m, 2H), 2.77-2.85 (m, 2H). **^{13}C NMR (100 MHz, CDCl$_3$)** δ 169.2, 150.9, 137.1, 133.8, 130.6, 129.2, 129.1, 128.8, 128.7, 127.8, 126.4, 71.4, 37.5, 31.6. **HRMS(ESI)** calcd for $C_{19}H_{16}ClNNaO_4^+$ (M+Na)$^+$: 380.0660, Found: 380.0663.

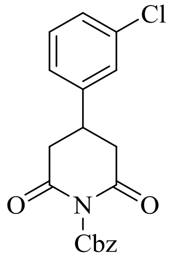

1j

Benzyl 4-(3-chlorophenyl)-2,6-dioxopiperidine-1-carboxylate (1j): white solid. **^{1}H NMR (400 MHz, CDCl$_3$)** δ 7.33-7.43 (m, 5H), 7.28-7.31 (m, 2H), 7.18-7.19 (m, 1H), 7.06-7.09 (m, 1H), 5.40 (s, 2H), 3.36-3.44 (m, 1H), 2.94-2.99 (m, 2H), 2.73-2.81 (m, 2H). **^{13}C NMR (100 MHz, CDCl$_3$)** δ 168.9, 150.8, 141.9, 135.2, 133.8, 130.7, 129.1, 128.8, 128.7, 128.3, 126.8, 124.7, 71.4, 38.9, 34.7. **HRMS(ESI)** calcd for $C_{19}H_{16}ClNNaO_4^+$ (M+Na)$^+$: 380.0660, Found: 380.0668.

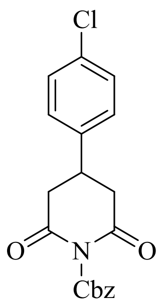

1k

Benzyl 4-(4-chlorophenyl)-2,6-dioxopiperidine-1-carboxylate (1k): white solid. **^{1}H NMR (400 MHz, CDCl$_3$)** δ 7.33-7.42 (m, 7H), 7.13 (d, J = 8.4 Hz, 2H), 5.40 (s, 2H), 3.38-3.46 (m, 1H), 2.94-3.00 (m, 2H), 2.77 (dd, J = 17.2, 12.0 Hz, 2H). **^{13}C NMR (100 MHz, CDCl$_3$)** δ 169.0, 150.8, 138.4, 133.9, 133.8, 129.6, 129.1, 128.8, 128.7, 127.8, 71.4, 39.0, 34.4. **HRMS(ESI)** calcd for $C_{19}H_{16}ClNNaO_4^+$ (M+Na)$^+$: 380.0660, Found: 380.0663.

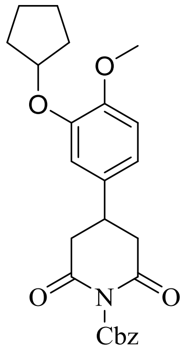

1l

Benzyl 4-(3-(cyclopentyloxy)-4-methoxyphenyl)-2,6-dioxopiperidine-1-carboxylate (1l): Colorless liquid. **^{1}H NMR (400 MHz, CDCl$_3$)** δ 7.33-7.41 (m, 5H), 6.82 (d, J = 8.0 Hz, 1H), 6.66-6.69 (m, 2H), 5.37 (s, 2H), 4.70-4.74 (m, 1H), 3.80 (s, 3H), 3.29-3.37 (m, 1H), 2.91-2.97 (m, 2H), 2.71-2.78 (m, 2H), 1.78-1.90 (m, 6H), 1.56-1.62 (m, 2H). **^{13}C NMR (100 MHz, CDCl$_3$)** δ 169.5, 151.0, 149.7, 148.2, 133.9, 132.4, 129.0, 128.8, 128.7, 118.3, 113.3, 112.4, 80.7, 71.3, 56.2, 39.3, 34.4, 32.9, 24.1. **HRMS(ESI)** calcd for $C_{25}H_{27}NNaO_6^+$ (M+Na)$^+$: 460.1731, Found: 460.1737.

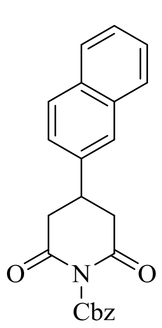

1m

Benzyl 4-(naphthalen-2-yl)-2,6-dioxopiperidine-1-carboxylate (1m): white solid. **^{1}H NMR (400 MHz, CDCl$_3$)** δ 7.77-7.86 (m, 3H), 7.59 (d, J = 2.0 Hz, 1H), 7.48-7.51 (m, 2H), 7.36-7.44 (m, 5H), 7.27 (dd, J = 8.4, 2.0 Hz, 1H), 5.40 (s, 2H), 3.51-3.59 (m, 1H), 3.01-3.06 (m, 2H), 2.82-2.89 (m, 2H). **^{13}C NMR (100 MHz, CDCl$_3$)** δ 169.4, 151.0, 137.3, 133.9, 133.5, 132.8, 129.3, 129.1, 128.8, 128.7, 127.9, 127.8, 126.9, 126.5, 125.1, 124.5, 71.3, 39.0, 35.0. **HRMS(ESI)** calcd for $C_{23}H_{19}NNaO_4^+$ (M+Na)$^+$: 396.1206, Found: 396.1215;

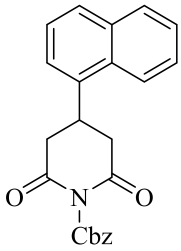

1n

Benzyl 4-(naphthalen-1-yl)-2,6-dioxopiperidine-1-carboxylate (1n): yellow liquid. **^{1}H NMR (400 MHz, CDCl$_3$)** δ 7.97 (d, J = 8.4 Hz, 1H), 7.90 (dd, J = 8.0, 1.6 Hz, 1H), 7.82 (d, J = 8.0 Hz, 1H), 7.52-7.60 (m, 2H), 7.35-7.49 (m, 6H), 7.30 (d, J = 7.2 Hz, 1H), 5.43 (s, 2H), 4.21-4.29 (m, 1H), 3.14 (dd, J = 17.2, 4.0 Hz, 2H), 2.93 (dd, J = 17.2, 11.6 Hz, 2H). **^{13}C NMR (100 MHz, CDCl$_3$)** δ 169.6, 151.0, 135.6, 134.1, 133.8, 130.7, 129.5, 129.1, 128.9, 128.8, 128.7, 127.1, 126.3, 122.2, 122.1, 71.4, 38.8, 30.2. **HRMS(ESI)** calcd for $C_{23}H_{19}NNaO_4^+$ (M+Na)$^+$: 396.1206, Found: 396.1212.

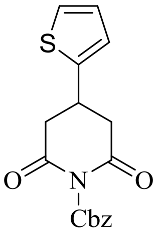

1o

Benzyl 2,6-dioxo-4-(thiophen-2-yl)piperidine-1-carboxylate (1o): Colorless liquid. **^{1}H NMR (400 MHz, CDCl$_3$)** δ 7.33-7.42 (m, 5H), 7.23 (dd, *J* = 5.2, 1.2 Hz, 1H), 6.95-6.97 (m, 1H), 6.87-6.88 (m, 1H), 5.37 (s, 2H), 3.65-3.73 (m, 1H), 3.04-3.10 (m, 2H), 2.78-2.86 (m, 2H). **^{13}C NMR (100 MHz, CDCl$_3$)** δ 168.7, 150.8, 143.6, 133.9, 129.0, 128.8, 128.7, 127.4, 124.8, 124.1, 71.3, 39.8, 30.6. **HRMS(ESI)** calcd for $C_{17}H_{15}NNaO_4S^+$ (M+Na)$^+$: 352.0614, Found: 352.0622.

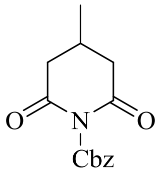

1p

Benzyl 4-methyl-2,6-dioxopiperidine-1-carboxylate (1p): white solid. **^{1}H NMR (400 MHz, $CDCl_3$)** δ 7.35-7.43 (m, 5H), 5.38 (s, 2H), 2.73-2.78 (m, 2H), 2.29-2.37 (m, 3H), 1.11-1.13 (m, 3H). **^{13}C NMR (100 MHz, $CDCl_3$)** δ 169.8, 151.1, 133.9, 129.0, 128.8, 128.7, 71.2, 39.8, 24.7, 20.3. **HRMS(ESI)** calcd for $C_{14}H_{15}NNaO_4^+$ $(M+Na)^+$: 284.0893, Found: 284.0890.

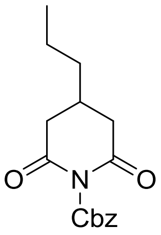

1q

Benzyl 2,6-dioxo-4-propylpiperidine-1-carboxylate (1q): colorless oily. **^{1}H NMR (400 MHz, $CDCl_3$)** δ 7.30-7.45 (m, 5H), 5.35 (s, 2H), 2.71-2.76 (m,2H), 2.25-2.32 (m, 2H), 2.10-2.14 (m, 1H), 1.33-1.34 (m, 4H), 0.89-0.94 (m, 3H). **^{13}C NMR (100 MHz, $CDCl_3$)** δ 170.1, 151.2, 134.0, 129.0, 128.8, 128.6, 71.1, 38.0, 36.8, 29.2, 19.6, 14.0. **HRMS(ESI)** calcd for $C_{16}H_{19}NNaO_4^+$ $(M+Na)^+$: 312.1206, Found: 312.1200.

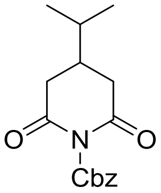

1r

Benzyl 4-isopropyl-2,6-dioxopiperidine-1-carboxylate (1r): colorless oily. **^{1}H NMR (400 MHz, $CDCl_3$)** δ 7.35-7.42 (m, 5H), 5.37 (s, 2H), 2.73 (dd, J = 4.0, 17.2 Hz, 2H), 2.30-2.38 (m, 2H), 1.90-2.00 (m, 1H), 1.58-1.63 (m, 1H), 0.95 (s, 3H), 0.93 (s, 3H). **^{13}C NMR (100 MHz, $CDCl_3$)** δ 170.3, 151.1, 133.9, 129.0, 128.8, 128.7, 71.2, 35.9, 35.7, 31.3, 19.3. **HRMS(ESI)** calcd for $C_{16}H_{19}NNaO_4^+$ $(M+Na)^+$: 312.1206, Found: 312.1200.

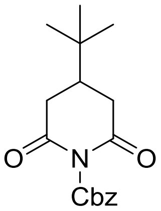

1s

Benzyl 4-(tert-butyl)-2,6-dioxopiperidine-1-carboxylate (1s): white solid. **^{1}H NMR (400 MHz, $CDCl_3$)** δ 7.35-7.40 (m, 5H), 5.35 (s, 2H), 2.75 (d, J = 17.2 Hz, 2H), 2.31 (t, J = 12.8 Hz, 2H), 1.87-1.94 (m, 1H) , 0.90 (s, 9H). **^{13}C NMR (100 MHz, $CDCl_3$)** δ 170.7, 151.1, 133.9, 129.0, 128.8, 128.6, 71.1, 39.4, 34.1, 32.0, 26.7. **HRMS(ESI)** calcd for $C_{17}H_{21}NNaO_4^+$ $(M+Na)^+$: 346.1050, Found: 346.1058.

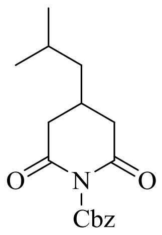

1t

Benzyl 4-isobutyl-2,6-dioxopiperidine-1-carboxylate (1t): colorless oily. **^{1}H NMR (400 MHz, CDCl$_3$)** δ 7.35-7.43 (m, 5H), 5.38 (s, 2H), 2.73-2.79 (m, 2H), 2.24-2.31 (m, 3H), 1.61-1.68 (m,1H), 1.23-1.26 (m, 2H), 0.90 (d, *J* = 6.4Hz, 6H). **^{13}C NMR (100 MHz, CDCl$_3$)** δ 169.9, 151.1, 133.9, 129.0, 128.8, 128.7, 71.2, 44.1, 38.3, 27.3, 24.8, 22.5. **HRMS(ESI)** calcd for $C_{17}H_{21}NNaO_4^+$ $(M+Na)^+$: 326.1363, Found: 326.1365.

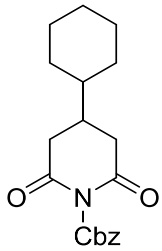

1u

Benzyl 4-cyclohexyl-2,6-dioxopiperidine-1-carboxylate (1u): white solid. **^{1}H NMR (400 MHz, CDCl$_3$)** δ 7.26-7.42 (m, 5H), 5.37 (s, 2H), 2.78 (dd, *J* = 4.0,17.2 Hz, 2H), 2.32-2.39 (m, 2H), 1.92-2.05 (m,1H), 1.66-1.79 (m, 5H), 1.11-1.28 (m, 4H), 0.91-1.00 (m, 2H). **^{13}C NMR (100 MHz, CDCl$_3$)** δ 170.5, 151.1, 133.9, 129.0, 128.8, 128.6, 71.1, 41.0, 35.9, 34.8, 29.7, 26.6, 26.3. **HRMS(ESI)** calcd for $C_{19}H_{23}NNaO_4^+$ $(M+Na)^+$: 352.1519, Found: 352.1508.

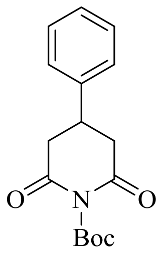

1v

***Tert*-butyl 2,6-dioxo-4-phenylpiperidine-1-carboxylate (1v)**: white solid.**^{1}H NMR (400 MHz, CDCl$_3$)** δ 7.36-7.40 (m, 2H), 7.28-7.33 (m, 1H), 7.20-7.22 (m, 2H), 3.40-3.48 (m, 1H), 2.94-3.00 (m, 2H), 2.76-2.83 (m, 2H), 1.58 (s, 9H). **^{13}C NMR (100 MHz, CDCl$_3$)** δ 169.5, 148.8, 140.2, 129.3, 127.9, 126.5, 86.6, 39.2, 35.1, 27.6. **HRMS(ESI)** calcd for $C_{16}H_{19}NNaO_4^+$ $(M+Na)^+$: 312.1206, Found: 312.1200.

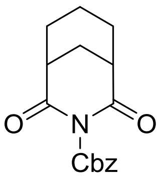

1w

Benzyl 2,4-dioxo-3-azabicyclo[3.3.1]nonane-3-carboxylate (1w): colorless oily. **^{1}H NMR (400 MHz, CDCl$_3$)** δ 7.33-7.44 (m, 5H), 5.40 (s, 2H), 2.89 (s, 2H), 2.24-2.29 (m, 1H), 2.04-2.10 (m, 2H), 1.73-1.80 (m, 3H), 1.55-1.69 (m, 2H). **^{13}C NMR (100 MHz, CDCl$_3$)** δ 173.4, 151.4, 134.1, 128.9, 128.7, 128.6, 71.0, 37.7, 28.2, 28.0, 19.2. **HRMS (ESI)** calcd for $C_{16}H_{18}NO_4^+$ $(M+H)^+$: 288.1230, Found: 288.1233.

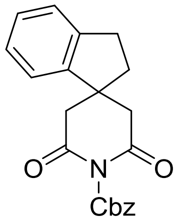

4a

Benzyl 2',6'-dioxo-2,3-dihydrospiro[indene-1,4'-piperidine]-1'-carboxylate (4a): white solid. **^{1}H NMR (400 MHz, CDCl$_3$)** δ 7.35-7.45 (m, 5H), 7.21-7.28 (m, 3H), 7.13-7.15 (m, 1H), 5.43 (s, 2H), 2.98 (t, J = 7.2 Hz, 2H), 2.90 (d, J = 16.8 Hz, 2H), 2.77 (d, J = 16.8 Hz, 2H) , 2.10 (t, J = 7.2 Hz, 2H). **^{13}C NMR (100 MHz, CDCl$_3$)** δ 169.3, 151.0, 145.1, 142.9, 134.0, 129.0, 128.8, 128.6, 128.4, 127.4, 125.5, 121.9, 71.2, 44.9, 43.3, 37.5, 29.7. **HRMS(ESI)** calcd for $C_{21}H_{20}NO_4^+$ $(M+H)^+$: 350.1387, Found: 350.1385.

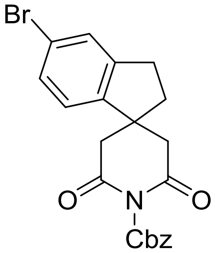

4b

Benzyl 5-bromo-2',6'-dioxo-2,3-dihydrospiro[indene-1,4'-piperidine]-1'-carboxyl ate (4b): white solid. **^{1}H NMR (400 MHz, CDCl$_3$)** δ 7.33-7.45 (m, 7H), 7.00 (d, J = 8.0 Hz 1H), 5.42 (s, 2H), 2.96 (t, J = 8.0 Hz, 2H), 2.81 (q, J = 16.0 Hz, J = 44.0 Hz, 4 H), 2.10 (t, J = 8.0 Hz, 2H). **^{13}C NMR (100 MHz, CDCl$_3$)** δ 168.9, 150.8, 145.2, 144. 1, 133.9, 130.5, 129.1, 128.8, 128.7, 128.6, 123.5, 122.3, 71.3, 44.6, 43.2, 37.6, 29.5. **HRMS (ESI)** calcd for $C_{21}H_{19}BrNO_4^+$ $(M+H)^+$: 428.0492, Found: 428.0490.

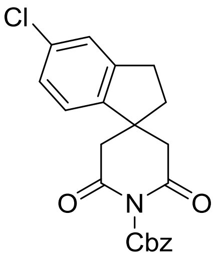

4c

Benzyl 5-chloro-2',6'-dioxo-2,3-dihydrospiro[indene-1,4'-piperidine]-1'-carboxylate (4c): white solid. **^{1}H NMR (400 MHz, $CDCl_3$)** *δ* 7.36-7.44 (m, 5H), 7.17-7.24 (m, 2H), 7.05 (d, *J* = 8 Hz 1H), 5.42 (s, 2H), 2.96 (t, *J* = 12.0 Hz, 2H), 2.81(q, *J* = 16.0 Hz, *J* = 40.0 Hz, 4H), 2.10 (t, *J* = 8.0 Hz, 2H). **^{13}C NMR (100 MHz, $CDCl_3$)** δ 168.9, 150.8, 144.9, 143.6, 134.2, 133.9, 129.0, 128.8, 128.6, 127.6, 125.7, 123.0, 71.3, 44.5, 43.2, 37.7, 29.5. **HRMS (ESI)** calcd for $C_{21}H_{19}ClNO_4^+$ $(M+H)^+$: 384.0997, Found: 384.0995.

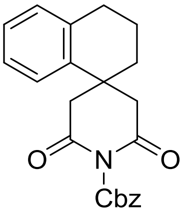

4j

(1'-((benzyloxy)carbonyl)-2'-oxo-3,4-dihydro-2H-spiro[naphthalene-1,4'-piperidin]-6'-ylidene)oxonium (4d): white solid. **^{1}H NMR (400 MHz, $CDCl_3$)** *δ* 7.37-7.46 (m, 5H), 7.17-7.25 (m, 3H), 7.13 (d, *J* = 4.0 Hz 4H), 3.05 (d, *J* = 20 Hz, 1H), 2.79-2.84 (m, 2H), 2.93 (m, 4H), 2.77 (d, *J* = 16.0 Hz, 1H), 2.23 (t, *J* = 8.0 Hz, 2H), 1.80-1.87 (m, 4H). **^{13}C NMR (100 MHz, $CDCl_3$)** δ 169.3, 151.1, 138.7, 137.4, 134, 130.2, 129.0, 128.9, 128.7, 127.3, 126.9, 125.8, 71.2, 45.2, 35.5, 33.6, 30.1, 18.9. **HRMS (ESI)** calcd for $C_{22}H_{21}ClNO_4^+$ $(M+H)^+$: 364.1543, Found: 364.1659.

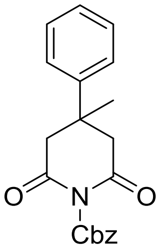

4k

Benzyl 4-methyl-2,6-dioxo-4-phenylpiperidine-1-carboxylate (4e): white solid. **^{1}H NMR (400 MHz, $CDCl_3$)** *δ* 7.25-7.38 (m, 10H), 5.34 (s, 2H), 3.16 (d, *J* = 16.8 Hz, 2H), 2.86 (d, *J* = 16.8 Hz, 2H), 1.45 (s, 3H). **^{13}C NMR (100 MHz, $CDCl_3$)** *δ* 169.3, 150.7, 143.5, 133.9, 129.3, 128.9, 128.7, 128.4, 127.6, 124.9, 71.1, 44.9, 36.7, 28.8.**HRMS (ESI)** calcd for $C_{20}H_{19}NNaO_4^+$ $(M+Na)^+$: 360.1206, Found: 360.1200.

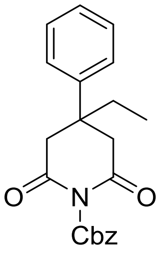

4l

Benzyl 4-ethyl-2,6-dioxo-4-phenylpiperidine-1-carboxylate (4f): white solid. **^{1}H NMR (400 MHz, $CDCl_3$)** δ 7.22-7.39 (m, 10H), 5.32 (s, 2H), 3.15 (d, *J* = 16.8 Hz, 2H), 2.86 (d, *J* = 16.8 Hz, 2H), 1.29 (q, *J* = 7.2 Hz, 2H), 0.67 (t, *J* = 7.2 Hz, 3H). **^{13}C NMR (100 MHz, $CDCl_3$)** δ169.4, 150.7, 141.2, 134.0, 129.2, 128.8, 128.7, 128.3, 127.5, 125.7, 70.9, 43.0, 40.5, 34.7, 8.3. **HRMS(ESI)** calcd for $C_{21}H_{21}NNaO_4^+$ $(M+Na)^+$: 374.1363, Found: 374.1360.

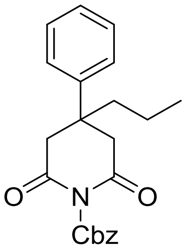

4m

Benzyl 2,6-dioxo-4-phenyl-4-propylpiperidine-1-carboxylate (4g): white solid. **^{1}H NMR (400 MHz, $CDCl_3$)** δ 7.30-7.37 (m, 7H), 7.20-7.27 (m, 3H), 5.30 (s, 2H), 3.15 (d, *J* = 16.4 Hz, 2H), 2.85 (d, *J* = 16.4 Hz, 2H),1.67-1.71 (m, 2H), 0.98-1.07 (m, 2H), 0.77-0.81 (m, 3H). **^{13}C NMR (100 MHz, $CDCl_3$)** δ 169.4, 150.7, 141.5, 134.0, 129.2, 128.8, 128.7, 128.3, 127.5, 125.6, 70.9, 44.4, 43.5, 40.2, 17.2, 14.3. **HRMS(ESI)** calcd for $C_{22}H_{23}NNaO_4^+$ $(M+Na)^+$: 388.1519, Found: 388.1516.

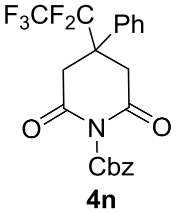

4n

Benzyl 2,6-dioxo-4-(perfluoroethyl)-4-phenylpiperidine-1-carboxylate (4i): white solid. **^{1}H NMR (400 MHz, $CDCl_3$)** δ 7.38-7.43 (m, 5H), 7.32-7.35 (m, 3H), 7.22-7.24 (m, 2H), 5.24 (s, 2H), 3.65 (d, *J* = 16.0 Hz 2H), 3.19 (d, *J* = 16.4 Hz 2H). **^{13}C NMR (100 MHz, $CDCl_3$)** δ 166.4, 149.5, 133.7, 131.3, 129.9, 129.6, 128.9, 128.7, 128.2, 127.4, 71.1, 45.4, 45.2, 45.0, 37.7. **HRMS(ESI)** calcd for $C_{21}H_{17}F_5NO_4^+$ $(M+H)^+$: 442.1072, Found: 442.1070.

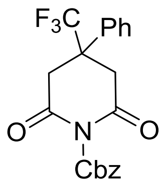

4o

Benzyl 2,6-dioxo-4-phenyl-4-(trifluoromethyl)piperidine-1-carboxylate (4h): **^{1}H NMR (400 MHz, CDCl$_3$)** δ 7.41-7.42 (m, 5H), 7.25-7.35 (m, 5H), 5.26 (s, 2H), 3.49-3.55 (m, 2H), 3.17-3.21 (m, 2H). **^{13}C NMR (100 MHz, CDCl$_3$)** δ 166.3, 149.6, 133.7, 132.0, 129.8, 129.6, 128.9, 128.7, 128.2, 127.2, 71.1, 45.8, 45.5, 37.4. **HRMS(ESI)** calcd for $C_{20}H_{16}F_3NNaO_4^+$ $(M+Na)^+$: 414.0924, Found: 414.0920.

b) Characterizations of Products

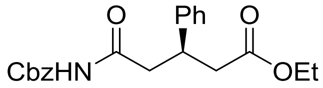

3a

Ethyl (*R*)-5-(((benzyloxy)carbonyl)amino)-5-oxo-3-phenylpentanoate (3a): Colorless liquid; **$[\alpha]_D$ ^{23}** (0.03, CHCl$_3$) = -328.9; **^{1}H NMR (400 MHz, CDCl$_3$)** δ 7.59 (s, 1H), 7.36 (d, J = 4.8 Hz, 5H), 7.23-7.29 (m,4H), 7.17-7.21 (m,1H), 5.14 (s, 2H), 3.98-4.06 (m,2H), 3.70-3.77 (m,1H), 3.07-3.25 (m, 2H), 2.61-2.78 (m, 2H), 1.13 (t, J = 7.2 Hz, 3H). **^{13}C NMR (100 MHz, CDCl$_3$)** δ 172.5, 171.9, 151.6, 142.8, 135.0, 128.9, 128.8, 128.7, 128.5, 127.5, 127.0, 68.0, 60.6, 42.1, 40.7, 37.5, 14.2. **HRMS(ESI)** calcd for $C_{21}H_{23}NNaO_5^+$ $(M+Na)^+$: 392.1468, Found: 392.1475; 95% *ee* as determined by **HPLC** (Chiralcel ODH, 90:10 hexanes/*i*-PrOH, 1.0 mL/min), t_r (major) = 50.7 min, t_r (minor) = 33.3 min.

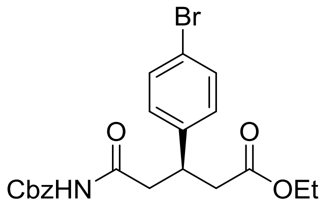

3b

Ethyl (*R*)-5-(((benzyloxy)carbonyl)amino)-3-(4-bromophenyl)-5-oxopentanoate (3b): Colorless liquid; **$[\alpha]_D$ ^{23}** (0.58,CHCl$_3$) = -25.8; **^{1}H NMR (400 MHz, CDCl$_3$)** δ 7.68 (s, 1H), 7.33-7.40 (m, 7H), 7.12 (dd, J =6.4, 2.0 Hz, 2H), 5.14 (s, 2H), 3.99-4.05 (m,2H), 3.67-3.74 (m,1H), 3.05-3.24 (m, 2H), 2.57-2.75 (m, 2H), 1.14 (t, J = 7.2 Hz, 3H). **^{13}C NMR (100 MHz, CDCl$_3$)** δ 172.5, 171.6, 151.8, 141.9, 135.0, 131.7, 129.4, 128.9, 128.8, 128.5, 120.7, 68.0, 60.7, 41.9, 40.5, 37.0, 14.2. **HRMS(ESI)** calcd for $C_{21}H_{22}BrNNaO_5^+$ $(M+Na)^+$: 470.0574, Found: 470.0582; 94% *ee* as determined by **HPLC** (Chiralcel ODH, 90:10 hexanes/*i*-PrOH, 1.0 mL/min), t_r (major) = 47.1 min, t_r (minor) = 40.1 min.

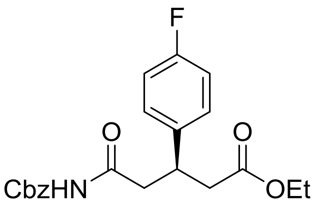

3c

Ethyl (*R*)-5-(((benzyloxy)carbonyl)amino)-3-(4-fluorophenyl)-5-oxopentanoate (3c): Colorless liquid; **[α]$_D$** ^{23} (c 0.46,CHCl$_3$) = -21.6; **^{1}H NMR (400 MHz, CDCl$_3$)** *δ* 7.65 (d, *J* = 6.0 Hz, 1H), 7.33-7.40 (m, 5H), 7.19-7.22 (m, 2H), 6.92-6.97 (m, 2H), 5.14 (s, 2H), 3.99-4.05 (m,2H), 3.69-3.76 (m,1H), 3.05-3.23 (m, 2H), 2.57-2.76 (m, 2H), 1.11-1.15 (m,3H). **^{13}C NMR (100 MHz, CDCl$_3$)** *δ* 172.5, 171.7, 163.0, 160.5, 151.7, 138.5, 135.0, 129.1, 129.0, 128.8, 128.5, 115.5, 115.3, 68.0, 60.6, 42.2, 40.7, 36.9, 14.2. **HRMS(ESI)** calcd for $C_{21}H_{22}FNNaO_5^+$ $(M+Na)^+$: 410.1374, Found: 410.1370; 94% *ee* as determined by **HPLC** (Chiralcel ODH, 90:10 hexanes/*i*-PrOH, 1.0 mL/min), t_r (major) = 42.7 min, t_r (minor) = 34.4 min.

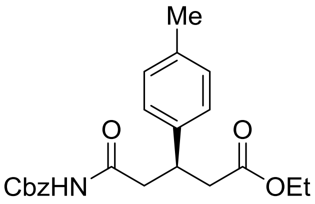

3d

Ethyl (*R*)-5-(((benzyloxy)carbonyl)amino)-5-oxo-3-(p-tolyl)pentanoate (3d): Colorless liquid; **[α]$_D$** ^{23} (0.45, CHCl$_3$) = -22.0; **^{1}H NMR (400 MHz, CDCl$_3$)** *δ* 7.79 (s, 1H), 7.32-7.36 (m, 5H), 7.05-7.13 (m, 4H), 5.13 (s, 2H), 4.01 (qd, *J* = 7.2, 2.0 Hz, 2H), 3.65-3.73 (m,1H), 3.03-3.19 (m, 2H), 2.58-2.74 (m, 2H), 2.28 (s, 3H), 1.11-1.15 (m,3H). **^{13}C NMR (100 MHz, CDCl$_3$)** *δ* 172.6, 172.0, 151.7, 139.8, 136.4, 135.1, 129.3, 128.8, 128.5, 127.4, 67.9, 60.5, 42.3, 40.8, 37.2, 21.1, 14.2. **HRMS(ESI)** calcd for $C_{22}H_{25}NNaO_5^+$ $(M+Na)^+$: 406.1625, Found: 406.1633; 95% *ee* as determined by **HPLC** (Chiralcel ODH, 90:10 hexanes/*i*-PrOH, 1.0 mL/min), t_r (major) = 34.3 min, t_r (minor) = 26.6 min.

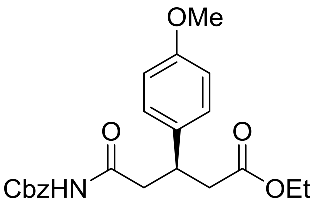

3e

Ethyl (*R*)-5-(((benzyloxy)carbonyl)amino)-3-(4-methoxyphenyl)-5-oxopentanoate (3e): Colorless liquid; **[α]$_D$** ^{23} (0.52, CHCl$_3$) = +38.6; **^{1}H NMR (400 MHz, CDCl$_3$)** *δ* 7.56 (s, 1H), 7.32-7.40 (m, 5H), 7.15-7.17 (m, 2H), 6.80-6.82 (m, 2H), 5.14 (s, 2H),

3.98-4.06 (m, 2H), 3.76 (s, 3H), 3.65-3.72 (m, 1H), 3.04-3.21 (m, 2H), 2.57-2.75(m, 2H), 1.14 (t, *J* = 7.2 Hz, 3H). **^{13}C NMR (100 MHz, CDCl$_3$)** δ 172.6, 172.0, 158.4, 151.7, 135.1, 134.9, 128.8, 128.6, 128.5, 114.0, 67.9, 60.5, 55.3, 42.4, 40.9, 36.9, 14.2. **HRMS(ESI)** calcd for $C_{22}H_{25}NNaO_6^+$ $(M+Na)^+$: 422.1574, Found: 422.1582; 96% *ee* as determined by **HPLC** (Chiralcel ODH, 90:10 hexanes/*i*-PrOH, 1.0 mL/min), t_r (major) = 56.7 min, t_r (minor) = 46.3 min.

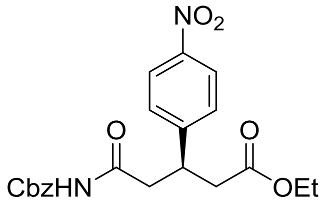

3f

Ethyl (*R*)-5-(((benzyloxy)carbonyl)amino)-3-(4-nitrophenyl)-5-oxopentanoate (3f): Colorless liquid; **$[\alpha]_D^{23}$** (0.23, CHCl$_3$) = +43.9; **^{1}H NMR (400 MHz, CDCl$_3$)** δ 8.12 (d, *J* = 8.8 Hz, 2H), 7.70 (s, 1H), 7.42 (d, *J* = 8.8 Hz, 2H), 7.32-7.38 (m,5H), 5.15 (s, 2H), 3.99-4.07 (m,2H), 3.82-3.90 (m,1H), 3.14-3.32 (m, 2H), 2.63-2.81 (m, 2H), 1.14 (t, *J* = 7.2 Hz, 3H). **^{13}C NMR (100 MHz, CDCl$_3$)** δ 172.1, 171.1, 151.8, 150.6, 146.9, 134.8, 129.0, 128.9, 128.7, 128.6, 123.9, 68.2, 60.9, 41.6, 40.1, 37.2, 14.2. **HRMS(ESI)** calcd for $C_{21}H_{22}N_2NaO_7^+$ $(M+Na)^+$: 437.1319, Found: 437.1311; 92% *ee* as determined by **HPLC** (Chiralcel ODH, 85:15 hexanes/*i*-PrOH, 1.0 mL/min), t_r (major) = 83.3 min, t_r (minor) = 65.0 min.

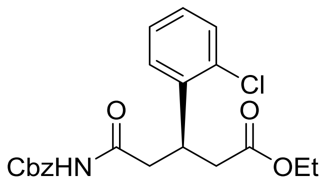

3g

Ethyl (*R*)-5-(((benzyloxy)carbonyl)amino)-3-(2-chlorophenyl)-5-oxopentanoate (3g): Colorless liquid; **$[\alpha]_D^{23}$** (0.44, CHCl$_3$) = -22.6; **^{1}H NMR (400 MHz, CDCl$_3$)** δ 7.69-7.72 (m,1H), 7.32-7.39 (m, 6H), 7.24-7.27 (m, 1H), 7.11-7.21 (m,2H), 5.15 (s, 2H), 4.20-4.27 (m,1H), 4.00-4.06 (m, 2H), 3.21 (dd, *J* = 7.2, 4.0 Hz, 2H), 2.69-2.83 (m, 2H), 1.13 (t, *J* = 7.2 Hz, 3H). **^{13}C NMR (100 MHz, CDCl$_3$)** δ 172.3, 171.7, 151.7, 140.0, 135.0, 133.9, 130.0, 128.8, 128.5, 128.2, 128.1, 127.1, 68.0, 60.6, 40.5, 38.9, 33.9, 14.1. **HRMS**(ESI) calcd for $C_{21}H_{22}ClNNaO_5^+$ $(M+Na)^+$: 426.1079, Found: 426.1078; 93% *ee* as determined by **HPLC** (Chiralcel ODH, 90:10 hexanes/*i*-PrOH, 1.0 mL/min), t_r (major) = 67.7 min, t_r (minor) = 37.8 min.

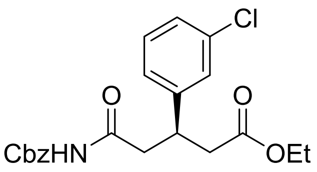

3h

Ethyl (*R*)-5-(((benzyloxy)carbonyl)amino)-3-(3-chlorophenyl)-5-oxopentanoate (3h): Colorless liquid; **$[\alpha]_D$ ^{23}** (0.52, $CHCl_3$) = +48.1; **^{1}H NMR (400 MHz, $CDCl_3$)** δ 7.61 (d, *J* = 8.0 Hz, 1H), 7.35-7.38 (m, 5H), 7.13-7.25 (m, 4H), 5.16 (s, 2H), 4.01-4.07 (m,2H), 3.69-3.76 (m,1H), 3.08-3.25 (m, 2H), 2.58-2.77 (m, 2H), 1.15 (t, *J* = 7.2 Hz, 3H). **^{13}C NMR (100 MHz, $CDCl_3$)** δ 172.3, 171.5, 151.7, 145.0, 135.0, 134.3, 129.9, 128.9, 128.8, 128.5, 127.8, 127.2, 125.9, 68.0, 60.7, 41.9, 40.4, 37.2, 14.2. **HRMS(ESI)** calcd for $C_{21}H_{22}ClNNaO_5^+$ $(M+Na)^+$: 426.1079, Found: 426.1071; 94% *ee* as determined by **HPLC** (Chiralcel ODH, 90:10 hexanes/*i*-PrOH, 1.0 mL/min), t_r (major) = 45.5 min, t_r (minor) = 33.4 min.

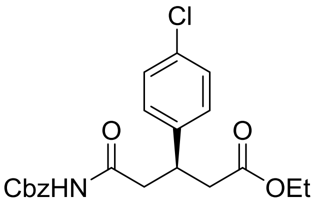

3i

Ethyl (*R*)-5-(((benzyloxy)carbonyl)amino)-3-(4-chlorophenyl)-5-oxopentanoate (3i): Colorless liquid; **$[\alpha]_D$ ^{23}** (0.52, $CHCl_3$) = -38.3; **^{1}H NMR (400 MHz, $CDCl_3$)** δ 7.85 (s, 1H), 7.32-7.39 (m, 5H), 7.15-7.22 (m, 4H), 5.13 (s, 2H), 3.98-4.04 (m,2H), 3.67-3.74 (m, 1H), 3.04-3.22 (m, 2H), 2.56-2.74 (m, 2H), 1.13 (t, *J* = 7.2 Hz, 3H). **^{13}C NMR (100 MHz, $CDCl_3$)** δ 172.5, 171.6, 151.8, 141.4, 135.0, 132.6, 129.0, 128.9, 128.8, 128.7, 128.5, 68.0, 60.6, 42.0, 40.5, 37.0, 14.2. **HRMS(ESI)** calcd for $C_{21}H_{22}ClNNaO_5^+$ $(M+Na)^+$: 426.1079, Found: 426.1086; 94% *ee* as determined by **HPLC** (Chiralcel ODH, 90:10 hexanes/*i*-PrOH, 1.0 mL/min), t_r (major) = 44.0 min, t_r (minor) = 36.9 min.

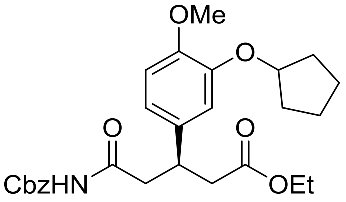

3j

Ethyl (*R*)-5-(((benzyloxy)carbonyl)amino)-3-(3-(cyclopentyloxy)-4-methoxyphenyl)-5-oxopentanoate (3j): Colorless liquid; **$[\alpha]_D$ ^{23}** (0.49, $CHCl_3$) = +173.3; **^{1}H NMR (400 MHz, $CDCl_3$)** δ 7.51 (d, *J* = 7.2 Hz, 1H), 7.34-7.38 (m, 5H), 6.76-6.79 (m, 3H), 5.14 (s, 2H), 4.73-4.78 (m,1H), 4.01-4.06 (m, 2H), 3.80 (d, *J* = 1.6

Hz, 3H), 3.63-3.71 (m,1H), 3.02-3.24 (m, 2H), 2.59-2.75 (m, 2H), 1.79-1.94 (m, 6H), 1.56-1.65 (m, 2H), 1.13-1.17 (m, 3H). **^{13}C NMR (100 MHz, $CDCl_3$)** δ 172.5, 171.9, 151.6, 148.9, 147.5, 135.3, 135.0, 128.8, 128.5, 119.3, 114.7, 112.1, 80.4, 67.9, 60.5, 56.1, 42.3, 40.8, 37.3, 32.9, 32.8, 24.1, 14.2. **HRMS(ESI)** calcd for $C_{27}H_{33}NNaO_7^+$ $(M+Na)^+$: 506.2149, Found: 506.2158; 93% *ee* as determined by **HPLC** (Chiralcel ODH, 90:10 hexanes/*i*-PrOH, 1.0 mL/min), t_r (major) = 45.3 min, t_r (minor) = 35.8 min.

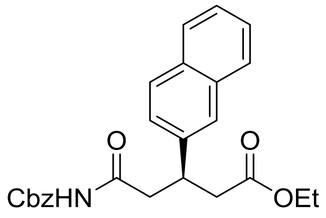

3k

Ethyl (*R*)-5-(((benzyloxy)carbonyl)amino)-3-(naphthalen-2-yl)-5-oxopentanoate (3k): Colorless liquid; **$[\alpha]_D^{23}$** (0.71, $CHCl_3$) = -14.1; **^{1}H NMR (400 MHz, $CDCl_3$)** δ 7.83 (d, *J* = 14.0 Hz, 1H), 7.73-7.77 (m, 3H), 7.68 (d, *J* = 2.0 Hz, 1H), 7.40-7.45 (m, 2H), 7.29-7.39 (m, 6H), 5.10 (s, 2H), 3.96-4.03 (m,2H), 3.87-3.95 (m, 1H), 3.12-3.32 (m,2H), 2.69-2.84 (m, 2H), 1.07-1.10 (m,3H). **^{13}C NMR (100 MHz, $CDCl_3$)** δ 172.6, 171.9, 151.7, 140.4, 135.0, 133.5, 132.6, 128.8, 128.5, 128.4, 127.9, 127.7, 126.2, 126.1, 126.0, 125.7, 67.9, 60.6, 42.1, 40.7, 37.7, 14.2. **HRMS(ESI)** calcd for $C_{25}H_{25}NNaO_5^+$ $(M+Na)^+$: 442.1625, Found: 442.1633; 96% *ee* as determined by **HPLC** (Chiralcel ODH, 90:10 hexanes/*i*-PrOH, 1.0 mL/min), t_r (major) = 71.6 min, t_r (minor) = 62.8 min.

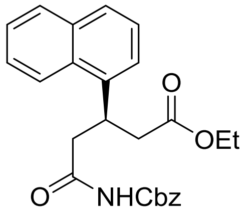

3l

Ethyl (*R*)-5-(((benzyloxy)carbonyl)amino)-3-(naphthalen-1-yl)-5-oxopentanoate (3l): Colorless liquid; **$[\alpha]_D^{23}$** (0.12, $CHCl_3$) = -40.8; **^{1}H NMR (400 MHz, $CDCl_3$)** δ 8.24 (d, *J* = 8.8 Hz, 1H), 7.84 (dd, *J* = 8.0, 1.6 Hz, 1H), 7.72 (dd, *J* = 7.2, 2.0 Hz, 1H), 7.46-7.55 (m, 3H), 7.32-7.43 (m, 7H), 5.14 (s, 2H), 4.64-4.72 (m,1H), 3.95-4.01 (m,2H), 3.27-3.39 (m, 2H), 2.79-2.90 (m, 2H), 1.05 (t, *J* = 7.2 Hz, 3H). **^{13}C NMR (100 MHz, $CDCl_3$)** δ 172.6, 172.0, 151.6, 139.2, 134.9, 134.1, 131.3, 129.0, 128.9, 128.8, 128.6, 127.5, 126.4, 125.7, 125.4, 123.3, 68.0, 60.6, 41.7, 40.4, 14.1. **HRMS(ESI)** calcd for $C_{25}H_{25}NNaO_5^+$ $(M+Na)^+$: 442.1625, Found: 442.1621; 98% *ee* as determined by **HPLC** (Chiralcel ODH, 85:15 hexanes/*i*-PrOH, 1.0 mL/min), t_r (major) = 52.7 min, t_r (minor) = 30.0 min.

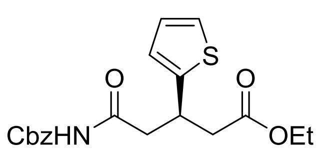

3m

Ethyl (*R*)-5-(((benzyloxy)carbonyl)amino)-5-oxo-3-(thiophen-2-yl)pentanoate (3m): Colorless liquid; **[α]$_D$ ^{23}** (0.48, $CHCl_3$) = +52.5; **^{1}H NMR (400 MHz, $CDCl_3$)** δ 7.73-7.77 (m,1H), 7.32-7.39 (m, 5H), 7.11-7.13 (m, 1H), 6.88 (d, *J* = 3.6 Hz, 2H), 5.15 (s, 2H), 4.03-4.11 (m,3H), 3.12-3.29 (m, 2H), 2.66-2.81 (m, 2H), 1.18 (t, *J* = 7.2 Hz, 3H). **^{13}C NMR (100 MHz, $CDCl_3$)** *δ* 172.1, 171.5, 151.6, 146.3, 135.0, 128.8, 128.5, 126.8, 124.4, 123.7, 68.0, 60.7, 43.0, 41.5, 32.9, 14.2. **HRMS(ESI)** calcd for $C_{19}H_{21}NNaO_5S^+$ $(M+Na)^+$: 398.1033, Found: 398.1042; 85% *ee* as determined by **HPLC** (Chiralcel ODH, 90:10 hexanes/*i*-PrOH, 1.0 mL/min), t_r (major) = 54.4 min, t_r (minor) = 40.7 min.

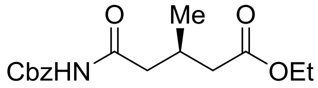

3n

Ethyl (*R*)-5-(((benzyloxy)carbonyl)amino)-3-methyl-5-oxopentanoate (3n): white solid; **[α]$_D$ ^{23}** (0.07, $CHCl_3$) = -138.9; **^{1}H NMR (400 MHz, $CDCl_3$)** *δ* 7.62 (s, 1H), 7.30 (s, 5H), 5.10 (s, 2H), 4.03-4.08 (m, 2H), 2.59-2.75 (m, 2H), 2.42-2.50 (m,1H), 2.15-2.36 (m, 2H), 1.18 (t, *J* = 7.2 Hz, 3H), 0.97 (d, *J* = 6.8 Hz, 3H). **^{13}C NMR (100 MHz, $CDCl_3$)** *δ* 173.0, 172.7, 151.6, 135.0, 128.8, 128.5, 67.9, 60.5, 42.5, 40.9, 26.9, 20.1, 14.3. **HRMS(ESI)** calcd for $C_{16}H_{21}NNaO_5^+$ $(M+Na)^+$: 330.1312, Found: 330.1310; 82% *ee* as determined by **HPLC** (Chiralcel AZH, 85:15 hexanes/*i*-PrOH, 1.0 mL/min), t_r (major) = 18.0 min, t_r (minor) = 13.9 min.

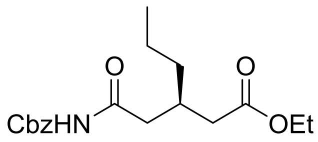

3o

Ethyl (R)-3-(2-(((benzyloxy)carbonyl)amino)-2-oxoethyl)hexanoate (3o): Colorless liquid; **[α]$_D$ ^{23}** (0.03, $CHCl_3$) = -128.9; **^{1}H NMR (400 MHz, $CDCl_3$)** δ 7.86 (s, 1H), 7.34-7.40 (m, 5H), 5.16 (s,2H), 4.11 (q, J = 6.8, 2H), 2.68-2.80 (m, 2H), 2.34-2.42 (m, 3H), 1.31-1.37 (m, 4H), 1.23 (t, J = 7.6, 3H), 0.87-0.90 (m, 3H). **^{13}C NMR (100 MHz, $CDCl_3$)** δ 173.2, 173.0, 151.5, 135.1, 128.8, 128.5, 67.9, 60.5, 40.5, 38.4, 36.5, 31.4, 19.9, 14.3, 14.2. **HRMS(ESI)** calcd for $C_{18}H_{25}NNaO_5^+$ $(M+Na)^+$: 358.1625, Found: 358.1631; 85% *ee* as determined by **HPLC** (Chiralcel ODH, 98:2 hexanes/*i*-PrOH, 1.0 mL/min), t_r (major) = 111.1 min, t_r (minor) = 128.0min.

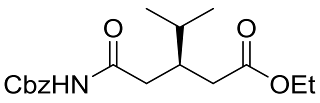

3p

Ethyl (*R*)-5-(((benzyloxy)carbonyl)amino)-3-isopropyl-5-oxopentanoate (3p): Colorless liquid; **[α]$_D$ ^{23}** (0.03, $CHCl_3$) = -34.78. **^{1}H NMR (400 MHz, $CDCl_3$)** δ 7.98 (s, 1H), 7.33-7.37 (m, 5H), 5.16 (s, 2H), 4.09 (q, *J* = 7.2 Hz, 2H), 2.60-2.81 (m, 2H), 2.24-2.38 (m, 3H), 1.73-1.81 (m, 1H), 1.22 (t, *J* = 6.0 Hz, 3H), 0.87-0.89 (m, 4H). **^{13}C NMR (100 MHz, $CDCl_3$)** δ 173.6, 173.5, 151.6, 135.1, 128.8, 128.7, 128.6, 128.5, 67.8, 60.6, 38.0, 37.0, 35.9, 30.7, 19.2, 19.1, 14.3. **HRMS(ESI)** calcd for $C_{18}H_{25}NNaO_5^+$ $(M+Na)^+$: 358.1625, Found: 358.1627; 95% *ee* as determined by **HPLC** (Chiralcel ODH, 98:2 hexanes/*i*-PrOH, 1.0 mL/min), t_r (major) = 96.8 min, t_r (minor) = 113.3 min.

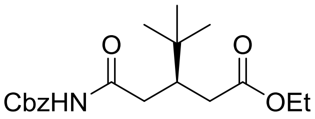

3q

Ethyl (*R*)-5-(((benzyloxy)carbonyl)amino)-3-(tert-butyl)-5-oxopentanoa (3q): Colorless liquid. **[α]$_D$ ^{23}** (0.03, $CHCl_3$) = -24.64. **^{1}H NMR (400 MHz, $CDCl_3$)** δ 8.02 (s, 1H), 7.34-7.38 (m, 5H), 5.18 (s, 2H), 4.10 (q, *J* = 7.2 Hz, 2H), 2.84-2.90 (m, 1H), 2.48-2.55 (m, 2H), 2.31-2.37 (m, 1H), 2.16-2.22 (m, 1H), 1.23 (t, *J* = 7.2 Hz, 4H), 0.91 (s, 9H). **^{13}C NMR (100 MHz, $CDCl_3$)** δ 174.2, 151.5, 135.2, 128.8, 128.7, 128.5, 67.7, 60.8, 40.8, 38.2, 35.8, 33.5, 27.3, 14.2. **HRMS(ESI)** calcd for $C_{19}H_{27}NNaO_5^+$ $(M+Na)^+$: 372.1781, Found: 372.1787; 92% *ee* as determined by **HPLC** (Chiralcel AZH, 90:10 hexanes/*i*-PrOH, 1.0 mL/min), t_r (major) = 13.4 min, t_r (minor) = 17.5 min.

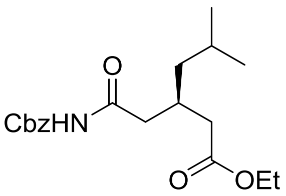

3r

Ethyl (*R*)-3-(2-(((benzyloxy)carbonyl)amino)-2-oxoethyl)-5-methylhexanoate (3r): Colorless liquid; **[α]$_D$ ^{23}** (0.44, $CHCl_3$) = -68.7; **^{1}H NMR (400 MHz, $CDCl_3$)** δ 7.92 (d, *J* = 6.4 Hz, 1H), 7.34-7.39 (m,5H), 5.17 (s, 2H), 4.07-4.15 (m, 2H), 2.74 (dd, *J* = 6.4, 1.2 Hz,, 2H), 2.43-2.50 (m, 1H), 2.36 (dd, *J* = 6.4, 1.2 Hz, 2H), 1.59-1.67 (m, 1H), 1.20-1.26 (m, 5H), 0.88-0.90 (m, 6H). **^{13}C NMR (100 MHz, $CDCl_3$)** δ 173.2, 173.0, 151.6, 135.2, 128.8, 128.7, 128.5, 67.8, 60.5, 43.7, 40.6, 38.6, 29.4, 25.2, 22.7, 22.6, 14.3. **HRMS(ESI)** calcd for $C_{19}H_{27}NNaO_5^+$ $(M+Na)^+$: 372.1781, Found: 372.1788; 87% *ee* as determined by **HPLC** (Chiralcel ODH, 95:5 hexanes/*i*-PrOH, 1.0 mL/min), t_r (major) = 38.4 min, t_r (minor) = 33.3 min.

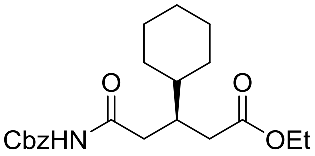

3s

Ethyl (*R*)-5-(((benzyloxy)carbonyl)amino)-3-cyclohexyl-5-oxopentanoate (3s): Colorless liquid; **$[\alpha]_D^{23}$** (0.6, $CHCl_3$) = -46.07; **^{1}H NMR (400 MHz, $CDCl_3$)** δ 7.88 (s, 1H), 7.34-7.37 (m, 5H), 5.17 (s, 2H), 4.10 (q, *J* = 6.8 Hz, 2H), 2.61-2.84 (m, 2H), 2.26-2.41 (m, 3H), 1.64-1.75 (s, 6H), 1.23 (t, *J* = 7.2 Hz, 4H), 0.96-1.16 (s, 4H). **^{13}C NMR (100 MHz, $CDCl_3$)** δ 173.6, 151.6, 135.1, 128.8, 128.8, 128.5, 67.8, 60.6, 41.1, 38.3, 36.6, 36.2, 29.8, 29.7, 26.6, 26.6, 14.3. **HRMS(ESI)** calcd for $C_{21}H_{29}NNaO_5^+$ $(M+Na)^+$: 398.1938, Found: 398.1939; 92% *ee* as determined by **HPLC** (Chiralcel IA, 95:5 hexanes/*i*-PrOH, 1.0 mL/min), t_r (major) = 21.4 min, t_r (minor) = 25.4 min.

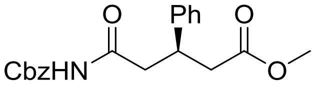

3t

Methyl (*R*)-5-(((benzyloxy)carbonyl)amino)-5-oxo-3-phenylpentanoate (3t): Colorless liquid; **$[\alpha]_D^{23}$** (0.5, $CHCl_3$) = -100.00; **^{1}H NMR (400 MHz, $CDCl_3$)** δ 7.66 (s, 1H), 7.28-7.40 (m, 7H), 7.18-7.25 (m, 3H), 5.15 (s, 2H), 3.70-3.78 (m, 1H), 3.57 (s, 3H), 3.08-3.25 (m, 2H), 2.63-2.79 (m, 2H). **^{13}C NMR (100 MHz, $CDCl_3$)** δ 172.5, 172.3, 151.7, 142.9, 135.0, 128.8, 128.7, 128.7, 128.5, 128.4, 128.3, 127.5, 127.0, 68.0, 67.1, 51.7, 42.1, 40.5, 37.5. **HRMS(ESI)** calcd for $C_{20}H_{22}NO_5^+$ $(M+H)^+$: 356.1492, Found: 356.1495.; 80% *ee* as determined by **HPLC** (Chiralcel ASH, 90:10 hexanes/*i*-PrOH, 1.0 mL/min), t_r (major) = 42.8 min, t_r (minor) = 57 min.

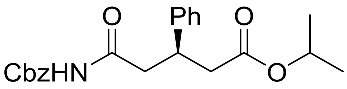

3u

Isopropyl (*R*)-5-(((benzyloxy)carbonyl)amino)-5-oxo-3-phenylpentanoate (3u): Colorless liquid; **$[\alpha]_D^{23}$** (0.6, $CHCl_3$) = -324.36; **^{1}H NMR (400 MHz, $CDCl_3$)** δ 7.88 (s, 1H), 7.33-7.38 (m, 5H), 7.22-7.26 (m, 4H), 7.16-7.20 (m, 1H), 5.14(s, 2H), 4.84-4.91 (m, 1H), 3.68-3.76 (m, 1H) 3.19 (dd, J = 4 Hz, J = 8 Hz, 1H), 3.06 (dd, *J* = 4 Hz, *J* = 8 Hz, 1H), 2.60 (dd, J = 4 Hz, J = 8 Hz, 1H), 2.58-2.63 (dd, J = 4 Hz, J = 8 Hz 1H), 1.09 (dd, J = 4 Hz, J = 6 Hz). **^{13}C NMR (100 MHz, $CDCl_3$)** δ 171.2, 170.2, 151.3, 136.7, 134.0, 129.1, 129.0, 128.8, 128.7, 128.3, 128.0, 71.2, 48.0, 31.0, 25.4. **HRMS (ESI)** calcd for $C_{24}H_{25}ClNO_5^+$ $(M+H)^+$: 384.1805, Found: 384.1801. 82% *ee* as determined by **HPLC** (Chiralcel OJH, 90:10 hexanes/*i*-PrOH, 1.0 mL/min), t_r (major) = 67.7 min, t_r (minor) = 82.6min.

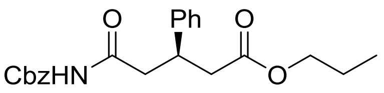

3v

Propyl (*R*)-5-(((benzyloxy)carbonyl)amino)-5-oxo-3-phenylpentanoate (3v): Colorless liquid; **[α]$_D$ ^{23}** (0.22, $CHCl_3$) = -464.7; **^{1}H NMR (400 MHz, $CDCl_3$)** *δ* 7.31-7.38 (m, 5H), 7.21-7.28 (m, 4H), 7.16-7.20 (m, 1H), 5.13 (s, 2H), 3.91 (t, *J* = 6.8 Hz, 2H), 3.69-3.76 (m, 1H), 3.06-3.23 (m, 2H), 2.61-2.78 (m, 2H), 1.51 (dt, *J* = 21.2, 7.2 Hz, 2H), 0.80-0.84 (m, 3H). **^{13}C NMR (100 MHz, $CDCl_3$)** *δ* 172.5, 172.0, 151.6, 142.8, 135.0, 128.8, 128.7, 128.5, 127.5, 127.0, 68.0, 66.2, 53.6, 42.2, 40.7, 37.6, 21.9, 10.4. **HRMS(ESI)** calcd for $C_{22}H_{25}NNaO_5^+$ $(M+Na)^+$: 406.1625, Found: 406.1630; 94% *ee* as determined by **HPLC** (Chiralcel ODH, 85:15 hexanes/*i*-PrOH, 1.0 mL/min), t_r (major) = 29.8 min, t_r (minor) = 20.5 min.

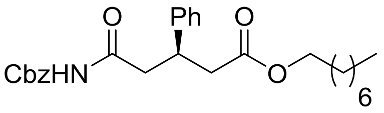

3w

Octyl (*R*)-5-(((benzyloxy)carbonyl)amino)-5-oxo-3-phenylpentanoate (3w): White solid; **[α]$_D$ ^{23}** (0.19, $CHCl_3$) = -53.0; **^{1}H NMR (400 MHz, $CDCl_3$)** *δ* 7.25-7.33 (m, 5H), 7.15-7.22 (m, 4H), 7.09-7.13 (m, 1H), 5.07 (s, 2H), 3.86-3.89 (m, 2H), 3.66 (dd, *J* = 8.0, 6.8 Hz, 1H), 3.00-3.17 (m, 2H), 2.55-2.71 (m, 2H), 1.37-1.44 (m, 2H), 1.11-1.24 (m, 10H), 0.79-0.83 (m, 3H). **^{13}C NMR (100 MHz, $CDCl_3$)** *δ* 172.4, 172.0, 151.6, 142.8, 135.0, 128.9, 128.8, 128.7, 128.5, 127.5, 127.0, 68.0, 64.8, 42.2, 40.7, 37.6, 31.9, 29.3, 29.3, 28.6, 25.9, 22.8, 14.2. **HRMS(ESI)** calcd for $C_{27}H_{35}NNaO_5^+$ $(M+Na)^+$: 476.2407, Found: 476.2401; 93% *ee* as determined by **HPLC** (Chiralcel ODH, 90:10 hexanes/*i*-PrOH, 1.0 mL/min), t_r (major) = 36.1 min, t_r (minor) = 25.8 min.

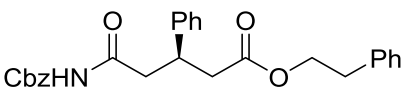

3x

Phenethyl (*R*)-5-(((benzyloxy)carbonyl)amino)-5-oxo-3-phenylpentanoate (3x): Colorless liquid; [α]$_D$ ^{23} (0.18, $CHCl_3$) = -27.3; **^{1}H NMR (400 MHz, $CDCl_3$)** *δ* 7.42 (s, 1H), 7.25-7.32 (m, 5H), 7.10-7.22 (m, 8H), 7.05-7.08 (m, 2H), 5.07 (s, 2H), 4.09-4.13 (m, 2H), 3.64 (t, *J* = 7.2 Hz, 1H), 2.96-3.15 (m, 2H), 2.72-2.75 (m, 2H), 2.53-2.70 (m, 2H). **^{13}C NMR (100 MHz, $CDCl_3$)** *δ* 172.4, 171.8, 151.6, 142.8, 137.9, 135.0, 129.0, 128.9, 128.8, 128.7, 128.6, 128.6, 127.5, 127.0, 126.6, 68.0, 65.1, 42.1, 40.7, 37.5, 35.0. **HRMS(ESI)** calcd for $C_{27}H_{27}NNaO_5^+$ $(M+Na)^+$: 468.1781, Found: 468.1784; 95% *ee* as determined by HPLC (Chiralcel AZH, 85:15 hexanes/*i*-PrOH, 1.0 mL/min), t_r (major) = 37.8 min, t_r (minor) = 34.0 min.

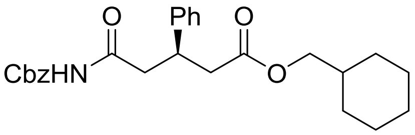

3y

Cyclohexylmethyl (*R*)-5-(((benzyloxy)carbonyl)amino)-5-oxo-3-phenylpentanoate (3y): Colorless liquid; **[α]$_D$ ^{23}** (0.10, $CHCl_3$) = -49.6; **^{1}H NMR (400 MHz, $CDCl_3$)** *δ* 7.42 (s, 1H), 7.26-7.33 (m, 5H), 7.16-7.22 (m, 5H), 7.10-7.14 (m, 1H), 5.07 (s, 2H), 3.63-3.74 (m, 3H), 3.00-3.18 (m, 2H), 2.56-2.72 (m, 2H), 1.54-1.61 (m, 3H), 1.47-1.51 (m, 2H), 1.35-1.45 (m, 1H), 1.01-1.15 (m, 3H), 0.70-0.80 (m,2H). **^{13}C NMR (100 MHz, $CDCl_3$)** *δ* 172.4, 172.0, 151.6, 142.8, 135.0, 128.8, 128.7, 128.5, 127.5, 127.0, 69.8, 68.0, 42.2, 40.7, 37.7, 37.1, 29.6, 26.4, 25.7. **HRMS(ESI)** calcd for $C_{26}H_{31}NNaO_5^+$ $(M+Na)^+$: 460.2094, Found: 460.2090; 90% *ee* as determined by **HPLC** (Chiralcel ODH, 90:10 hexanes/*i*-PrOH, 1.0 mL/min), t_r (major) = 45.1 min, t_r (minor) = 29.6 min.

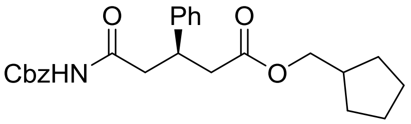

3z

Cyclopentylmethyl (*R*)-5-(((benzyloxy)carbonyl)amino)-5-oxo-3-phenylpentanoate (3z): Colorless liquid; **[α]$_D$ ^{23}** (0.09, $CHCl_3$) = -53.0; **^{1}H NMR (400 MHz, $CDCl_3$)** *δ* 7.49 (s, 1H), 7.33-7.39 (m, 5H), 7.17-7.29 (m, 5H), 5.14 (s, 2H), 3.85 (d, *J* = 7.2 Hz, 2H), 3.70-3.77 (m,1H), 3.08-3.25 (m, 2H), 2.63-2.79 (m, 2H), 2.02-2.10 (m, 1H), 1.47-1.64 (m, 6H), 1.07-1.16 (m, 2H). **^{13}C NMR (100 MHz, $CDCl_3$)** *δ* 172.4, 172.0, 151.6, 142.8, 135.0, 128.8, 128.7, 128.5, 127.5, 127.0, 68.6, 68.0, 42.2, 40.7, 38.5, 37.6, 29.4, 29.3, 25.4, 25.3. **HRMS(ESI)** calcd for $C_{25}H_{29}NNaO_5^+$ $(M+Na)^+$: 446.1938, Found: 446.1931; 90% *ee* as determined by **HPLC** (Chiralcel ODH, 90:10 hexanes/*i*-PrOH, 1.0 mL/min), t_r (major) = 45.2 min, t_r (minor) = 29.9 min.

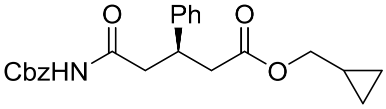

3aa

Cyclopropylmethyl (*R*)-5-(((benzyloxy)carbonyl)amino)-5-oxo-3-phenylpentanoate (3aa): Colorless liquid; **[α]$_D$ ^{23}** (0.45, $CHCl_3$) = -33.2; **^{1}H NMR (400 MHz, $CDCl_3$)** *δ* 7.77 (s, 1H), 7.31-7.38 (m, 5H), 7.23-7.27 (m, 4H), 7.16-7.20 (m, 1H), 5.14 (s, 2H), 3.79 (dd, *J* = 7.2, 2.8 Hz, 2H), 3.72-3.76 (m, 1H), 3.07-3.23 (m, 2H), 2.63-2.80 (m, 2H), 0.94-1.04 (m, 1H), 0.45-0.49 (m, 2H), 0.14-0.18 (m,2H). **^{13}C NMR (100 MHz, $CDCl_3$)** *δ* 172.5, 172.0, 151.7, 142.8, 135.0, 128.8, 128.6, 128.5,

127.6, 127.0, 69.4, 67.9, 42.2, 40.7, 37.6, 9.8, 3.3, 3.2. **HRMS(ESI)** calcd for $C_{23}H_{25}NNaO_5^+$ $(M+Na)^+$: 418.1625, Found: 418.1630; 94% *ee* as determined by **HPLC** (Chiralcel ODH, 90:10 hexanes/*i*-PrOH, 1.0 mL/min), t_r (major) = 53.9 min, t_r (minor) = 36.0 min.

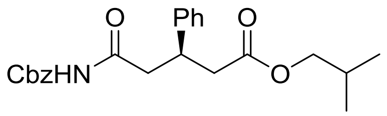

3ab

Isobutyl (*R*)-5-(((benzyloxy)carbonyl)amino)-5-oxo-3-phenylpentanoate (3w): Colorless liquid; $[\alpha]_D^{23}$ (0.22, $CHCl_3$) = -46.3; **^{1}H NMR (400 MHz, $CDCl_3$)** δ 7.54 (s, 1H), 7.25-7.31 (m, 5H), 7.16-7.19 (m, 4H), 7.09-7.13 (m, 1H), 5.07 (s, 2H), 3.63-3.70 (m, 3H), 2.99-3.17 (m, 2H), 2.56-2.72 (m, 2H), 1.67-1.77 (m, 1H), 0.74 (d, *J* = 6.8 Hz, 6H). **^{13}C NMR (100 MHz, $CDCl_3$)** δ 172.4, 172.0, 151.6, 142.8, 135.0, 128.8, 128.7, 128.5, 127.5, 127.0, 70.8, 68.0, 42.2, 40.6, 37.6, 27.7, 19.1. **HRMS(ESI)** calcd for $C_{23}H_{27}NNaO_5^+$ $(M+Na)^+$: 420.1781, Found: 420.1790; 95% *ee* as determined by **HPLC** (Chiralcel ODH, 90:10 hexanes/*i*-PrOH, 1.0 mL/min), t_r (major) = 40.4 min, t_r (minor) = 27.7 min.

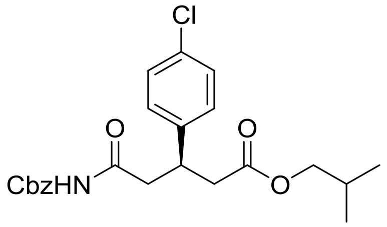

3ac

Isobutyl (*R*)-5-(((benzyloxy)carbonyl)amino)-3-(4-chlorophenyl)-5-oxopentanoate (3w): Colorless liquid; $[\alpha]_D^{23}$ (0.46, $CHCl_3$) = -43.8; **^{1}H NMR (400 MHz, $CDCl_3$)** δ 7.49 (s, 1H), 7.32-7.39 (m, 5H), 7.16-7.24 (m, 4H), 5.13 (s, 2H), 3.74 (dd, *J* =8.0, 1.6 Hz, 2H), 3.68-3.72 (m, 1H), 3.05-3.24 (m, 2H), 2.59-2.77 (m, 2H), 1.78 (td, *J* = 13.2, 6.8 Hz, 1H), 0.82 (d, *J* = 6.8 Hz, 6H). **^{13}C NMR (100 MHz, $CDCl_3$)** δ 172.5, 171.7, 151.8, 141.4, 135.0, 132.6, 129.0, 128.8, 128.7, 128.5, 70.8, 68.0, 42.1, 40.5, 37.0, 27.7, 19.1. **HRMS(ESI)** calcd for $C_{23}H_{26}ClNNaO_5^+$ $(M+Na)^+$: 454.1392, Found: 454.1397; 94% *ee* as determined by **HPLC** (Chiralcel ODH, 90:10 hexanes/*i*-PrOH, 1.0 mL/min), t_r (major) = 37.1 min, t_r (minor) = 31.0 min.

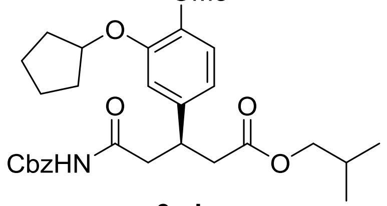

3ad

Isobutyl (*R*)-5-(((benzyloxy)carbonyl)amino)-3-(3-(cyclopentyloxy)-4-methoxyphenyl)-5-oxopentanoate (3ad): White solid; **[α]$_D$^{23}** (0.56, $CHCl_3$) = -45.0; **^{1}H NMR (400 MHz, $CDCl_3$)** *δ* 7.74-7.77 (m, 1H), 7.32-7.40 (m, 5H), 6.75-6.78 (m, 3H), 5.13 (s, 2H), 4.72-4.77 (m, 1H), 3.78 (s, 3H), 3.74-3.76 (m,2H), 3.66 (dd, *J* = 14.8, 7.2 Hz, 1H), 3.00-3.21 (m, 2H), 2.61-2.76 (m, 2H), 1.75-1.95 (m, 8H), 1.57-1.61 (m, 1H), 0.83 (d, *J* = 6.8 Hz, 6H). **^{13}C NMR (100 MHz, $CDCl_3$)** *δ* 172.5, 172.0, 151.6, 148.9, 147.6, 135.3, 135.0, 128.8, 128.4, 119.3, 114.7, 112.1, 80.4, 70.7, 67.9, 56.1, 42.4, 40.8, 37.3, 32.9, 32.8, 27.7, 24.1, 19.1. **HRMS(ESI)** calcd for $C_{29}H_{37}NNaO_7^+$ $(M+Na)^+$: 534.2462, Found: 534.2460; 94% *ee* as determined by **HPLC** (Chiralcel ODH, 90:10 hexanes/*i*-PrOH, 1.0 mL/min), t_r (major) = 37.6 min, t_r (minor) = 32.1 min.

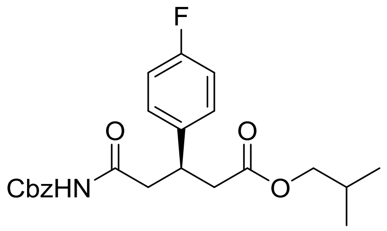

3ae

Isobutyl (*R*)-5-(((benzyloxy)carbonyl)amino)-3-(4-fluorophenyl)-5-oxopentanoate (3ae): Colorless liquid; **[α]$_D$^{23}** (0.34, $CHCl_3$) = -14.7; **^{1}H NMR (400 MHz, $CDCl_3$)** *δ* 7.68 (d, *J* = 30.8 Hz, 1H), 7.32-7.40 (m, 5H), 7.19-7.22 (m, 2H), 6.92-6.96 (m, 2H), 5.14 (s, 2H), 3.69-3.78 (m, 3H), 3.05-3.23 (m, 2H), 2.60-2.78 (m, 2H), 1.74-1.83 (m,1H), 0.82 (d, *J* = 6.8 Hz, 6H). **^{13}C NMR (100 MHz, $CDCl_3$)** *δ* 172.6, 171.8, 163.0, 160.5, 151.8, 138.5, 138.5, 135.0, 129.1, 129.0, 128.8, 128.5, 115.5, 115.3, 70.8, 68.0, 42.3, 40.7, 36.9, 27.7, 19.1. **HRMS(ESI)** calcd for $C_{23}H_{26}FNNaO_5^+$ $(M+Na)^+$: 438.1687, Found: 438.1692; 92% *ee* as determined by **HPLC** (Chiralcel ODH, 90:10 hexanes/*i*-PrOH, 1.0 mL/min), t_r (major) = 35.7 min, t_r (minor) = 28.9 min.

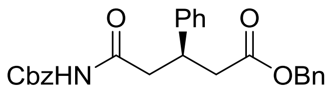

3af

Benzyl (*R*)-5-(((benzyloxy)carbonyl)amino)-5-oxo-3-phenylpentanoate (3af): Colorless liquid; **[α]$_D$^{23}** (0.27, $CHCl_3$) = +55.8; **^{1}H NMR (400 MHz, $CDCl_3$)** *δ* 7.70-7.73 (m, 1H), 7.31-7.37 (m, 5H), 7.26-7.29 (m,3H), 7.16-7.25 (m, 7H), 5.12 (s, 2H), 4.99 (s, 2H), 3.71-3.79 (m, 1H), 3.05-3.22 (m, 2H), 2.67-2.83 (m, 2H). **^{13}C NMR (100 MHz, $CDCl_3$)** *δ* 172.6, 171.7, 151.7, 142.7, 135.9, 135.1, 128.8, 128.7, 128.6, 128.5, 128.3, 128.2, 127.6, 127.0, 68.0, 66.4, 42.2, 40.6, 37.6. **HRMS(ESI)** calcd for $C_{26}H_{25}NNaO_5^+$ $(M+Na)^+$: 454.1625, Found: 454.1633; 85% *ee* as determined by **HPLC** (Chiralcel ODH, 90:10 hexanes/*i*-PrOH, 1.0 mL/min), t_r (major) = 89.9 min, t_r (minor) = 67.1 min.

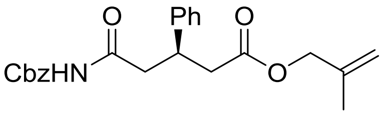

3ag

2-Methylallyl (*R*)-5-(((benzyloxy)carbonyl)amino)-5-oxo-3-phenylpentanoate (3ag):$[\alpha]_D^{23}$ (0.29, $CHCl_3$) = -34.2;**^{1}H NMR (400 MHz, $CDCl_3$)** δ 7.66 (s, 1H), 7.33-7.36 (m, 5H), 7.24-7.26 (m, 4H), 7.17-7.21 (m, 1H), 5.13 (s, 2H), 4.83 (s, 2H), 4.38 (s, 2H), 3.71-3.79 (m, 1H), 3.07-3.25 (m, 2H), 2.67-2.84 (m, 2H), 1.62 (s, 3H). **^{13}C NMR (100 MHz, $CDCl_3$)** δ 172.5, 171.6, 151.6, 142.8, 139.9, 135.0, 128.8, 128.7, 128.5, 127.5, 127.0, 113.1, 68.0, 67.9, 42.2, 40.5, 37.5, 19.5. **HRMS(ESI)** calcd for $C_{23}H_{25}NNaO_5^+$ $(M+Na)^+$: 418.1625, Found: 418.1621; 93% *ee* as determined by **HPLC** (Chiralcel ODH, 85:15 hexanes/*i*-PrOH, 1.0 mL/min), t_r (major) = 31.3 min, t_r (minor) = 23.1 min.

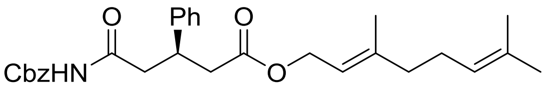

3ah

(E)-3,7-dimethylocta-2,6-dien-1-yl (*R*)-5-(((benzyloxy)carbonyl)amino)-5-oxo-3-phenylpentanoate (3ab): White solid; **$[\alpha]_D^{23}$** (0.29, $CHCl_3$) = -51.8; **^{1}H NMR (400 MHz, $CDCl_3$)** δ 7.67 (s, 1H), 7.32-7.37 (m, 5H), 7.21-7.28 (m, 5H), 7.15-7.20 (m, 1H), 5.18-5.22 (m,1H), 5.13 (s, 2H), 5.05-5.09 (m,1H), 4.47-4.50 (dd, *J* = 7.1, 4.0 Hz, 2H), 3.70-3.77 (m,1H), 3.06-3.23 (m, 2H), 2.61-2.78 (m, 2H), 2.04-2.09 (m, 2H), 1.97-2.01 (m,2H), 1.61 (dd, *J* = 9.6, 1.2 Hz, 6H). **^{13}C NMR (100 MHz, $CDCl_3$)** δ 172.5, 171.9, 151.6, 142.8, 142.4, 135.0, 131.9, 128.8, 128.6, 128.5, 127.6, 127.0, 123.9, 118.2, 68.0, 61.5, 42.1, 40.7, 39.6, 37.6, 26.4, 25.8, 17.8, 16.5. **HRMS(ESI)** calcd for $C_{29}H_{35}NNaO_5^+$ $(M+Na)^+$: 500.2407, Found: 500.2415; 91% *ee* as determined by **HPLC** (Chiralcel IB, 90:10 hexanes/*i*-PrOH, 1.0 mL/min), t_r (major) = 15.6 min, t_r (minor) = 13.1 min.

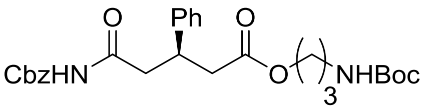

3ai

3-((*tert*-butoxycarbonyl)amino)propyl (*R*)-5-(((benzyloxy)carbonyl)amino)-5-oxo-3-phenylpentanoate (3ac): White solid; **$[\alpha]_D^{23}$** (0.19, $CHCl_3$) = -60.4; **^{1}H NMR (400MHz, $CDCl_3$)** δ= 7.31-7.37 (m, 5H), 7.16-7.27 (m, 5H), 5.13 (s, 2H), 4.62-4.66 (m, 1H), 4.00 (t, *J* = 6.0 Hz, 2H),3.68-3.74 (m, *J* = 7.3 Hz, 1H), 3.05-3.21 (m, 2H), 2.98 (dd, *J* = 13.2, 6.4 Hz, 2H), 2.62-2.78 (m, 2H), 1.62-1.68 (m, 2H), 1.41 (s, 9H). **^{13}C NMR (100 MHz, $CDCl_3$)** δ 172.4, 172.0, 156.1, 151.7, 142.7, 135.0, 128.8, 128.7, 128.5, 127.5, 127.1, 79.4, 68.0, 61.8, 42.2, 40.7, 37.6, 37.1, 29.0, 28.5;. **HRMS(ESI)** calcd for $C_{27}H_{34}N_2NaO_7^+$ $(M+Na)^+$: 521.2258, Found: 521.2263; 99% *ee* as determined by **HPLC** (Chiralcel ODH, 85:15 hexanes/*i*-PrOH, 1.0 mL/min), t_r (major) = 45.6 min, t_r (minor) = 37.7 min.

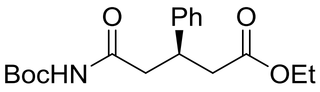

3aj

Ethyl (*R*)-5-((tert-butoxycarbonyl)amino)-5-oxo-3-phenylpentanoate (3ad): Colorless liquid; **[α]$_D$^{23}** (0.03, $CHCl_3$) = +457.3; **^{1}H NMR (400 MHz, $CDCl_3$)** δ 7.18-7.24 (m, 5H), 7.12-7.15 (m, 1H), 3.92-3.98 (m, 2H), 3.64-3.71 (m, 1H), 3.00-3.17 (m, 2H), 2.54-2.72 (m, 2H), 1.40 (s, 9H), 1.07 (t, J = 7.2 Hz, 3H). **^{13}C NMR (100 MHz, $CDCl_3$)** δ 172.7, 171.9, 150.5, 143.0, 128.6, 127.6, 126.9, 82.7, 60.5, 42.1, 40.8, 37.5, 28.1, 14.2, 1.1. **HRMS(ESI)** calcd for $C_{18}H_{25}NNaO_5^+$ $(M+Na)^+$: 358.1625, Found: 358.1620; 72% *ee* as determined by **HPLC** (Chiralcel ASH, 85:15 hexanes/*i*-PrOH, 1.0 mL/min), t_r (major) = 13.7 min, t_r (minor) = 10.5 min.

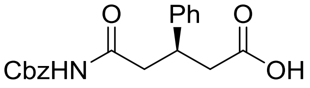

3ak

(*R*)-5-(((benzyloxy)carbonyl)amino)-5-oxo-3-phenylpentanoic acid (3ak): Colorless liquid; **[α]$_D$^{23}** (0.30, $CHCl_3$) = +12.56; **^{1}H NMR (400 MHz, $CDCl_3$)** δ 8.55 (s, 1H), 7.27-7.33 (m, 5H), 7.13-7.23 (m, 5H), 5.06 (s, 2H), 3.69 (s, 1H), 3.08 (s, 2H), 2.54-2.69 (m, 2H). **^{13}C NMR (100 MHz, $CDCl_3$)** δ 173.7, 151.9, 143.6, 135.1, 128.7, 128.7, 128.6, 128.4, 127.4, 126.8, 67.7, 42.6, 41.8, 37.8, 29.8. **HRMS (ESI)** calcd for $C_{19}H_{20}NO_5^+$ $(M+H)^+$: 342.1336, Found: 342.1335.

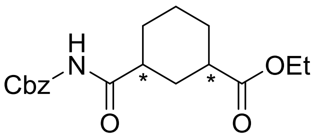

3al

Ethyl 3-(((benzyloxy)carbonyl)carbamoyl)cyclohexane-1-carboxylate (3al): Colorless liquid; **[α]$_D$^{23}** (0.40, $CHCl_3$) = -24.36; **^{1}H NMR (400 MHz, $CDCl_3$)** δ 7.85 (s, 1H), 7.32-7.39 (m, 5H), 5.16 (s, 2H), 4.11 (q, *J* = 7.2 Hz, 2H), 3.05 (s, 1H), 2.30-2.38 (m, 1H), 2.12-2.15 (m, 1H), 1.79-2.03 (m, 4H), 1.60 (q, *J* = 12.8 Hz, 1H), 1.38 (s, 2H), 1.23 (t, *J* = 7.2 Hz, 3H). **^{13}C NMR (100 MHz, $CDCl_3$)** δ 176.3, 175.2, 151.4, 135.0, 128.8, 128.7, 128.5, 128.2, 67.9, 67.0, 60.5, 43.0, 42.6, 30.8, 28.4, 28.3, 24.9, 14.3. **HRMS(ESI)** calcd for $C_{18}H_{24}NO_5^+$ $(M+H)^+$: 334.1649, Found: 334.1645. 46% *ee* as determined by **HPLC** (Chiralcel ODH, 85:15 hexanes/*i*-PrOH, 1.0 mL/min), t_r (major) = 45.6 min, t_r (minor) = 37.7 min.

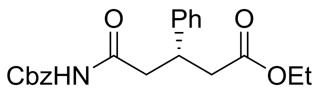

3a'

Ethyl (*S*)-5-(((benzyloxy)carbonyl)amino)-5-oxo-3-phenylpentanoate (3a'): Colorless liquid; **[α]$_D$ ^{23}** (0.25, $CHCl_3$) = +60.3; **HRMS(ESI)** calcd for $C_{21}H_{23}NNaO_5^+$ $(M+Na)^+$: 392.1468, Found: 392.1466; 93% *ee* as determined by **HPLC** (Chiralcel ODH, 90:10 hexanes/*i*-PrOH, 1.0 mL/min), t_r (major) = 47.3 min, t_r (minor) = 34.9 min.

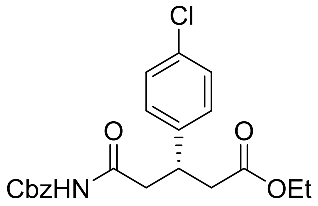

3i'

Ethyl (S)-5-(((benzyloxy)carbonyl)amino)-3-(4-chlorophenyl)-5-oxopentanoate (3i'): Colorless liquid; **[α]$_D$ ^{23}** (0.32, $CHCl_3$) = +47.6; **HRMS(ESI)** calcd for $C_{21}H_{22}NNaO_5^+$ $(M+Na)^+$: 426.1079, Found: 426.1074; 92% *ee* as determined by **HPLC** (Chiralcel ODH, 90:10 hexanes/*i*-PrOH, 1.0 mL/min), t_r (major) = 41.4 min, t_r (minor) = 38.2 min.

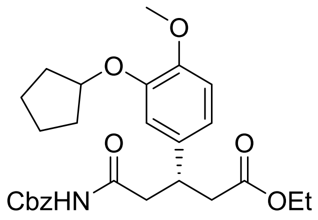

3j'

Ethyl (*S*)-5-(((benzyloxy)carbonyl)amino)-3-(3-(cyclopentyloxy)-4-methoxyphenyl)-5-oxopentanoate (3j'): Colorless liquid; **[α]$_D$ ^{23}** (0.31, $CHCl_3$) = +81.4; **HRMS(ESI)** calcd for $C_{27}H_{33}NNaO_7^+$ $(M+Na)^+$: 506.2149, Found: 506.2144; 91% *ee* as determined by **HPLC** (Chiralcel ODH, 90:10 hexanes/*i*-PrOH, 1.0 mL/min), t_r (major) = 41.8 min, t_r (minor) = 37.3 min.

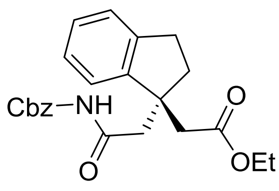

5a

Ethyl (*R*)-2-(1-(2-(((benzyloxy)carbonyl)amino)-2-oxoethyl)-2,3-dihydro-1H-inden-1-yl)acetate (5a): Colorless liquid; **[α]$_D$ ^{23}** (0.5, $CHCl_3$) = -32.03; **^{1}H NMR**

(400 MHz, $CDCl_3$) δ 8.12 (s, 1H), 7.31-7.40 (m, 5H), 7.08-7.24 (m, 4H), 5.13 (s, 2H), 4.07 (q, *J* = 7.2 Hz, *J* = 14.4 Hz, 2H), 3.40 (d, *J* = 15.6 Hz, 1H), 2.85-2.99 (m, 4H), 2.75 (d, *J* = 14.8 Hz, 1H), 2.23 (t, *J* = 7.6 Hz, 2H), 1.16 (t, *J* = 7.2 Hz, 3H). **^{13}C NMR (100 MHz, $CDCl_3$)** δ 172.5, 171.5, 151.2, 147.1, 143.0, 135.2, 128.7, 128.7, 128.5, 127.6, 126.4, 125.0, 123.6, 67.7, 60.6, 48.2, 43.9, 42.1, 38.8, 30.1, 14.2. **HRMS(ESI)** calcd for $C_{23}H_{26}NO_5^+$ (M+H)$^+$: 396.1805, Found: 396.1807. 93% *ee* as determined by **HPLC** (Chiralcel ASH, 95:5 hexanes/*i*-PrOH, 1.0 mL/min), t_r (major) = 62.4 min, t_r (minor) = 71.7 min.

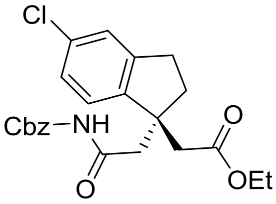

5b

Ethyl (*R*)-2-(1-(2-(((benzyloxy)carbonyl)amino)-2-oxoethyl)-5-chloro-2,3-dihydro-1H-inden-1-yl)acetate (5b): Colorless liquid; **[α]$_D$^{23}** (0.5, $CHCl_3$) = -42.56; **^{1}H NMR (400 MHz, $CDCl_3$)** δ 7.89 (s, 1H),7.32-7.40(m, 5H), 7.18 (s, 1H), 7.09 (d, *J* = 4 Hz 2H), 5.13 (s, 2H), 4.03-4.09 (m, 2H), 3.39 (d, *J* = 16.0 Hz, 1H), 2.87-2.98 (m, 4H), 2.75 (d, *J* = 16.0 Hz, 1H), 2.22-2.26 (m, 2H), 1.17 (t, *J* = 8.0 Hz 3H). **^{13}C NMR (100 MHz, $CDCl_3$)** δ 172.1, 151.5, 145.7, 145.2, 135.1, 133.2, 128.8, 128.8, 128.7, 128.5, 128.4, 126.6, 125.1, 124.9, 67.8, 66.4, 60.7, 47.7, 43.5, 42.0, 38.8, 30.0, 29.8, 14.2. **HRMS(ESI)** calcd for $C_{23}H_{25}ClNO_5^+$ (M+H)$^+$: 430.1416, Found: 430.1418. 87% *ee* as determined by **HPLC** (Chiralcel AZH, 95:5 hexanes/*i*-PrOH, 1.0 mL/min), t_r (major) = 56.3 min, t_r (minor) = 71.8 min.

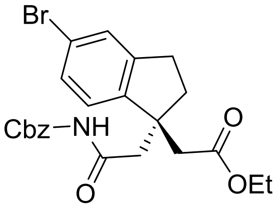

5c

Ethyl (*R*)-2-(1-(2-(((benzyloxy)carbonyl)amino)-2-oxoethyl)-5-bromo-2,3-dihydro-1H-inden-1-yl)acetate (5c): Colorless liquid; **[α]$_D$ ^{23}** (0.50, $CHCl_3$) = -47.48; **^{1}H NMR (400 MHz, $CDCl_3$)** δ 7.86 (s, 1H), 7.31-7.43 (m, 6H), 7.21-7.25 (m, 1H), 7.05 (d, *J* = 8.0 Hz, 1H), 5.13 (s, 2H), 4.03-4.12 (m, 2H), 3.40 (d, *J* = 16.0 Hz, 1H), 2.84-3.03 (m, 4H), 2.74 (d, *J* = 14.8 Hz, 1H), 2.23 (t, *J* = 8.0 Hz, 2H), 1.17 (t, *J* = 6.8 Hz, 3H). **^{13}C NMR (100 MHz, $CDCl_3$)** δ 1721, 171.9, 151.4, 146.4, 145.6, 135.1, 129.4, 128.8, 128.8, 128.5, 128.1, 125.4, 121.3, 67.8, 60.6, 47.8, 43.4, 41.9, 38.7, 30.0, 29.8, 14.2. **HRMS(ESI)** calcd for $C_{23}H_{25}BrNO_5^+$ (M+H)$^+$: 474.0911, Found: 474.0915. 89% *ee* as determined by **HPLC** (Chiralcel IA, 95:5 hexanes/*i*-PrOH, 1.0 mL/min), t_r (major) = 35.5 min, t_r (minor) = 42.9 min.

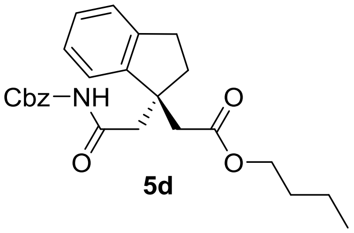

5d

Butyl (*R*)-2-(1-(2-(((benzyloxy)carbonyl)amino)-2-oxoethyl)-2,3-dihydro-1H-inden-1-yl)acetate (5d): Colorless liquid; **$[\alpha]_D$ ^{23}** (0.40, $CHCl_3$) = +10.27. **^{1}H NMR (400 MHz, $CDCl_3$)** δ 8.05 (s, 1H), 7.32-7.40 (m, 5H), 7.12-7.22 (m, 4H), 5.13 (s, 2H), 4.01 (t, J = 6.0 Hz, 2H), 3.39 (d, J = 15.6 Hz, 1H), 2.89-2.95 (m, 4H), 2.76 (d, J = 14.8 Hz, 1H), 2.23 (t, J = 7.2 Hz, 2H), 1.48-1.55 (m, 2H), 1.23-1.34 (m, 2H), 0.88 (t, J = 7.6 Hz, 3H). **^{13}C NMR (100 MHz, $CDCl_3$)** δ 172.6, 171.5, 151.2, 147.2, 143.0, 135.2, 128.7, 128.7, 128.5, 127.6, 126.5, 125.0, 123.6, 67.7, 64.5, 48.2, 43.9, 42.1, 38.8, 30.6, 30.1, 29.8, 19.2, 13.8. **HRMS(ESI)** calcd for $C_{25}H_{30}NO_5^+$ $(M+H)^+$: 424.2118, Found: 424.2115. 89% *ee* as determined by **HPLC** (Chiralcel IA, 95:5 hexanes/*i*-PrOH, 1.0 mL/min), t_r (major) = 24.1 min, t_r (minor) = 26.1 min.

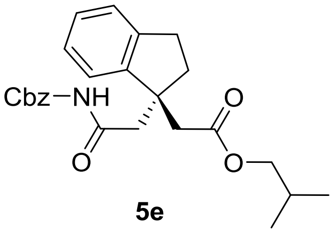

5e

Isobutyl (*R*)-2-(1-(2-(((benzyloxy)carbonyl)amino)-2-oxoethyl)-2,3-dihydro-1H-inden-1-yl)acetate (5e): Colorless liquid; **$[\alpha]_D$ ^{23}** (0.50, $CHCl_3$) = +29.34; **^{1}H NMR (400 MHz, $CDCl_3$)** δ 8.08 (s, 1H),7.31-7.39(m, 5H), 7.10-7.20 (m, 1H), 5.13 (s, 2H),3.76-3.84 (m, 2H), 3.38 (d, J = 16.0 Hz 1H), 2.93 (m, 4H), 2.77 (d, J = 16.0 Hz, 1H), 2.23 (t, J = 8.0 Hz, 2H), 1.77-1.97 (m, 1H), 0.84 (d, J = 12 Hz 6H). **^{13}C NMR (100 MHz, $CDCl_3$)** δ 172.6, 151.7, 147.2, 143.0, 135.2, 128.7, 128.7, 128.5, 127.6, 126.5, 125.0, 123.6, 70.9, 67.7, 48.2, 43.9, 42.1, 38.8, 30.1, 29.8, 27.6, 19.2. **HRMS(ESI)** calcd for $C_{23}H_{25}ClNO_5^+$ $(M+H)^+$: 424.2118, Found: 424.2263. 90% *ee* as determined by **HPLC** (Chiralcel IA, 98:2 hexanes/*i*-PrOH, 1.0 mL/min), t_r (major) = 57.3 min, t_r (minor) = 61.9 min.

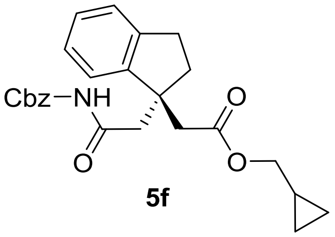

5f

Cyclopropylmethyl (*R*)-2-(1-(2-(((benzyloxy)carbonyl)amino)-2-oxoethyl)-2,3-dihydro-1H-inden-1-yl)acetate (5f): Colorless liquid; **$[\alpha]_D^{23}$** (0.4, $CHCl_3$) = +34.34; **^{1}H NMR (400 MHz, $CDCl_3$)** δ 8.11 (s, 1H), 7.30-7.38 (m, 5H), 7.09-7.2 (m, 4H), 5.13 (s, 2H), 3.76-3.90 (m, 2H), 3.39 (d, *J* = 16.0 Hz, 1H), 2.71-2.99 (m, 5H), 2.24 (t, *J* = 7.2 Hz, 2H), 0.98-1.11 (m, 1H), 0.45-0.55 (m, 2H), 0.16-0.26 (m, 2H). **^{13}C NMR (100 MHz, $CDCl_3$)** δ 172.6, 151.2, 147.1, 143.0, 135.2, 128.7, 128.7, 128.5, 127.6, 126.4, 125.0, 123.6, 69.5, 67.7, 48.3, 44.0, 42.1, 38.8, 30.1, 9.8, 3.4. **HRMS(ESI)** calcd for $C_{25}H_{28}NO_5^+$ $(M+H)^+$: 422.1962, Found: 422.1967. 94% *ee* as determined by **HPLC** (Chiralcel ASH, 95:5 hexanes/*i*-PrOH, 1.0 mL/min), t_r (major) = 84.5 min, t_r (minor) = 96.4 min.

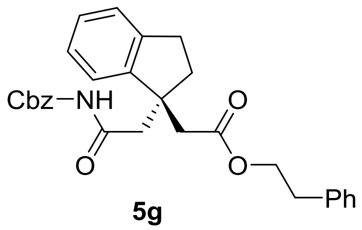

5g

Phenethyl (*R*)-2-(1-(2-(((benzyloxy)carbonyl)amino)-2-oxoethyl)-2,3-dihydro-1H-inden-1-yl)acetate (5g): Colorless liquid; **$[\alpha]_D^{23}$** (0.20, $CHCl_3$) = +41.41; **^{1}H NMR (400 MHz, $CDCl_3$)** δ 7.99 (s, 1H), 7.34-7.39 (m, 5H), 7.27-7.32 (m, 2H), 7.10-7.24 (m, 7H), 5.13 (s, 2H), 4.22-4.27 (m, 2H), 3.36 (d, *J* = 16.0 Hz, 1H), 2.89-2.96 (m, 4H), 2.75 (t, *J* = 7.2 Hz, 2H), 2.76 (d, *J* = 14.8 Hz, 1H), 2.19 (t, *J* = 7.2 Hz, 2H). **^{13}C NMR (100 MHz, $CDCl_3$)** δ 172.3, 171.6, 151.2, 147.2, 143.0, 137.8, 135.2, 129.0, 128.8, 128.7, 128.6, 128.5, 128.4, 127.6, 126.7, 126.5, 125.0, 123.6, 67.7, 66.4, 65.0, 48.3, 48.2, 43.7, 42.1, 38.7, 35.0, 30.1. **HRMS(ESI)** calcd for $C_{29}H_{30}NO_5^+$ $(M+H)^+$: 472.2118, Found: 472.2115. 84% *ee* as determined by **HPLC** (Chiralcel IA, 98:2 hexanes/*i*-PrOH, 1.0 mL/min), t_r (major) = 122.8 min, t_r (minor) = 131.0min.

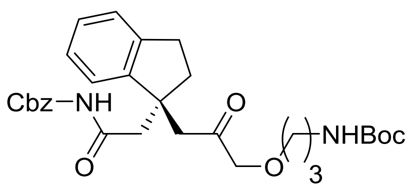

5h

Benzyl (*S*)-(2-(1-(3-(((tert-butoxycarbonyl)amino)methoxy)-2-oxopropyl)-2,3-dihydro-1H-inden-1-yl)acetyl)carbamate (5h): Colorless liquid; **$[\alpha]_D^{23}$** (0.18, $CHCl_3$) = +43.43; **^{1}H NMR (400 MHz, $CDCl_3$)** δ 8.11 (s, 1H), 7.32-7.39 (m, 5H), 7.13-7.22 (m, 4H), 5.13 (s, 2H), 4.63 (s, 1H), 4.04-4.07 (m, 2H), 3.40 (d, *J* = 16 Hz, 1H), 3.05 (s, 2H), 2.87-2.95 (m, 4H), 2.78 (d, *J* = 14.8 Hz, 1H), 2.19-2.31 (m, 2H), 1.70-1.74 (m, 2H), 1.42 (s, 9H). **^{13}C NMR (100 MHz, $CDCl_3$)** δ 172.3, 171.4, 156.1, 151.2, 147.2, 143.1, 135.2, 128.7, 128.7, 128.5, 127.6, 126.4, 125.0, 123.5, 79.4, 67.7, 61.9, 48.2, 43.8, 42.2, 38.8, 37.3, 30.1, 29.8, 29.0, 28.5. **HRMS(ESI)** calcd for

$C_{29}H_{37}N_2O_7^+$ $(M+H)^+$: 525.2595, Found: 525.2598. 89% *ee* as determined by **HPLC** (Chiralcel IA, 95:5 hexanes/*i*-PrOH, 1.0 mL/min), t_r (major) = 80.8 min, t_r (minor) = 92.9min.

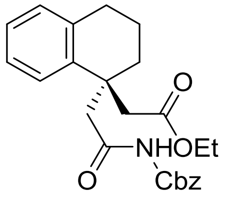

5i

Ethyl 2-(1-(2-(((benzyloxy)carbonyl)amino)-2-oxoethyl)-1,2,3,4-tetrahydronaphthalen-1-yl)acetate (S6): Colorless liquid; $[\alpha]_D^{23}$ (0.5, $CHCl_3$) = +19.19; **^{1}H NMR (400 MHz, $CDCl_3$)** δ 8.18 (s, 1H), 7.35-7.37 (m, 5H) 7.14-7.17 (m, 1H), 7.03-7.10 (m, 3H), 5.13 (s, 2H), 4.07 (q, *J* = 4 Hz, *J* = 12 Hz, 2H), 3.30(d, *J* = 12 Hz 1H), 2.75-2.86 (m, 4H), 1.98-2.11 (m, 2H), 1.78-1.87 (m, 2H), 1.66 (s, 1H), 1.15 (t, *J* = 8 Hz 3H). **^{13}C NMR (100 MHz, $CDCl_3$)** δ 172.9, 170.82, 151.2, 144.1, 135.3, 128.7, 128.6, 128.5, 128.4, 126.5, 125.9, 67.6, 60.6, 43.1, 42.9, 42.8, 42.0, 16.9, 14.4, 14.0. **HRMS** (ESI) calcd for $C_{24}H_{25}ClNO_5^+$ $(M+H)^+$: 410.1962, Found: 410.1979. 60% *ee* as determined by HPLC (Chiralcel ODH, 90:10 hexanes/*i*-PrOH, 1.0 mL/min), t_r (major) = 64.8 min, t_r (minor) = 89.7 min.

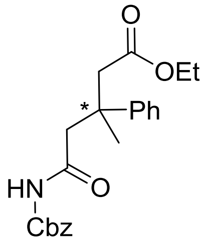

5j

Ethyl 5-(((benzyloxy)carbonyl)amino)-3-methyl-5-oxo-3-phenylpentanoate (S8): Colorless liquid; $[\alpha]_D^{23}$ (0.30, $CHCl_3$) = +26.63; **^{1}H NMR (400 MHz, $CDCl_3$)** δ 8.04 (s, 1H), 7.28-7.38 (m, 9H), 7.17-7.22 (m, 1H), 5.14 (s, 2H), 3.96-4.02 (m, 2H), 3.14-3.28 (m, 2H), 2.87 (q, *J* = 13.2 Hz, 2H), 1.58 (s, 3H), 1.07 (t, *J* = 11.2 Hz, 3H). **^{13}C NMR (100 MHz, $CDCl_3$)** δ 172.2, 171.0, 151.2, 145.7, 135.2, 128.7, 128.7, 128.5, 128.3, 126.6, 125.5, 67.7, 60.5, 46.2, 45.1, 39.6, 26.9, 14.1. **HRMS** (ESI) calcd for $C_{22}H_{26}NO_5^+$ $(M+H)^+$: 384.1805, Found: 384.1801. 42 % *ee* as determined by **HPLC** (Chiralcel ASH, 90:10 hexanes/*i*-PrOH, 1.0 mL/min), t_r (major) = 27.2 min, t_r (minor) = 50.3min.

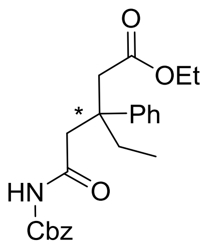

5k

Ethyl 5-(((benzyloxy)carbonyl)amino)-3-ethyl-5-oxo-3-phenylpentanoate (S9): Colorless liquid; **[α]$_D$^{23}** (0.30, $CHCl_3$) = -73.74; **^{1}H NMR (400 MHz, $CDCl_3$)** *δ* 8.45 (s, 1H), 7.28-7.40 (m, 7H), 7.17-7.27 (m, 3H), 5.17 (q, *J* = 12.0 Hz, *J* = 13.6 Hz, 2H), 3.93-4.04 (m, 2H), 2.82-3.37(m, 4H), 1.80-2.00 (m, 2H), 1.04 (t, *J* = 7.2 Hz, 3H), 0.67 (t, *J* = 7.2 Hz, 3H). **^{13}C NMR (100 MHz, $CDCl_3$)** *δ* 172.9, 170.9, 151.2, 143.7, 135.3, 128.7, 128.6, 128.5, 128.4, 127.1, 126.5, 126.0, 67.6, 60.6, 43.3, 42.5, 41.6, 33.0, 14.4, 14.0, 8.1. **HRMS(ESI)** calcd for $C_{23}H_{28}NO_5^+$ $(M+H)^+$: 398.1962, Found: 398.1963. 20% *ee* as determined by **HPLC** (Chiralcel AZH, 95:15 hexanes/*i*-PrOH, 1.0 mL/min), t_r (major) = 66.1 min, t_r (minor) = 75.0min.

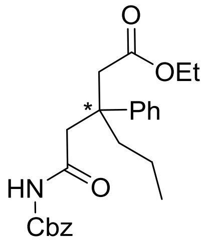

5l

Ethyl 3-(2-(((benzyloxy)carbonyl)amino)-2-oxoethyl)-3-phenylhexanoate (S10): Colorless liquid; **[α]$_D$^{23}** (0.50, $CHCl_3$) = -82.63;**^{1}H NMR (400 MHz, $CDCl_3$)** *δ* 8.46 (s, 1H), 7.28-7.38 (m, 7H), 7.17-7.26 (m, 3H), 5.17 (s, 2H), 3.93-4.03 (m, 2H), 2.80-3.45(m, 4H), 1.56-1.99 (m, 3H), 1.07-1.21 (m, 1H), 1.04 (t, *J* = 7.2 Hz, 3H), 0.79 (t, *J* = 7.2 Hz, 3H). **^{13}C NMR (100 MHz, $CDCl_3$)** *δ* 172.8, 171.0, 151.3, 144.2, 135.3, 128.7, 128.6, 128.5, 128.4, 126.5, 125.9, 67.5, 60.6, 60.5, 43.0, 42.8, 42.8, 41.9, 16.9, 14.4, 14.3, 14.0. **HRMS(ESI)** calcd for $C_{24}H_{30}NO_5^+$ $(M+H)^+$: 412.2118, Found: 412.2117. 9 % *ee* as determined by HPLC (Chiralcel IA, 95:15 hexanes/i-PrOH, 1.0 mL/min), tr (major) = 80.4 min, tr (minor) = 87.4min.

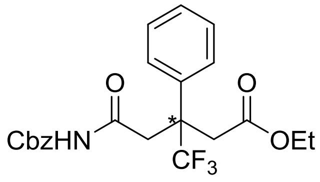

5m

Ethyl 5-(((benzyloxy)carbonyl)amino)-5-oxo-3-phenyl-3-(trifluoromethyl)pentanoate (S2): Colorless liquid; **[α]$_D$^{23}** (0.10, $CHCl_3$) = +144.2; **^{1}H NMR (400 MHz, $CDCl_3$)** *δ* 7.96 (s, 1H), 7.32-7.41 (m, 10H), 5.17-5.18 (m, Hz, 2H), 4.08-4.14 (m, 2H), 3.78-4.05 (m, 2H), 3.41-3.51 (m, 2H), 1.19-1.23 (m, 3H). **^{13}C NMR (100 MHz, $CDCl_3$)** *δ* 170.6, 170.2, 151.7, 136.5, 135.0, 128.9, 128.8, 128.7, 128.6, 128.4, 127.0, 68.1, 61.1, 48.5, 48.2, 36.2, 35.1, 14.1; **HRMS(ESI)** calcd for $C_{22}H_{22}F_3NNaO_5^+$ $(M+Na)^+$: 460.1342, Found: 460.1348; 64% *ee* as determined by **HPLC** (Chiralcel ASH, 90:10 hexanes/*i*-PrOH, 1.0 mL/min), t_r (major) = 22.5 min, t_r (minor) = 18.4 min.

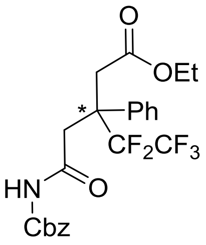

5n

Ethyl 3-(2-(((benzyloxy)carbonyl)amino)-2-oxoethyl)-4,4,5,5,5-pentafluoro-3-phenylpentanoate (S7): Colorless liquid; **[α]$_D$** ^{23} (0.40, $CHCl_3$) = +22.20; ^{1}**H NMR (400 MHz, $CDCl_3$)** δ 7.71 (s, 1H), 7.29-7.42 (m, 10H), 5.20 (q, J = 12.4 Hz, J = 14.8 Hz, 2H), 4.12-4.20 (m, 3H), 3.90 (d, J = 9.2 Hz, 1H), 3.58 (s, 2H), 1.23 (t, 3H), 2.24 (t, J = 7.2 Hz, 2H), 0.98-1.11 (m, 1H), 0.45-0.55 (m, 2H), 0.16-0.26 (m, J = 6.8 Hz, 2H). ^{13}**C NMR (100 MHz, $CDCl_3$)** δ 170.6, 170.0, 151.5, 136.0, 134.9, 128.9, 128.8, 128.6, 128.4, 127.2, 68.1, 61.0, 48.2, 48.0, 47.8, 35.9, 34.9, 29.8, 14.0. ^{19}**F NMR (376 MHz, $CDCl_3$)** δ -77.0, -115.4; **HRMS(ESI)** calcd for $C_{23}H_{23}F_5NO_5^+$ $(M+H)^+$: 488.1491, Found: 488.1493. 43% *ee* as determined by **HPLC** (Chiralcel ODH, 95:15 hexanes/*i*-PrOH, 1.0 mL/min), t_r (major) = 64.8 min, t_r (minor) = 89.7 min.

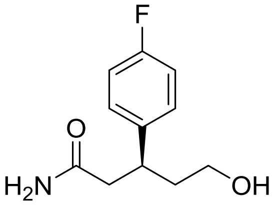

6

(*S*)-3-(4-fluorophenyl)-5-hydroxypentanamide (6): white solid; **[α]$_D$** ^{23} (0.13, $CHCl_3$) = +153.4; ^{1}**H NMR (400 MHz, $CDCl_3$)** δ 7.11-7.14 (m, 2H), 6.91-6.96 (m, 2H), 5.29 (s, 2H), 3.41-3.54 (m, 2H), 3.24-3.31 (m, 1H), 2.39-2.55 (m, 2H), 1.76-1.92 (m, 3H). ^{13}**C NMR (100 MHz, $CDCl_3$)** δ 173.9, 129.0, 128.9, 115.7, 115.5, 60.4, 43.14, 39.1, 38.0. **HRMS(ESI)** calcd for $C_{11}H_{14}FNNaO_2^+$ $(M+Na)^+$: 234.0901, Found: 234.0901; 91% *ee* as determined by **HPLC** (Chiralcel IC, 85:15 hexanes/*i*-PrOH, 1.0 mL/min), t_r (major) = 42.0 min, t_r (minor) = 36.8 min.

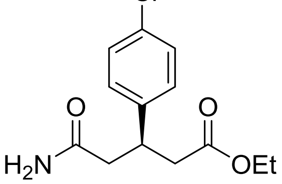

7'

Ethyl (*R*)-5-amino-3-(4-chlorophenyl)-5-oxopentanoate (7'): white solid; **[α]$_D$** ^{23} (0.47, EA) = -21.2; ^{1}**H NMR (400 MHz, $CDCl_3$)** δ 7.20-7.30 (m, 3H), 7.16 (d, J = 8.0

Hz, 1H), 5.53 (d, J = 37.2 Hz, 2H), 3.98-4.04 (m, 2H), 3.57-3.65 (m, 1H), 2.70-2.77 (m, 1H), 2.58-2.66 (m, 2H), 2.43-2.54 (m, 1H), 1.10-1.14 (m, 3H). **^{13}C NMR (100 MHz, CDCl$_3$)** δ 171.7, 141.2, 132.8, 128.9, 128.8, 127.4, 60.7, 42.1, 40.4, 38.2, 14.2. **HRMS(ESI)** calcd for $C_{13}H_{16}ClNNaO_3^+$ $(M+Na)^+$: 292.0711, Found: 292.0718; 93% *ee* as determined by **HPLC** (Chiralcel OJH, 85:15 hexanes/*i*-PrOH, 1.0 mL/min), t_r (major) = 15.2 min, t_r (minor) = 29.1 min.

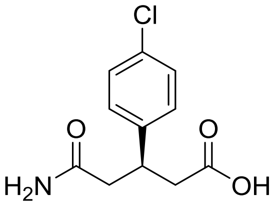

7

(*R*)-5-amino-3-(4-chlorophenyl)-5-oxopentanoic acid (7)[3]: white solid; $[\alpha]_D^{23}$ (0.05, MeOH) = +302.4; **HRMS(ESI)** calcd for $C_{11}H_{12}ClNNaO_3^+$ $(M+Na)^+$: 264.0398, Found: 264.0393;

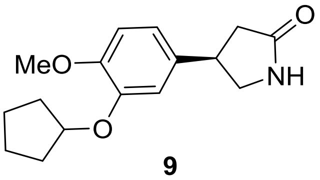

9

(*R*)-4-(3-(cyclopentyloxy)-4-methoxyphenyl)pyrrolidin-2-one (9)[2]: white solid; **$[\alpha]_D^{23}$** (0.05, CHCl$_3$) = -100.8; **HRMS(ESI)** calcd for $C_{16}H_{21}NNaO_3^+$ $(M+Na)^+$: 298.1414, Found: 298.1410; 95% *ee* as determined by **HPLC** (Chiralcel ID, 90:10 hexanes/*i*-PrOH, 1.0 mL/min), t_r (major) = 28.3 min, t_r (minor) = 24.3 min.

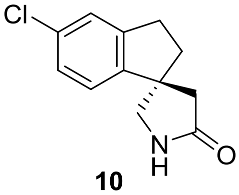

10

Methyl (R)-5-(((benzyloxy)carbonyl)amino)-5-oxo-3-phenylpentanoate (3t): white solid; **$[\alpha]_D^{23}$** (0.32, CHCl$_3$) = -49.500; **^{1}H NMR (400 MHz, CDCl$_3$)** δ7.16-7.26 (m, 3H), 6.12 (s, 1H), 3.47 (s, 2H), 2.93 (t, J = 8.4 Hz, 2H), 2.54 (dd, J = 48.0 Hz, J = 31.2 Hz, 2H), 2.16-2.31 (m, 2H). **^{13}C NMR (100 MHz, CDCl$_3$)** δ 177.1, 145.6, 145.2, 133.4, 127.3, 125.2, 123.2, 54.2, 50.9, 43.6, 40.1, 30.1.**HRMS(ESI)** calcd for Chemical Formula: $C_{12}H_{13}ClNO^+$ $(M+H)^+$: 222.0680, Found: 222.0682. 99% *ee* as determined by **HPLC** (Chiralcel IA, 90:10 hexanes/*i*-PrOH, 1.0 mL/min), t_r (major) = 11.5 min, t_r (minor) = 96.4 min.

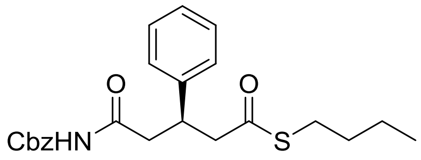

S4

n-butyl (*R*)-5-(((benzyloxy)carbonyl)amino)-5-oxo-3-phenylpentanethioate (S4): Colorless liquid; **[α]$_D$^{23}** (0.5, $CHCl_3$) = +69.46; **^{1}H NMR (400 MHz, $CDCl_3$)** δ 7.90 (s, 1H), 7.32-7.37 (m, 5H), 7.16-7.27` (m, 5H), 5.13 (s, 2H), 3.75-3.83 (m, 1H), 3.05-3.23 (m, 2H), 2.85-2.98 (m, 2H), 2.78 (t, *J* = 7.6, 2H), 1.40-1.47 (m, 2H), 1.23-1.32 (m, 2H), 0.85 (t, *J* = 7.6, 3H). **^{13}C NMR (100 MHz, $CDCl_3$)** δ 197.9, 172.5, 151.7, 142.4, 135.0, 128.8, 128.6, 128.5, 127.6, 127.0, 67.9, 49.7, 41.9, 38.1, 31.5, 28.7, 21.9, 13.7. **HRMS(ESI)** calcd for $C_{26}H_{25}NNaO_4S^+$ $(M+Na)^+$: 470.1397, Found: 470.1395; 8% *ee* as determined by **HPLC** (Chiralcel AZH, 90:10 hexanes/*i*-PrOH, 1.0 mL/min), t_r (major) = 24.7 min, t_r (minor) = 28.4 min.

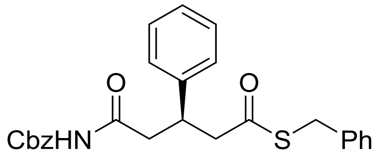

S5

S-benzyl (*R*)-5-(((benzyloxy)carbonyl)amino)-5-oxo-3-phenylpentanethioate (S5): Colorless liquid; **[α]$_D$^{23}** (0.5, $CHCl_3$) = +59.64; **^{1}H NMR (400 MHz, $CDCl_3$)** δ 7.78 (s, 1H), 7.33-7.40 (m, 5H), 7.14-7.29 (m, 10H), 5.14 (s, 2H), 4.03 (s, 2H), 3.78-3.86 (m, 1H), 3.07-3.25 (m, 2H), 2.88-3.02 (m, 2H). **^{13}C NMR (100 MHz, $CDCl_3$)** δ 197.0, 172.5, 151.7, 142.2, 137.5, 135.0, 128.9, 128.8, 128.7, 128.7, 128.5, 128.3, 127.6, 127.3, 127.1, 68.0, 49.4, 41.9, 38.1, 33.3. **HRMS(ESI)** calcd for $C_{26}H_{25}NNaO_4S^+$ $(M+Na)^+$: 470.1397, Found: 470.1395; 0% *ee* as determined by **HPLC** (Chiralcel IA, 90:10 hexanes/*i*-PrOH, 1.0 mL/min), t_r (major) = 64.3 min, t_r (minor) = 71.6 min.

4. ^{1}H, ^{13}C NMR and HPLC date

^{1}H and ^{13}C NMR spectra of substrates and products

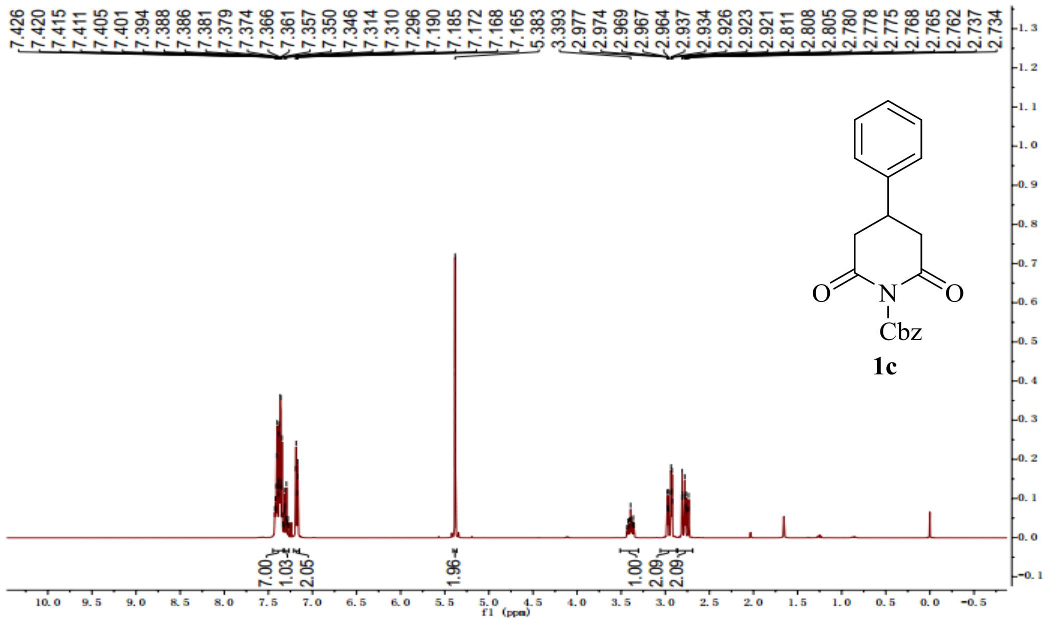

Supplementary Fig. 3. ^{1}H NMR Spectrum of **1c**

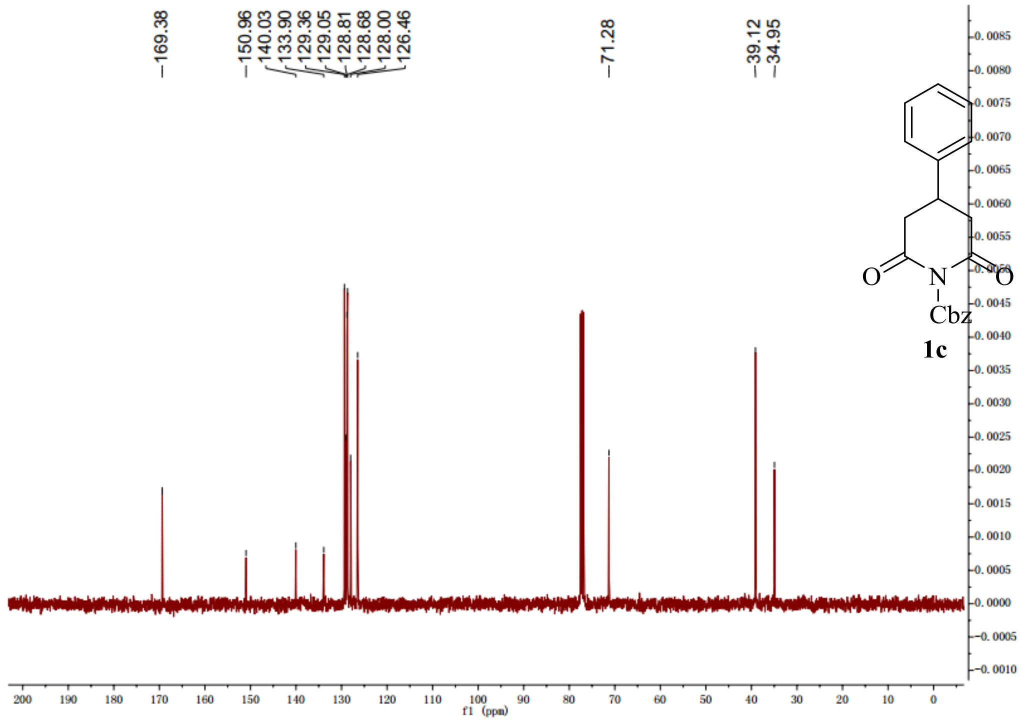

Supplementary Fig. 4. ^{13}C NMR Spectrum of **1c**

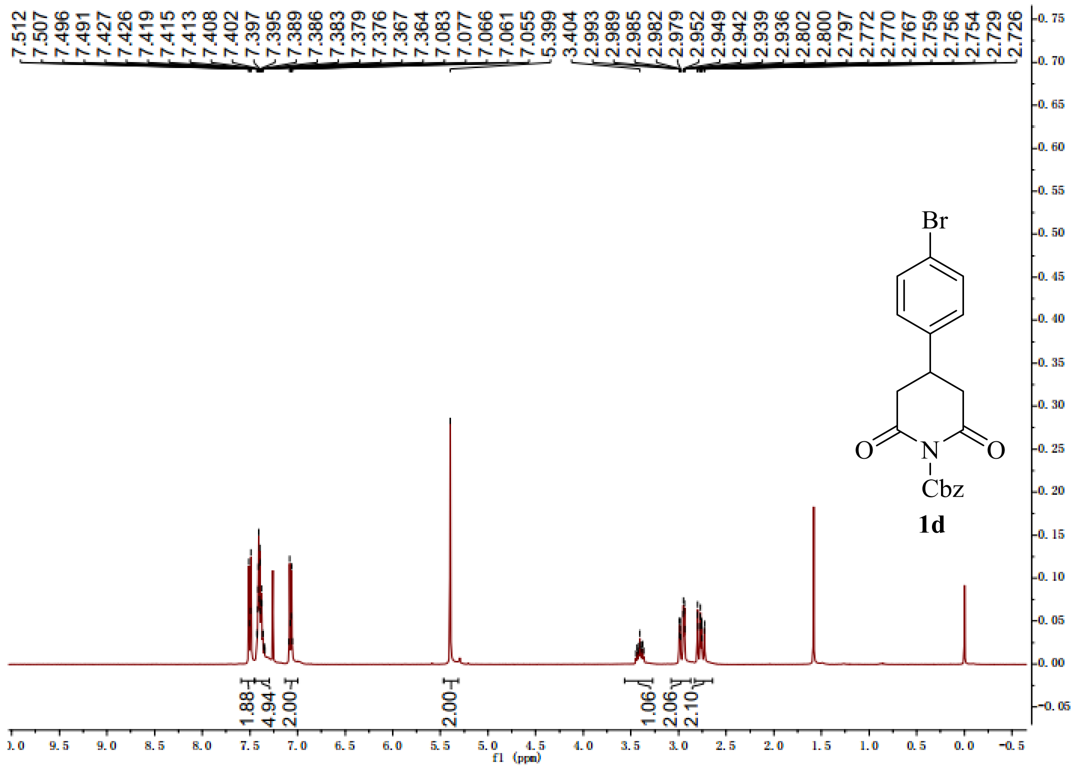

Supplementary Fig. 5. ^{1}H NMR Spectrum of **1d**

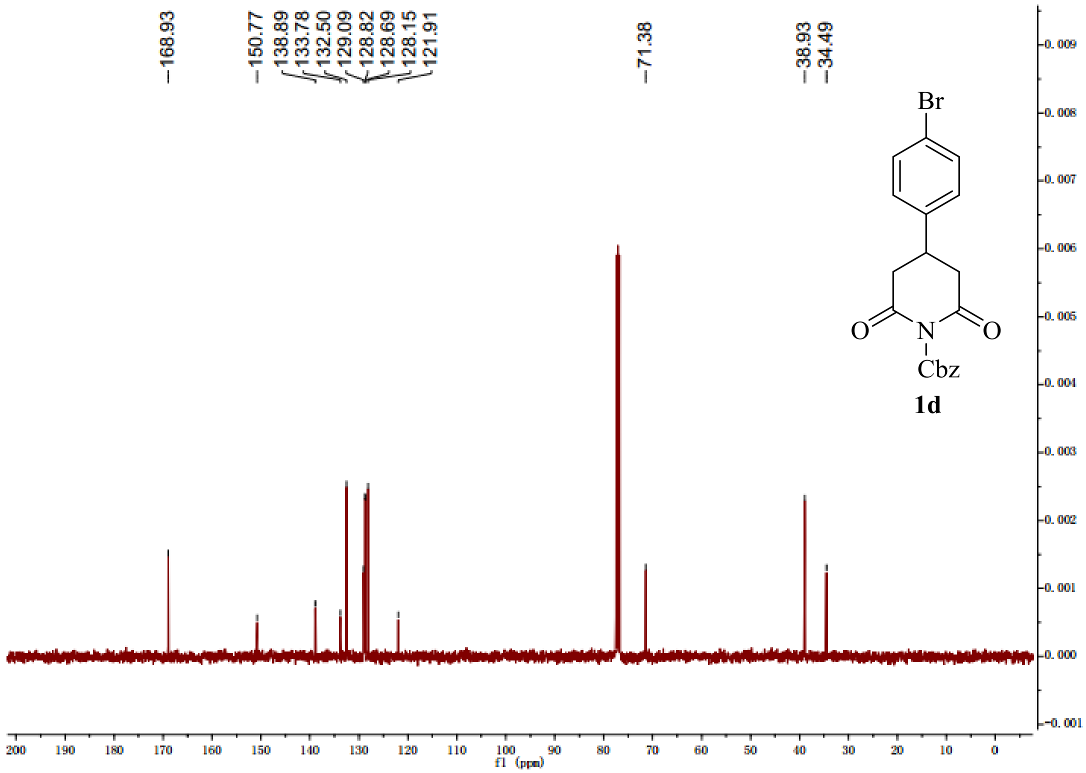

Supplementary Fig. 6. ^{13}C NMR Spectrum of **1d**

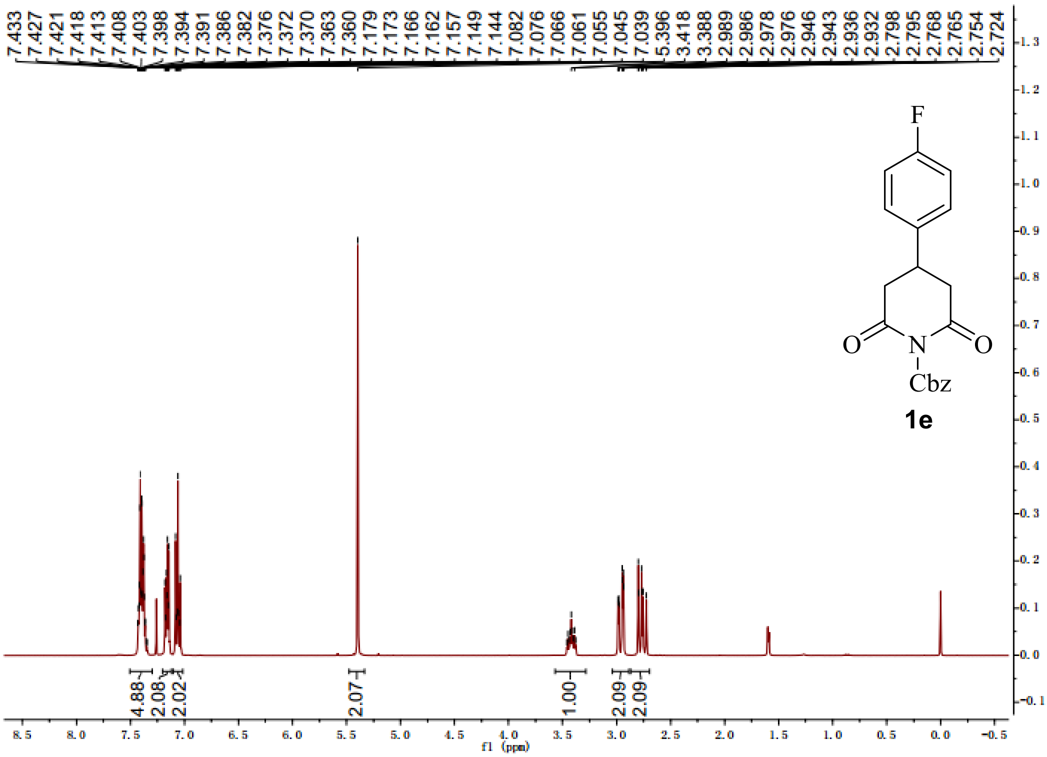

Supplementary Fig. 7. ^{1}H NMR Spectrum of **1e**

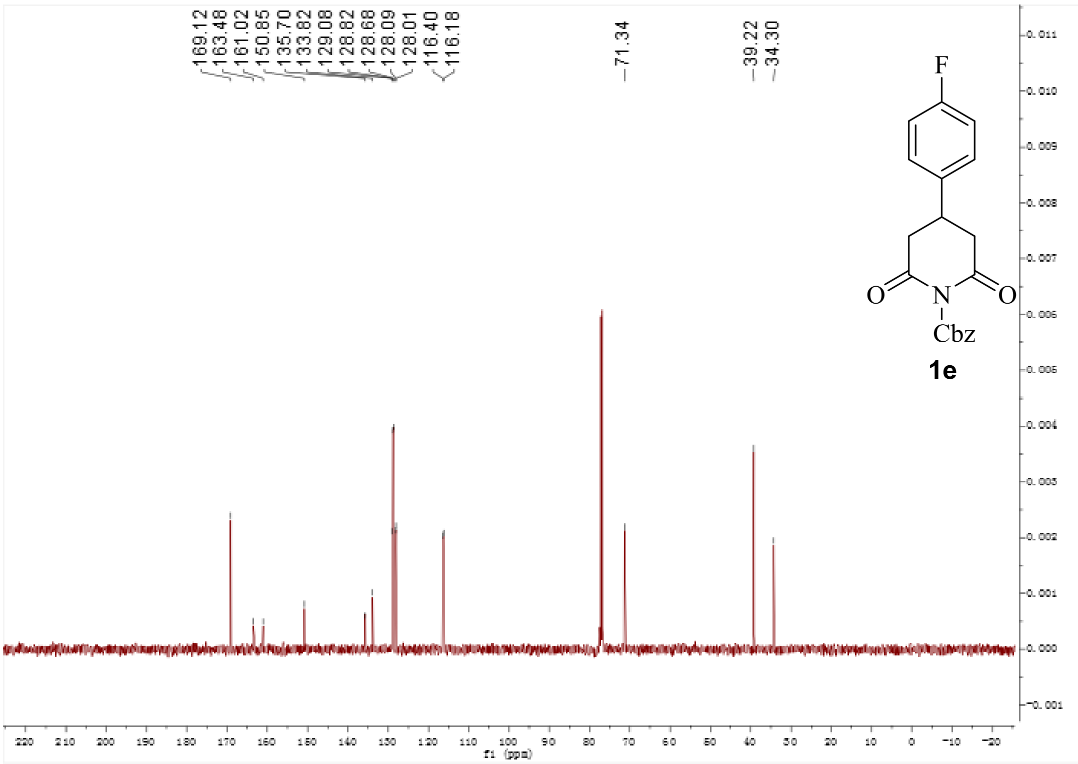

Supplementary Fig. 8. ^{13}C NMR Spectrum of **1e**

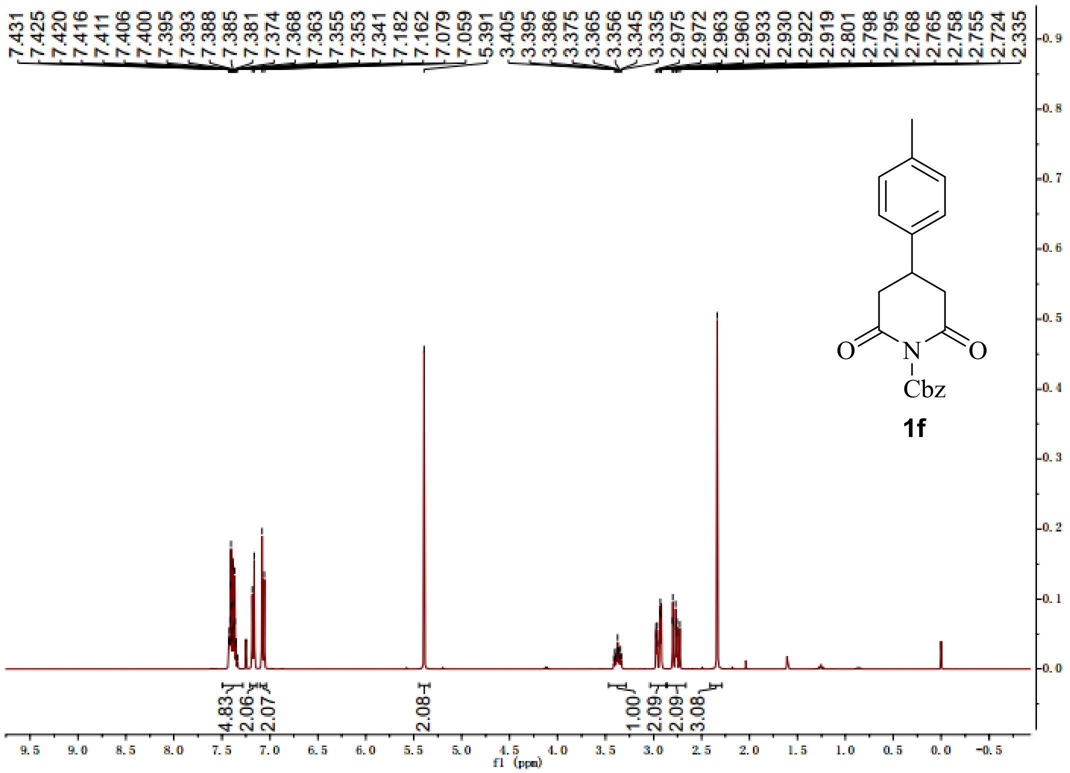

Supplementary Fig. 9. ^{1}H NMR Spectrum of **1f**

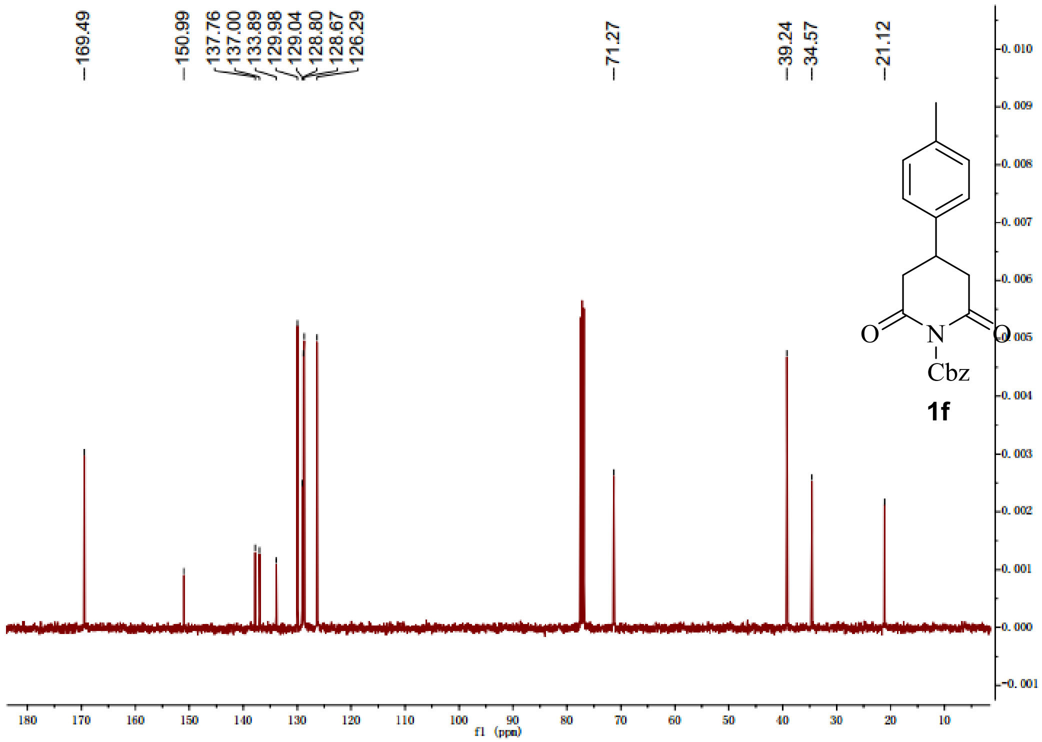

Supplementary Fig. 10. ^{13}C NMR Spectrum of **1f**

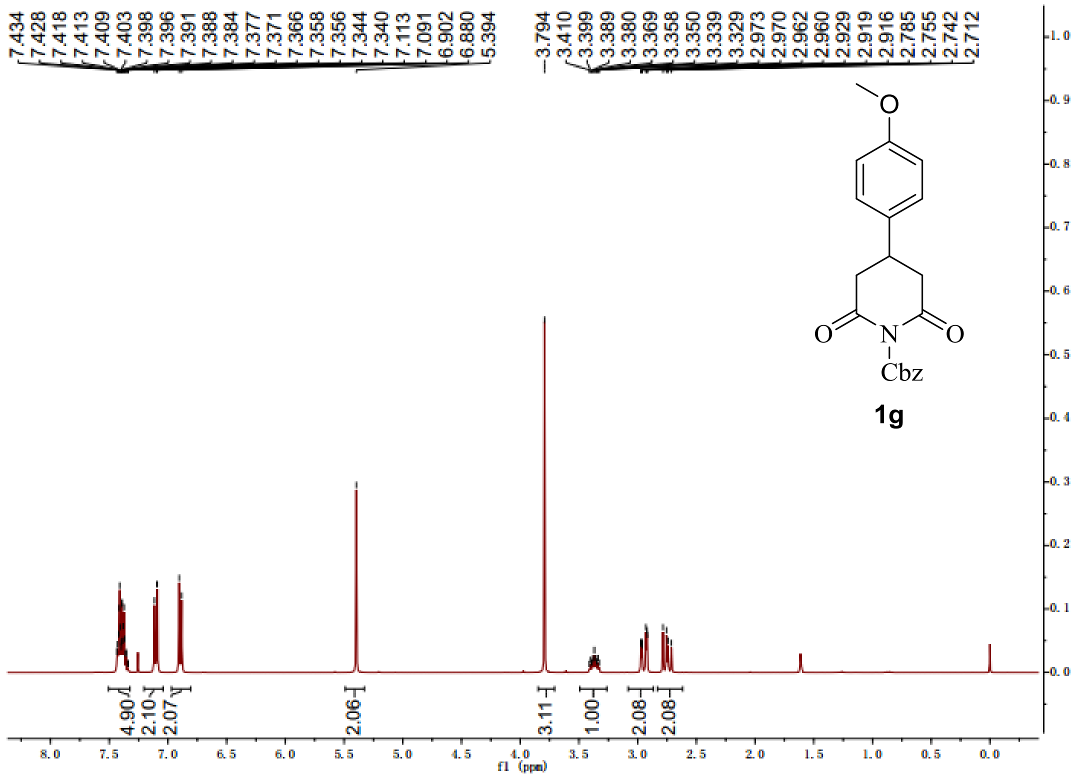

Supplementary Fig. 11. ^{1}H NMR Spectrum of **1g**

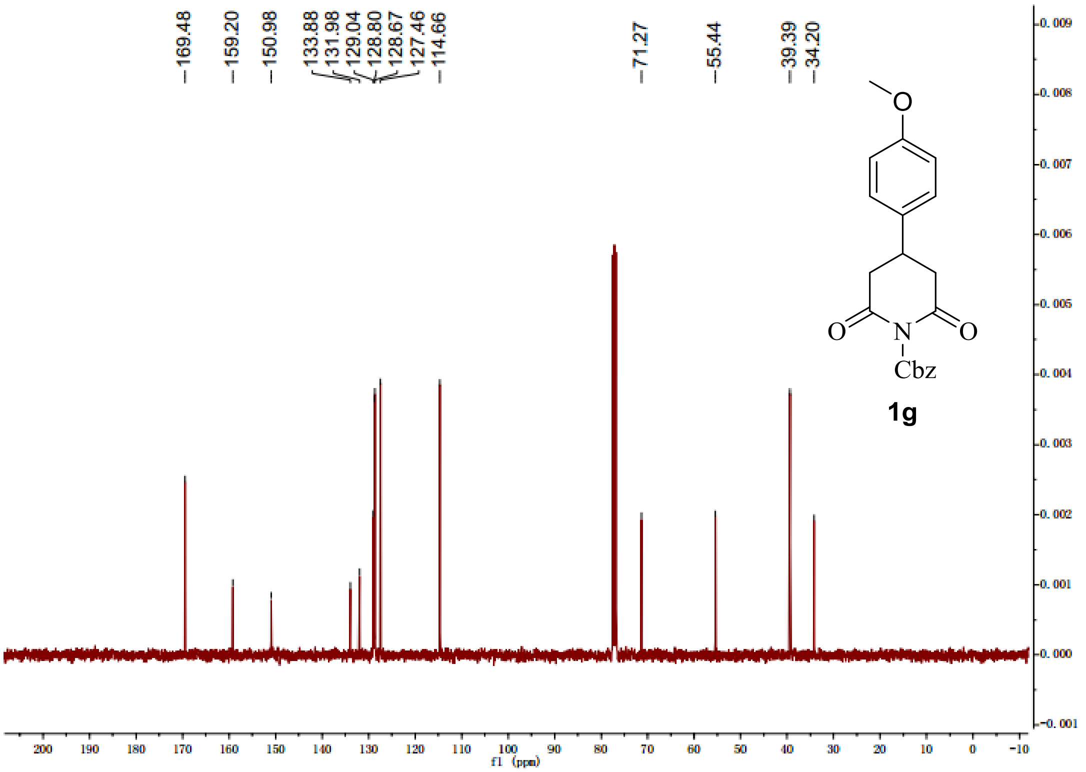

Supplementary Fig. 12. ^{13}C NMR Spectrum of **1g**

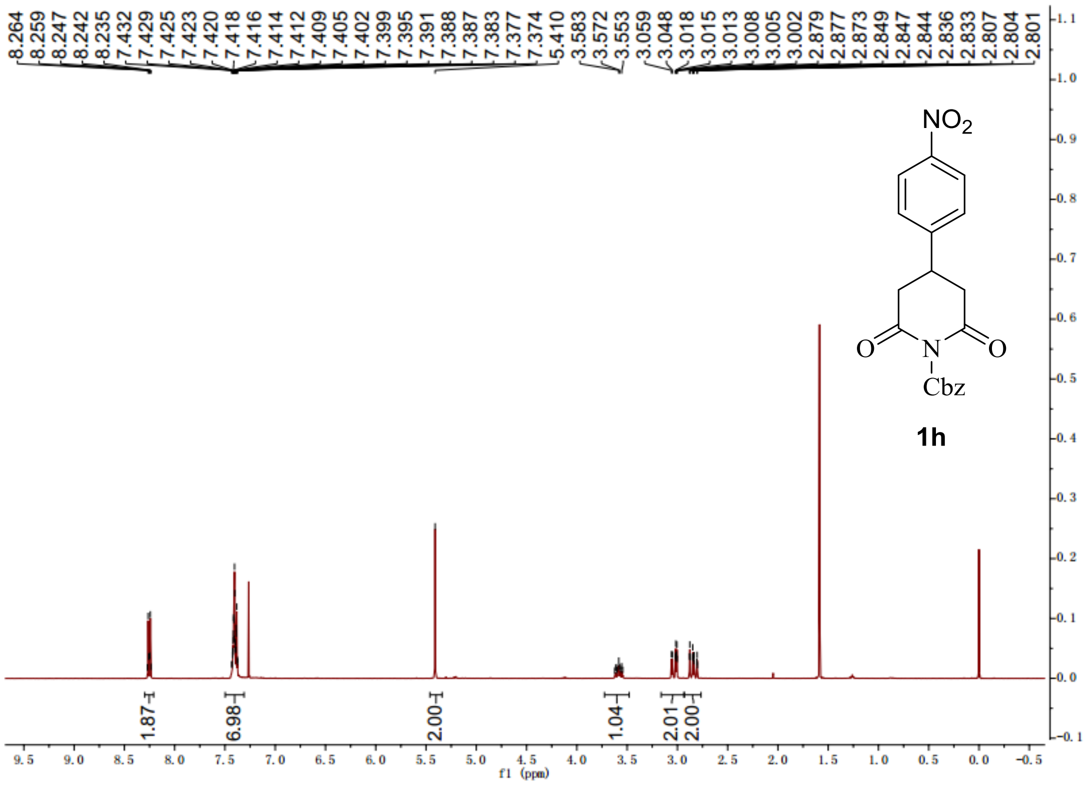

Supplementary Fig. 13. ^{1}H NMR Spectrum of **1h**

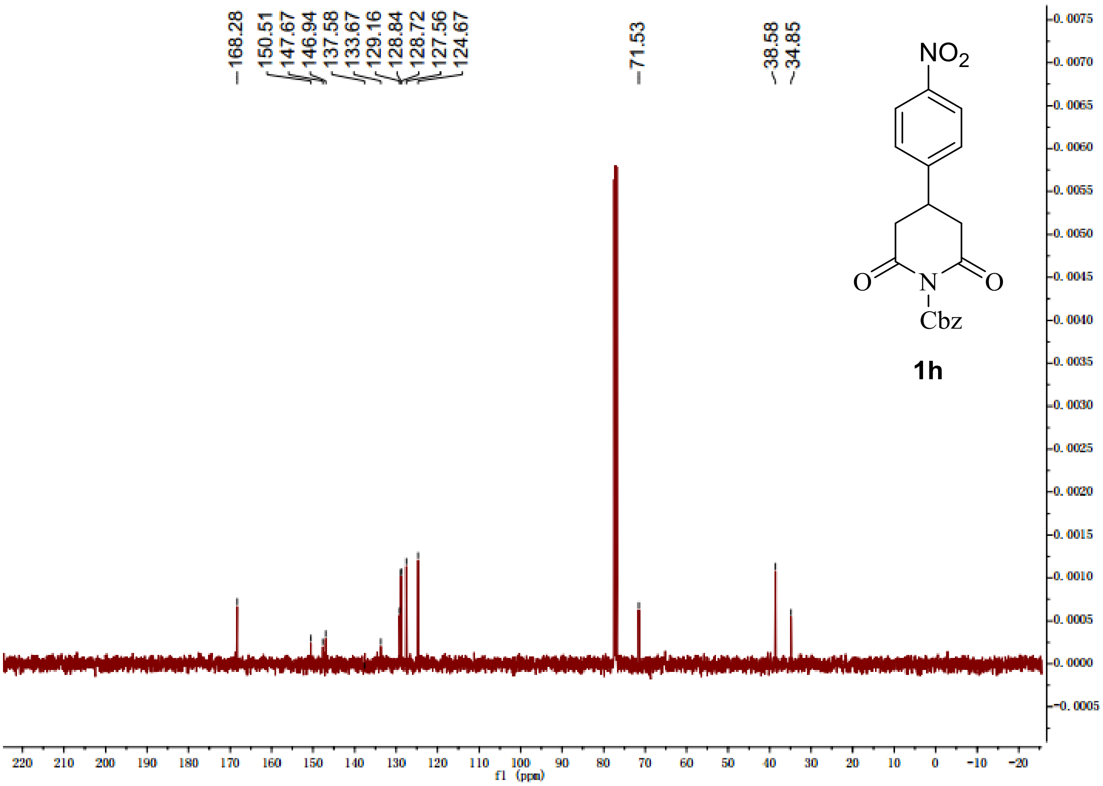

Supplementary Fig. 14. ^{13}C NMR Spectrum of **1h**

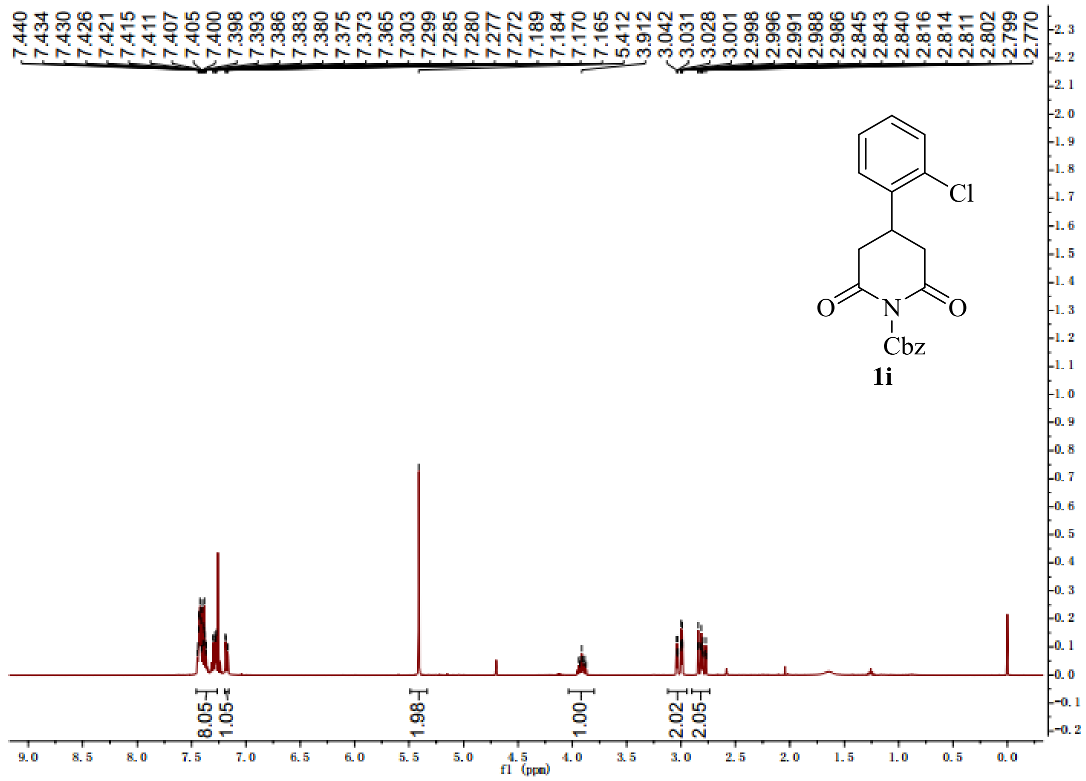

Supplementary Fig. 15. ^{1}H NMR Spectrum of **1i**

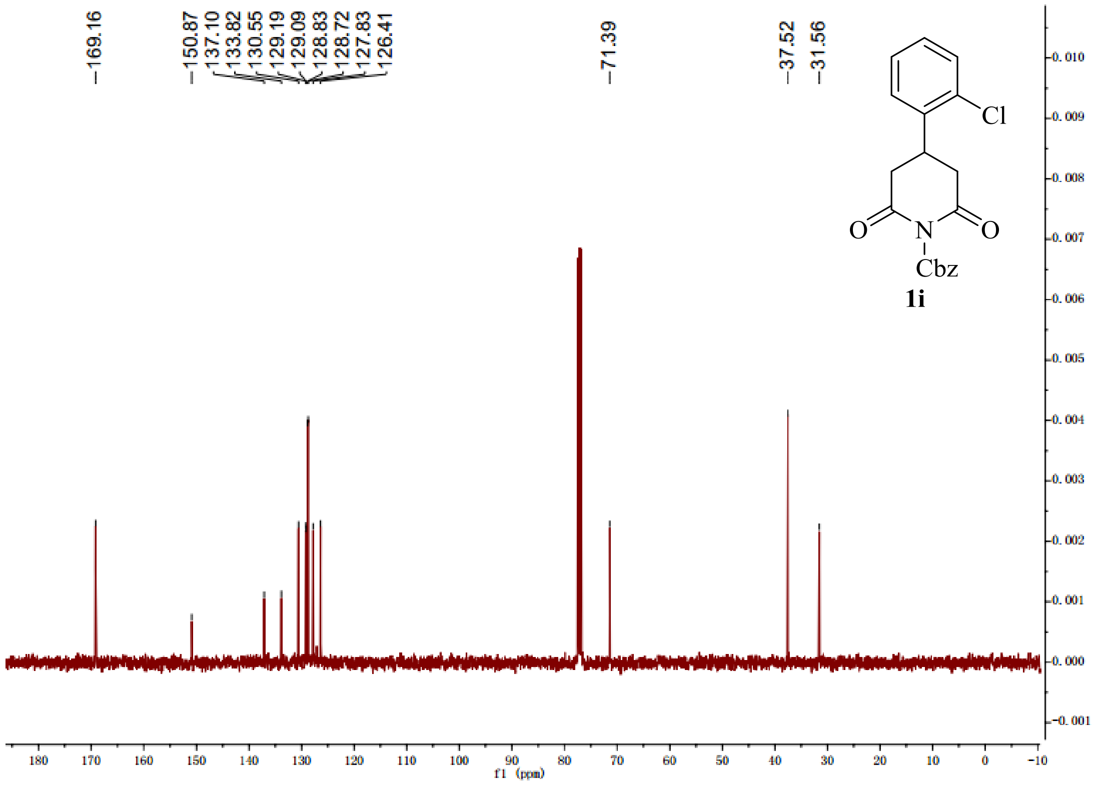

Supplementary Fig. 16. ^{13}C NMR Spectrum of **1i**

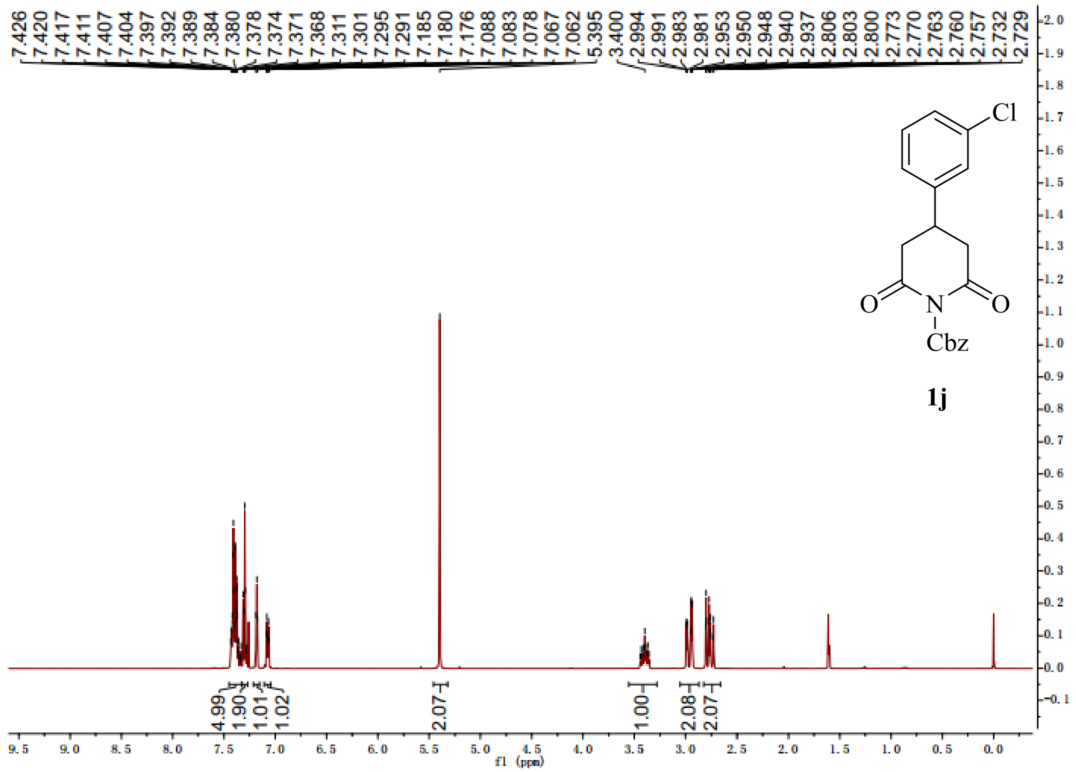

Supplementary Fig. 17. ^{1}H NMR Spectrum of **1j**

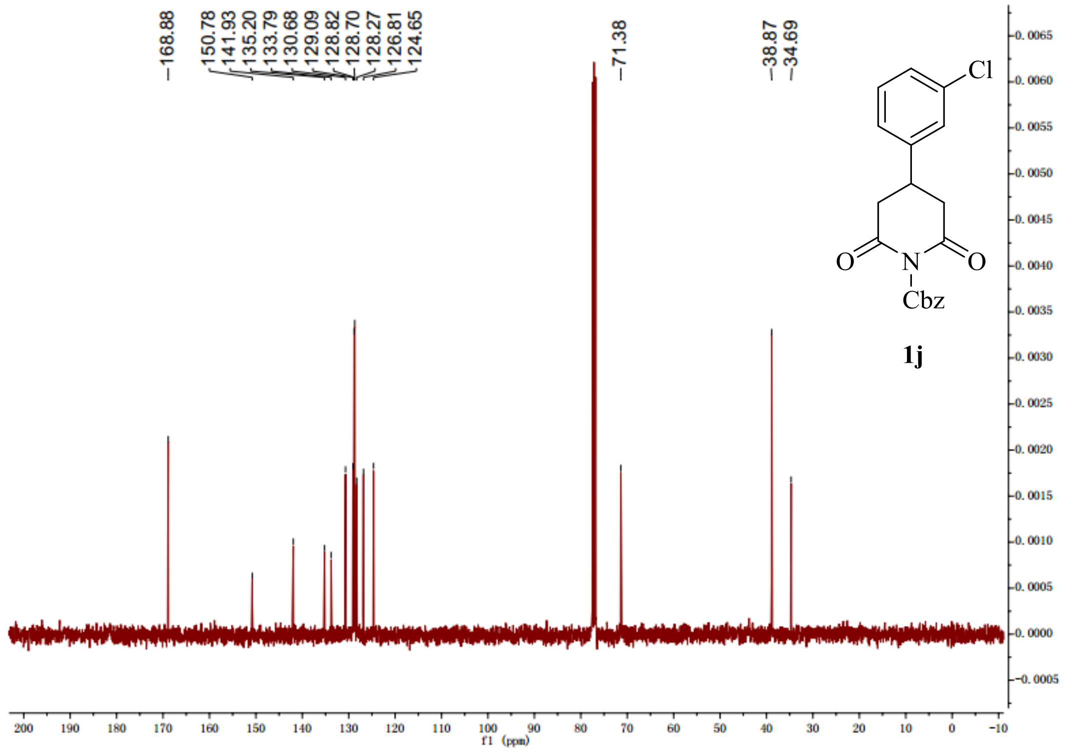

Supplementary Fig. 18. ^{13}C NMR Spectrum of **1j**

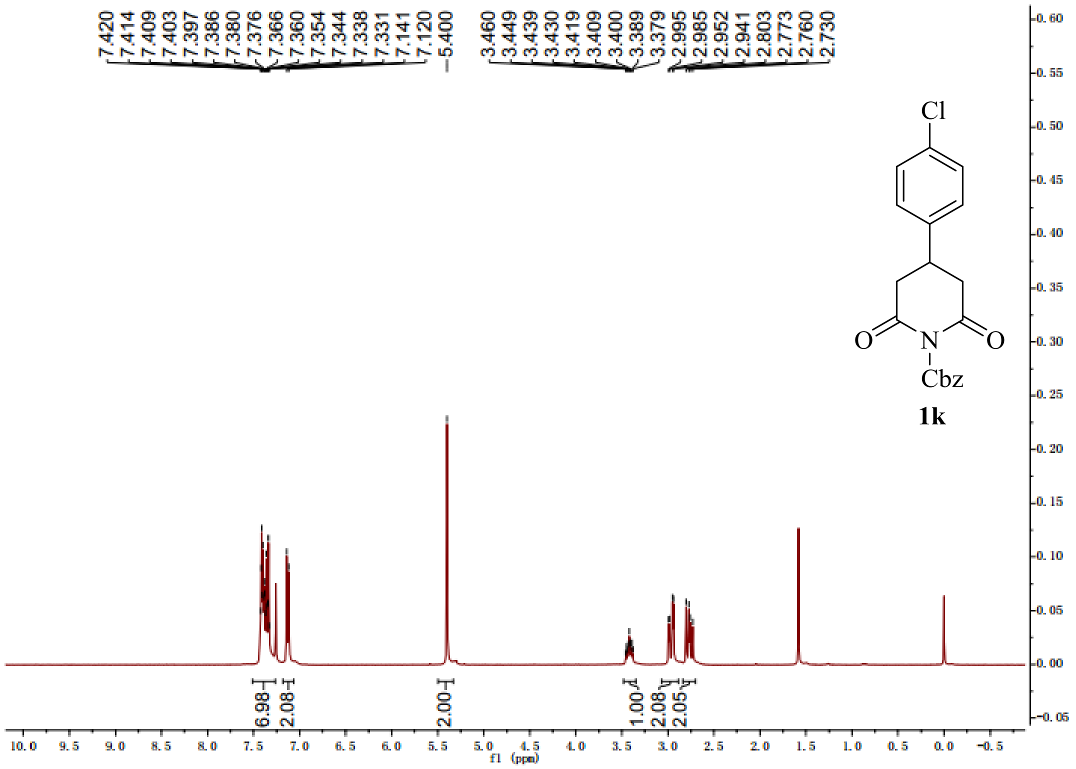

Supplementary Fig. 19. ^{1}H NMR Spectrum of **1k**

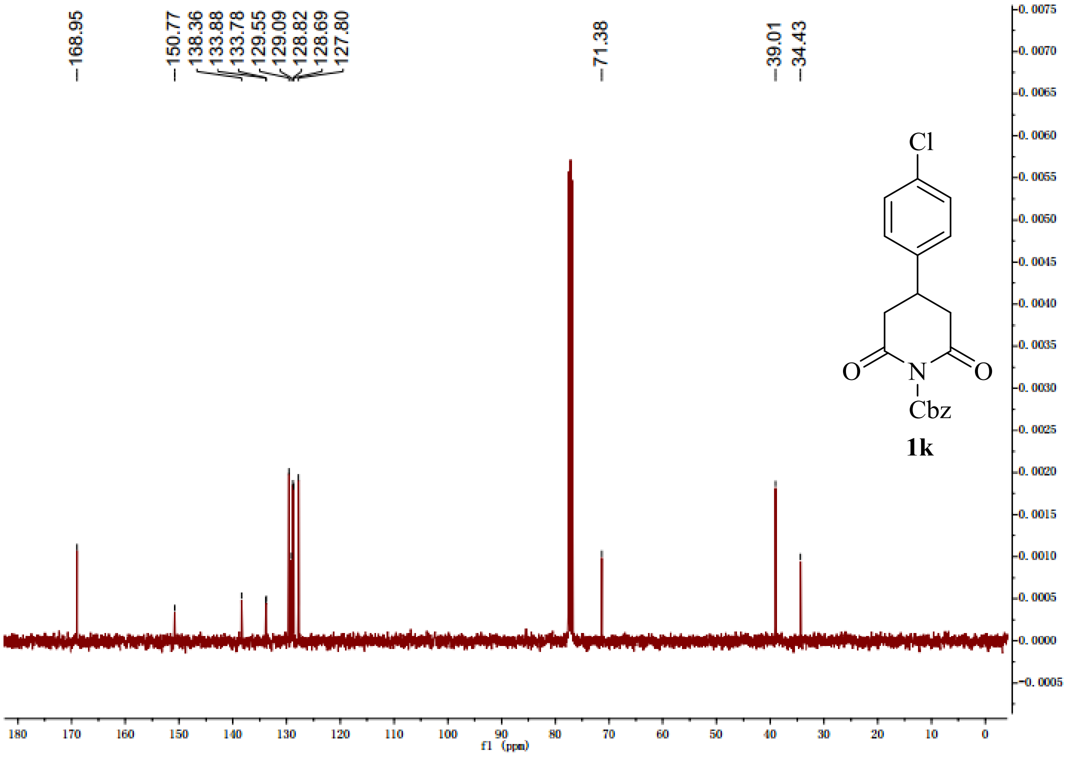

Supplementary Fig. 20. ^{13}C NMR Spectrum of **1k**

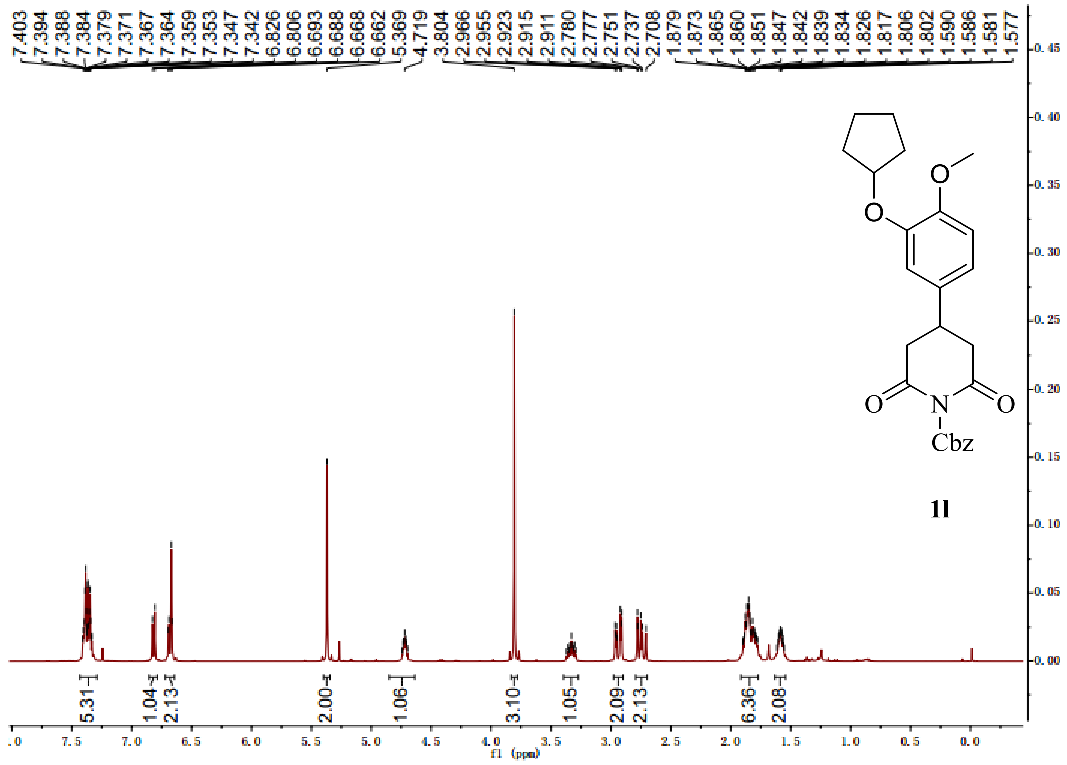

Supplementary Fig. 21. ^{1}H NMR Spectrum of **1l**

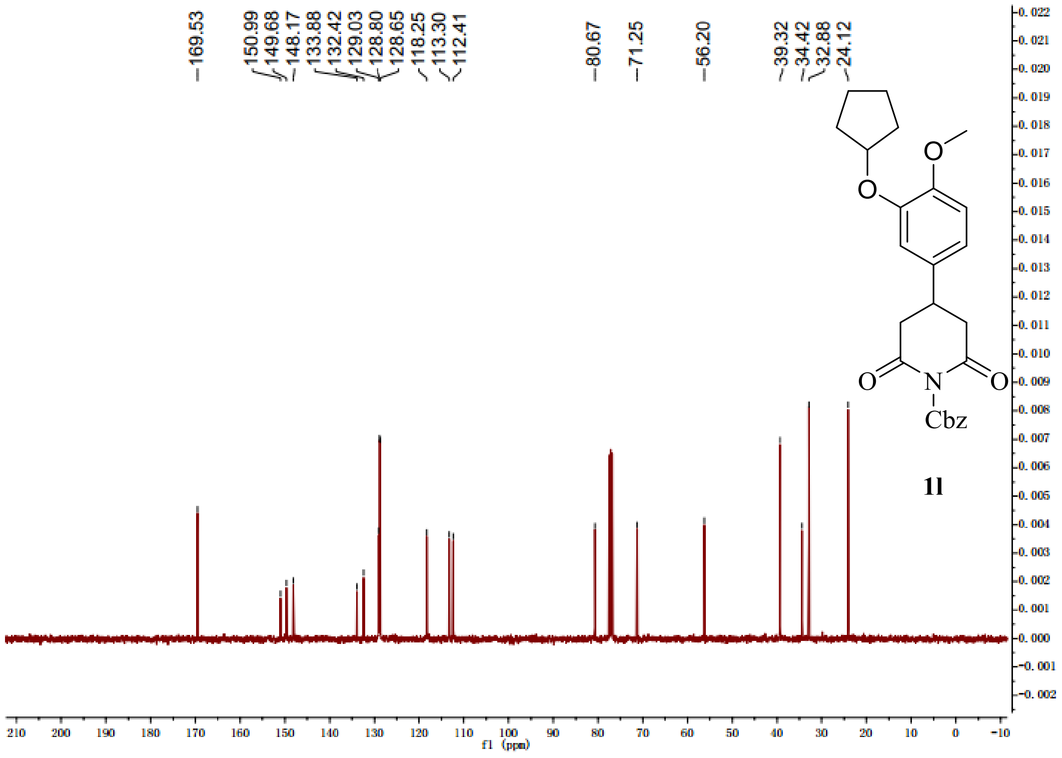

Supplementary Fig. 22. ^{13}C NMR Spectrum of **1l**

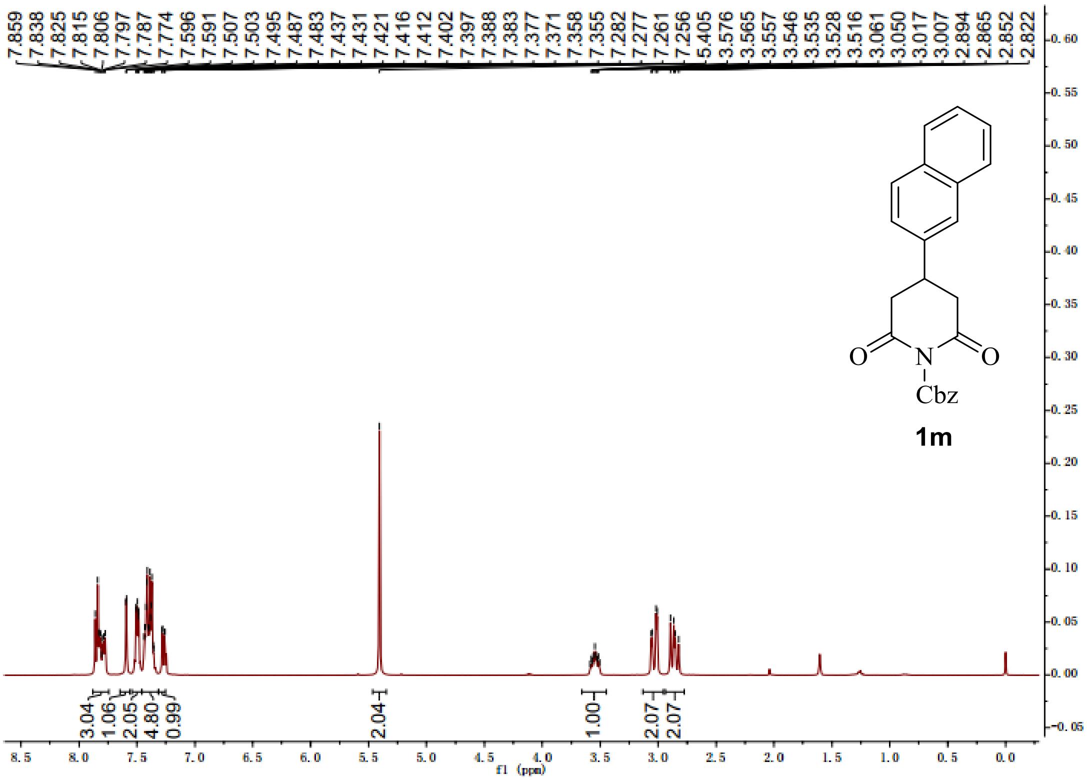

Supplementary Fig. 23. ^{1}H NMR Spectrum of **1m**

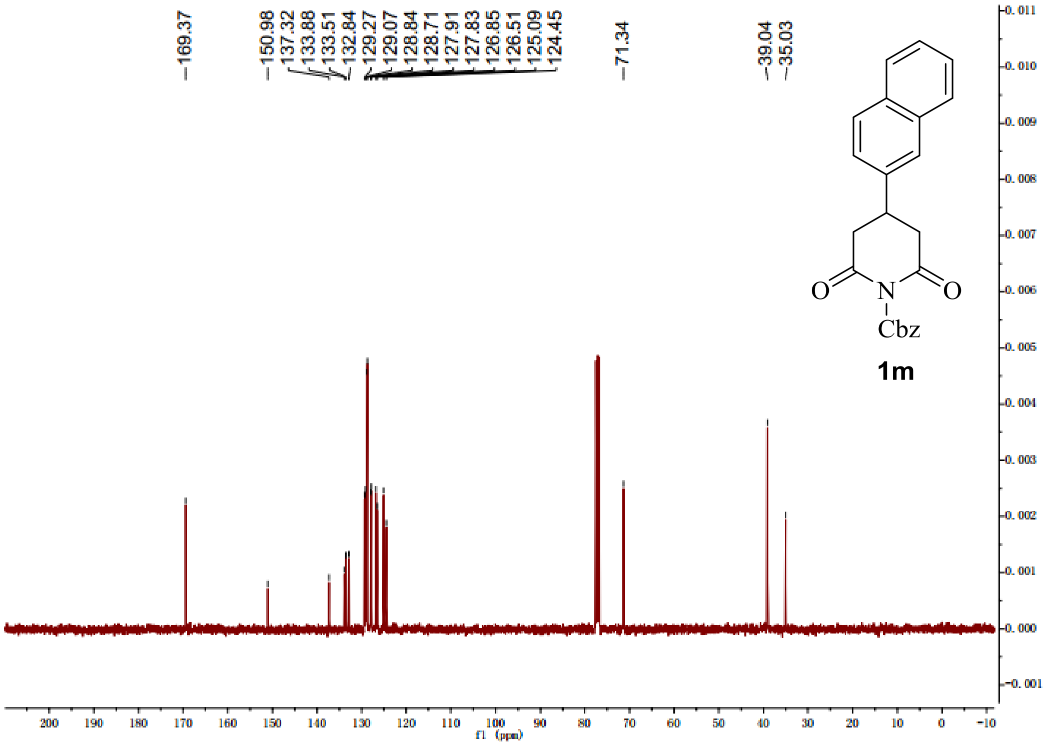

Supplementary Fig. 24. ^{13}C NMR Spectrum of **1m**

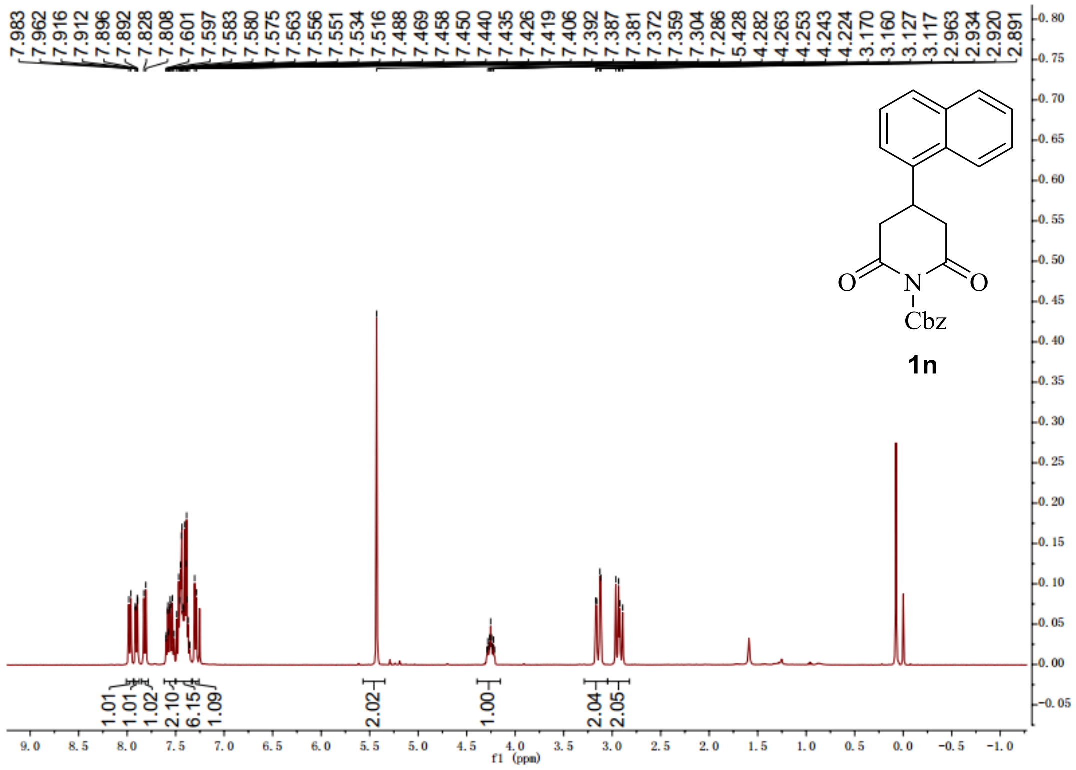

Supplementary Fig. 25. ^{1}H NMR Spectrum of **1n**

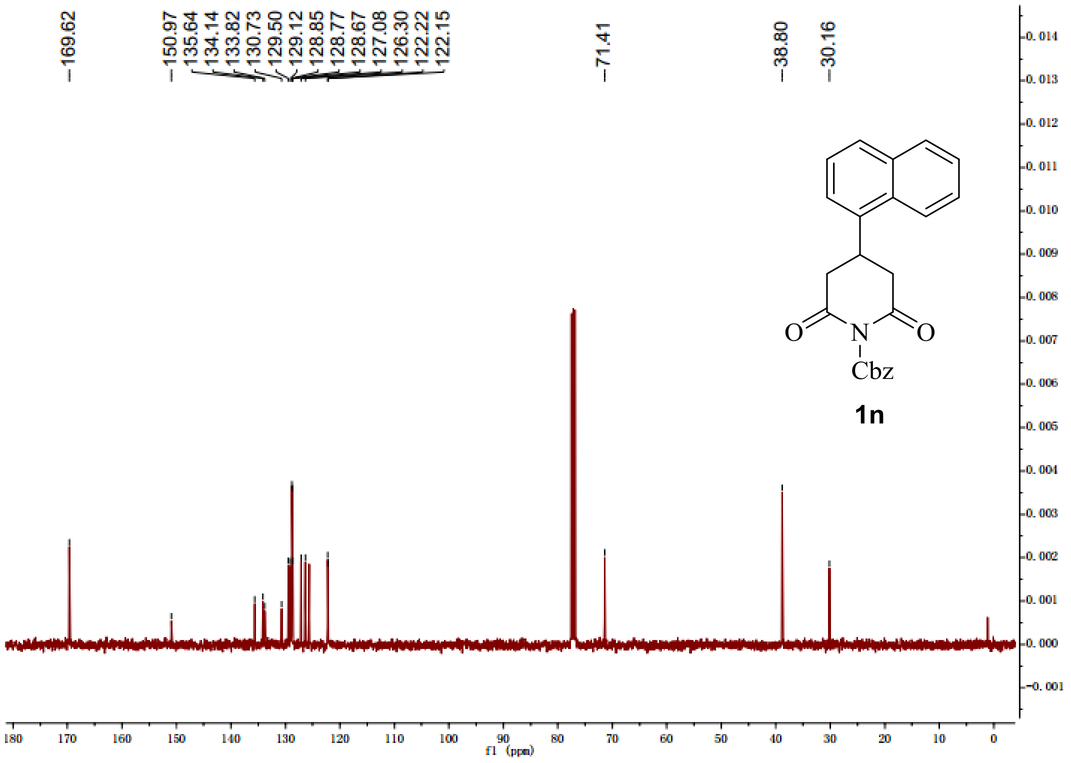

Supplementary Fig. 26. ^{13}C NMR Spectrum of **1n**

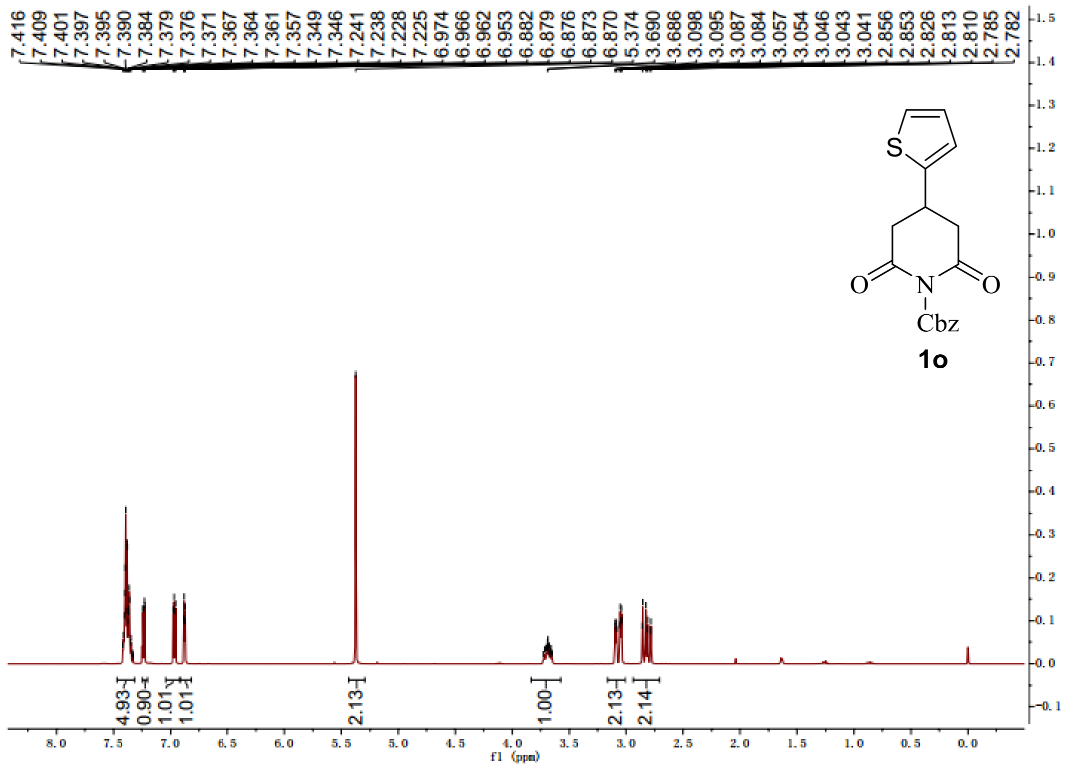

Supplementary Fig. 27. ^{1}H NMR Spectrum of **1o**

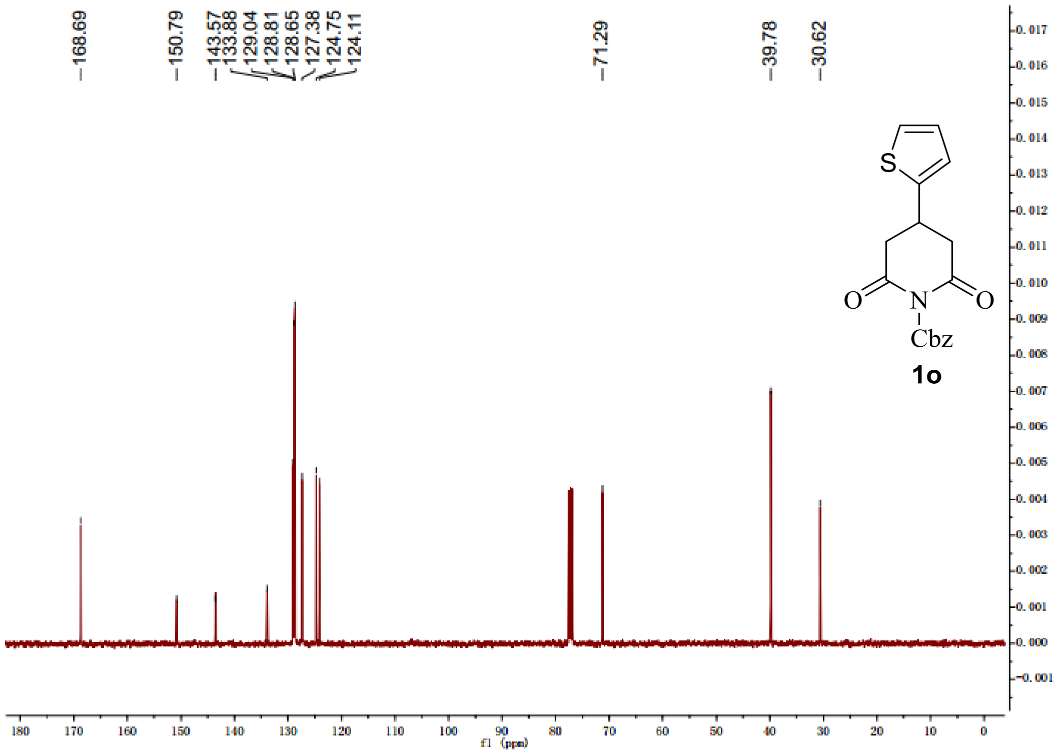

Supplementary Fig. 28. ^{13}C NMR Spectrum of **1o**

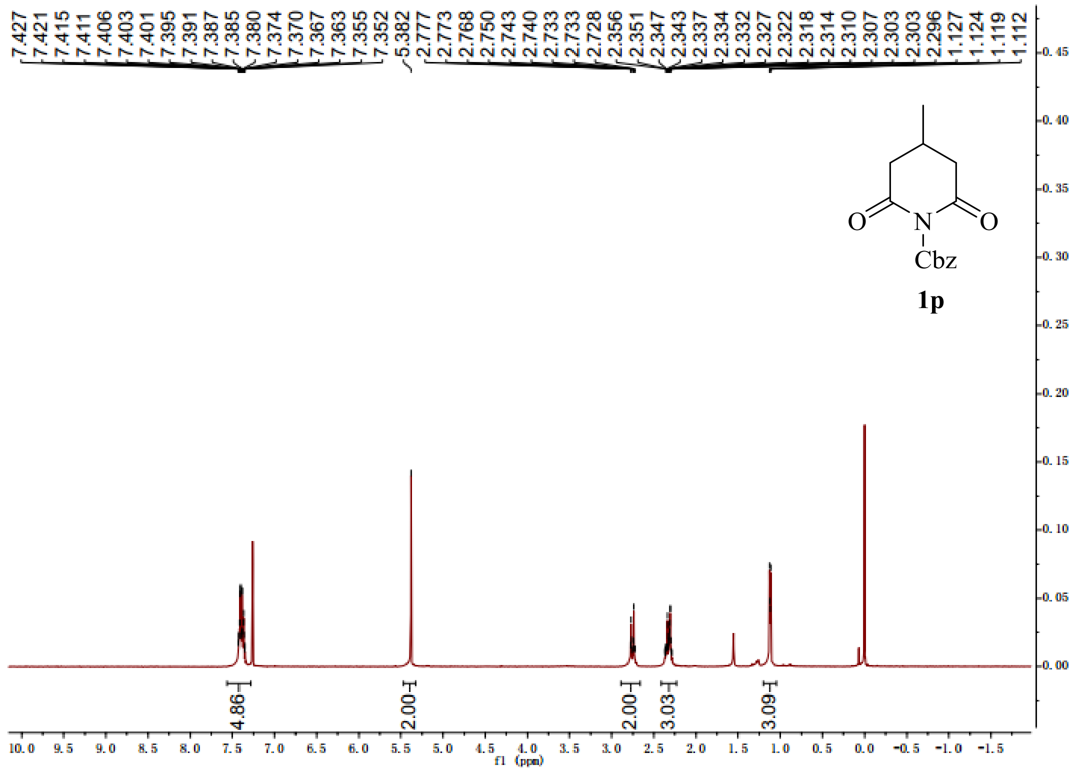

Supplementary Fig. 29. ^{1}H NMR Spectrum of **1p**

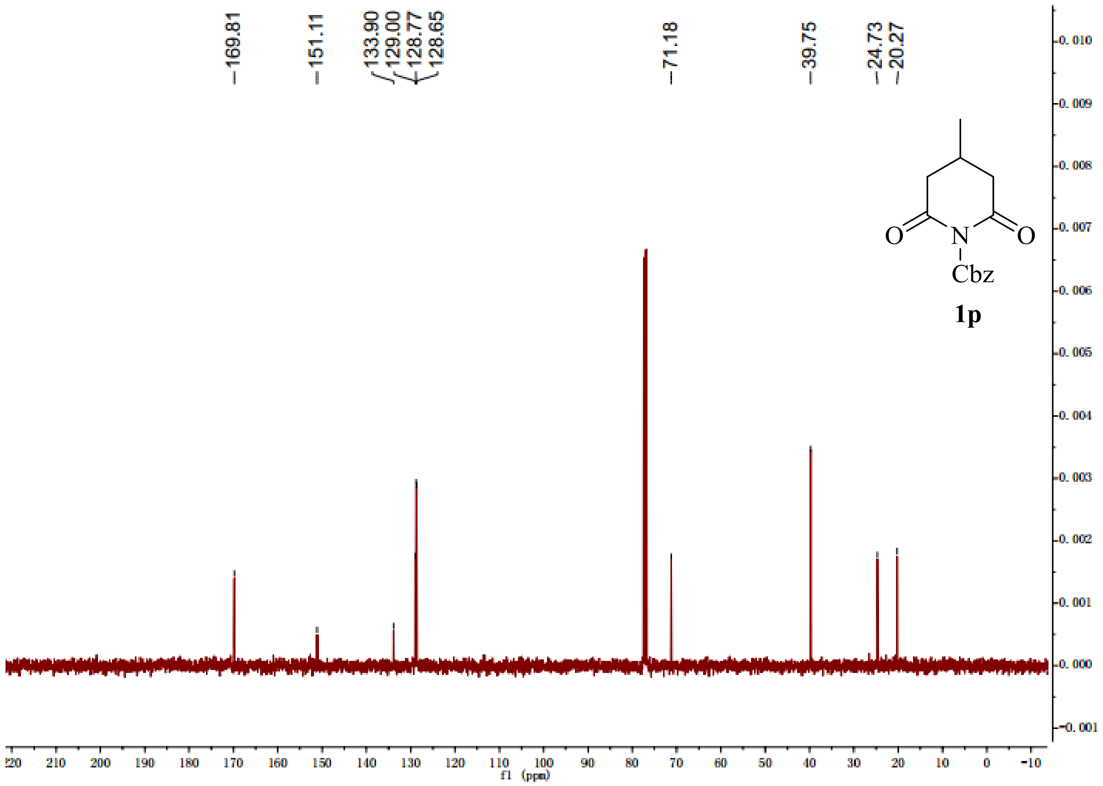

Supplementary Fig. 30. ^{13}C NMR Spectrum of **1p**

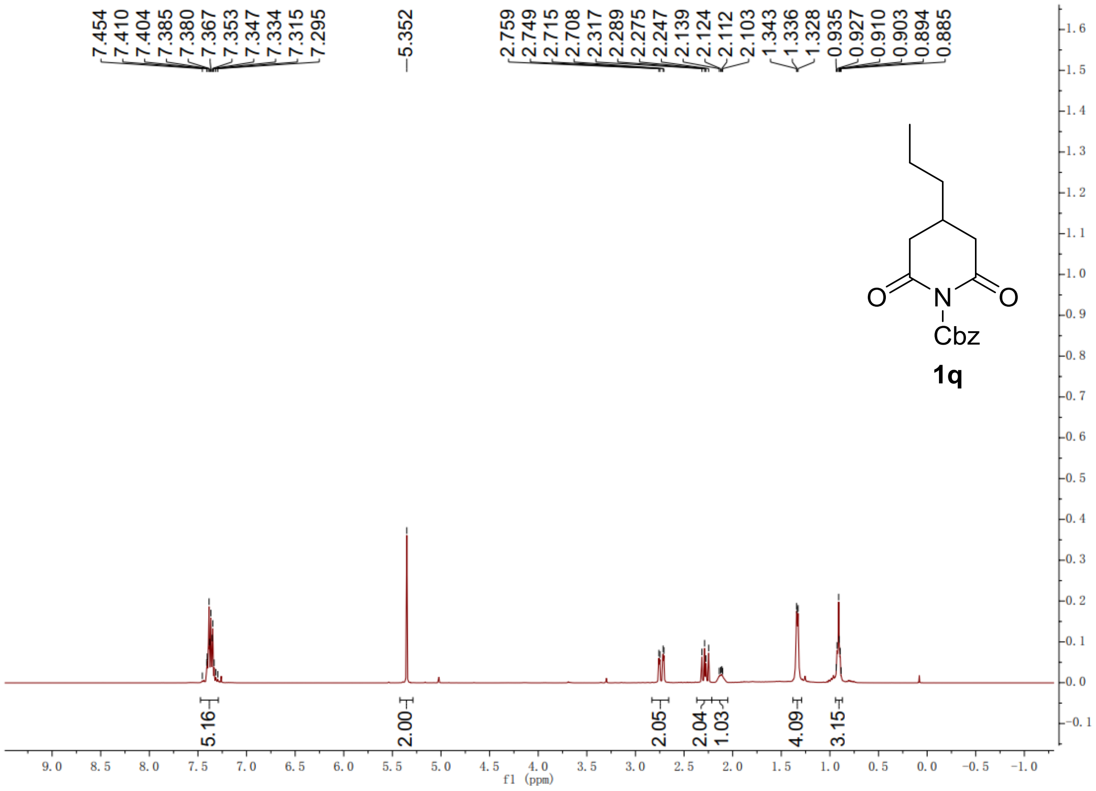

Supplementary Fig. 31. ^{1}H NMR Spectrum of **1q**

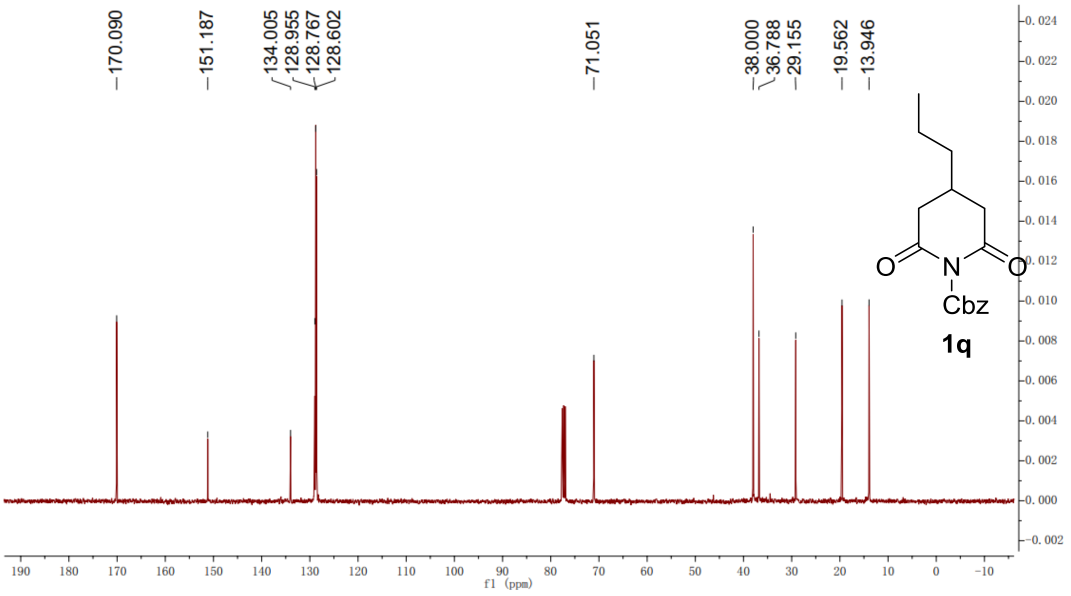

Supplementary Fig. 32. ^{13}C NMR Spectrum of **1q**

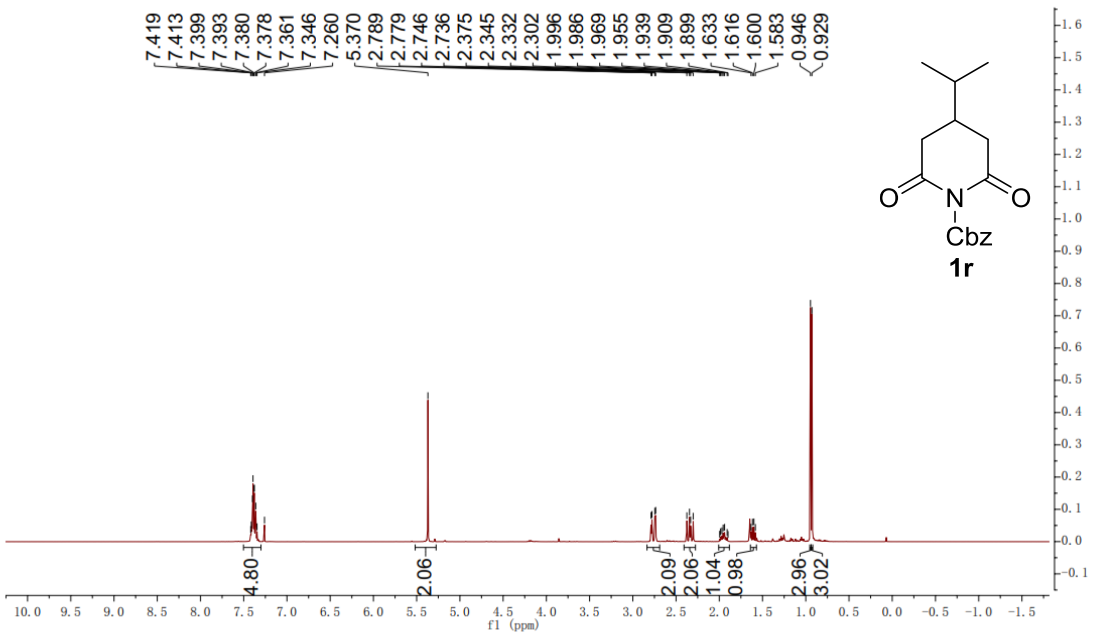

Supplementary Fig. 33. ^{1}H NMR Spectrum of **1r**

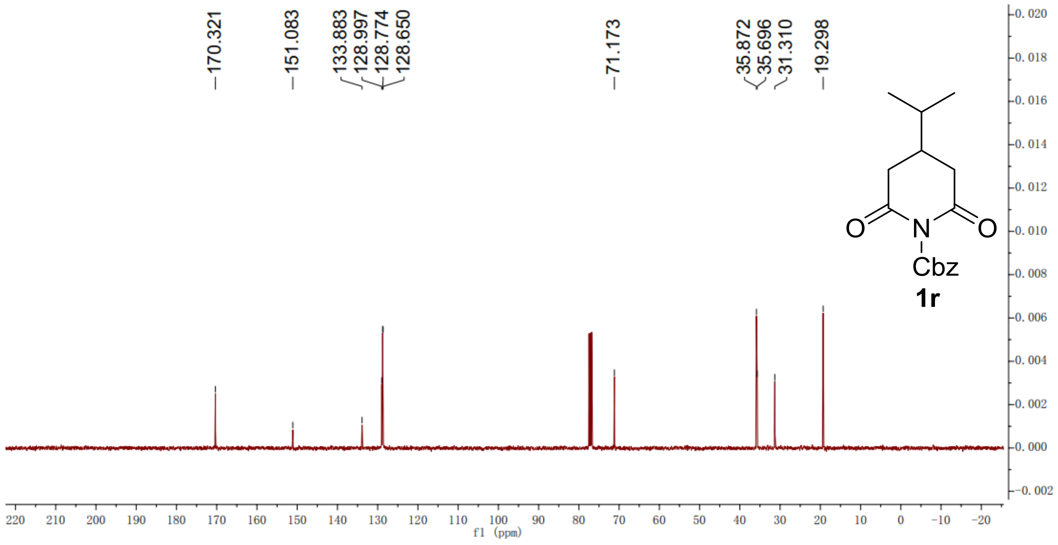

Supplementary Fig. 34. ^{13}C NMR Spectrum of **1r**

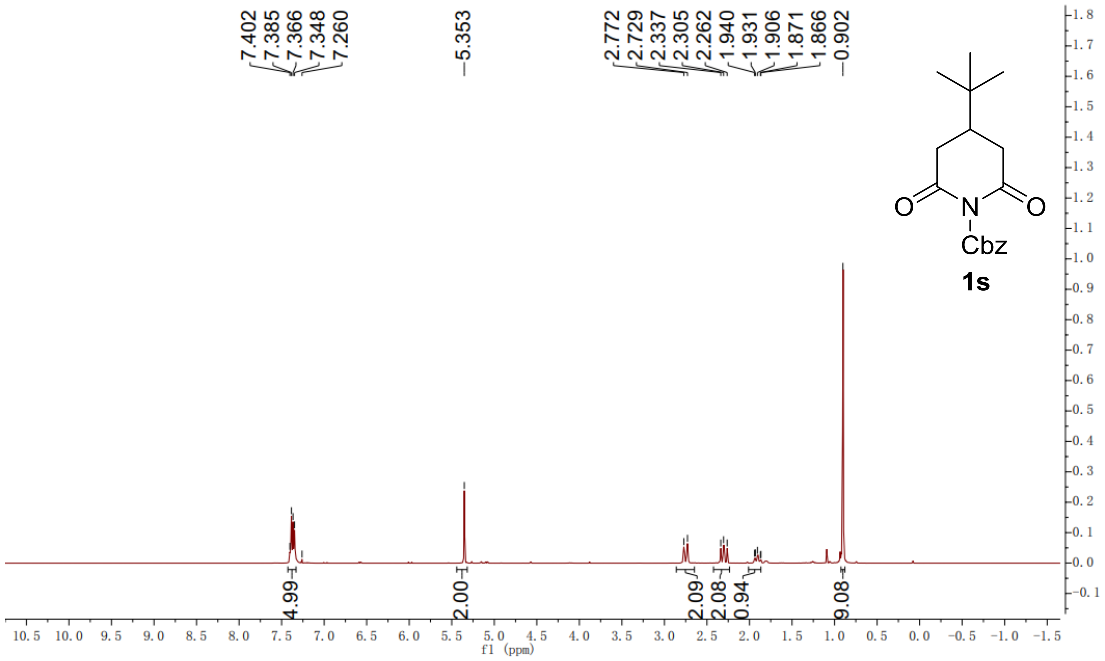

Supplementary Fig. 35. ^{1}H NMR Spectrum of **1s**

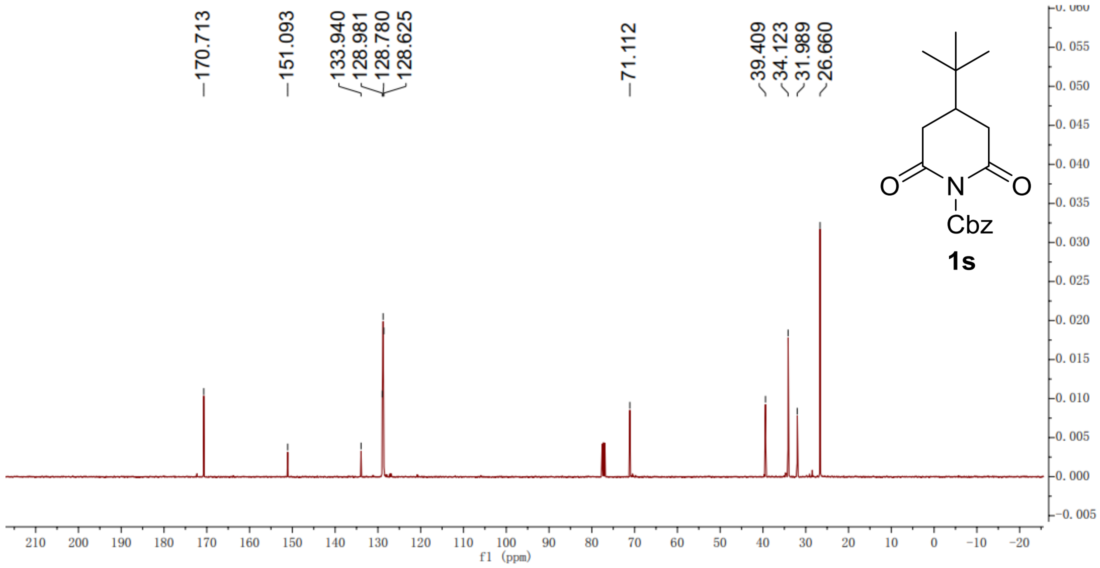

Supplementary Fig. 36. ^{13}C NMR Spectrum of **1s**

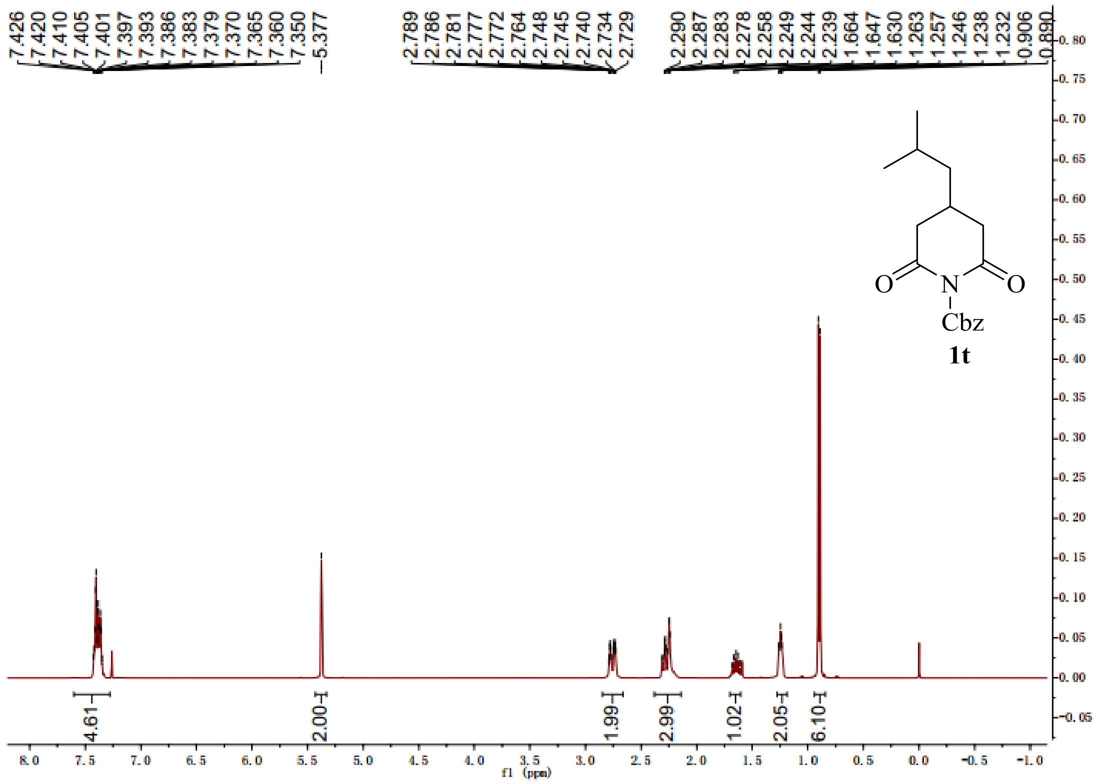

Supplementary Fig. 37. ^{1}H NMR Spectrum of **1t**

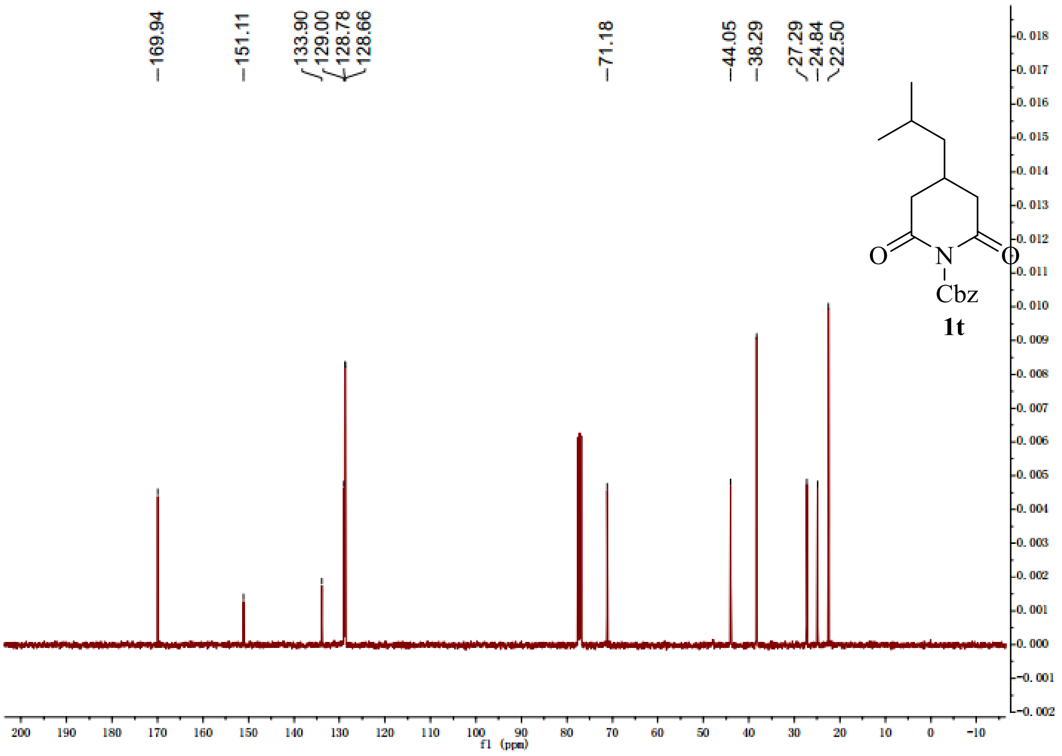

Supplementary Fig. 38. ^{13}C NMR Spectrum of **1t**

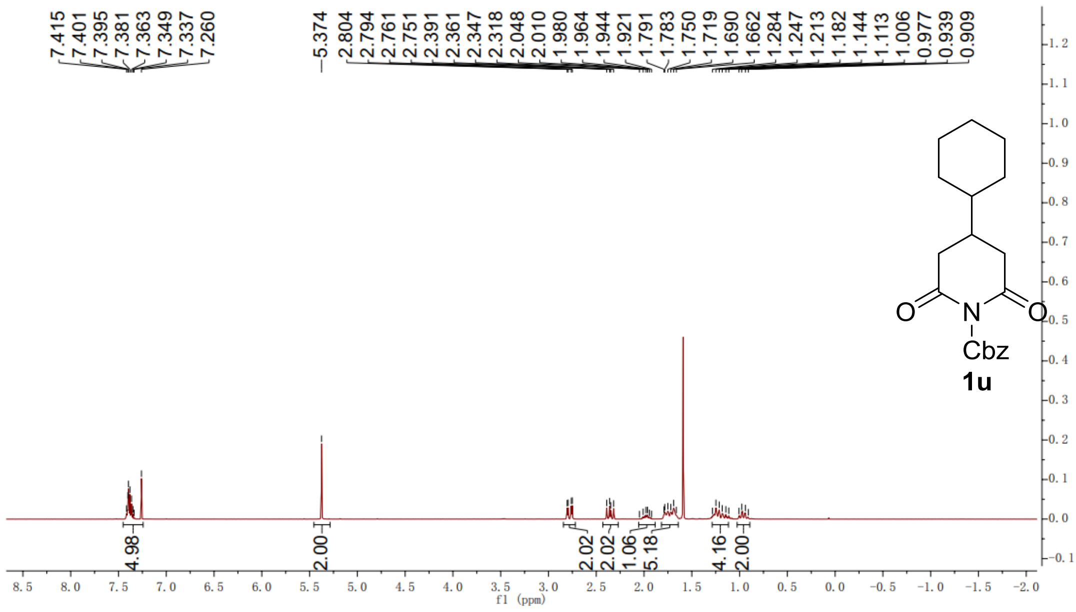

Supplementary Fig. 39. ^{1}H NMR Spectrum of **1u**

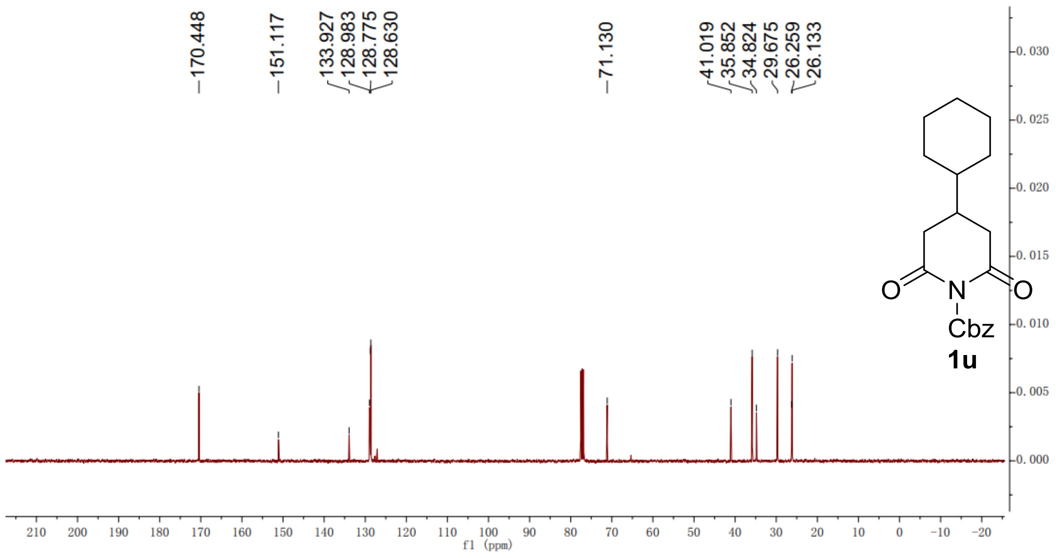

Supplementary Fig. 40. ^{13}C NMR Spectrum of **1u**

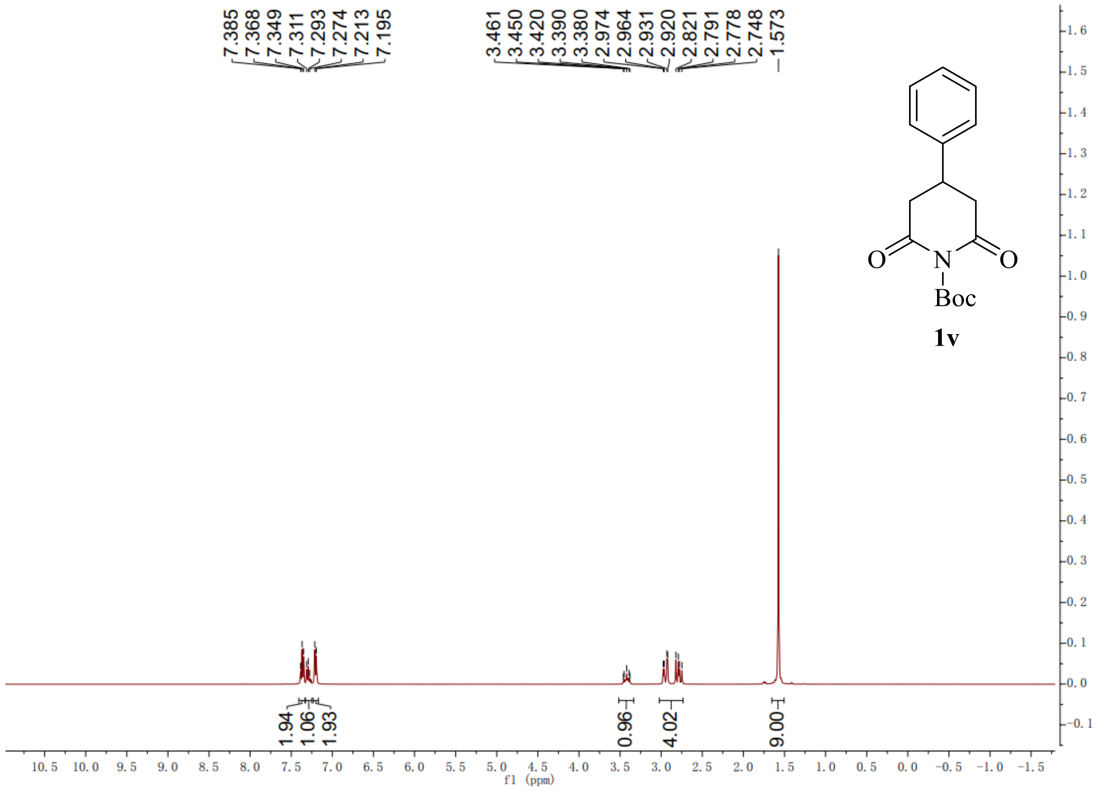

Supplementary Fig. 41. ^{1}H NMR Spectrum of **1v**

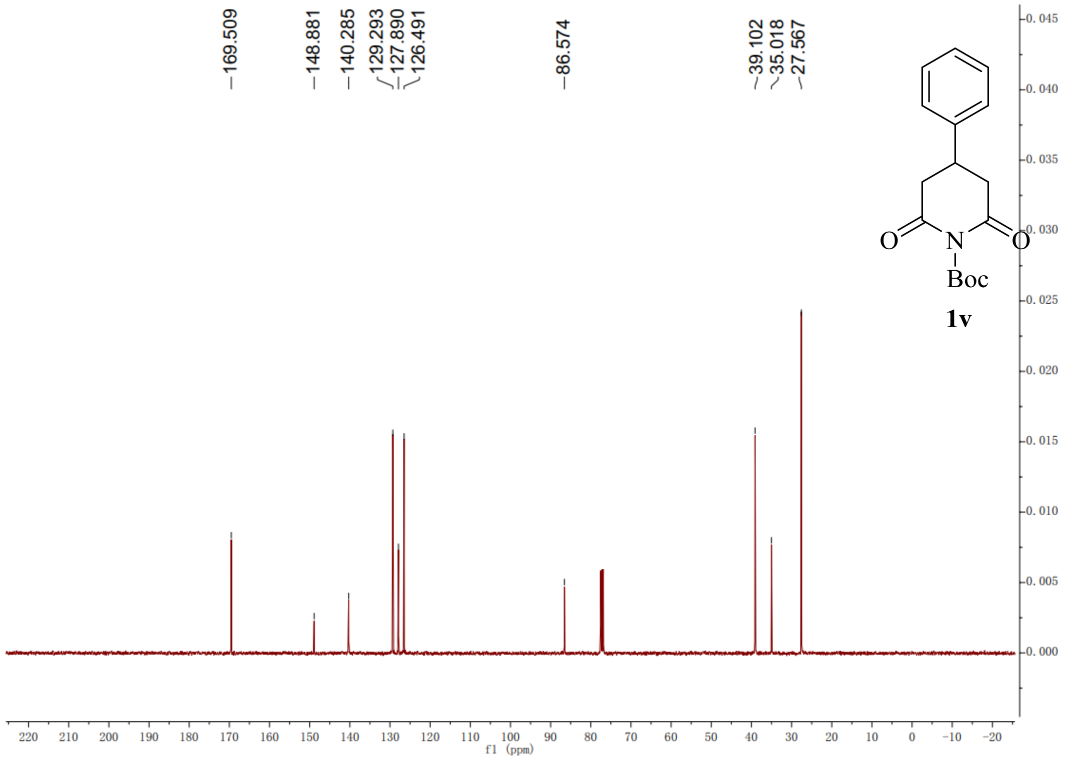

Supplementary Fig. 42. ^{13}C NMR Spectrum of **1v**

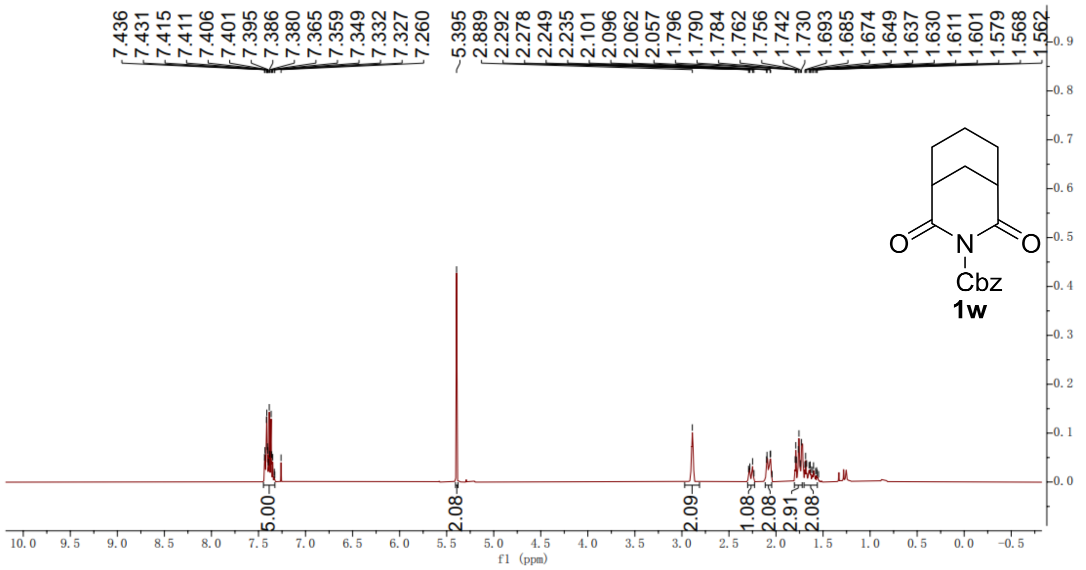

Supplementary Fig. 43. ^{1}H NMR Spectrum of **1w**

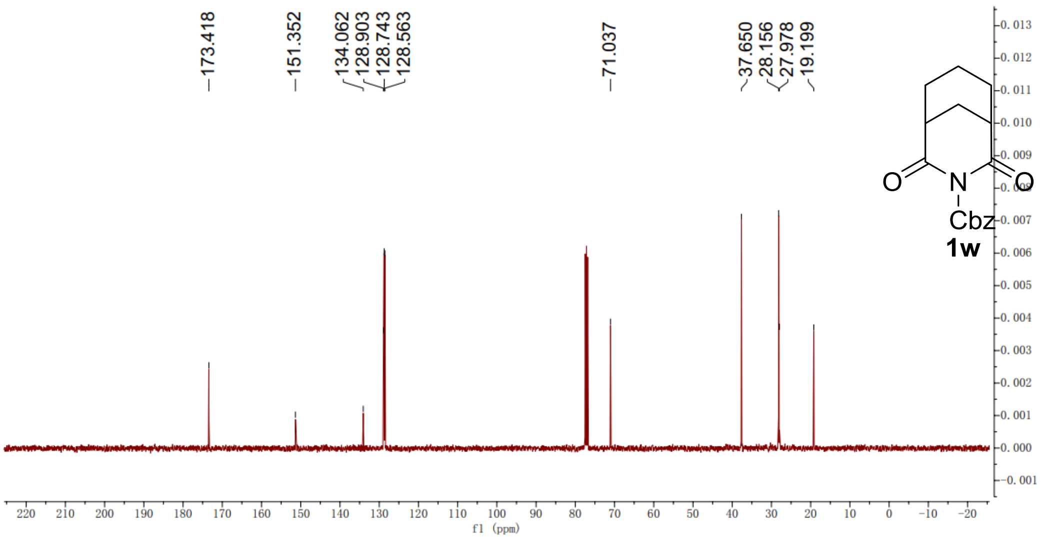

Supplementary Fig. 44. ^{13}C NMR Spectrum of **1w**

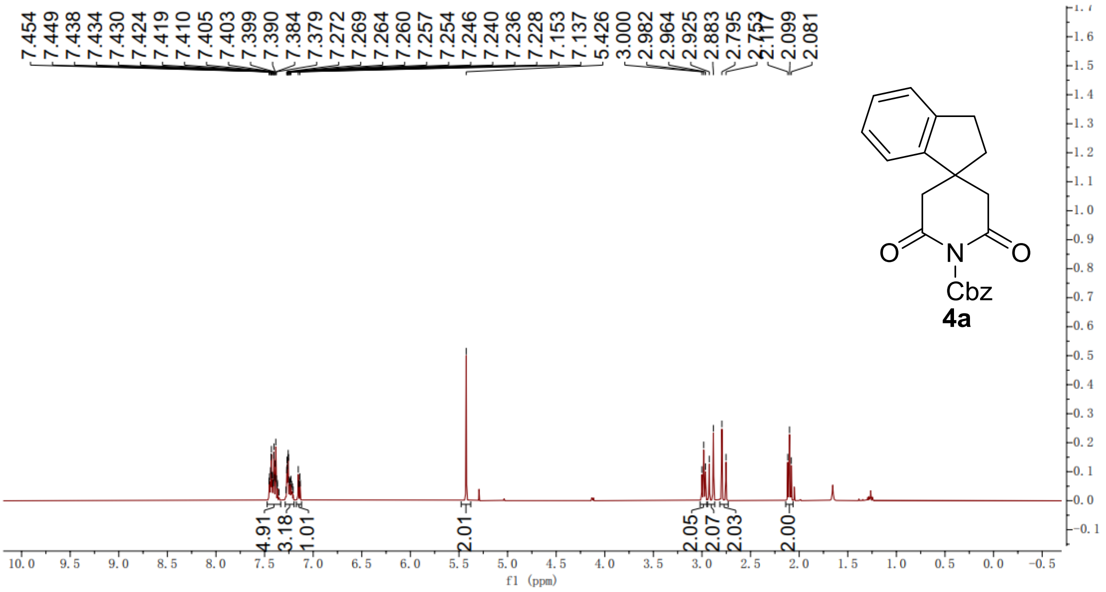

Supplementary Fig. 45. ^{1}H NMR Spectrum of **4a**

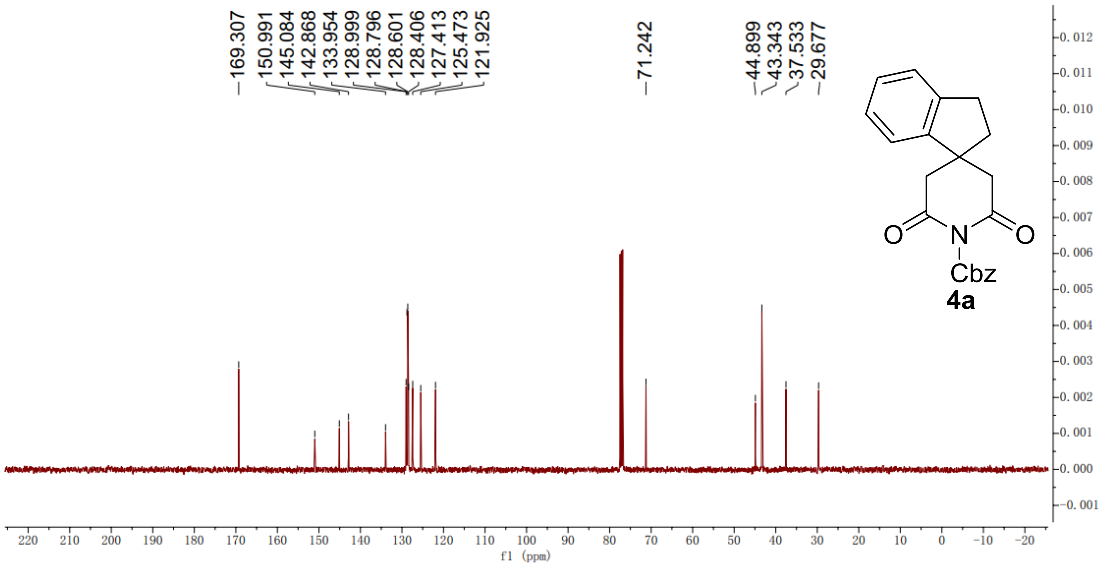

Supplementary Fig. 46. ^{13}C NMR Spectrum of **4a**

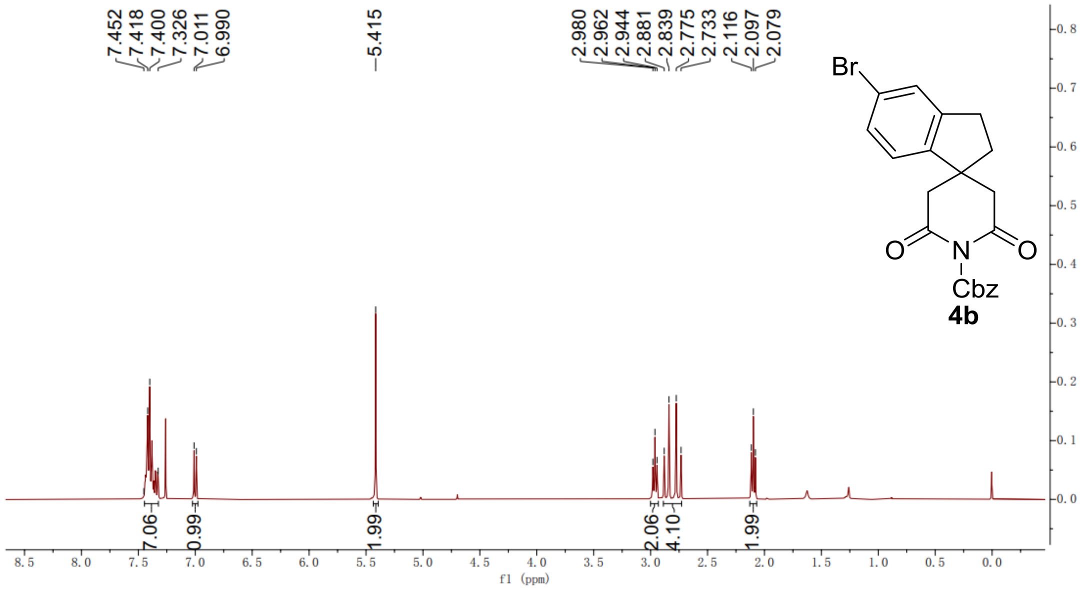

Supplementary Fig. 47. ^{1}H NMR Spectrum of **4b**

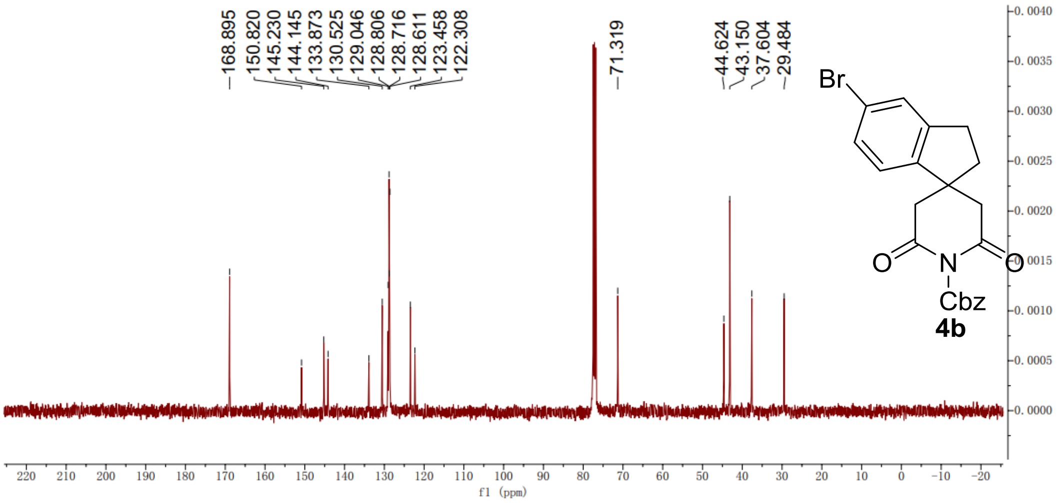

Supplementary Fig. 48. ^{13}C NMR Spectrum of **4b**

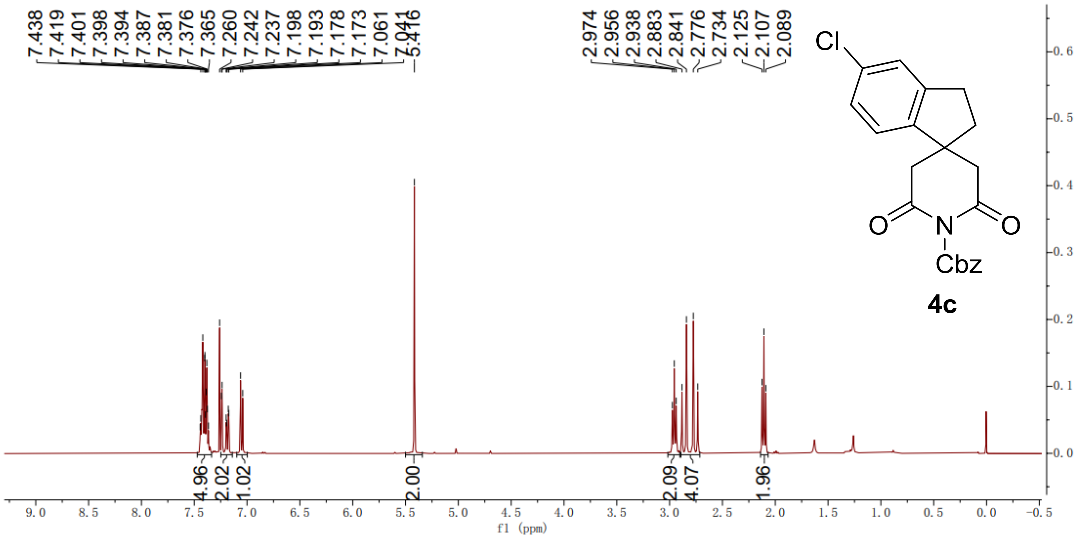

Supplementary Fig. 49. ^{1}H NMR Spectrum of **4c**

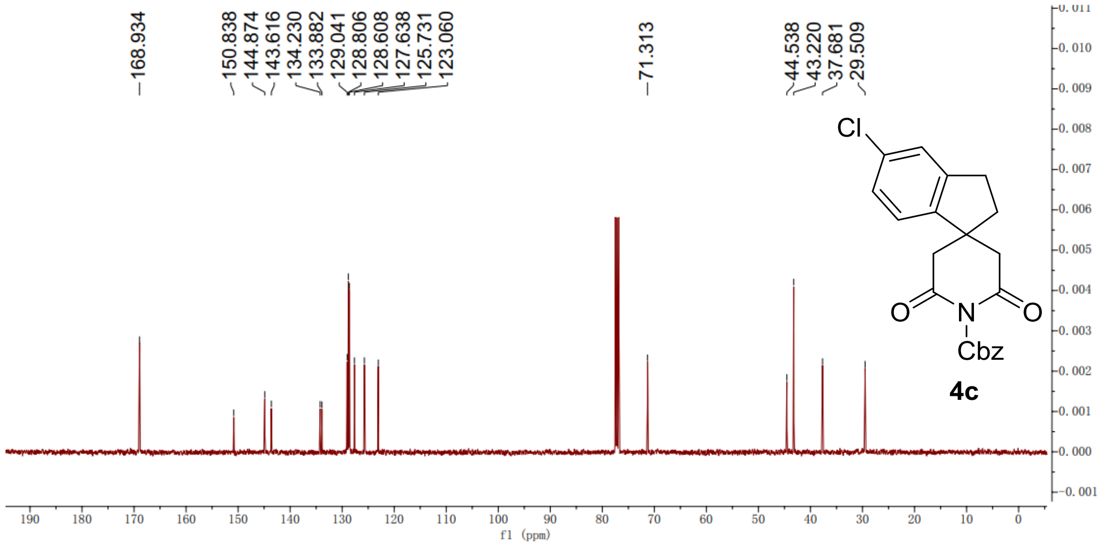

Supplementary Fig. 50. ^{13}C NMR Spectrum of **4c**

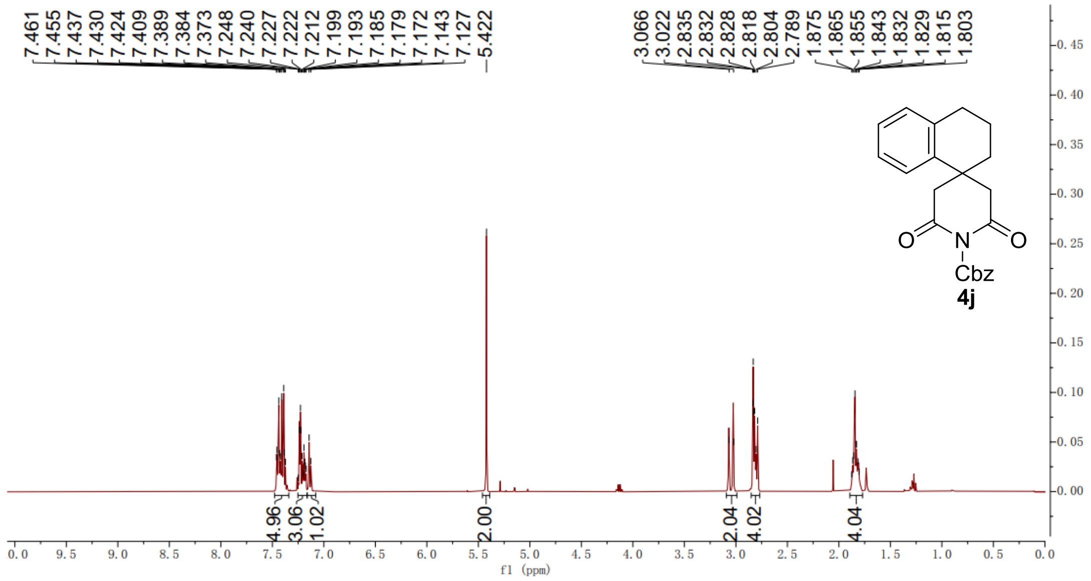

Supplementary Fig. 51. ^{1}H NMR Spectrum of **4j**

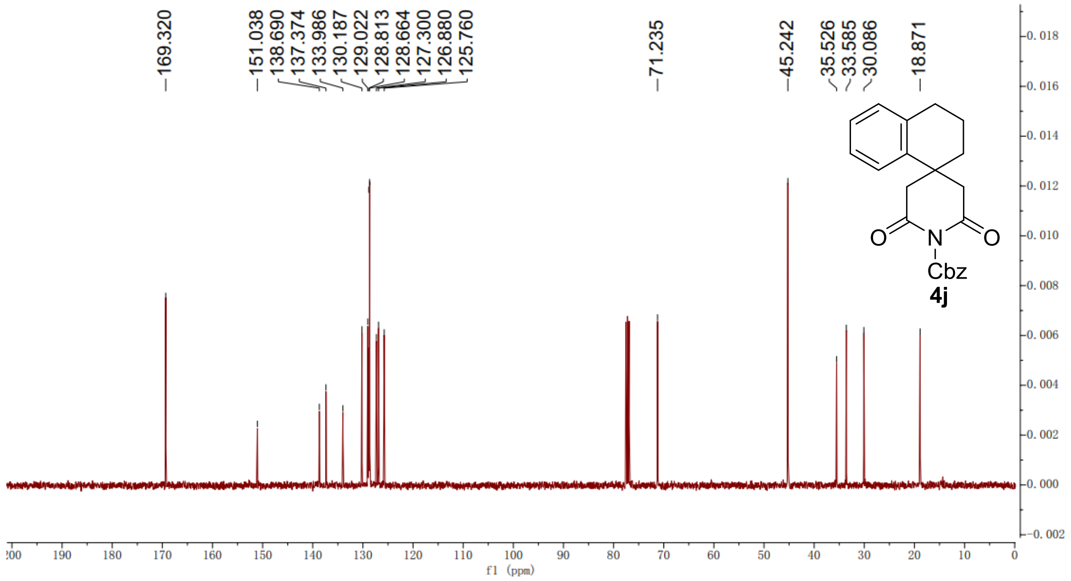

Supplementary Fig. 52. ^{13}C NMR Spectrum of **4j**

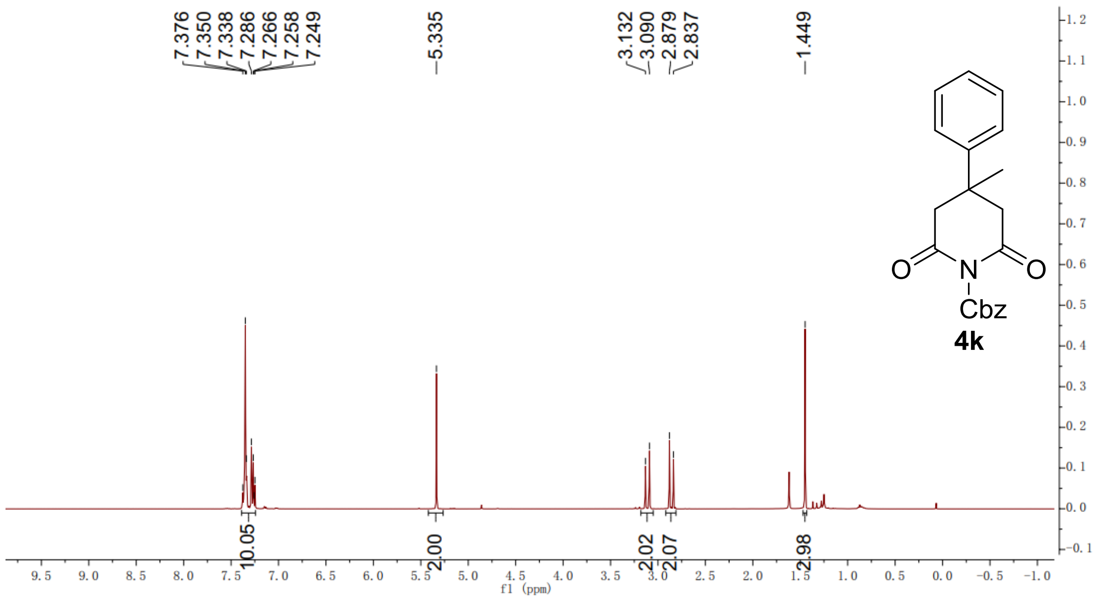

Supplementary Fig. 53. ^{1}H NMR Spectrum of **4k**

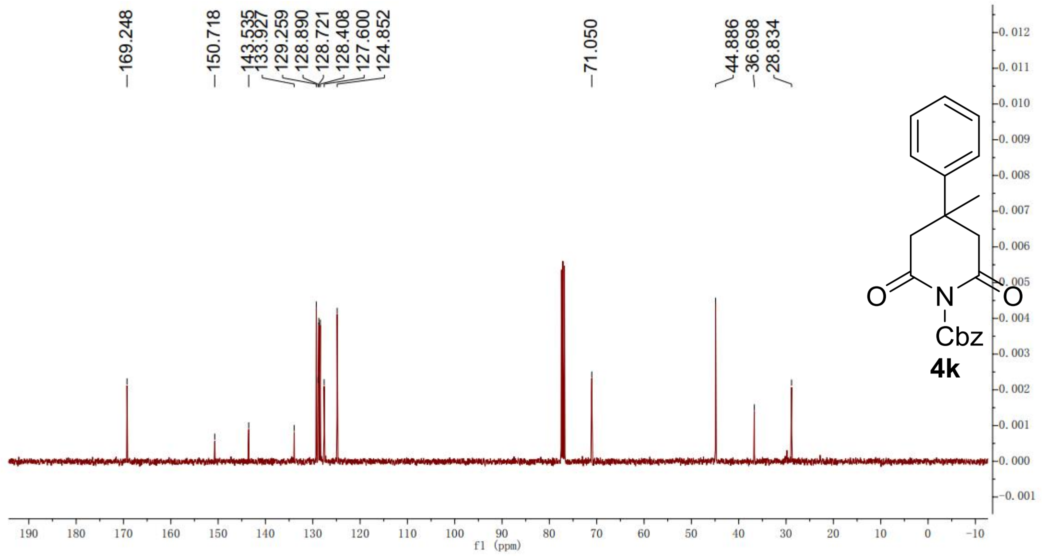

Supplementary Fig. 54. ^{13}C NMR Spectrum of **4k**

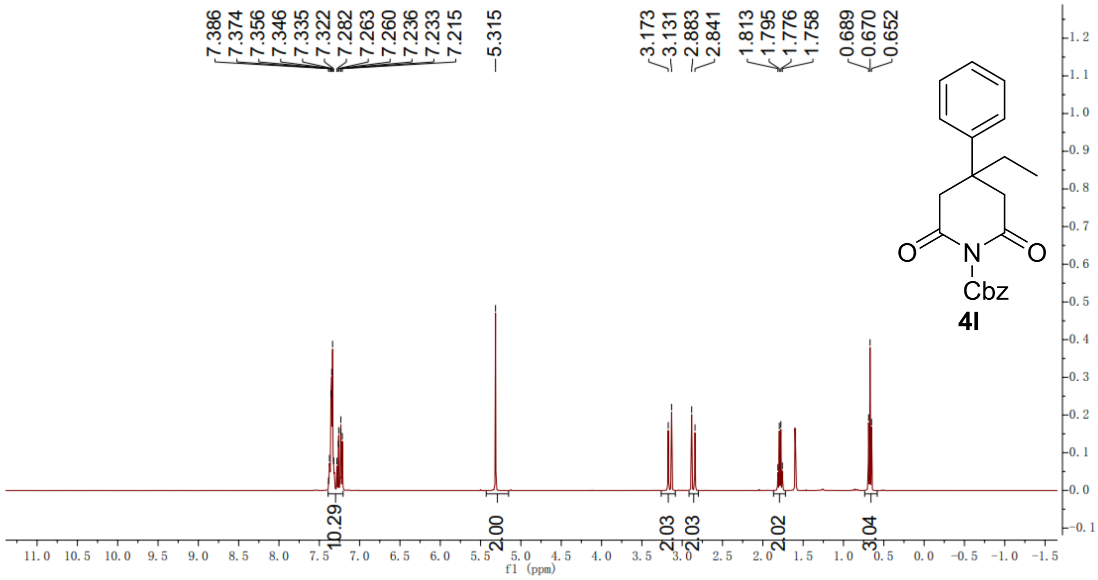

Supplementary Fig. 55. ^{1}H NMR Spectrum of **4l**

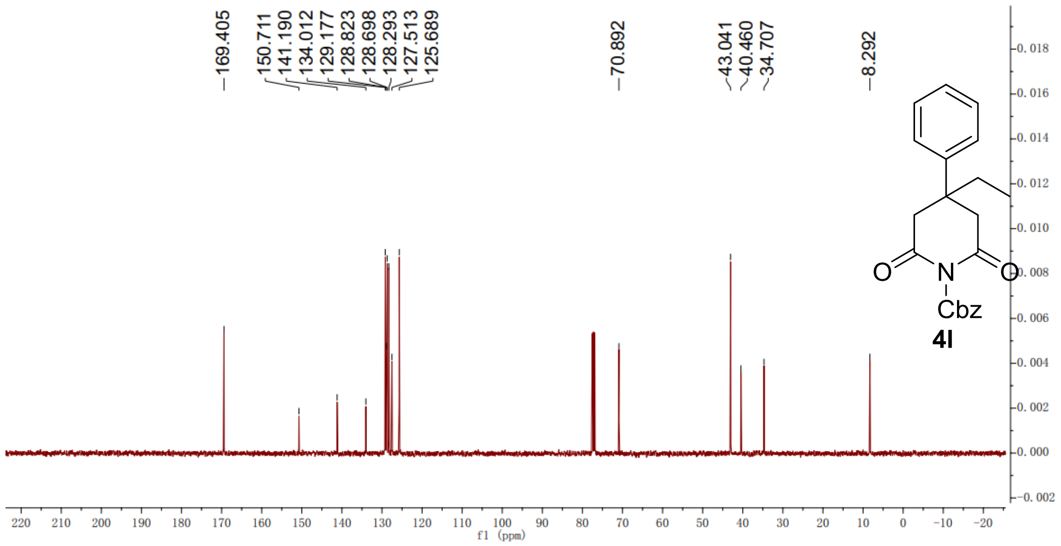

Supplementary Fig. 56. ^{13}C NMR Spectrum of **4l**

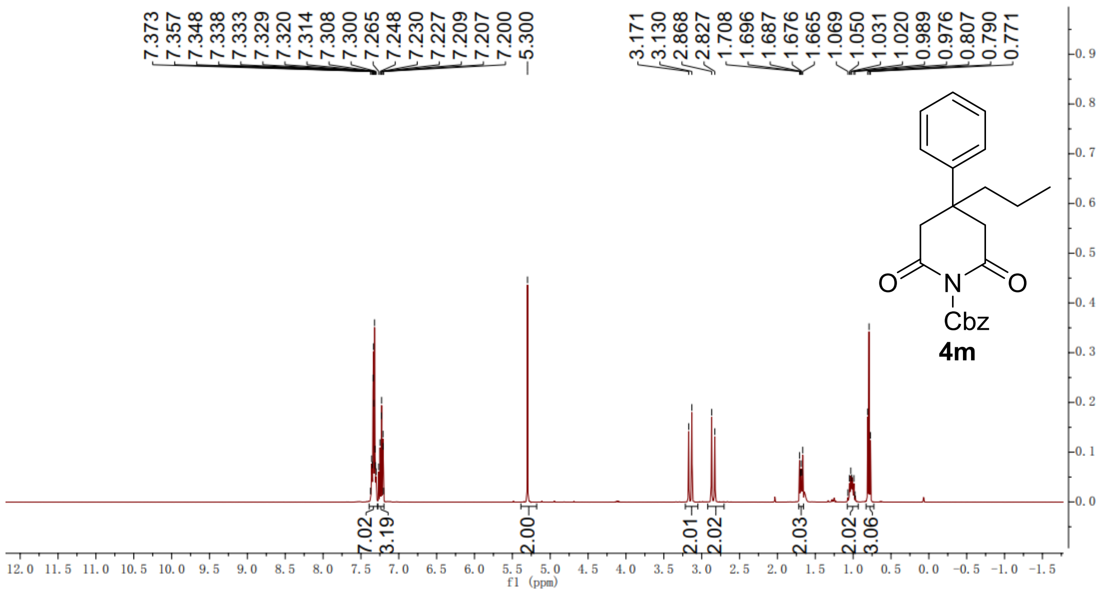

Supplementary Fig. 57. ^{1}H NMR Spectrum of **4m**

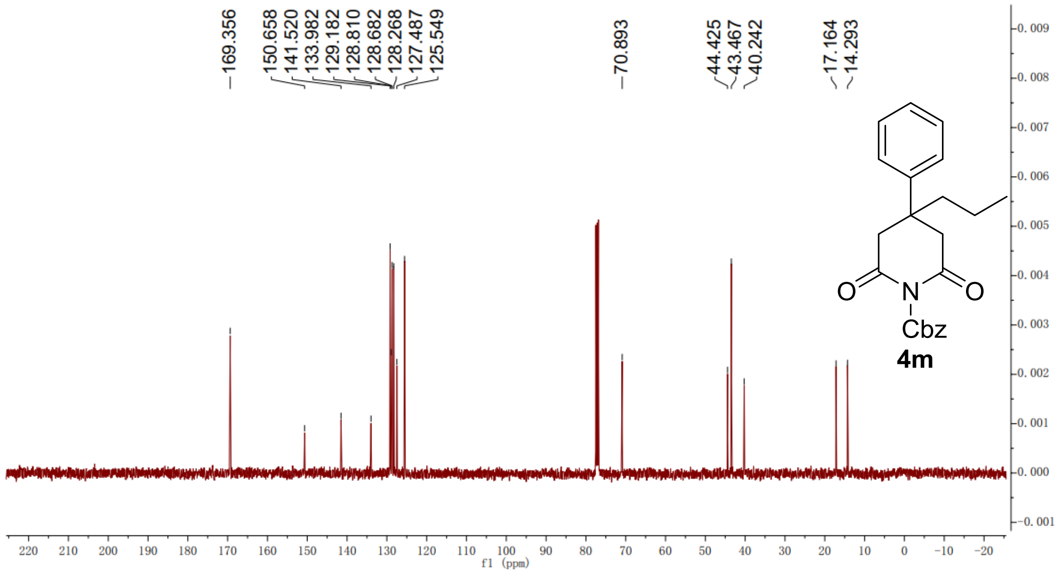

Supplementary Fig. 58. ^{13}C NMR Spectrum of **4m**

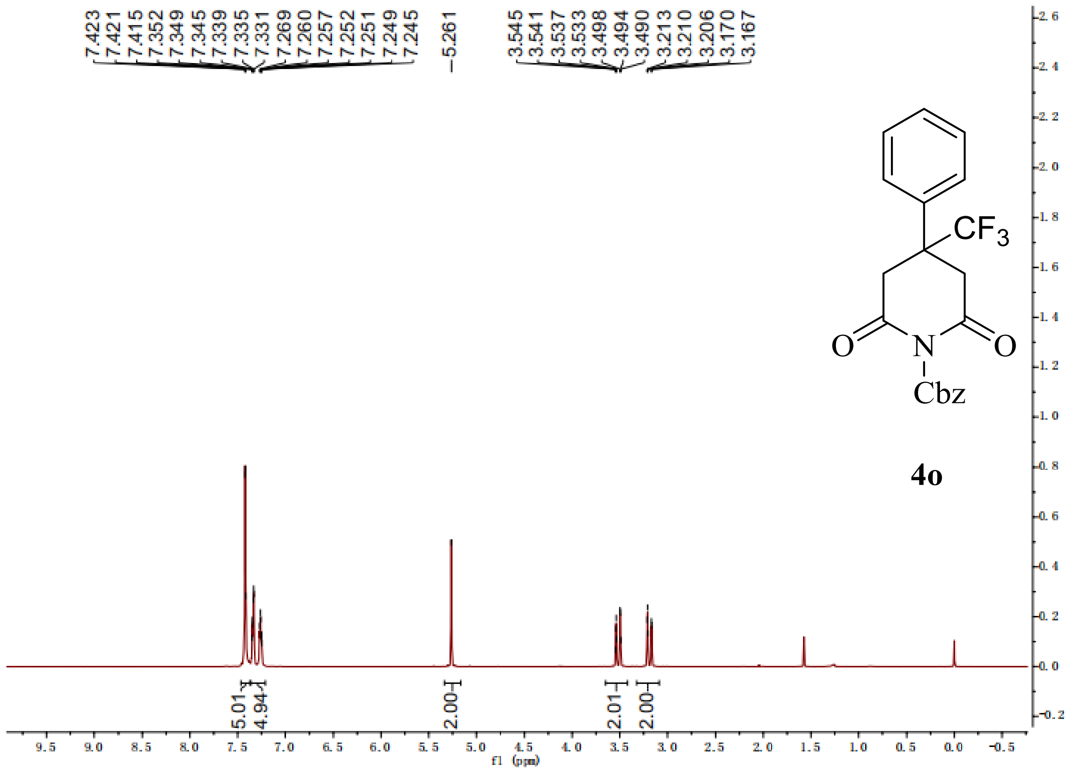

Supplementary Fig. 59. ^{1}H NMR Spectrum of **4o**

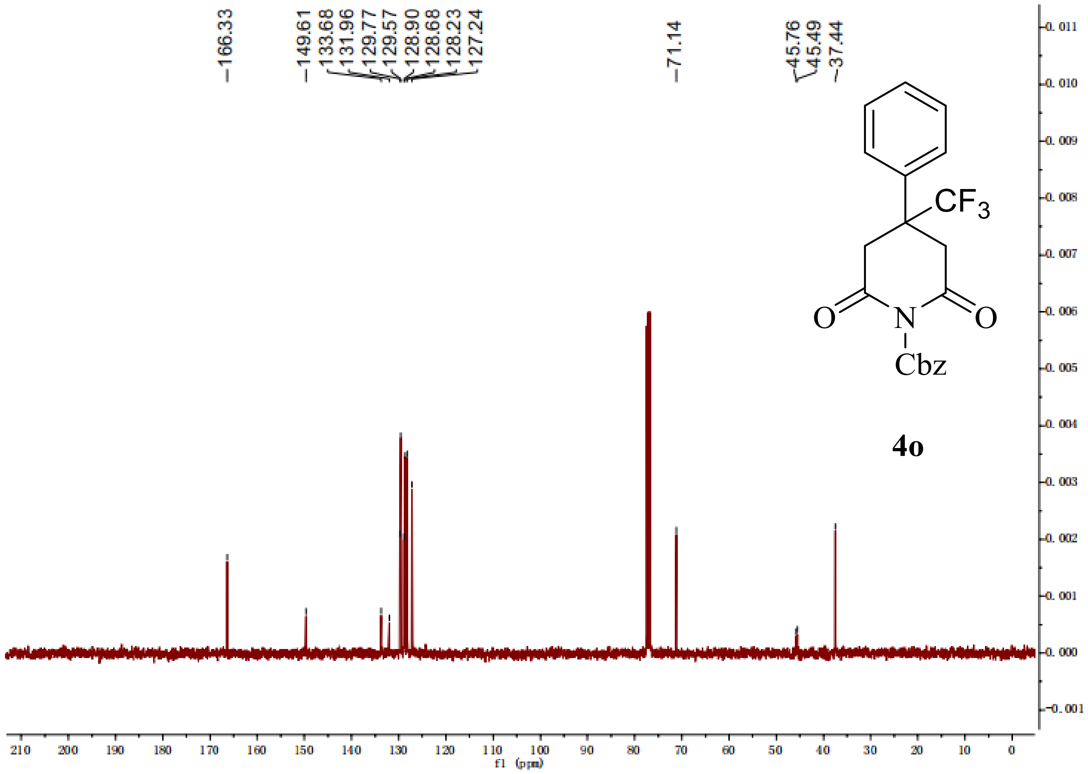

Supplementary Fig. 60. ^{13}C NMR Spectrum of **4o**

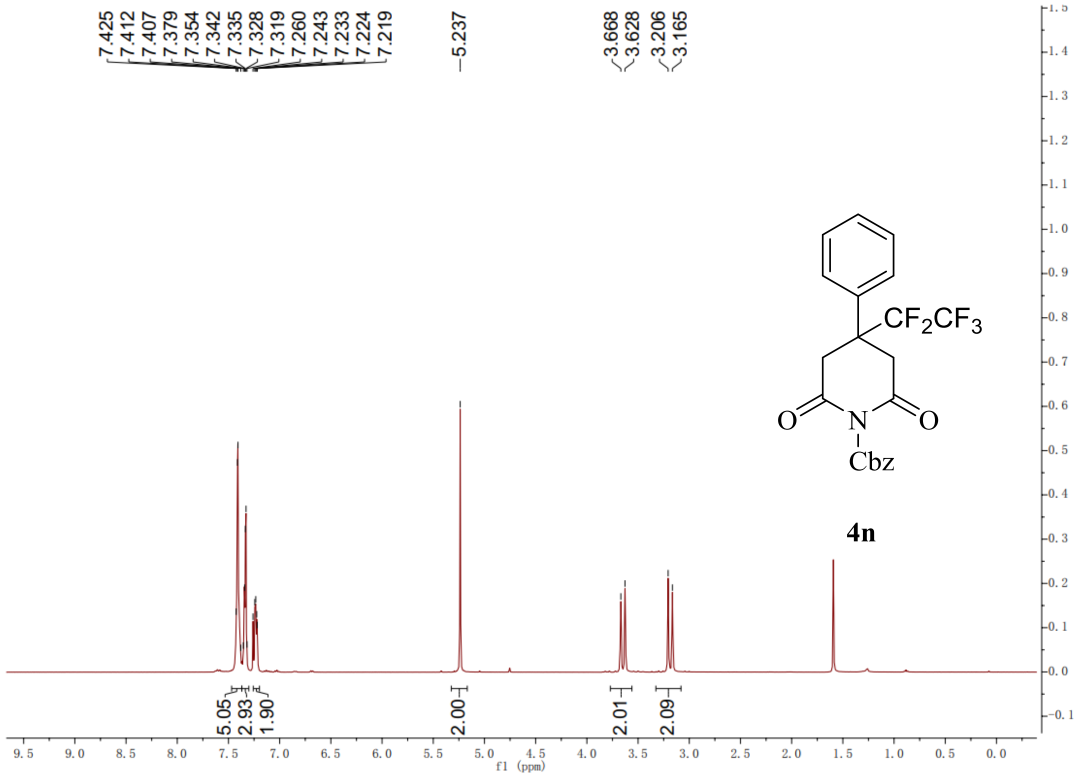

Supplementary Fig. 61. ^{1}H NMR Spectrum of **4n**

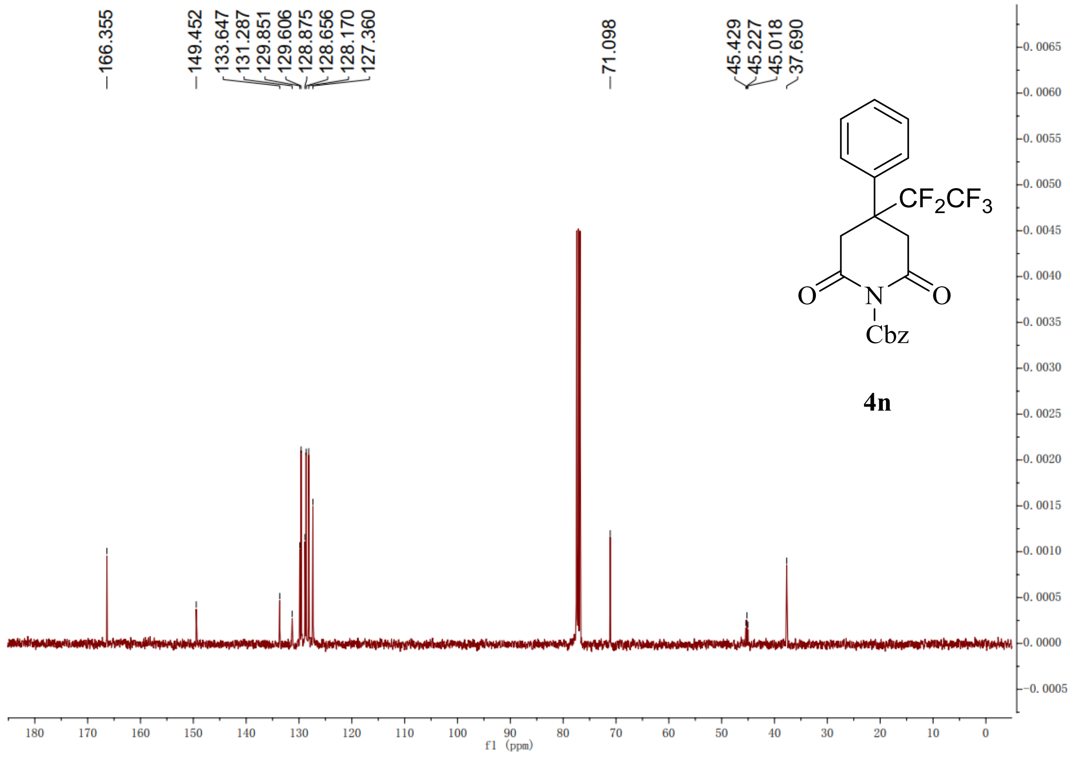

Supplementary Fig. 62. ^{13}C NMR Spectrum of **4n**

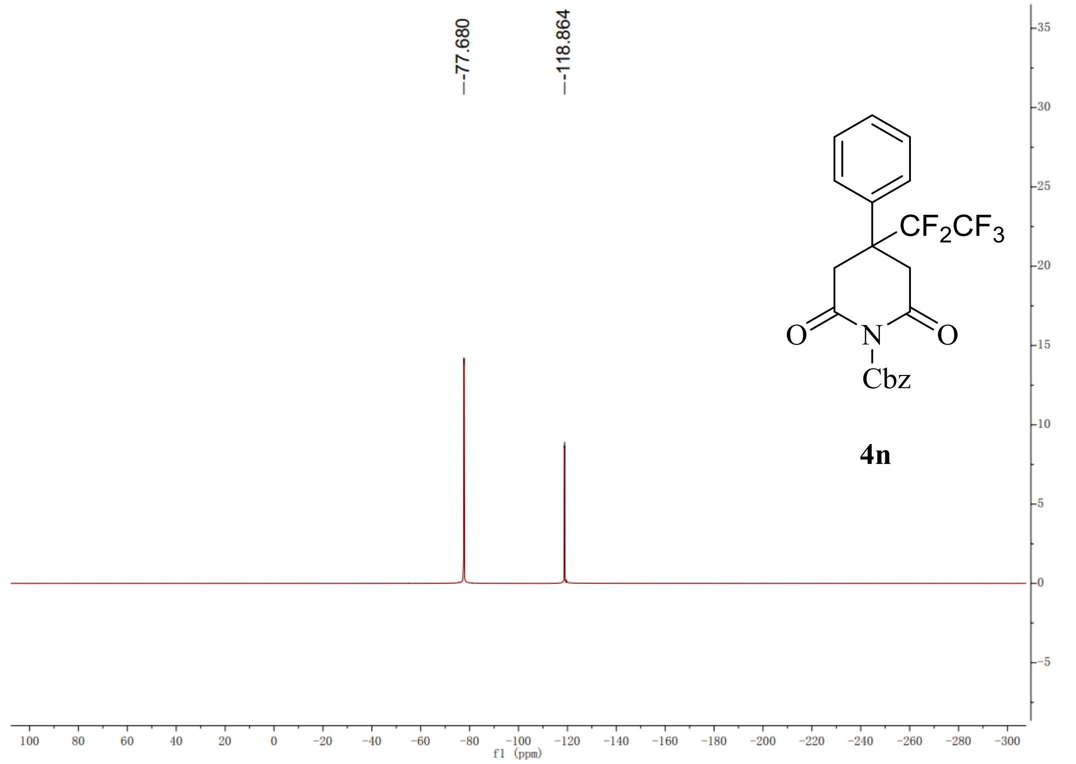

Supplementary Fig. 63. ^{19}F NMR Spectrum of **4n**

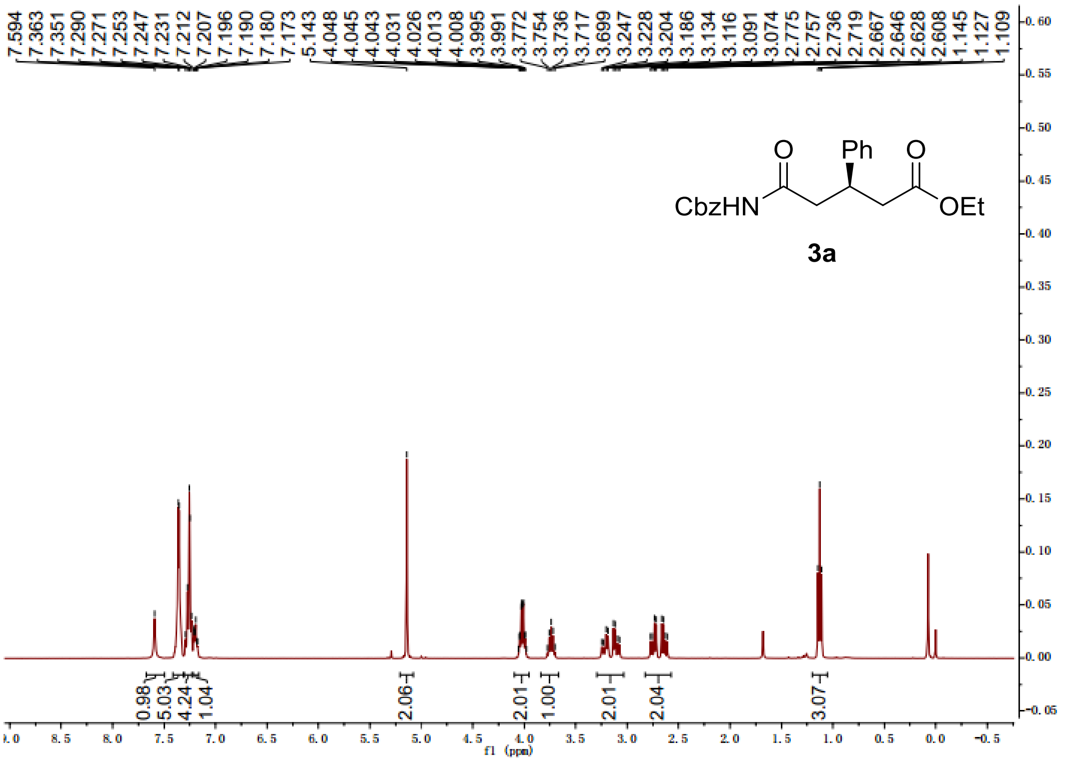

Supplementary Fig. 64. ^{1}H NMR Spectrum of **3a**

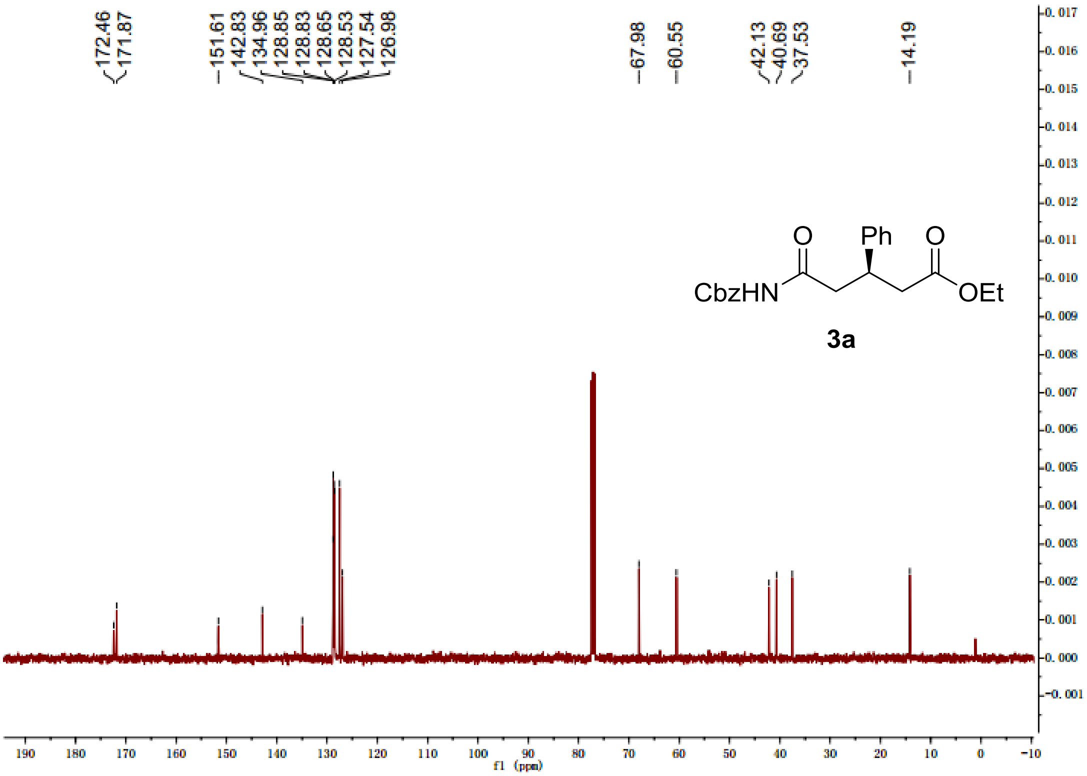

Supplementary Fig. 65. ^{13}C NMR Spectrum of **3a**

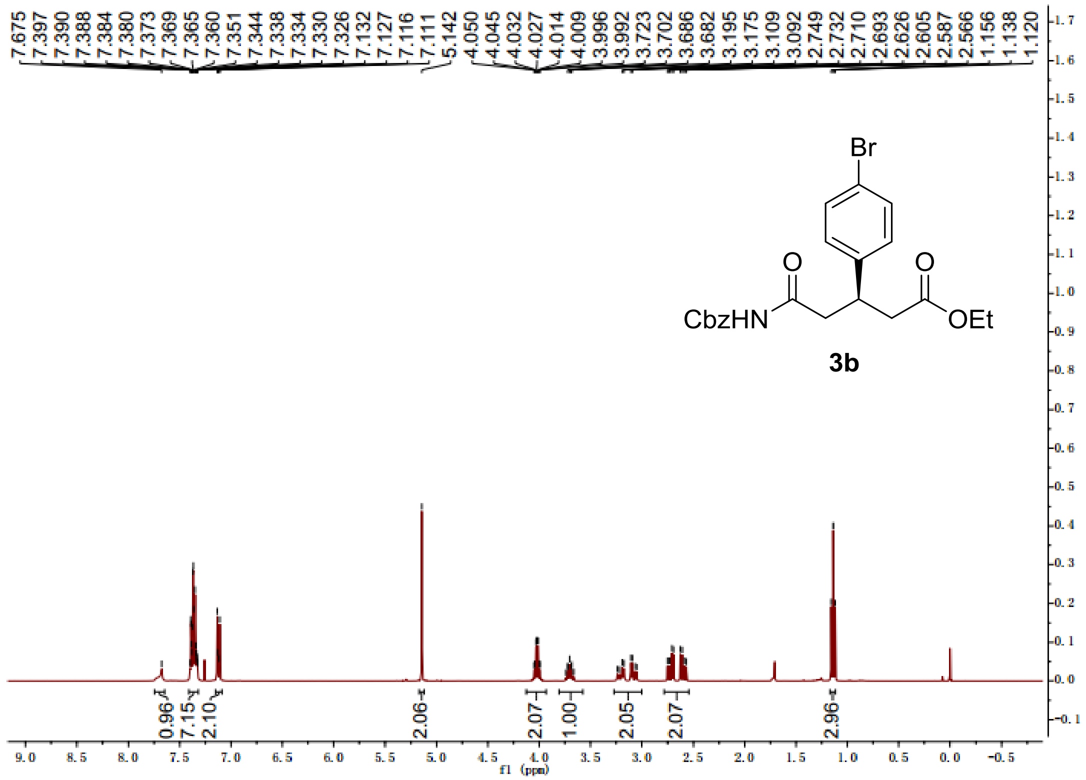

Supplementary Fig. 66. ^{1}H NMR Spectrum of **3b**

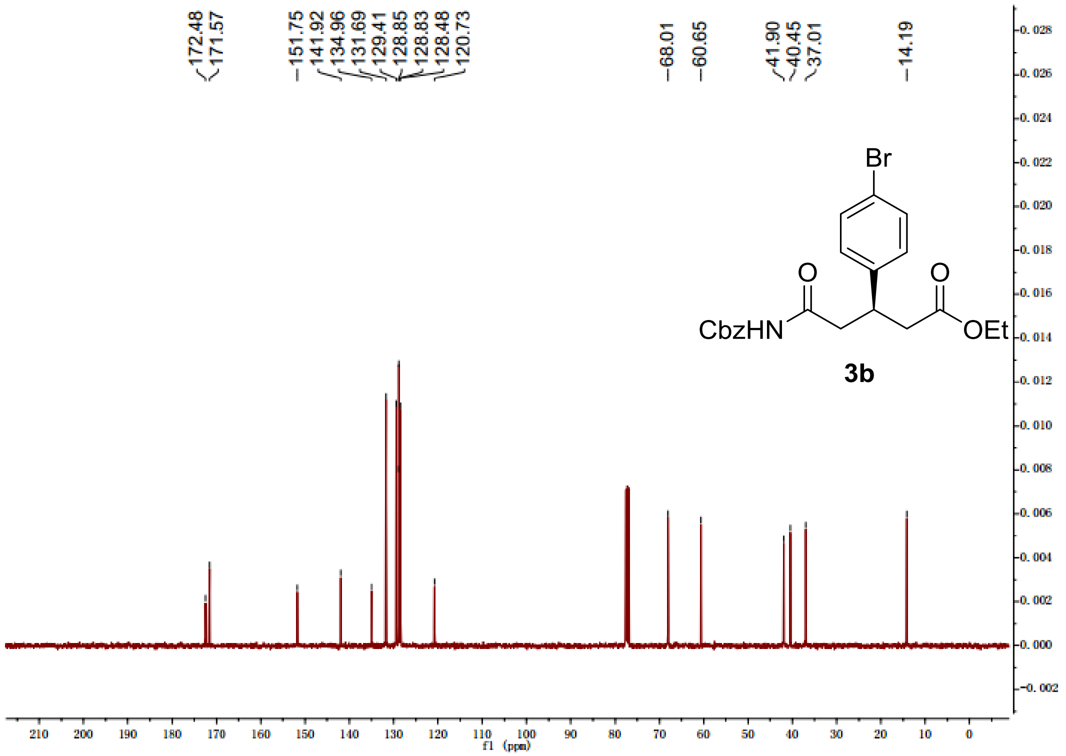

Supplementary Fig. 67. ^{13}C NMR Spectrum of **3b**

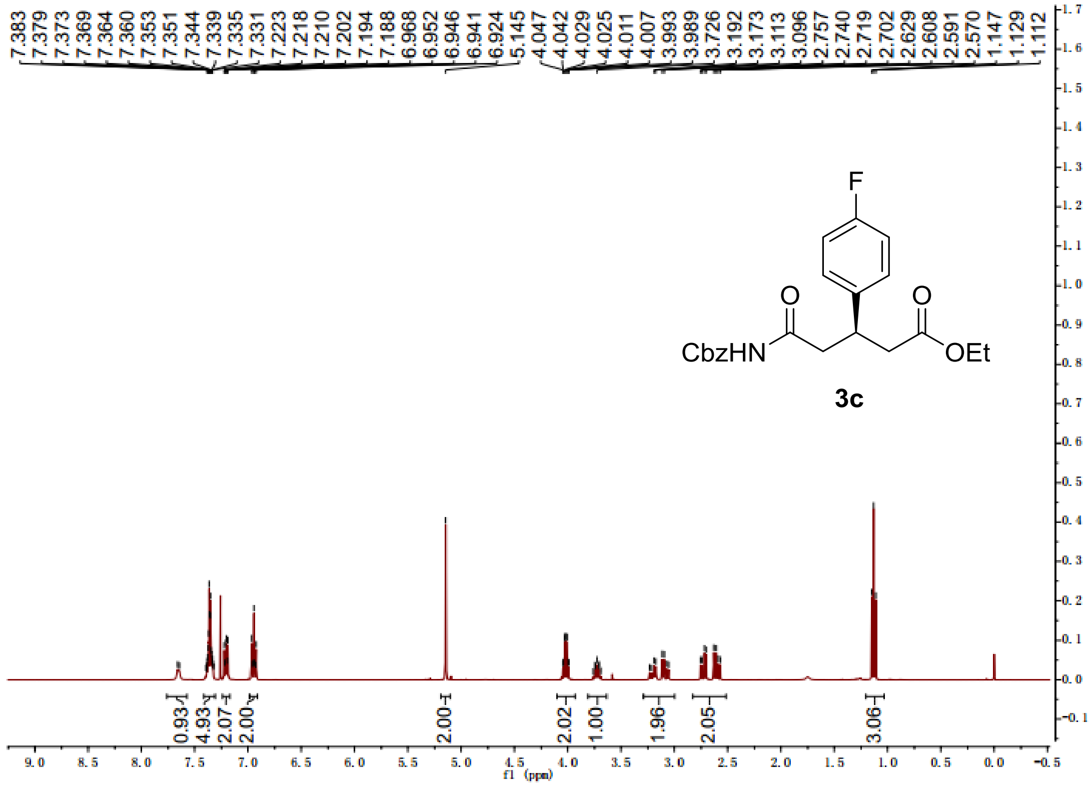

Supplementary Fig. 68. ^{1}H NMR Spectrum of **3c**

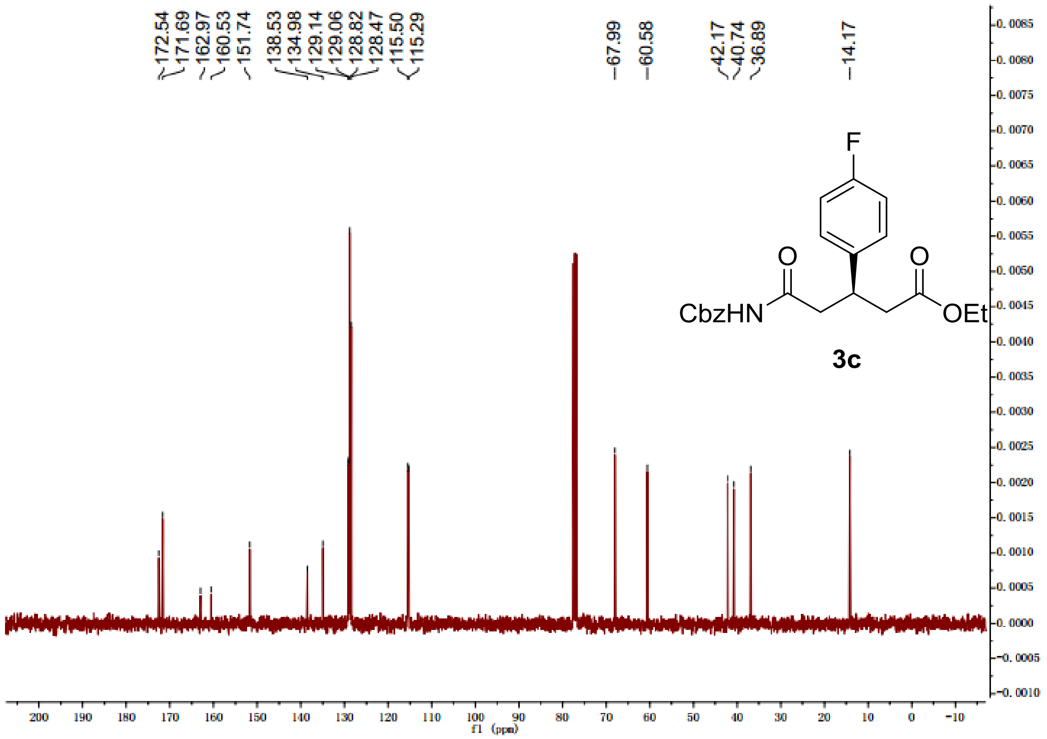

Supplementary Fig. 69. ^{13}C NMR Spectrum of **3c**

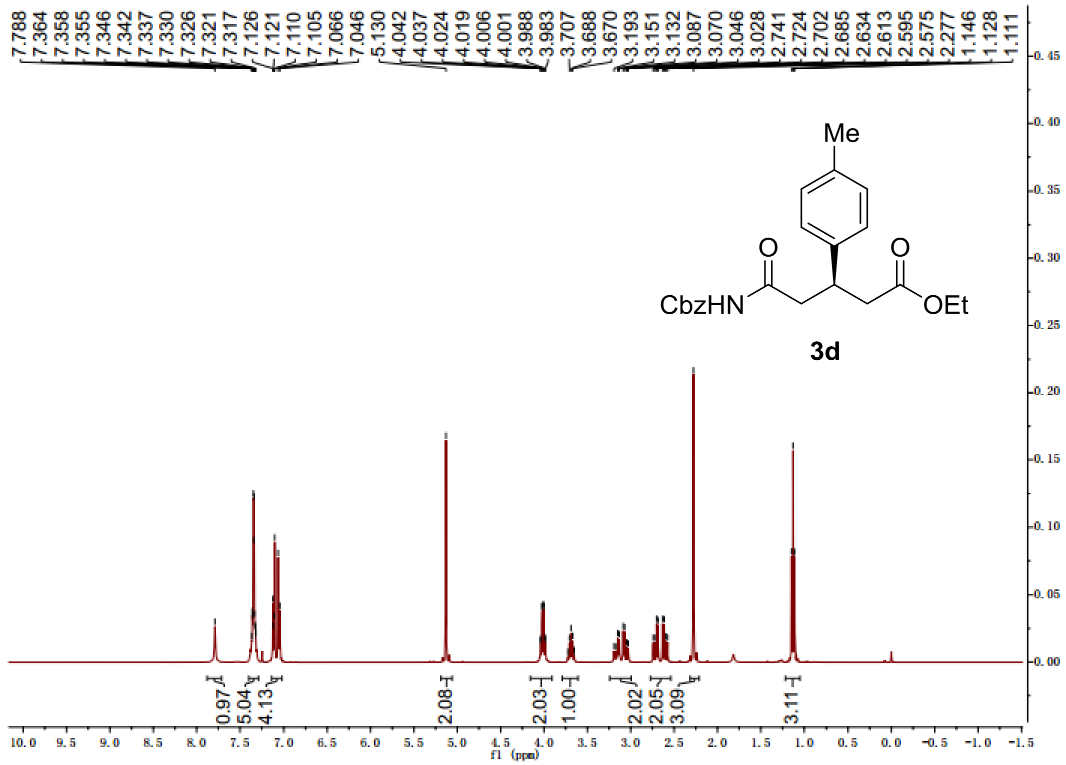

Supplementary Fig. 70. ^{1}H NMR Spectrum of **3d**

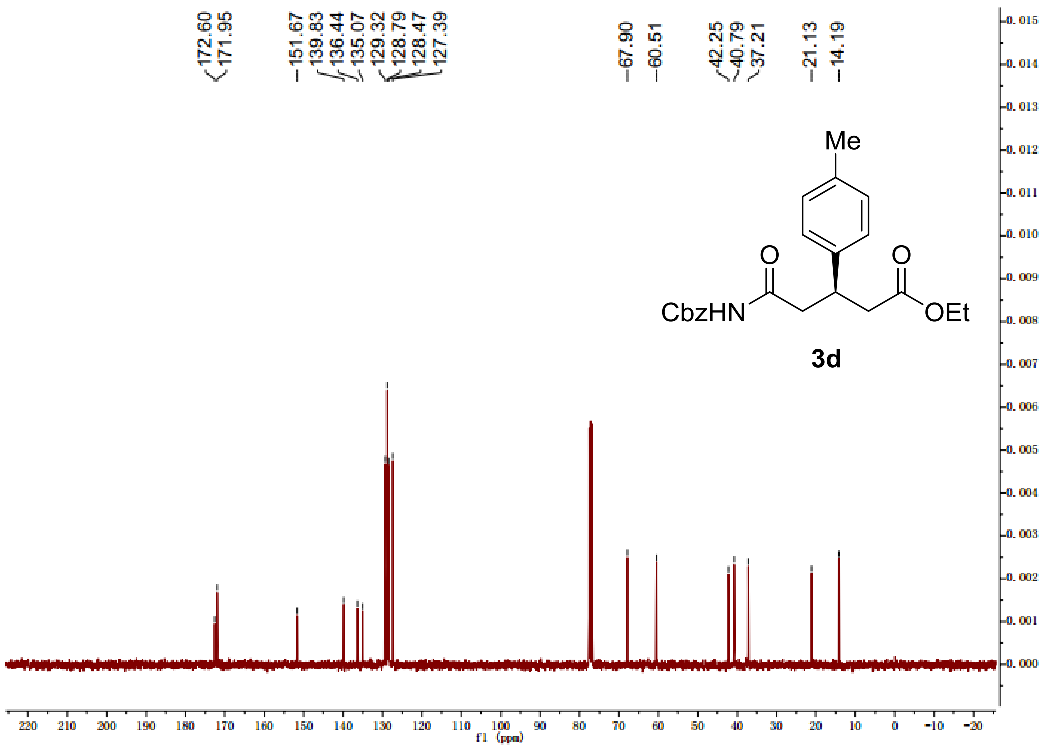

Supplementary Fig. 71. ^{13}C NMR Spectrum of **3d**

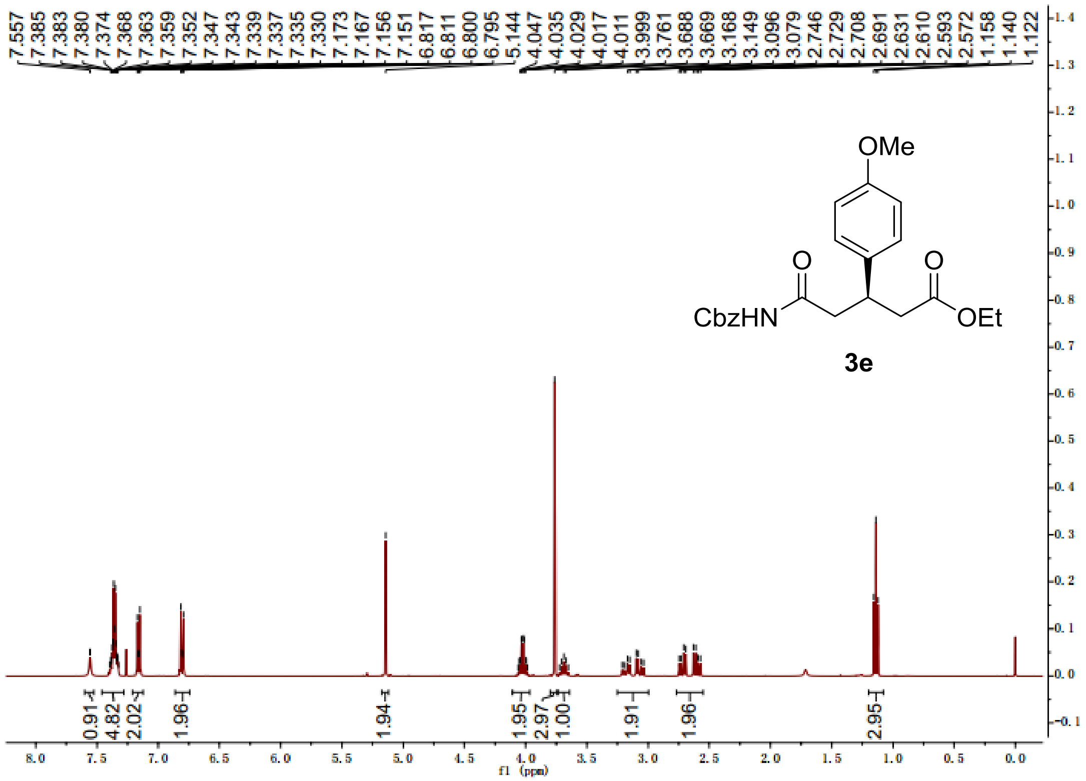

Supplementary Fig. 72. ^{1}H NMR Spectrum of **3e**

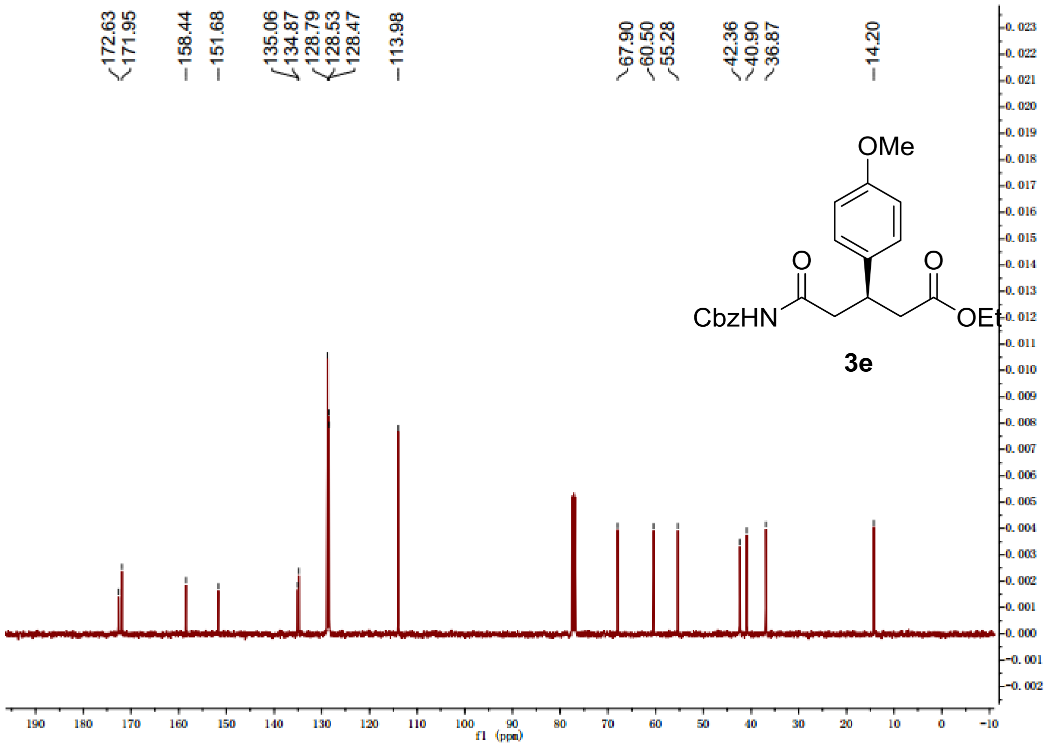

Supplementary Fig. 73. ^{13}C NMR Spectrum of **3e**

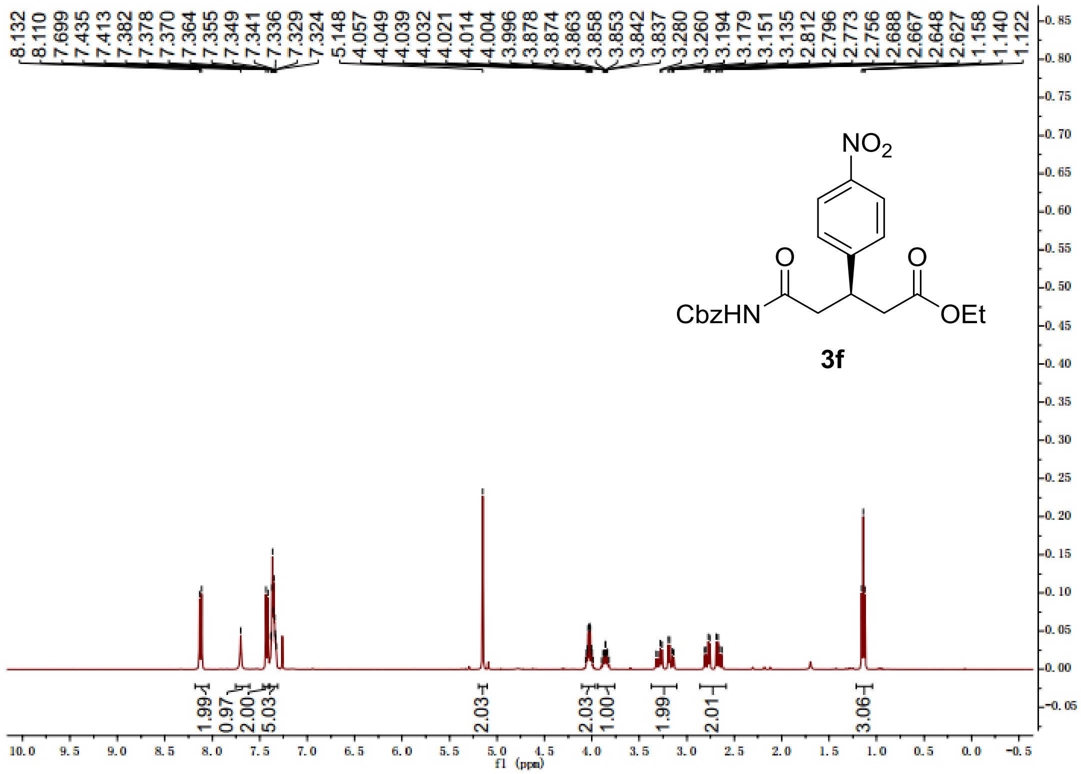

Supplementary Fig. 74. ^{1}H NMR Spectrum of **3f**

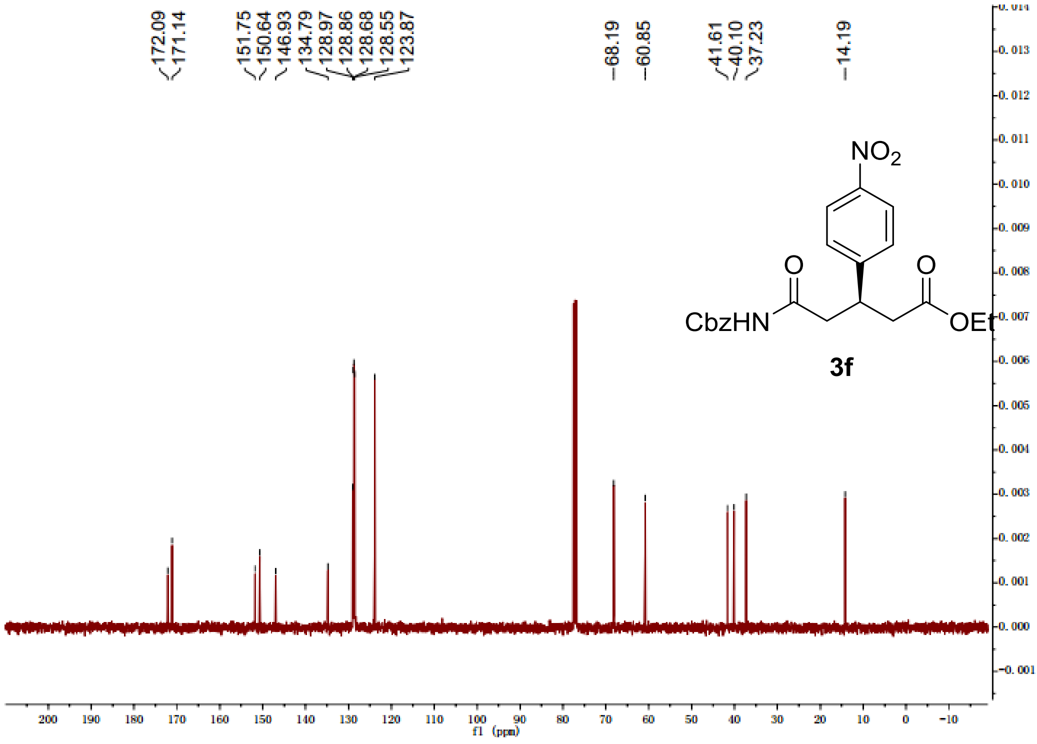

Supplementary Fig. 75. ^{13}C NMR Spectrum of **3f**

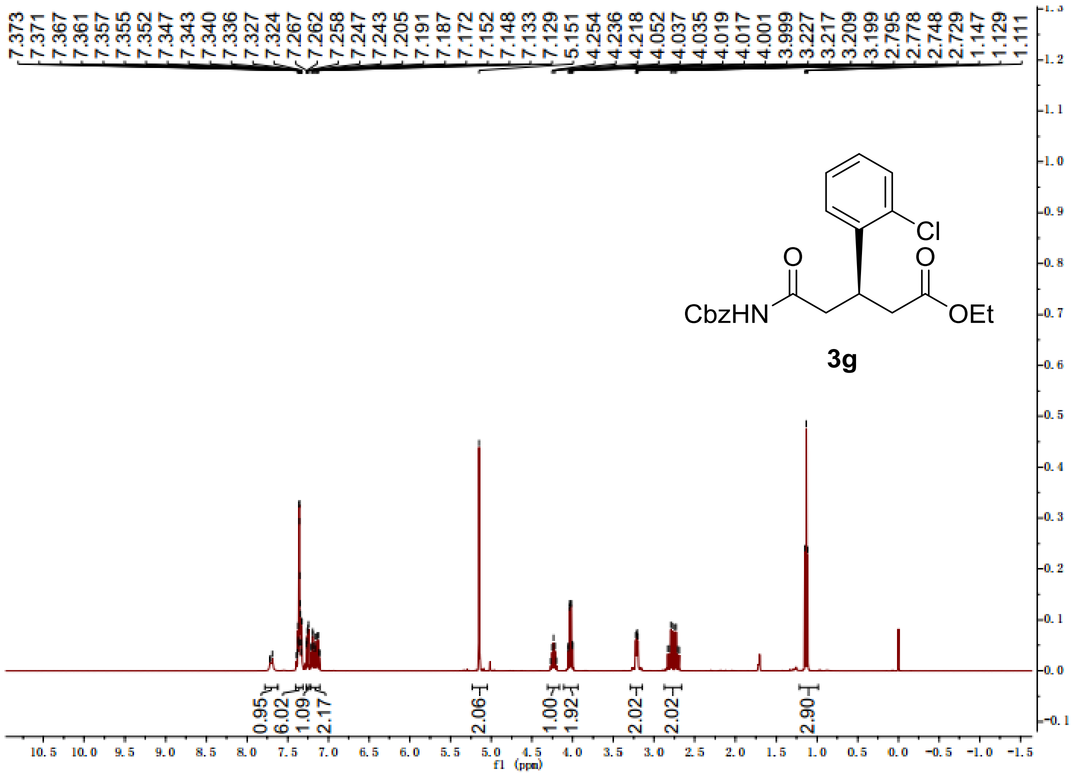

Supplementary Fig. 76. ^{1}H NMR Spectrum of **3g**

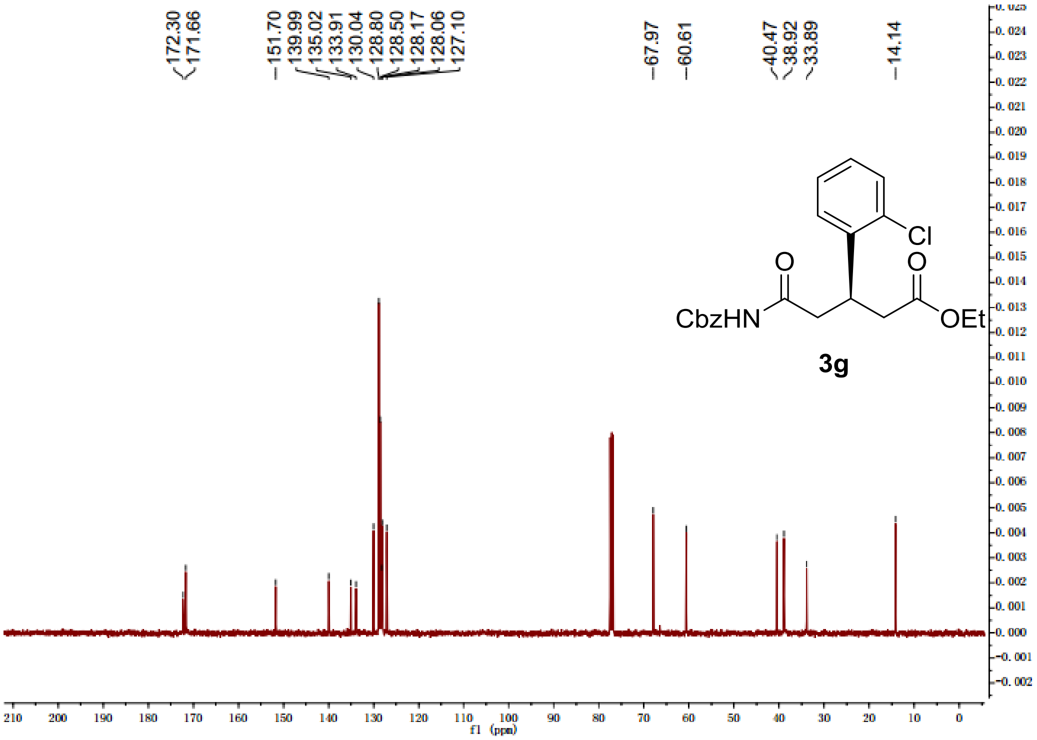

Supplementary Fig. 77. ^{13}C NMR Spectrum of **3g**

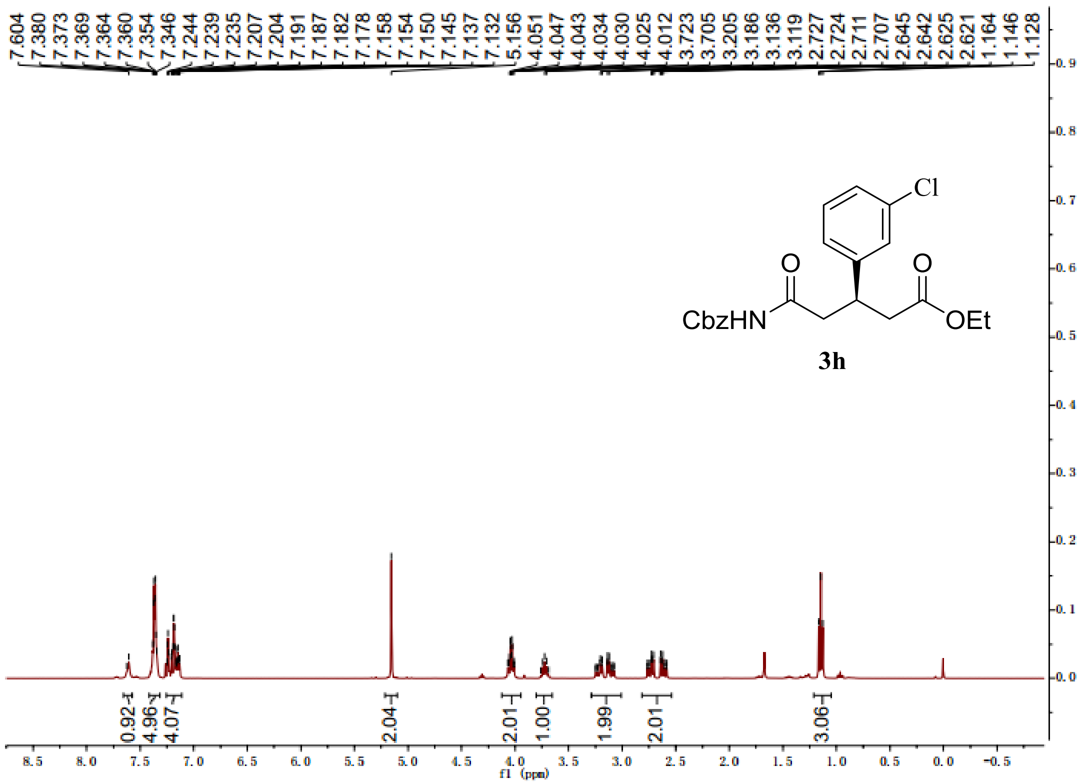

Supplementary Fig. 78. ^{1}H NMR Spectrum of **3h**

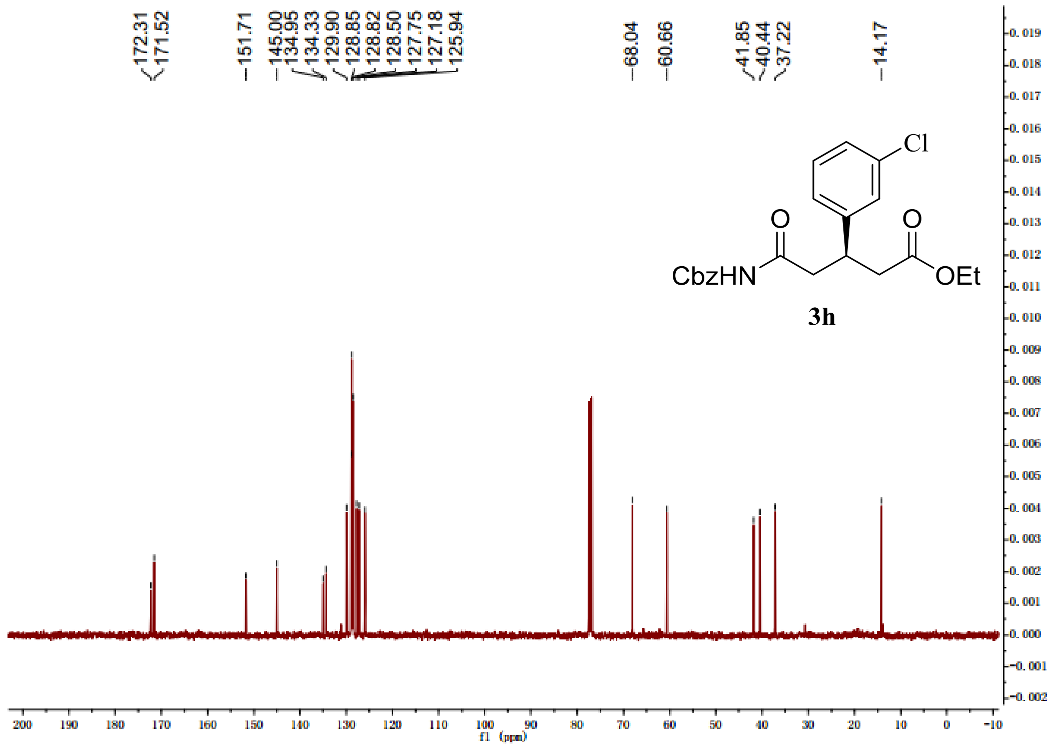

Supplementary Fig. 79. ^{13}C NMR Spectrum of **3h**

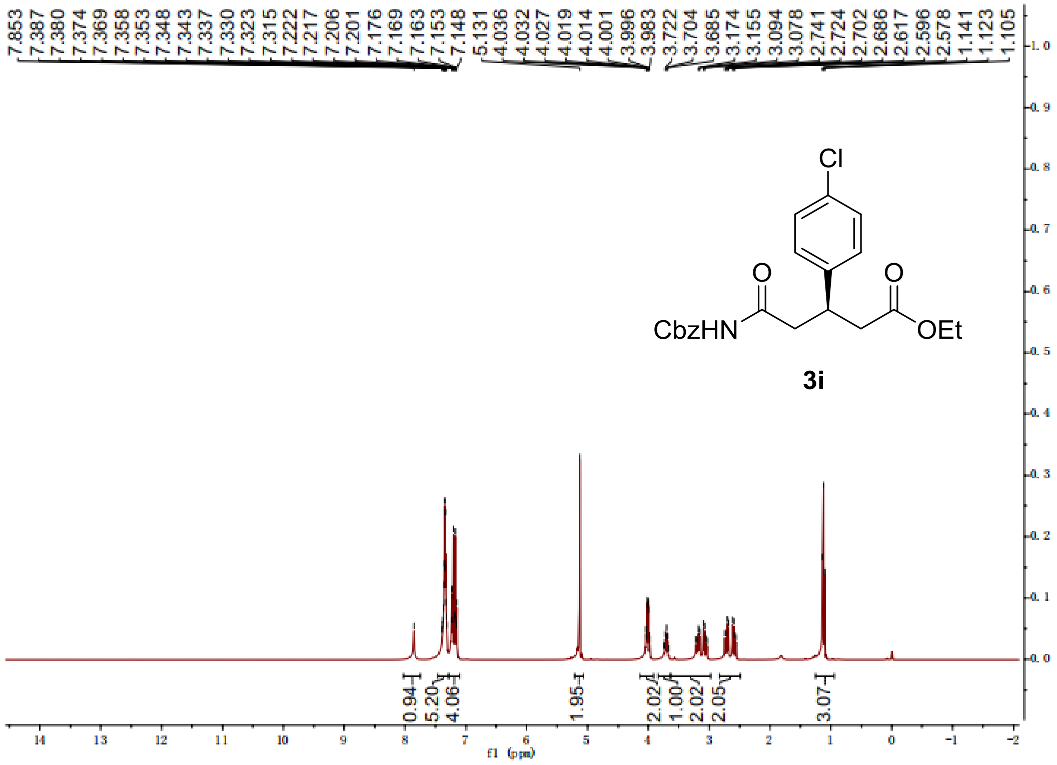

Supplementary Fig. 80. ^{1}H NMR Spectrum of **3i**

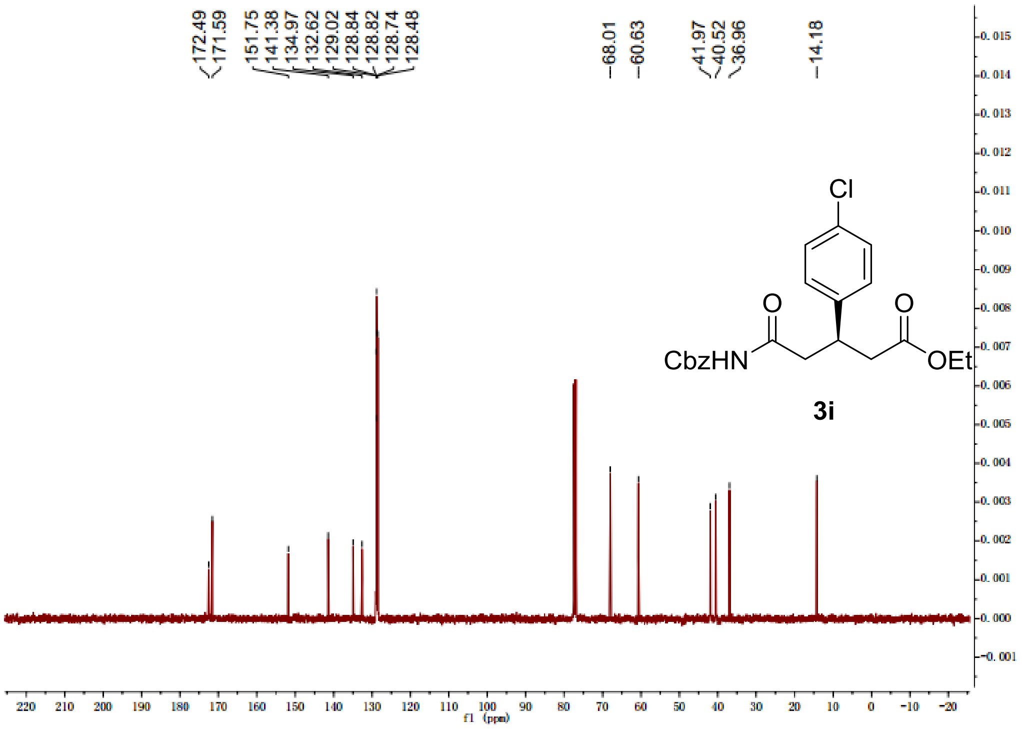

Supplementary Fig. 81. ^{13}C NMR Spectrum of **3i**

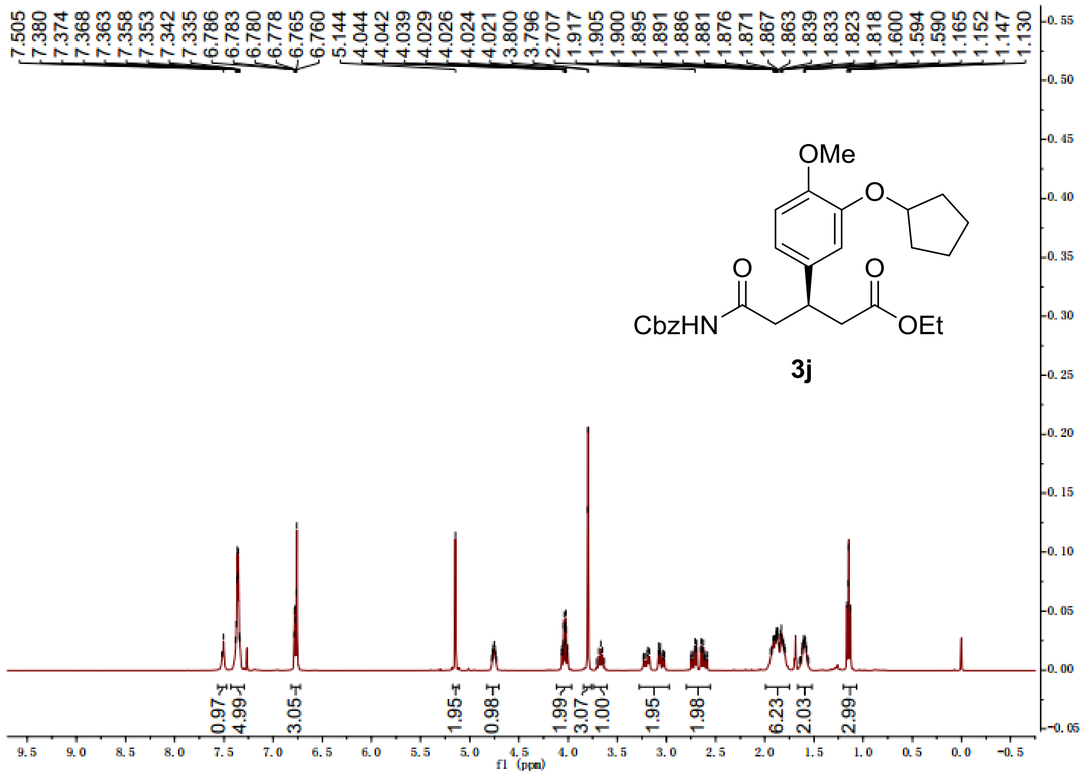

Supplementary Fig. 82. ^{1}H NMR Spectrum of **3j**

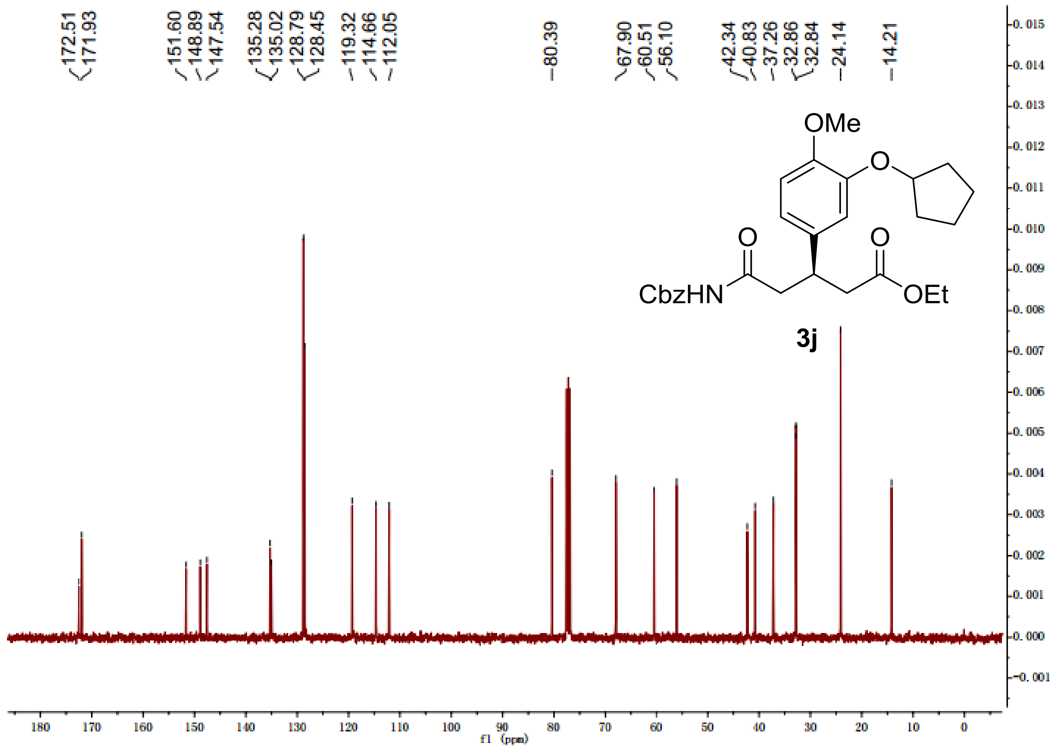

Supplementary Fig. 83. ^{13}C NMR Spectrum of **3j**

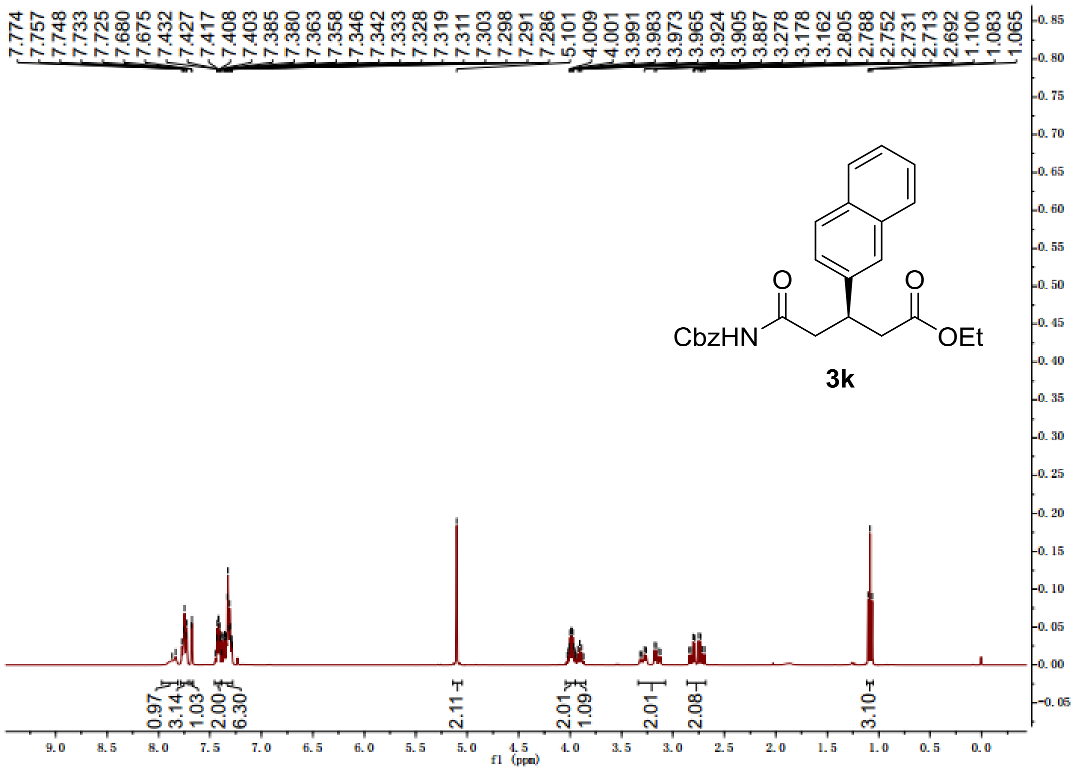

Supplementary Fig. 84. ^{1}H NMR Spectrum of **3k**

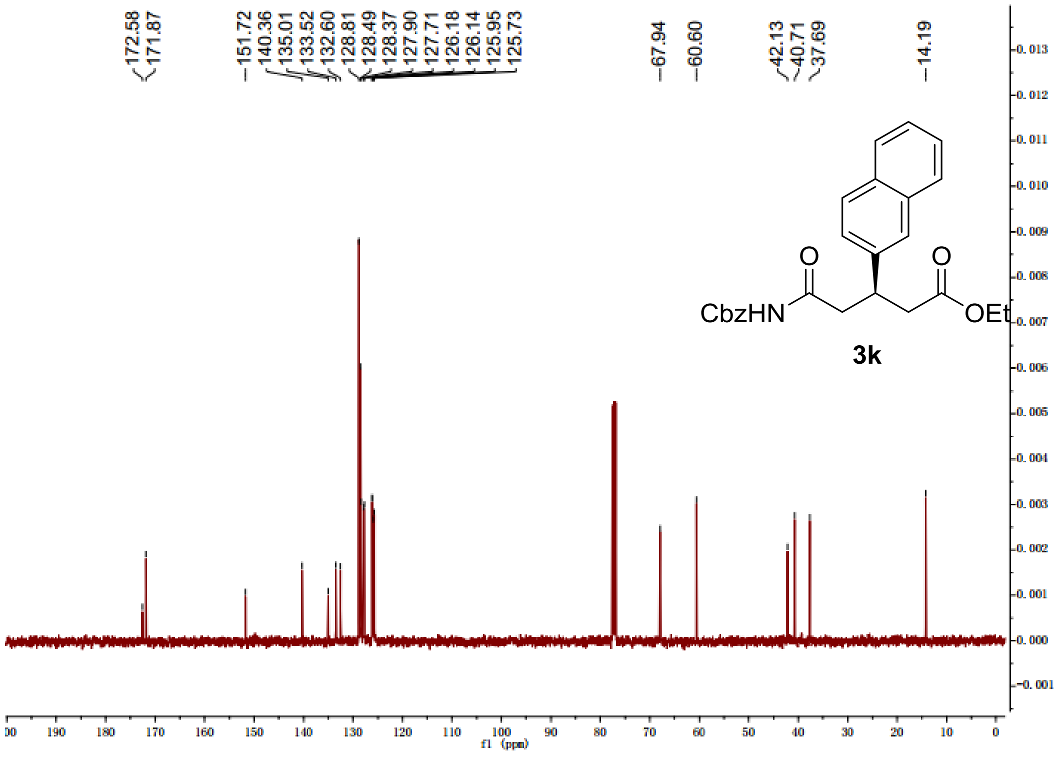

Supplementary Fig. 85. ^{13}C NMR Spectrum of **3k**

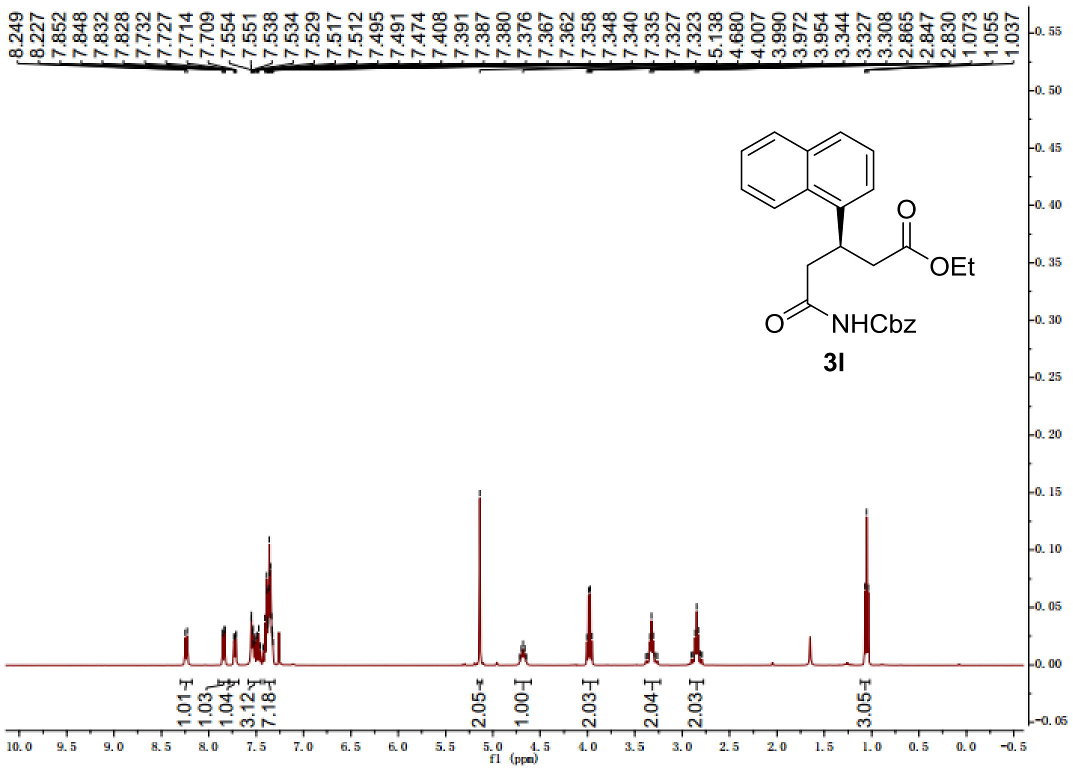

Supplementary Fig. 86. ^{1}H NMR Spectrum of **3l**

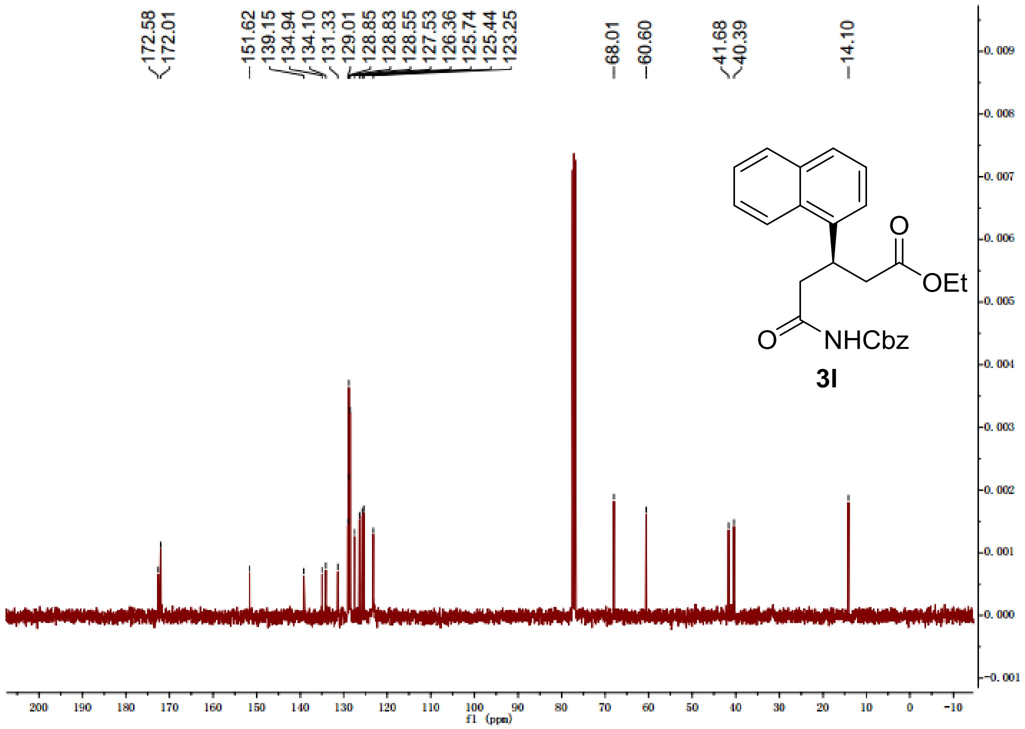

Supplementary Fig. 87. ^{13}C NMR Spectrum of **3l**

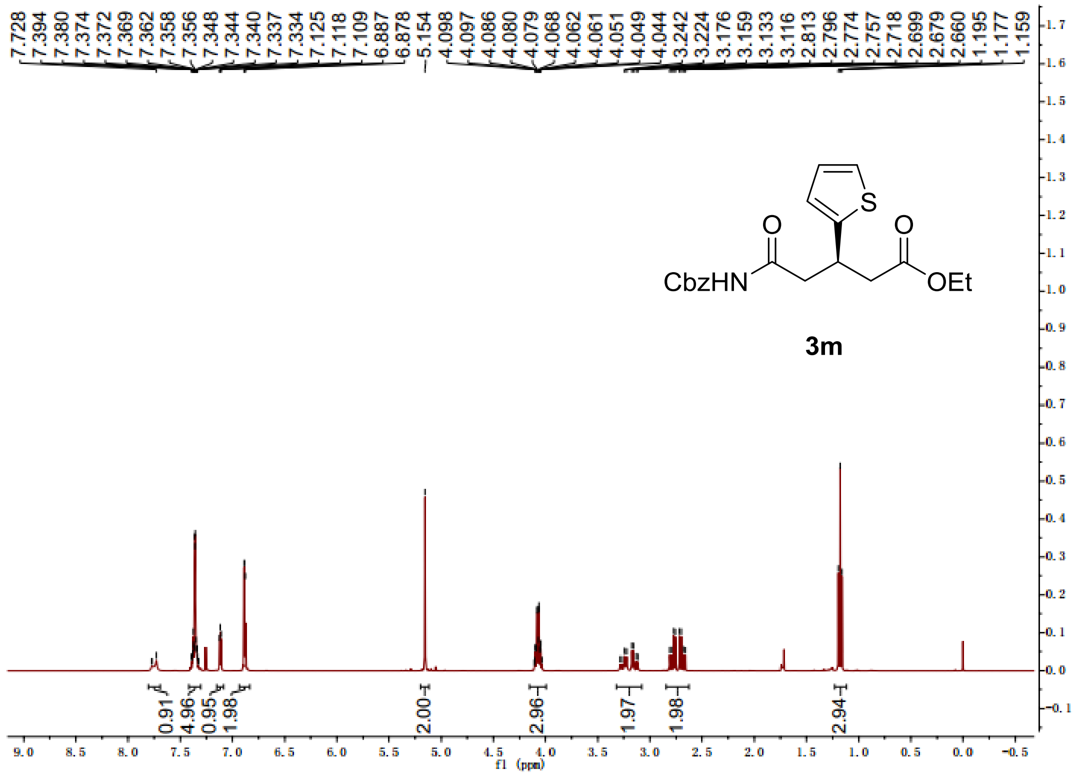

Supplementary Fig. 88. ^{1}H NMR Spectrum of **3m**

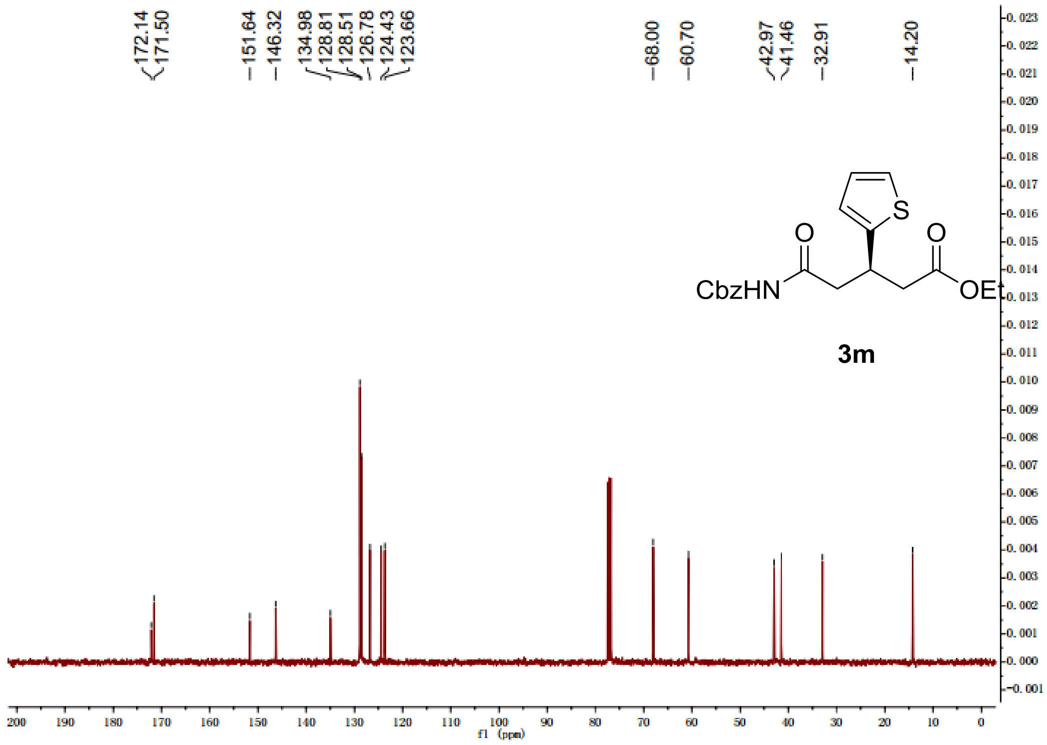

Supplementary Fig. 89. ^{13}C NMR Spectrum of **3m**

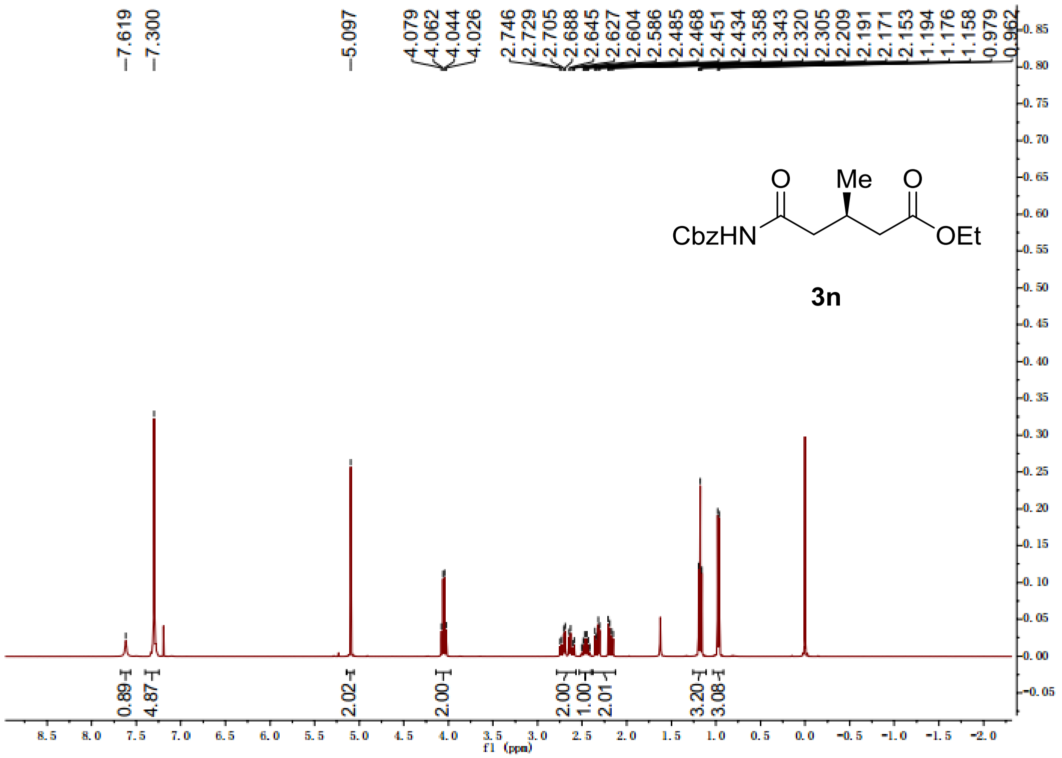

Supplementary Fig. 90. ^{1}H NMR Spectrum of **3n**

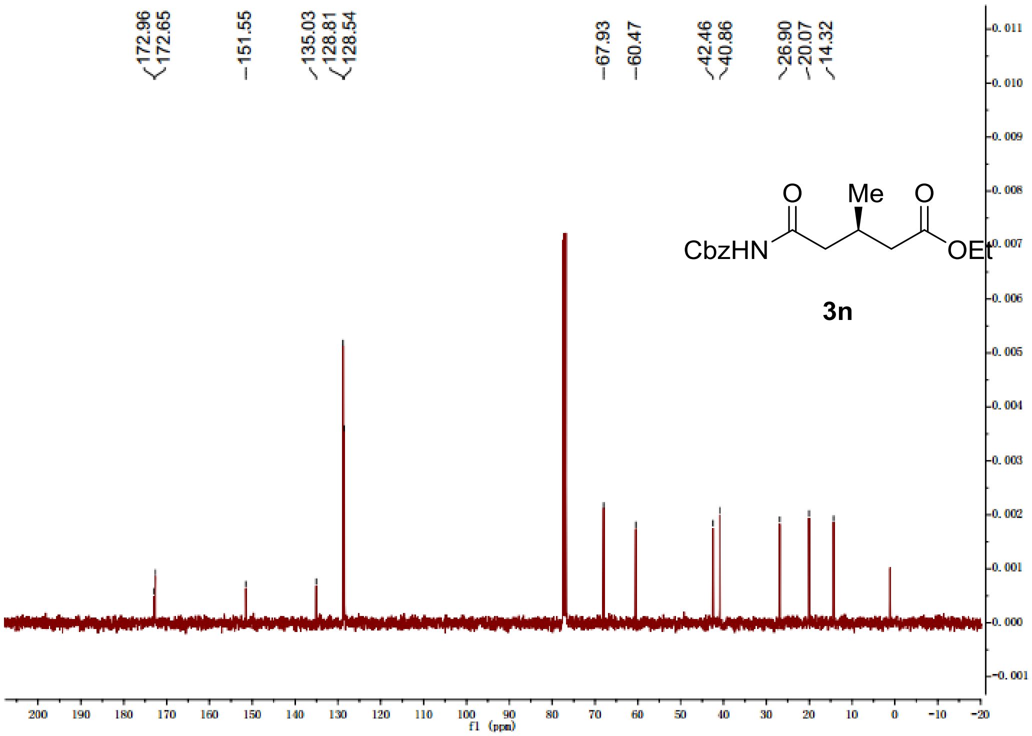

Supplementary Fig. 91. ^{13}C NMR Spectrum of **3n**

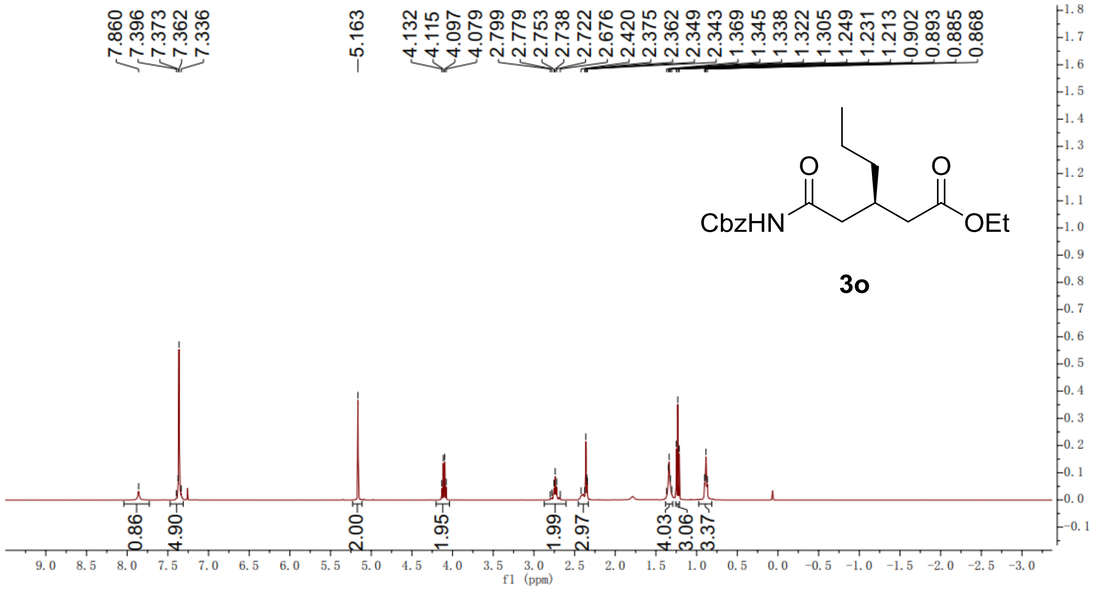

Supplementary Fig. 92. ^{1}H NMR Spectrum of **3o**

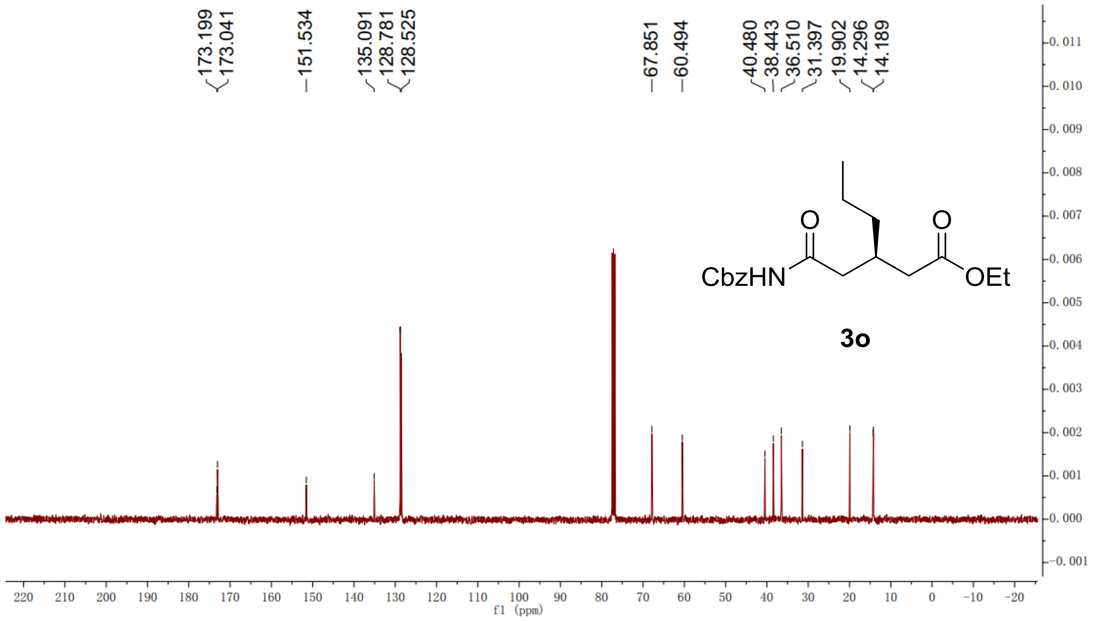

Supplementary Fig. 93. ^{13}C NMR Spectrum of **3o**

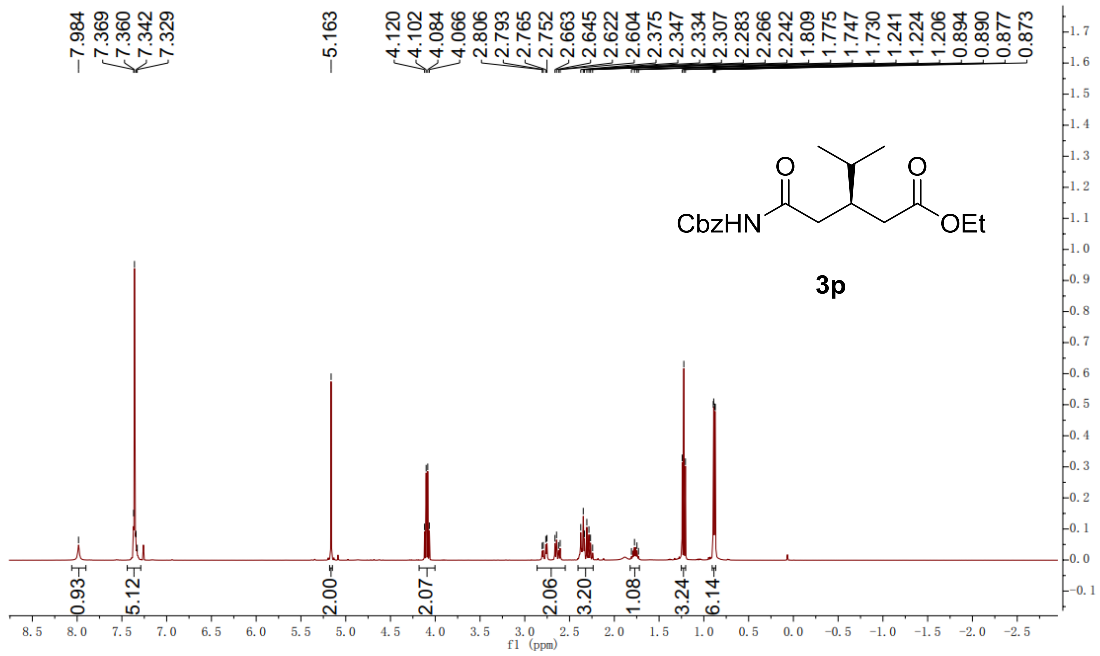

Supplementary Fig. 94. ^{1}H NMR Spectrum of **3p**

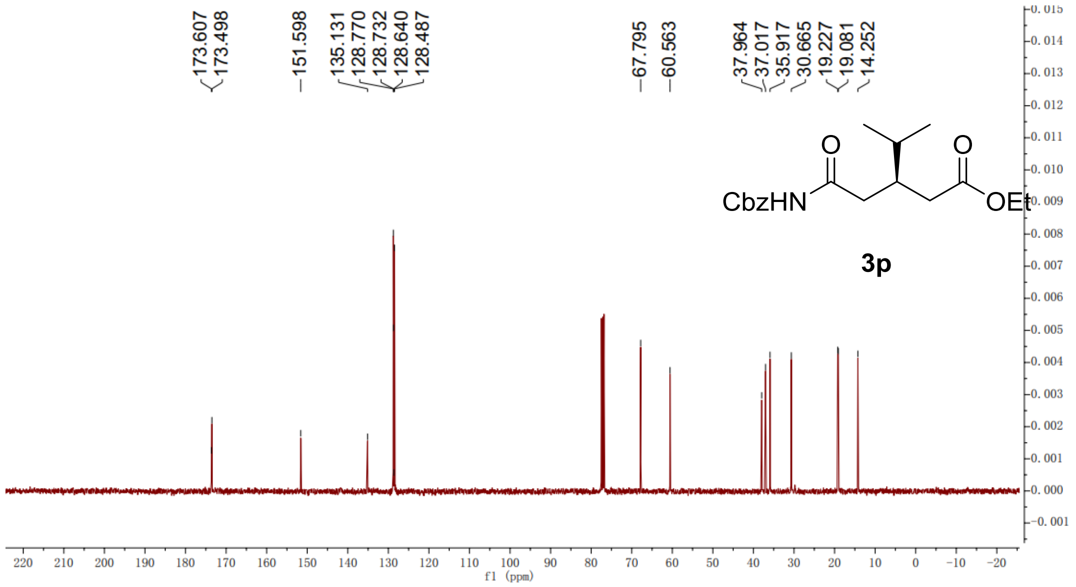

Supplementary Fig. 95. ^{13}C NMR Spectrum of **3p**

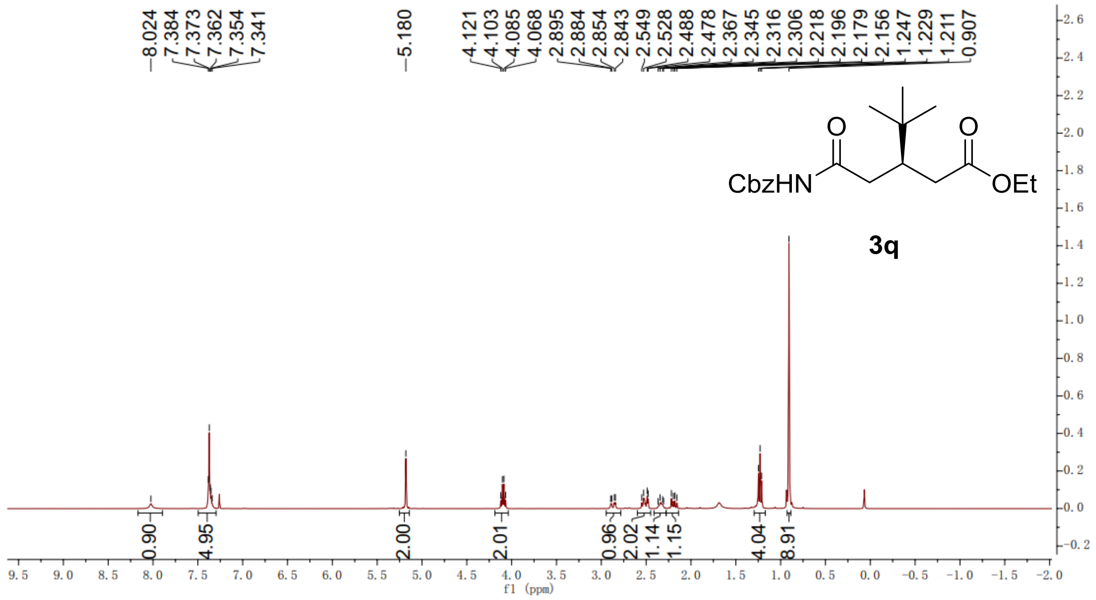

Supplementary Fig. 96. ^{1}H NMR Spectrum of **3q**

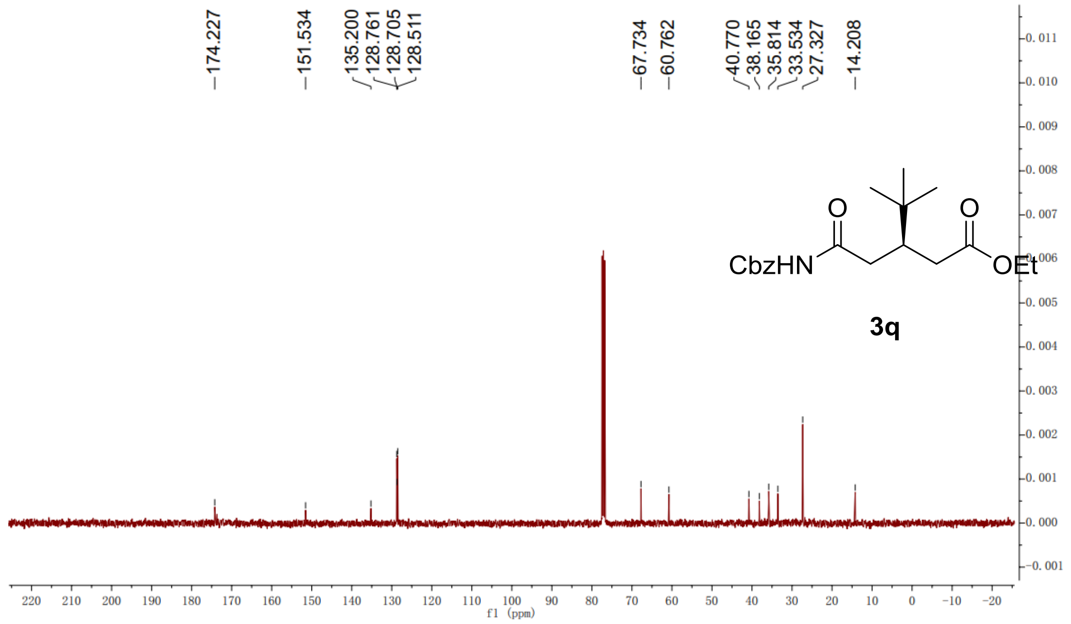

Supplementary Fig. 97. ^{13}C NMR Spectrum of **3q**

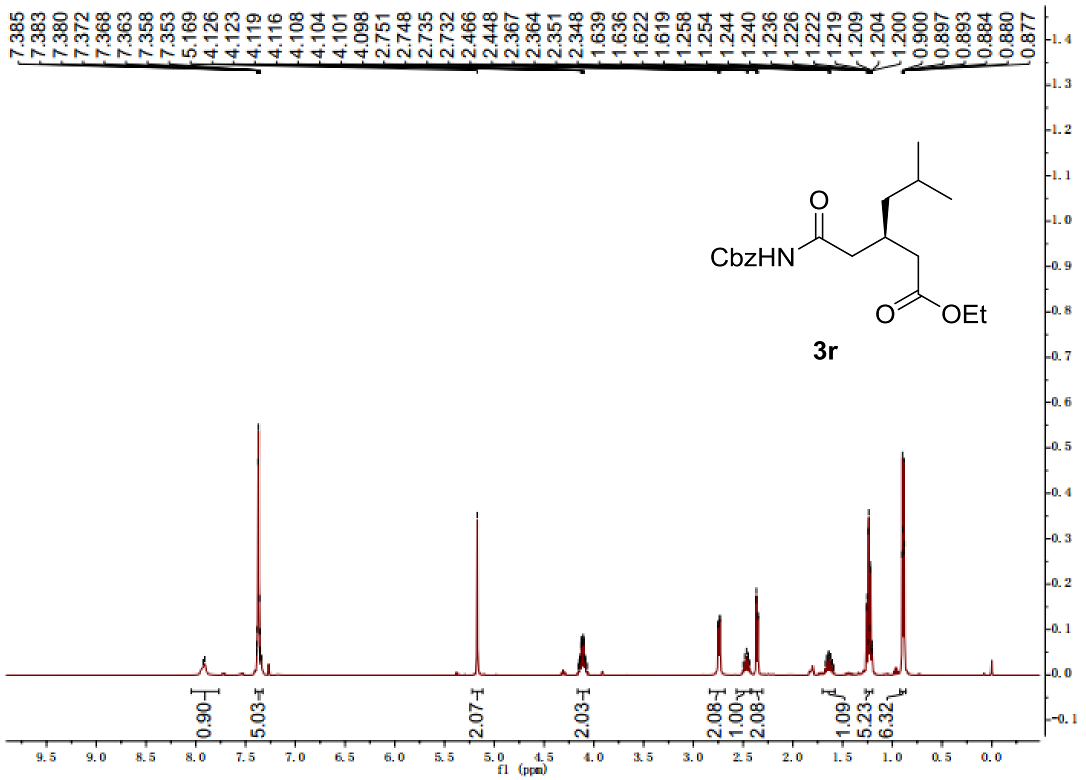

Supplementary Fig. 98. ^{1}H NMR Spectrum of **3r**

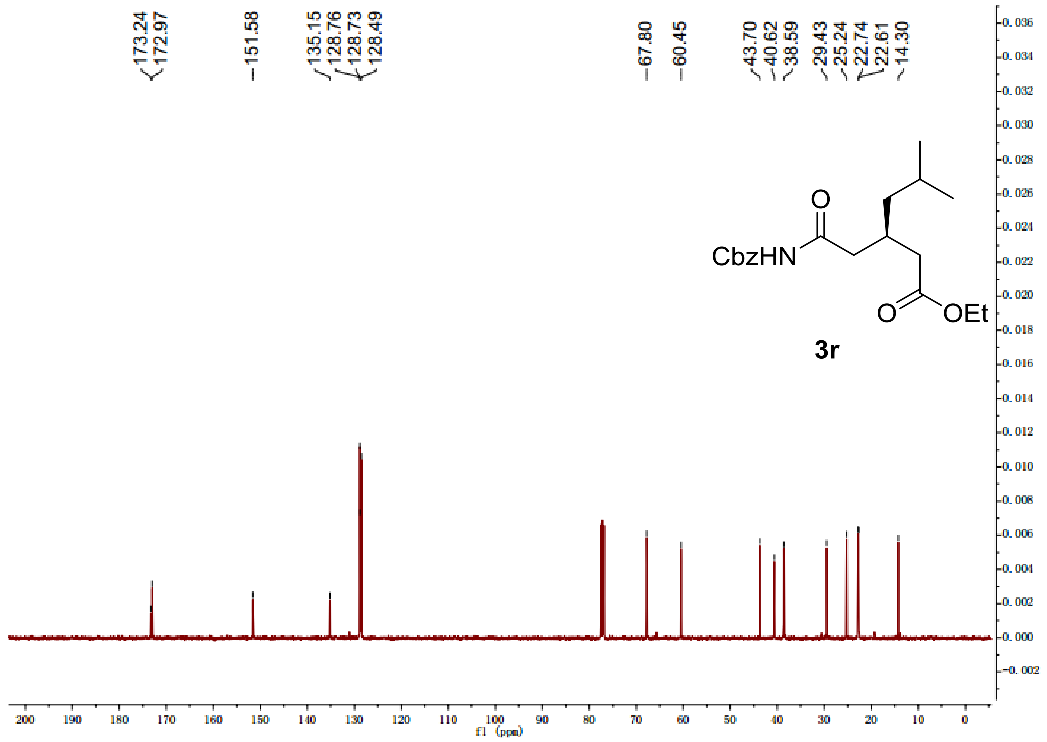

Supplementary Fig. 99. ^{13}C NMR Spectrum of **3r**

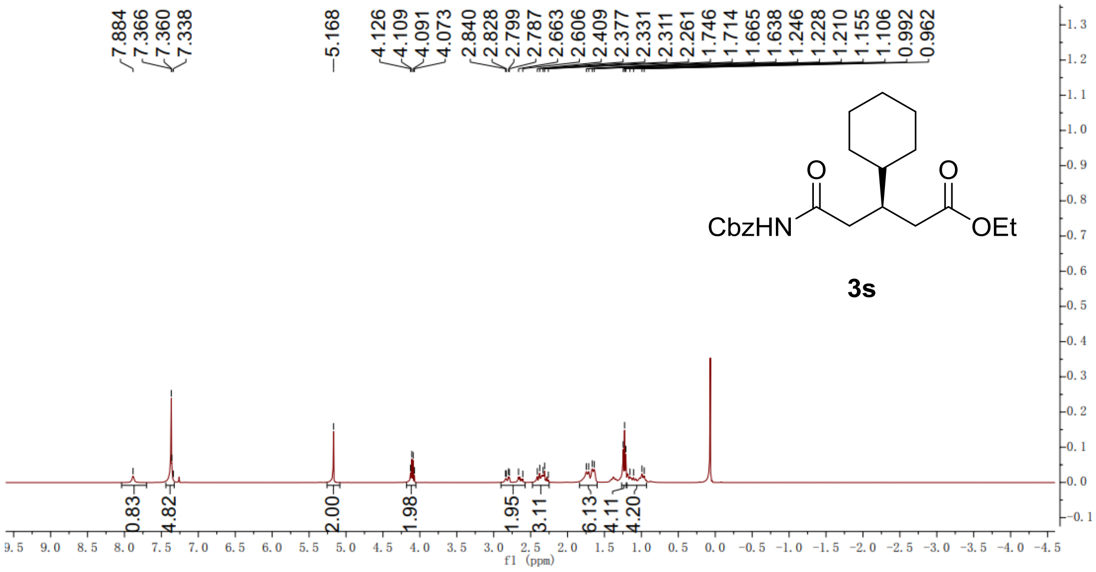

Supplementary Fig. 100. ^{1}H NMR Spectrum of **3s**

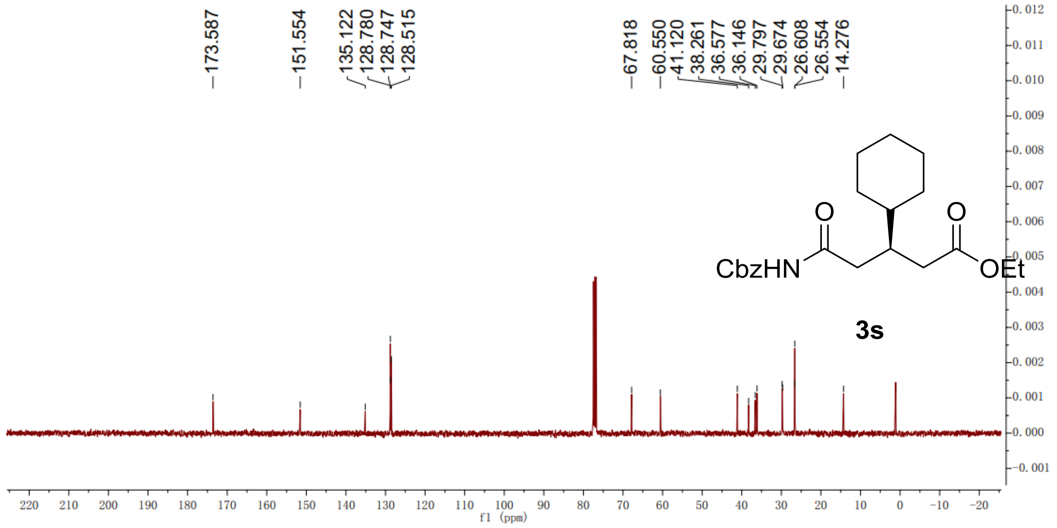

Supplementary Fig. 101. ^{13}C NMR Spectrum of **3s**

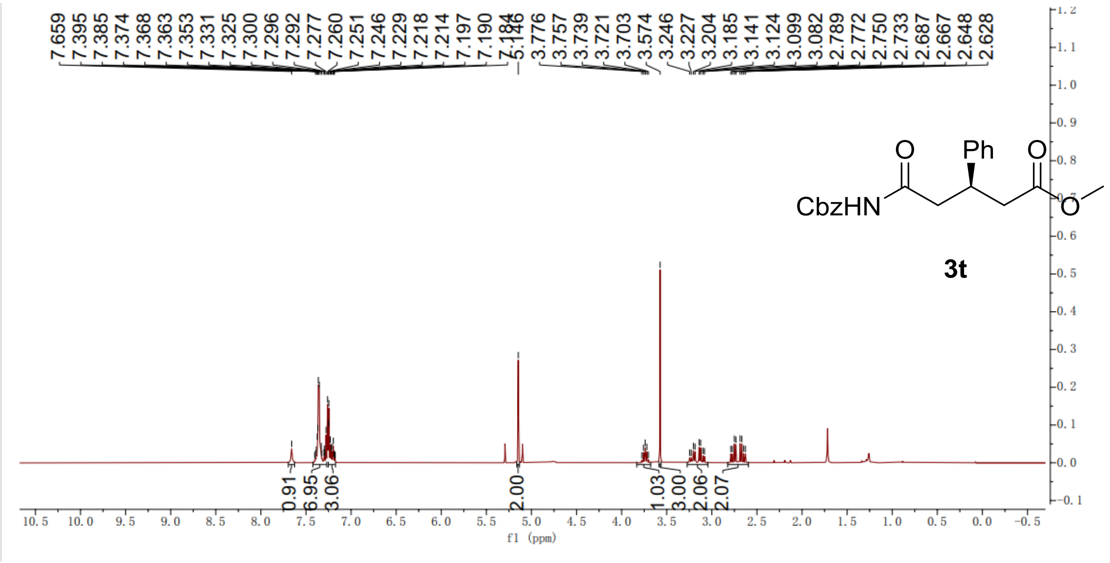

Supplementary Fig. 102. ^{1}H NMR Spectrum of **3t**

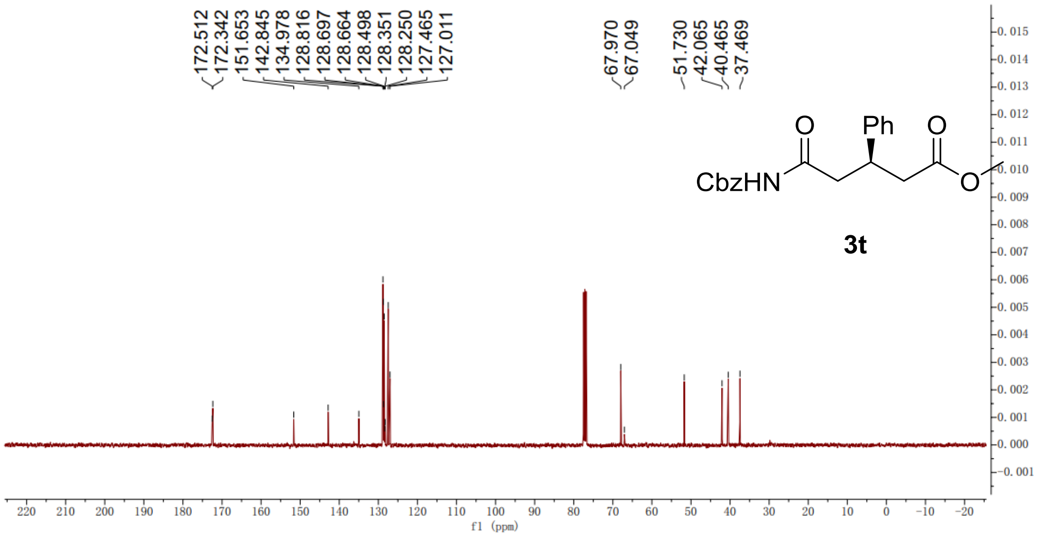

Supplementary Fig. 103. ^{13}C NMR Spectrum of **3t**

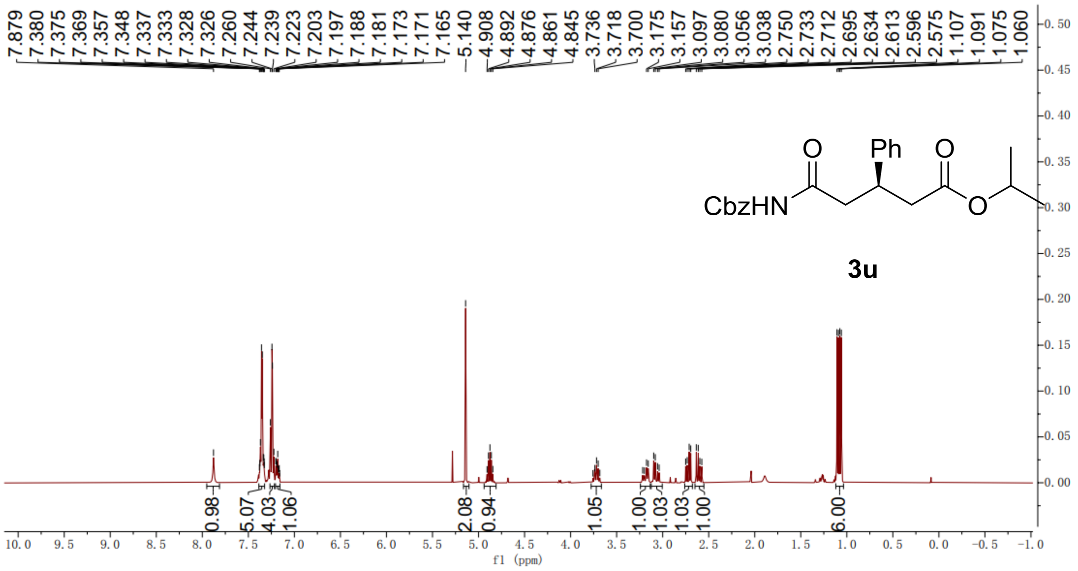

Supplementary Fig. 104. ^{1}H NMR Spectrum of **3u**

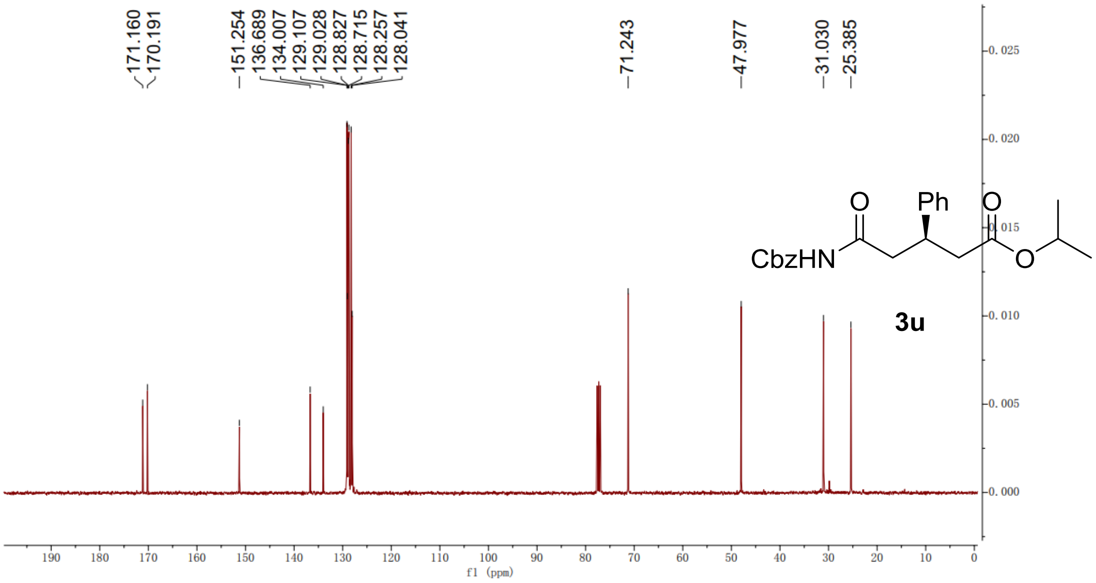

Supplementary Fig. 105. ^{13}C NMR Spectrum of **3u**

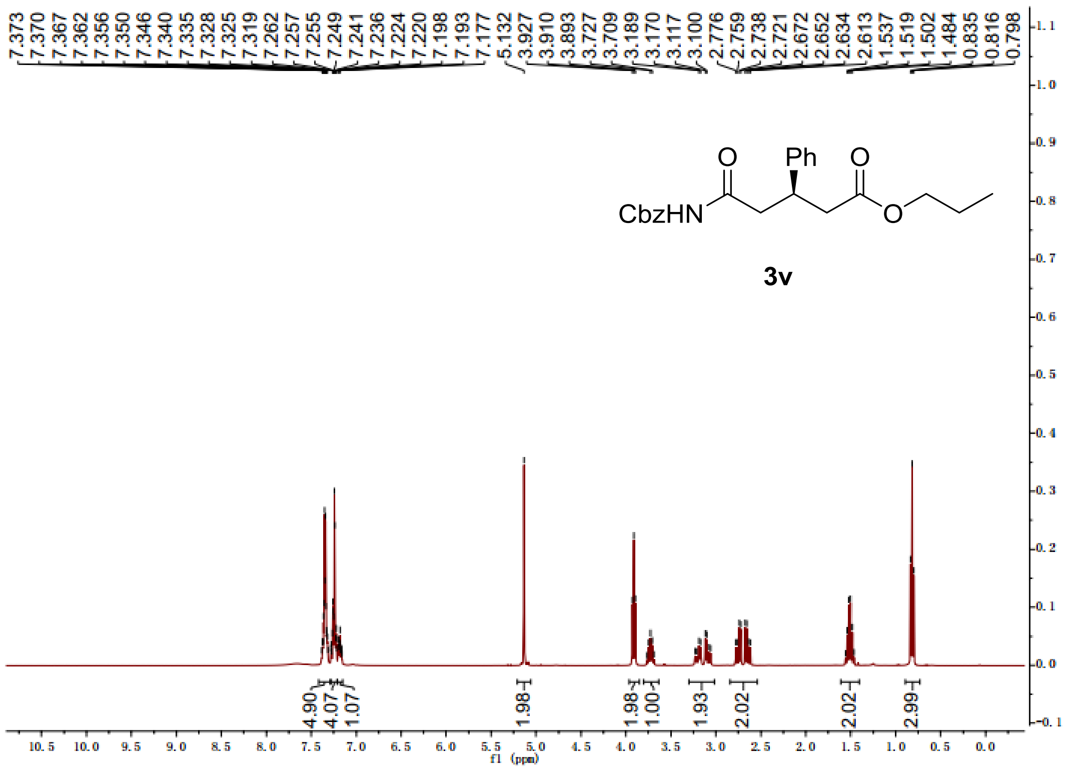

Supplementary Fig. 106. ^{1}H NMR Spectrum of **3v**

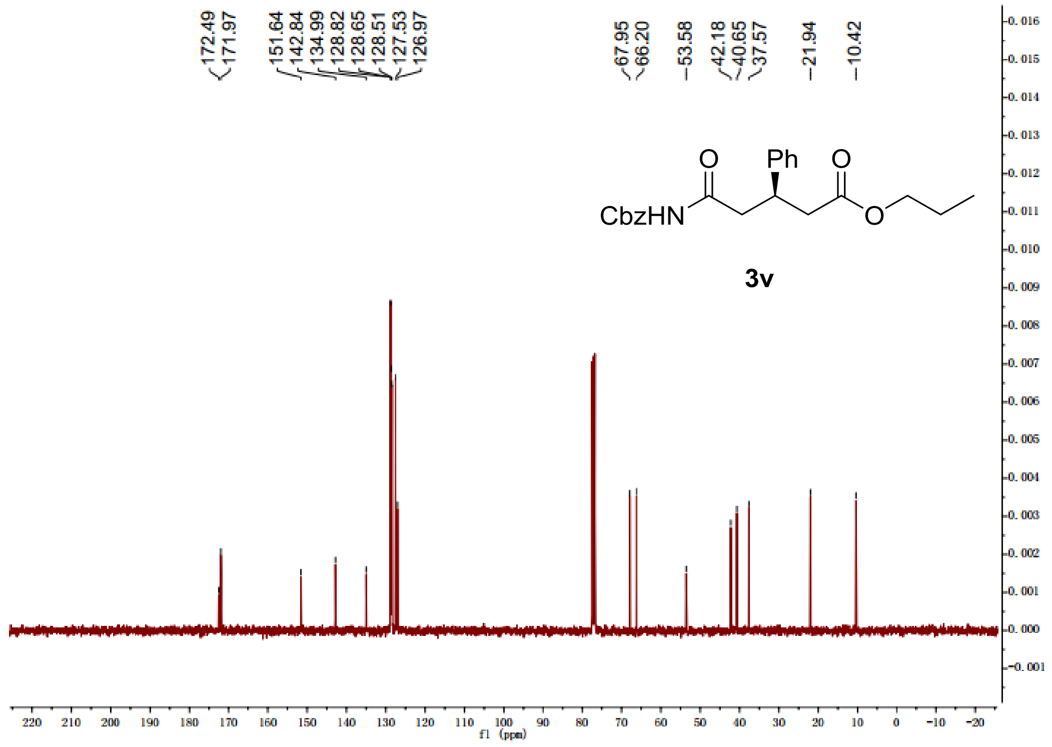

Supplementary Fig. 107. ^{13}C NMR Spectrum of **3v**

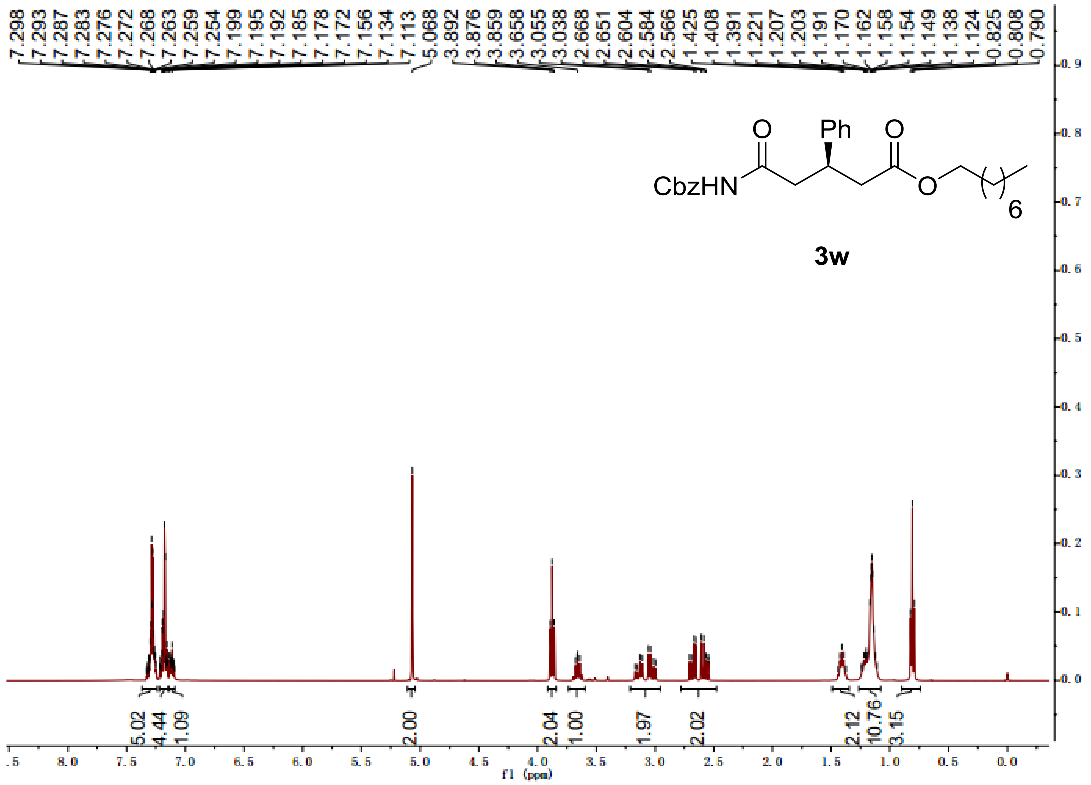

Supplementary Fig. 108. ^{1}H NMR Spectrum of **3w**

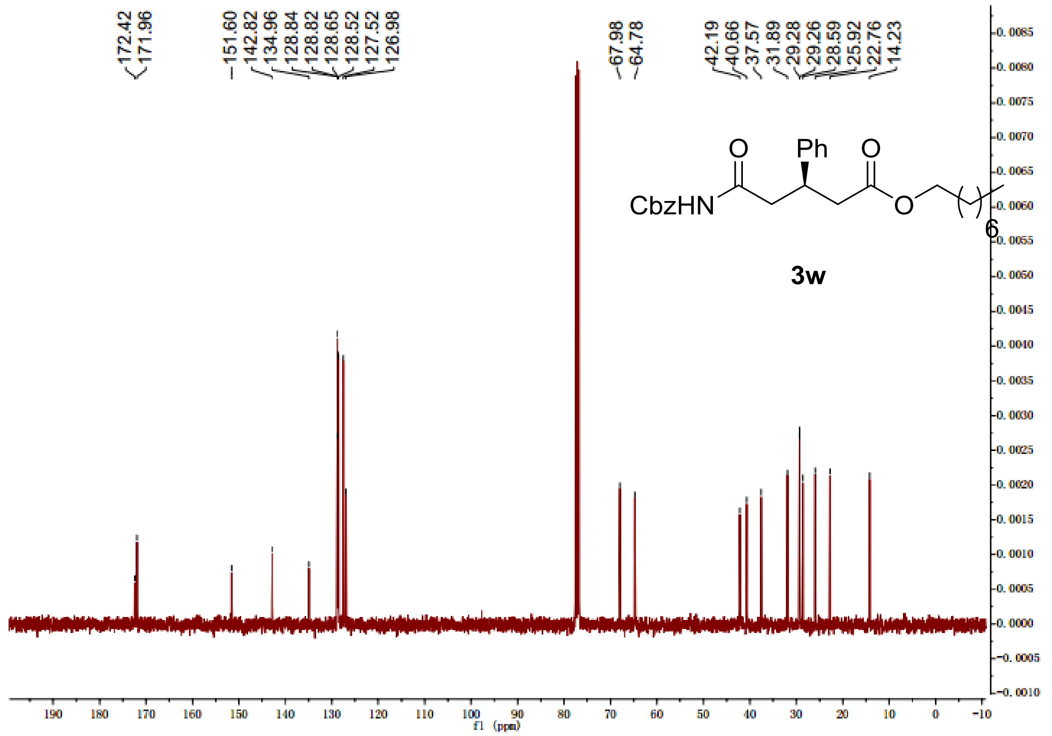

Supplementary Fig. 109. ^{13}C NMR Spectrum of **3w**

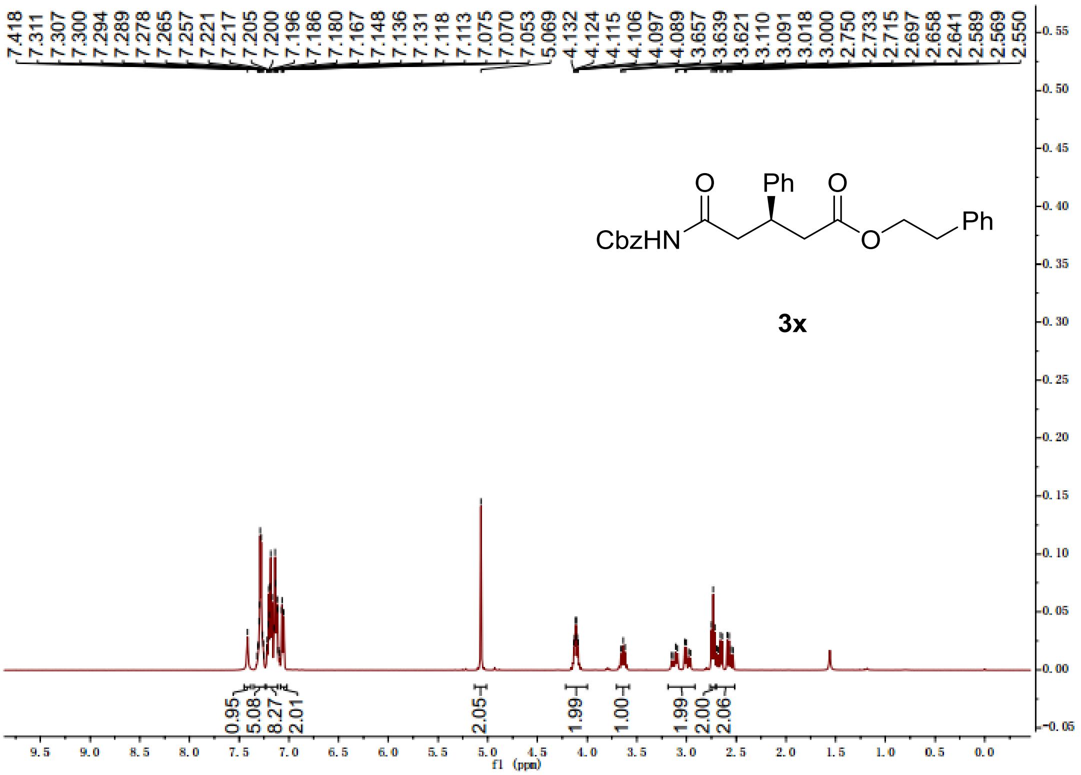

Supplementary Fig. 110. ^{1}H NMR Spectrum of **3x**

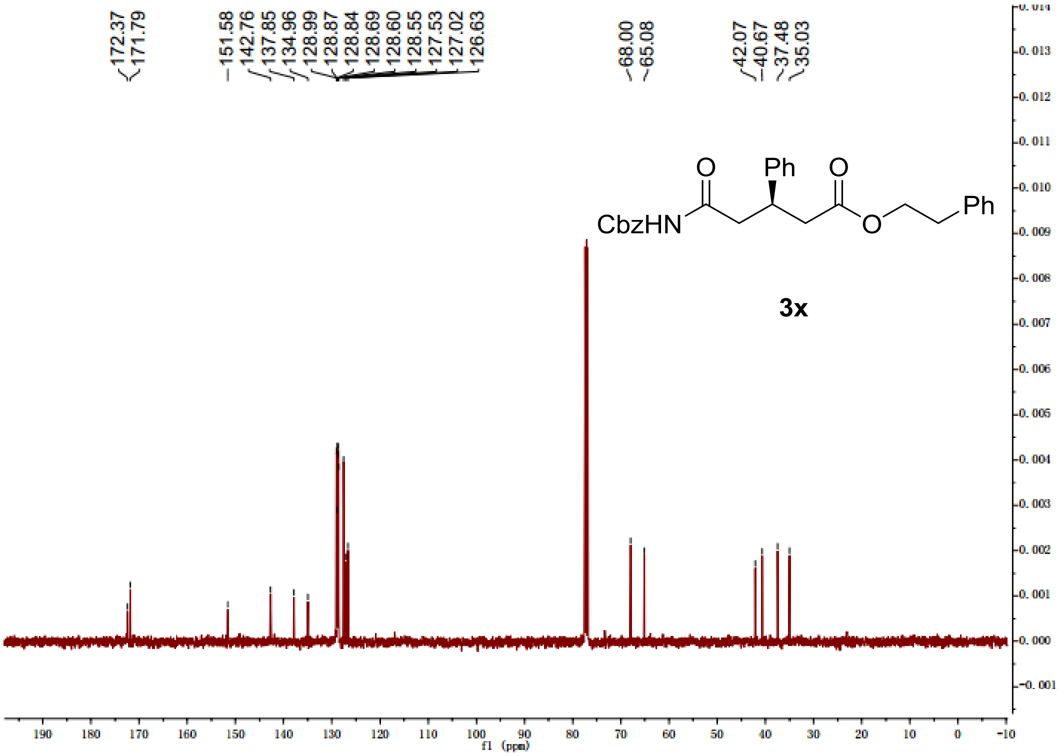

Supplementary Fig. 111. ^{13}C NMR Spectrum of **3x**

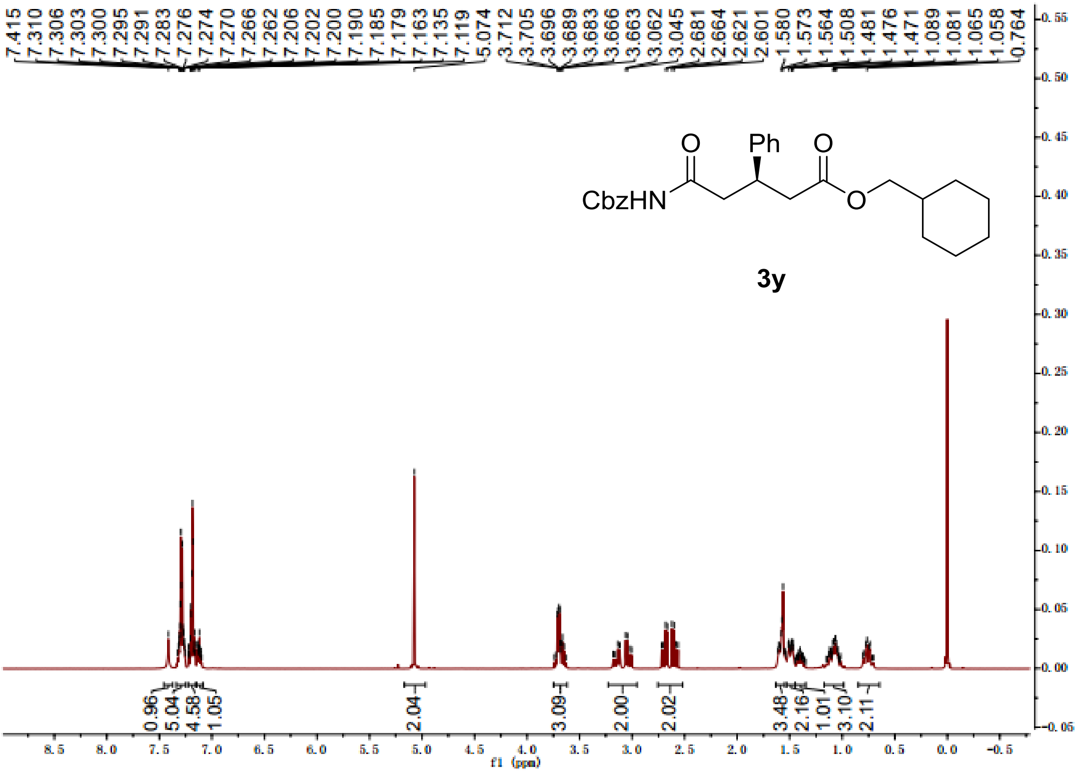

Supplementary Fig. 112. ^{1}H NMR Spectrum of **3y**

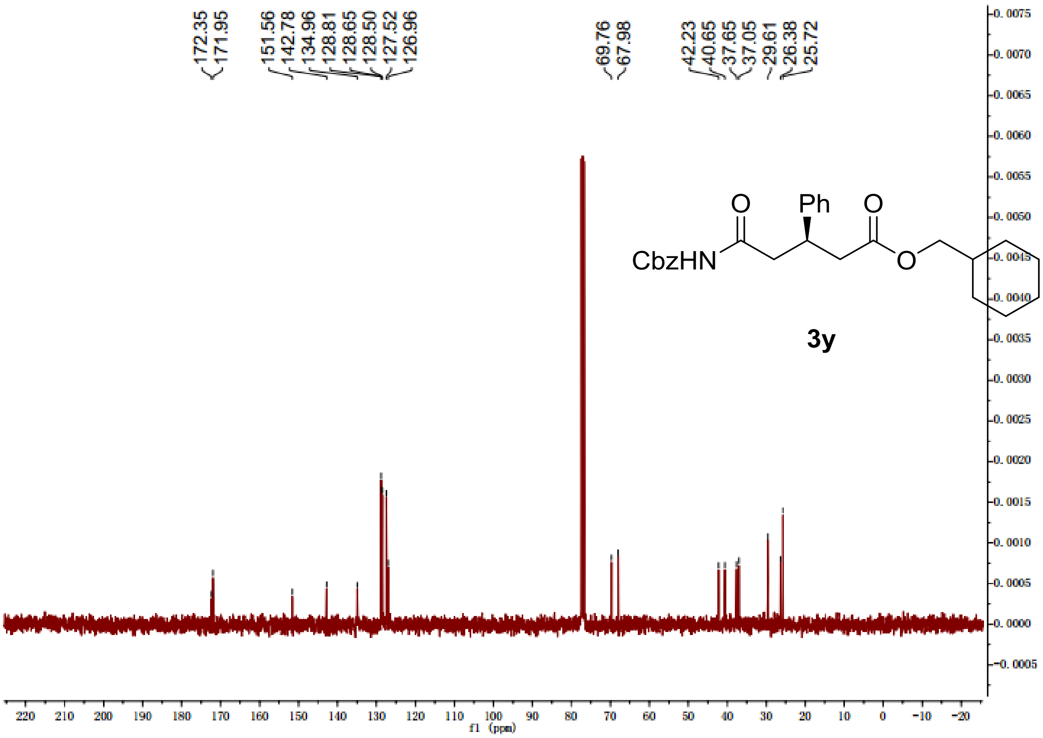

Supplementary Fig. 113. ^{13}C NMR Spectrum of **3y**

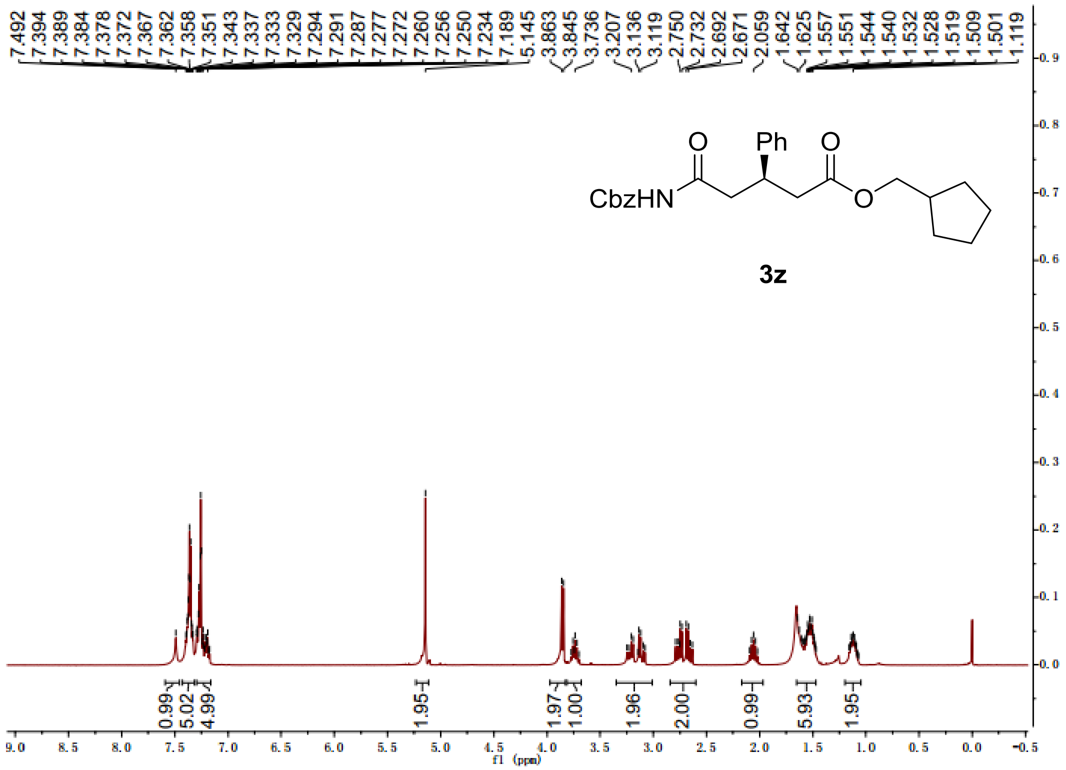

Supplementary Fig. 114. ^{1}H NMR Spectrum of **3z**

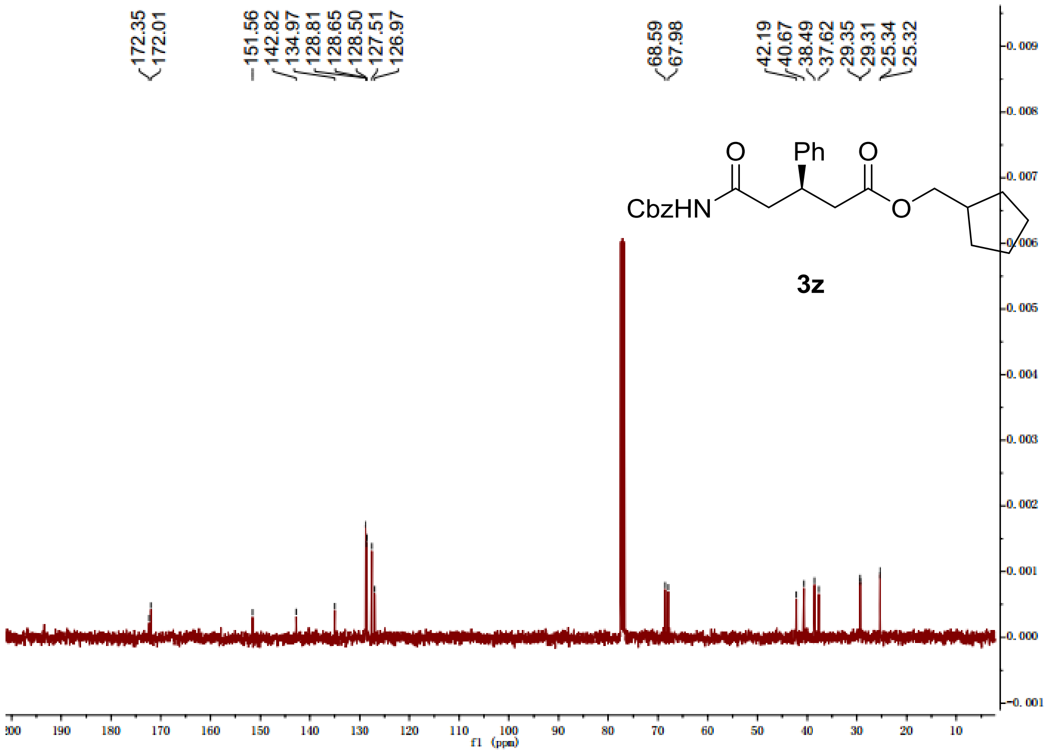

Supplementary Fig. 115. ^{13}C NMR Spectrum of **3z**

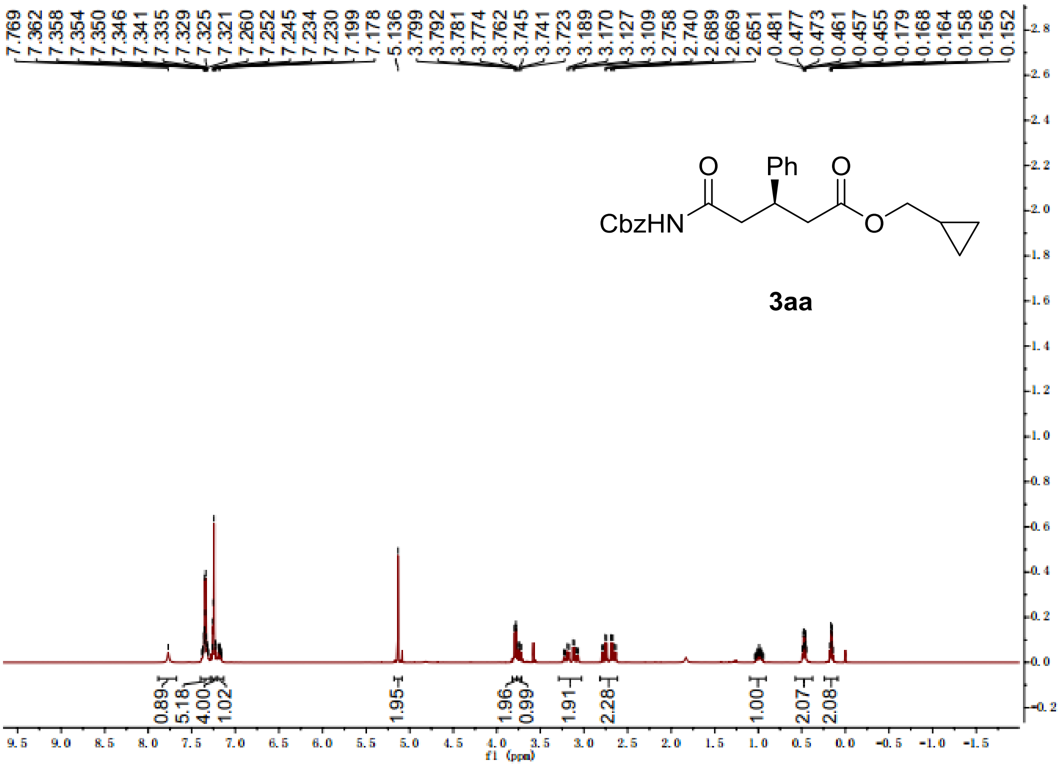

Supplementary Fig. 116. ^{1}H NMR Spectrum of **3aa**

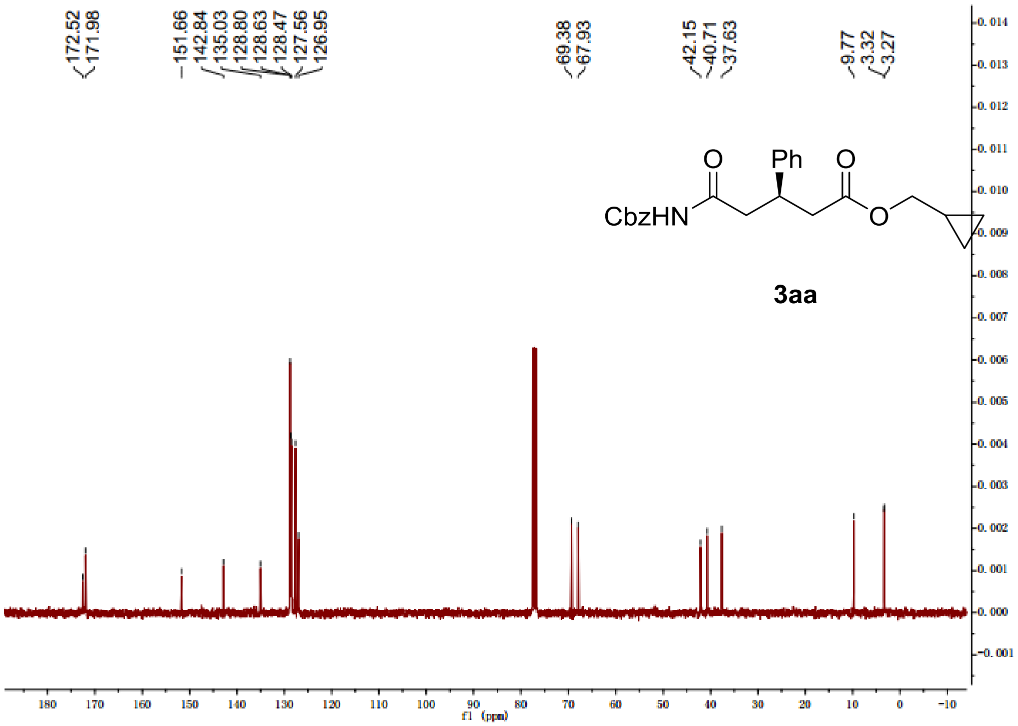

Supplementary Fig. 117. ^{13}C NMR Spectrum of **3aa**

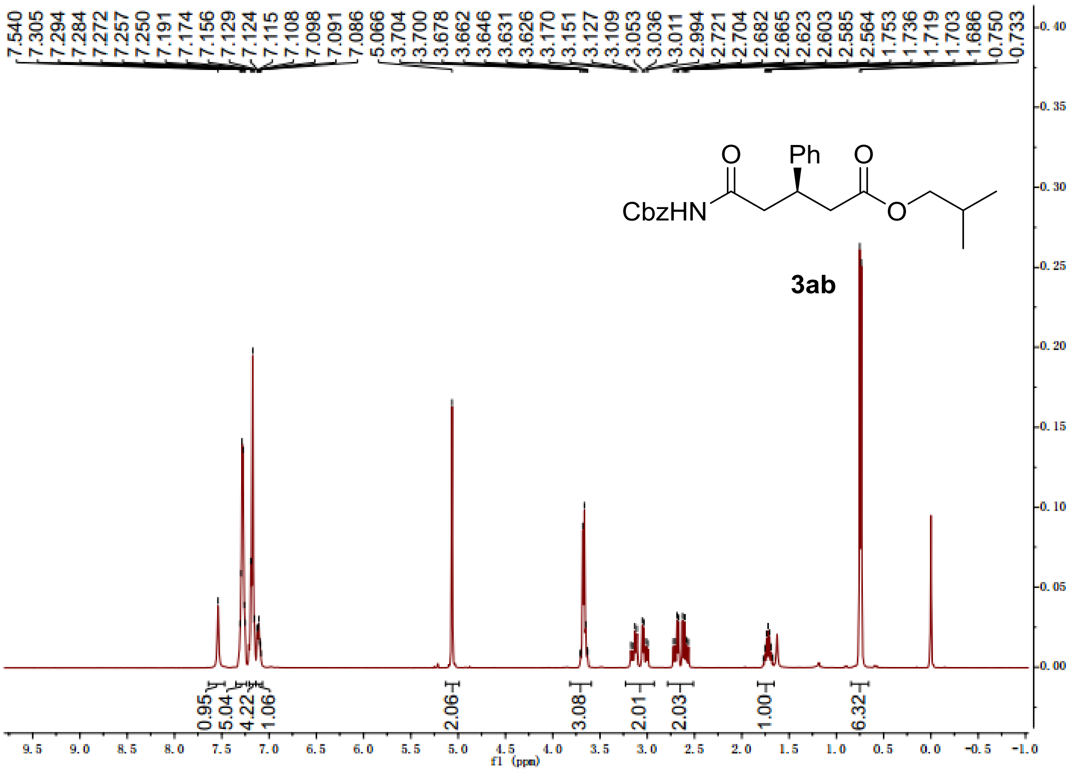

Supplementary Fig. 118. ^{1}H NMR Spectrum of **3ab**

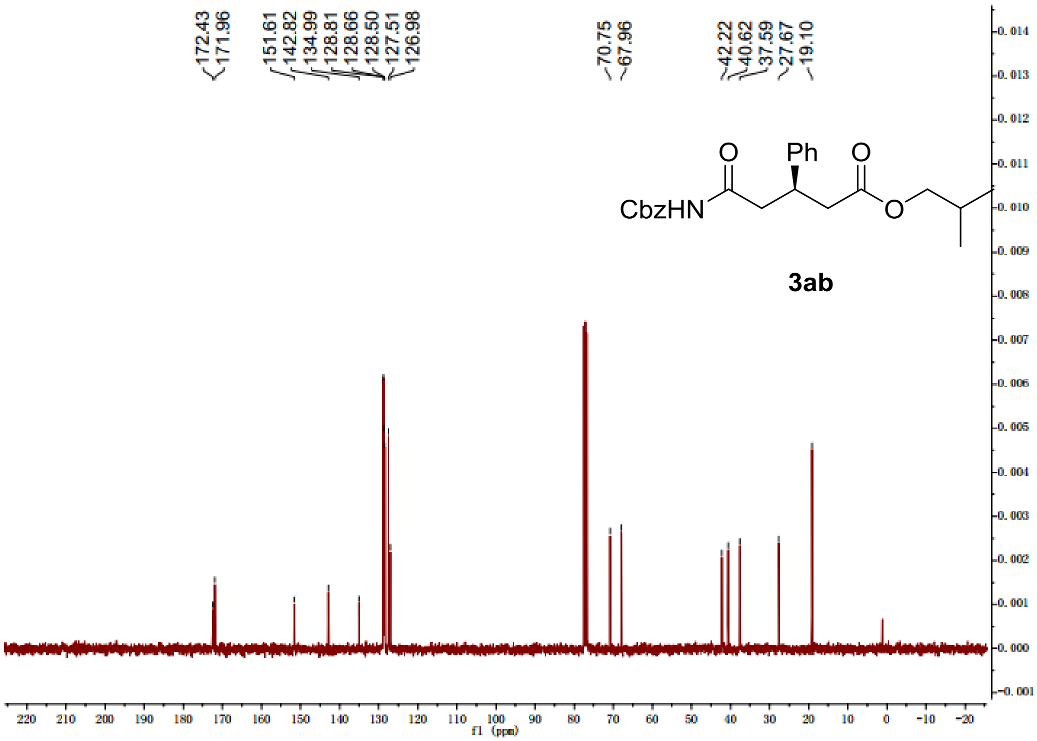

Supplementary Fig. 119. ^{13}C NMR Spectrum of **3ab**

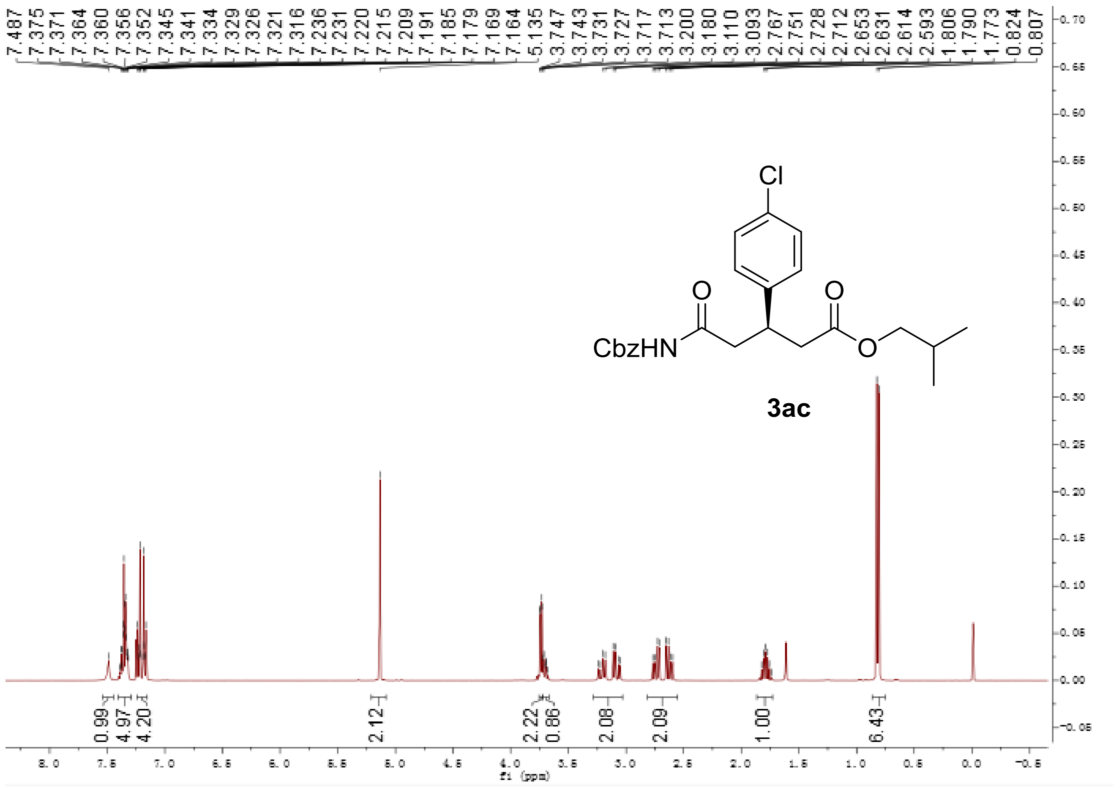

Supplementary Fig. 120. ^{1}H NMR Spectrum of **3ac**

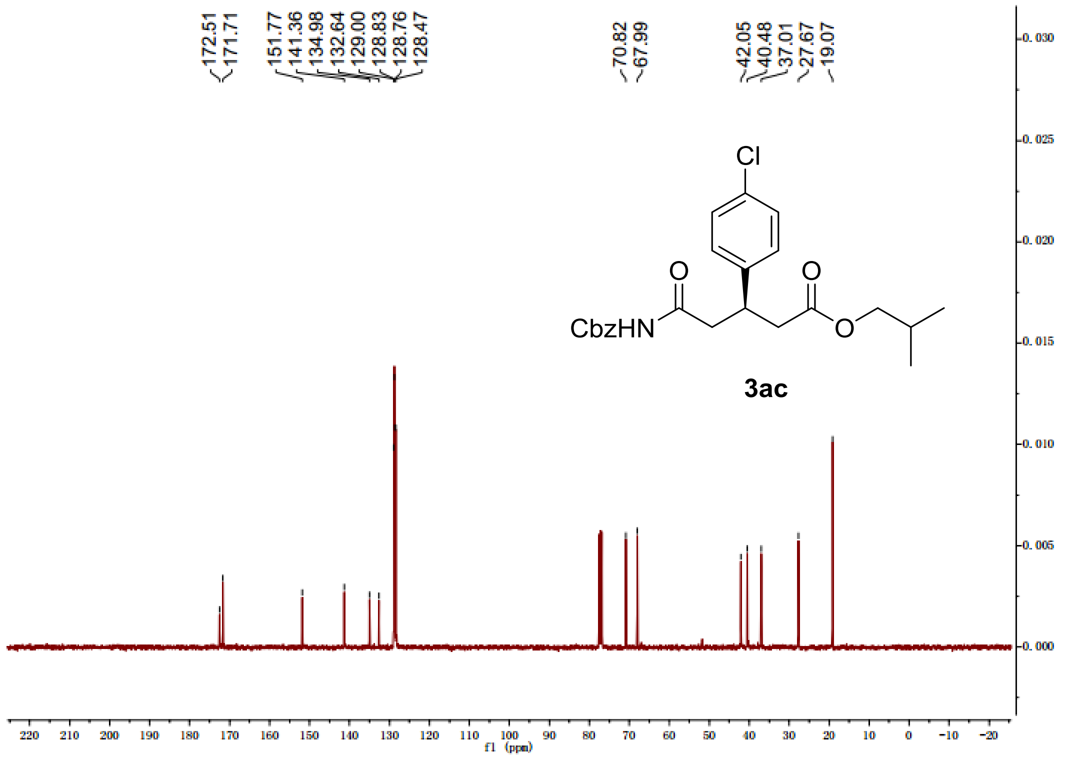

Supplementary Fig. 121. ^{13}C NMR Spectrum of **3ac**

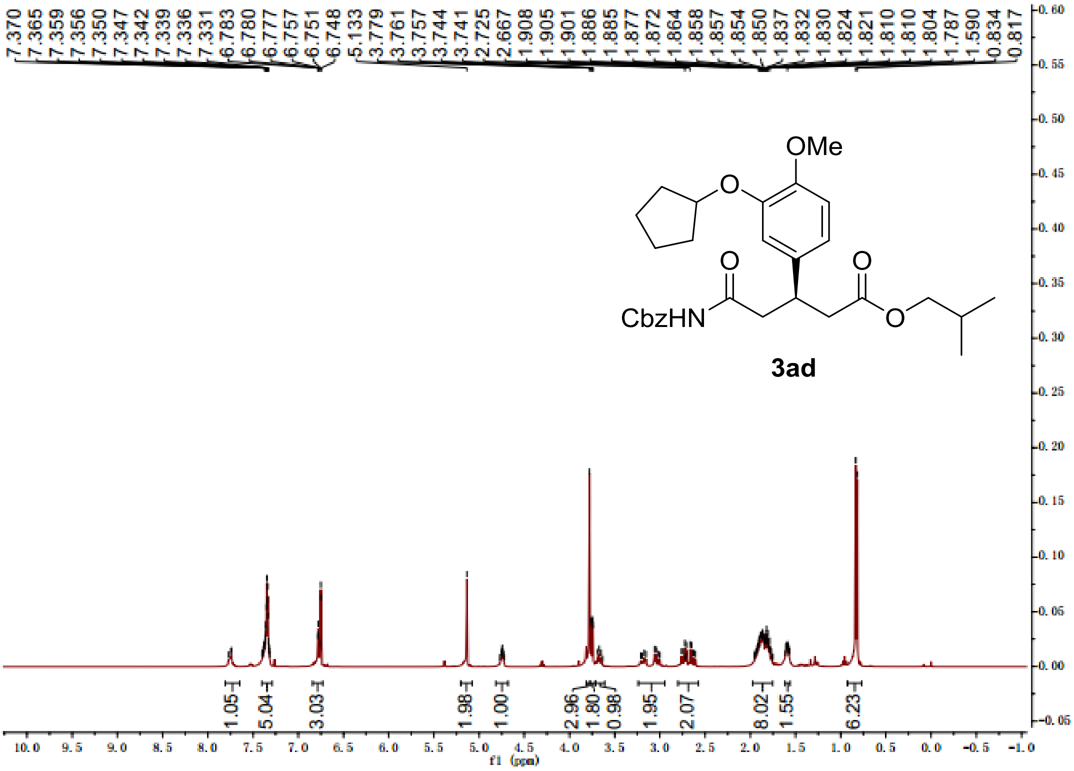

Supplementary Fig. 122. ^{1}H NMR Spectrum of **3ad**

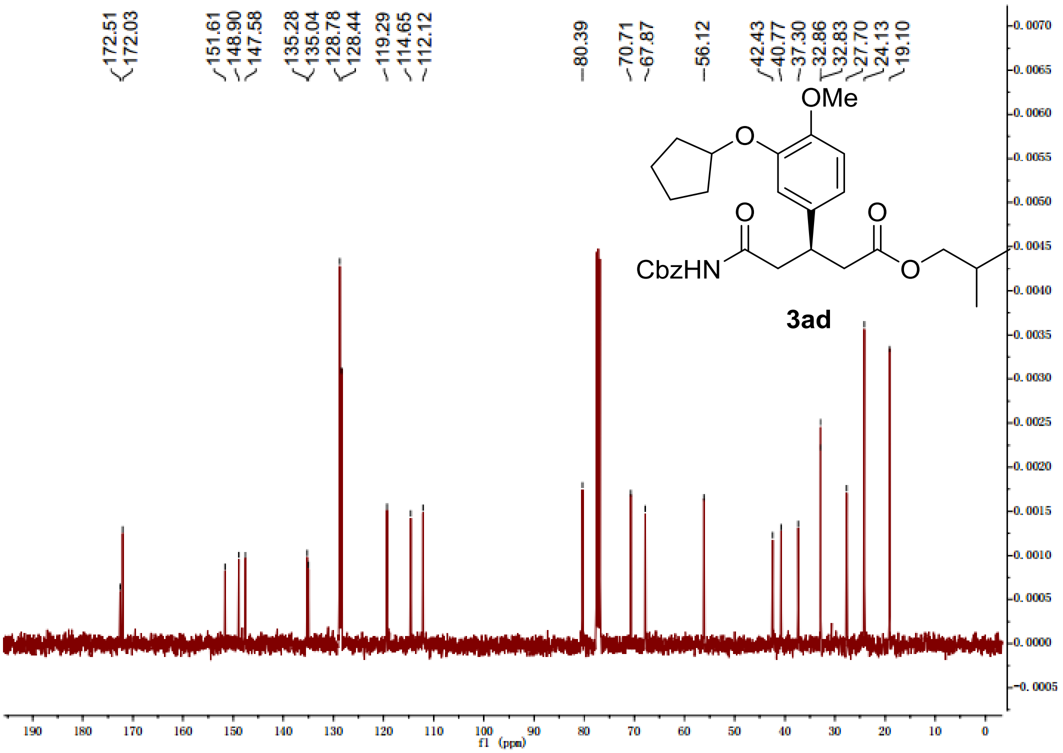

Supplementary Fig. 123. ^{13}C NMR Spectrum of **3ad**

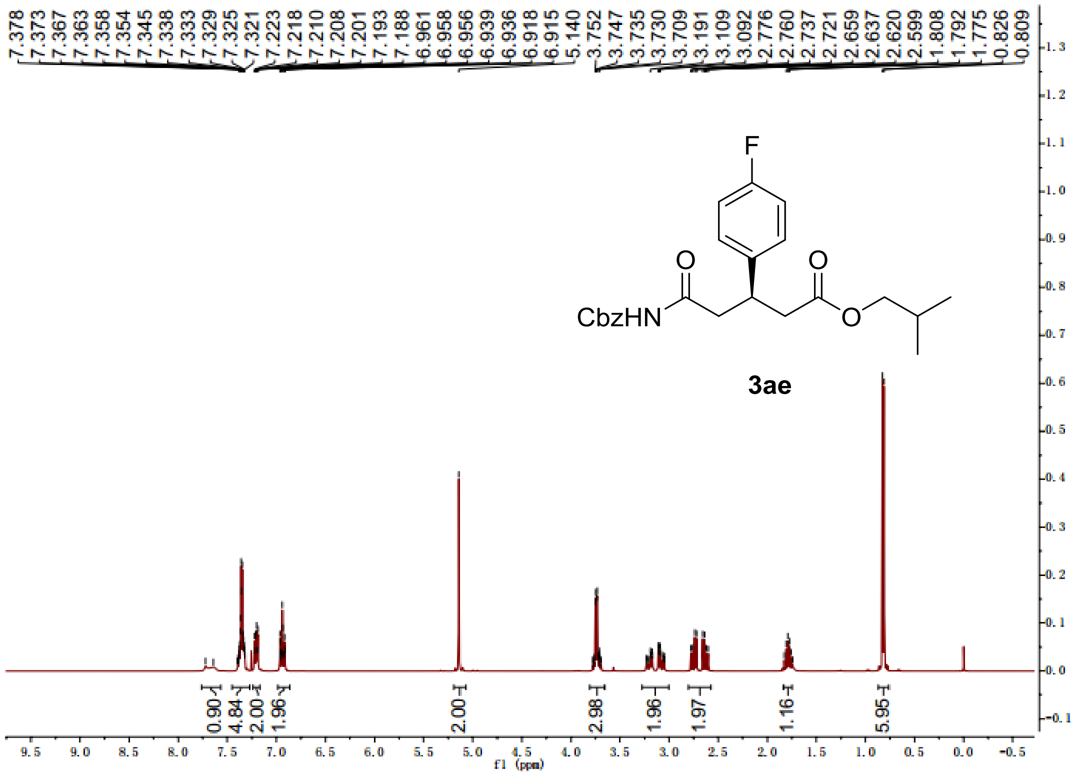

Supplementary Fig. 124. ^{1}H NMR Spectrum of **3ae**

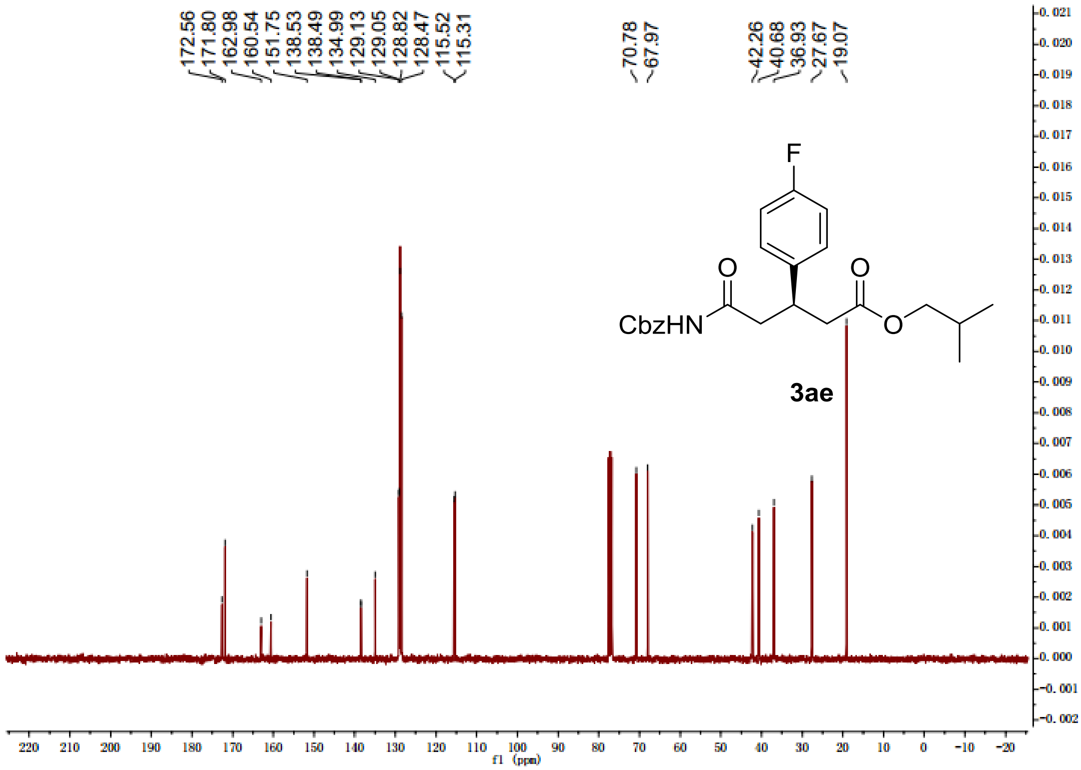

Supplementary Fig. 125. ^{13}C NMR Spectrum of **3ae**

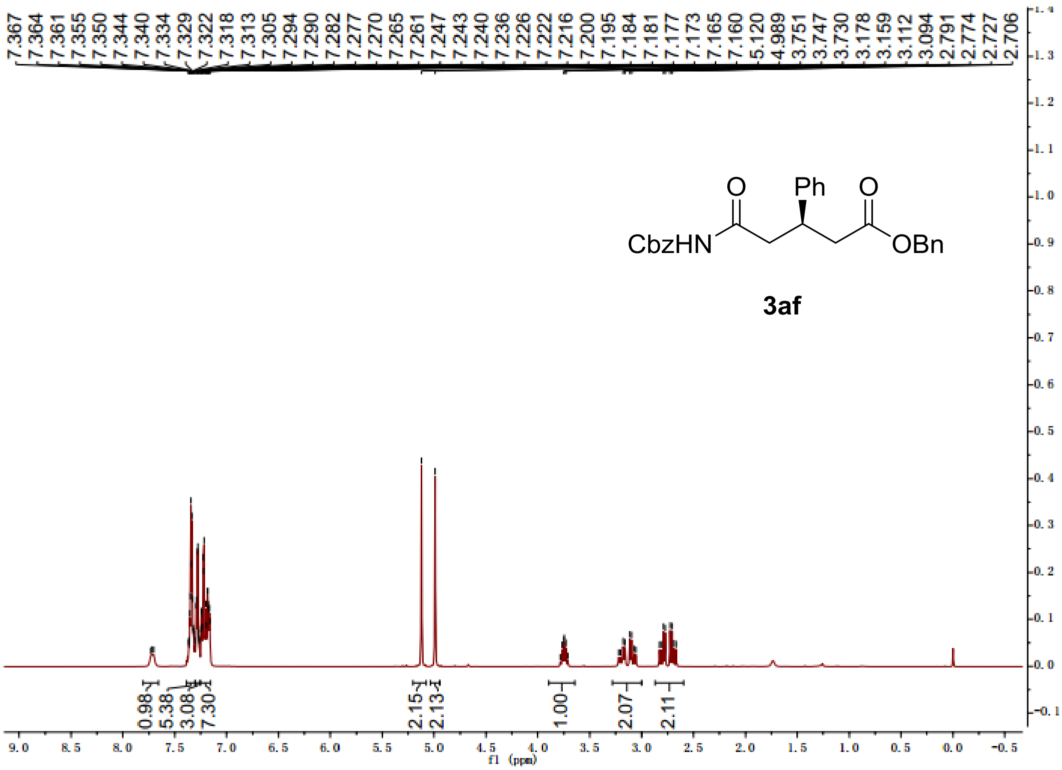

Supplementary Fig. 126. ^{1}H NMR Spectrum of **3af**

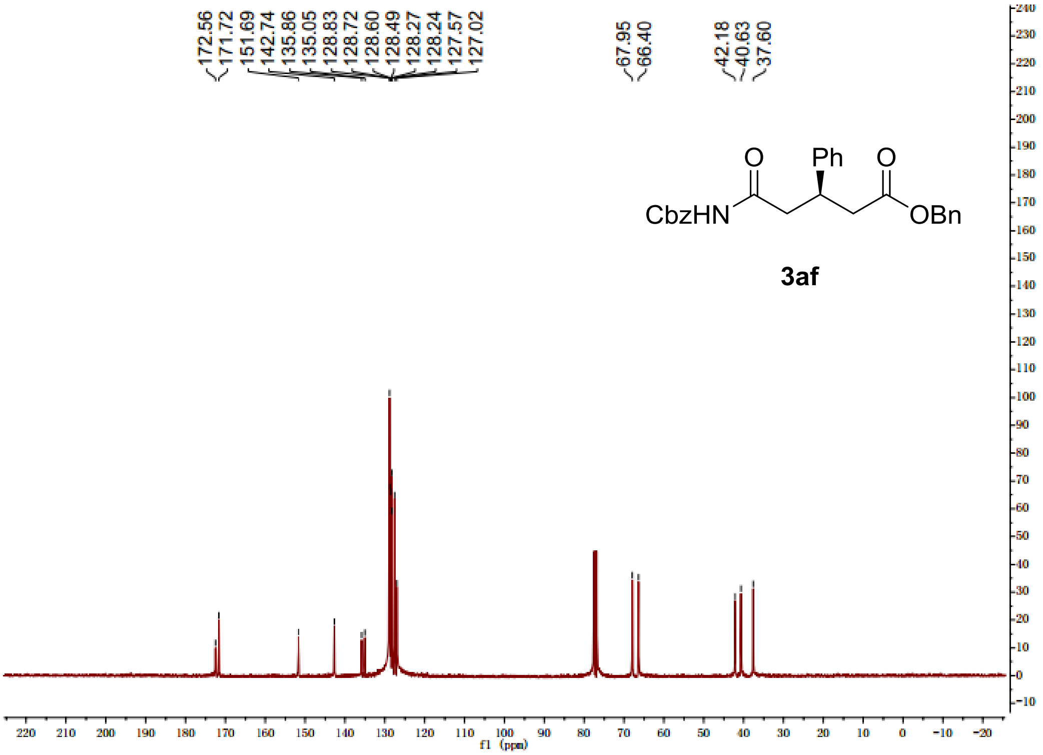

Supplementary Fig. 127. ^{13}C NMR Spectrum of **3af**

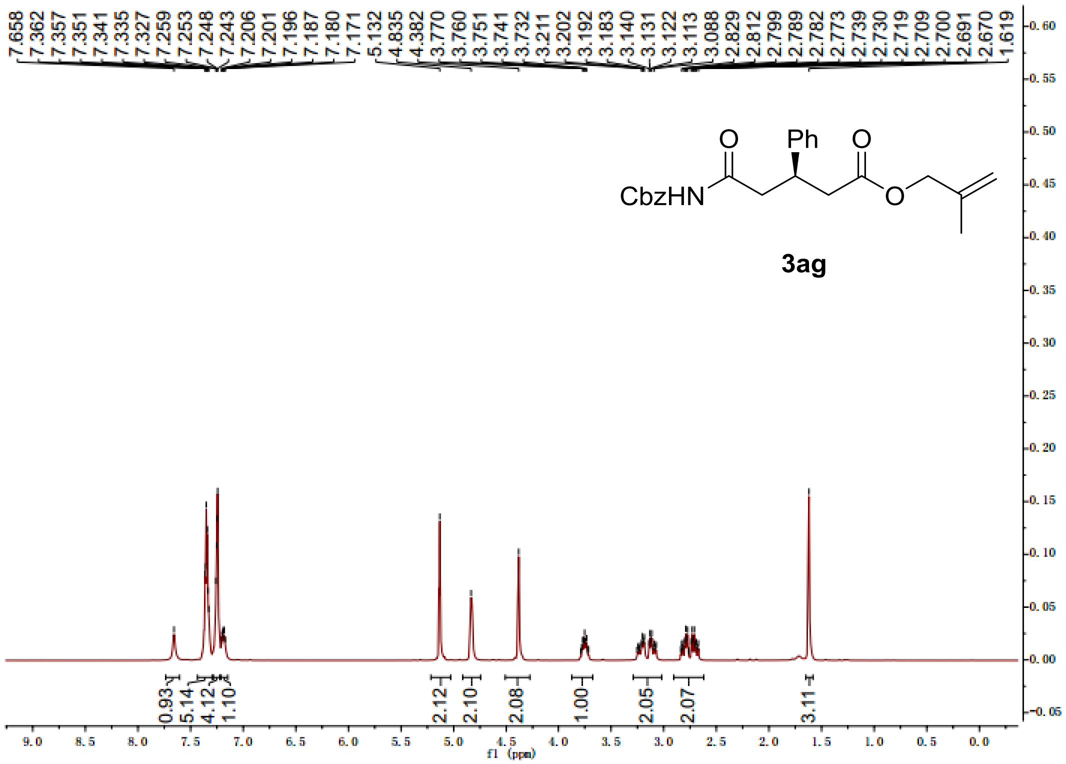

Supplementary Fig. 128. ^{1}H NMR Spectrum of **3ag**

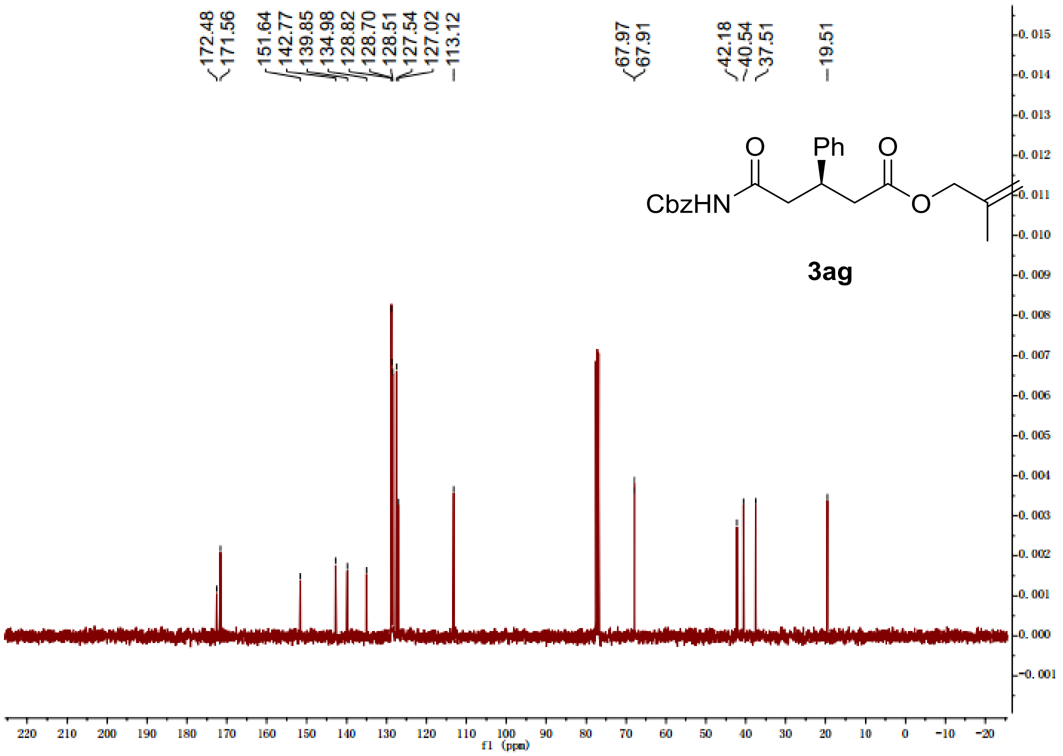

Supplementary Fig. 129. ^{13}C NMR Spectrum of **3ag**

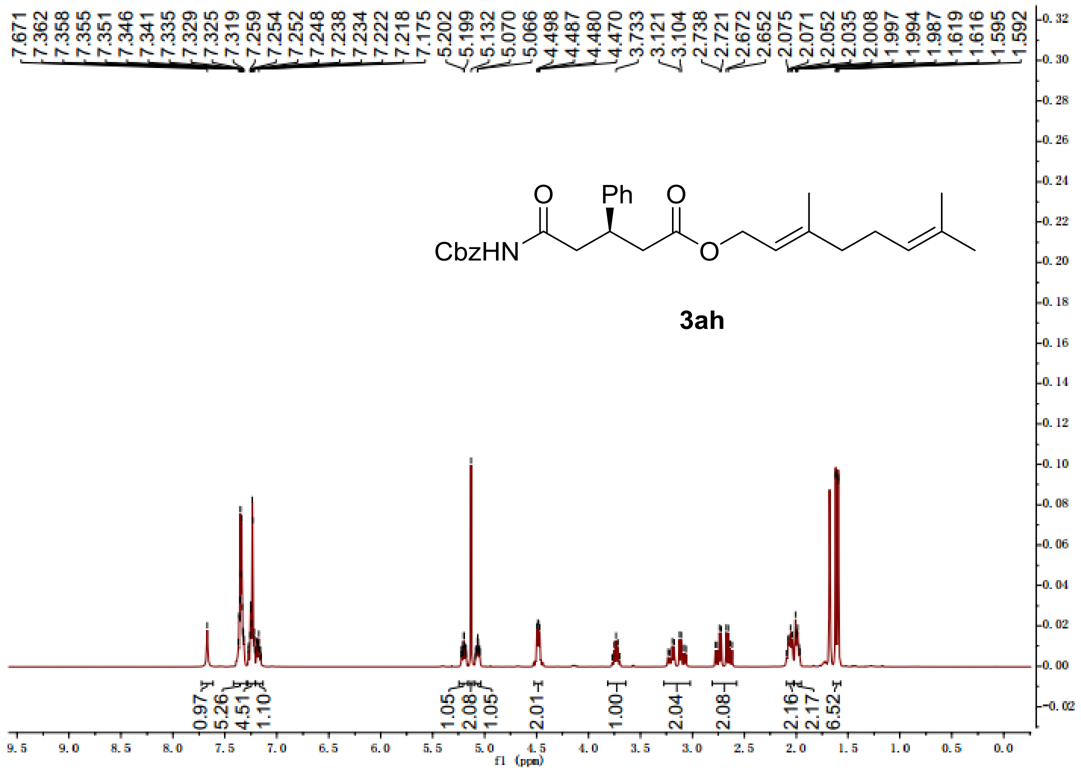

Supplementary Fig. 130. ^{1}H NMR Spectrum of **3ah**

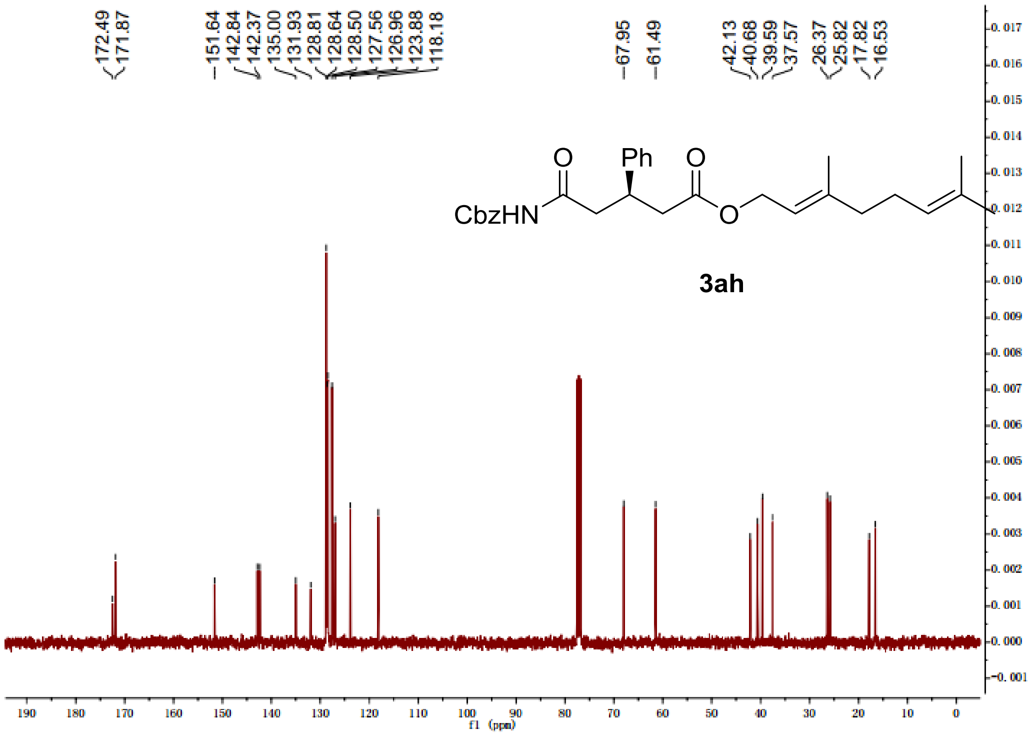

Supplementary Fig. 131. ^{13}C NMR Spectrum of **3ah**

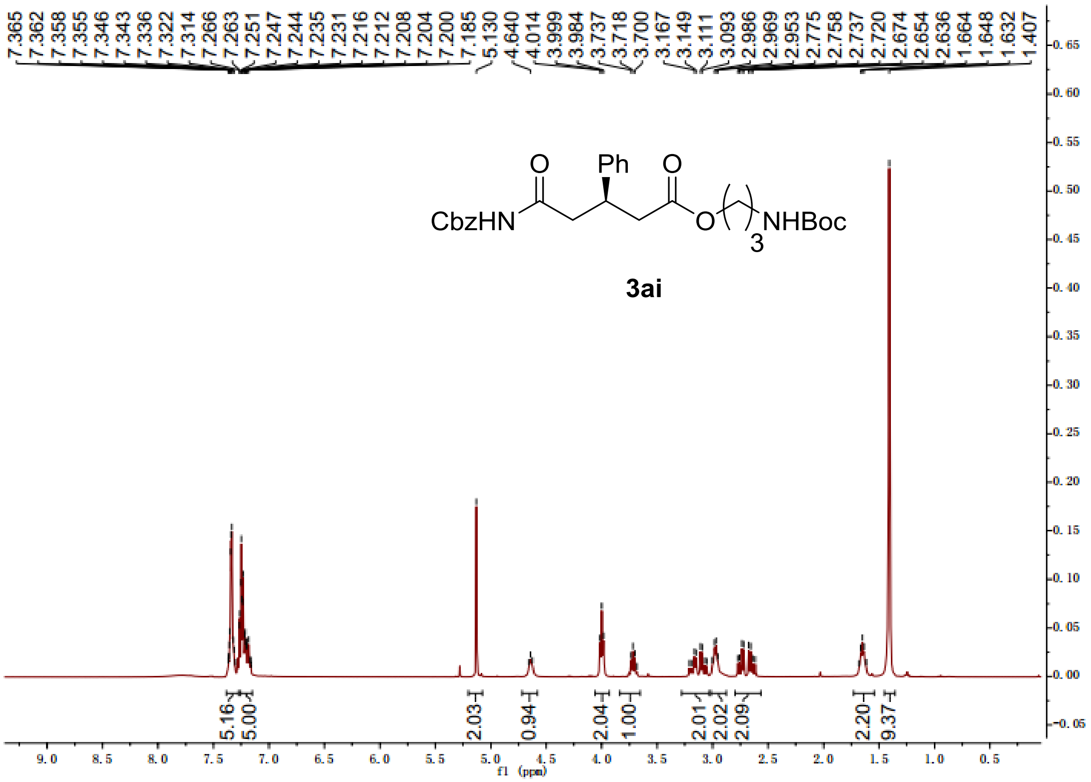

Supplementary Fig. 132. ^{1}H NMR Spectrum of **3ai**

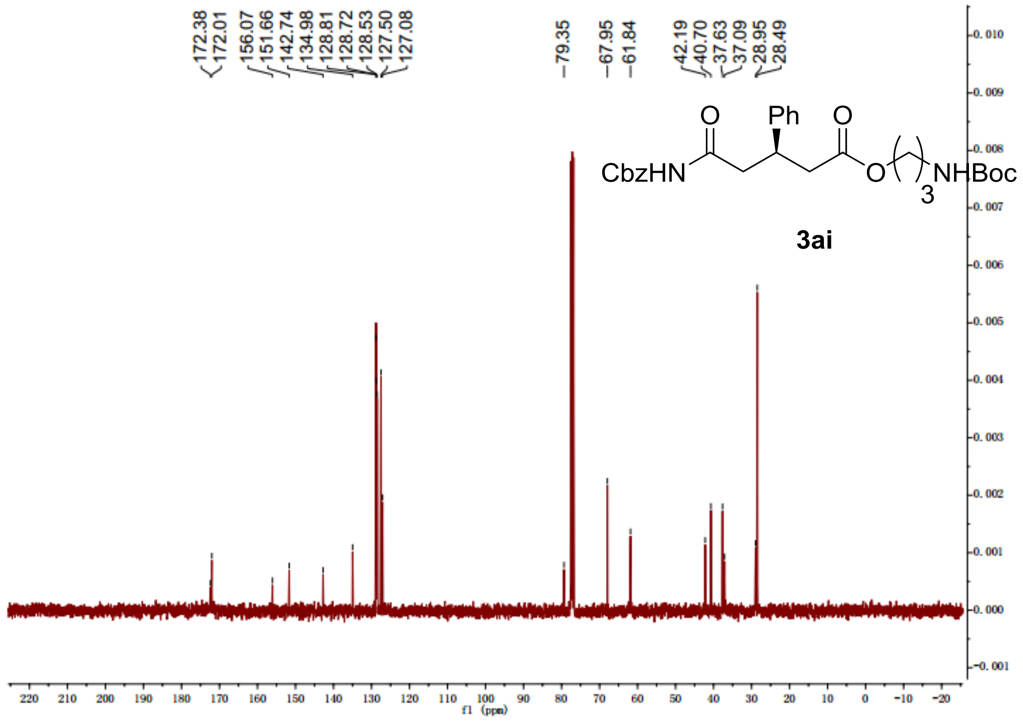

Supplementary Fig. 133. ^{13}C NMR Spectrum of **3ai**

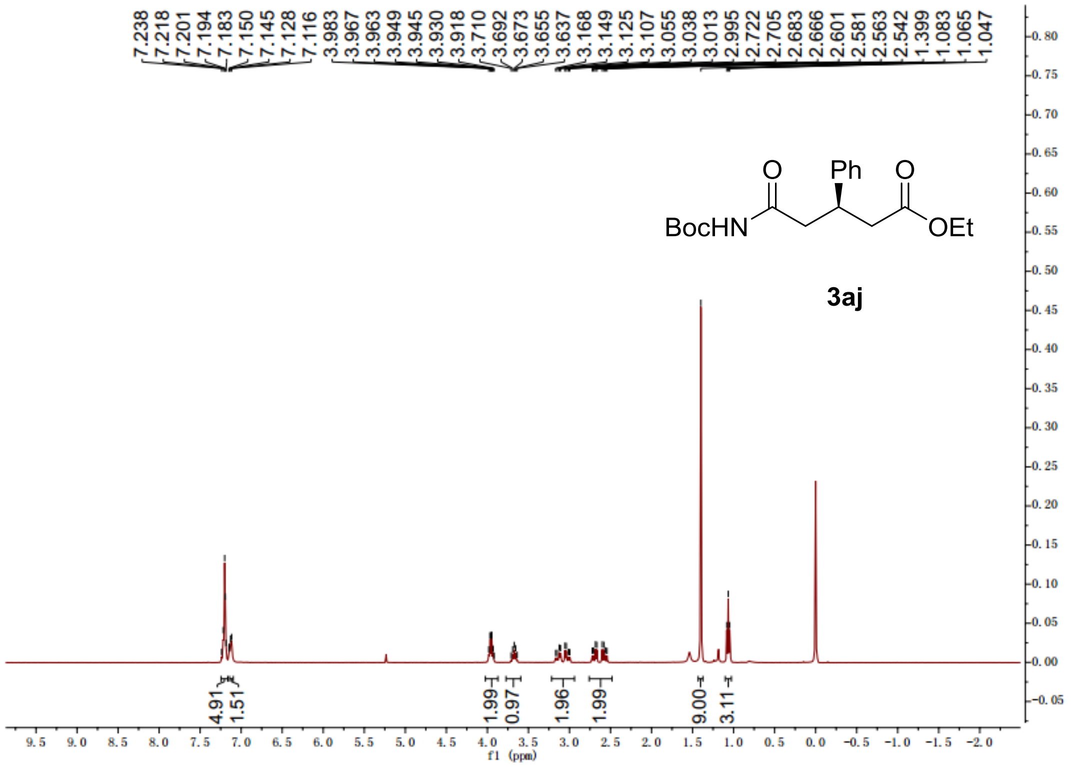

Supplementary Fig. 134. ^{1}H NMR Spectrum of **3aj**

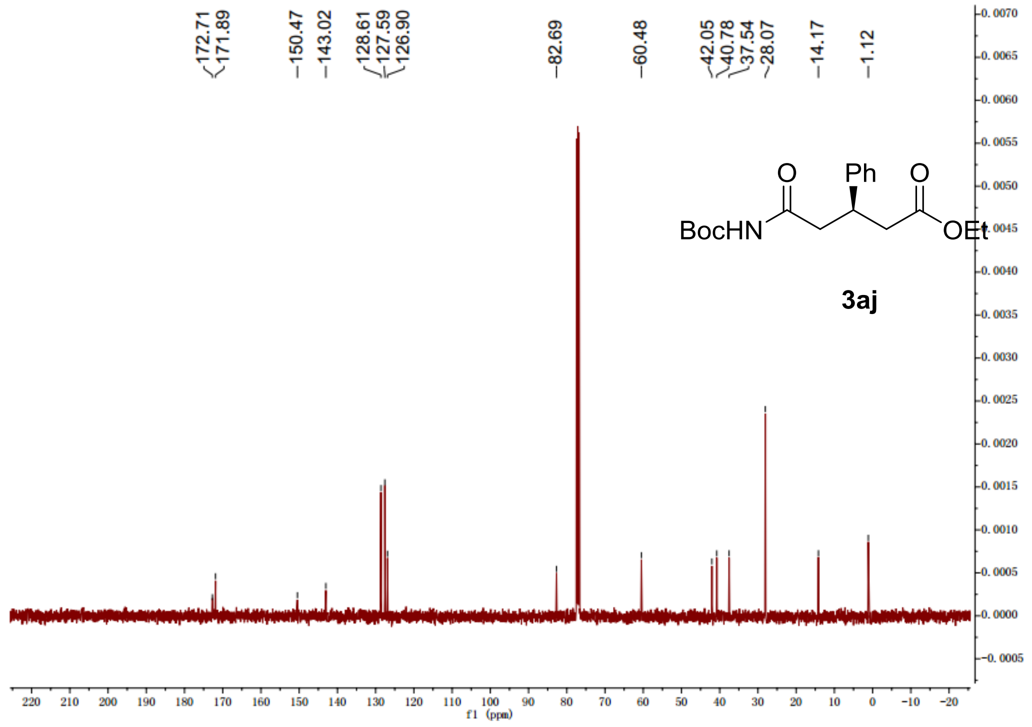

Supplementary Fig. 135. ^{13}C NMR Spectrum of **3aj**

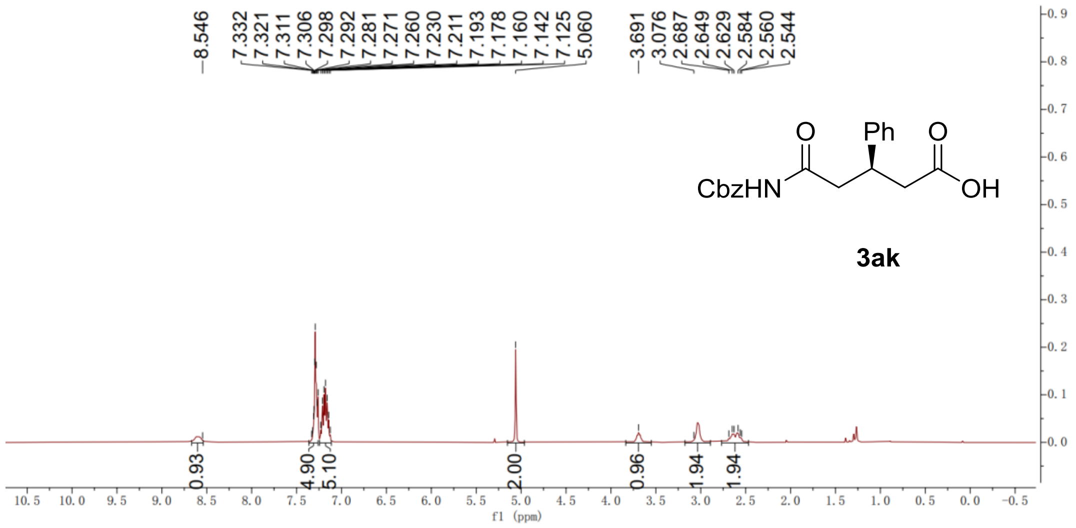

Supplementary Fig. 136. ^{1}H NMR Spectrum of **3ak**

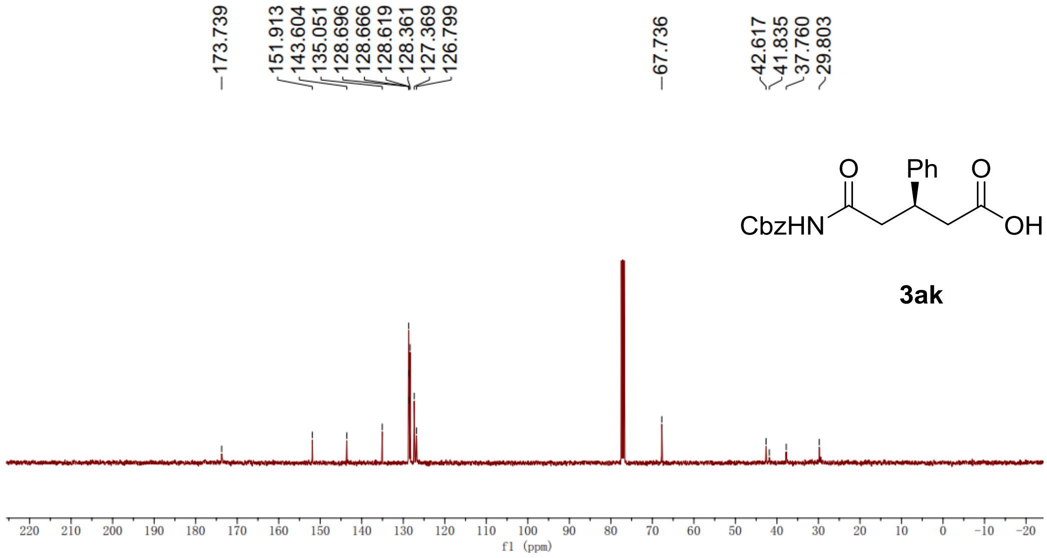

Supplementary Fig. 137. ^{13}C NMR Spectrum of **3ak**

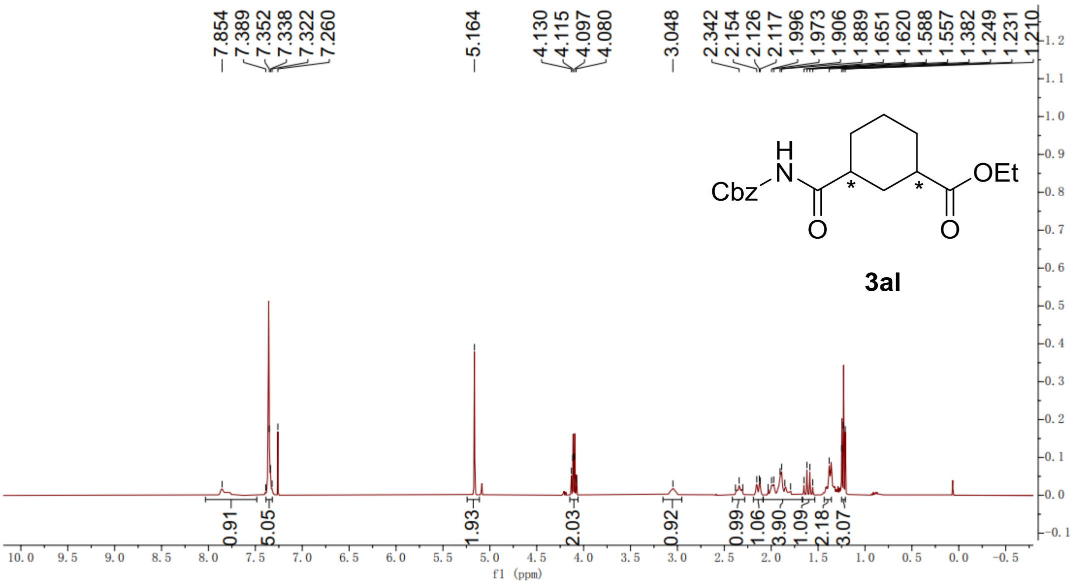

Supplementary Fig. 138. ^{1}H NMR Spectrum of **3al**

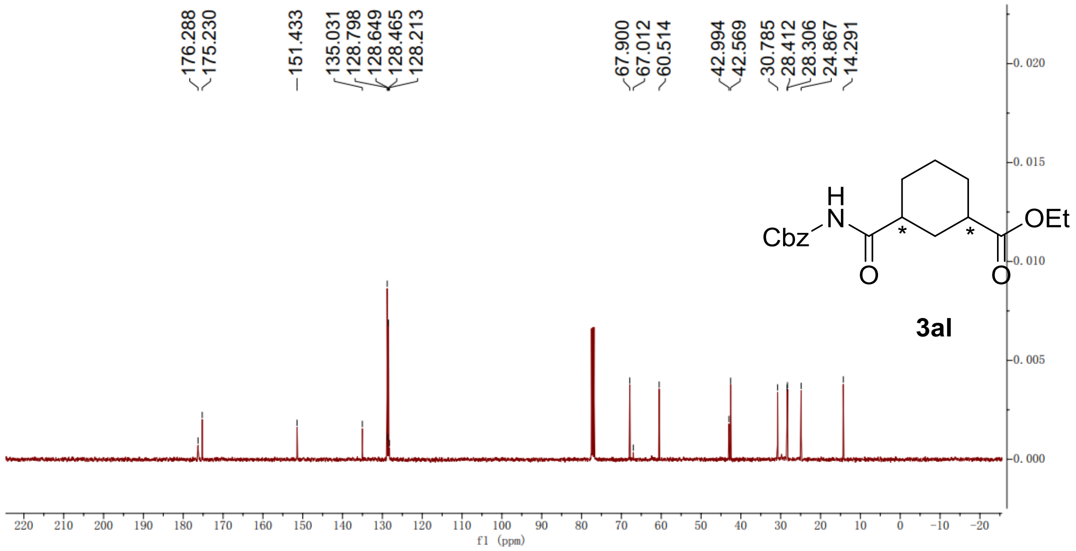

Supplementary Fig. 139. ^{13}C NMR Spectrum of **3al**

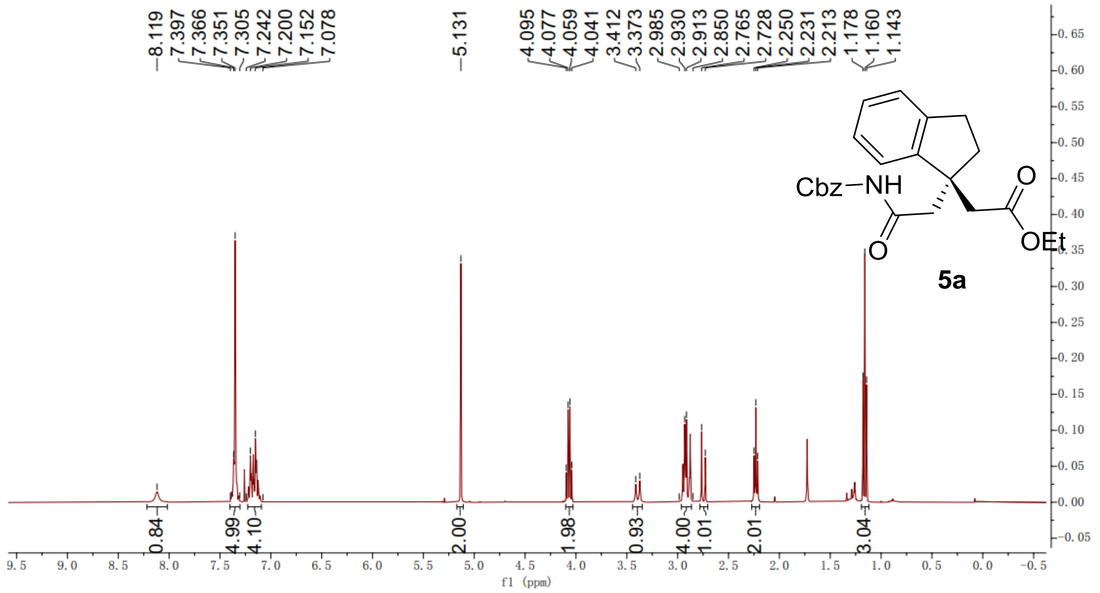

Supplementary Fig. 140. ^{1}H NMR Spectrum of **5a**

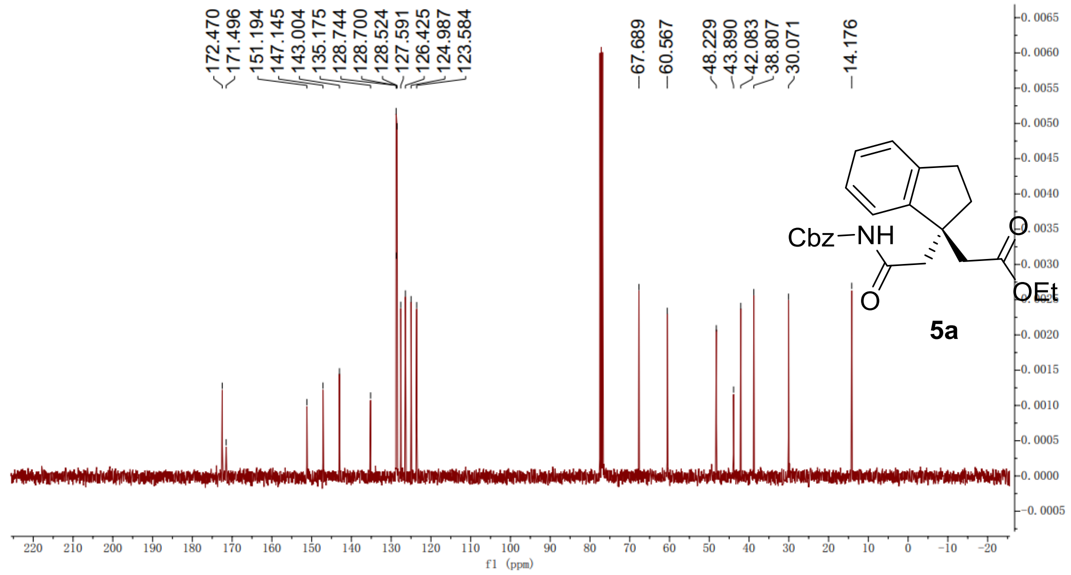

Supplementary Fig. 141. ^{13}C NMR Spectrum of **5a**

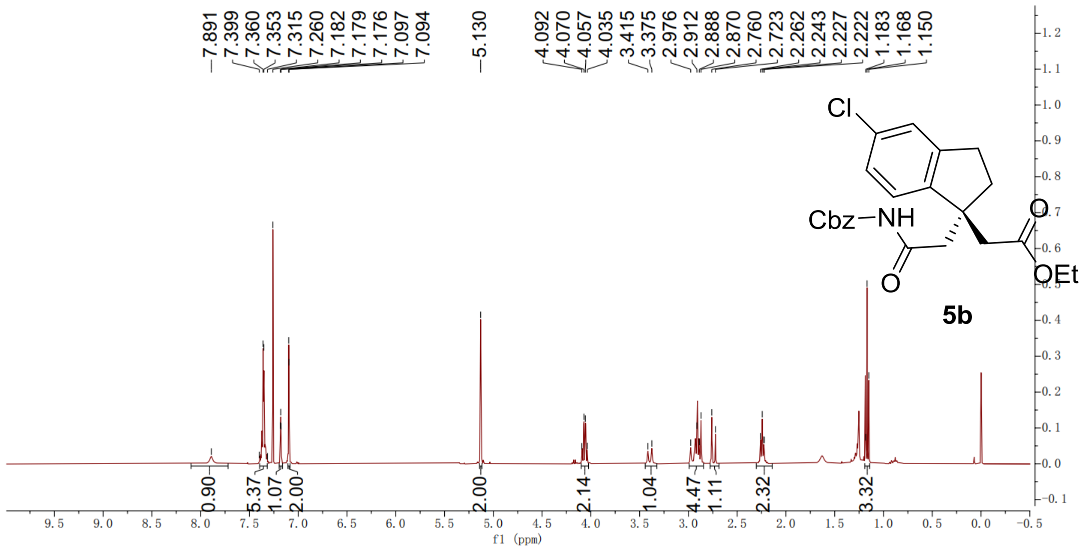

Supplementary Fig. 142. ^{1}H NMR Spectrum of **5b**

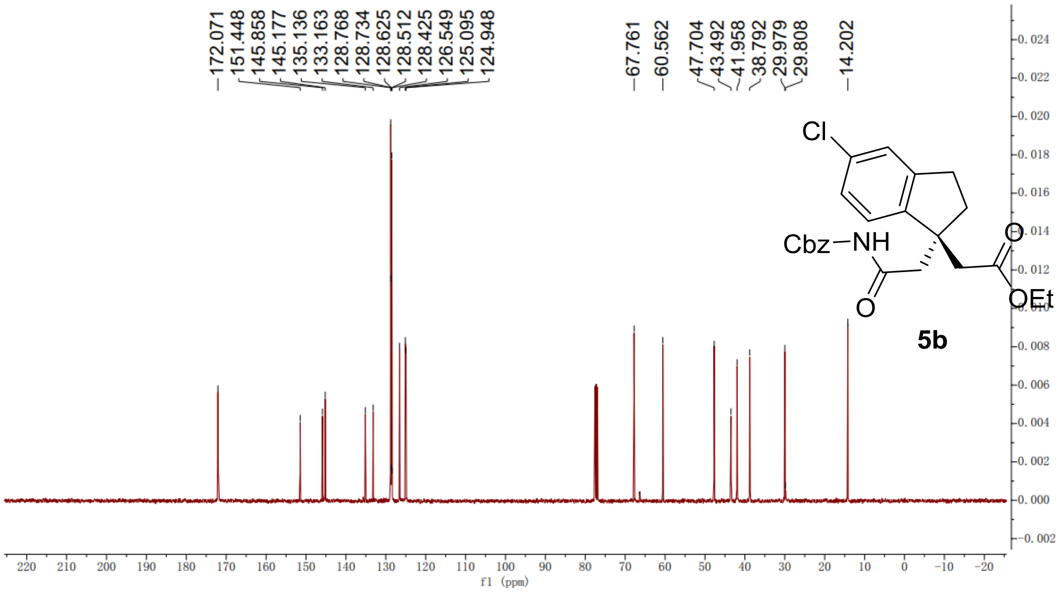

Supplementary Fig. 143. ^{13}C NMR Spectrum of **5b**

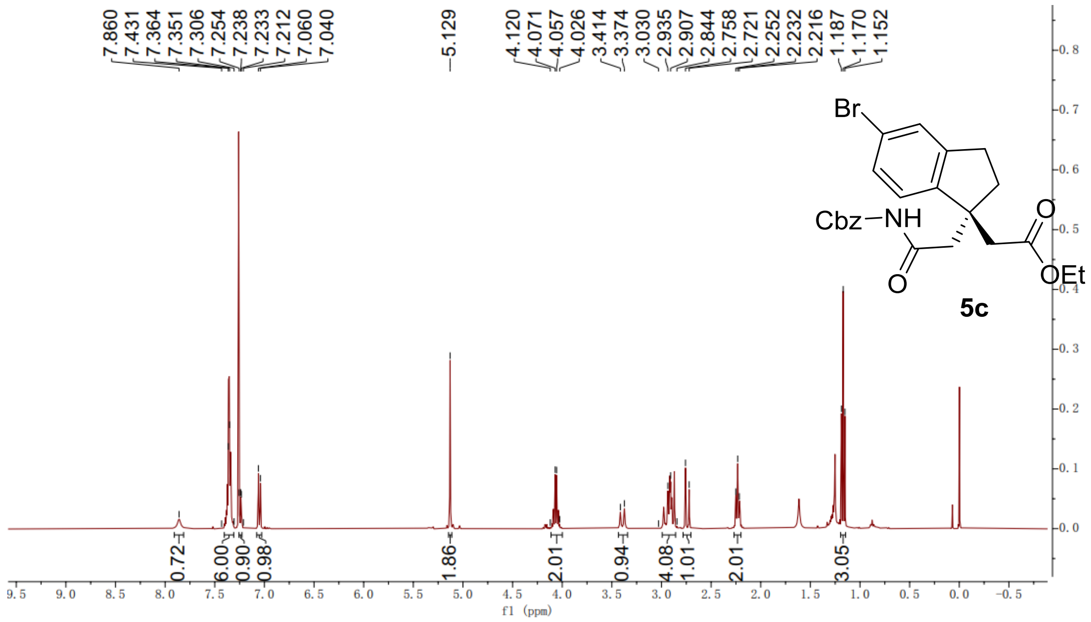

Supplementary Fig. 144. ^{1}H NMR Spectrum of **5c**

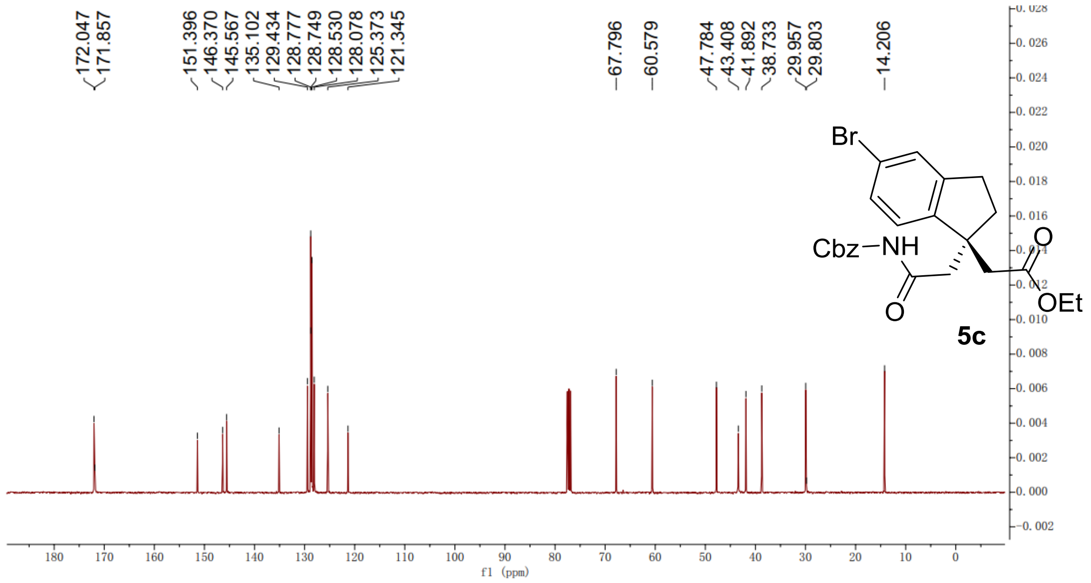

Supplementary Fig. 145. ^{13}C NMR Spectrum of **5c**

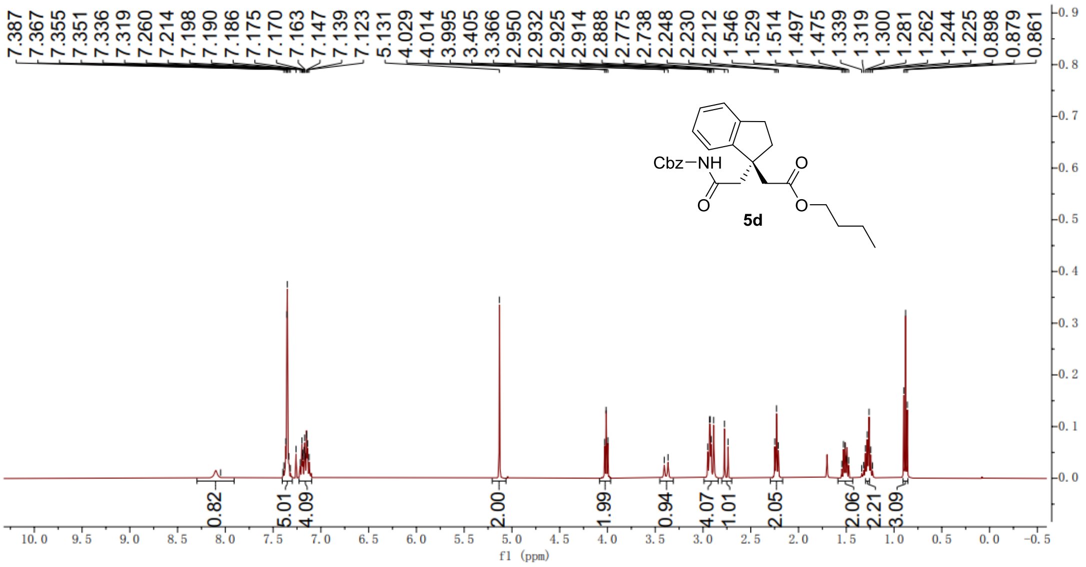

Supplementary Fig. 146. ^{1}H NMR Spectrum of **5d**

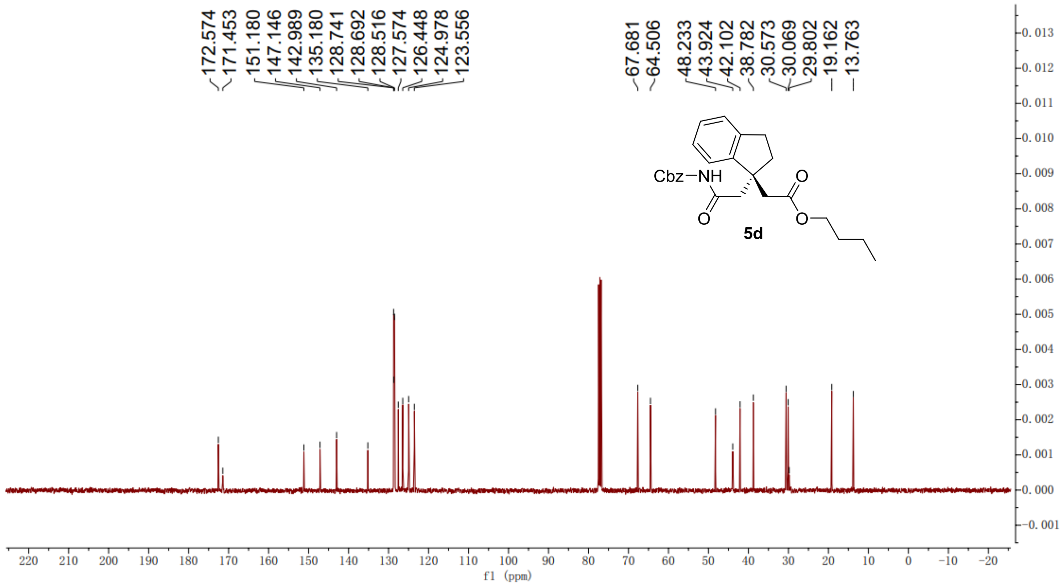

Supplementary Fig. 147. ^{13}C NMR Spectrum of **5d**

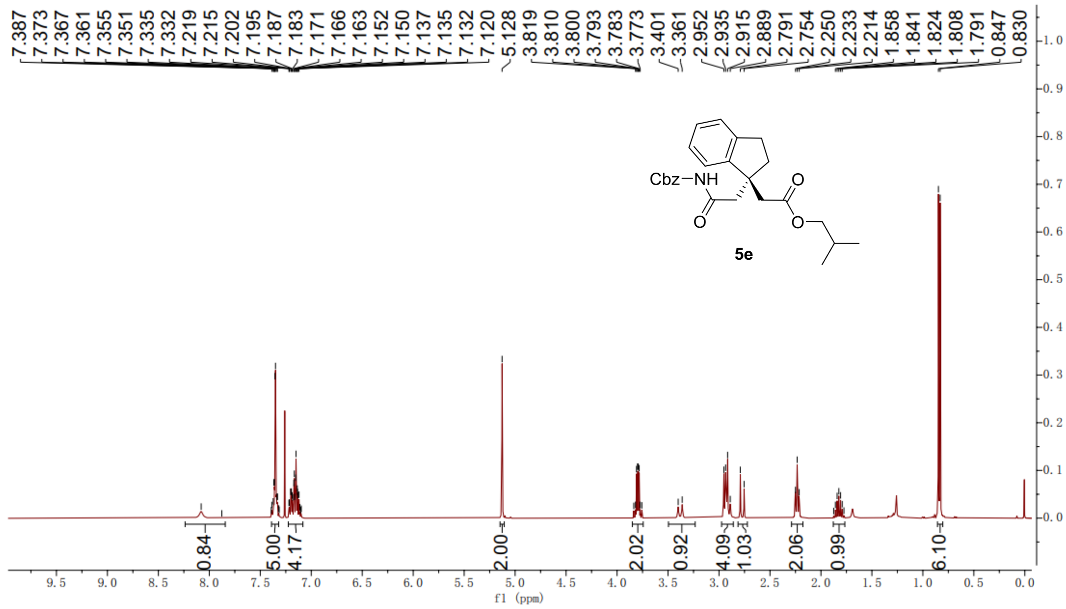

Supplementary Fig. 148. ^{1}H NMR Spectrum of **5e**

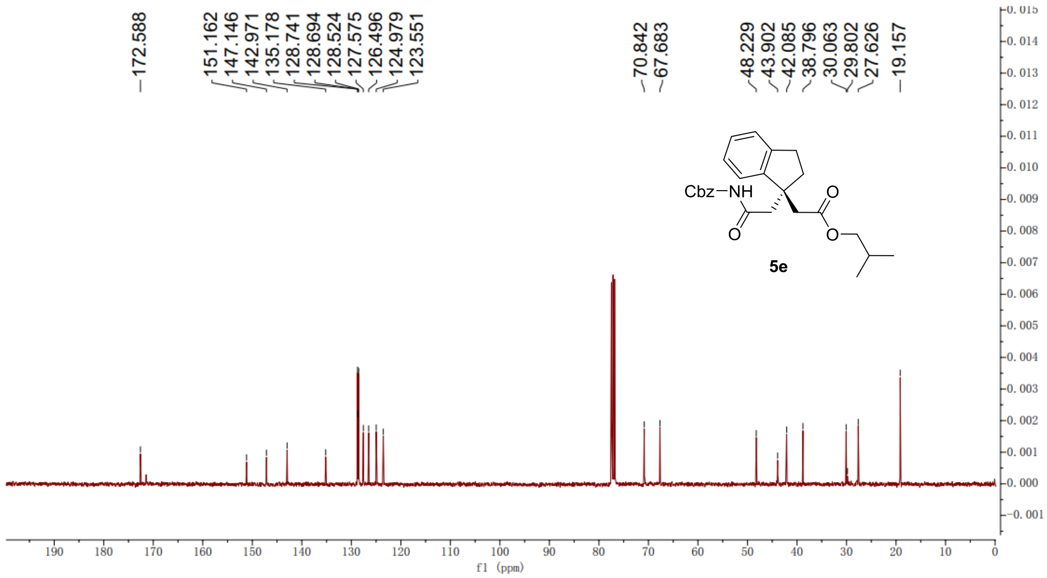

Supplementary Fig. 149. ^{13}C NMR Spectrum of **5e**

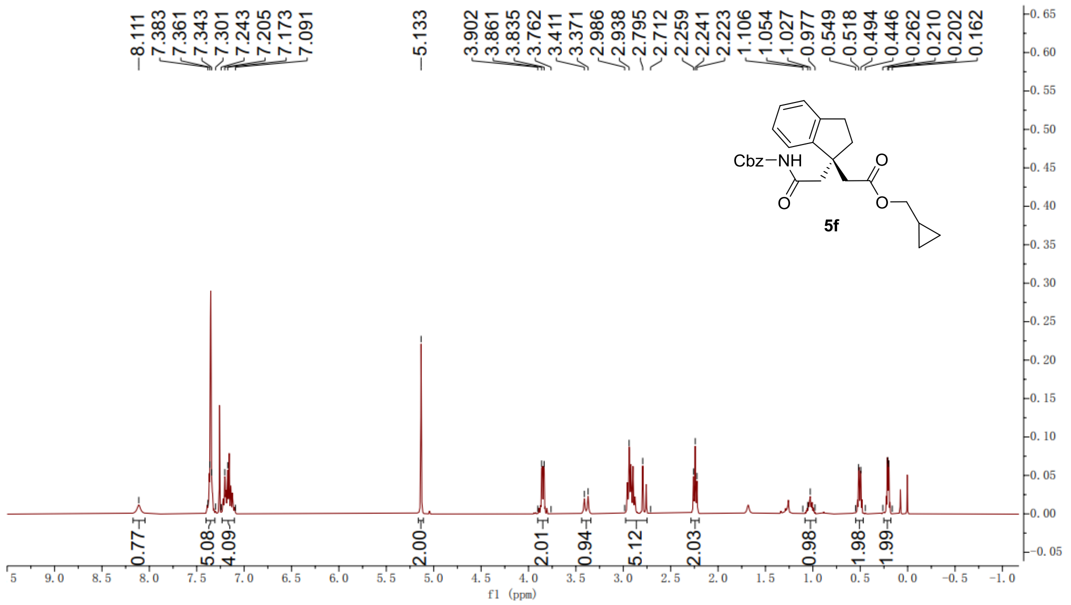

Supplementary Fig. 150. ^{1}H NMR Spectrum of **5f**

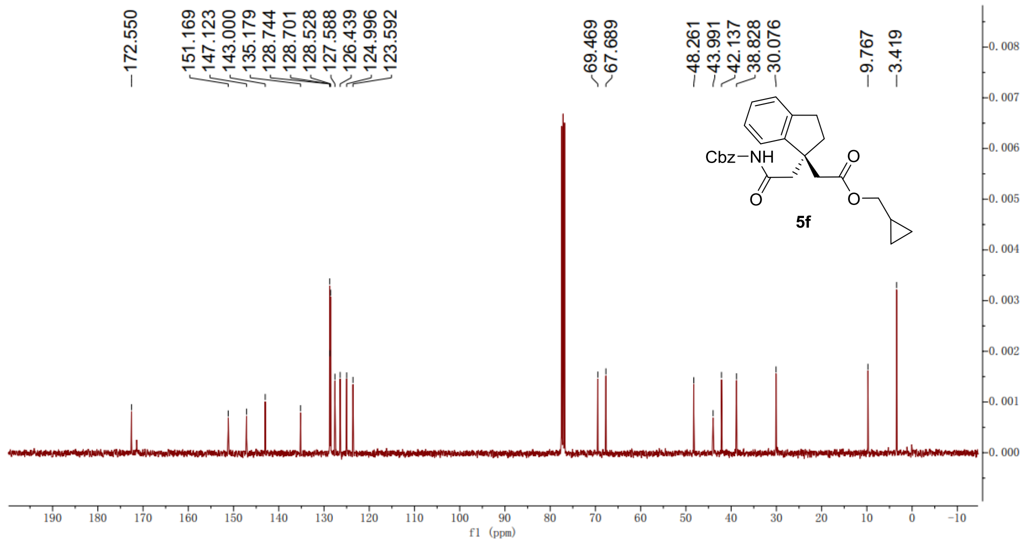

Supplementary Fig. 151. ^{13}C NMR Spectrum of **5f**

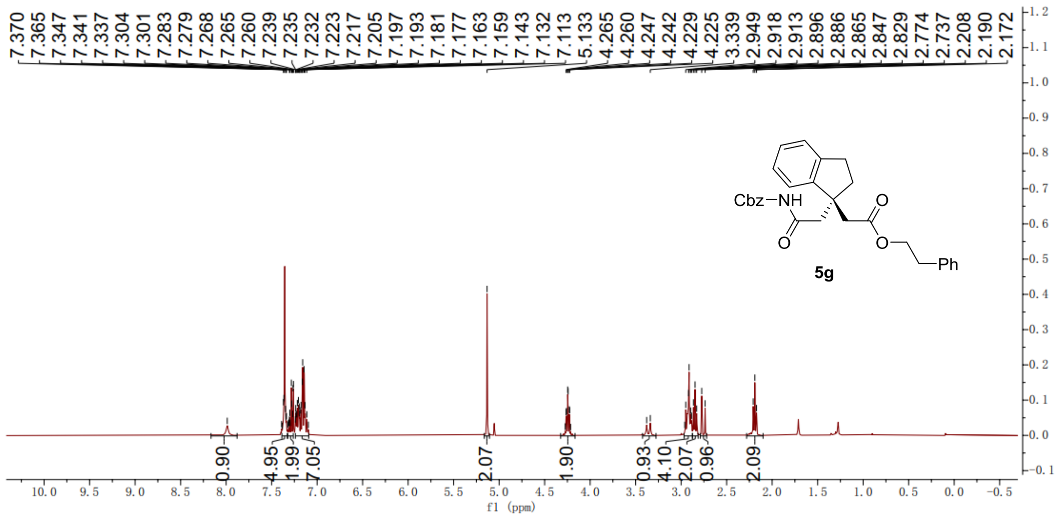

Supplementary Fig. 152. ^{1}H NMR Spectrum of **5g**

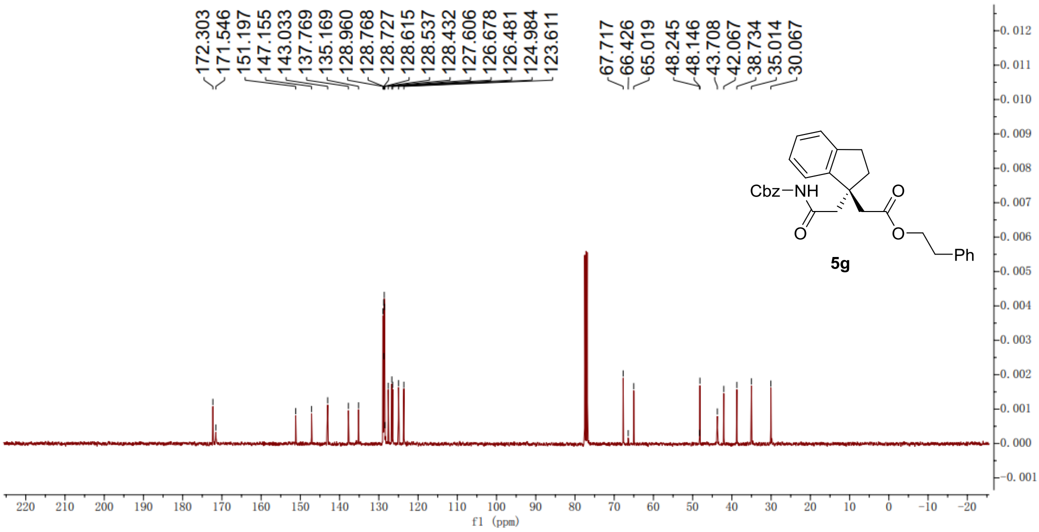

Supplementary Fig. 153. ^{13}C NMR Spectrum of **5g**

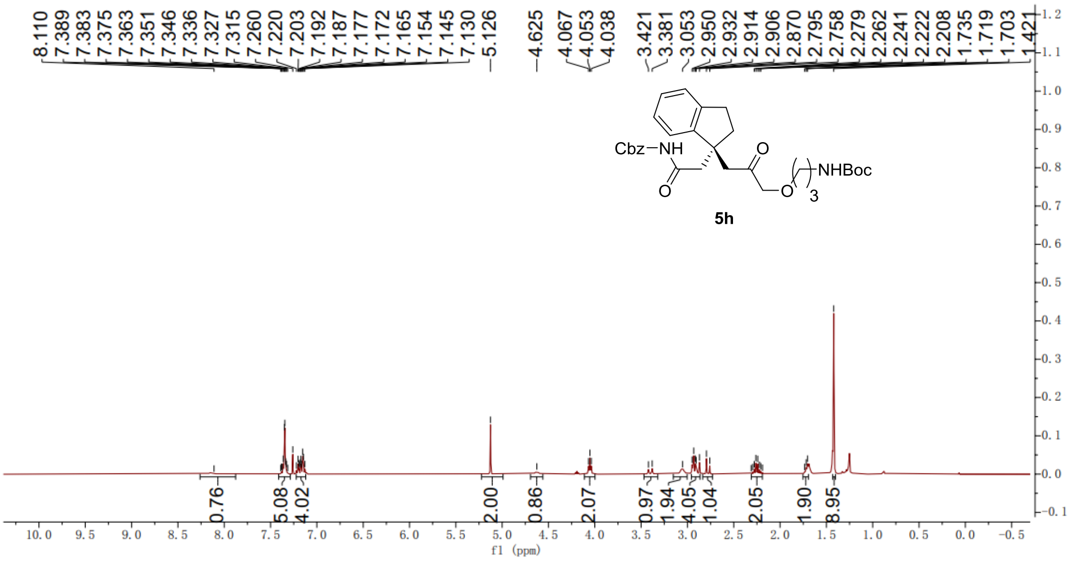

Supplementary Fig. 154. ^{1}H NMR Spectrum of **5h**

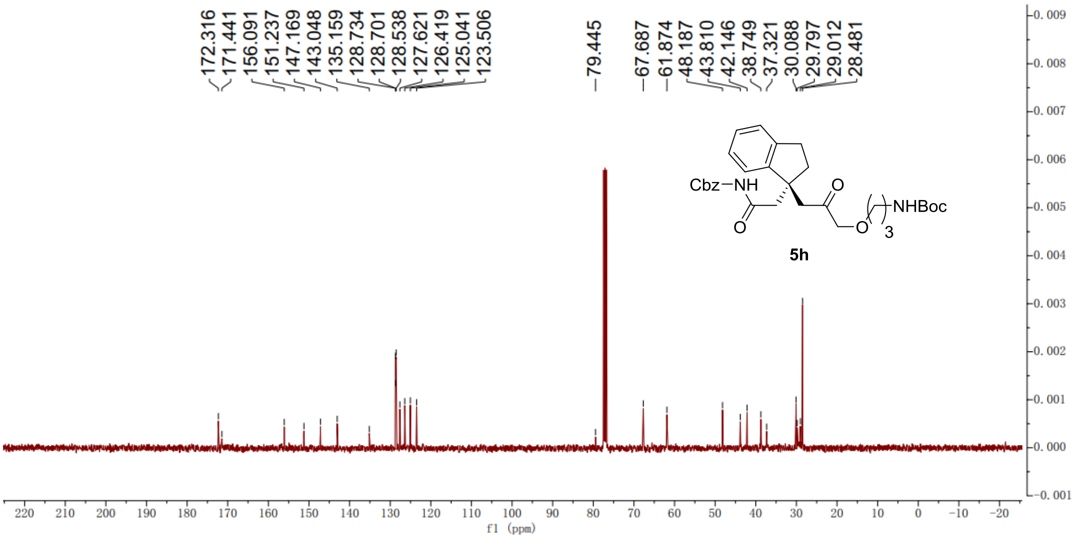

Supplementary Fig. 155. ^{13}C NMR Spectrum of **5h**

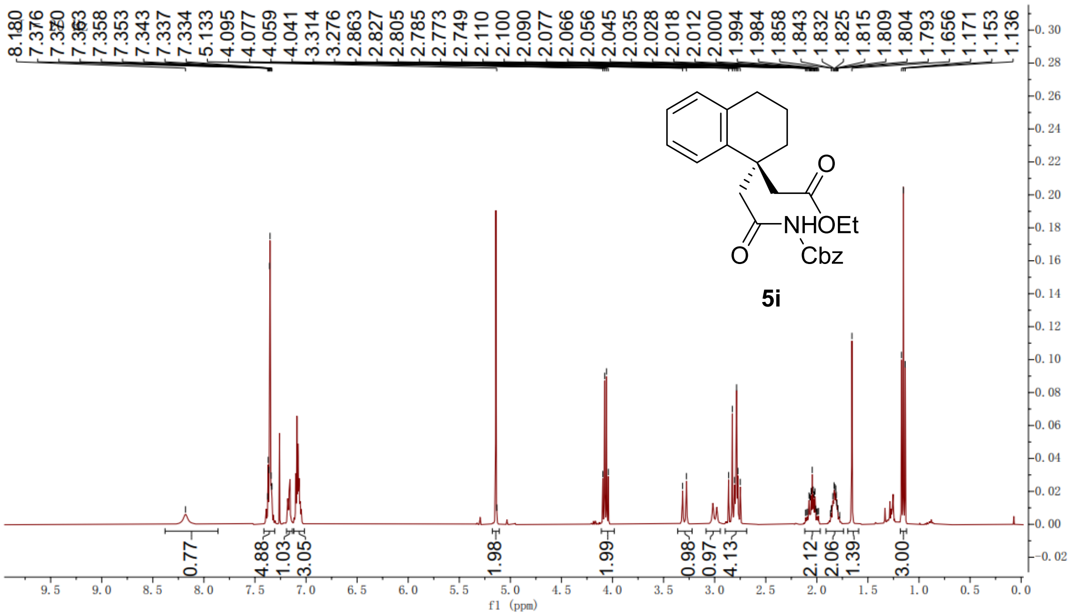

Supplementary Fig. 156. ^{1}H NMR Spectrum of **5i**

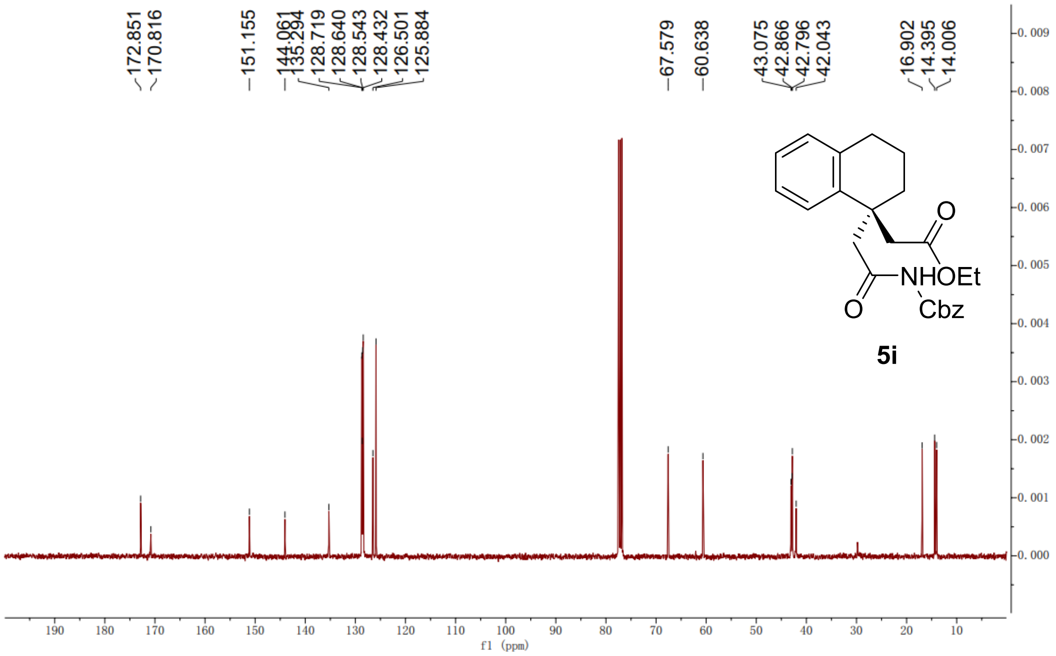

Supplementary Fig. 157. ^{13}C NMR Spectrum of **5i**

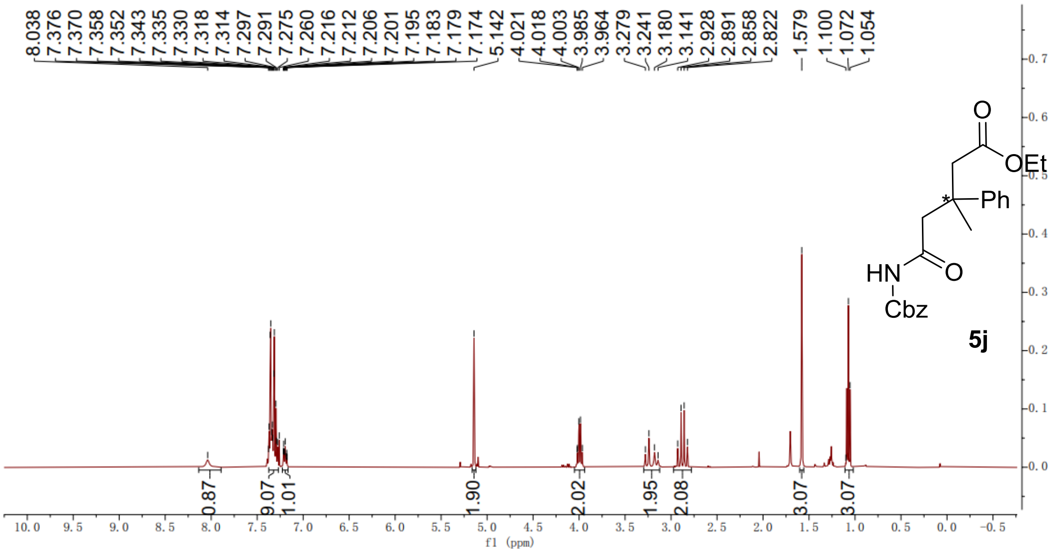

Supplementary Fig. 158. ^{1}H NMR Spectrum of **5j**

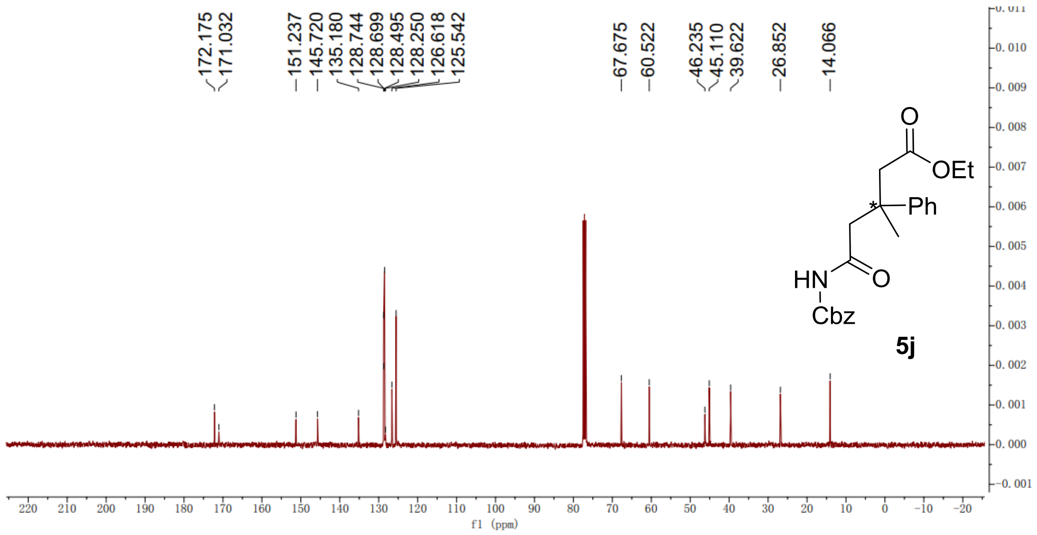

Supplementary Fig. 159. ^{13}C NMR Spectrum of **5j**

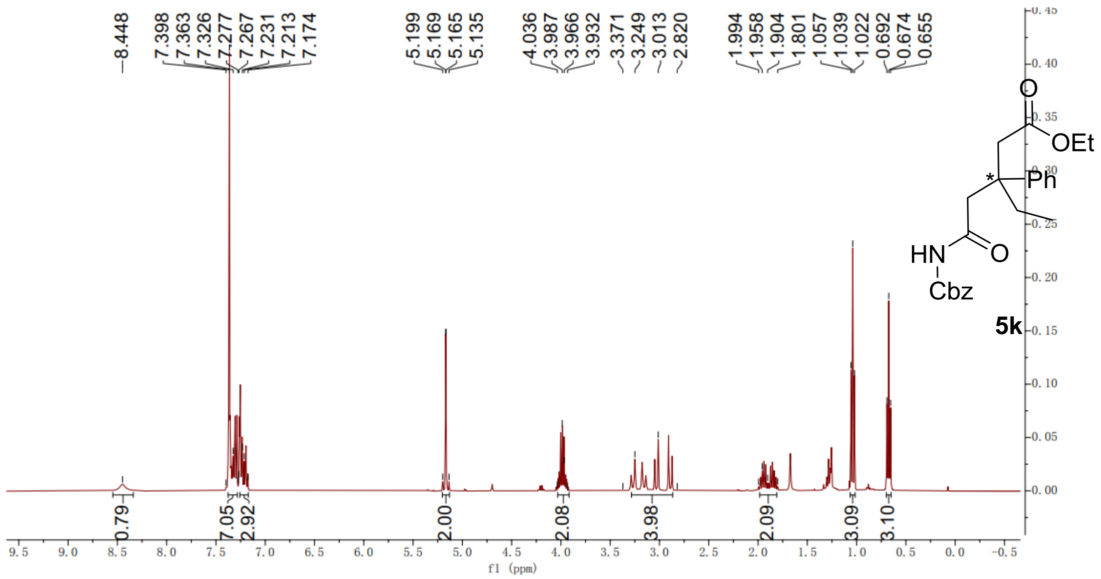

Supplementary Fig. 160. ^{1}H NMR Spectrum of **5k**

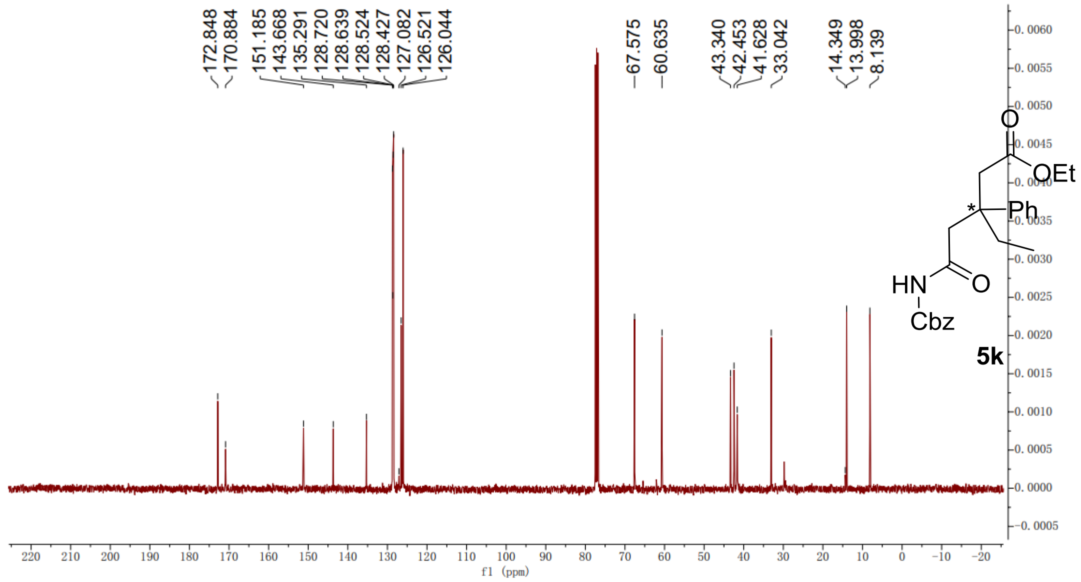

Supplementary Fig. 161. ^{13}C NMR Spectrum of **5k**

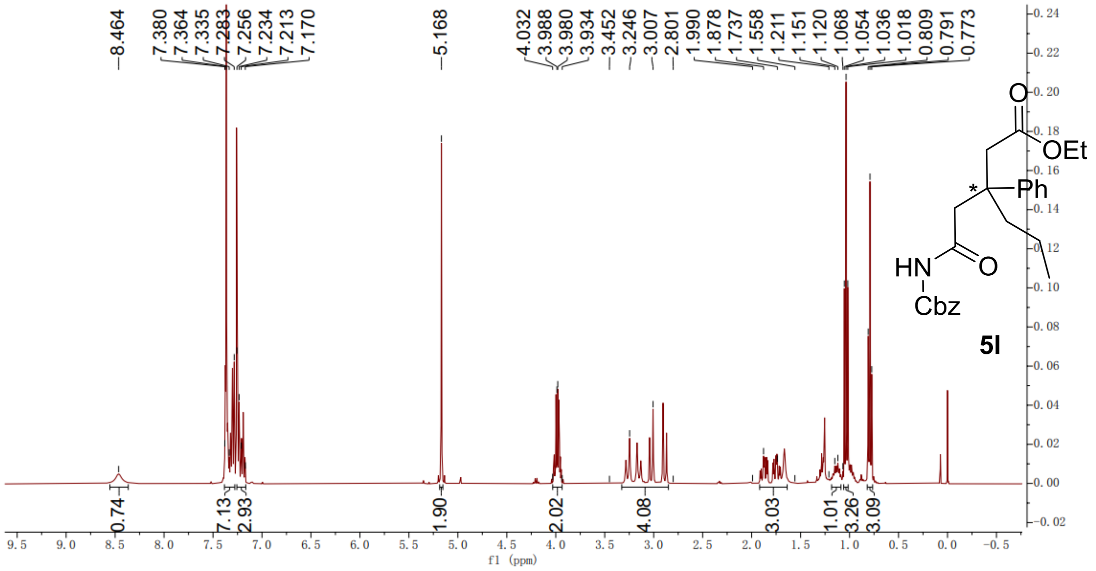

Supplementary Fig. 162. ^{1}H NMR Spectrum of **5l**

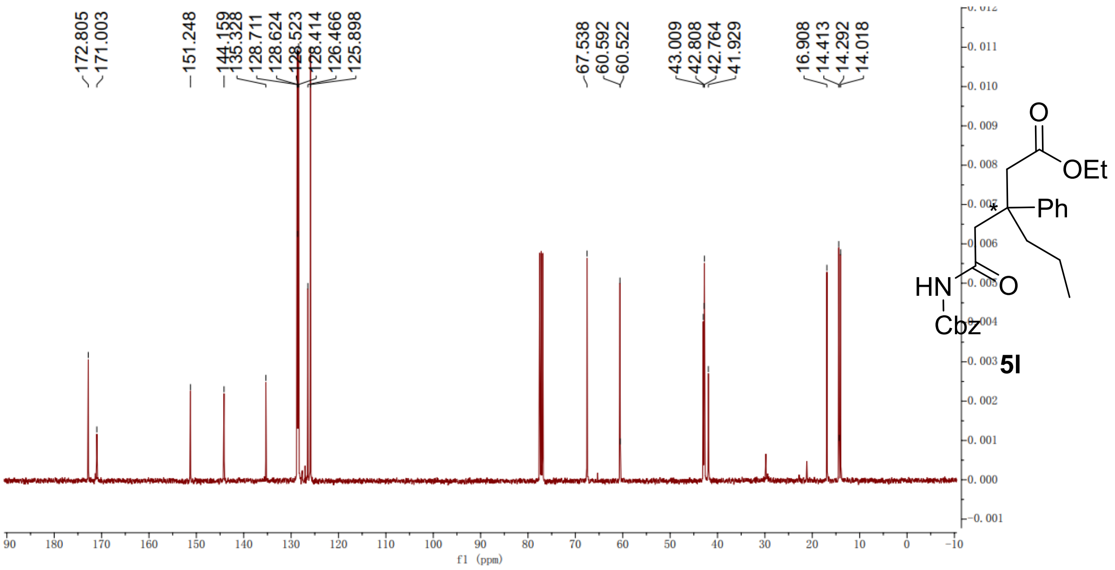

Supplementary Fig. 163. ^{13}C NMR Spectrum of **5l**

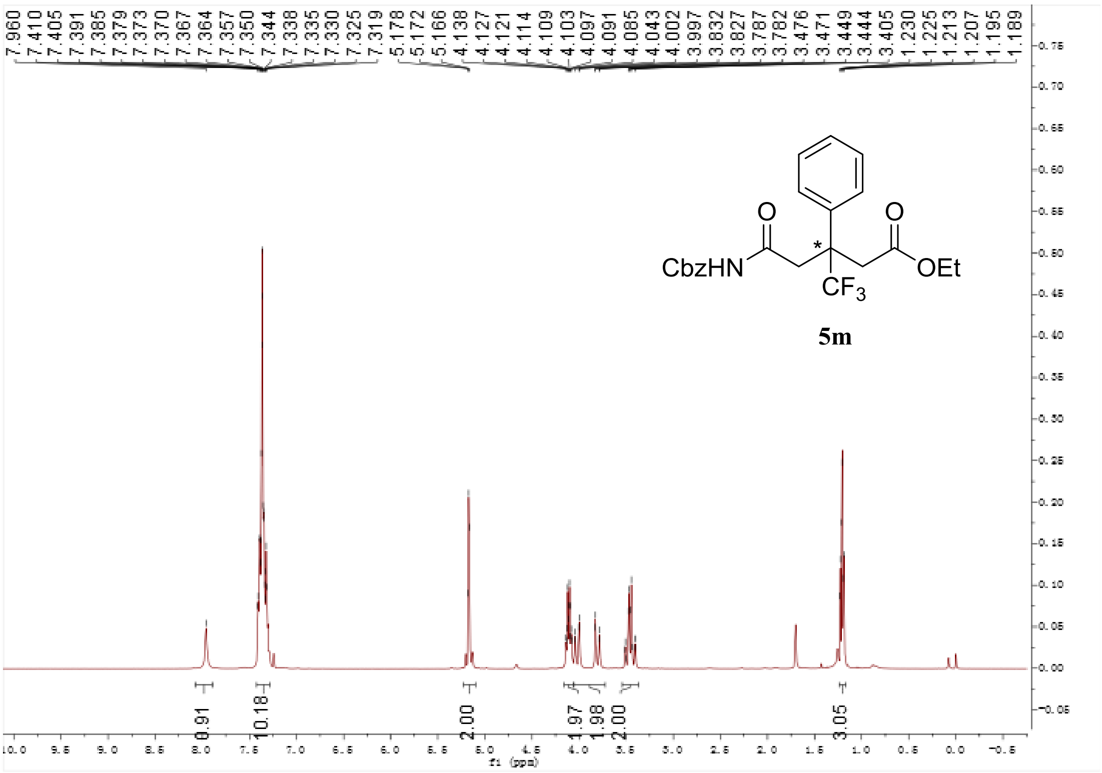

Supplementary Fig. 164. ^{1}H NMR Spectrum of **5m**

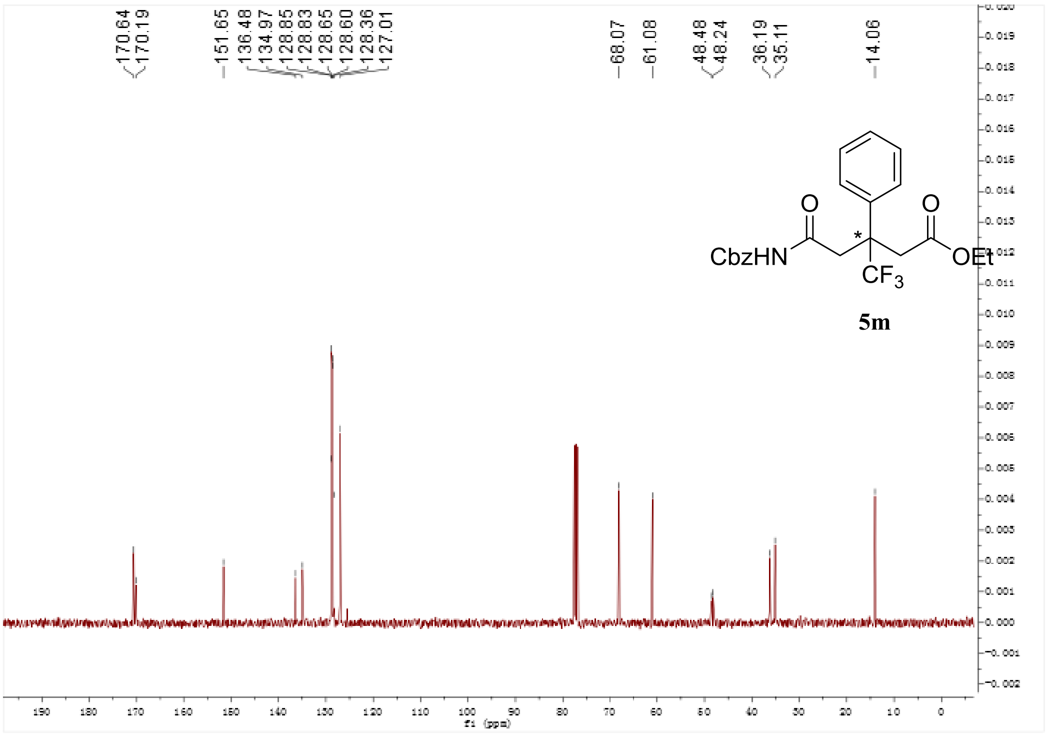

Supplementary Fig. 165. ^{13}C NMR Spectrum of **5m**

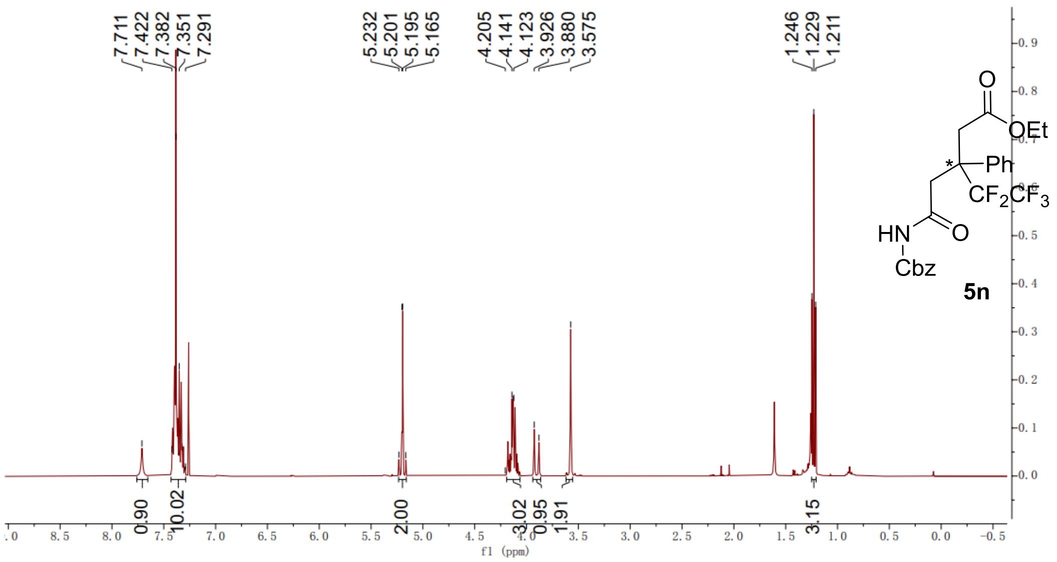

Supplementary Fig. 166. ^{1}H NMR Spectrum of **5n**

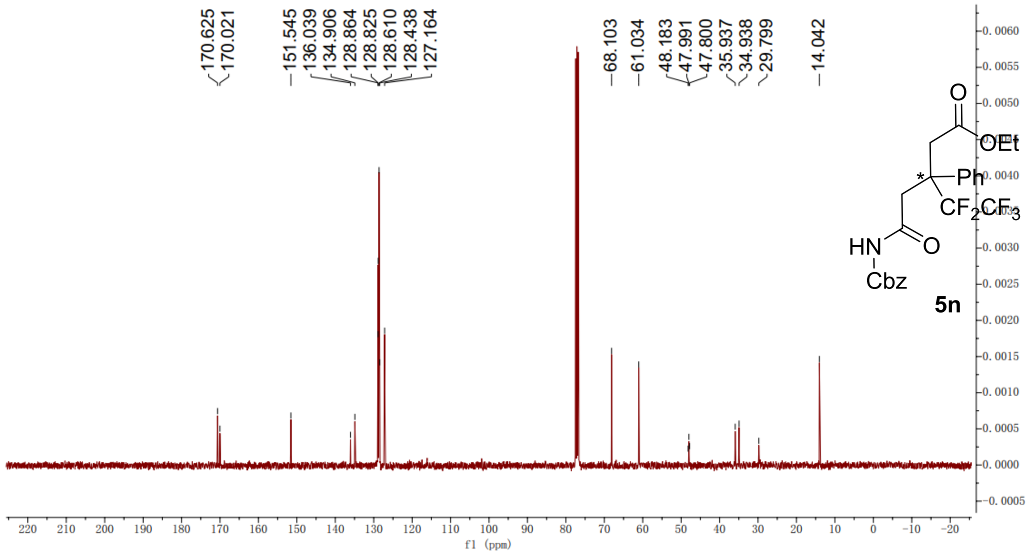

Supplementary Fig. 167. ^{13}C NMR Spectrum of **5n**

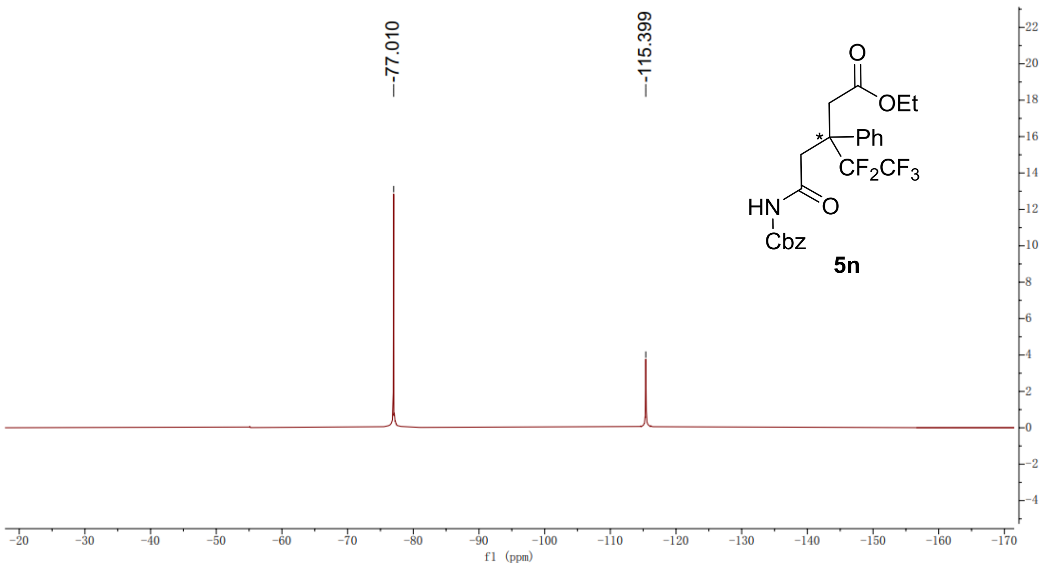

Supplementary Fig. 168. ^{19}F NMR Spectrum of **5n**

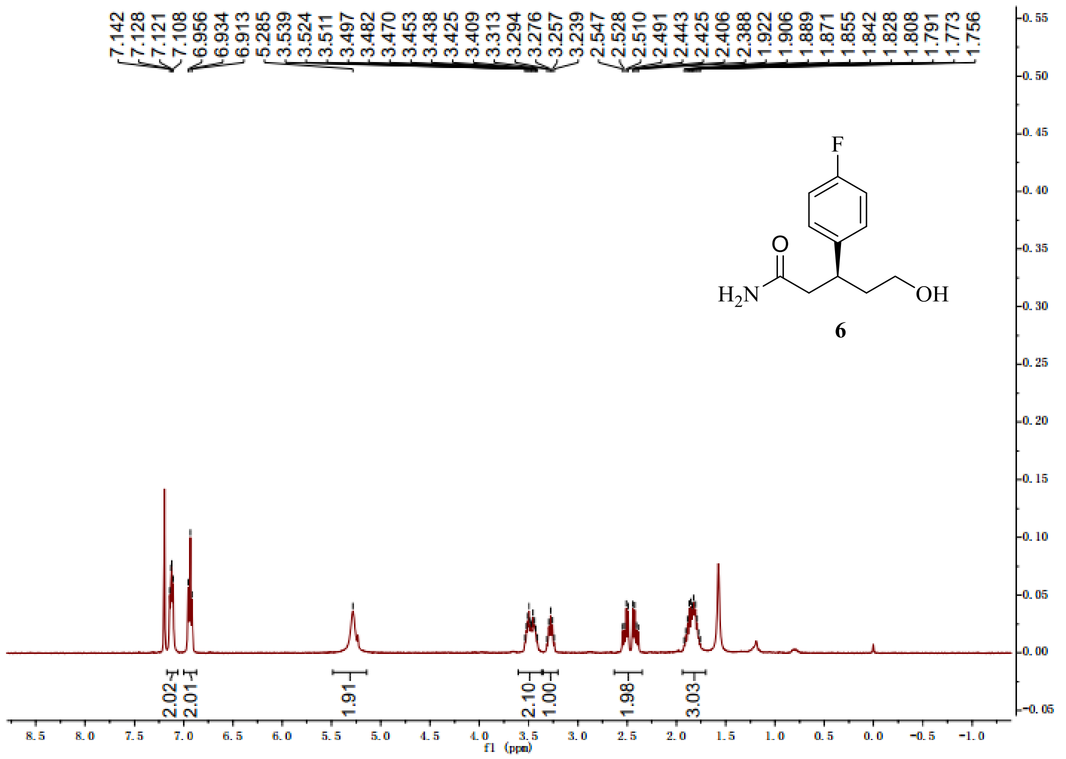

Supplementary Fig. 169. ^{1}H NMR Spectrum of **6**

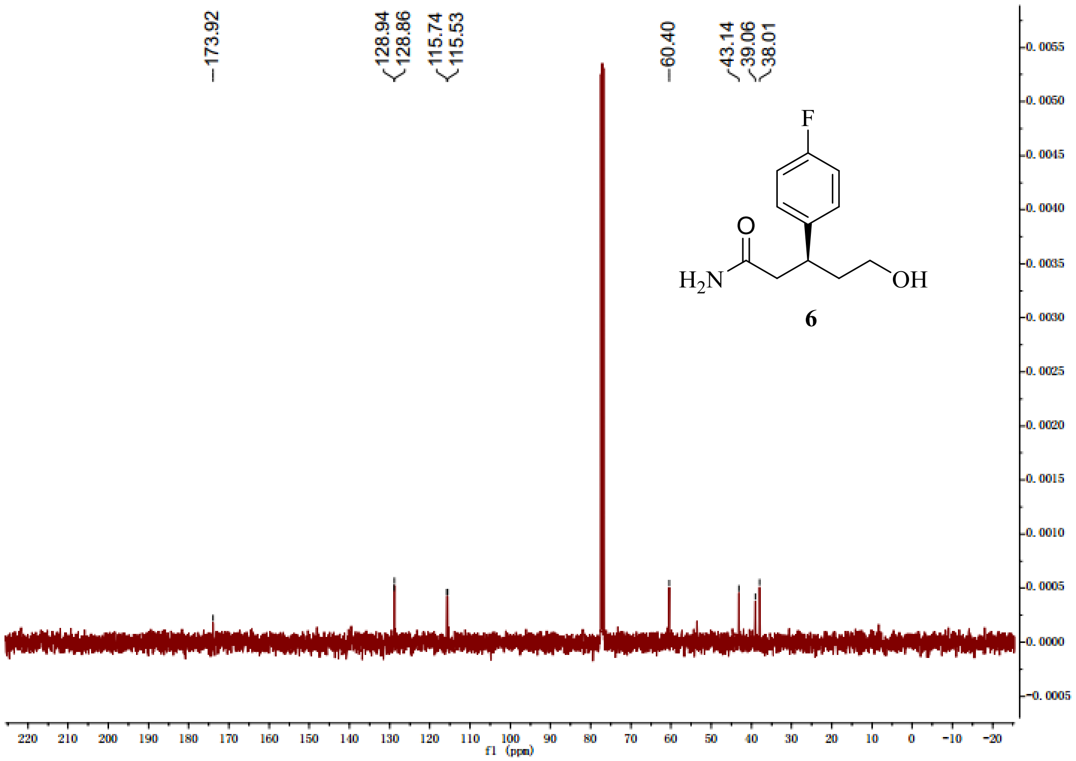

Supplementary Fig. 170. ^{13}C NMR Spectrum of **6**

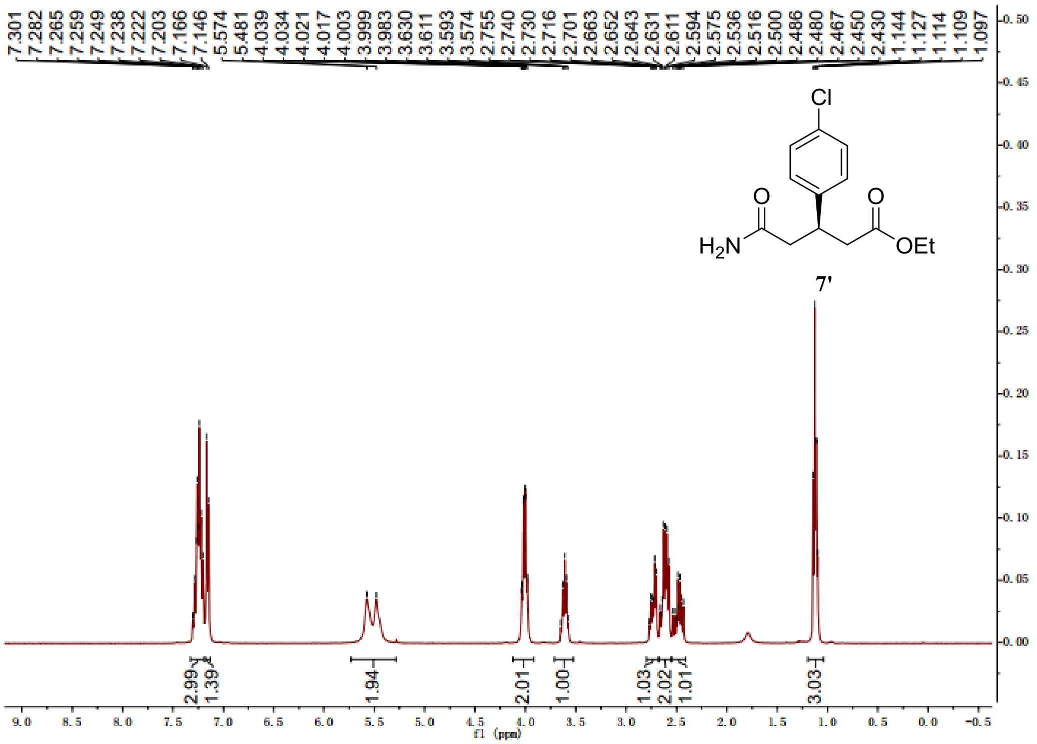

Supplementary Fig. 171. ^{1}H NMR Spectrum of **7'**

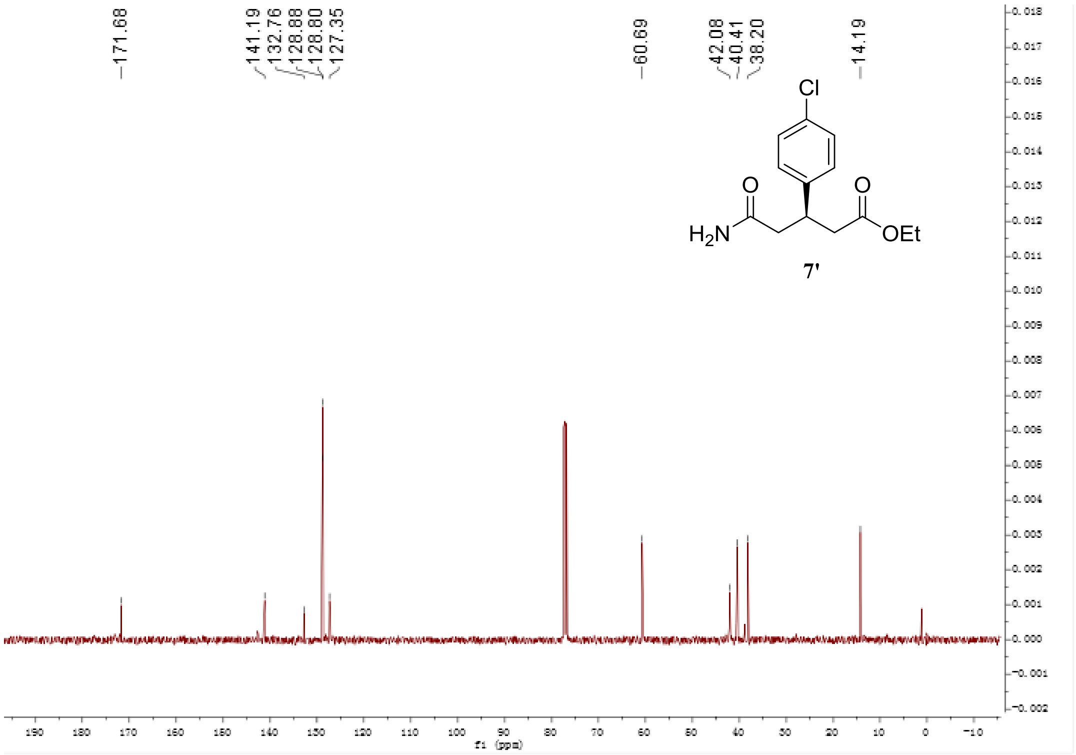

Supplementary Fig. 172. ^{13}C NMR Spectrum of **7'**

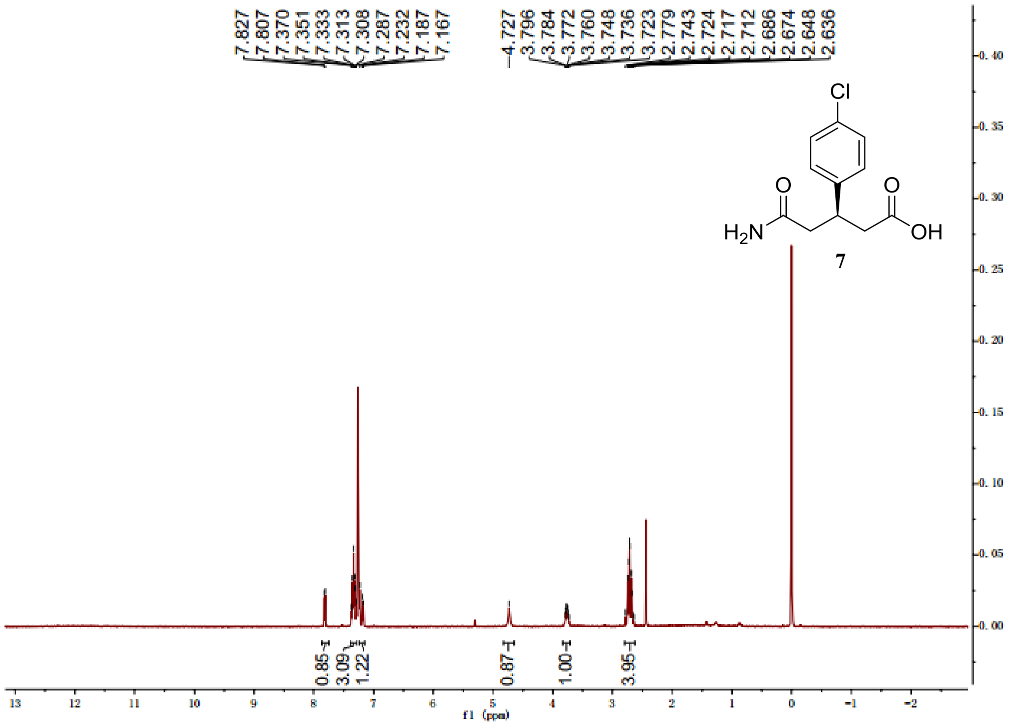

Supplementary Fig. 173. ^{1}H NMR Spectrum of **7**

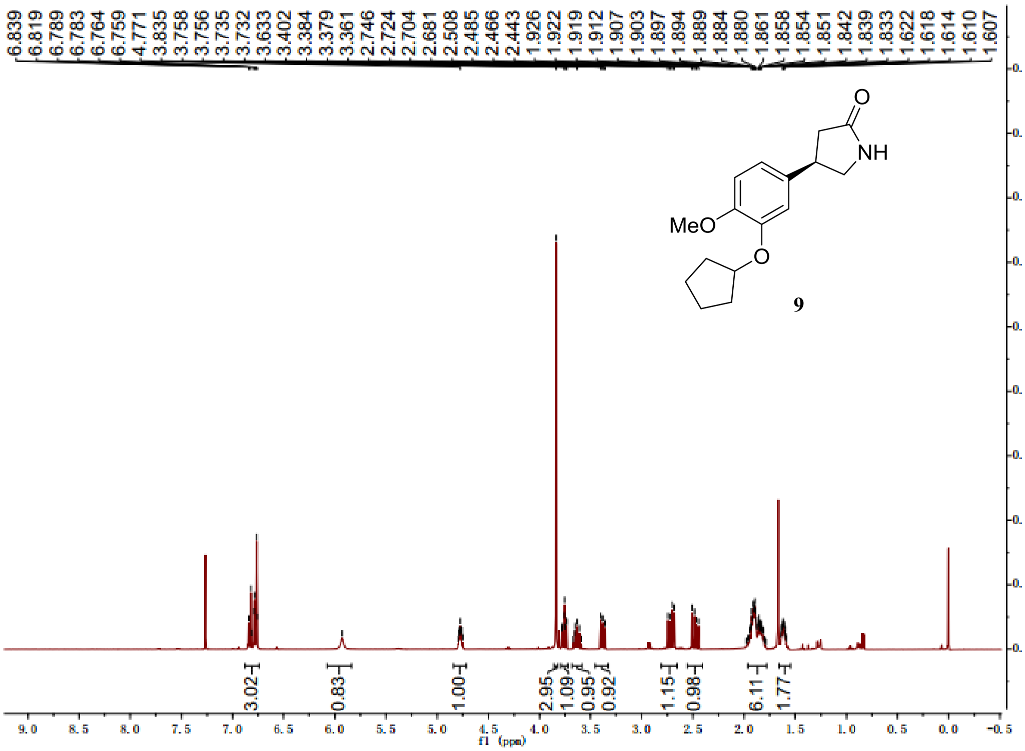

Supplementary Fig. 174. ^{1}H NMR Spectrum of **9**

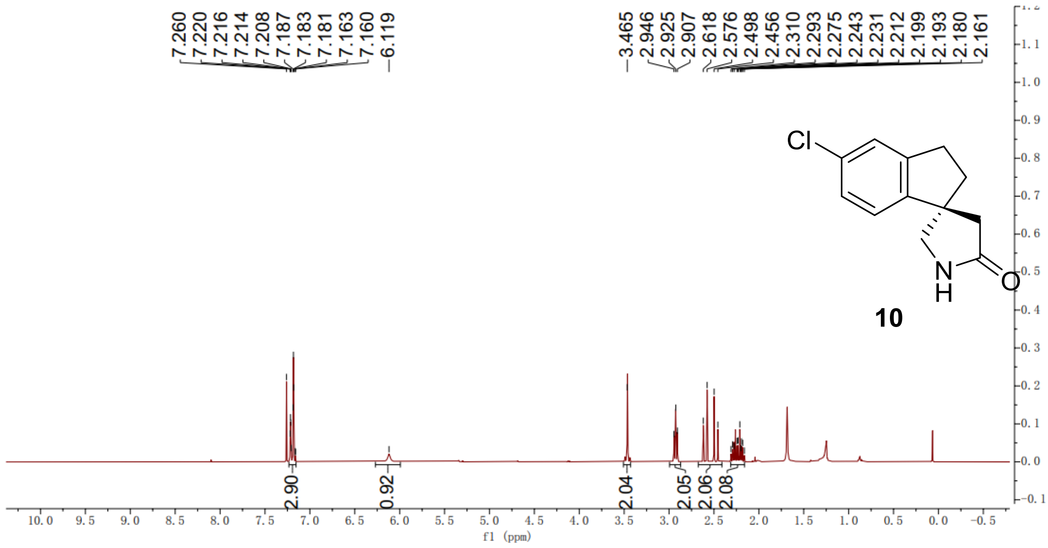

Supplementary Fig. 175. ^{1}H NMR Spectrum of **10**

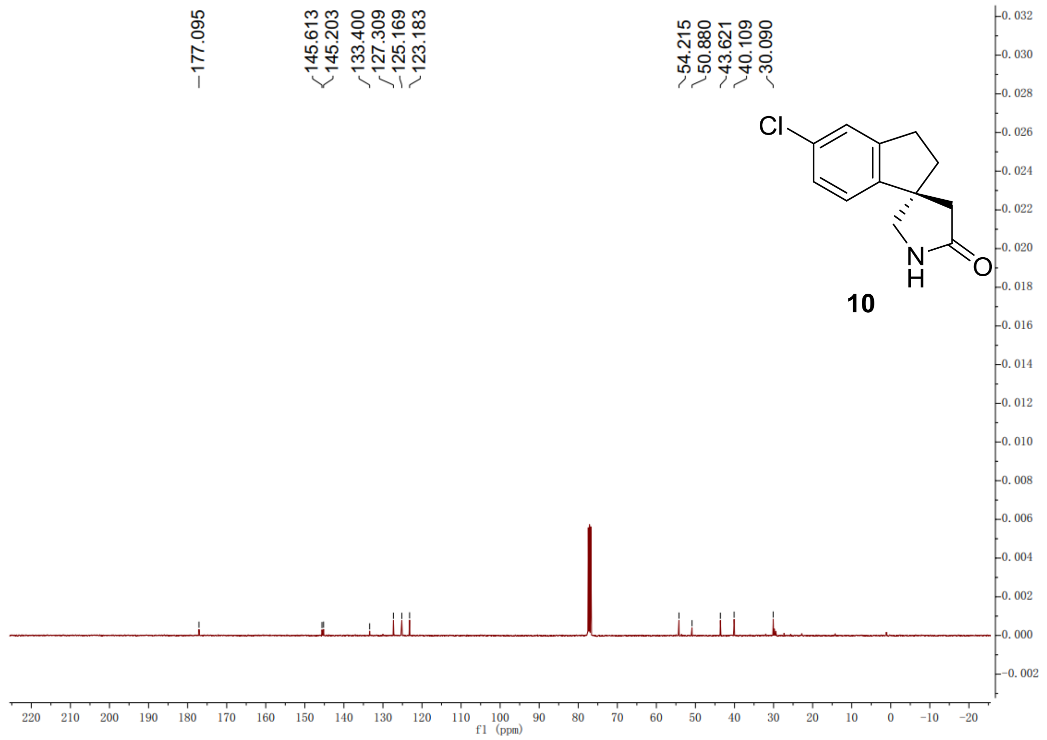

Supplementary Fig. 176. ^{13}C NMR Spectrum of **10**

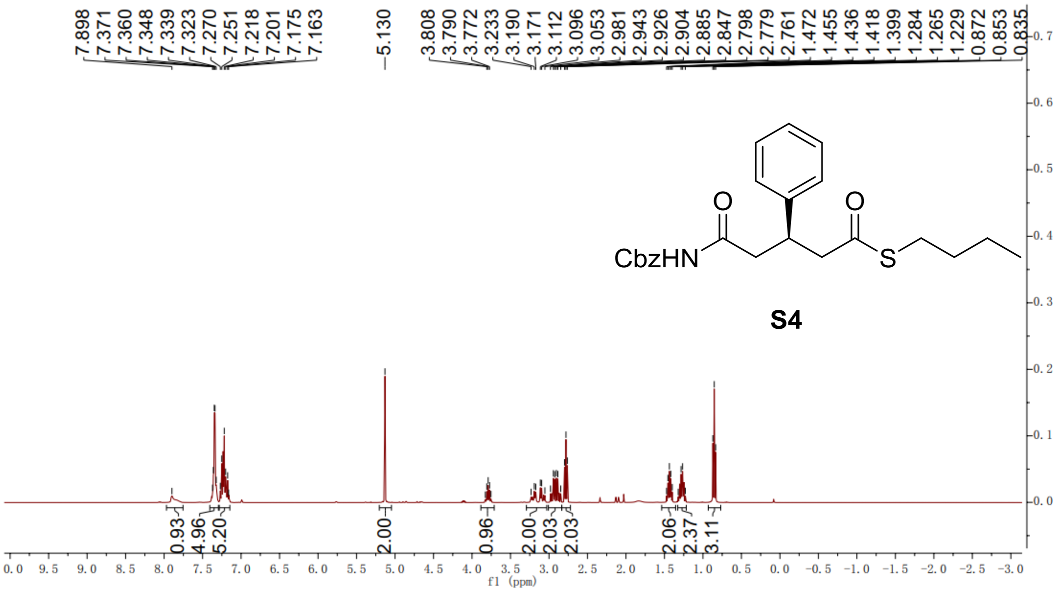

Supplementary Fig. 177. ^{1}H NMR Spectrum of **S4**

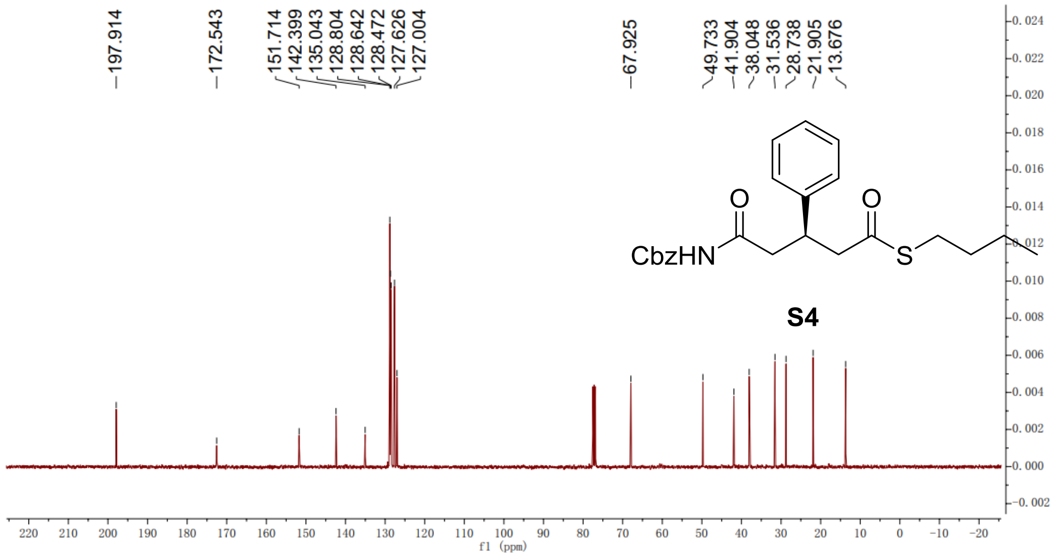

Supplementary Fig. 178. ^{13}C NMR Spectrum of **S4**

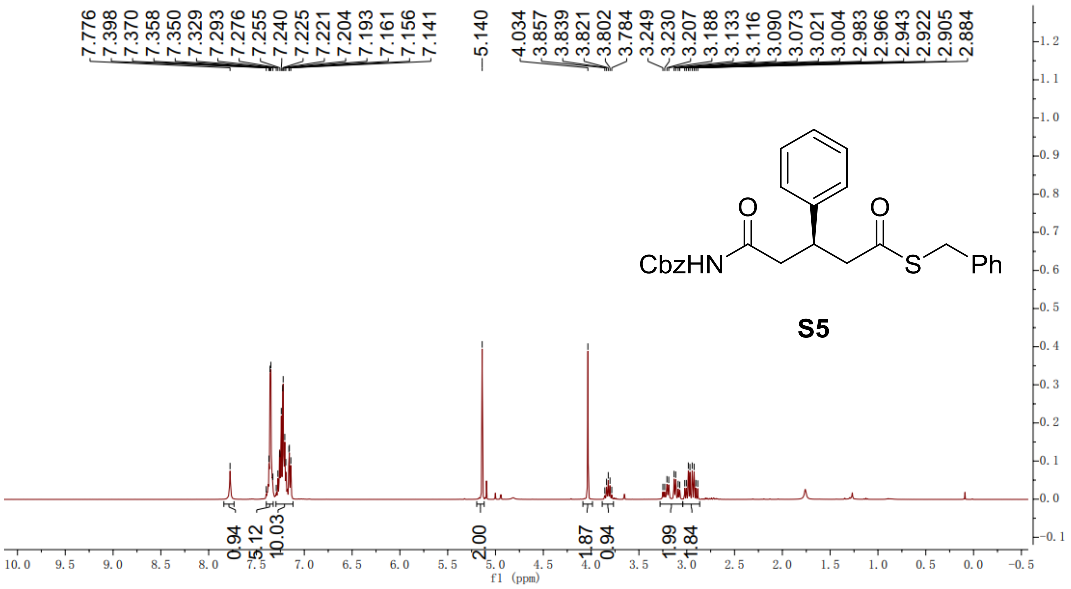

Supplementary Fig. 179. ^{1}H NMR Spectrum of **S5**

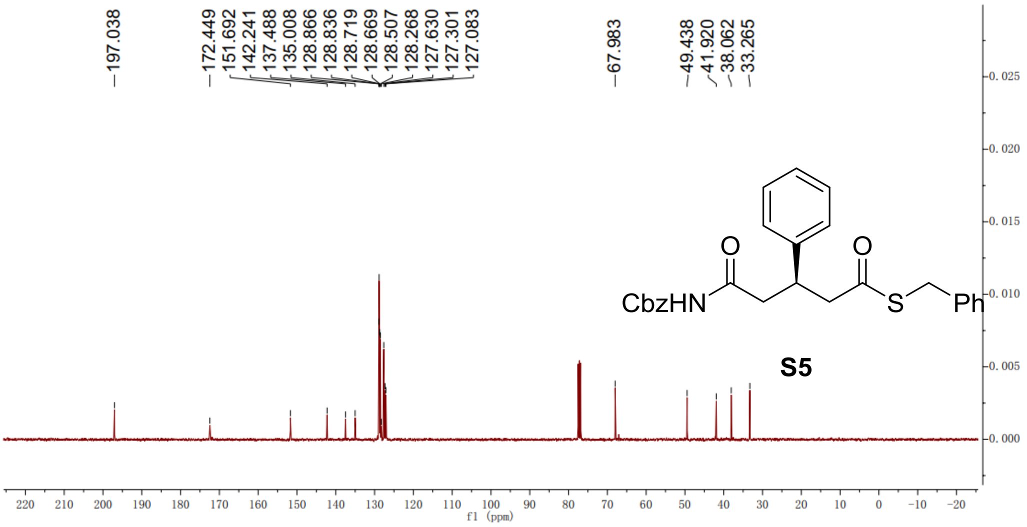

Supplementary Fig. 180. ^{13}C NMR Spectrum of **S5**

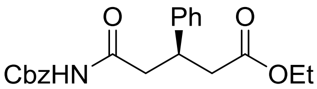

3a

<Chromatogram>

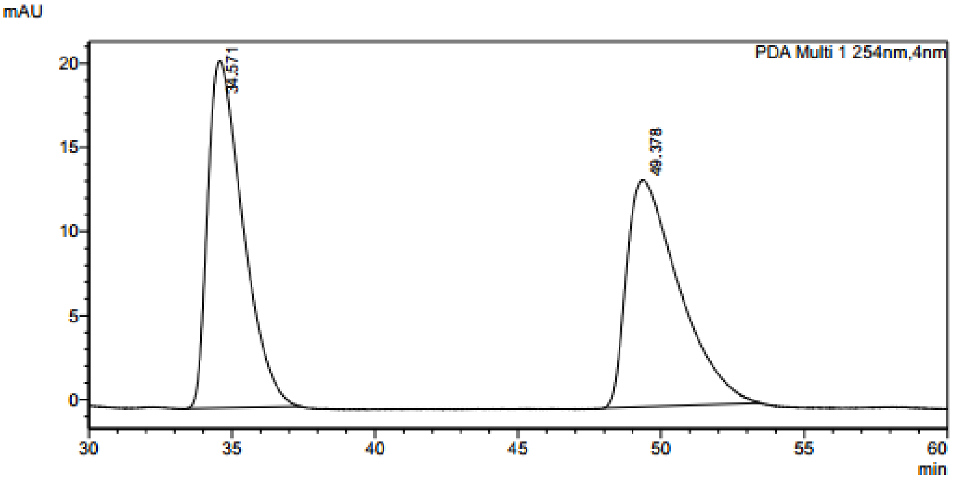

<Peak Table>

PDA Ch1 254nm

Peak#	Ret. Time	Area	Height	Area%
1	34.571	1771393	20634	50.659
2	49.378	1725290	13466	49.341
Total		3496684	34100	100.000

Supplementary Fig. 181. HPLC Spectra of racemic **3a**

<Chromatogram>

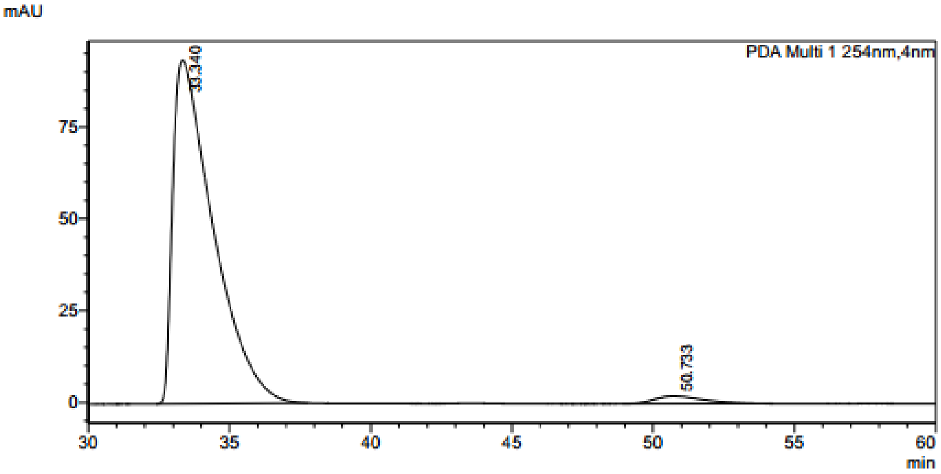

<Peak Table>

PDA Ch1 254nm

Peak#	Ret. Time	Area	Height	Area%
1	33.340	9334511	93525	97.507
2	50.733	238643	2013	2.493
Total		9573155	95538	100.000

Supplementary Fig. 182. HPLC Spectra of **3a**

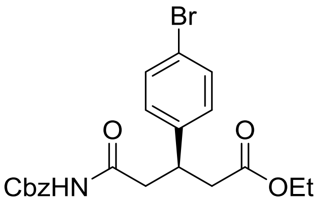

3b

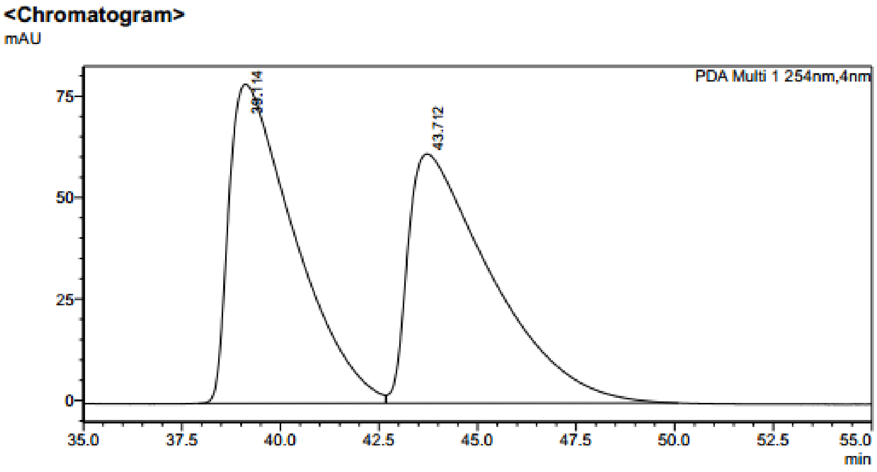

<Peak Table>

PDA Ch1 254nm

Peak#	Ret. Time	Area	Height	Area%
1	39.114	9123015	78632	50.135
2	43.712	9073980	61426	49.865
Total		18196994	140058	100.000

Supplementary Fig. 183. HPLC Spectra of racemic **3b**

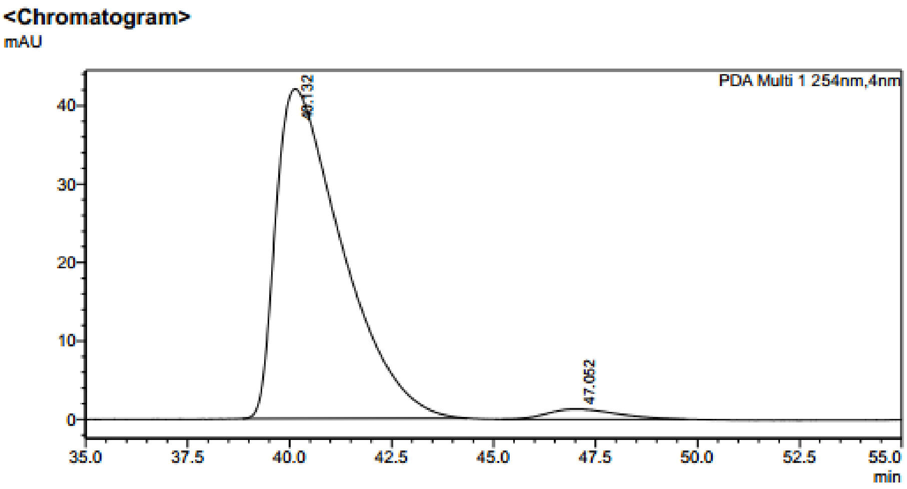

<Peak Table>

PDA Ch1 254nm

Peak#	Ret. Time	Area	Height	Area%
1	40.132	4761991	42031	96.885
2	47.052	153102	1296	3.115
Total		4915093	43326	100.000

Supplementary Fig. 184. HPLC Spectra of **3b**

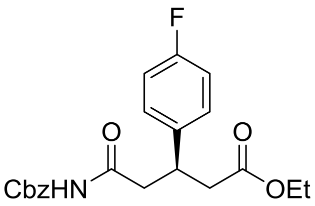

3c

<Chromatogram>

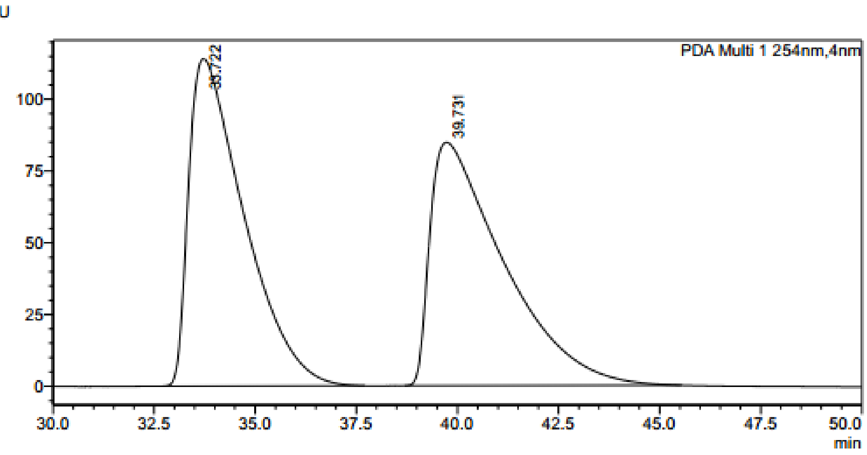

<Peak Table>

PDA Ch1 254nm

Peak#	Ret. Time	Area	Height	Area%
1	33.722	10877811	114116	50.286
2	39.731	10754271	84801	49.714
Total		21632082	198917	100.000

Supplementary Fig. 185. HPLC Spectra of racemic **3c**

<Chromatogram>

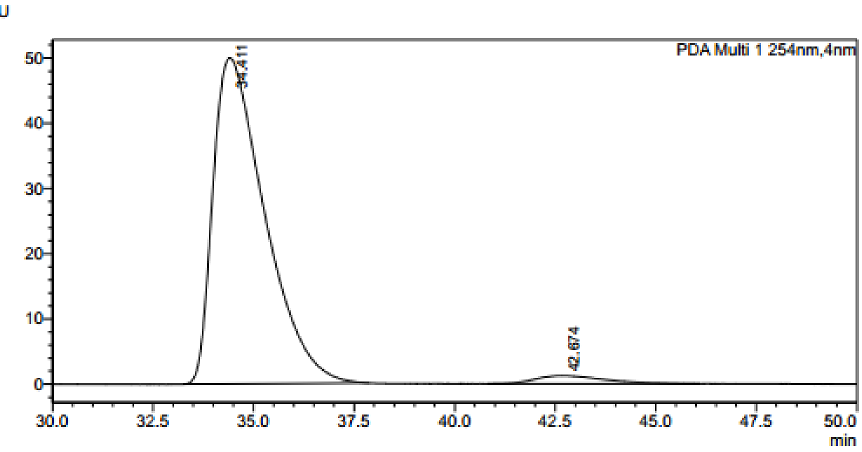

<Peak Table>

PDA Ch1 254nm

Peak#	Ret. Time	Area	Height	Area%
1	34.411	4475957	49988	96.895
2	42.674	143424	1232	3.105
Total		4619382	51219	100.000

Supplementary Fig. 186. HPLC Spectra of **3c**

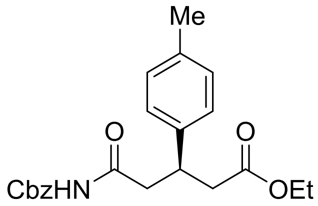

3d

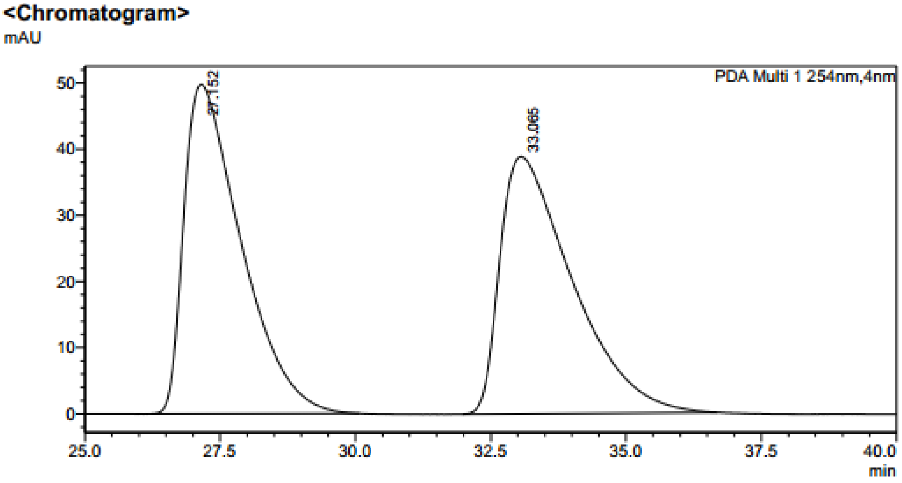

<Peak Table>

PDA Ch1 254nm

Peak#	Ret. Time	Area	Height	Area%
1	27.152	3660261	49736	50.254
2	33.065	3623265	38878	49.746
Total		7283526	88615	100.000

Supplementary Fig. 187. HPLC Spectra of racemic **3d**

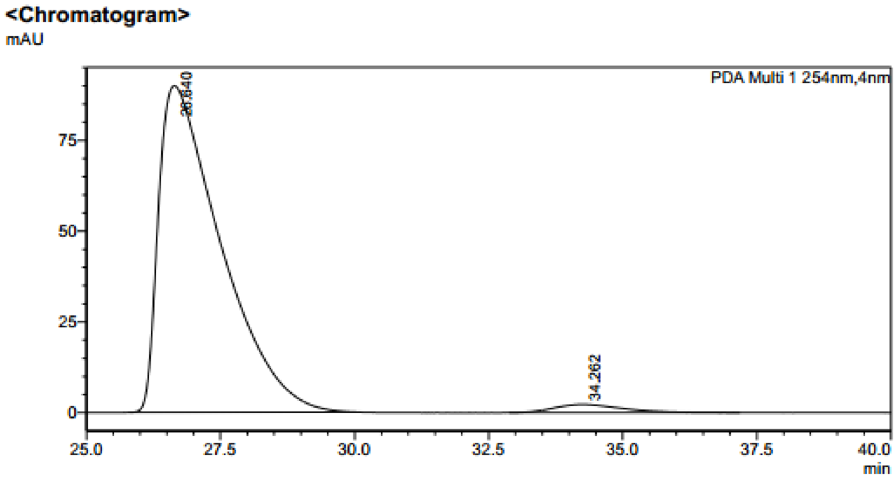

<Peak Table>

PDA Ch1 254nm

Peak#	Ret. Time	Area	Height	Area%
1	26.640	7264598	89959	97.423
2	34.262	192186	2212	2.577
Total		7456784	92171	100.000

Supplementary Fig. 188. HPLC Spectra of **3d**

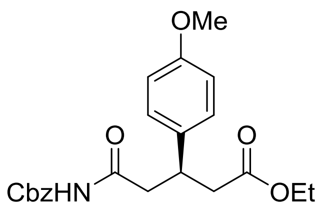

3e

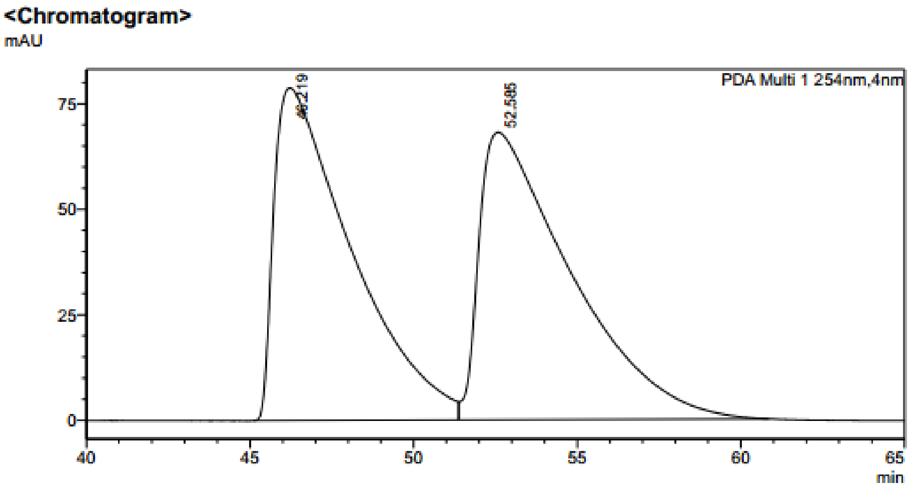

<Peak Table>

PDA Ch1 254nm

Peak#	Ret. Time	Area	Height	Area%
1	46.219	12958549	78761	49.368
2	52.585	13290529	68137	50.632
Total		26249079	146898	100.000

Supplementary Fig. 189. HPLC Spectra of racemic **3e**

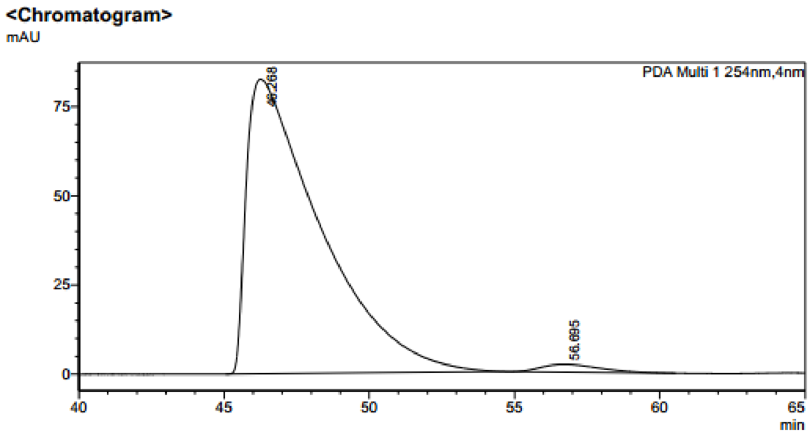

<Peak Table>

PDA Ch1 254nm

Peak#	Ret. Time	Area	Height	Area%
1	46.268	14625866	82578	97.889
2	56.695	315402	2221	2.111
Total		14941268	84799	100.000

Supplementary Fig. 190. HPLC Spectra of **3e**

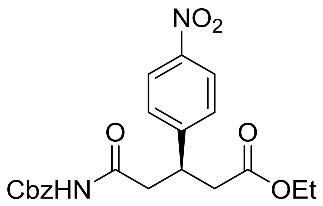

3f

<Chromatogram>

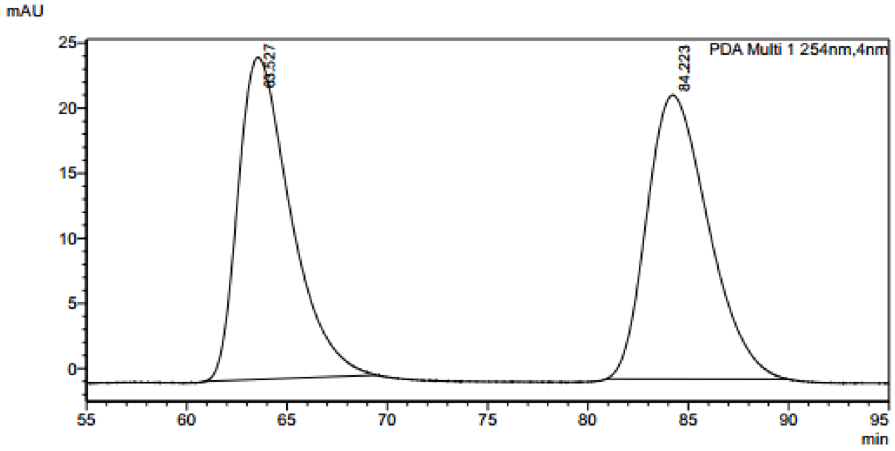

<Peak Table>

PDA Ch1 254nm

Peak#	Ret. Time	Area	Height	Area%
1	63.527	4508072	24741	49.156
2	84.223	4662960	21817	50.844
Total		9171032	46558	100.000

Supplementary Fig. 191. HPLC Spectra of racemic **3f**

<Chromatogram>

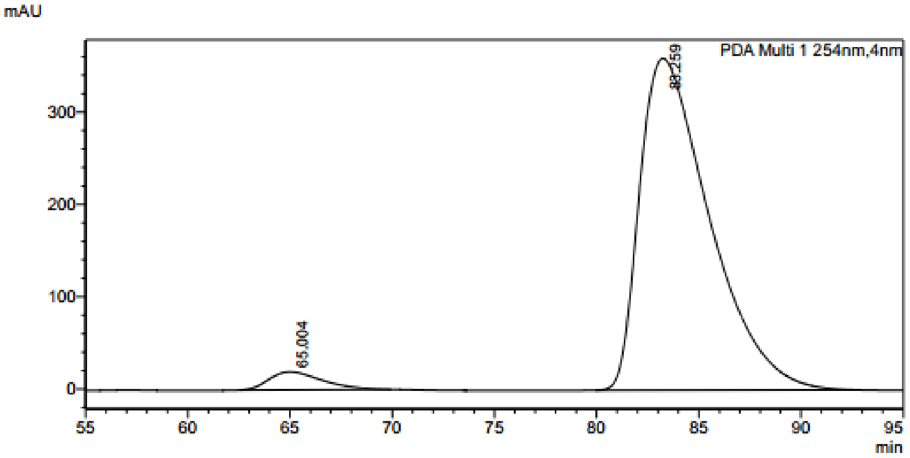

<Peak Table>

PDA Ch1 254nm

Peak#	Ret. Time	Area	Height	Area%
1	65.004	3548028	19717	3.980
2	83.259	85596501	359505	96.020
Total		89144529	379223	100.000

Supplementary Fig. 192. HPLC Spectra of **3f**

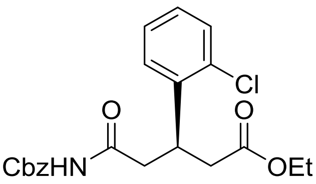

3g

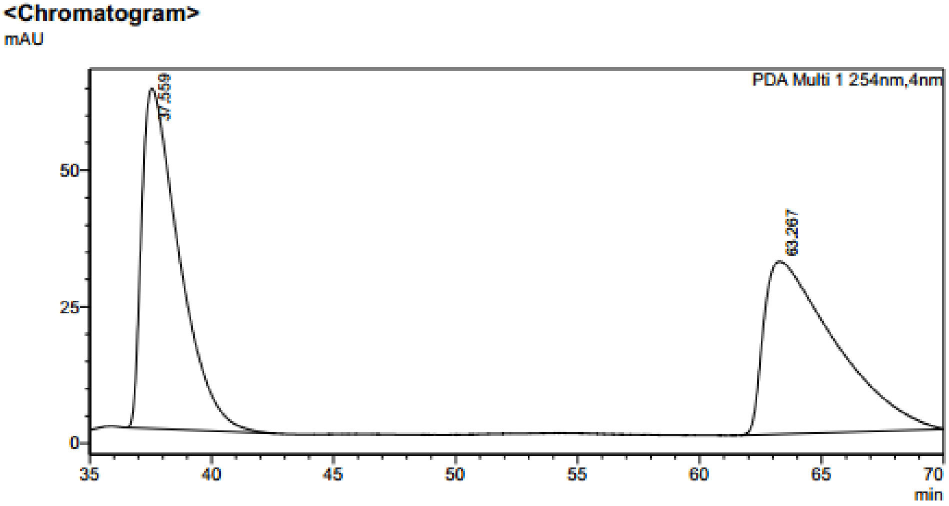

<Peak Table>

PDA Ch1 254nm

Peak#	Ret. Time	Area	Height	Area%
1	37.559	6669859	62280	50.880
2	63.267	6439031	31605	49.120
Total		13108890	93885	100.000

Supplementary Fig. 193. HPLC Spectra of racemic **3g**

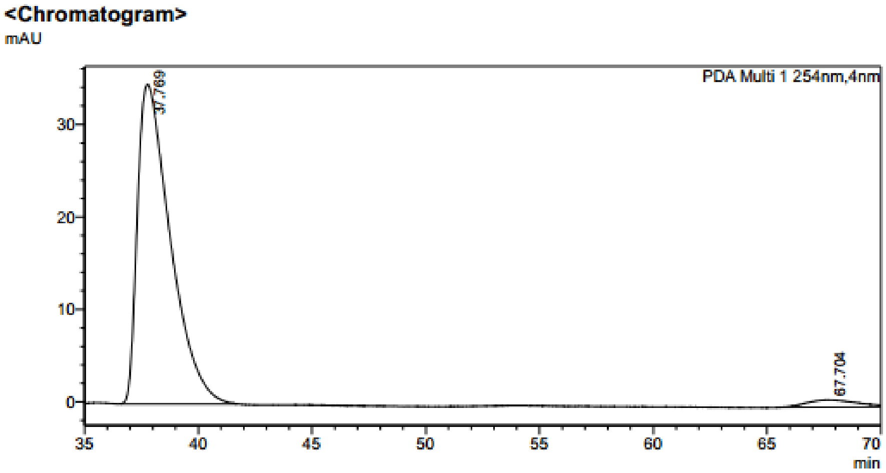

<Peak Table>

PDA Ch1 254nm

Peak#	Ret. Time	Area	Height	Area%
1	37.769	3480864	34563	96.531
2	67.704	125103	776	3.469
Total		3605967	35339	100.000

Supplementary Fig. 194. HPLC Spectra of **3g**

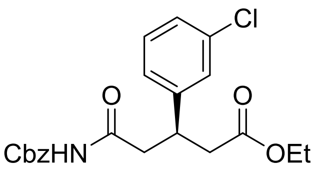

3h

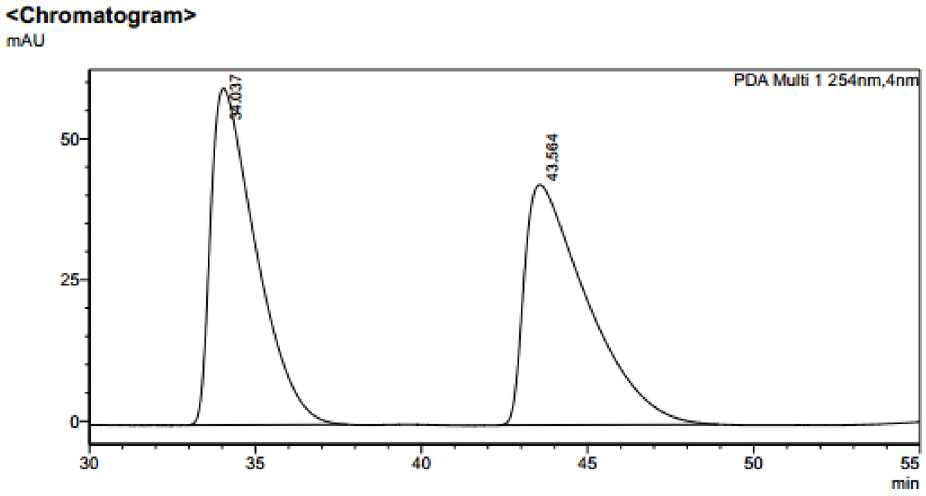

<Peak Table>

PDA Ch1 254nm

Peak#	Ret. Time	Area	Height	Area%
1	34.037	5647002	59622	50.041
2	43.564	5637746	42589	49.959
Total		11284748	102211	100.000

Supplementary Fig. 195. HPLC Spectra of racemic **3h**

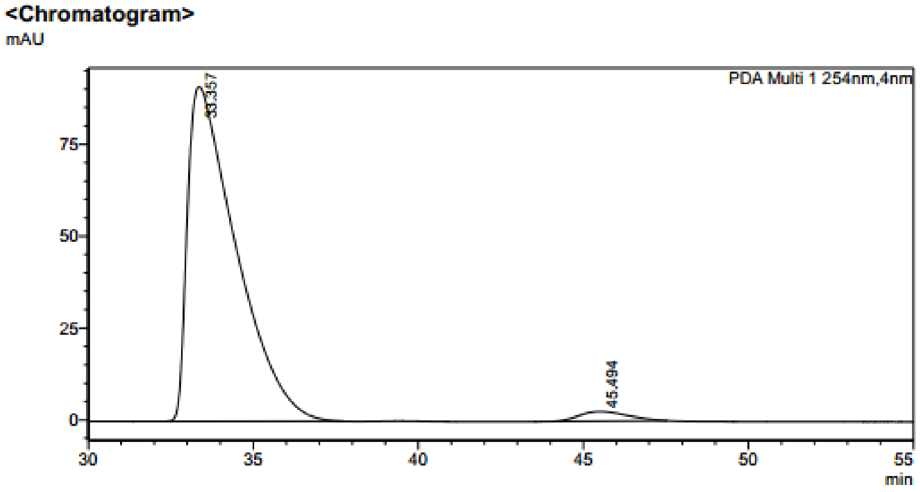

<Peak Table>

PDA Ch1 254nm

Peak#	Ret. Time	Area	Height	Area%
1	33.357	9316158	91025	97.254
2	45.494	263046	2619	2.746
Total		9579204	93645	100.000

Supplementary Fig. 196. HPLC Spectra of **3h**

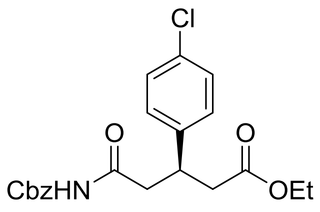

3i

<Chromatogram>

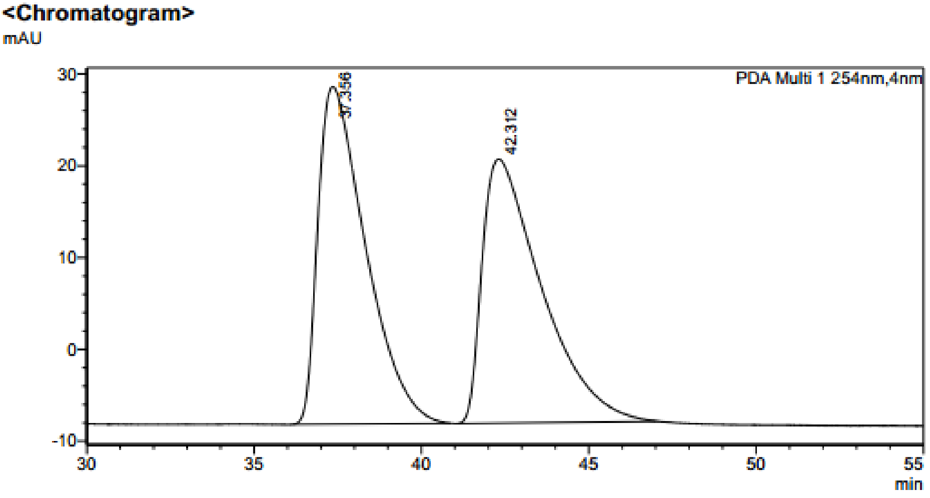

<Peak Table>

PDA Ch1 254nm

Peak#	Ret. Time	Area	Height	Area%
1	37.356	3640179	36737	50.636
2	42.312	3548785	28754	49.364
Total		7188963	65491	100.000

Supplementary Fig. 197. HPLC Spectra of racemic **3i**

<Chromatogram>

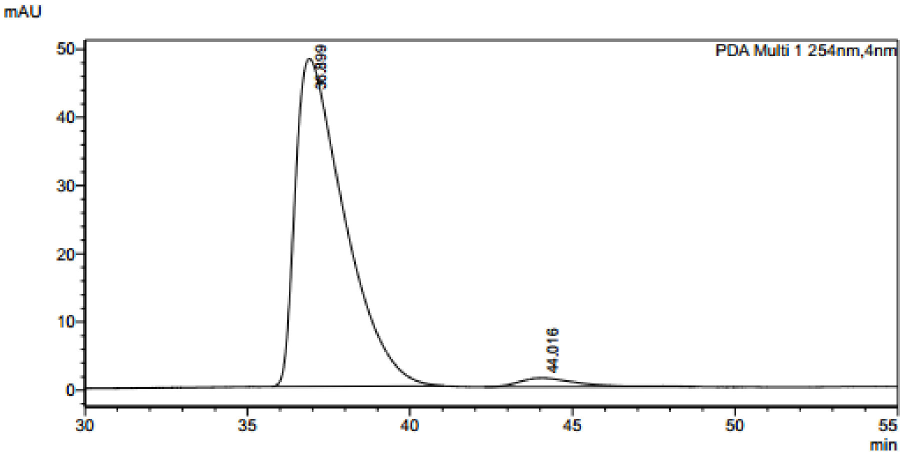

<Peak Table>

PDA Ch1 254nm

Peak#	Ret. Time	Area	Height	Area%
1	36.899	5044354	48054	96.970
2	44.016	157645	1286	3.030
Total		5201999	49340	100.000

Supplementary Fig. 198. HPLC Spectra of **3i**

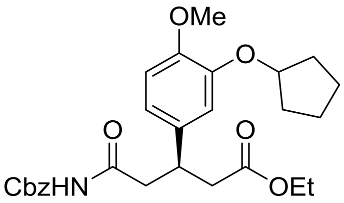

3j

<Chromatogram>

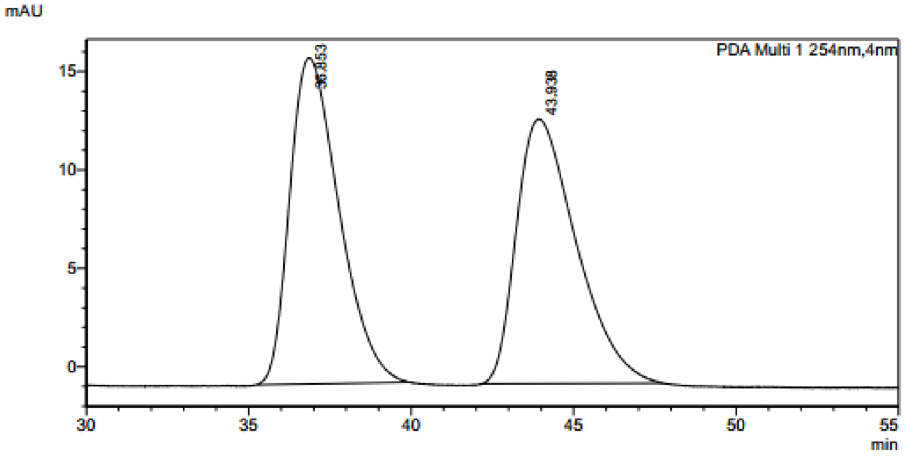

<Peak Table>

PDA Ch1 254nm

Peak#	Ret. Time	Area	Height	Area%
1	36.853	1747760	16562	50.321
2	43.938	1725450	13436	49.679
Total		3473210	29999	100.000

Supplementary Fig. 199. HPLC Spectra of racemic **3j**

<Chromatogram>

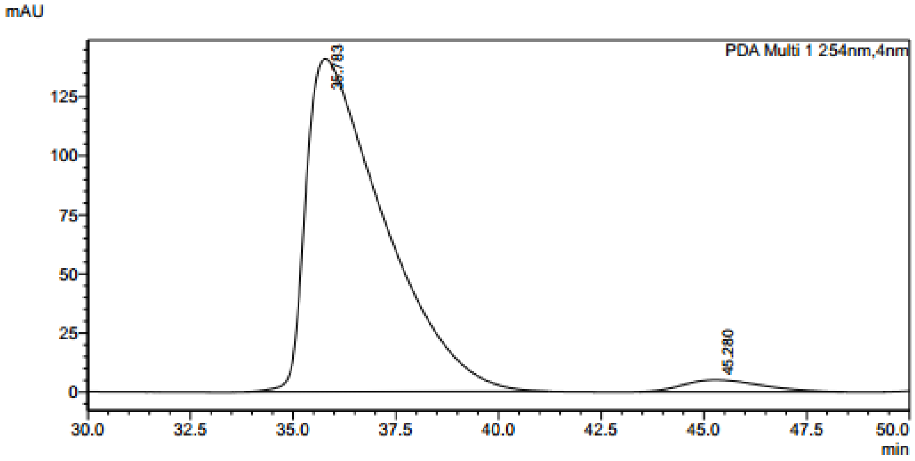

<Peak Table>

PDA Ch1 254nm

Peak#	Ret. Time	Area	Height	Area%
1	35.783	18593973	140982	96.542
2	45.280	665994	5092	3.458
Total		19259968	146074	100.000

Supplementary Fig. 200. HPLC Spectra of **3j**

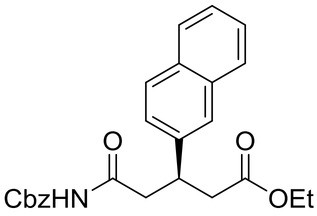

3k

<Chromatogram>

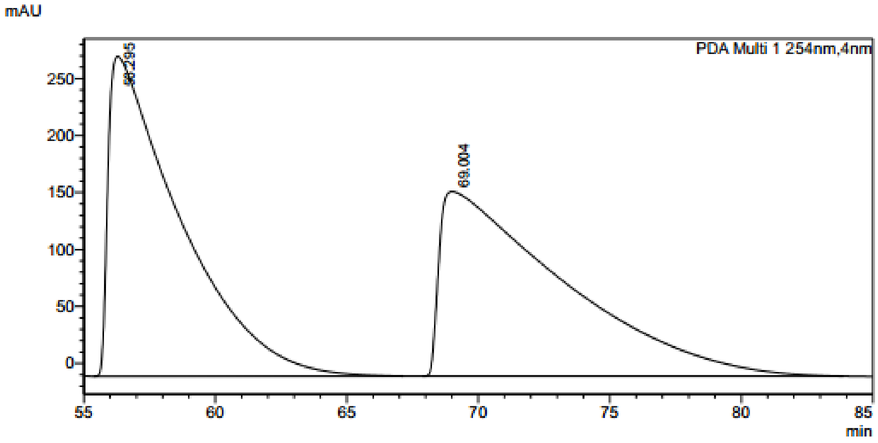

<Peak Table>

PDA Ch1 254nm

Peak#	Ret. Time	Area	Height	Area%
1	56.295	53117189	280738	50.052
2	69.004	53006034	162068	49.948
Total		106123222	442806	100.000

Supplementary Fig. 201. HPLC Spectra of racemic **3k**

<Chromatogram>

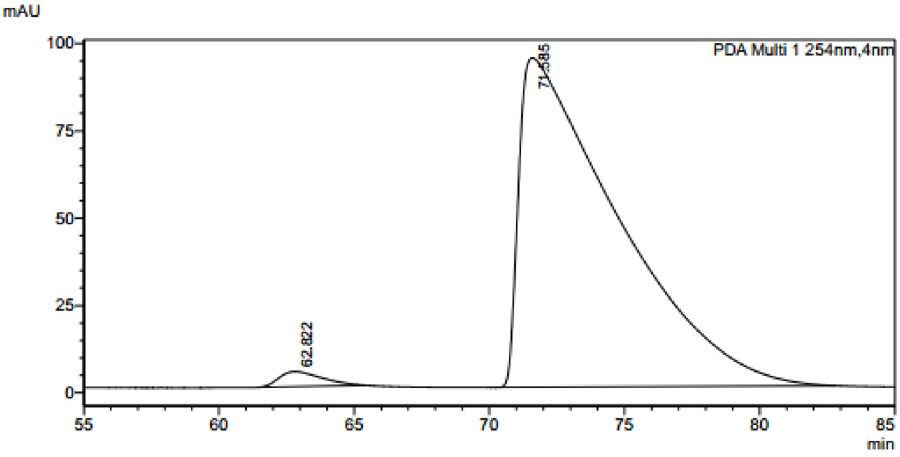

<Peak Table>

PDA Ch1 254nm

Peak#	Ret. Time	Area	Height	Area%
1	62.822	476735	4326	1.952
2	71.585	23945501	94223	98.048
Total		24422236	98549	100.000

Supplementary Fig. 202. HPLC Spectra of **3k**

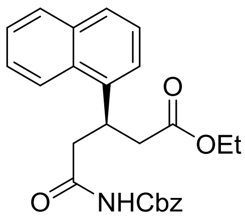

3l

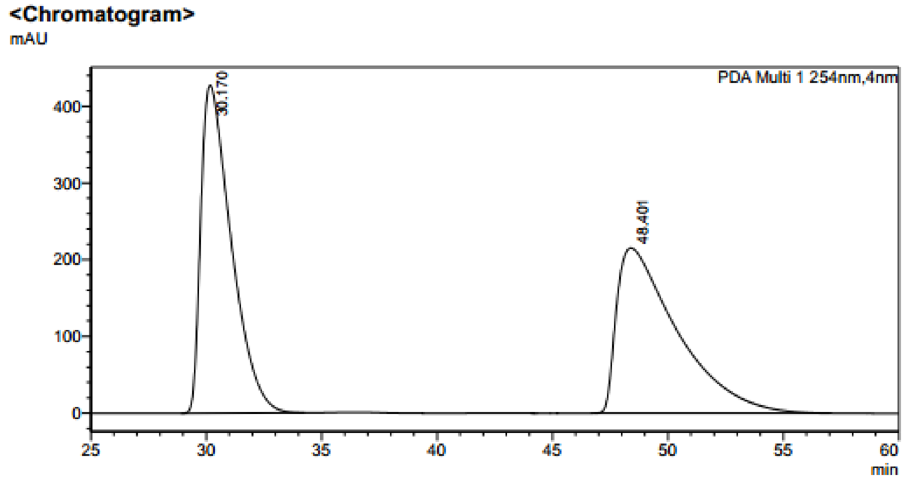

<Peak Table>

PDA Ch1 254nm

Peak#	Ret. Time	Area	Height	Area%
1	30.170	39263401	427464	49.889
2	48.401	39437472	215282	50.111
Total		78700873	642746	100.000

Supplementary Fig. 203. HPLC Spectra of racemic **3l**

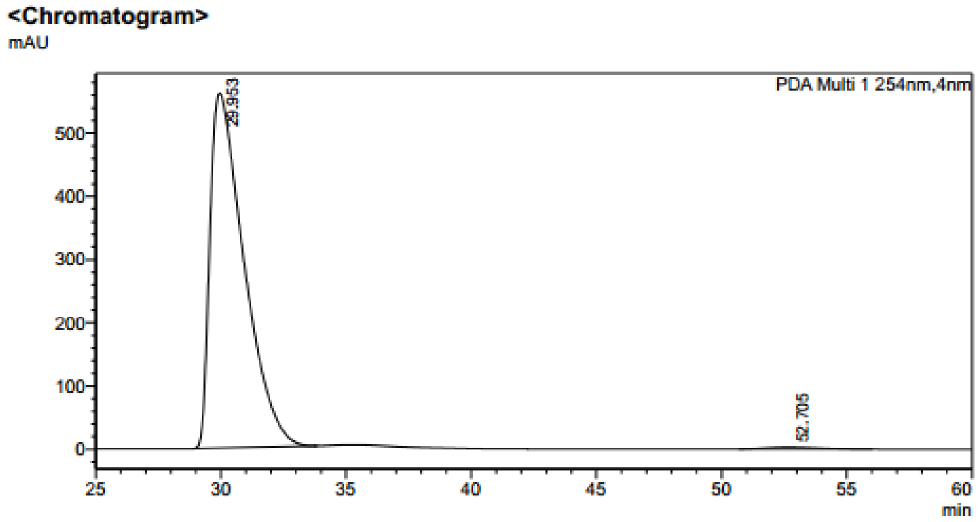

<Peak Table>

PDA Ch1 254nm

Peak#	Ret. Time	Area	Height	Area%
1	29.953	53066778	561187	99.185
2	52.705	436164	3020	0.815
Total		53502942	564208	100.000

Supplementary Fig. 204. HPLC Spectra of **3l**

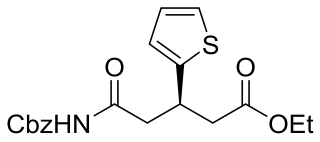

3m

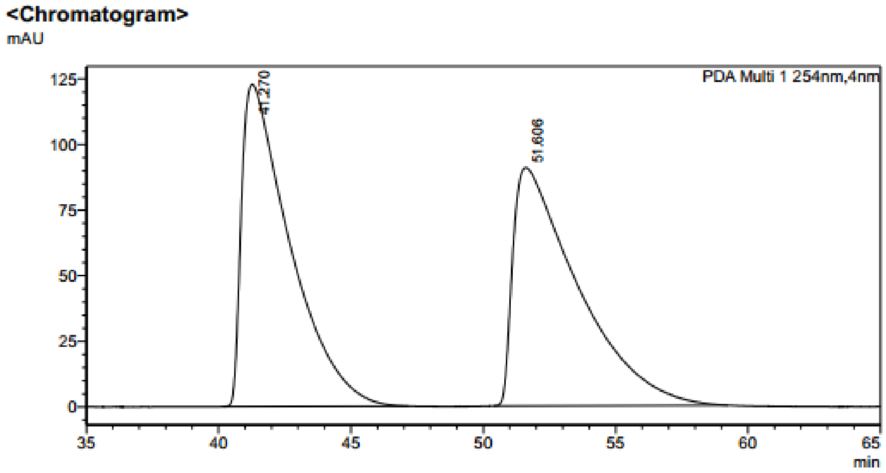

<Peak Table>

PDA Ch1 254nm

Peak#	Ret. Time	Area	Height	Area%
1	41.270	15630505	122741	50.187
2	51.606	15514137	90734	49.813
Total		31144642	213475	100.000

Supplementary Fig. 205. HPLC Spectra of racemic **3m**

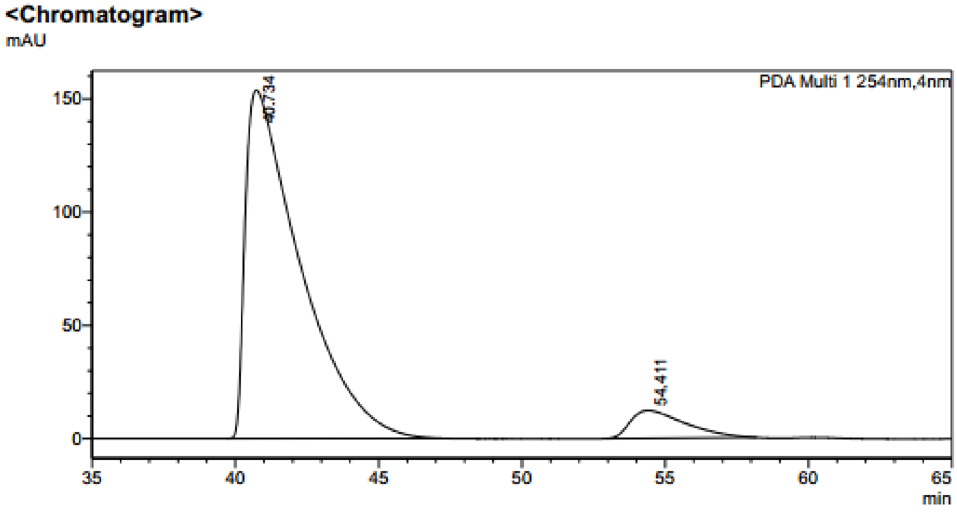

<Peak Table>

PDA Ch1 254nm

Peak#	Ret. Time	Area	Height	Area%
1	40.734	20523014	153736	92.618
2	54.411	1635795	12267	7.382
Total		22158809	166002	100.000

Supplementary Fig. 206. HPLC Spectra of **3m**

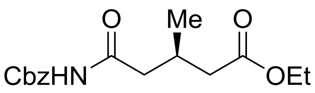

3n

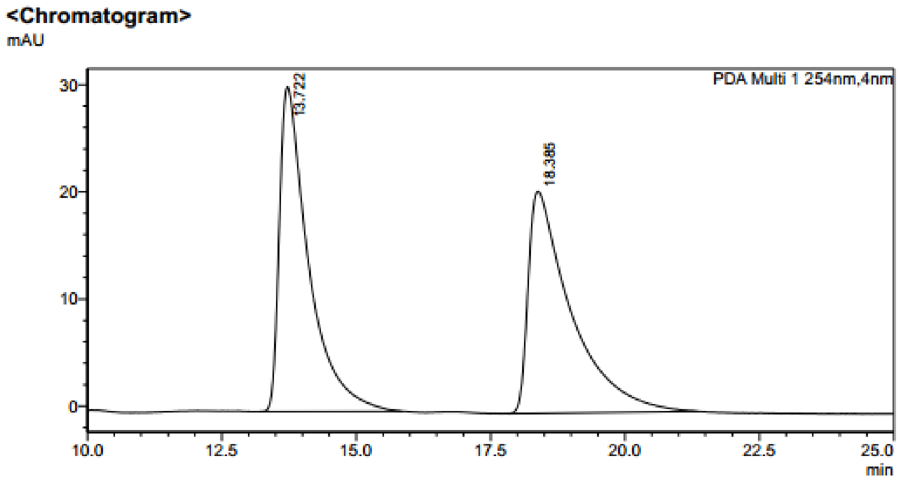

<Peak Table>

PDA Ch1 254nm

Peak#	Ret. Time	Area	Height	Area%
1	13.722	1145550	30325	49.948
2	18.385	1147915	20721	50.052
Total		2293465	51046	100.000

Supplementary Fig. 207. HPLC Spectra of racemic **3n**

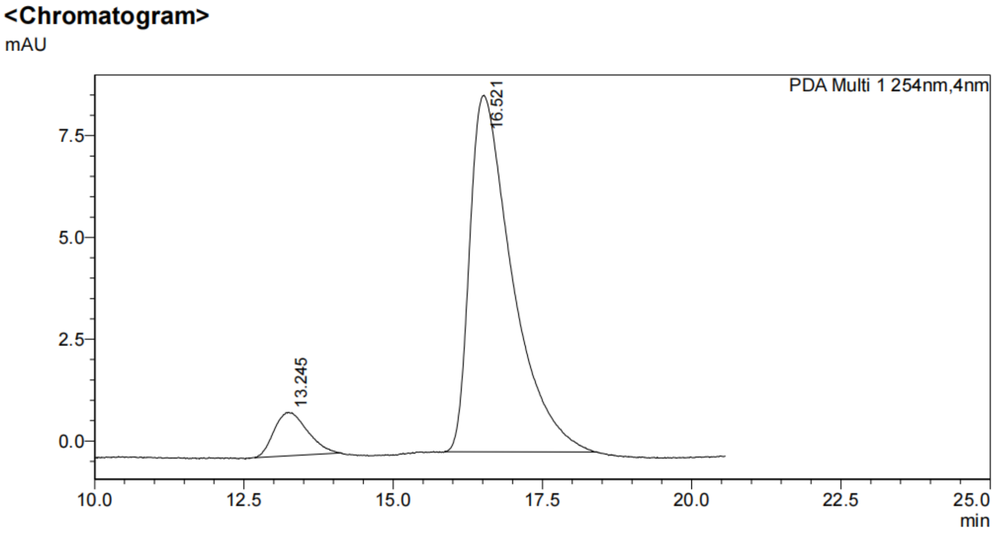

<Peak Table>

PDA Ch1 254nm

Peak#	Ret. Time	Area	Area%	Height
1	13.245	41612	8.744	1069
2	16.521	434262	91.256	8758
Total		475874	100.000	9827

Supplementary Fig. 208. HPLC Spectra of **3n**

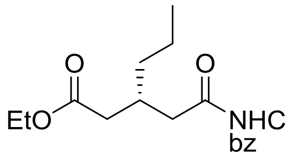

3o

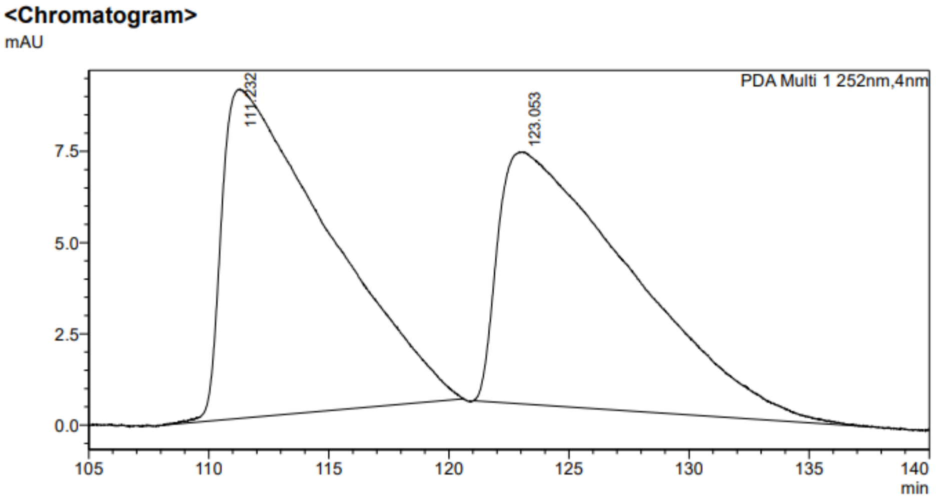

<Peak Table>

PDA Ch1 252nm

Peak#	Ret. Time	Area	Area%	Height
1	111.232	2783207	50.861	9017
2	123.053	2688943	49.139	6907
Total		5472150	100.000	15924

Supplementary Fig. 209. HPLC Spectra of racemic **3o**

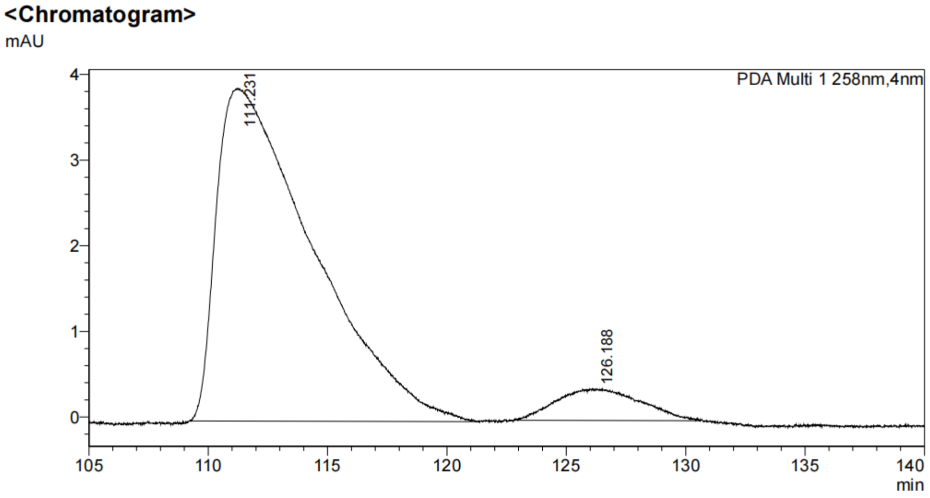

<Peak Table>

PDA Ch1 258nm

Peak#	Ret. Time	Area	Area%	Height
1	111.231	1092282	92.330	3882
2	126.188	90732	7.670	367
Total		1183014	100.000	4249

Supplementary Fig. 210. HPLC Spectra of **3o**

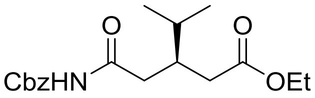

3p

<Chromatogram>

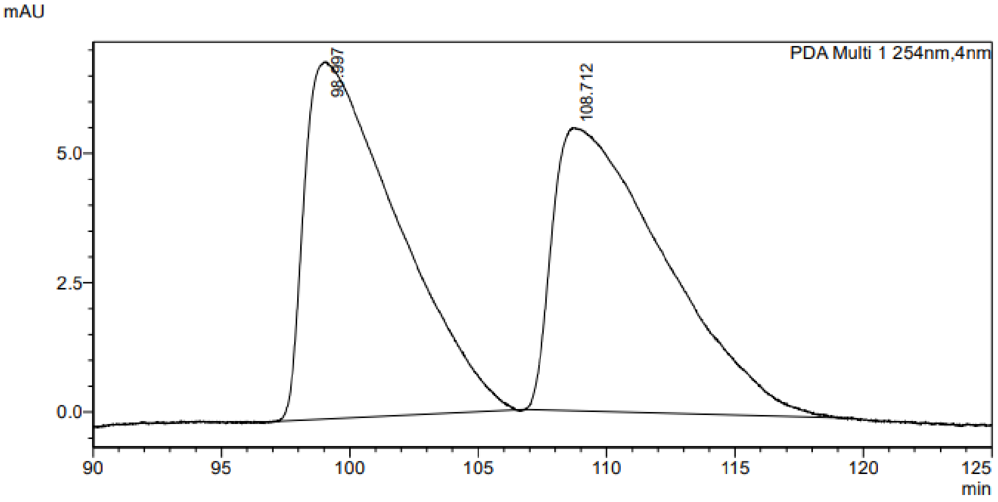

<Peak Table>

PDA Ch1 254nm

Peak#	Ret. Time	Area	Area%	Height
1	98.997	1734651	50.962	6899
2	108.712	1669168	49.038	5471
Total		3403819	100.000	12370

Supplementary Fig. 211. HPLC Spectra of racemic **3p**

<Chromatogram>

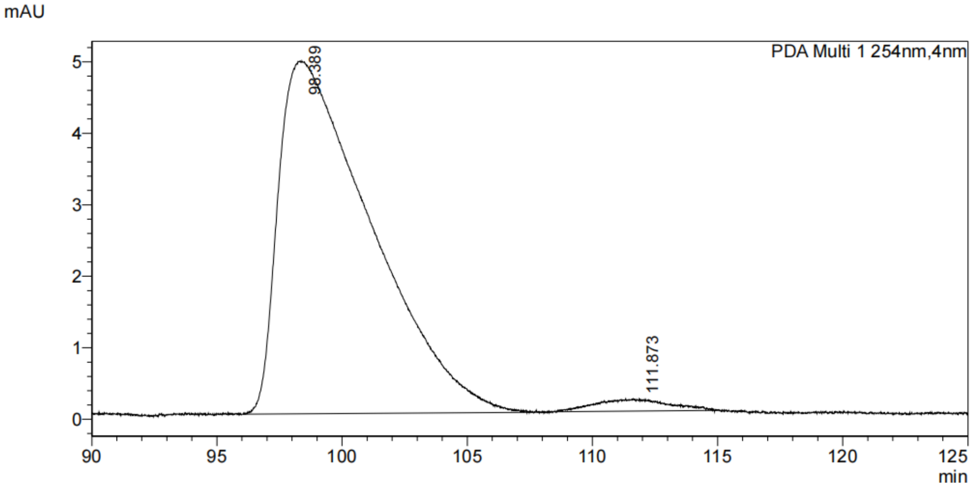

<Peak Table>

PDA Ch1 254nm

Peak#	Ret. Time	Area	Area%	Height
1	98.389	1274178	97.379	4931
2	111.873	34301	2.621	171
Total		1308479	100.000	5102

Supplementary Fig. 212. HPLC Spectra of **3p**

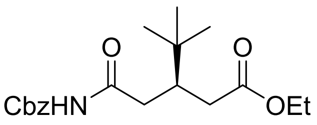

3q

<Chromatogram>

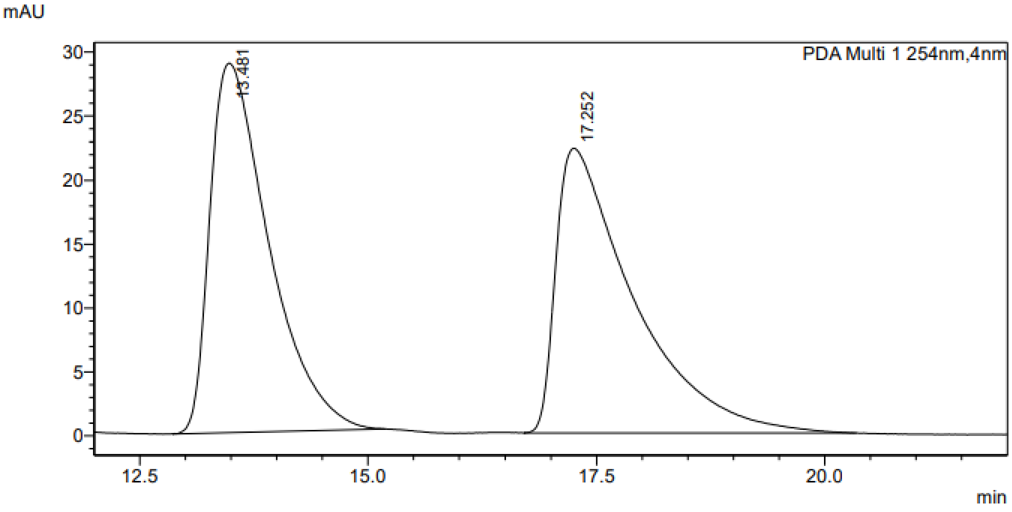

<Peak Table>

PDA Ch1 254nm

Peak#	Ret. Time	Area	Area%	Height
1	13.481	1300600	49.686	28878
2	17.252	1317034	50.314	22282
Total		2617634	100.000	51159

Supplementary Fig. 213. HPLC Spectra of racemic **3q**

<Chromatogram>

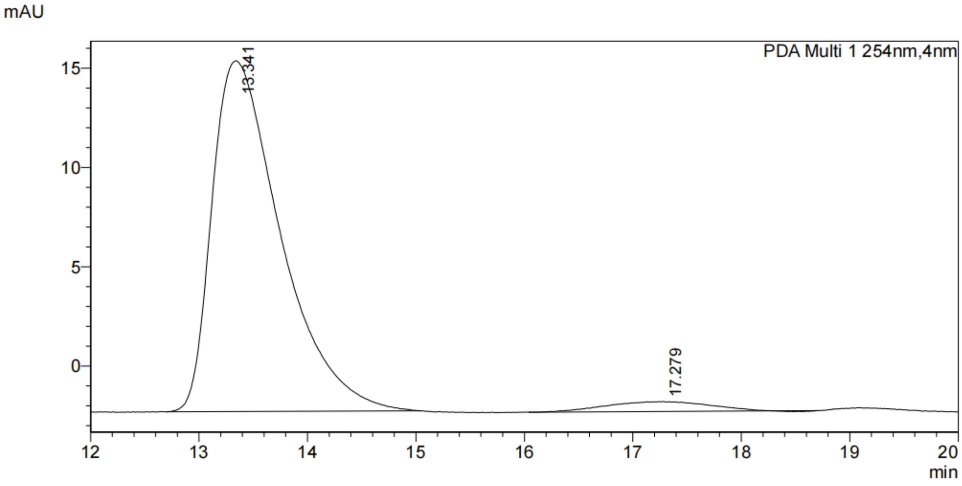

<Peak Table>

PDA Ch1 254nm

Peak#	Ret. Time	Area	Area%	Height
1	13.341	777514	95.957	17667
2	17.279	32758	4.043	500
Total		810272	100.000	18167

Supplementary Fig. 214. HPLC Spectra of **3q**

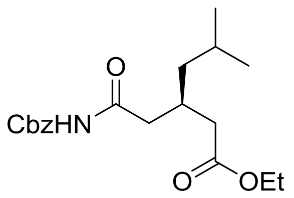

3r

<Chromatogram>

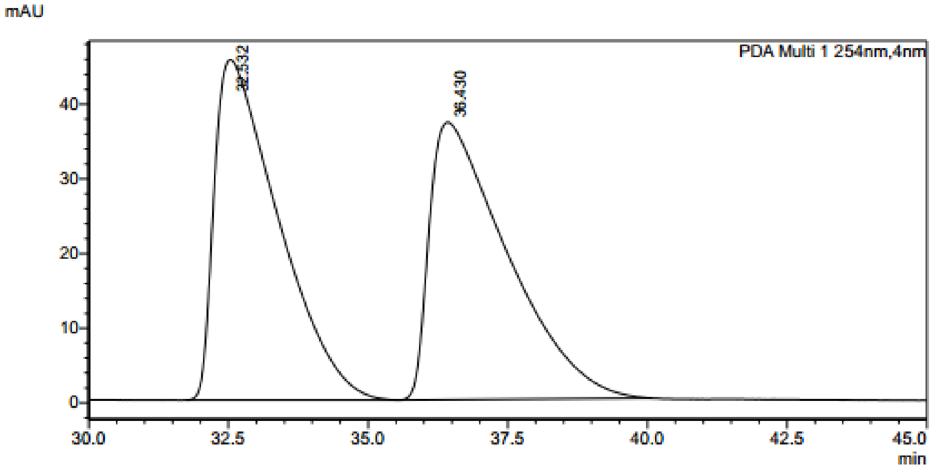

<Peak Table>

PDA Ch1 254nm

Peak#	Ret. Time	Area	Height	Area%
1	32.532	3640329	45578	50.195
2	36.430	3612005	37148	49.805
Total		7252334	82726	100.000

Supplementary Fig. 215. HPLC Spectra of racemic **3r**

<Chromatogram>

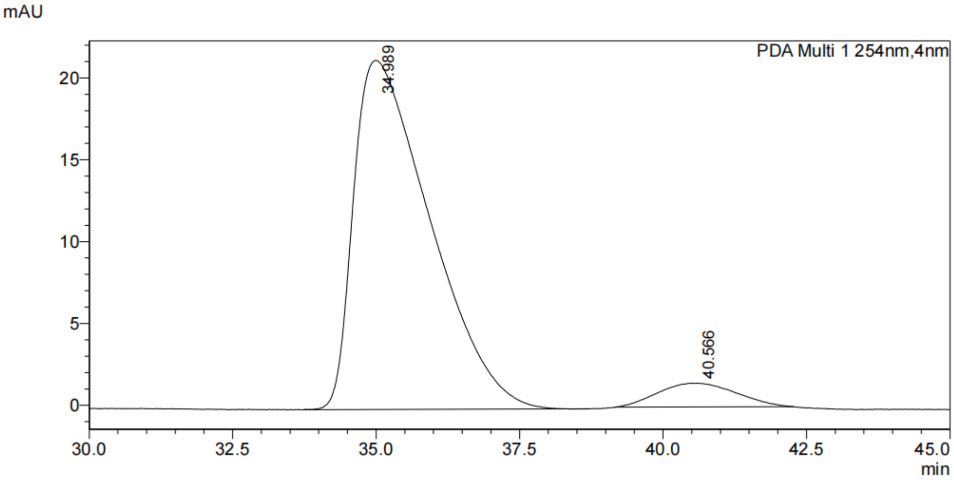

<Peak Table>

PDA Ch1 254nm

Peak#	Ret. Time	Area	Area%	Height
1	34.989	1976833	93.506	21317
2	40.566	137302	6.494	1459
Total		2114136	100.000	22776

Supplementary Fig. 216. HPLC Spectra of **3r**

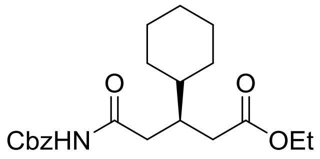

3s

<Chromatogram>

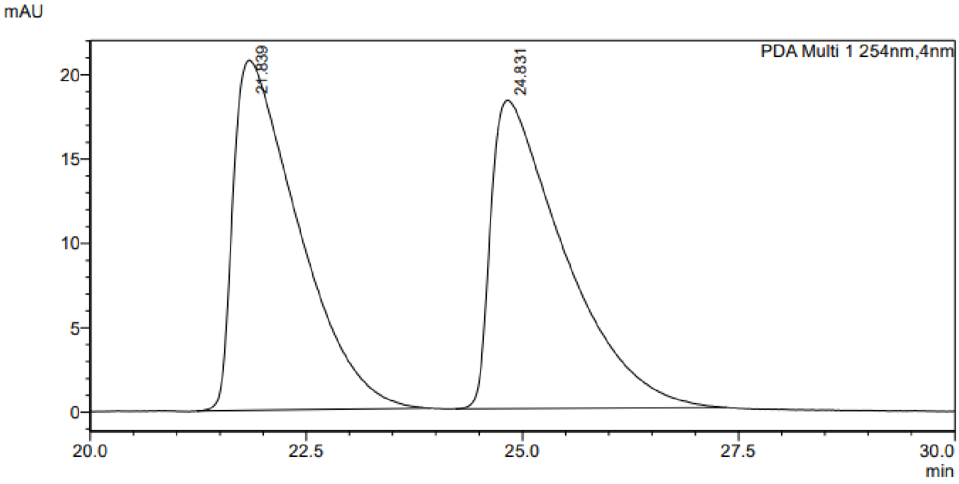

<Peak Table>

PDA Ch1 254nm

Peak#	Ret. Time	Area	Area%	Height
1	21.839	1092112	50.155	20770
2	24.831	1085372	49.845	18268
Total		2177485	100.000	39038

Supplementary Fig. 217. HPLC Spectra of racemic **3s**

<Chromatogram>

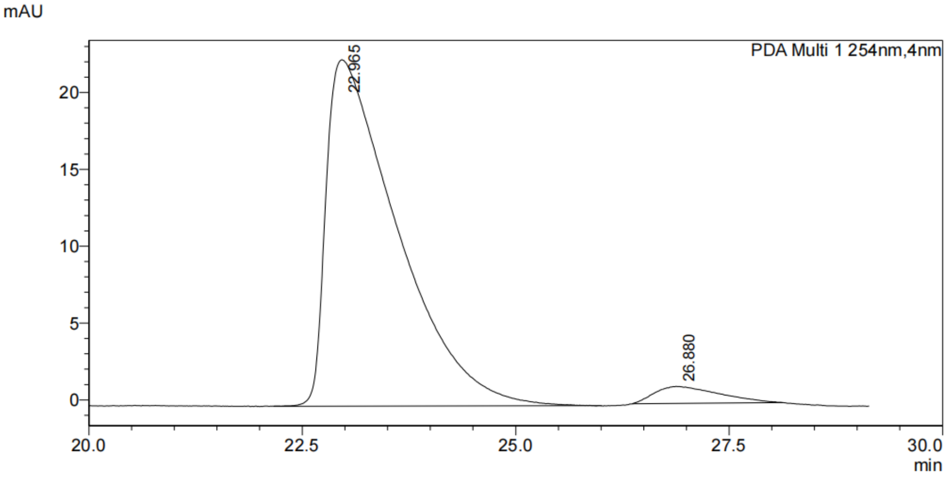

<Peak Table>

PDA Ch1 254nm

Peak#	Ret. Time	Area	Area%	Height
1	22.965	1310249	95.812	22536
2	26.880	57273	4.188	1105
Total		1367522	100.000	23641

Supplementary Fig. 218. HPLC Spectra of **3s**

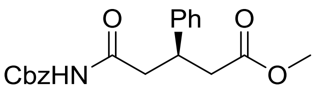

3t

<Chromatogram>

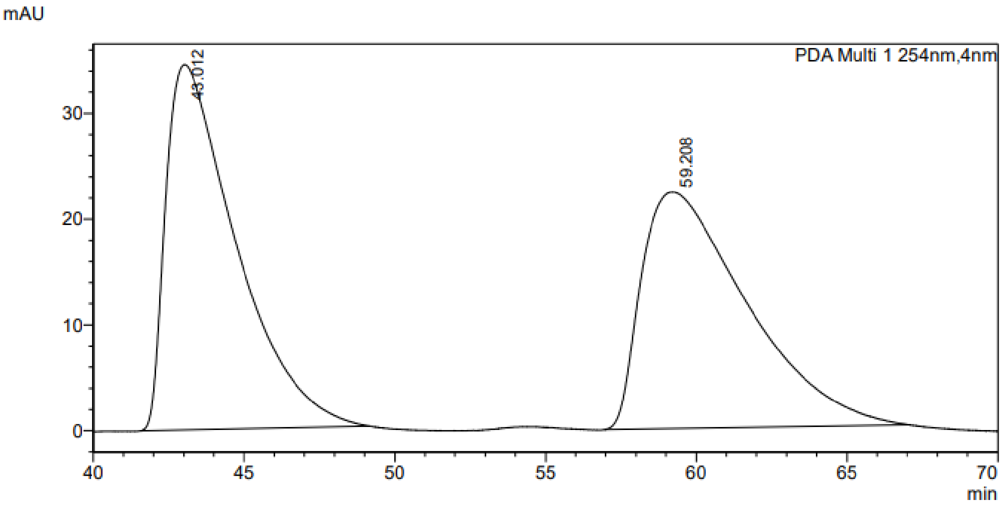

<Peak Table>

PDA Ch1 254nm

Peak#	Ret. Time	Area	Area%	Height
1	43.012	5595166	50.692	34560
2	59.208	5442379	49.308	22376
Total		11037545	100.000	56936

Supplementary Fig. 219. HPLC Spectra of racemic **3t**

<Chromatogram>

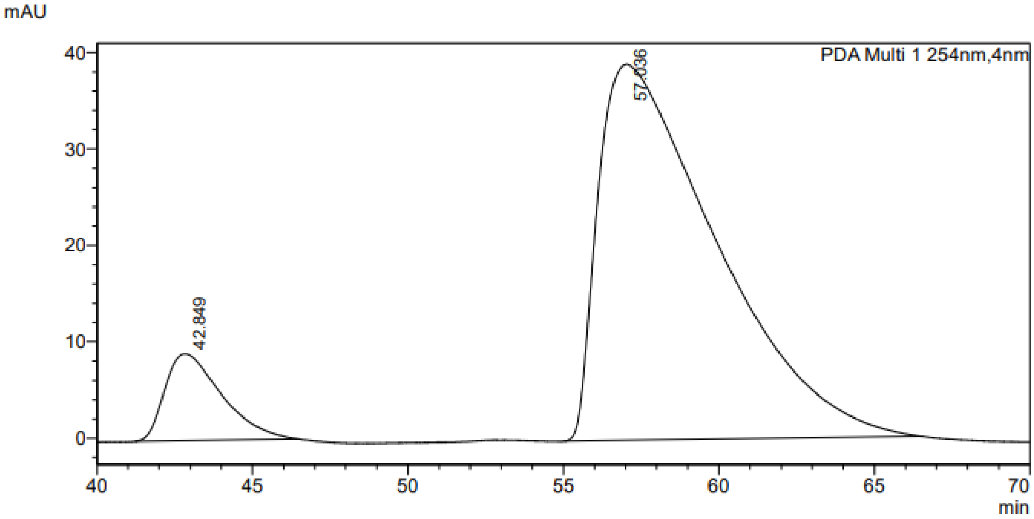

<Peak Table>

PDA Ch1 254nm

Peak#	Ret. Time	Area	Area%	Height
1	42.849	1146858	9.997	9031
2	57.036	10325669	90.003	38989
Total		11472527	100.000	48020

Supplementary Fig. 220. HPLC Spectra of **3t**

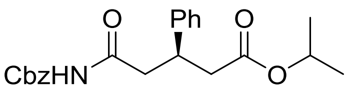

3u

<Chromatogram>

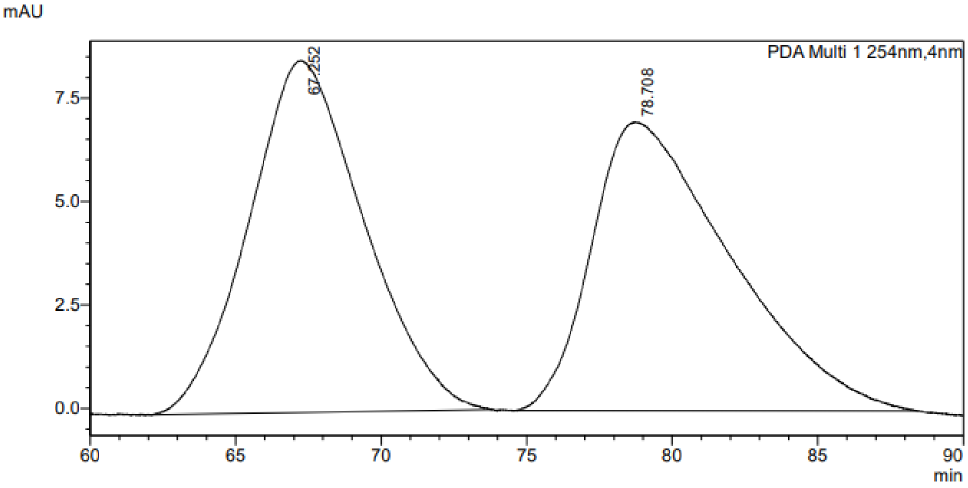

<Peak Table>

PDA Ch1 254nm

Peak#	Ret. Time	Area	Area%	Height
1	67.252	2335960	50.163	8498
2	78.708	2320795	49.837	6981
Total		4656754	100.000	15479

Supplementary Fig. 221. HPLC Spectra of racemic **3u**

<Chromatogram>

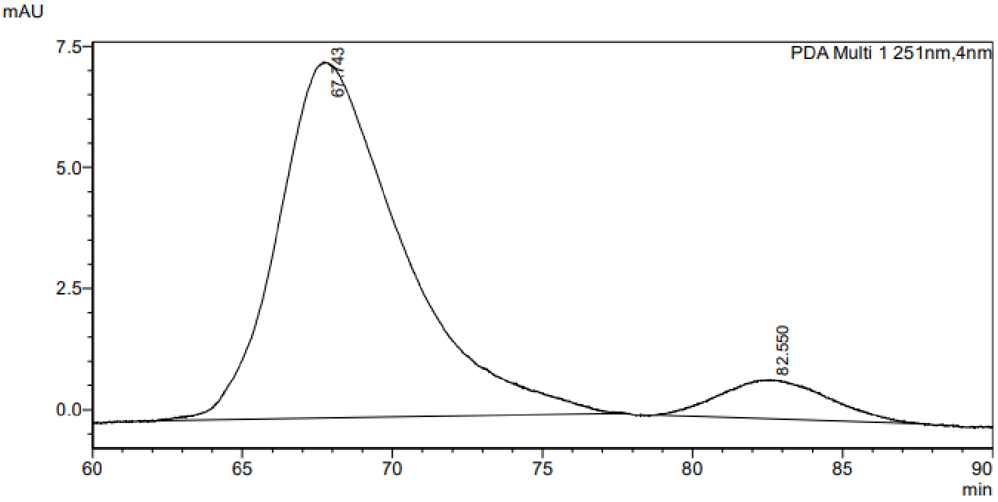

<Peak Table>

PDA Ch1 251nm

Peak#	Ret. Time	Area	Area%	Height
1	67.743	2106533	91.149	7348
2	82.550	204546	8.851	808
Total		2311079	100.000	8157

Supplementary Fig. 222. HPLC Spectra of **3u**

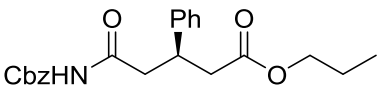

3v

<Chromatogram>

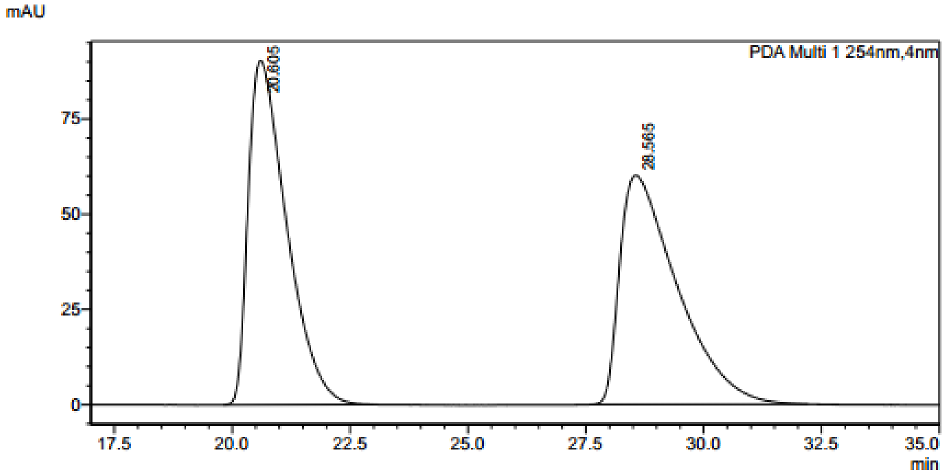

<Peak Table>

PDA Ch1 254nm

Peak#	Ret. Time	Area	Height	Area%
1	20.605	5122469	90351	50.002
2	28.565	5122017	60146	49.998
Total		10244486	150497	100.000

Supplementary Fig. 223. HPLC Spectra of racemic **3v**

<Chromatogram>

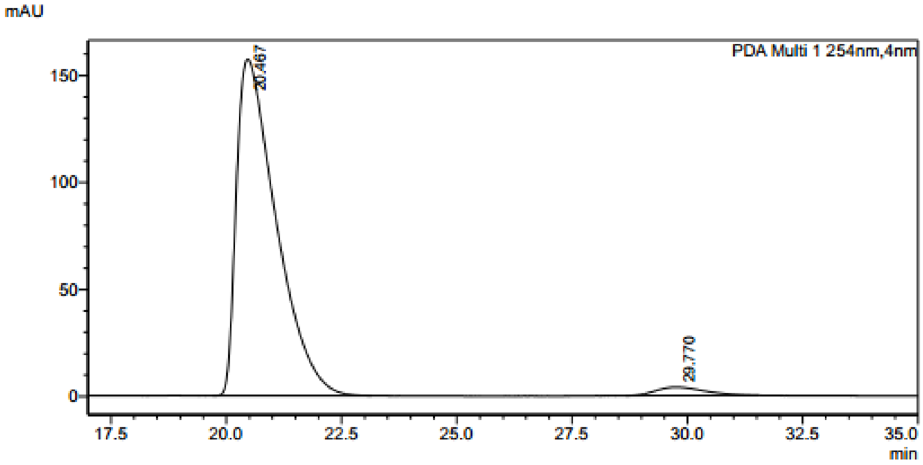

<Peak Table>

PDA Ch1 254nm

Peak#	Ret. Time	Area	Height	Area%
1	20.467	9424160	157300	96.992
2	29.770	292319	3938	3.008
Total		9716479	161239	100.000

Supplementary Fig. 224. HPLC Spectra of **3v**

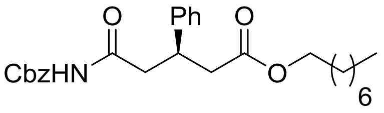

3w

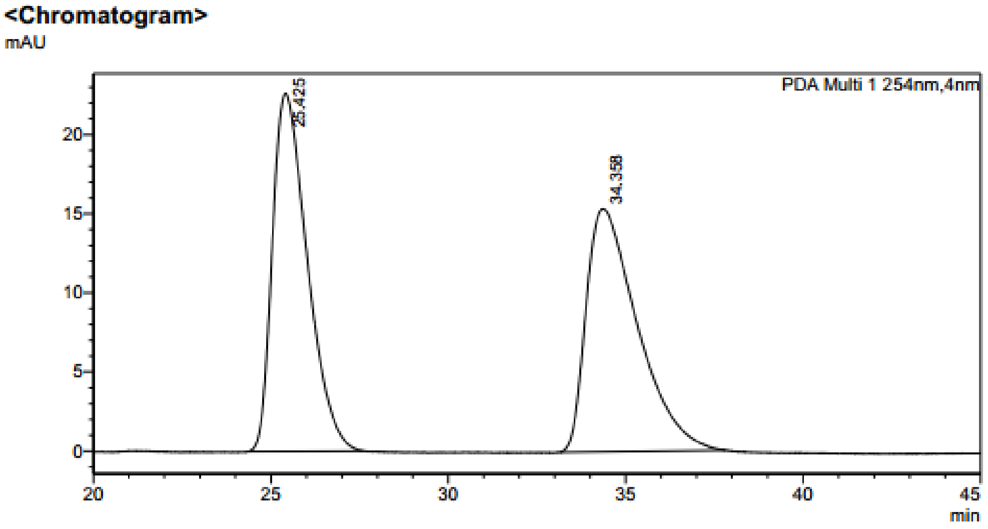

<Peak Table>

PDA Ch1 254nm

Peak#	Ret. Time	Area	Height	Area%
1	25.425	1548410	22613	50.059
2	34.358	1544767	15350	49.941
Total		3093177	37964	100.000

Supplementary Fig. 225. HPLC Spectra of racemic **3w**

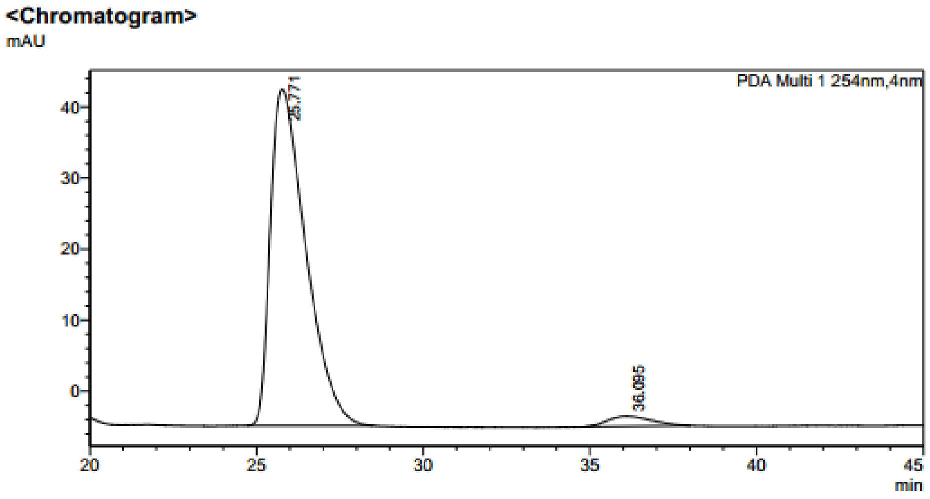

<Peak Table>

PDA Ch1 254nm

Peak#	Ret. Time	Area	Height	Area%
1	25.771	3487533	47318	96.504
2	36.095	126335	1389	3.496
Total		3613869	48708	100.000

Supplementary Fig. 226. HPLC Spectra of **3w**

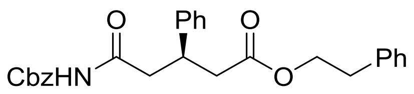

3x

<Chromatogram>

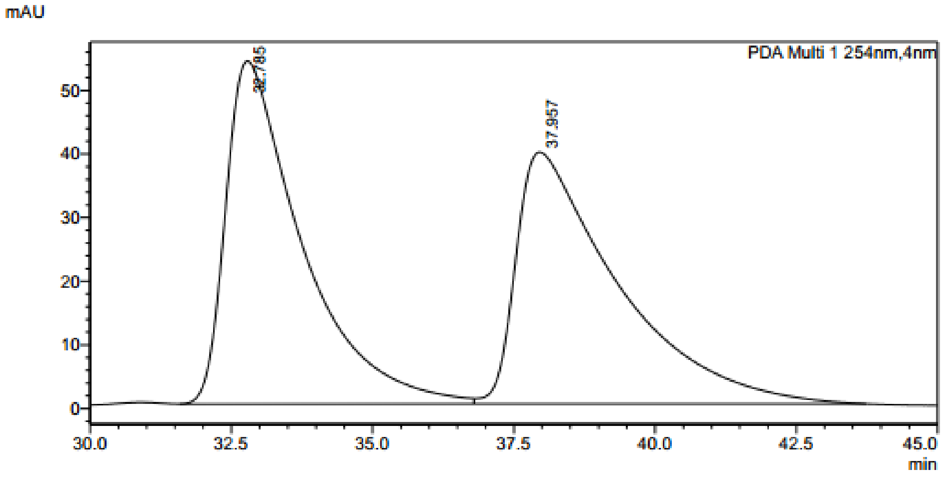

<Peak Table>

PDA Ch1 254nm

Peak#	Ret. Time	Area	Height	Area%
1	32.785	5056437	53909	50.523
2	37.957	4951657	39546	49.477
Total		10008094	93455	100.000

Supplementary Fig. 227. HPLC Spectra of racemic **3x**

<Chromatogram>

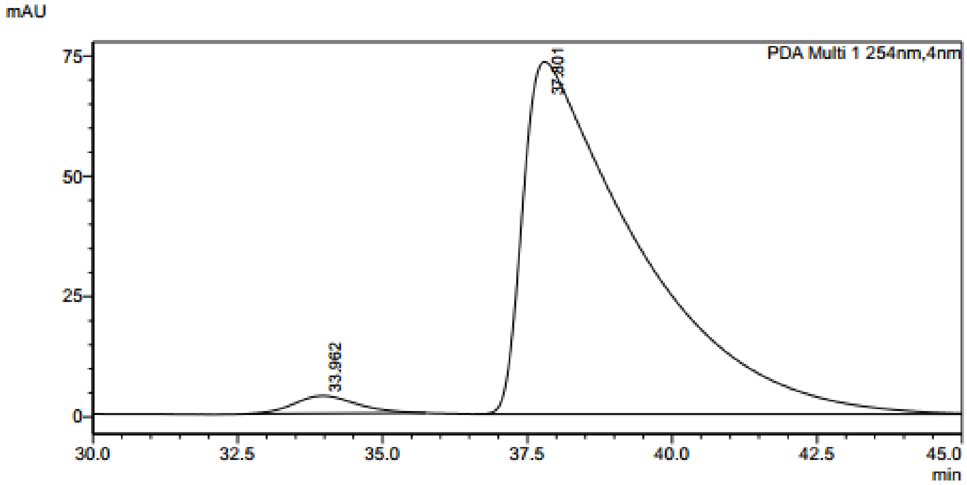

<Peak Table>

PDA Ch1 254nm

Peak#	Ret. Time	Area	Height	Area%
1	33.962	275646	3662	2.657
2	37.801	10099708	73182	97.343
Total		10375355	76844	100.000

Supplementary Fig. 228. HPLC Spectra of **3x**

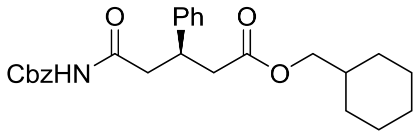

3y

<Chromatogram>

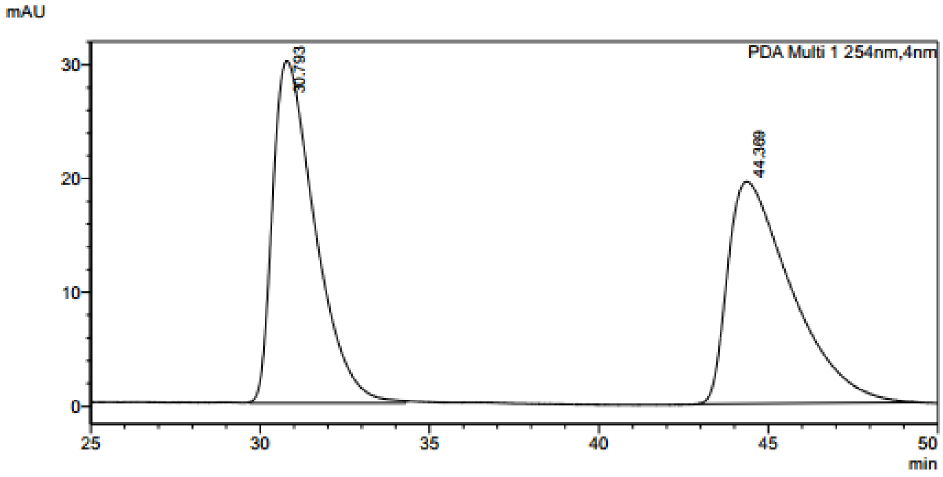

<Peak Table>

PDA Ch1 254nm

Peak#	Ret. Time	Area	Height	Area%
1	30.793	2617355	30057	50.522
2	44.369	2563308	19491	49.478
Total		5180663	49548	100.000

Supplementary Fig. 229. HPLC Spectra of racemic **3y**

<Chromatogram>

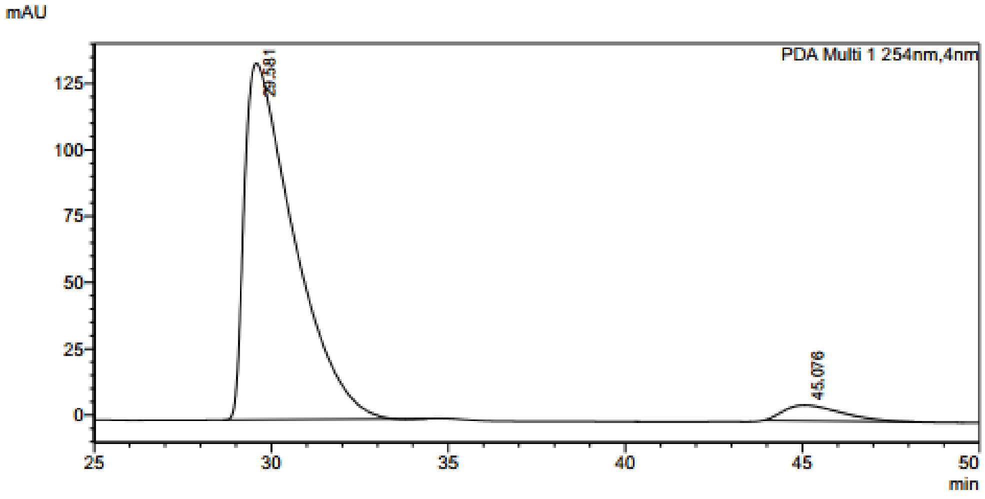

<Peak Table>

PDA Ch1 254nm

Peak#	Ret. Time	Area	Height	Area%
1	29.581	13060855	134291	95.082
2	45.076	675528	5934	4.918
Total		13736383	140225	100.000

Supplementary Fig. 230. HPLC Spectra of **3y**

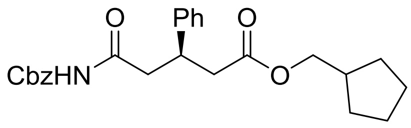

3z

<Chromatogram>

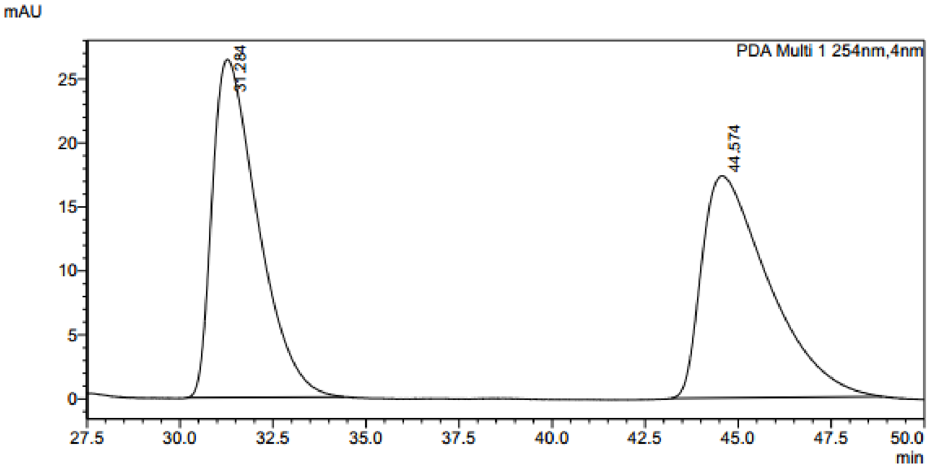

<Peak Table>

PDA Ch1 254nm

Peak#	Ret. Time	Area	Height	Area%
1	31.284	2254610	26437	50.738
2	44.574	2188981	17356	49.262
Total		4443590	43794	100.000

Supplementary Fig. 231. HPLC Spectra of racemic **3z**

<Chromatogram>

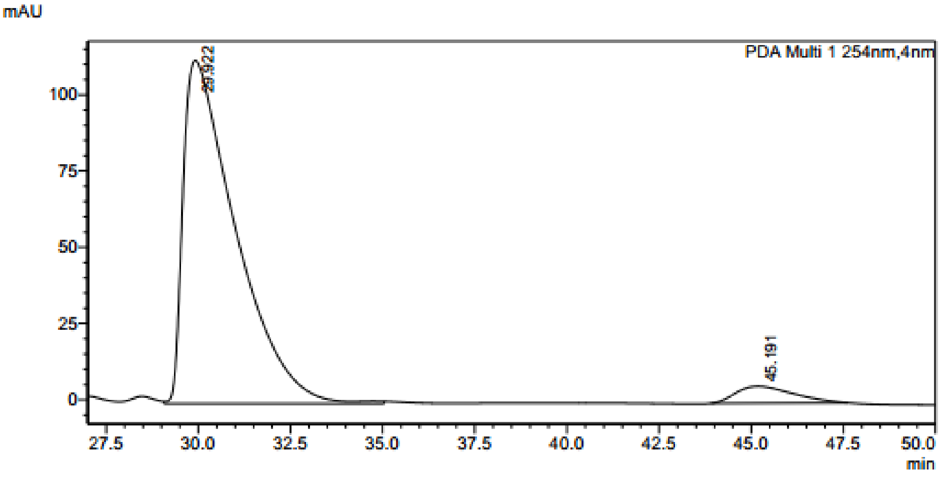

<Peak Table>

PDA Ch1 254nm

Peak#	Ret. Time	Area	Height	Area%
1	29.922	11145884	112512	94.913
2	45.191	597368	5520	5.087
Total		11743252	118033	100.000

Supplementary Fig. 232. HPLC Spectra of **3z**

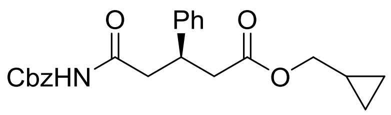

3aa

<Chromatogram>

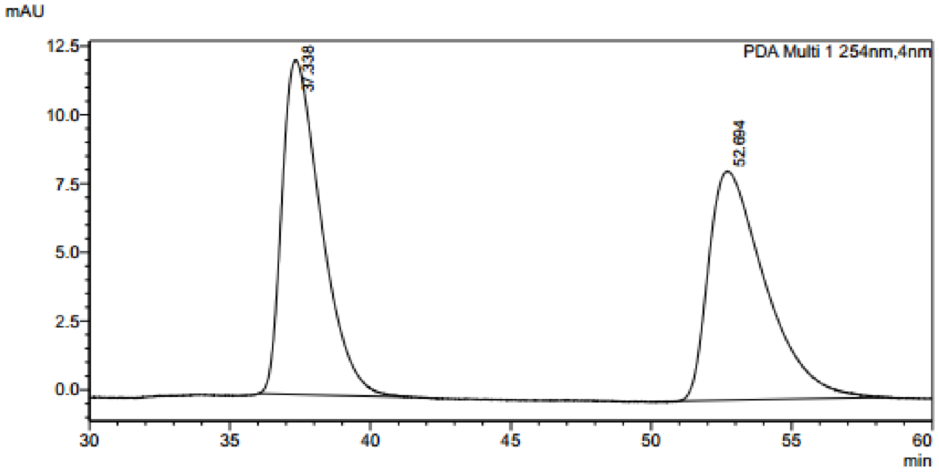

<Peak Table>

PDA Ch1 254nm

Peak#	Ret. Time	Area	Height	Area%
1	37.338	1179852	12169	50.244
2	52.694	1168389	8318	49.756
Total		2348241	20487	100.000

Supplementary Fig. 233. HPLC Spectra of racemic **3aa**

<Chromatogram>

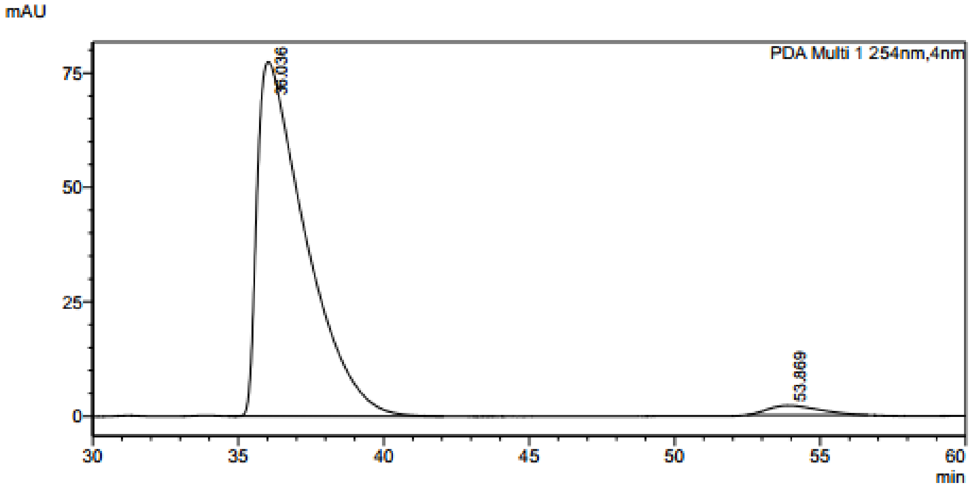

<Peak Table>

PDA Ch1 254nm

Peak#	Ret. Time	Area	Height	Area%
1	36.036	8957488	77349	97.213
2	53.869	256801	2099	2.787
Total		9214289	79448	100.000

Supplementary Fig. 234. HPLC Spectra of **3aa**

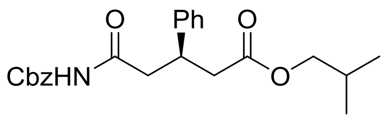

3ab

<Chromatogram>

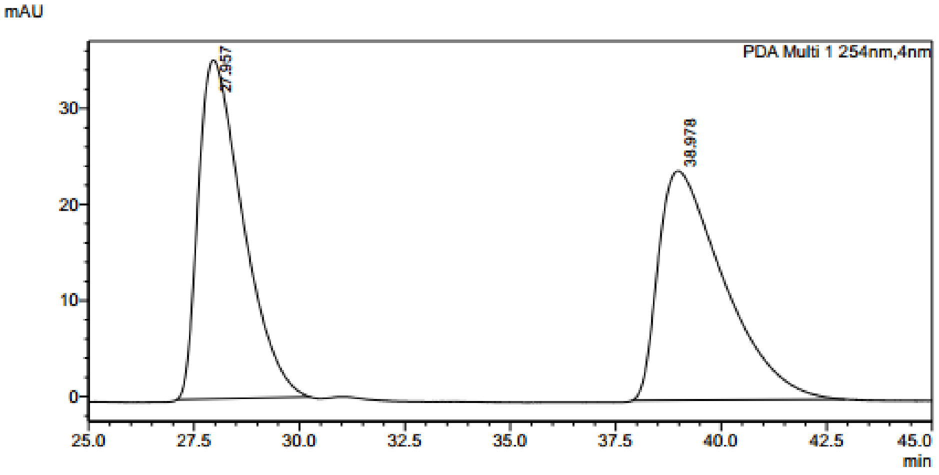

<Peak Table>

PDA Ch1 254nm

Peak#	Ret. Time	Area	Height	Area%
1	27.957	2596769	35257	50.064
2	38.978	2590101	23862	49.936
Total		5186870	59119	100.000

Supplementary Fig. 235. HPLC Spectra of racemic **3ab**

<Chromatogram>

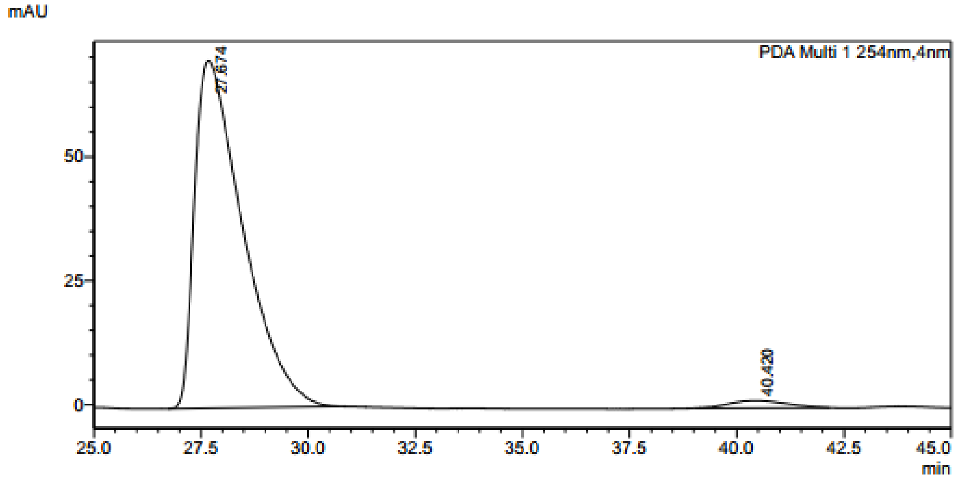

<Peak Table>

PDA Ch1 254nm

Peak#	Ret. Time	Area	Height	Area%
1	27.674	5487539	69993	97.505
2	40.420	140440	1540	2.495
Total		5627980	71533	100.000

Supplementary Fig. 236. HPLC Spectra of **3ab**

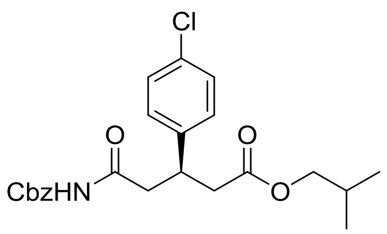

3ac

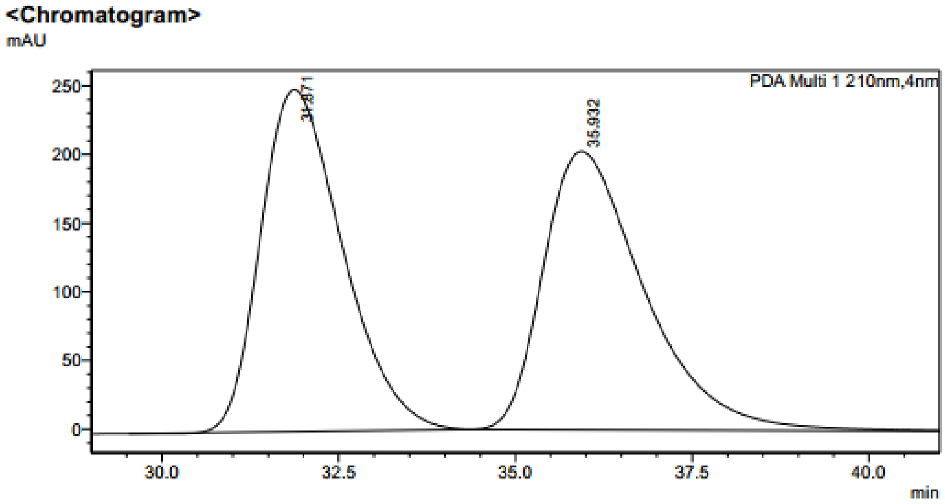

<Peak Table>

PDA Ch1 210nm

Peak#	Ret. Time	Area	Height	Area%
1	31.871	20079070	249013	49.861
2	35.932	20191422	202767	50.139
Total		40270492	451780	100.000

Supplementary Fig. 237. HPLC Spectra of racemic **3ac**

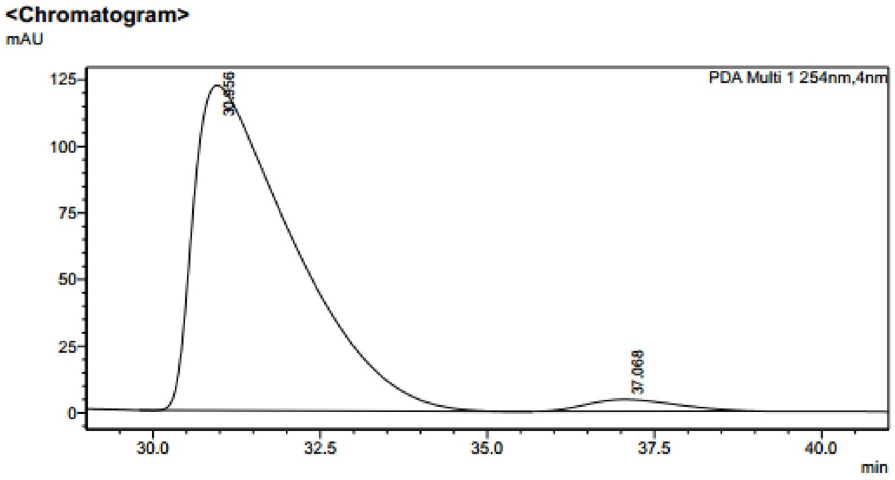

<Peak Table>

PDA Ch1 254nm

Peak#	Ret. Time	Area	Height	Area%
1	30.956	12602826	121964	96.900
2	37.068	403119	4369	3.100
Total		13005945	126333	100.000

Supplementary Fig. 238. HPLC Spectra of **3ac**

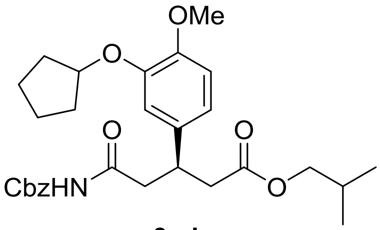

3ad

<Chromatogram>

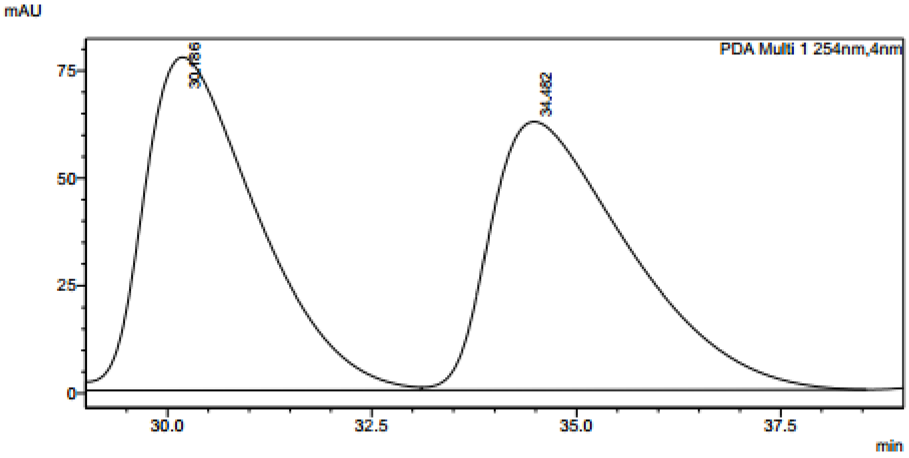

<Peak Table>

PDA Ch1 254nm

Peak#	Ret. Time	Area	Height	Area%
1	30.186	7420488	77441	50.237
2	34.482	7350556	62331	49.763
Total		14771044	139771	100.000

Supplementary Fig. 239. HPLC Spectra of racemic **3ad**

<Chromatogram>

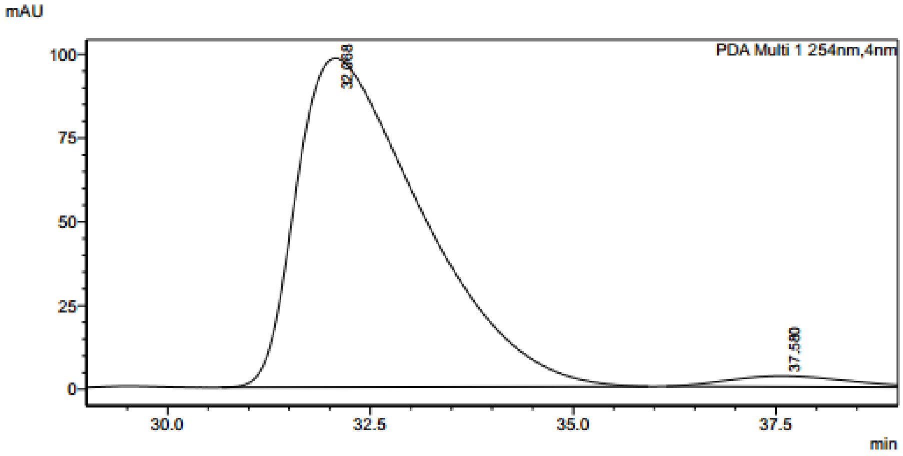

<Peak Table>

PDA Ch1 254nm

Peak#	Ret. Time	Area	Height	Area%
1	32.068	10593212	98226	96.984
2	37.580	329447	3201	3.016
Total		10922658	101427	100.000

Supplementary Fig. 240. HPLC Spectra of **3ad**

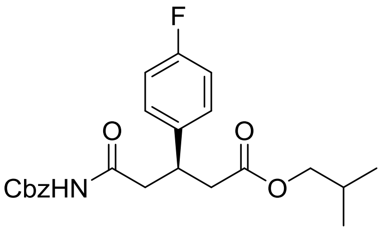

3ae

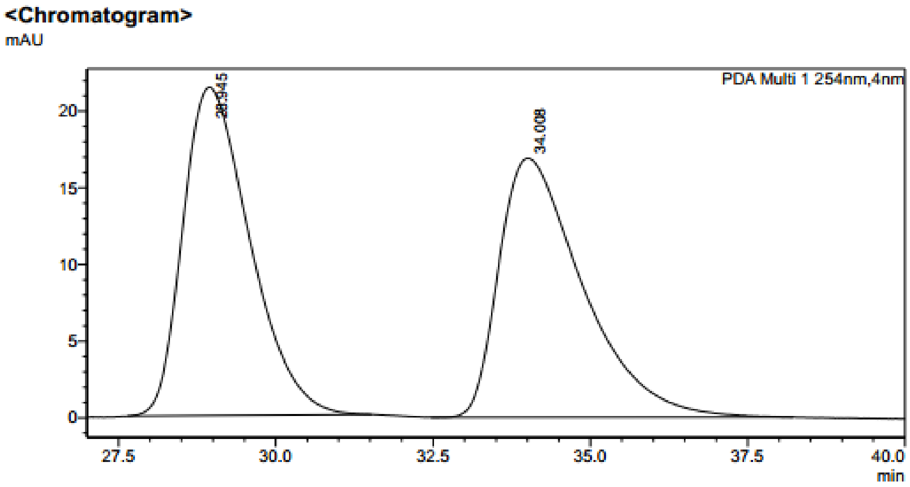

<Peak Table>

PDA Ch1 254nm

Peak#	Ret. Time	Area	Height	Area%
1	28.945	1590079	21426	50.346
2	34.008	1568237	16906	49.654
Total		3158316	38332	100.000

Supplementary Fig. 241. HPLC Spectra of racemic **3ae**

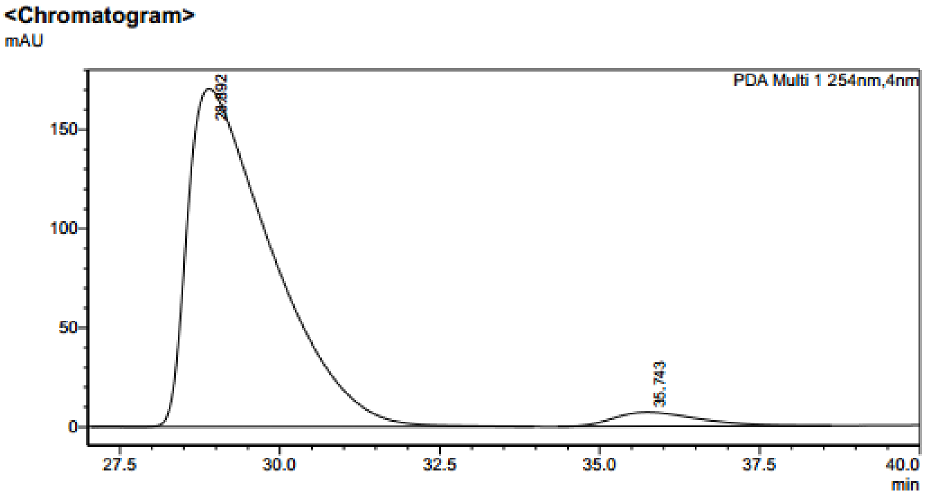

<Peak Table>

PDA Ch1 254nm

Peak#	Ret. Time	Area	Height	Area%
1	28.892	15563494	170477	95.947
2	35.743	657426	7147	4.053
Total		16220920	177624	100.000

Supplementary Fig. 242. HPLC Spectra of **3ae**

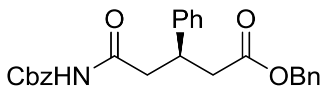

3af

<Chromatogram>

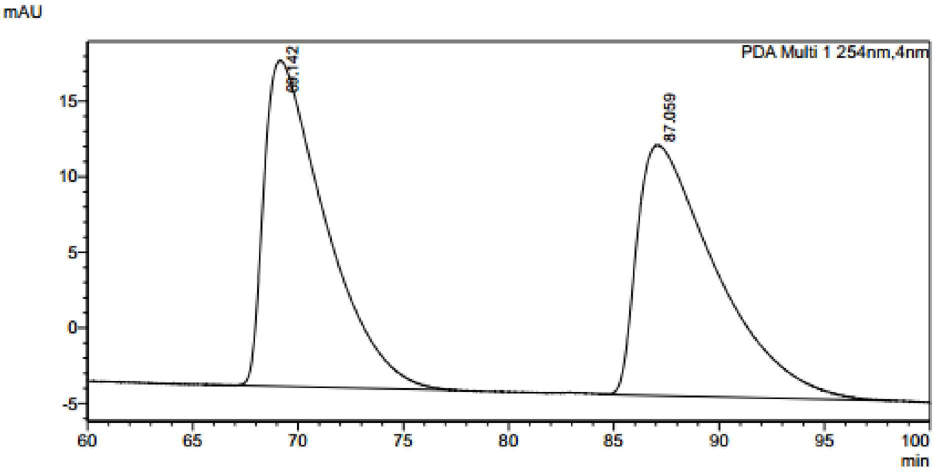

<Peak Table>

PDA Ch1 254nm

Peak#	Ret. Time	Area	Height	Area%
1	69.142	4373986	21572	50.263
2	87.059	4328222	16569	49.737
Total		8702209	38142	100.000

Supplementary Fig. 243. HPLC Spectra of racemic **3af**

<Chromatogram>

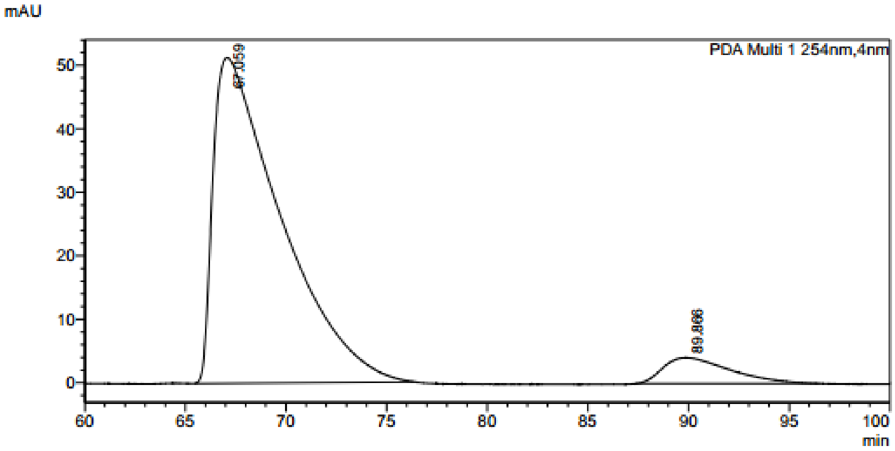

<Peak Table>

PDA Ch1 254nm

Peak#	Ret. Time	Area	Height	Area%
1	67.059	12006180	51268	92.673
2	89.866	949239	4093	7.327
Total		12955419	55362	100.000

Supplementary Fig. 244. HPLC Spectra of **3af**

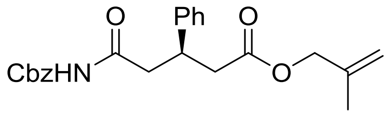

3ag

<Chromatogram>

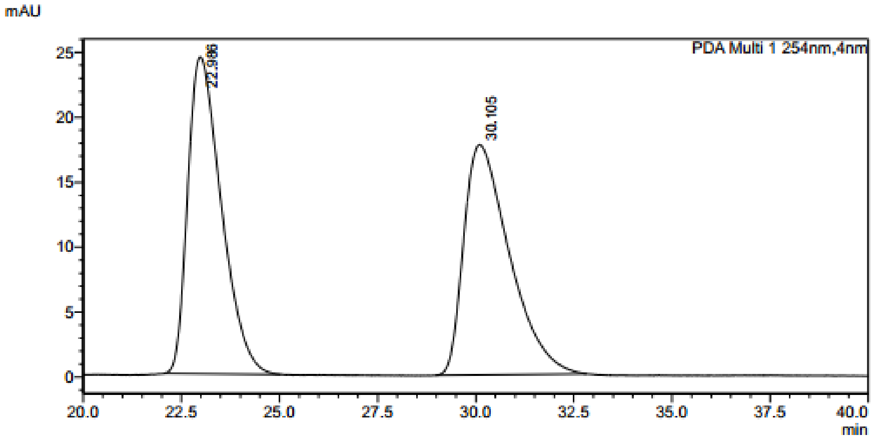

<Peak Table>

PDA Ch1 254nm

Peak#	Ret. Time	Area	Height	Area%
1	22.986	1455315	24396	50.300
2	30.105	1437937	17736	49.700
Total		2893252	42132	100.000

Supplementary Fig. 245. HPLC Spectra of racemic **3ag**

<Chromatogram>

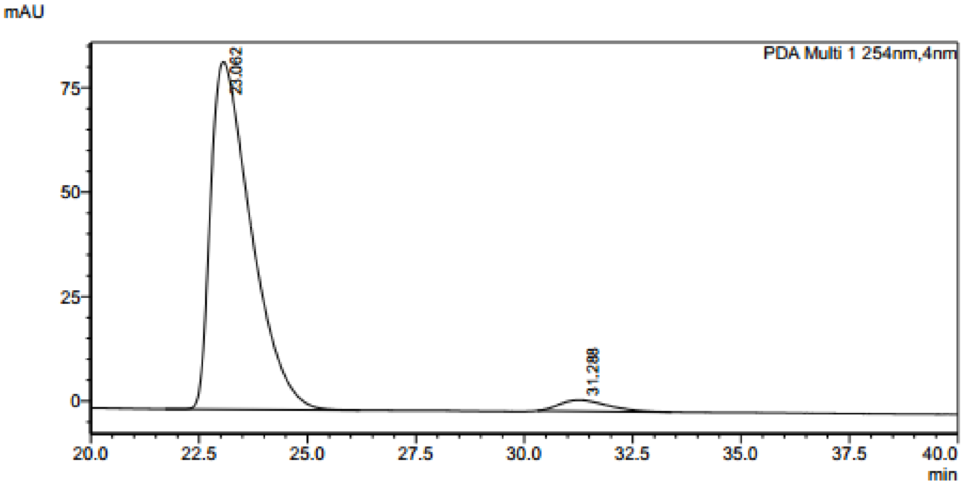

<Peak Table>

PDA Ch1 254nm

Peak#	Ret. Time	Area	Height	Area%
1	23.062	5411611	83202	96.372
2	31.288	203718	2637	3.628
Total		5615329	85839	100.000

Supplementary Fig. 246. HPLC Spectra of **3ag**

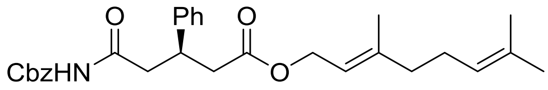

3ah

<Chromatogram>

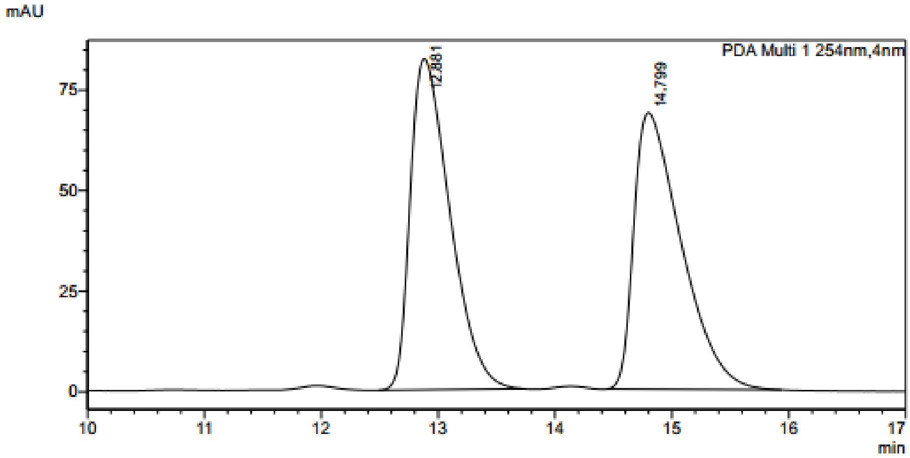

<Peak Table>

PDA Ch1 254nm

Peak#	Ret. Time	Area	Height	Area%
1	12.881	1944263	82303	50.076
2	14.799	1938365	68705	49.924
Total		3882628	151008	100.000

Supplementary Fig. 247. HPLC Spectra of racemic **3ah**

<Chromatogram>

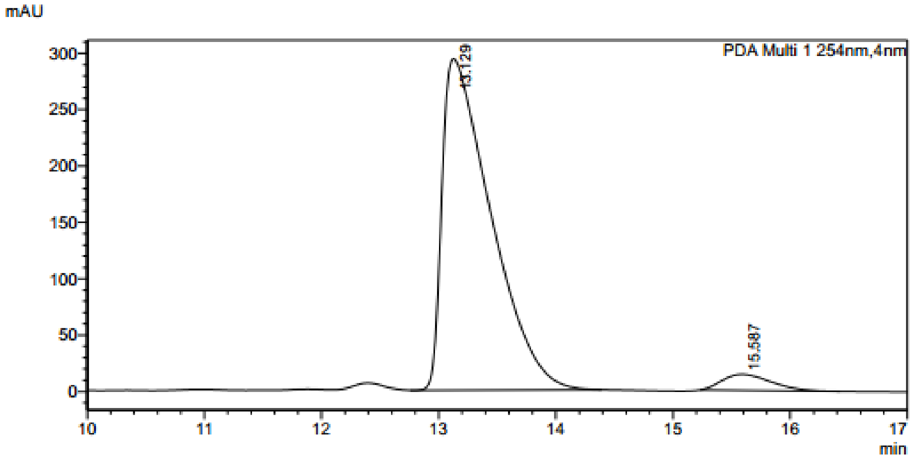

<Peak Table>

PDA Ch1 254nm

Peak#	Ret. Time	Area	Height	Area%
1	13.129	8551409	294070	95.446
2	15.587	408036	14172	4.554
Total		8959445	308241	100.000

Supplementary Fig. 248. HPLC Spectra of **3ah**

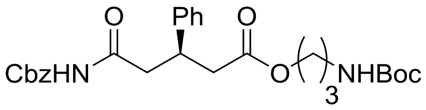

3ai

<Chromatogram>

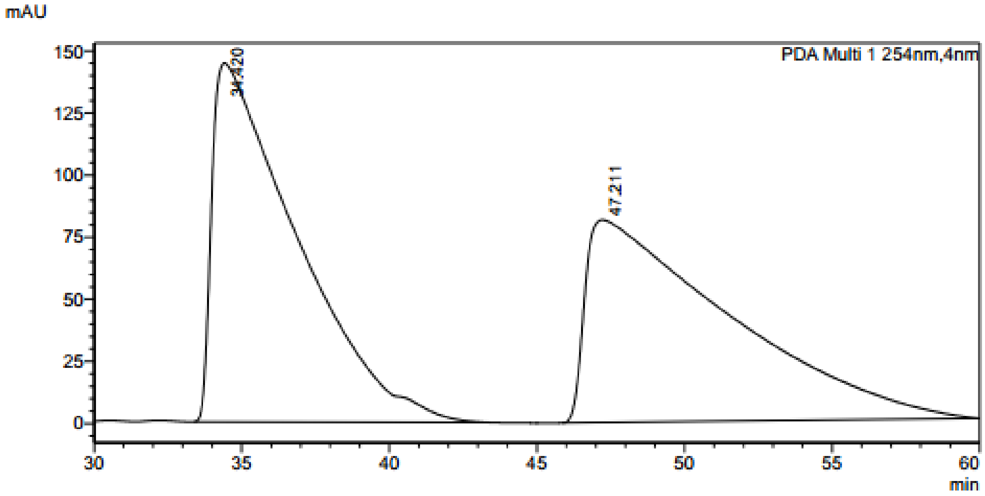

<Peak Table>

PDA Ch1 254nm

Peak#	Ret. Time	Area	Height	Area%
1	34.420	28789304	144448	51.023
2	47.211	27634979	81547	48.977
Total		56424283	225995	100.000

Supplementary Fig. 249. HPLC Spectra of racemic **3ai**

<Chromatogram>

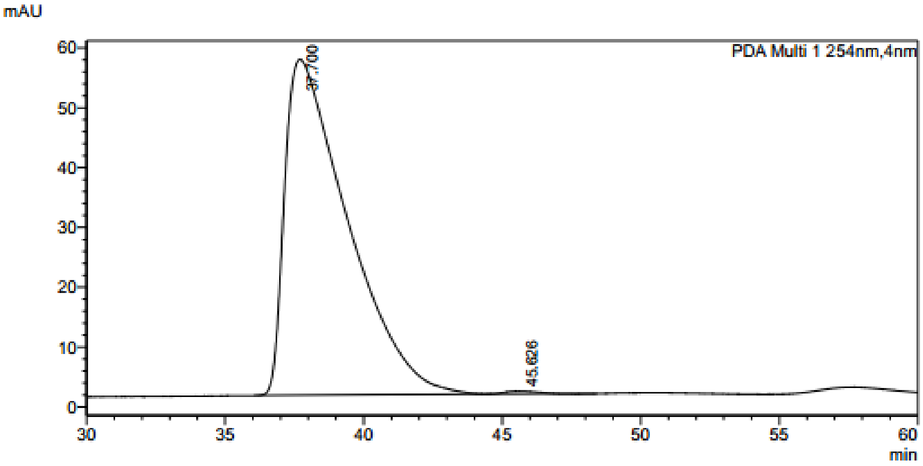

<Peak Table>

PDA Ch1 254nm

Peak#	Ret. Time	Area	Height	Area%
1	37.700	8871010	56132	99.606
2	45.626	35073	456	0.394
Total		8906083	56589	100.000

Supplementary Fig. 250. HPLC Spectra of **3ai**

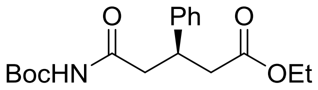

3aj

<Chromatogram>

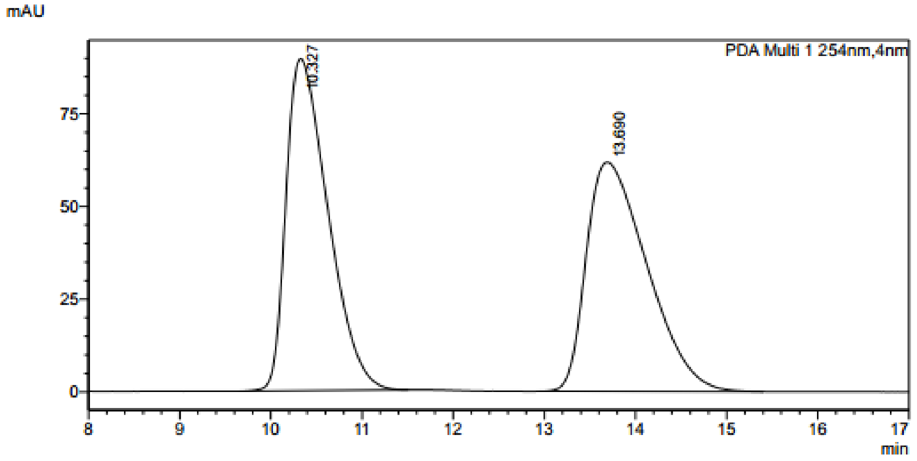

<Peak Table>

PDA Ch1 254nm

Peak#	Ret. Time	Area	Height	Area%
1	10.327	2863918	89574	49.980
2	13.690	2866230	61791	50.020
Total		5730148	151365	100.000

Supplementary Fig. 251. HPLC Spectra of racemic **3aj**

<Chromatogram>

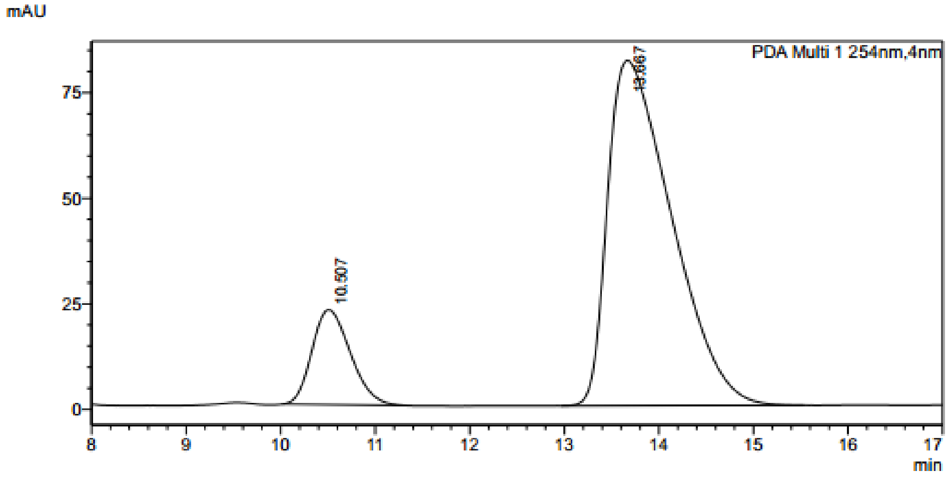

<Peak Table>

PDA Ch1 254nm

Peak#	Ret. Time	Area	Height	Area%
1	10.507	635275	22462	14.156
2	13.667	3852475	81739	85.844
Total		4487750	104201	100.000

Supplementary Fig. 252. HPLC Spectra of **3aj**

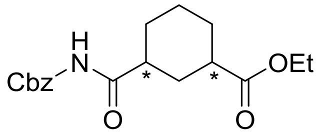

3al

<Chromatogram>

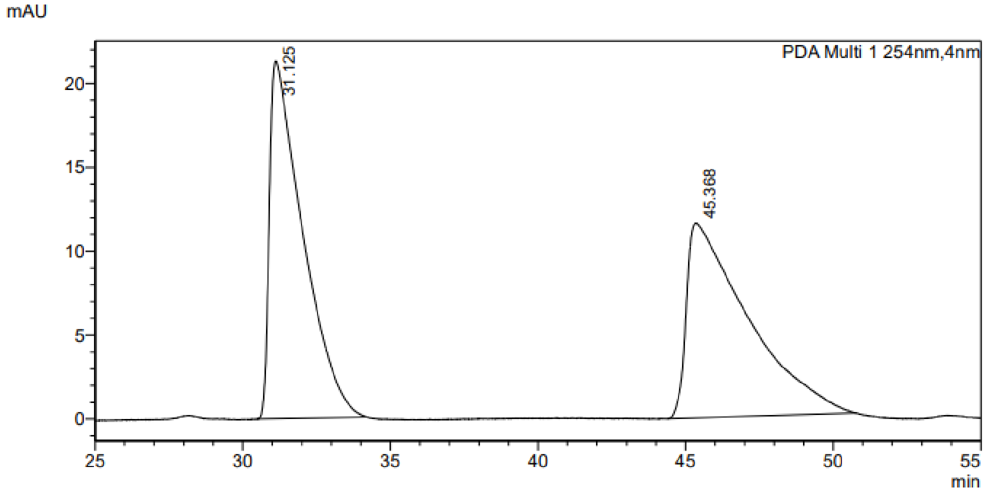

<Peak Table>

PDA Ch1 254nm

Peak#	Ret. Time	Area	Area%	Height
1	31.125	1672270	49.921	21300
2	45.368	1677565	50.079	11582
Total		3349835	100.000	32882

Supplementary Fig. 253. HPLC Spectra of racemic **3al**

<Chromatogram>

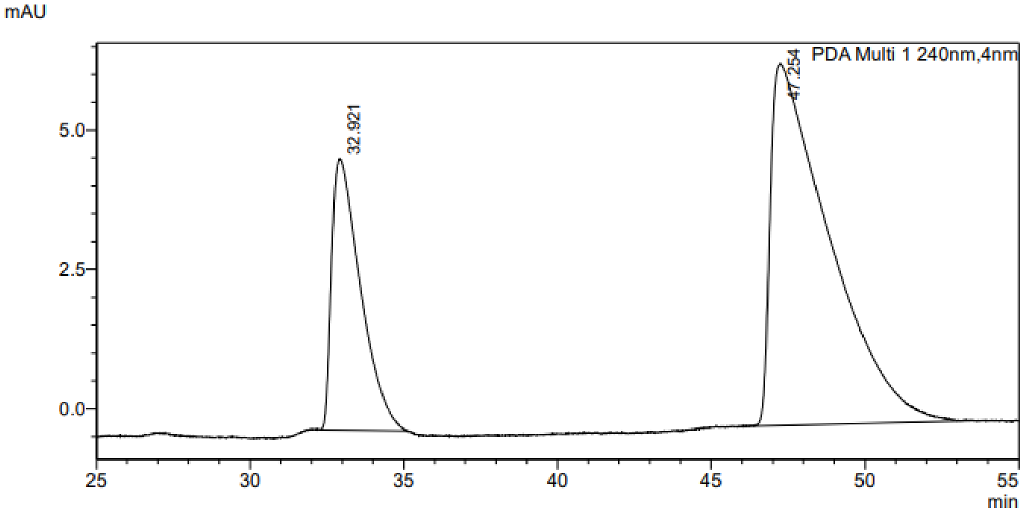

<Peak Table>

PDA Ch1 240nm

Peak#	Ret. Time	Area	Area%	Height
1	32.921	317549	26.792	4869
2	47.254	867709	73.208	6495
Total		1185259	100.000	11363

Supplementary Fig. 254. HPLC Spectra of **3al**

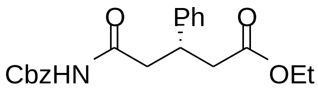

3a'

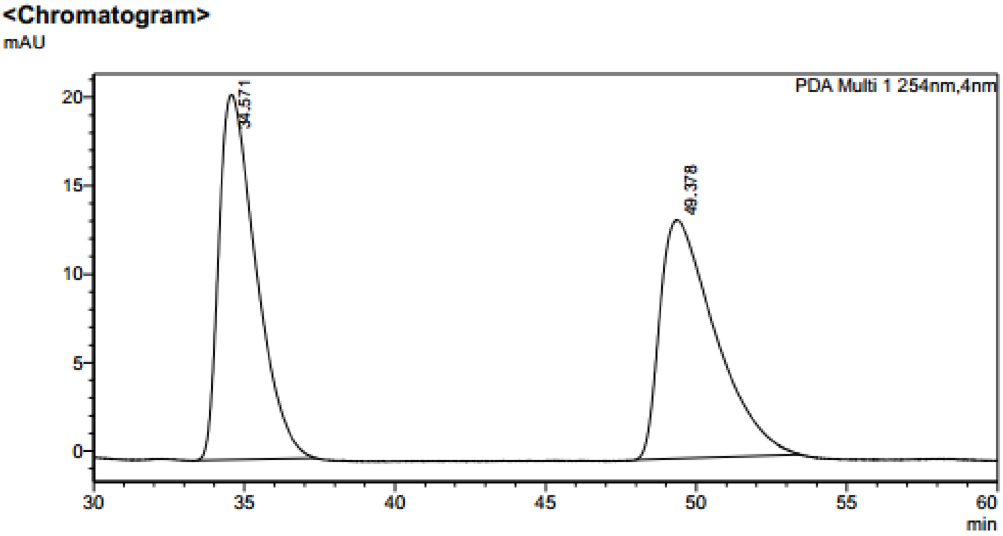

<Peak Table>

PDA Ch1 254nm

Peak#	Ret. Time	Area	Height	Area%
1	34.571	1771393	20634	50.659
2	49.378	1725290	13466	49.341
Total		3496684	34100	100.000

Supplementary Fig. 255. HPLC Spectra of racemic **3a'**

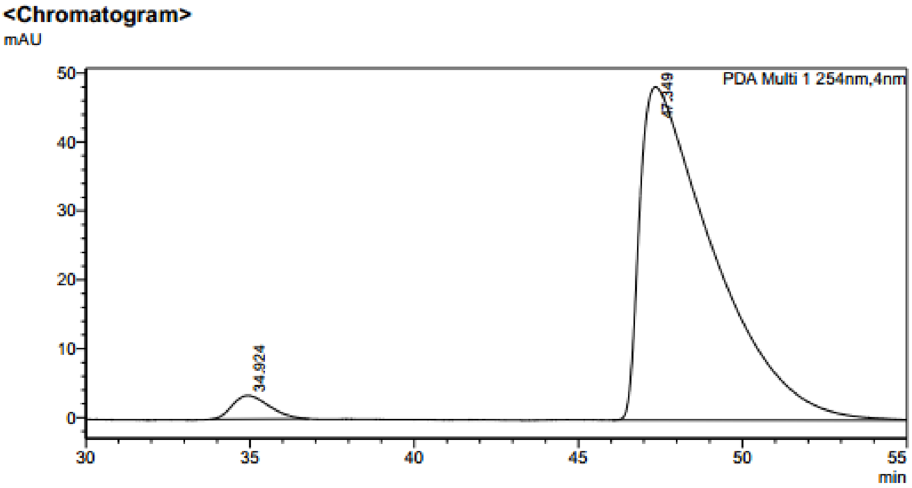

<Peak Table>

PDA Ch1 254nm

Peak#	Ret. Time	Area	Height	Area%
1	34.924	269417	3433	3.438
2	47.349	7567628	48359	96.562
Total		7837044	51792	100.000

Supplementary Fig. 256. HPLC Spectra of **3a'**

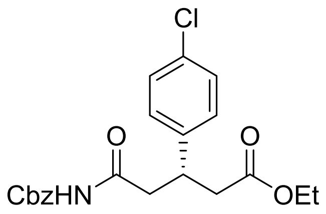

3i'

<Chromatogram>

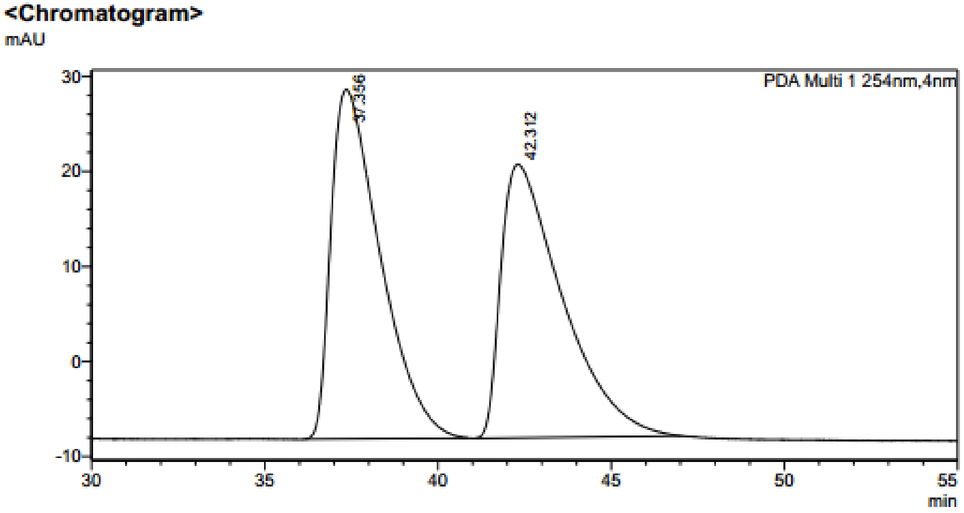

<Peak Table>

PDA Ch1 254nm

Peak#	Ret. Time	Area	Height	Area%
1	37.356	3640179	36737	50.636
2	42.312	3548785	28754	49.364
Total		7188963	65491	100.000

Supplementary Fig. 257. HPLC Spectra of racemic **3i'**

<Chromatogram>

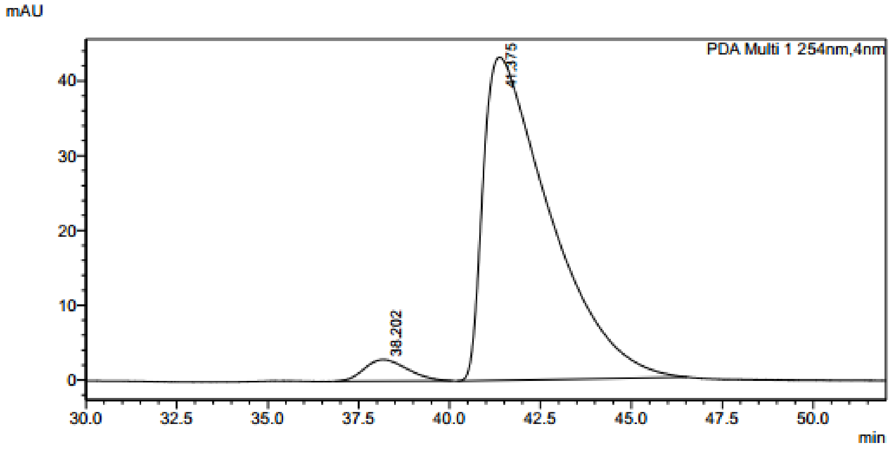

<Peak Table>

PDA Ch1 254nm

Peak#	Ret. Time	Area	Height	Area%
1	38.202	236271	2850	4.085
2	41.375	5547340	43191	95.915
Total		5783612	46041	100.000

Supplementary Fig. 258. HPLC Spectra of **3i'**

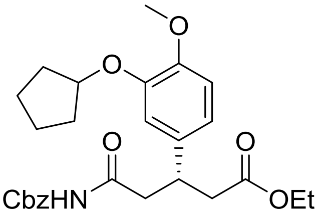

3j'

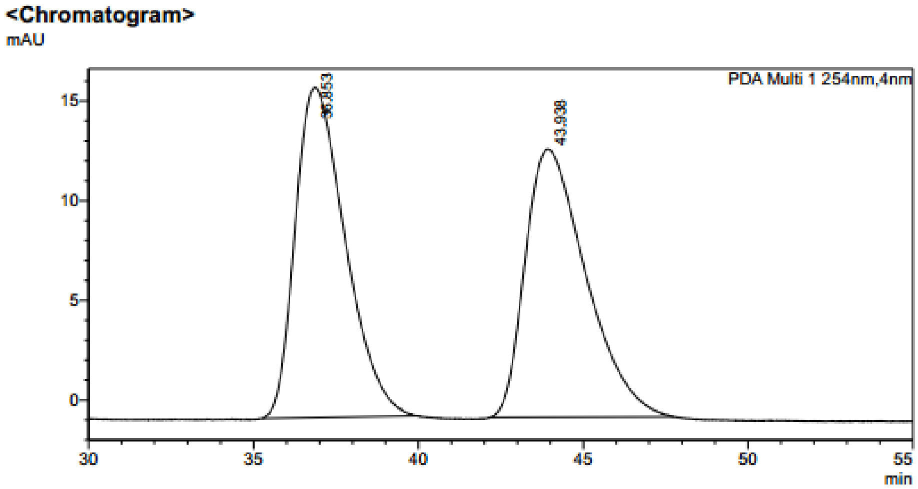

<Peak Table>

PDA Ch1 254nm

Peak#	Ret. Time	Area	Height	Area%
1	36.853	1747760	16562	50.321
2	43.938	1725450	13436	49.679
Total		3473210	29999	100.000

Supplementary Fig. 259. HPLC Spectra of racemic **3j'**

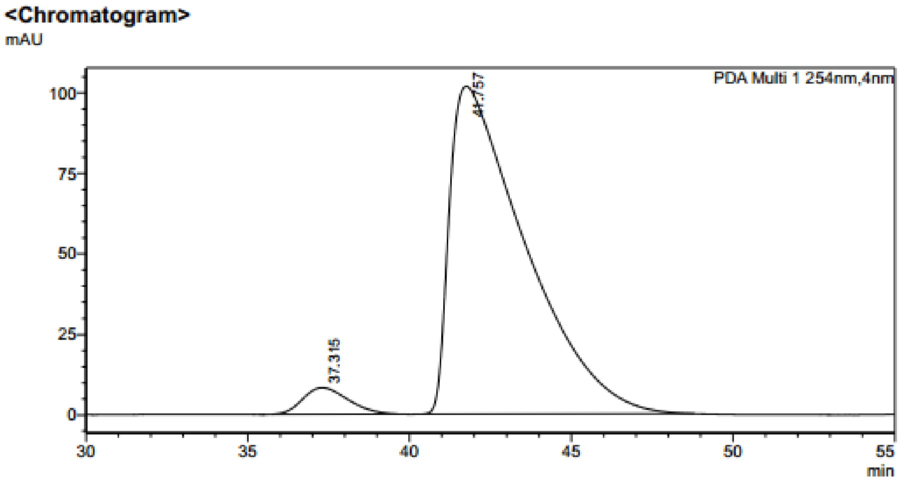

<Peak Table>

PDA Ch1 254nm

Peak#	Ret. Time	Area	Height	Area%
1	37.315	795555	8210	4.586
2	41.757	16552438	101751	95.414
Total		17347993	109961	100.000

Supplementary Fig. 260. HPLC Spectra of **3j'**

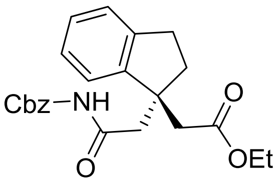

5a

<Chromatogram>

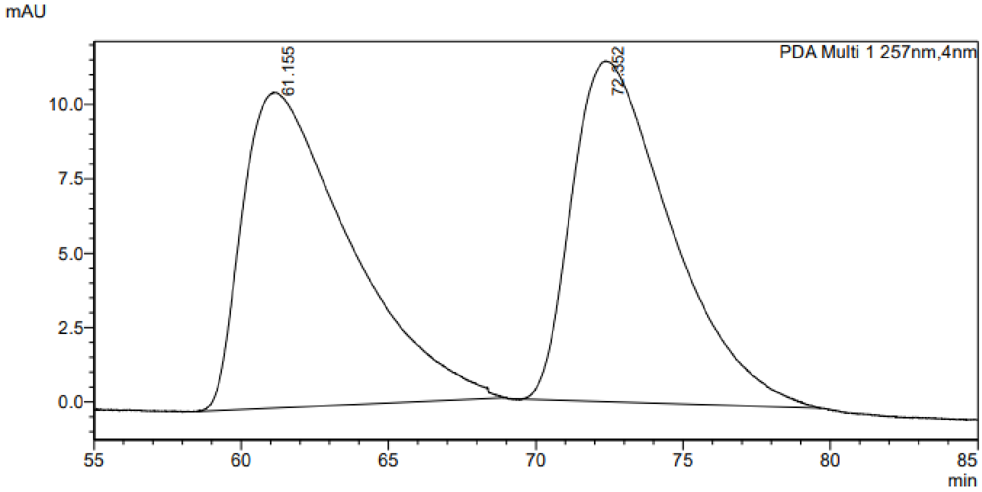

<Peak Table>

PDA Ch1 257nm

Peak#	Ret. Time	Area	Area%	Height
1	61.155	2699972	50.241	10616
2	72.352	2674093	49.759	11444
Total		5374066	100.000	22059

Supplementary Fig. 261. HPLC Spectra of racemic **5a**

<Chromatogram>

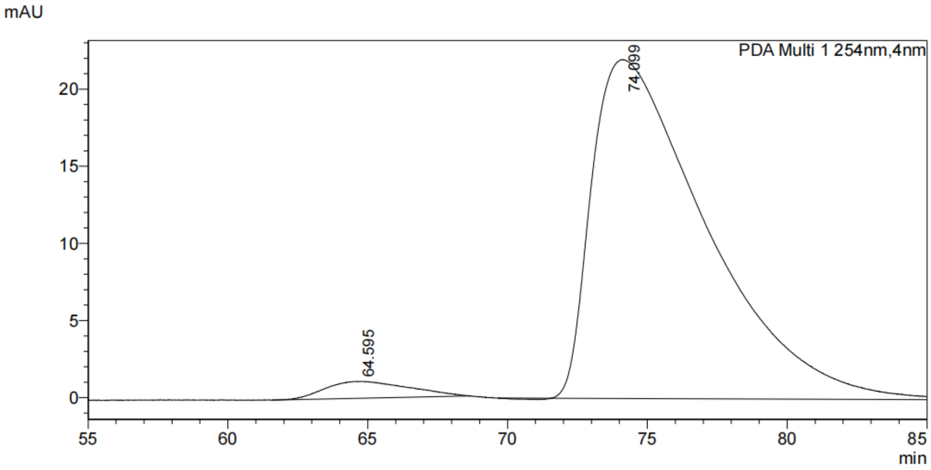

<Peak Table>

PDA Ch1 254nm

Peak#	Ret. Time	Area	Area%	Height
1	64.595	227937	3.496	1100
2	74.099	6292747	96.504	21988
Total		6520684	100.000	23088

Supplementary Fig. 262. HPLC Spectra of **5a**

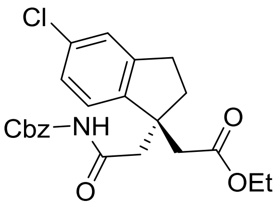

5b

<Chromatogram>

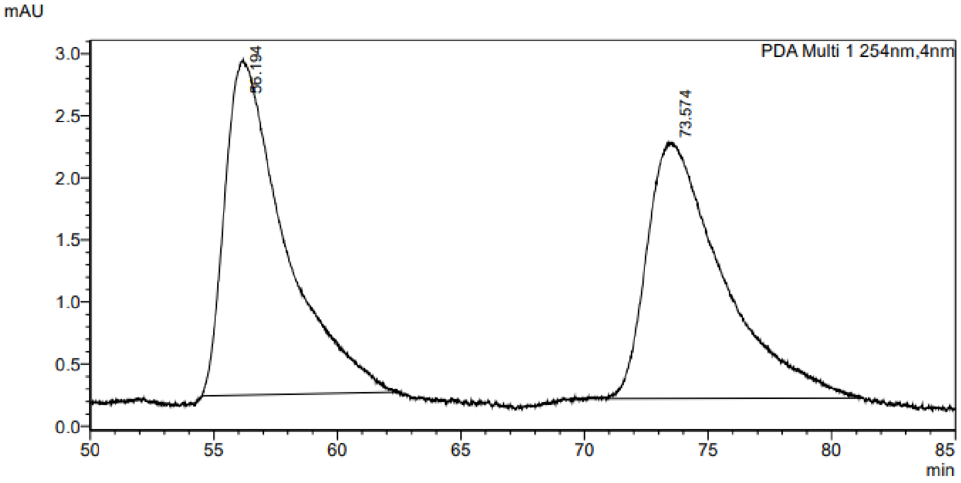

<Peak Table>

PDA Ch1 254nm

Peak#	Ret. Time	Area	Area%	Height
1	56.194	454733	50.656	2699
2	73.574	442957	49.344	2061
Total		897690	100.000	4760

Supplementary Fig. 263. HPLC Spectra of racemic **5b**

<Chromatogram>

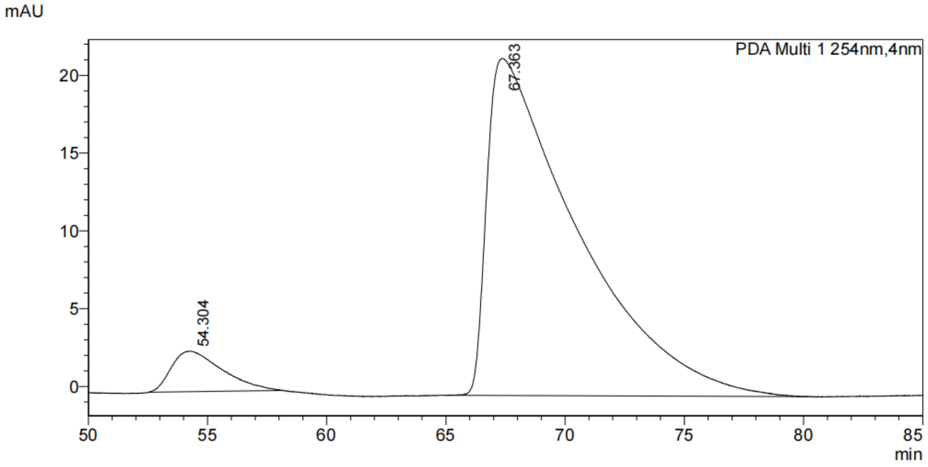

<Peak Table>

PDA Ch1 254nm

Peak#	Ret. Time	Area	Area%	Height
1	54.304	387096	6.339	2602
2	67.363	5719707	93.661	21656
Total		6106802	100.000	24259

Supplementary Fig. 264. HPLC Spectra of **5b**

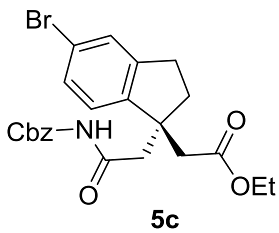

5c

<Chromatogram>

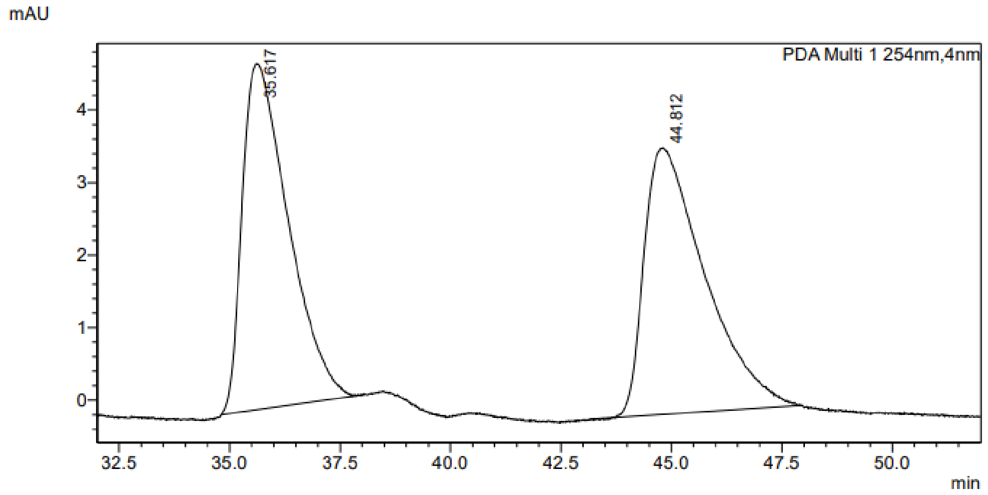

<Peak Table>

PDA Ch1 254nm

Peak#	Ret. Time	Area	Area%	Height
1	35.617	346361	49.673	4770
2	44.812	350917	50.327	3672
Total		697278	100.000	8442

Supplementary Fig. 265. HPLC Spectra of racemic **5c**

<Chromatogram>

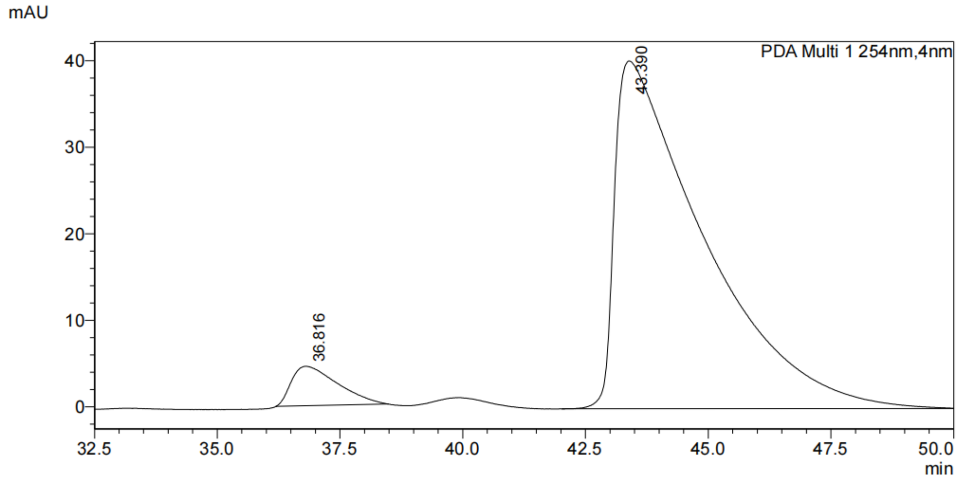

<Peak Table>

PDA Ch1 254nm

Peak#	Ret. Time	Area	Area%	Height
1	36.816	299555	5.554	4571
2	43.390	5093880	94.446	40211
Total		5393435	100.000	44782

Supplementary Fig. 266. HPLC Spectra of **5c**

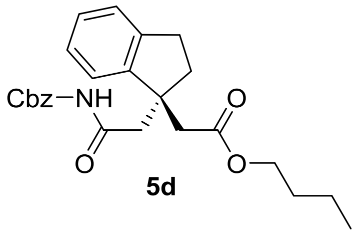

<Chromatogram>

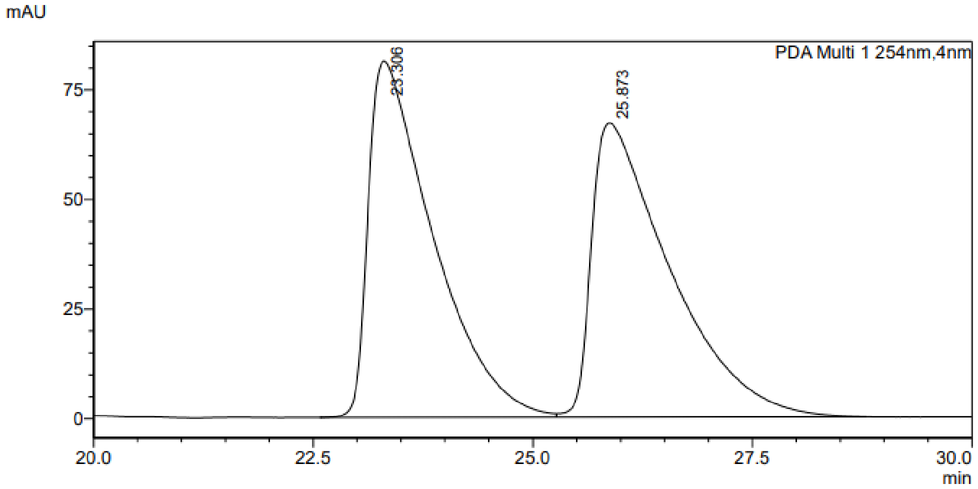

<Peak Table>

PDA Ch1 254nm

Peak#	Ret. Time	Area	Area%	Height
1	23.306	4167211	50.037	81206
2	25.873	4161093	49.963	67068
Total		8328304	100.000	148274

Supplementary Fig. 267. HPLC Spectra of racemic **5d**

<Chromatogram>

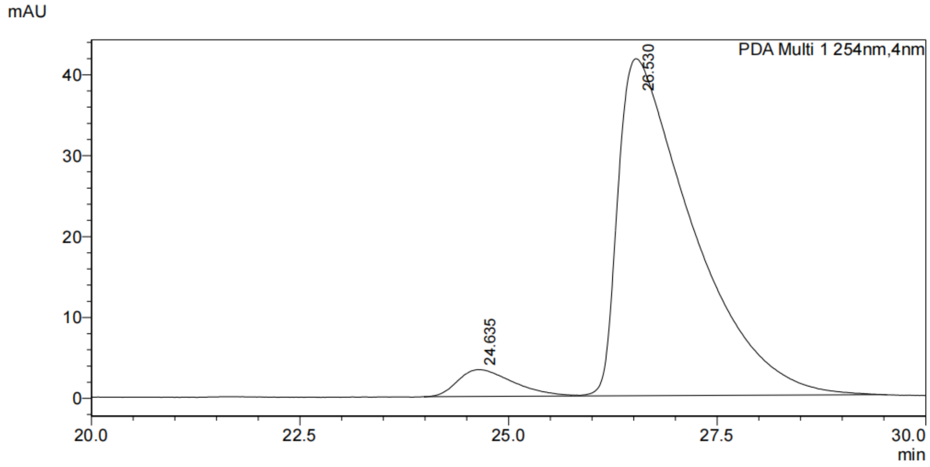

<Peak Table>

PDA Ch1 254nm

Peak#	Ret. Time	Area	Area%	Height
1	24.635	153827	5.593	3320
2	26.530	2596411	94.407	41670
Total		2750239	100.000	44990

Supplementary Fig. 268. HPLC Spectra of **5d**

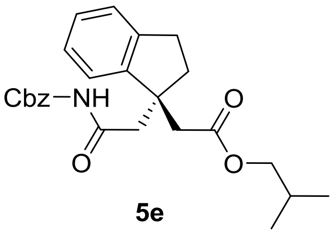

5e

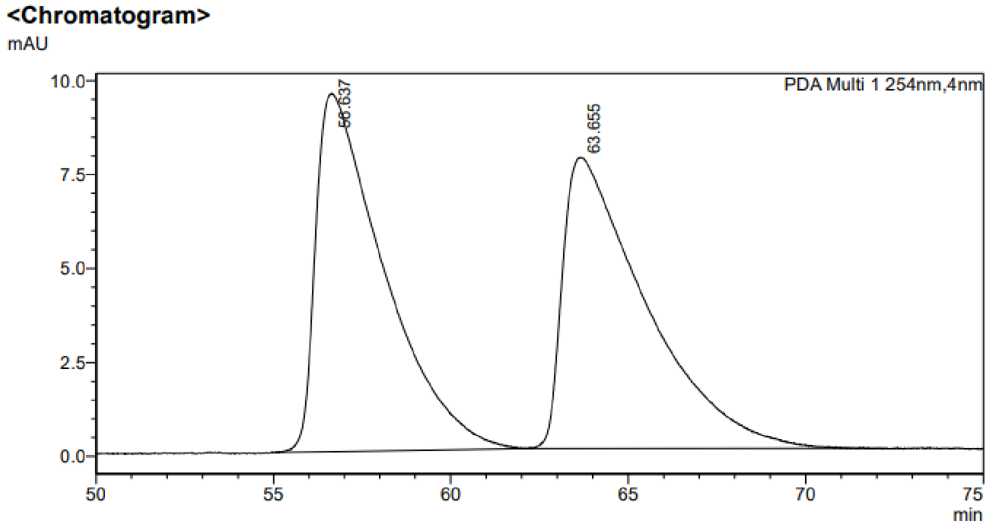

<Peak Table>

PDA Ch1 254nm

Peak#	Ret. Time	Area	Area%	Height
1	56.637	1271006	50.243	9524
2	63.655	1258700	49.757	7736
Total		2529706	100.000	17260

Supplementary Fig. 269. HPLC Spectra of racemic **5e**

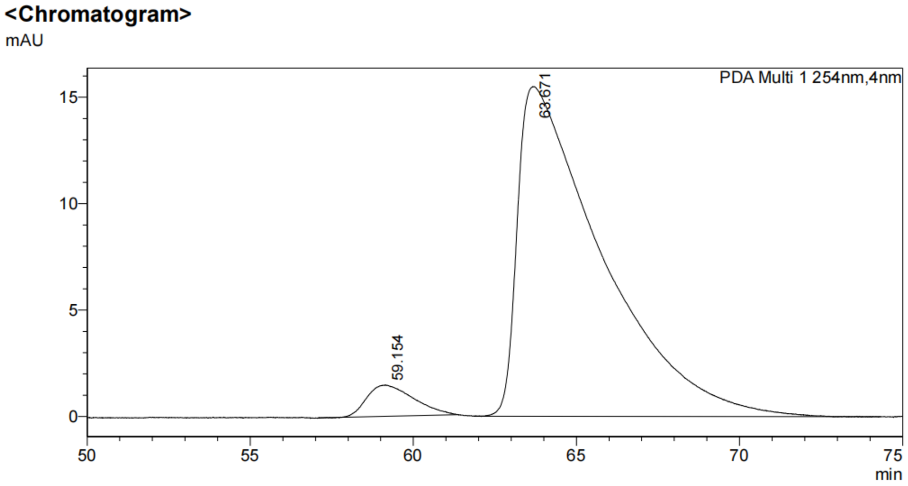

<Peak Table>

PDA Ch1 254nm

Peak#	Ret. Time	Area	Area%	Height
1	59.154	146633	4.988	1473
2	63.671	2792942	95.012	15493
Total		2939576	100.000	16965

Supplementary Fig. 270. HPLC Spectra of **5e**

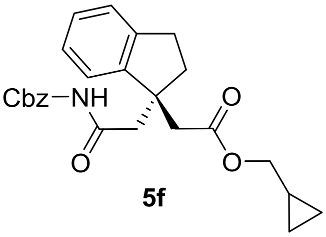

5f

<Chromatogram>

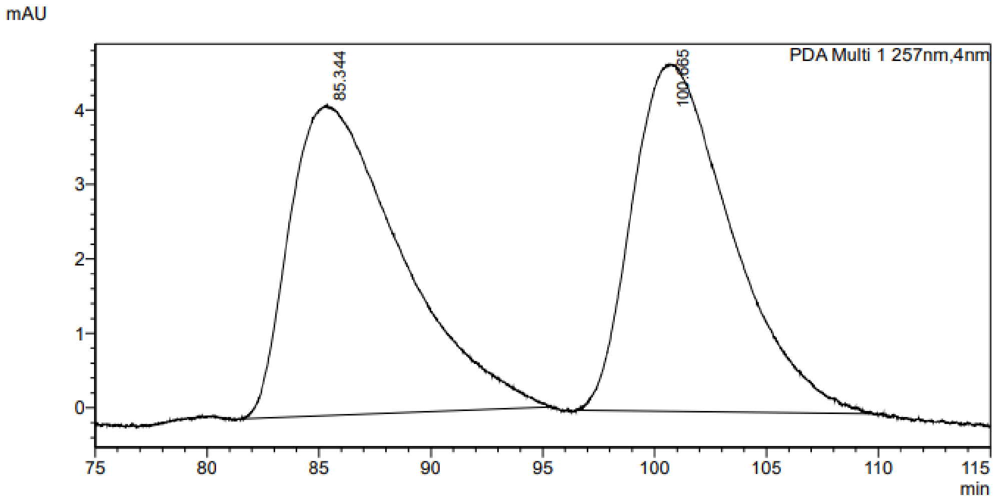

<Peak Table>

PDA Ch1 257nm

Peak#	Ret. Time	Area	Area%	Height
1	85.344	1444772	50.080	4159
2	100.665	1440175	49.920	4663
Total		2884947	100.000	8822

Supplementary Fig. 271. HPLC Spectra of racemic **5f**

<Chromatogram>

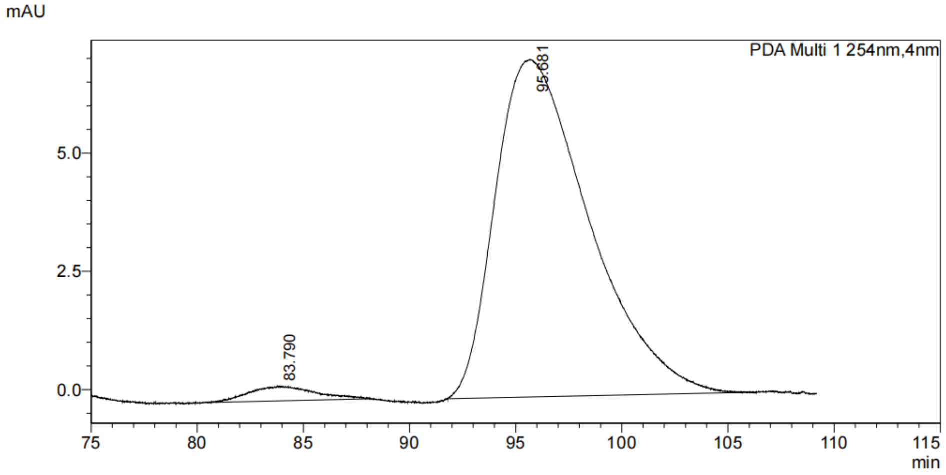

<Peak Table>

PDA Ch1 254nm

Peak#	Ret. Time	Area	Area%	Height
1	83.790	66866	2.956	316
2	95.681	2195403	97.044	7133
Total		2262269	100.000	7449

Supplementary Fig. 272. HPLC Spectra of **5f**

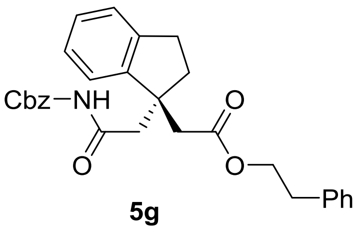

5g

<Chromatogram>

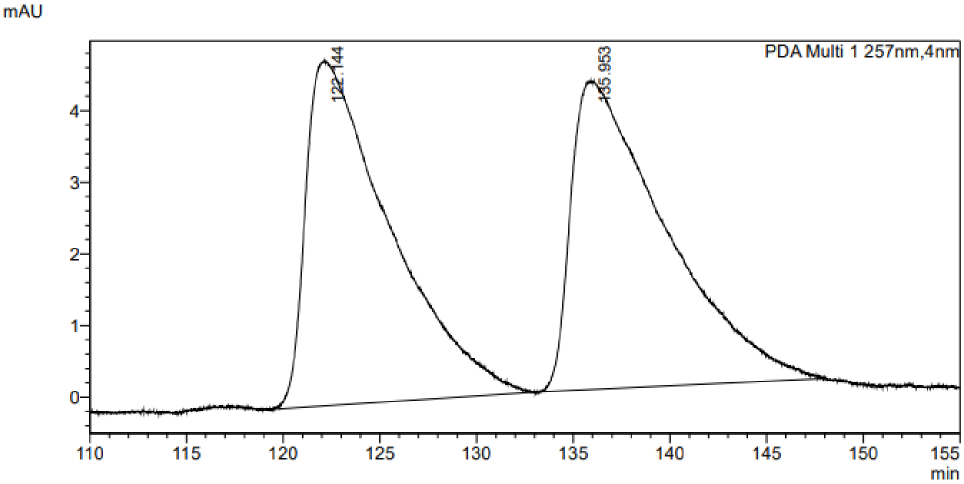

<Peak Table>

PDA Ch1 257nm

Peak#	Ret. Time	Area	Area%	Height
1	122.144	1452973	49.881	4823
2	135.953	1459904	50.119	4304
Total		2912877	100.000	9128

Supplementary Fig. 273. HPLC Spectra of racemic **5g**

<Chromatogram>

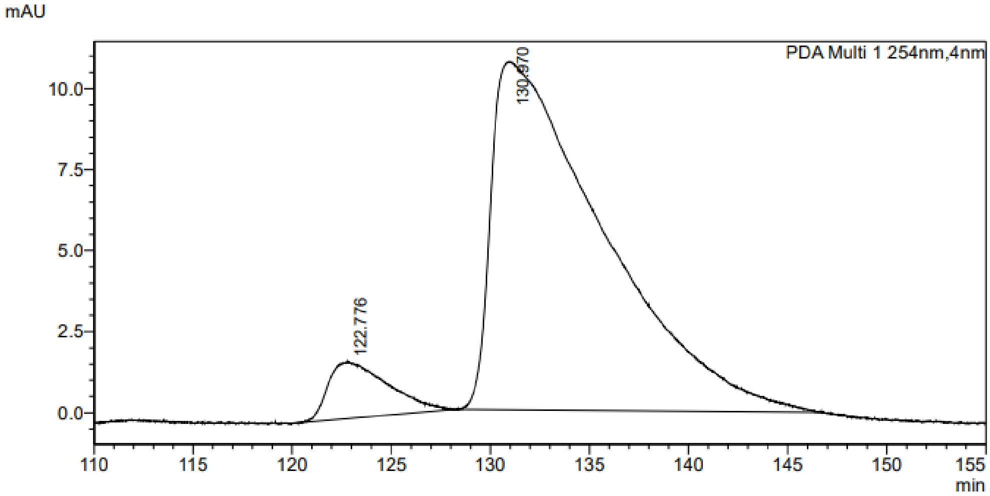

<Peak Table>

PDA Ch1 254nm

Peak#	Ret. Time	Area	Area%	Height
1	122.776	364437	7.916	1757
2	130.970	4239128	92.084	10735
Total		4603565	100.000	12493

Supplementary Fig. 274. HPLC Spectra of **5g**

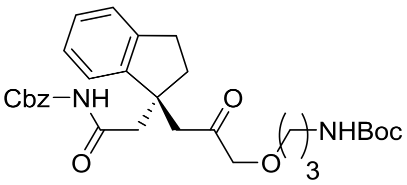

5h

<Chromatogram>

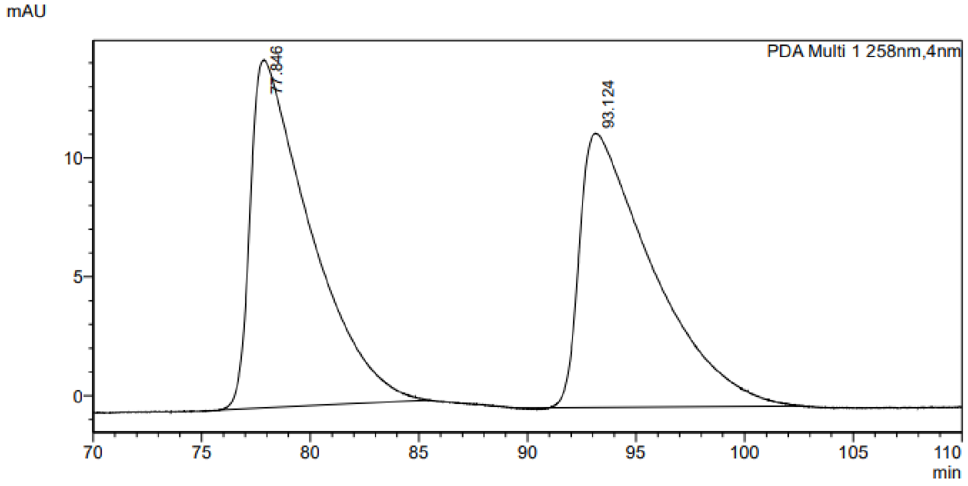

<Peak Table>

PDA Ch1 258nm

Peak#	Ret. Time	Area	Area%	Height
1	77.846	2818865	51.003	14624
2	93.124	2708030	48.997	11525
Total		5526896	100.000	26149

Supplementary Fig. 275. HPLC Spectra of racemic **5h**

<Chromatogram>

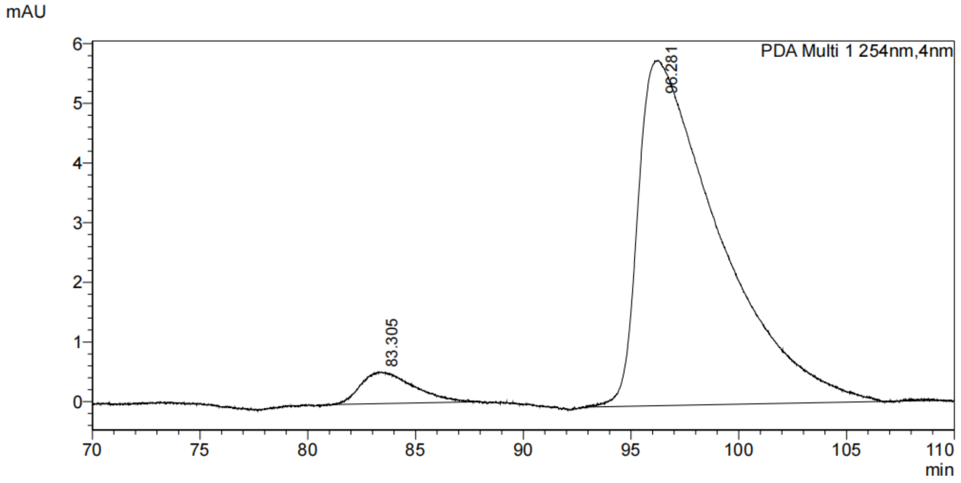

<Peak Table>

PDA Ch1 254nm

Peak#	Ret. Time	Area	Area%	Height
1	83.305	89512	5.577	535
2	96.281	1515432	94.423	5785
Total		1604944	100.000	6320

Supplementary Fig. 276. HPLC Spectra of **5h**

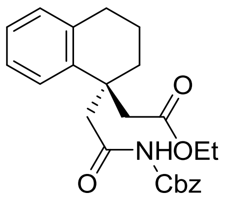

5i

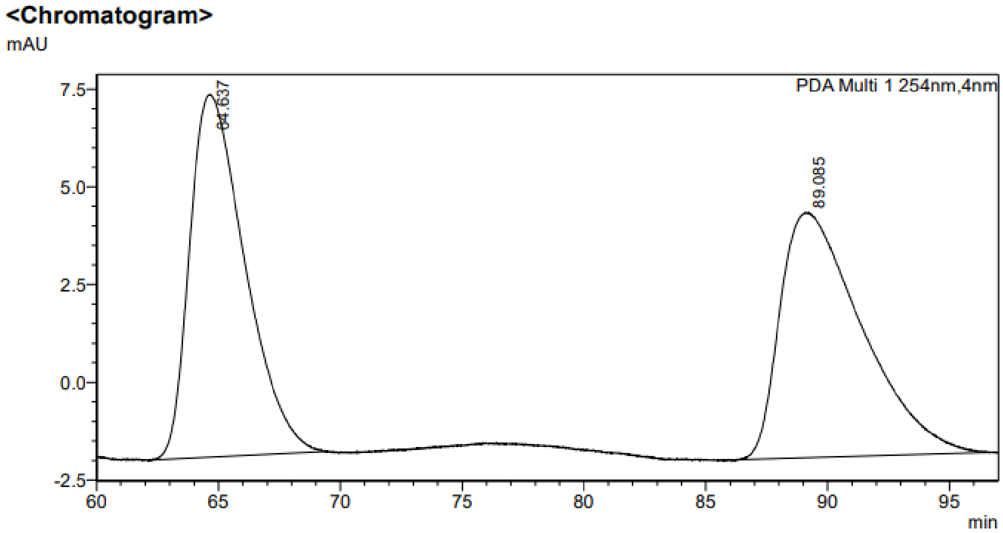

<Peak Table>

PDA Ch1 254nm

Peak#	Ret. Time	Area	Area%	Height
1	64.637	1459402	49.975	9264
2	89.085	1460835	50.025	6263
Total		2920237	100.000	15527

Supplementary Fig. 277. HPLC Spectra of racemic **5i**

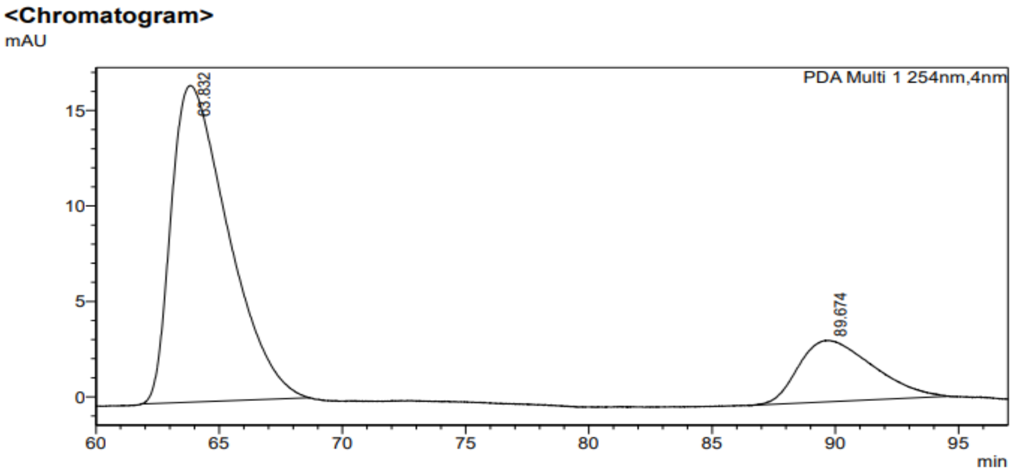

<Peak Table>

PDA Ch1 254nm

Peak#	Ret. Time	Area	Area%	Height
1	63.832	2675870	79.889	16586
2	89.674	673605	20.111	3218
Total		3349474	100.000	19803

Supplementary Fig. 278. HPLC Spectra of **5i**

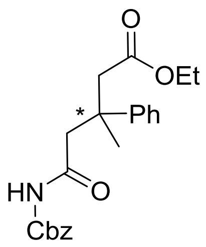

5j

<Chromatogram>

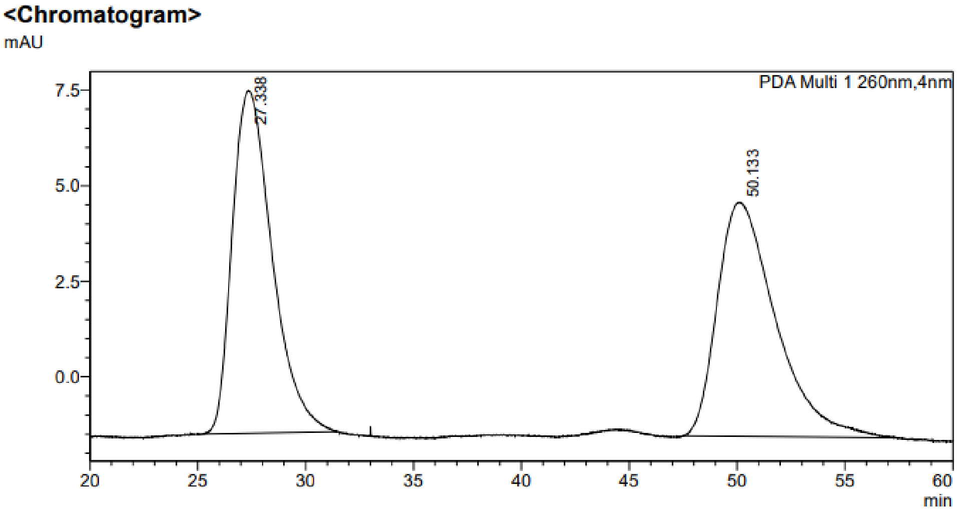

<Peak Table>

PDA Ch1 260nm

Peak#	Ret. Time	Area	Area%	Height
1	27.338	1153178	50.020	8952
2	50.133	1152242	49.980	6128
Total		2305420	100.000	15080

Supplementary Fig. 279. HPLC Spectra of racemic **5j**

<Chromatogram>

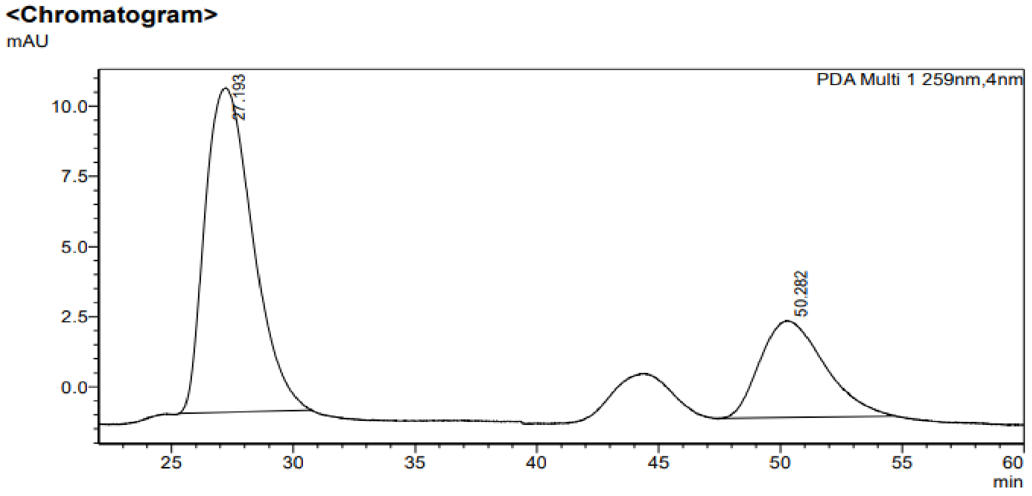

<Peak Table>

PDA Ch1 259nm

Peak#	Ret. Time	Area	Area%	Height
1	27.193	1575194	71.227	11555
2	50.282	636311	28.773	3450
Total		2211505	100.000	15004

Supplementary Fig. 280. HPLC Spectra of **5j**

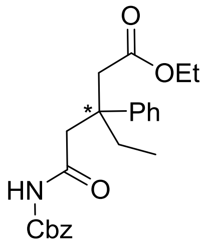

5k

<Chromatogram>

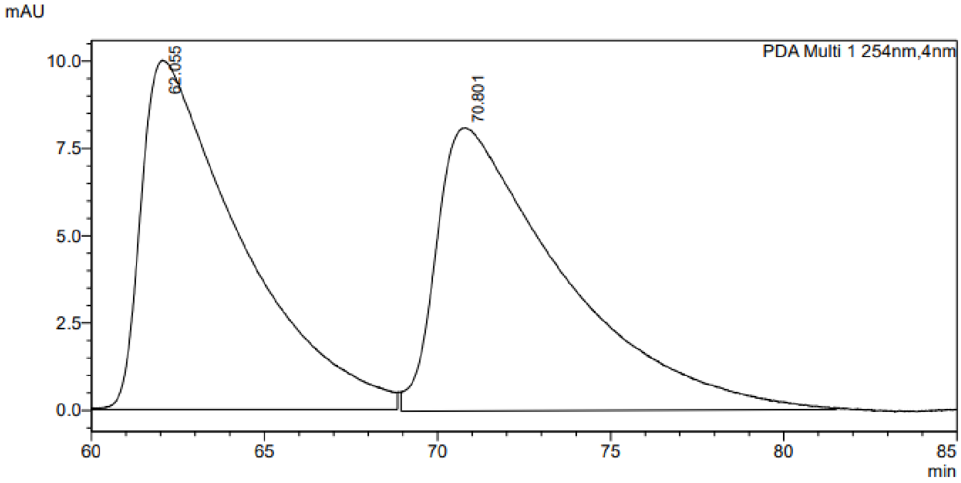

<Peak Table>

PDA Ch1 254nm

Peak#	Ret. Time	Area	Area%	Height
1	62.055	1989641	49.149	9998
2	70.801	2058538	50.851	8108
Total		4048179	100.000	18106

Supplementary Fig. 281. HPLC Spectra of racemic **5k**

<Chromatogram>

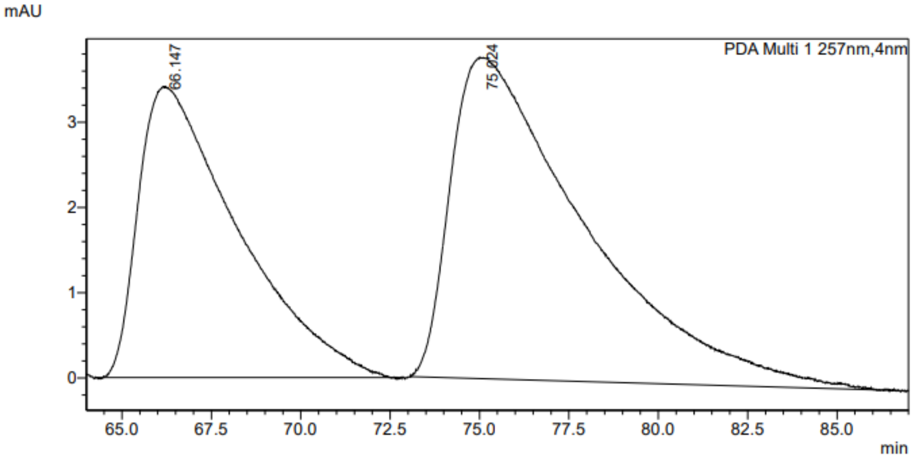

<Peak Table>

PDA Ch1 257nm

Peak#	Ret. Time	Area	Area%	Height
1	66.147	658063	40.101	3410
2	75.024	982944	59.899	3764
Total		1641007	100.000	7174

Supplementary Fig. 282. HPLC Spectra of **5k**

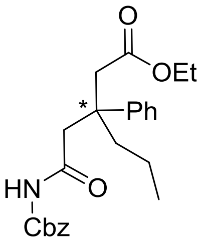

5l

<Chromatogram>

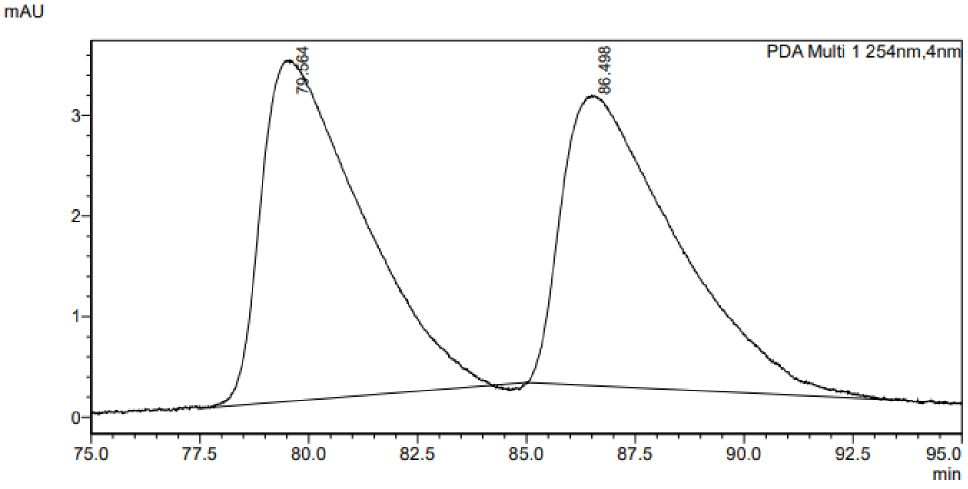

<Peak Table>

PDA Ch1 254nm

Peak#	Ret. Time	Area	Area%	Height
1	79.564	541698	50.608	3389
2	86.498	528679	49.392	2887
Total		1070377	100.000	6276

Supplementary Fig. 283. HPLC Spectra of racemic **5l**

<Chromatogram>

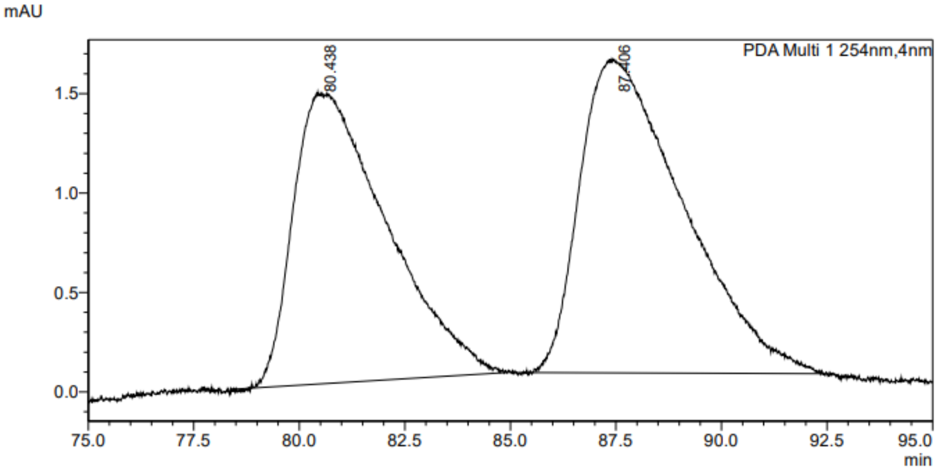

<Peak Table>

PDA Ch1 254nm

Peak#	Ret. Time	Area	Area%	Height
1	80.438	225459	45.513	1468
2	87.406	269912	54.487	1579
Total		495371	100.000	3047

Supplementary Fig. 284. HPLC Spectra of **5l**

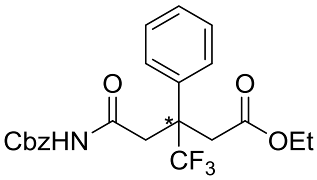

5m

<Chromatogram>

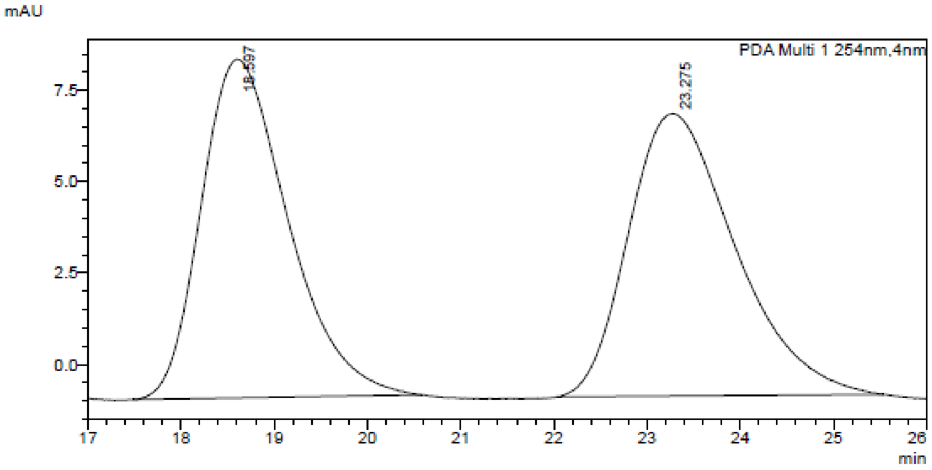

<Peak Table>

PDA Ch1 254nm

Peak#	Ret. Time	Area	Height	Area%
1	18.597	601089	9275	49.368
2	23.275	616491	7736	50.632
Total		1217580	17011	100.000

Supplementary Fig. 285. HPLC Spectra of racemic **5m**

<Chromatogram>

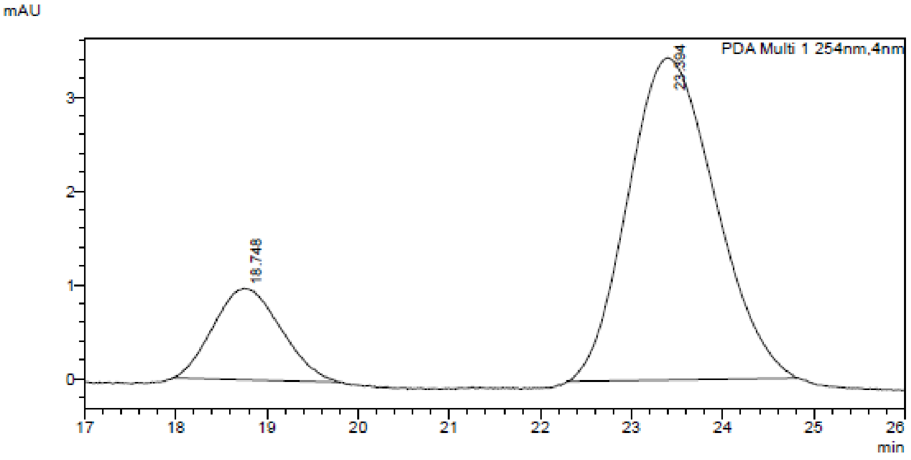

<Peak Table>

PDA Ch1 254nm

Peak#	Ret. Time	Area	Height	Area%
1	18.748	50020	973	17.968
2	23.394	228363	3432	82.032
Total		278384	4405	100.000

Supplementary Fig. 286. HPLC Spectra of **5m**

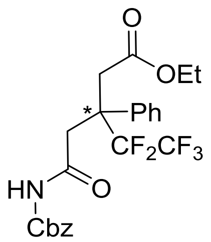

5n

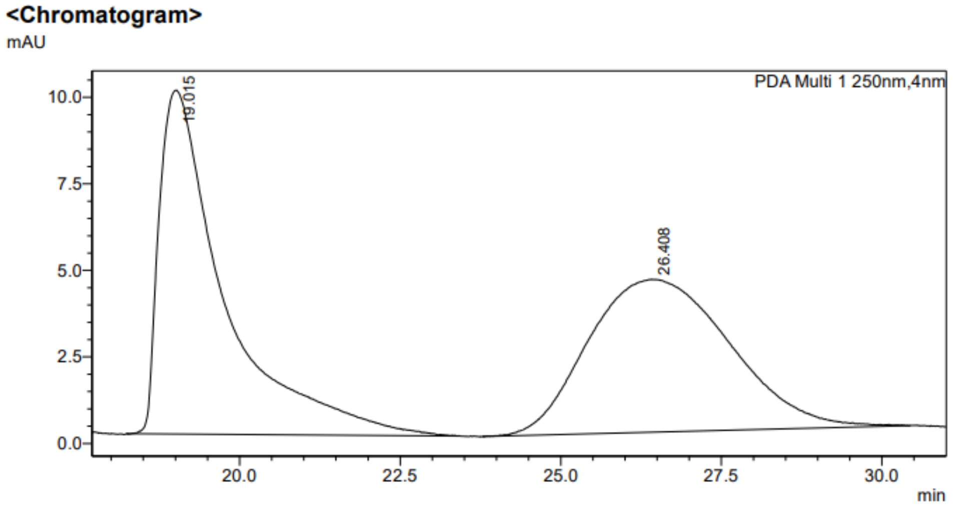

<Peak Table>

PDA Ch1 250nm

Peak#	Ret. Time	Area	Area%	Height
1	19.015	698461	50.747	9931
2	26.408	677885	49.253	4420
Total		1376346	100.000	14351

Supplementary Fig. 287. HPLC Spectra of racemic **5n**

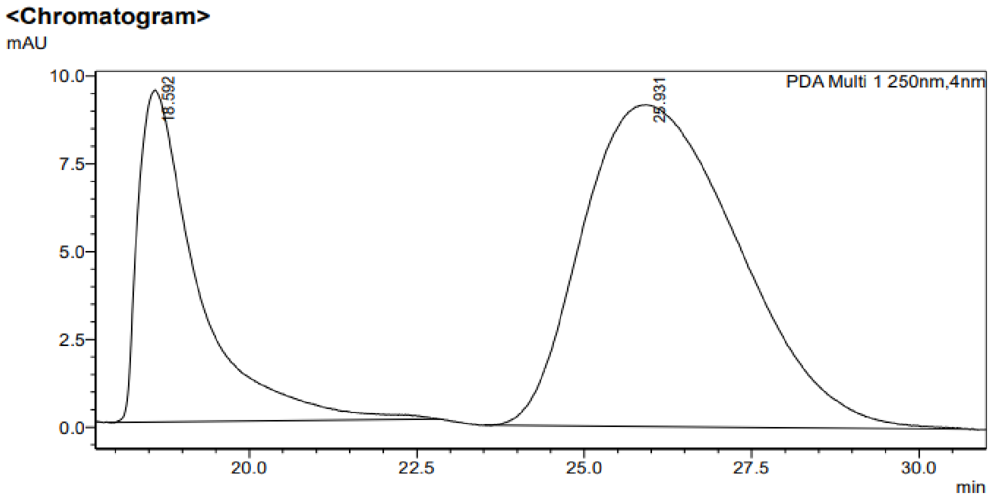

<Peak Table>

PDA Ch1 250nm

Peak#	Ret. Time	Area	Area%	Height
1	18.592	588028	28.389	9444
2	25.931	1483262	71.611	9164
Total		2071290	100.000	18607

Supplementary Fig. 288. HPLC Spectra of **5n**

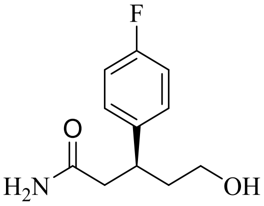

6

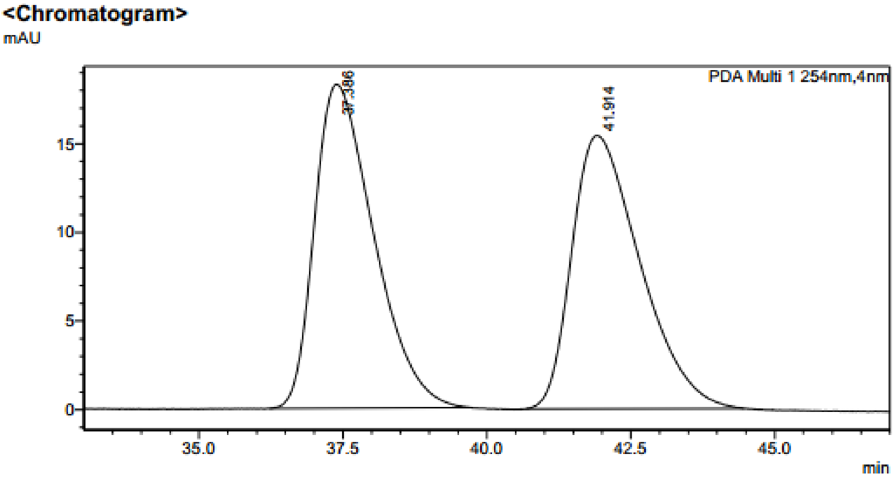

<Peak Table>

PDA Ch1 254nm

Peak#	Ret. Time	Area	Height	Area%
1	37.386	1306982	18244	50.643
2	41.914	1273786	15411	49.357
Total		2580768	33654	100.000

Supplementary Fig. 289. HPLC Spectra of racemic **6**

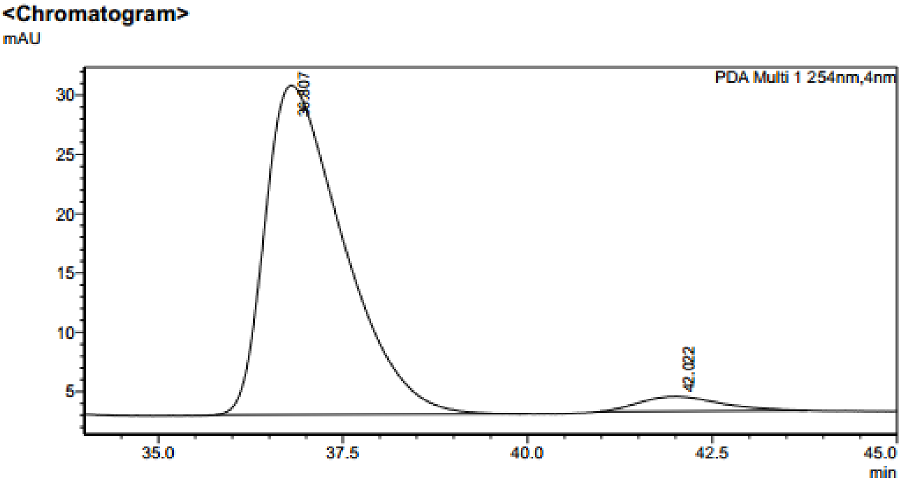

<Peak Table>

PDA Ch1 254nm

Peak#	Ret. Time	Area	Height	Area%
1	36.807	2049299	27773	95.646
2	42.022	93295	1242	4.354
Total		2142595	29015	100.000

Supplementary Fig. 290. HPLC Spectra of **6**

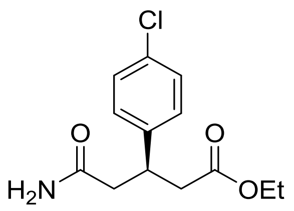

7'

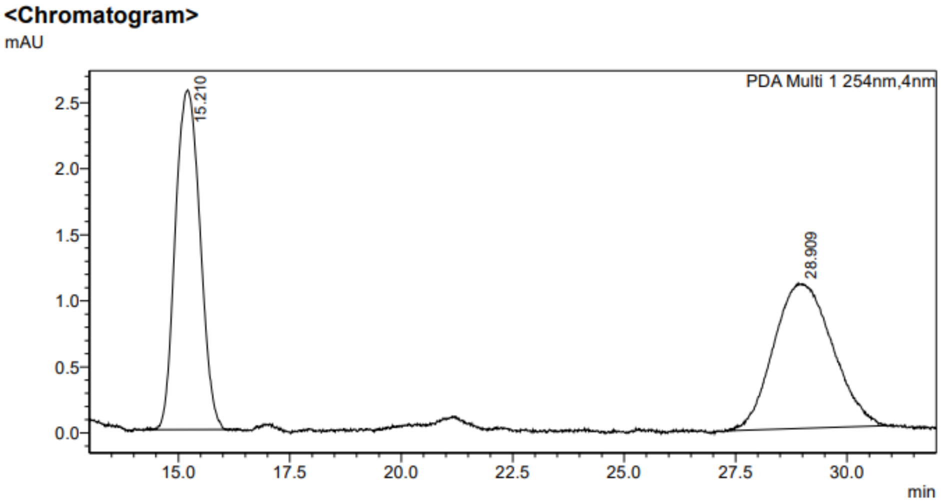

<Peak Table>

PDA Ch1 254nm

Peak#	Ret. Time	Area	Area%	Height
1	15.210	99287	49.641	2571
2	28.909	100722	50.359	1102
Total		200009	100.000	3674

Supplementary Fig. 291. HPLC Spectra of racemic **7’**

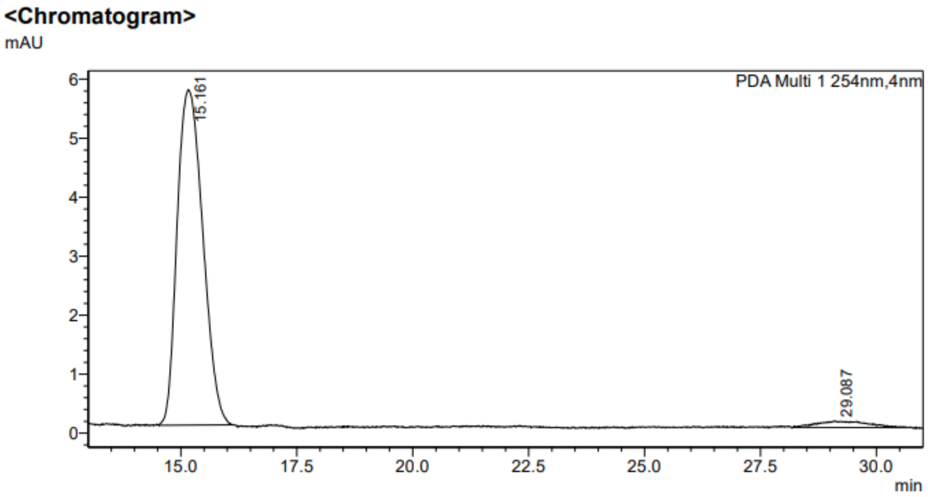

<Peak Table>

PDA Ch1 254nm

Peak#	Ret. Time	Area	Area%	Height
1	15.161	218741	96.455	5685
2	29.087	8039	3.545	109
Total		226781	100.000	5793

Supplementary Fig. 292. HPLC Spectra of **7’**

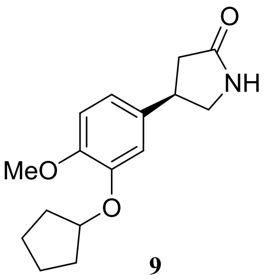

9

<Chromatogram>

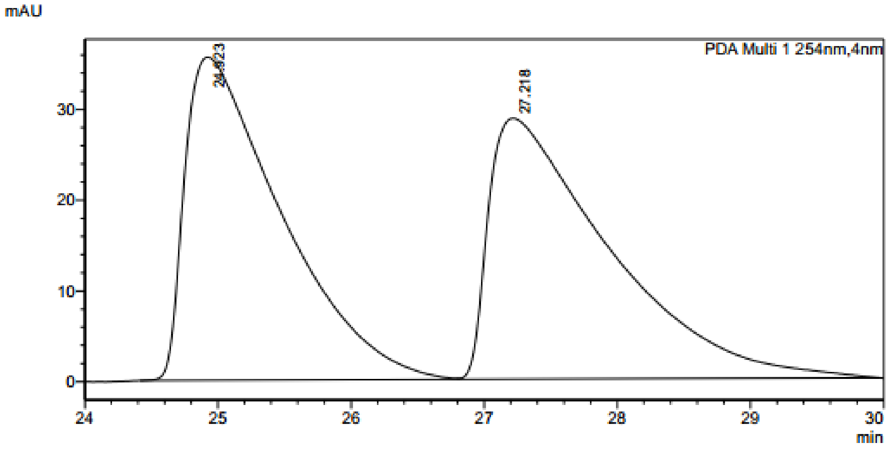

<Peak Table>

PDA Ch1 254nm

Peak#	Ret. Time	Area	Height	Area%
1	24.923	1790426	35595	49.802
2	27.218	1804643	28731	50.198
Total		3595069	64326	100.000

Supplementary Fig. 293. HPLC Spectra of racemic **9**

<Chromatogram>

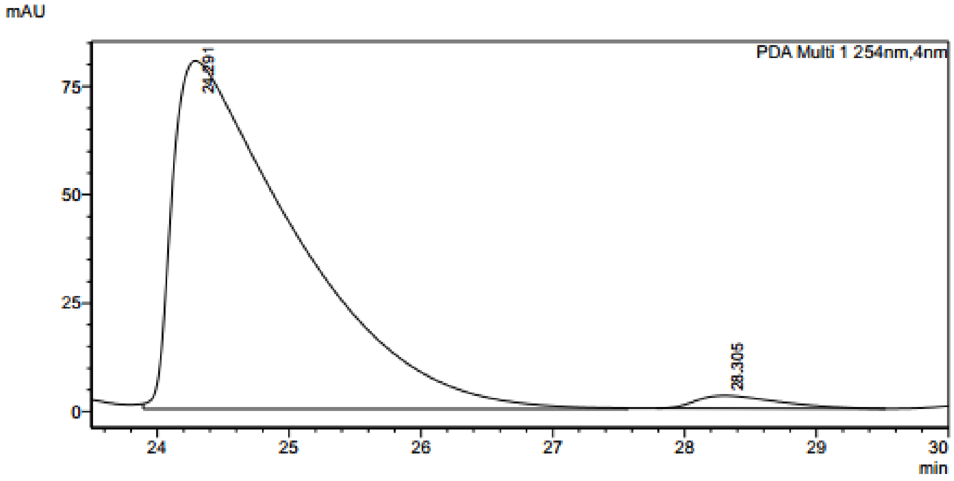

<Peak Table>

PDA Ch1 254nm

Peak#	Ret. Time	Area	Height	Area%
1	24.291	5213871	80299	97.647
2	28.305	125634	2910	2.353
Total		5339506	83210	100.000

Supplementary Fig. 294. HPLC Spectra of **9**

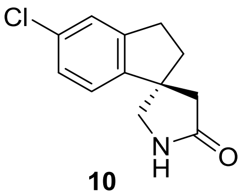

10

<Chromatogram>

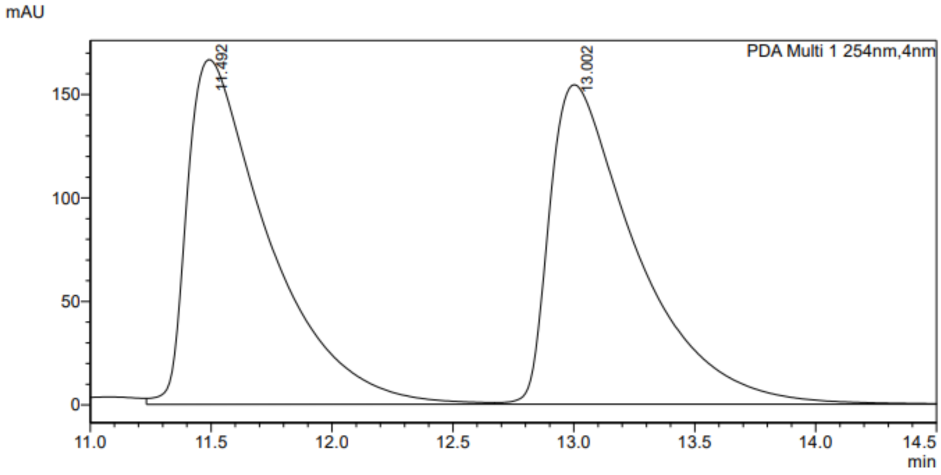

<Peak Table>

PDA Ch1 254nm

Peak#	Ret. Time	Area	Area%	Height
1	11.492	3909894	50.134	166521
2	13.002	3888916	49.866	154451
Total		7798810	100.000	320972

Supplementary Fig. 295. HPLC Spectra of racemic **10**

<Chromatogram>

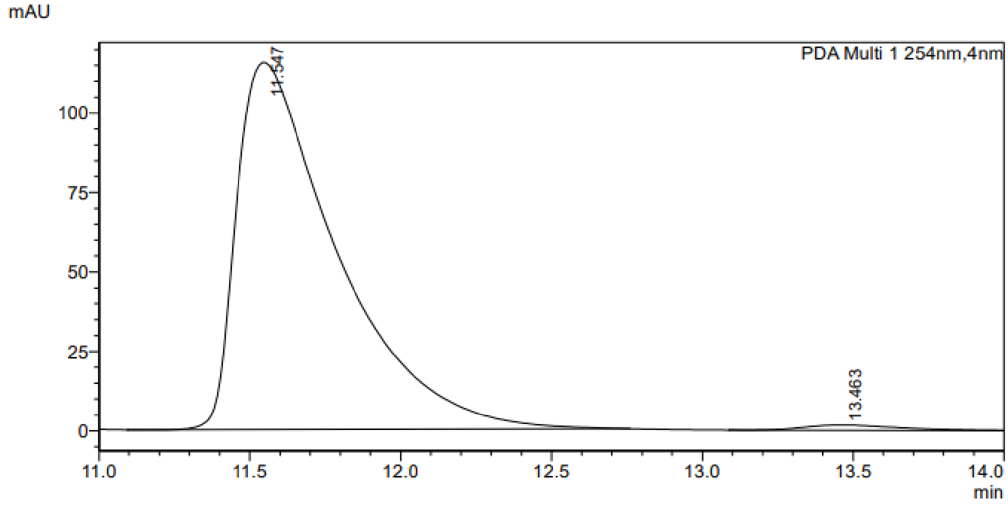

<Peak Table>

PDA Ch1 254nm

Peak#	Ret. Time	Area	Area%	Height
1	11.547	2672147	98.683	115417
2	13.463	35650	1.317	1641
Total		2707796	100.000	117057

Supplementary Fig. 296. HPLC Spectra of **10**

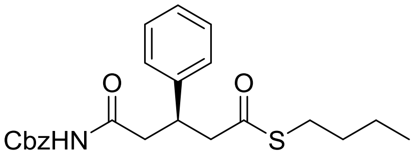

S4

<Chromatogram>

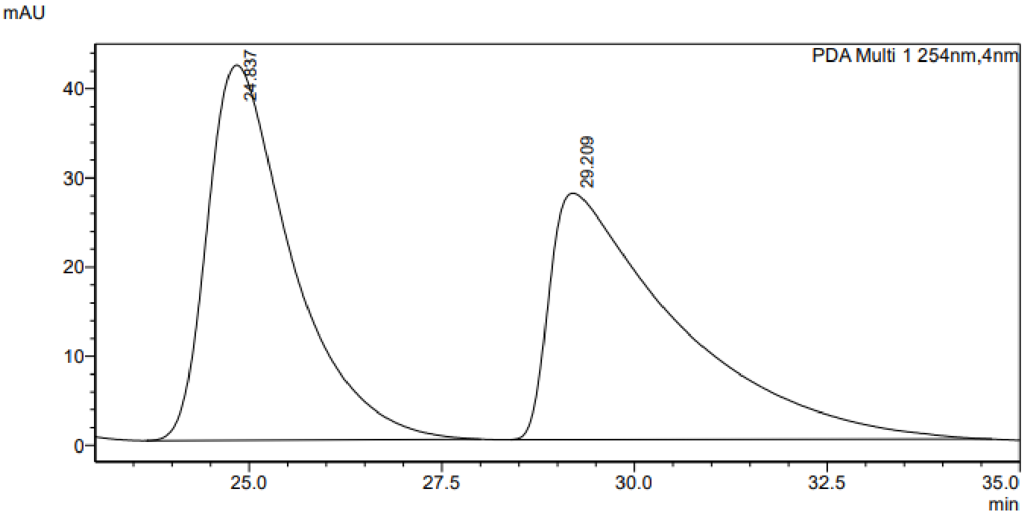

<Peak Table>

PDA Ch1 254nm

Peak#	Ret. Time	Area	Area%	Height
1	24.837	3208654	50.171	42103
2	29.209	3186810	49.829	27655
Total		6395464	100.000	69758

Supplementary Fig. 297. HPLC Spectra of racemic **S4**

<Chromatogram>

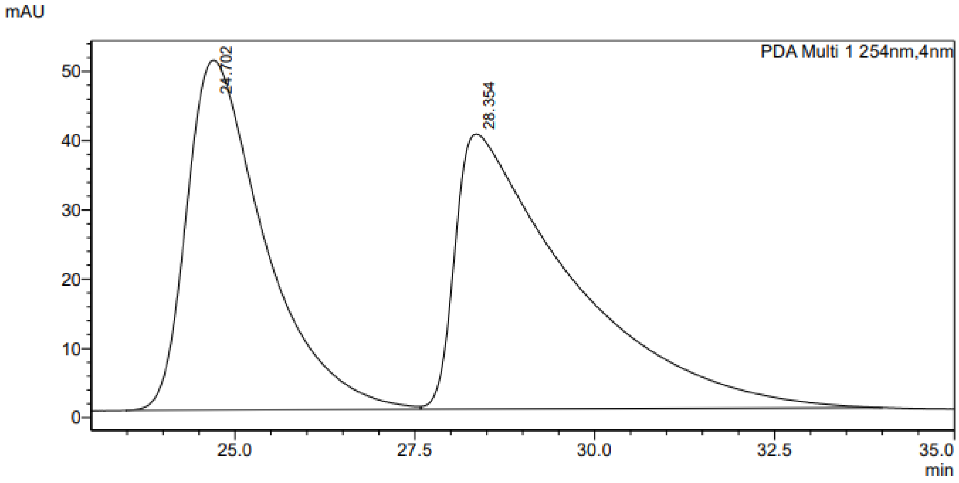

<Peak Table>

PDA Ch1 254nm

Peak#	Ret. Time	Area	Area%	Height
1	24.702	3818579	45.980	50539
2	28.354	4486346	54.020	39715
Total		8304926	100.000	90253

Supplementary Fig. 298. HPLC Spectra of **S4**

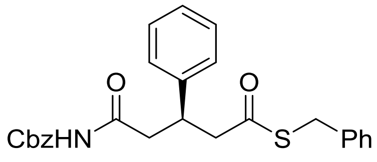

S5

<Chromatogram>

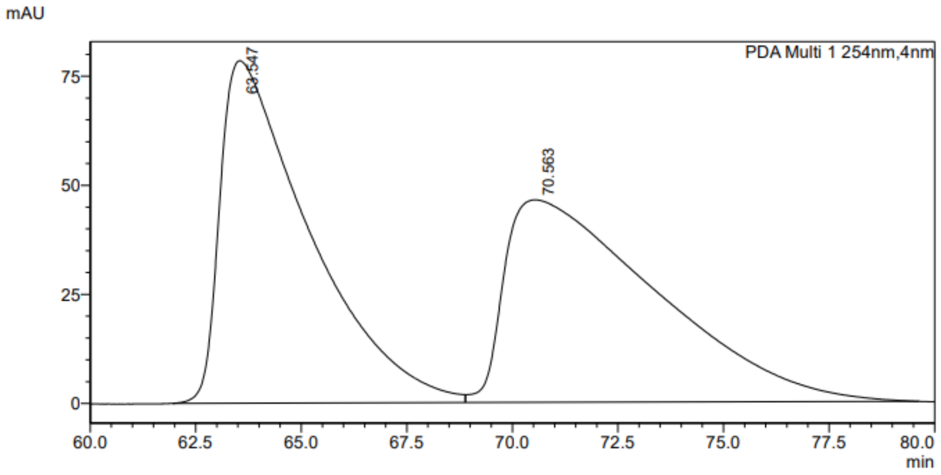

<Peak Table>

PDA Ch1 254nm

Peak#	Ret. Time	Area	Area%	Height
1	63.547	11566141	49.790	78482
2	70.563	11663688	50.210	46425
Total		23229829	100.000	124906

Supplementary Fig. 299. HPLC Spectra of racemic **S5**

<Chromatogram>

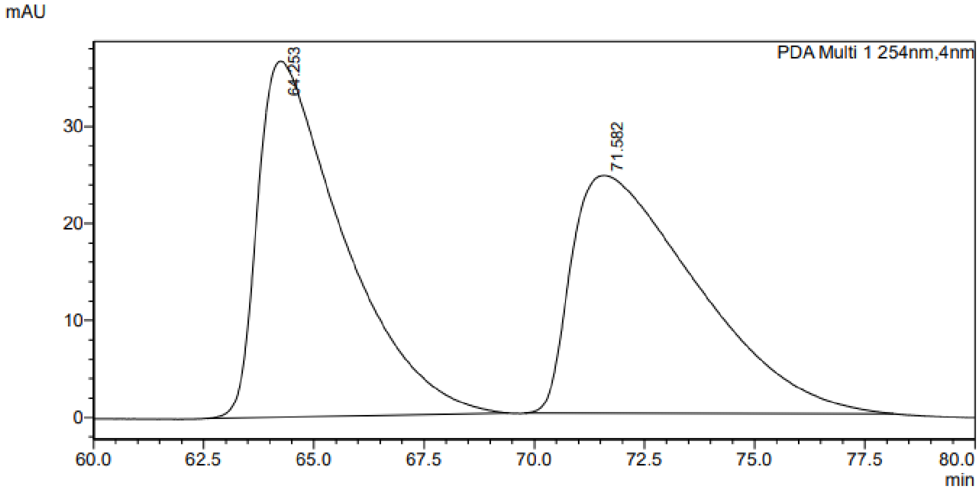

<Peak Table>

PDA Ch1 254nm

Peak#	Ret. Time	Area	Area%	Height
1	64.253	4966070	50.526	36695
2	71.582	4862730	49.474	24498
Total		9828801	100.000	61192

Supplementary Fig. 300. HPLC Spectra of **S5**

5. Computational details

The DFT calculations were performed using the Gaussian 09 program[4]. We have considered and constructed different conformations for the TSs and intermediates, and the conformational searches were performed to select global minima structures using Molclus 1.9[5]. The algorithm was to rotate each rotatable bond by 120° per time, and the generated conformers were prescreened through the UFF force field and then confirmed by DFT calculations, and more results and discussions have been provided as following. All structures were optimized at the M06-2X[6,7]/6-31G(d, p) level in DCM solvent using the integral equation formalism polarizable continuum model (IEF-PCM)[8,9]. Then the frequencies were computed at the same level and used to confirm that all the transition states have only one imaginary frequency and the intermediates have no imaginary frequency. Subsequently, the single-point energies of the stationary points have been refined at the M06-2X/6-311++G(2d, 2p) level, and the discussed energies have been obtained at the theory of M06-2X/6-311++G(2d, 2p)/IEF-PCM_{DCM}//M06-2X/6-31G(d, p)/IEF-PCM_{DCM} level. Based on the ΔG_{gas} reference state (24.46 L at 298.15 K) from 1 atm to 1M, the Gibbs free energy can be computed by using equation $\Delta G_{gas}(1M) = \Delta G_{gas}(1\ atm) + 1.89$ kcal/mol.[10] Furthermore, other different functionals including (*i.e.* B3LYP[11-13]-D3[14] and ωB97XD[15]) were employed to optimize the structures and compute the energy barriers of the *R*- and *S*-conformational transition states, which demonstrates that the selected method and the calculated results are reliable and reasonable for this kind of system.

6. Conformational search

We have shown the selected DFT-optimized structures of the intermediates, transition states, and products as following:

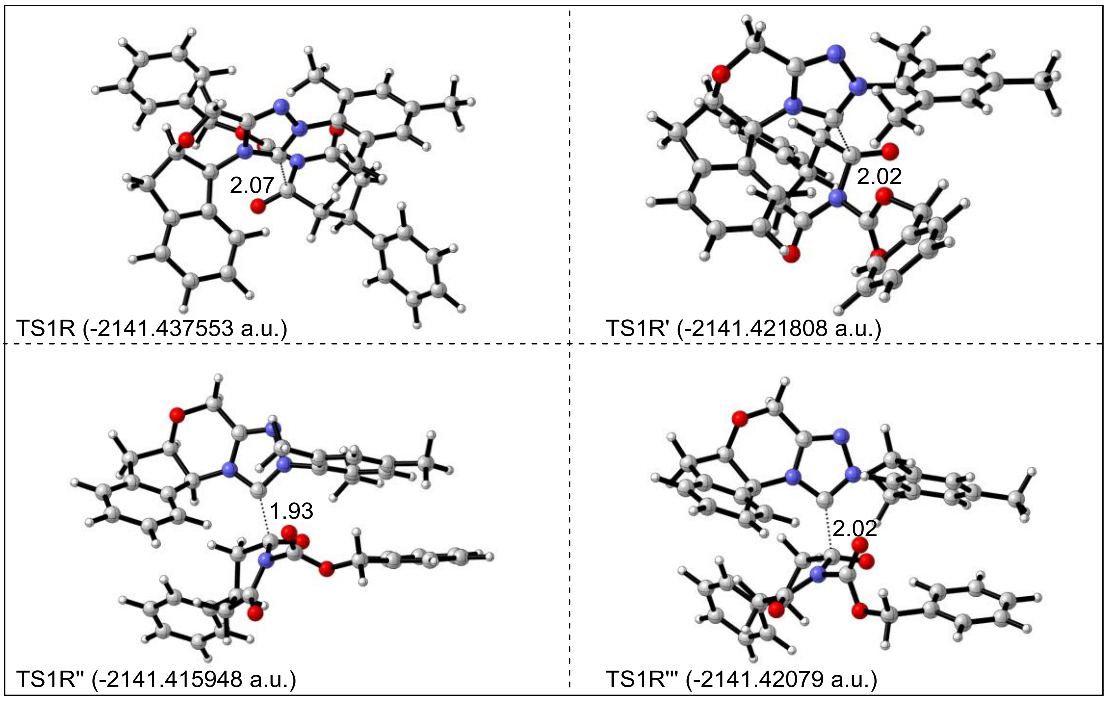

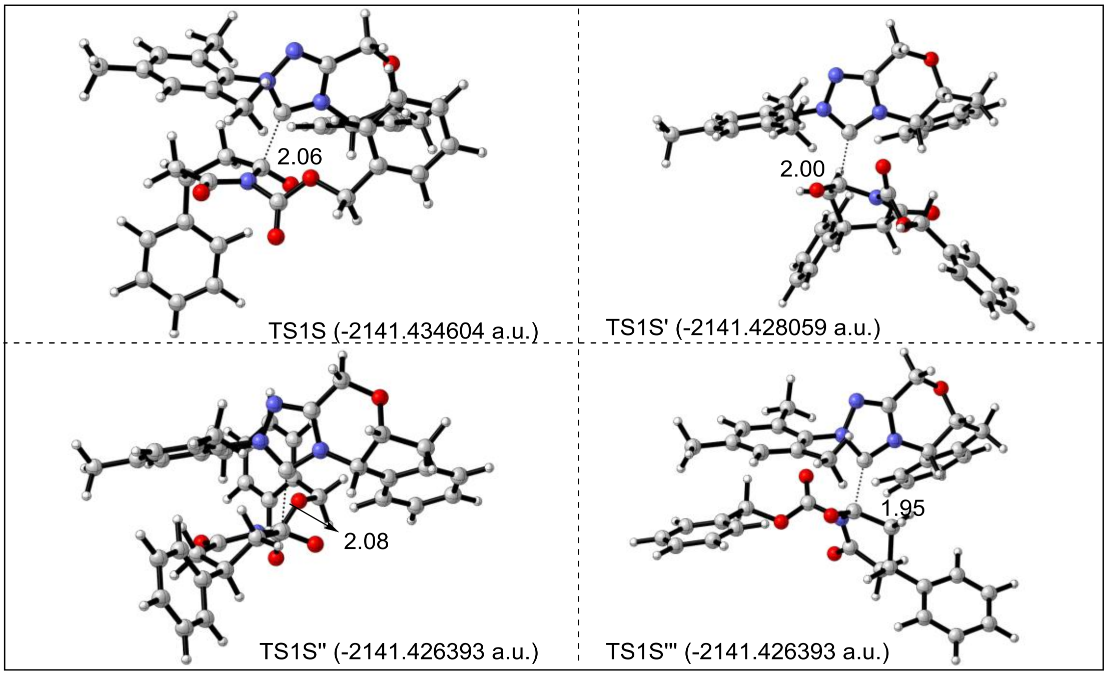

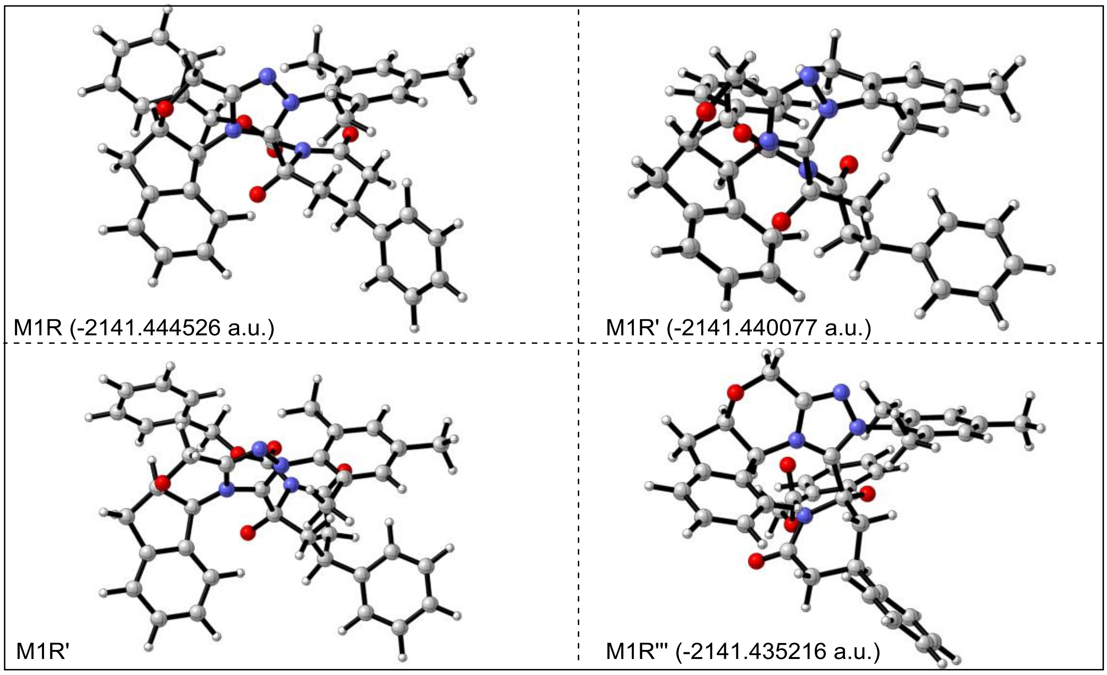
M1R (-2141.444526 a.u.)
M1R' (-2141.440077 a.u.)
M1R'
M1R''' (-2141.435216 a.u.)

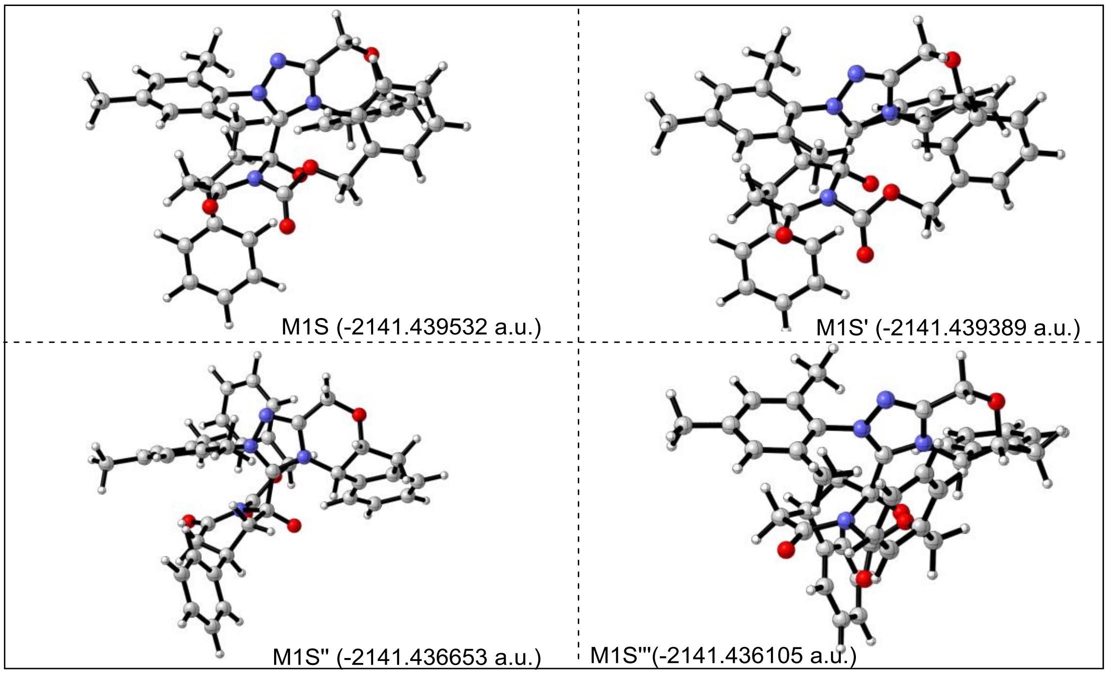
M1S (-2141.439532 a.u.)
M1S' (-2141.439389 a.u.)
M1S'' (-2141.436653 a.u.)
M1S'''(-2141.436105 a.u.)

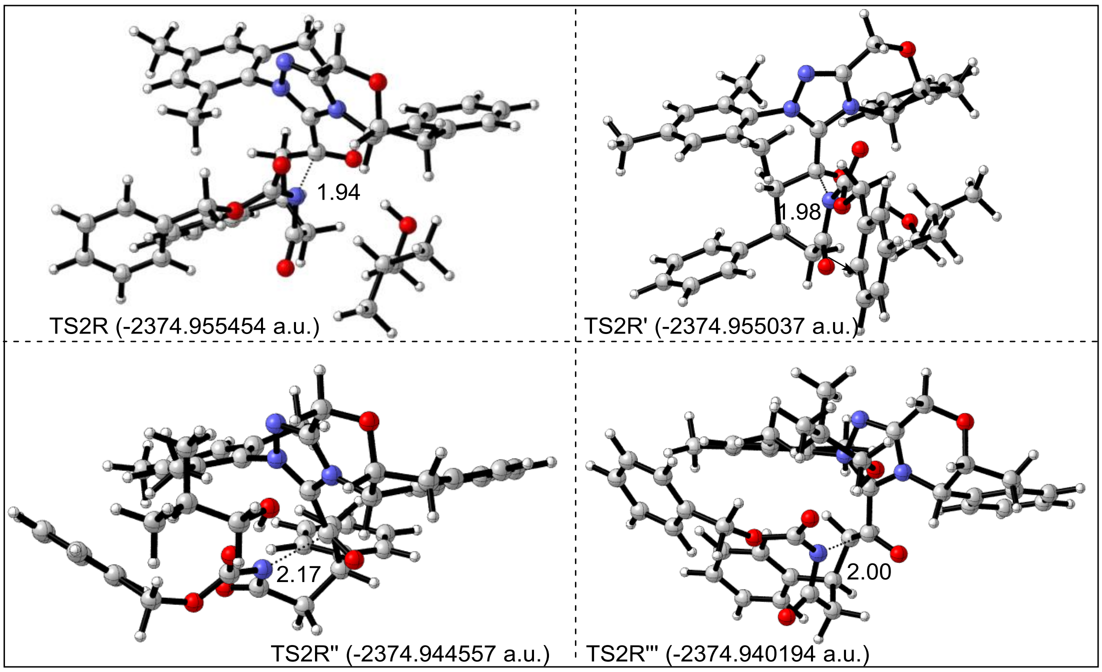
1.94
TS2R (-2374.955454 a.u.)
1.98
TS2R' (-2374.955037 a.u.)
2.17
TS2R'' (-2374.944557 a.u.)
2.00
TS2R''' (-2374.940194 a.u.)

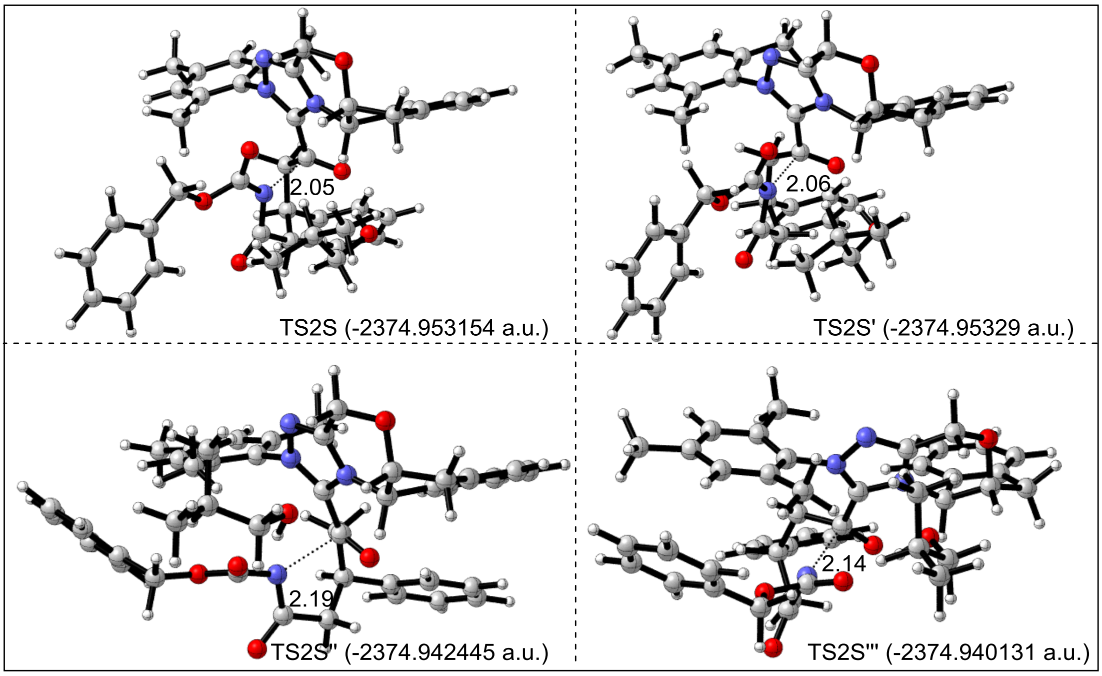
2.05
TS2S (-2374.953154 a.u.)
2.06
TS2S' (-2374.95329 a.u.)
2.19
TS2S'' (-2374.942445 a.u.)
2.14
TS2S''' (-2374.940131 a.u.)

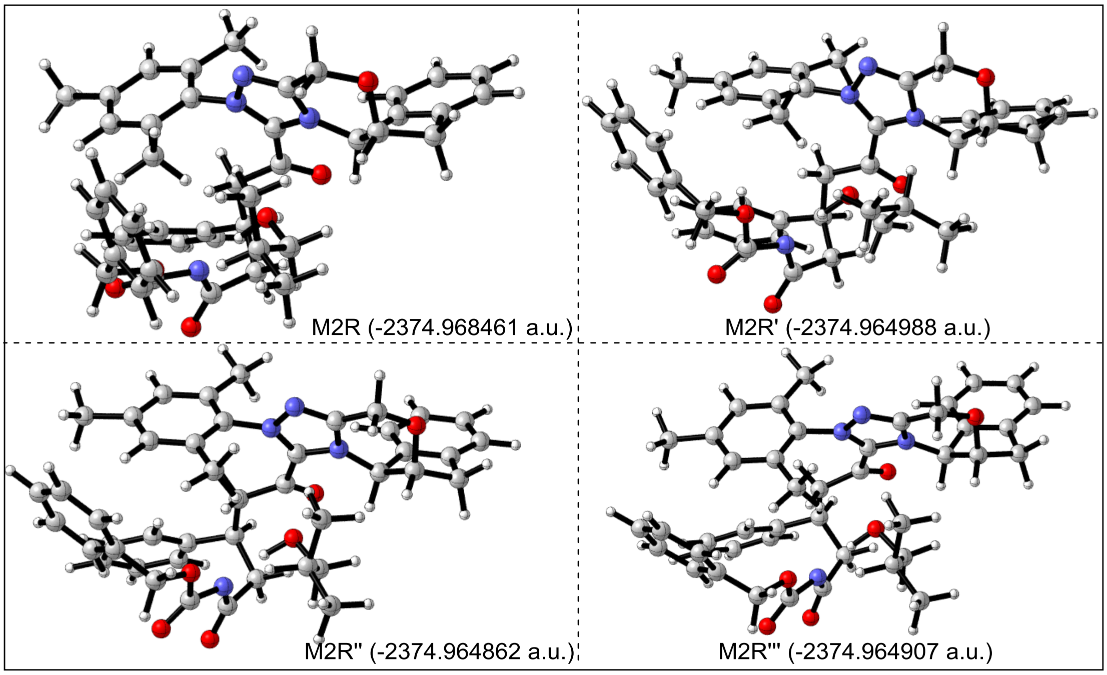
M2R (-2374.968461 a.u.)
M2R' (-2374.964988 a.u.)
M2R'' (-2374.964862 a.u.)
M2R''' (-2374.964907 a.u.)

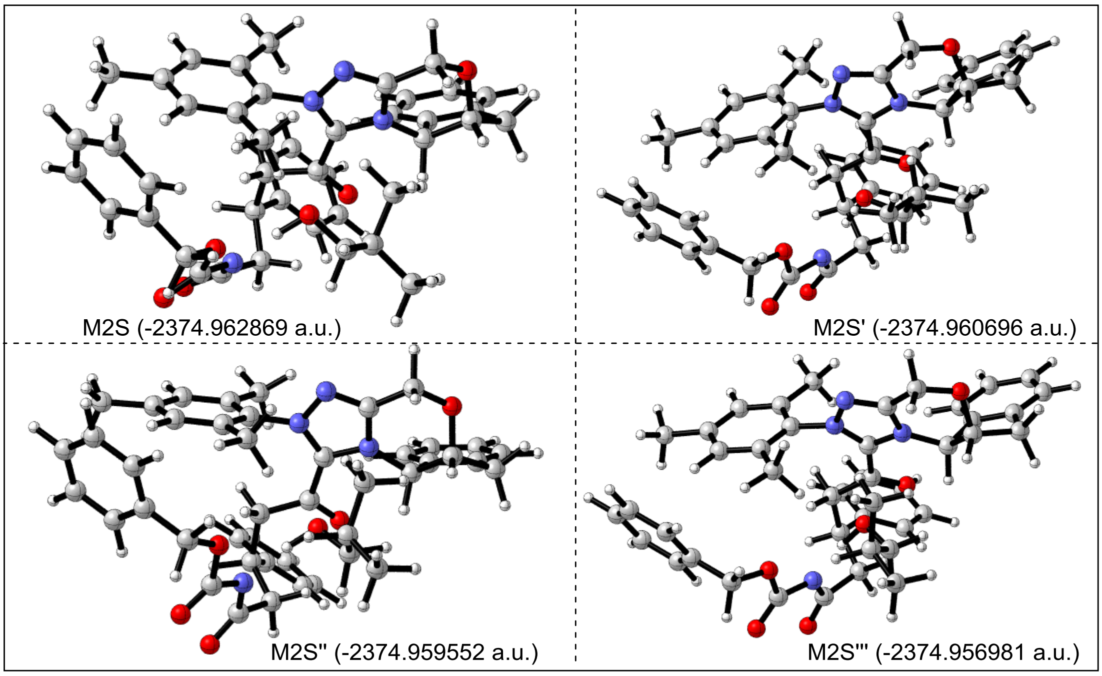
M2S (-2374.962869 a.u.)
M2S' (-2374.960696 a.u.)
M2S'' (-2374.959552 a.u.)
M2S''' (-2374.956981 a.u.)

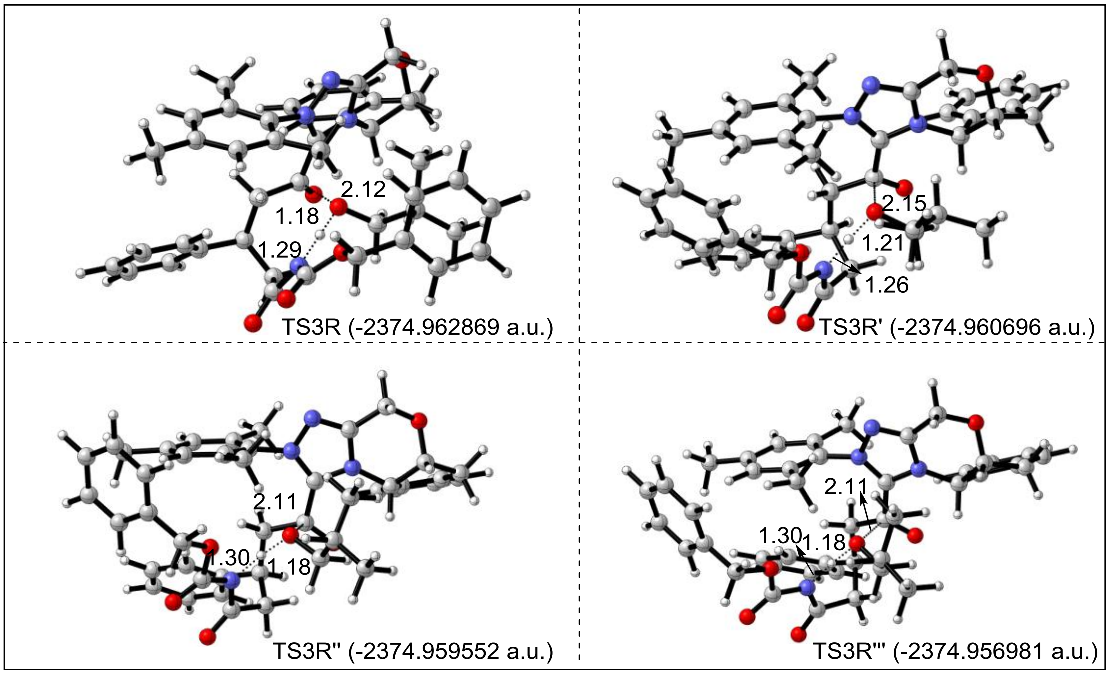
2.12
1.18
1.29
TS3R (-2374.962869 a.u.)
2.15
1.21
1.26
TS3R' (-2374.960696 a.u.)
2.11
1.30
1.18
TS3R'' (-2374.959552 a.u.)
2.11
1.30
1.18
TS3R''' (-2374.956981 a.u.)

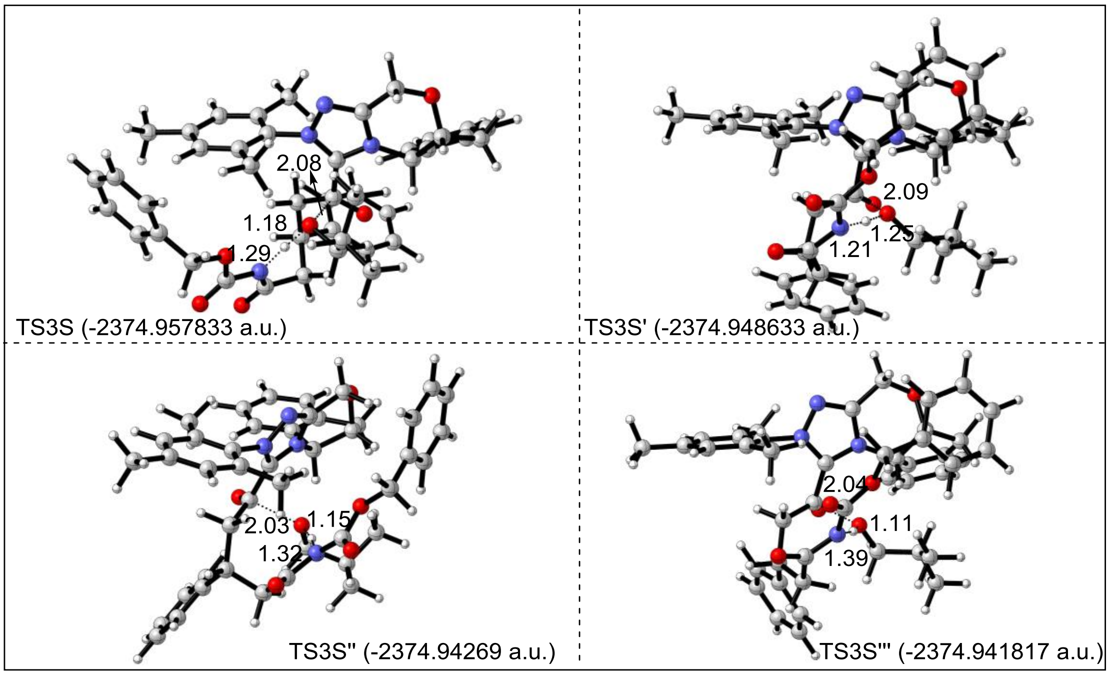
2.08
1.18
1.29
TS3S (-2374.957833 a.u.)
2.09
1.25
1.21
TS3S' (-2374.948633 a.u.)
2.03
1.15
1.32
TS3S'' (-2374.94269 a.u.)
2.04
1.11
1.39
TS3S''' (-2374.941817 a.u.)

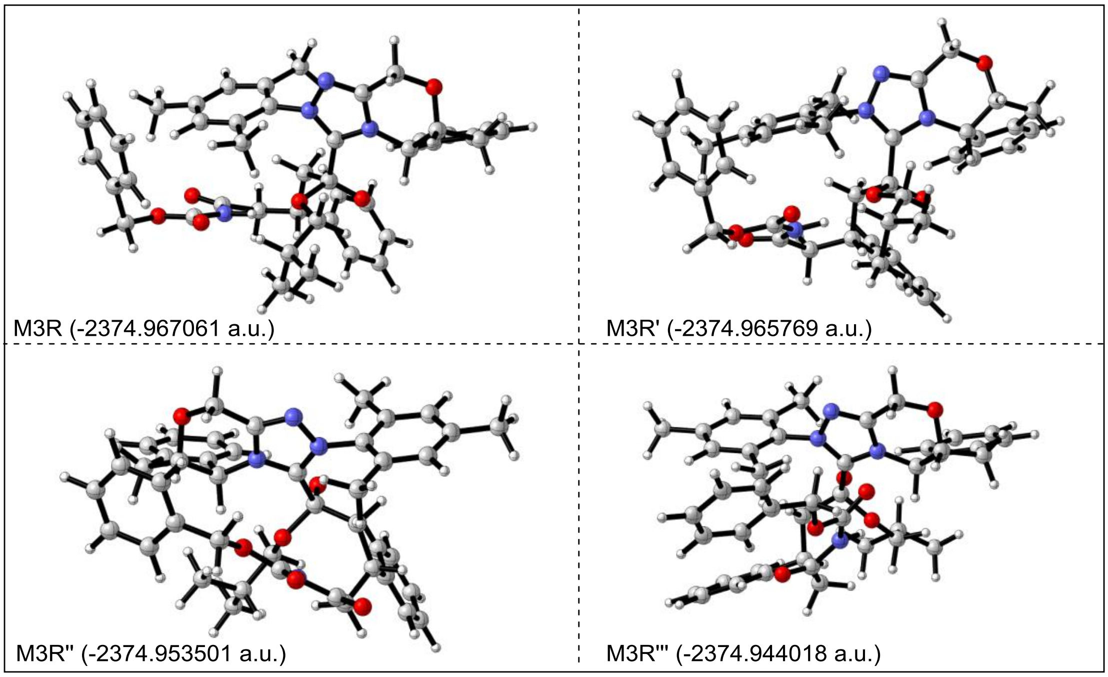
M3R (-2374.967061 a.u.)
M3R' (-2374.965769 a.u.)
M3R'' (-2374.953501 a.u.)
M3R''' (-2374.944018 a.u.)

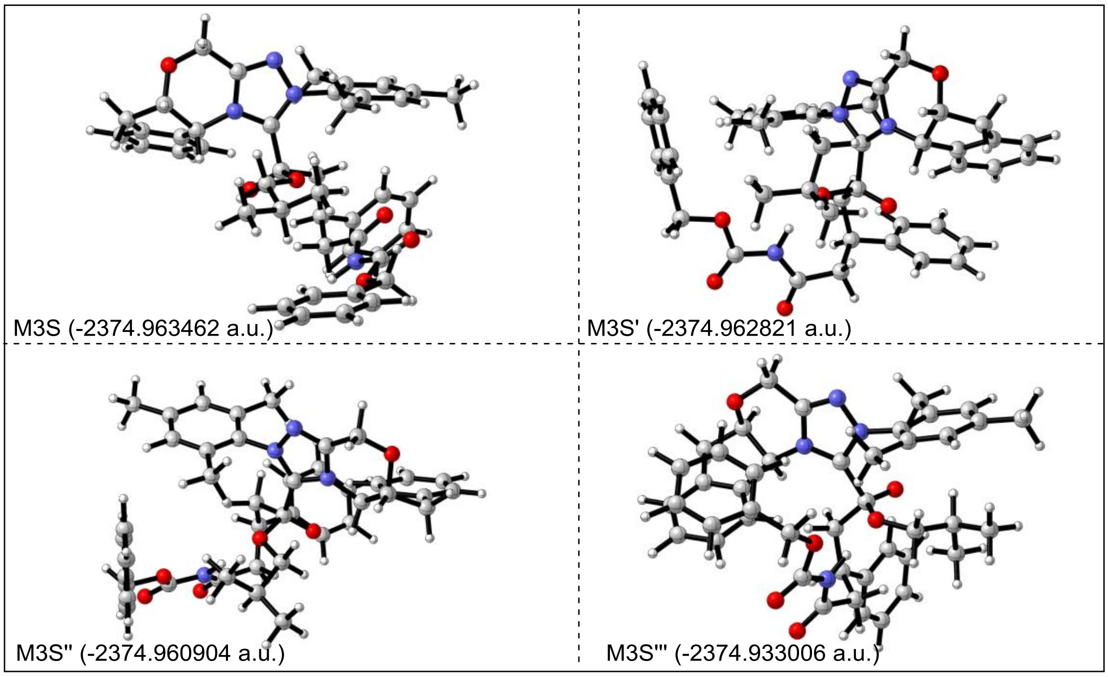
M3S (-2374.963462 a.u.)
M3S' (-2374.962821 a.u.)
M3S'' (-2374.960904 a.u.)
M3S''' (-2374.933006 a.u.)

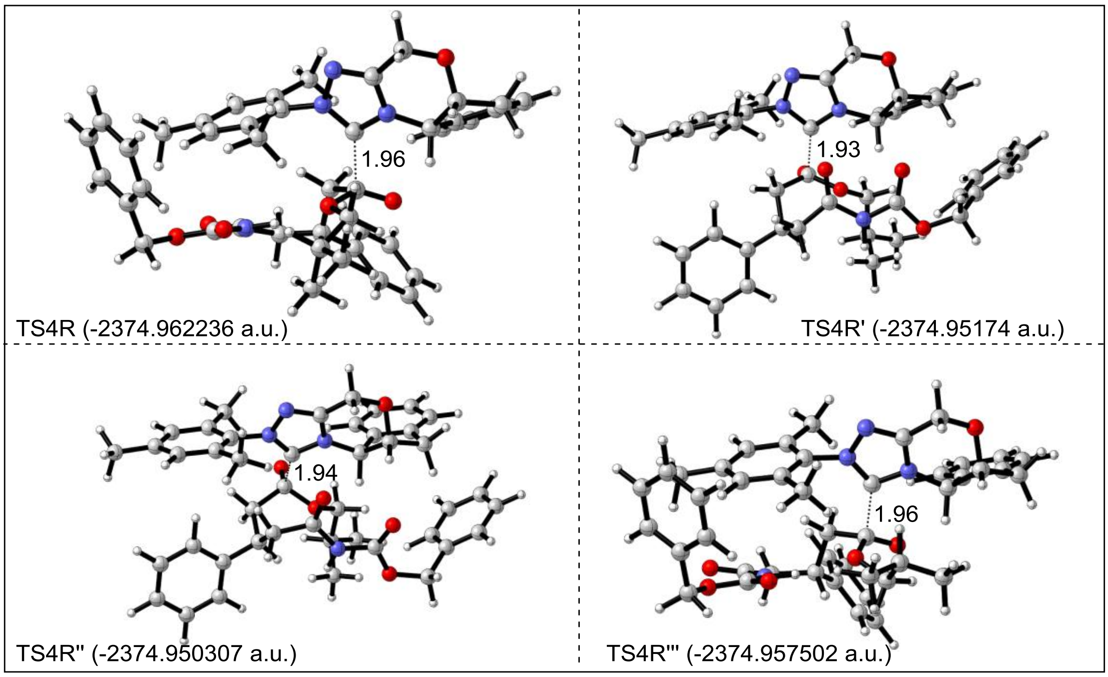
1.96
TS4R (-2374.962236 a.u.)
1.93
TS4R' (-2374.95174 a.u.)
1.94
TS4R'' (-2374.950307 a.u.)
1.96
TS4R''' (-2374.957502 a.u.)

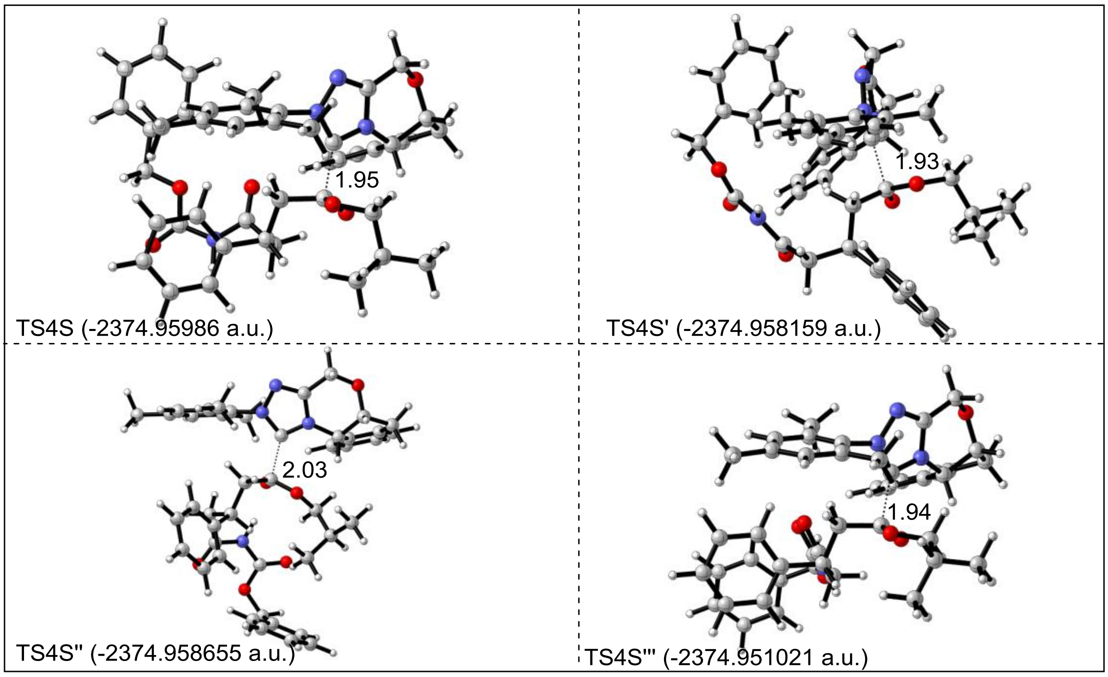
1.95
TS4S (-2374.95986 a.u.)
1.93
TS4S' (-2374.958159 a.u.)
2.03
TS4S'' (-2374.958655 a.u.)
1.94
TS4S''' (-2374.951021 a.u.)

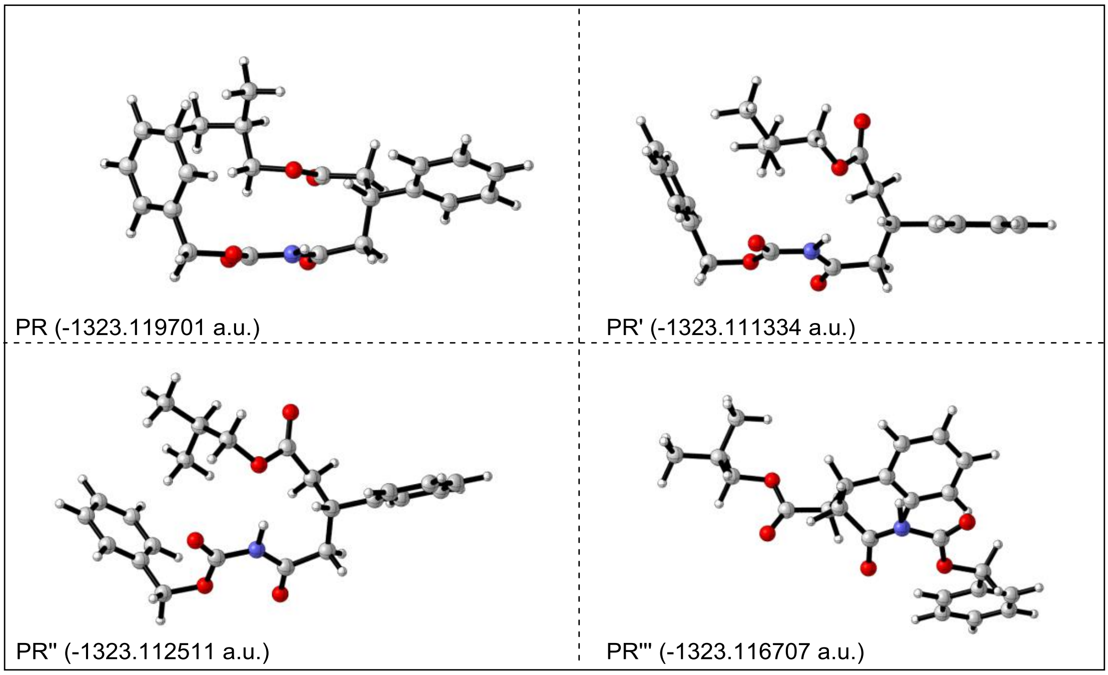
PR (-1323.119701 a.u.)
PR' (-1323.111334 a.u.)
PR'' (-1323.112511 a.u.)
PR''' (-1323.116707 a.u.)

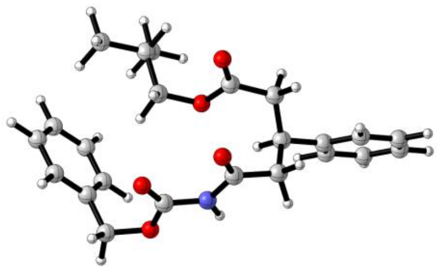

PS (-1323.119525 a.u.)

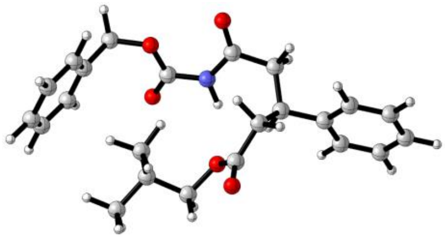

PS' (-1323.11133 a.u.)

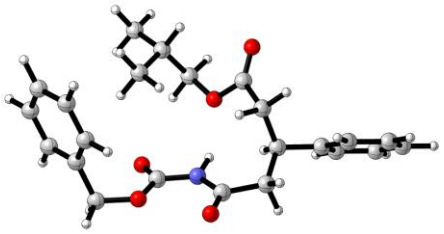

PS'' (-1323.112523 a.u.)

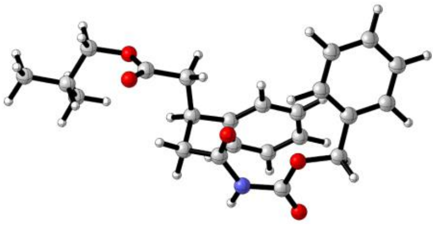

PS''' (-1323.116722 a.u.)

7. Comparisons of the relative Gibbs free energies for selected stationary points at different computational levels

Different functionals including M06-2X, B3LYP-D3 and ωB97XD were employed to optimize the structures and compute the energy barriers of the *R*- and *S*-conformational transition states TS1R and TS1S. As shown in Table 7, the relative Gibbs free energies for the selected stationary points at different computational levels have small differences, and the computed results obtained by using different functionals have the same trend. Therefore, it can be concluded that the selected method and the calculated results are reliable and reasonable for this kind of system.

Supplementary Table 7. Comparisons of the relative Gibbs free energies for selected stationary points at different computational levels Ln = L1~L4 (Unit: kcal/mol)

	L1	L2	L3	L4
TS1R	14.1	11.5	12.1	12.1
TS1S	15.9	14.6	13.5	14.1

L1: M062X/6-311++G(2d, 2p)/IEF-PCM_{DCM}//M062X/6-31G(d, p)/IEF-PCM_{DCM},

L2: B3LYP-D3/6-311++G(2d, 2p)/IEF-PCM_{DCM}//B3LYP/6-31G(d, p)/IEF-PCM_{DCM},

L3: ωB97XD/6-311++G(2d, 2p)/IEF-PCM_{DCM}//ωB97XD/6-31G(d, p)/IEF-PCM_{DCM},

L4: M062X/6-311++G(d, p)/IEF-PCM_{DCM}//M062X/6-31G(d, p)/IEF-PCM_{DCM}.

8. Energy decomposition analyses for key transition states TS3R and TS3S

The energy decomposition analysis was performed and summarized in Supplementary Table 8, the dispersion energies of TS3R and TS23S are -41.3 kcal/mol and -36.7 kcal/mol, respectively. The energies of electrostatics, exchange, and induction of TS3R and TS3S are very close and similar. Therefore, it can be concluded that the interim dispersion should be responsible for the favorability of the transition state TS3R.

Supplementary Table 8. Energy decomposition analyses for key transition states TS3R and TS3S (Unit: kcal/mol)

	Electrostatics	Exchange	Induction	Dispersion
TS3R	-102.4	151.8	-70.8	-41.3
TS3S	-101.6	150.5	-71.2	-36.7

Ⅸ. NCI anayses for key transition states by using NCIplot

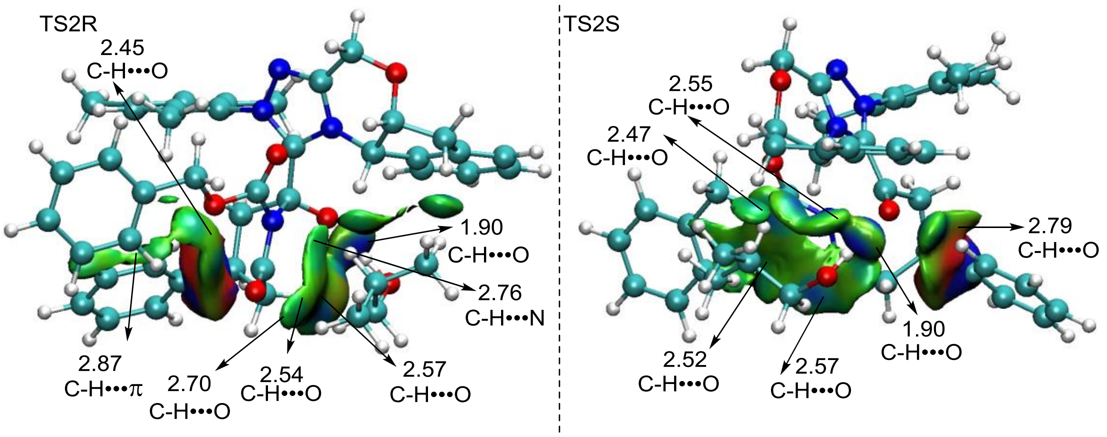

Supplementary Fig. 301. NCI anaysis pictures for key transition state TS2R and TS2S by using NCIplot. (Unit: Å)

As depicted in Fig. 301, there are two C–H···π (2.75, 2.75, and 2.76 Å) and two C–H···O (2.21 and 2.53 Å) interactions in TS3R, while there are one C–H···π (2.79 Å) and two C–H···O (2.23 and 2.29 Å) interactions in TS3S, indicating the strength of C–H···O hydrogen-bond interactions and number of C–H···π interactions should be responsible for the favorability of R-isomer pathway.

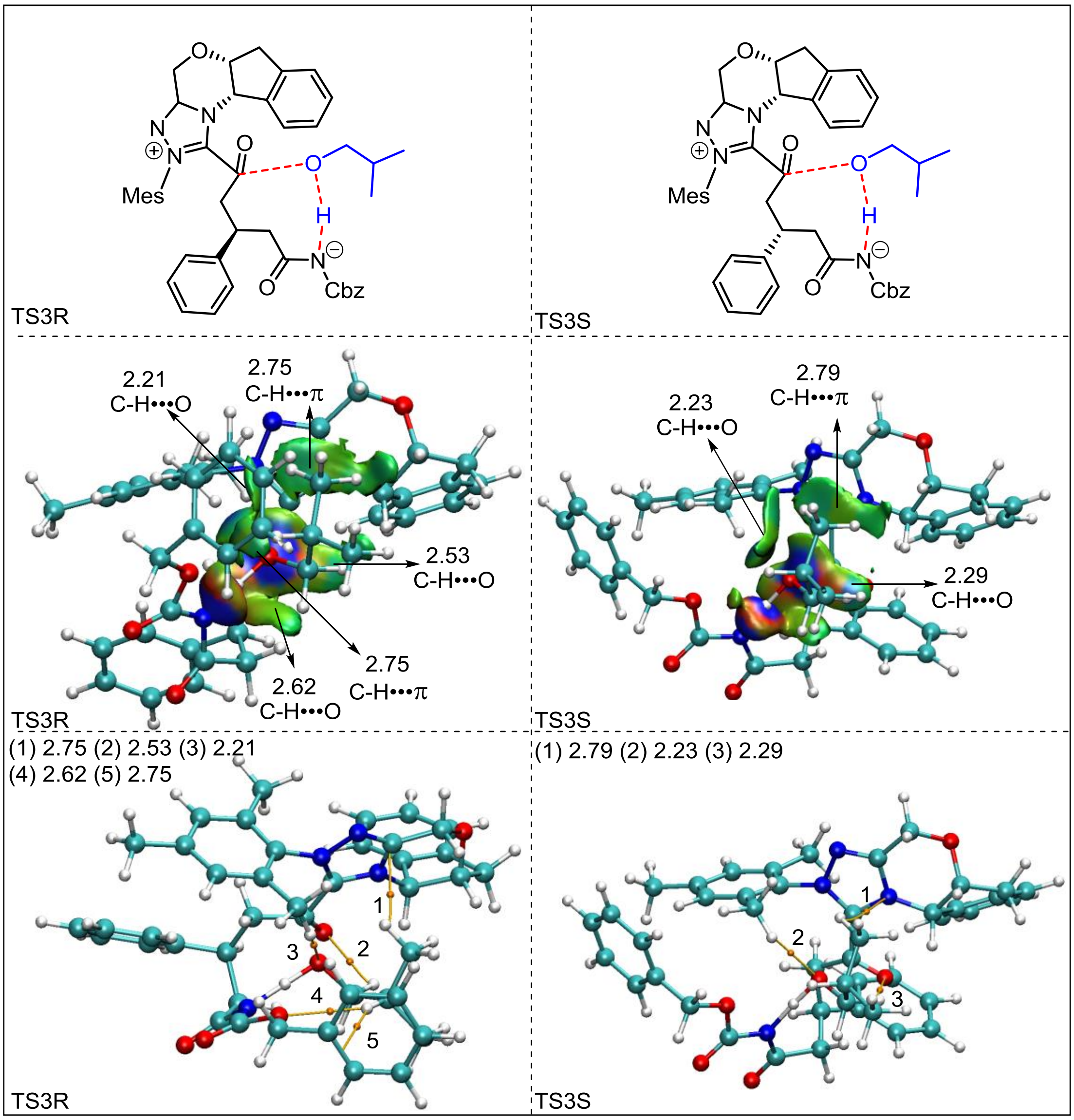

Supplementary Fig. 302. NCI and AIM analyses for key transition states TS3R and TS3S (Unit: Å).

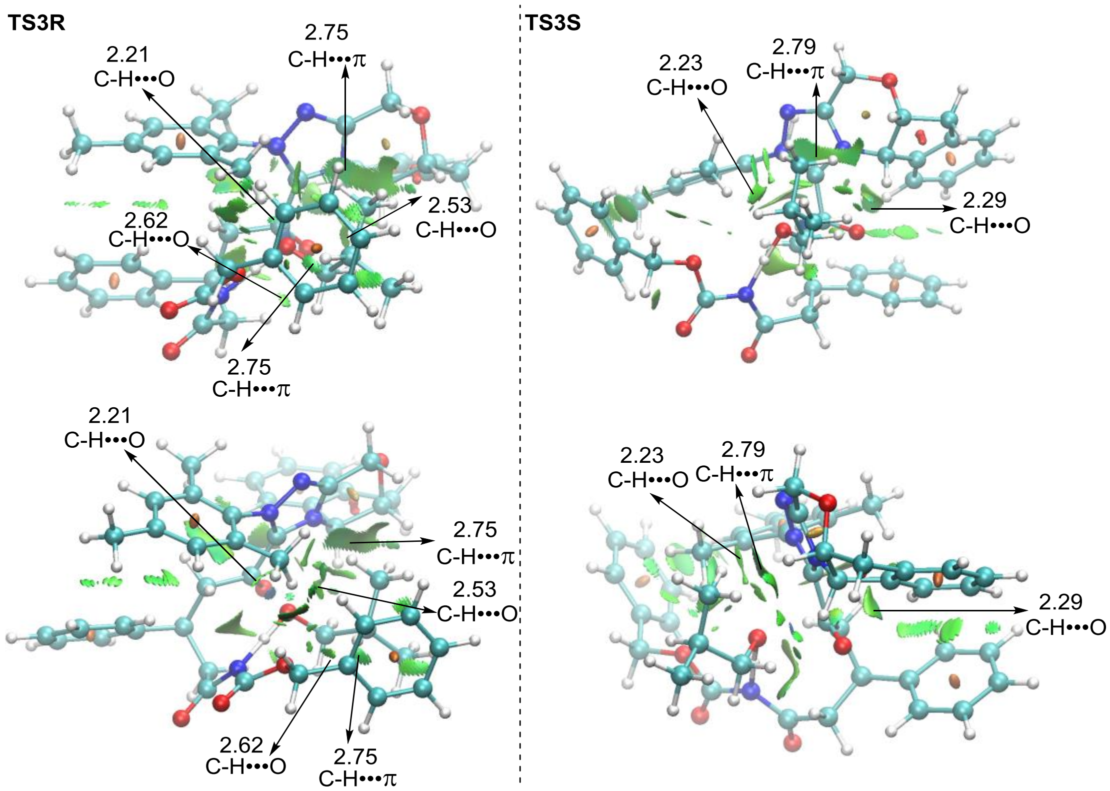

Supplementary Fig. 303. NCI anaysis pictures from differnt views for key transition state TS3R and TS3S by using NCIplot. (Unit: Å)

II. Supplementary references

[1] Y. Chen, X. Q. Xu, X. Liu, B. F. Liu, G. S. Zhang, *Arch. Pharm. Chem. Life Sci.*, **2012**, *345*, 859.

[2] C. Shao, H. J. Yu, N. Y. Wu, P. Tian, R. Wang, C. G. Feng, G. Q. Lin, *Org. Lett*., **2011**, 13, 788.

[3] L. Ji, Y. H. Ma, J. Li, L. R. Zhang, L. H. Zhang, *Tetrahedron.* **2009**, *50,* 6166.

[4] M. J. Frisch, G. W. Trucks, H. B. Schlegel, G. E. Scuseria, M. A. Robb, J. R. Cheeseman, G. Scalmani, V. Barone, B. Mennucci, G. A. Petersson, H. Nakatsuji, M. Caricato, X. Li, H. P. Hratchian, A. F. Izmaylov, J. Bloino, G. Zheng, J. L. Sonnenberg, M. Hada, M. Ehara, K. Toyota, R. Fukuda, J. Hasegawa, M. Ishida, T. Nakajima, Y. Honda, O. Kitao, H. Nakai, T. Vreven, J. A. Montgomery, Jr., J. E. Peralta, F. Ogliaro, M. Bearpark, J. J. Heyd, E. Brothers, K. N. Kudin, V. N. Staroverov, T. Keith, R. Kobayashi, J. Normand, K. Raghavachari, A. Rendell, J. C. Burant, S. S. Iyengar, J. Tomasi, M. Cossi, N. Rega, J. M. Millam, M. Klene, J. E. Knox, J. B. Cross, V. Bakken, C. Adamo, J. Jaramillo, R. Gomperts, R. E. Stratmann, O. Yazyev, A. J. Austin, R. Cammi, C. Pomelli, J. W. Ochterski, R. L. Martin, K. Morokuma, V. G. Zakrzewski, G. A. Voth, P. Salvador, J. J. Dannenberg, S. Dapprich, A. D. Daniels, O. Farkas, J. B. Foresman, J. V. Ortiz, J. Cioslowski, and D. J. Fox, Gaussian 09 Rev. D.01, Inc., Wallingford CT, 2013.

[5] T. Lu, Molclus program, version 1.9, http://www.keinsci.com/research/molclus.html (accessed July 15, 2021)

[6] Y. Zhao, D. G. Truhlar, *Theor. Chem. Acc.* **2008**, *120*, 215.

[7] Y. T. Zhao, D. G. Truhlar, *Acc. Chem. Res*. **2008**, *41*, 157–167.

[8] B. Mennucci, J. Tomasi, *J. Chem. Phys.* **1997**, *106*, 5151–5158.

[9] V. Barone, M. Cossi, *J. Phys. Chem. A*, **1998**, *102*, 1995–2001.

[10] M.D. Liptak, G.C. Shields, *J. Am. Chem. Soc.* **2001**, *123*, 7314-7319.

[11] A. D. Becke. *J. Chem. Phys.* **1993**, *98*, 5648−5652.

[12] C. Lee, W. Yang, and R. G. Parr. *Matter Mater. Phys.* **1988**, *37*, 785−789.

[13] B. Miehlich, A. Savin, H. Stoll, H. Preuss, *Chem. Phys. Lett.* **1989**, *157*, 200−206.

[14] S. Grimme, J. Antony, S. Ehrlich and H. Krieg, *J. Chem. Phys.* **2010**, *132*, 154104.

[15] J.-D. Chai, M, Head-Gordon, *Phys. Chem. Chem. Phys.* **2008**, *10*, 6615-6620.
