# Supplementary material for: Desymmetrization of N-Cbz glutarimides through N-heterocyclic carbene organocatalysis
Source: Nat Commun. 2022 Jul 13;13:4042. doi: 10.1038/s41467-022-31760-z (PMC9279320; doi:10.1038/s41467-022-31760-z)
Supplement: Supplementary file 2 — Description of Additional Supplementary Files [file 41467_2022_31760_MOESM2_ESM.docx]

Description of Additional Supplementary Files

File Name: Supplementary Data 1
Description: Cartesian coordinates and energies of the optimized structures
